# *In silico* capsule locus typing for serovar prediction of *Actinobacillus pleuropneumoniae*

**DOI:** 10.1099/mgen.0.000780

**Published:** 2022-04-11

**Authors:** Siou-Cen Li, Jing-Fang Huang, Yu-Ting Hung, Hsiu-Hui Wu, Jyh-Perng Wang, Jiunn-Horng Lin, Zeng-Weng Chen, Shih-Ling Hsuan

**Affiliations:** ^1^​ Graduate Institute of Veterinary Pathobiology, College of Veterinary Medicine, National Chung Hsing University, Taichung, Taiwan, ROC; ^2^​ Animal Technology Research Center, Agricultural Technology Research Institute, Miaoli, Taiwan, ROC

**Keywords:** *Actinobacillus pleuropneumoniae*, capsule locus typing, genomic analysis

## Abstract

*
Actinobacillus pleuropneumoniae* is a causative agent of pleuropneumonia in pigs of all ages. *A *. *pleuropneumoniae* is divided into 19 serovars based on capsular polysaccharides (CPSs) and lipopolysaccharides. The serovars of isolates are commonly determined by serological tests and multiplex PCR. This study aimed to develop a genomic approach for *in silico A. *pleuropneumoniae** typing by screening for the presence of the species-specific *apxIV* gene in whole-genome sequencing (WGS) reads and identifying capsule locus (KL) types in genome assemblies. A database of the *A *. *pleuropneumoniae* KL, including CPS synthesis and CPS export genes, was established and optimized for Kaptive. To test the developed genomic approach, WGS reads of 189 *A *. *pleuropneumoniae* isolates and those of 66 samples from 14 other bacterial species were analysed. ariba analysis showed that *apxIV* was detected in all 189 *A *. *pleuropneumoniae* samples. These *apxIV*-positive WGS reads were *de novo* assembled into genome assemblies and assessed. A total of 105 *A *. *pleuropneumoniae* genome assemblies that passed the quality assessment were analysed by Kaptive analysis against the *A *. *pleuropneumoniae* KL database. The results showed that 97 assemblies were classified and predicted as 13 serovars, which matched the serovar information obtained from the literature. The six genome assemblies from previously nontypable isolates were typed and predicted as serovars 17 and 18. Notably, one of the two “*Actinobacillus porcitonsillarum*” samples was *apxIV* positive, and its genome assembly was typed as KL03 with high identity and predicted as *A *. *pleuropneumoniae* serovar 3. Collectively, a genomic approach was established and could accurately determine the KL type of *A *. *pleuropneumoniae* isolates using WGS reads. This approach can be used with high-quality genome assemblies for predicting *A *. *pleuropneumoniae* serovars and for retrospective analysis.

## Data Summary

**Impact Statement**

*
Actinobacillus pleuropneumoniae* is an important respiratory pathogen of pigs. The prevalence of *A *. *pleuropneumoniae* serovars varies among countries and areas. Serotyping of *A *. *pleuropneumoniae* isolates provides information on the regional prevalence, which is the basis for disease prevention and control. In recent years, the use of comparative genomic analysis of capsular polysaccharide synthesis genes has helped scientists identify potential new *A *. *pleuropneumoniae* serovars, and molecular typing methods based on PCR have been improved accordingly. Nevertheless, some clinical *A *. *pleuropneumoniae* isolates remained nontypable, indicating that the diversity of the *A *. *pleuropneumoniae* capsule locus (KL) needed further investigation. This study developed a genomic approach to predict serovars through *in silico* KL typing using whole-genome sequencing (WGS) reads and bioinformatics tools from the public domain. This approach provides the compatibility of retrospective analysis and flexibility in an expandable database for new *A *. *pleuropneumoniae* serovars. It is important for increasing awareness toward emerging serovars and can be combined with other WGS-based analysis methods for genomic characterization of isolates.

Supplementary data S1 and S2 and Tables S1 and S2 can be found on FigShare (https://doi.org/10.6084/m9.figshare.19492493) and with the online version of this article. The capsule locus (KL) sequence of *Actinobacillus pleuropneumoniae* serovar 6 strain Femo produced as part of this work is available from the National Center for Biotechnology Information GenBank with accession number MZ450073, and is shown in Supplementary data S2 as the KL06 reference sequence.

## Introduction

*Actinobacillus pleuropneumoniae *, a Gram-negative bacterium, is a causative agent of porcine pleuropneumonia worldwide [1, 2]. *A *. *pleuropneumoniae* is currently classified into 19 serovars based on capsular polysaccharide (CPS, K antigen) and lipopolysaccharide [3, 4]. The main serovar determinant is CPS, production of which requires a functional capsule locus (KL) harbouring CPS synthesis (*cps*) genes and CPS export (*cpx*) genes [3, 5, 6].

Whole-genome sequencing (WGS) is emerging as a new technology for *A *. *pleuropneumoniae* studies, including for the prediction of antimicrobial resistance, identification of new serovars, and investigation of potential targets of drugs and vaccines [4, 7–10]. Through WGS and sequence analysis of *cps* genes, previously nontypable *A *. *pleuropneumoniae* isolates were determined to be new serovars 16–19 [4, 7, 8]. Molecular typing methods using multiplex PCR were modified accordingly to detect serovar-specific *cps* genes and the *A *. *pleuropneumoniae *-specific *apxIV* gene [4, 11]; however, serovars 9 and 11 remain undistinguishable. Comparative analysis of KLs between *A *. *pleuropneumoniae* serovars revealed differences in the coding sequences of proteins participating in CPS biosynthesis [4, 7, 8, 11], implying that *in silico* WGS data analysis of the KL may provide a higher resolution for typing. To the best of our knowledge, using whole-genome sequences to predict serovars has yet to be established in *A *. *pleuropneumoniae *.

*Klebsiella* is an antibiotic-resistant priority pathogen listed by the World Health Organization. Molecular typing of *Klebsiella* has relied on a multiplex PCR method targeting genes involved in the biosynthesis of surface polysaccharides over the past decade [12, 13]. Recently, WGS-based genotyping has been introduced to characterize the molecular types of *Klebsiella *. The genomic typing tool Kaptive, with both command-line and web interfaces, was developed for *Klebsiella* species, especially *Klebsiella pneumoniae *, to identify gene loci associated with biosynthesis of surface polysaccharides in a genome assembly [14, 15]. This WGS-based typing tool was integrated into genomic analysis workflows for pathogen characterization and epidemiological investigation [16, 17]. Kaptive is now used for genotyping *Klebsiella variicola* and *Acinetobacter baumannii* [16, 18], and whether Kaptive can be applied to *A *. *pleuropneumoniae* typing is of interest.

This study aimed to develop a genomic approach for the prediction of *A *. *pleuropneumoniae* serovars. Because genetic variation in the KL leads to diversity of the main serovar determinant, CPS, we tested whether the serovar of *A *. *pleuropneumoniae* isolates can be distinguished and predicted by *in silico* typing of KL sequences in genomes.

## Methods

### *In silico* analysis workflow

**Fig. 1.**
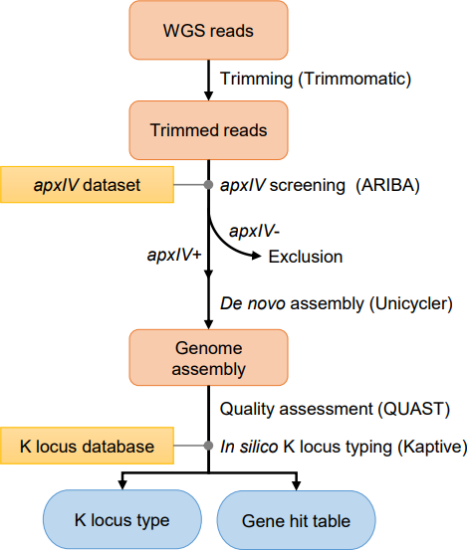
Outline of the *in silico* KL typing of *A *. *pleuropneumoniae* using WGS reads. WGS short-read data were trimmed using Trimmomatic. The trimmed reads were analysed by ariba against the *apxIV* dataset to screen for the presence of the *A *. *pleuropneumoniae *-specific *apxIV* gene. The *apxIV*-positive sample was subjected to *de novo* assembly using Unicycler, and genome assembly quality was analysed using quast. Genome assemblies that passed the quality assessment were analysed using Kaptive against the KL database established in this study. The molecular type of the KL and details of the gene hit were reported by Kaptive and are shown in Tables S1 and S2.

The workflow of *in silico* typing of *A *. *pleuropneumoniae* is outlined in Fig. 1. Briefly, an *apxIV* dataset and a KL database were established. WGS short-read data without serovar information in the Sequence Read Archive (SRA) metadata of *A *. *pleuropneumoniae* and WGS short-read data of non-* A *. *pleuropneumoniae* bacteria were obtained from the National Center for Biotechnology Information (NCBI). Antimicrobial Resistance Identification By Assembly (ariba) and KL typing and variant evaluation (Kaptive) were used as tools to screen *A *. *pleuropneumoniae *-specific *apxIV* in WGS reads and to type the KL in the genome assembly, respectively. A KL type was assigned through Kaptive analysis, and the serovar of the *A *. *pleuropneumoniae* genome was predicted accordingly.

### Establishment of an *apxIV* dataset

To establish an *apxIV* dataset compatible with ariba [19], a coding sequence of ApxIV (GenBank accession number AAD01698.1) with 1,805 amino acids from *A *. *pleuropneumoniae* serovar 1, strain 4074, was used as a reference sequence [20]. An associated metadata file describing information of the reference sequence was prepared to identify the presence or absence of the *apxIV* gene. The *apxIV* sequence and metadata are shown in Supplementary data S1.

### Establishment of an *A *. *pleuropneumoniae* KL database

To establish an *A *. *pleuropneumoniae* KL reference database compatible with Kaptive [15], reference genomes of *A *. *pleuropneumoniae* serovars 1 to 5 and 7 to 12 and publicly available KL sequences of serovars 13 to 19 were collected and downloaded from the NCBI database (Table 1). The region of the full-length KL was identified by analysing the locations of the *modF* and *ydeN* genes (GenBank accession number MG780416.1) at the termini of the KL and the *cpxD* gene (GenBank accession number AIA09380) flanking the *cps* genes as previously described using Basic Local Alignment Search Tool (blast+) v2.7.1 [11, 21]. The full-length KL sequences and annotation information were obtained from the NCBI in GenBank file format (.gbk). By manually deleting the sequence of the mobile element ISApl*1* from the serovar 7 strain AP76 [22], a modified KL sequence with annotation was obtained, and this sequence was used as one of the KL reference sequences for serovar 7.

**Table 1.** Sequences used in the *A *. *pleuropneumoniae* KL database

KL type	GenBank accession no.	Serovar	Strain	No. of CDSs in the KL	Reference
1	CP029003.1	1	4074	14	[43]
2	ADXN01000030.1	2	4226	14	[44]
3	CP000687.1	3	JL03	14	[45]
4	LS483358.1	4	NCTC11384	11	[46]
5	CP000569.1	5	L20	13	[47]
6	MZ450073	6	ATCC 33590	16	This study
7-I*	CP001091.1	7	AP76	13	[48]
7†	CP001091.1†	7	AP76	12	This study
8	LN908249.1	8	MIDG2331	16	[49]
9	ADOI01000049.1	9	CVJ13261	14	[43]
10	ADOJ01000030.1	10	D13039	14	[43]
11	ADOK01000031.1	11	56153	13	[43]
12	ADOL01000042.1	12	1096	10	[43]
13	MG868947.1	13	N273	12	[11]
14	MG868948.1	14	3906	17	[11]
15	MG868949.1	15	HS143	11	[11]
16	MG868950.1	16	A-85/14	13	[11]
17	MG780416.1	17	16287-1	14	[11]
18	MG780423.1	18	7311555	11	[11]
19	MT468887.1	19	7213384-1	13	[4]

CDSs, coding sequences.

*Mobile element ISApl*1* inserted in the KL.

†Sequence modified by deleting the mobile element in the KL reference sequence of KL07-I.

Due to the lack of a full-length serovar 6 KL sequence in the NCBI database, the serovar 6 strain Femo (ATCC 33590), a gift from the Animal Health Research Institute, Council of Agriculture, Executive Yuan, Taiwan, ROC, was subjected to WGS using both Illumina and Nanopore sequencing platforms. Briefly, for Illumina short-read sequencing, genomic DNA was prepared using the Gentra Puregene Yeast/Bact. kit (Qiagen), subjected to library preparation using the Nextera DNA Flex library prep kit (Illumina), and sequenced using an Illumina MiSeq sequencer. The short reads were trimmed using Trimmomatic v0.39 [23]. For Nanopore long-read sequencing, genomic DNA was prepared using the Quick-DNA HMW MagBead kit (Zymo Research), subjected to library preparation using a ligation sequencing kit (Oxford Nanopore Technologies; ONT), and sequenced using an ONT MinION sequencer. The long reads were trimmed by Guppy v4.0.14 (ONT) and filtered by Filtlong v0.2.0 [24]. The long-read data were subsampled and used to generate assemblies by genome assemblers, including Raven v1.1.10, Flye v2.8.1, Miniasm/Minipolish v0.1.3 and Redbean v2.5 [25–28]. The genome assemblies were used to generate a consensus genome using Trycycler v0.3.1 [29]. The consensus genome was polished with long reads by Medaka v1.0.3 (ONT; https://github.com/nanoporetech/medaka) and corrected with short reads by Pilon v1.23 [30]. The KL sequence in the genome of the *A *. *pleuropneumoniae* strain Femo was identified through analysis of the *modF*, *cpxD* and *ydeN* genes as mentioned above and annotated by Prokka v1.14.6 [31]. The KL sequence of the *A *. *pleuropneumoniae* serovar 6 strain Femo was deposited in the GenBank database with the accession number MZ450073.

A GenBank format file, including nucleotide sequences and annotations of coding sequences for reference KL types 1 to 19, was prepared with gene nomenclature consistent with that described by Bossé *et al*. [11]. Descriptions of KL types were added in the source feature of the GenBank file for compatibility with Kaptive (Supplementary data S2).

### Collection and pre-processing of WGS short reads

Publicly available WGS reads of 189 *A *. *pleuropneumoniae* isolates and those of 66 samples from 14 non-* A *. *pleuropneumoniae* bacterial species derived from Illumina paired-end sequencing were obtained from the SRA database hosted at the NCBI. The SRA run accession numbers for WGS reads of the *A *. *pleuropneumoniae* and non-* A *. *pleuropneumoniae* bacterial species are listed in Tables 2 and S1, respectively. The SRA files were processed using SRA Toolkit v2.8.2-1 (NCBI) to obtain the raw WGS reads. For further analysis, the raw WGS reads were trimmed at a Phred quality score of Q30 and removed if they were shorter than 30 nucleotides using Trimmomatic v0.39 [23].

**Table 2.** WGS reads of non-* A *. *pleuropneumoniae* bacterial species used in this study

Species	No. of samples (*n*=66)	SRA run accession no.
**Porcine bacterial pathogens**	46	–
“Actinobacillus porcitonsillarum”*	2	ERR200086, ERR200087
* Actinobacillus suis* *	4	SRR5184352, SRR5189134, SRR5189141, SRR5189321
* Bordetella bronchiseptica *	5	SRR931866, SRR942675, SRR9614213, SRR9614214, SRR9614215
* Escherichia coli* *	5	SRR10099931, SRR10099940, SRR10099946, SRR11647626, SRR9619978
* Glaesserella parasuis *	5	ERR175964, ERR176010, ERR176017, ERR225607, ERR270808
* Mycoplasma hyopneumoniae *	5	SRR7601664, SRR7601670, SRR7601671, SRR7601681, SRR7601682
* Pasteurella multocida *	5	SRR13148928, SRR13148936, SRR13148937, SRR13148946, SRR13148947
* Salmonella enterica* serovar Choleraesuis	5	ERR1777400, ERR1777414, ERR1777421, ERR1777449, ERR1777457
* Salmonella enterica* serovar Typhimurium	5	SRR2015698, SRR2015925, SRR2075991, SRR8291813, SRR9879548
* Streptococcus suis *	5	SRR4431635, SRR4431639, SRR4431646, SRR4431671, SRR5177695
**Others**	20	–
* Aggregatibacter actinomycetemcomitans* *,†	3	SRR12066794, SRR3170532, SRR3947678
* Bordetella pertussis* *,†	5	SRR11855995, SRR12105040, SRR5080696, SRR8689258, SRR9118293
* Mannheimia haemolytica* *,‡	5	SRR3749365, SRR3749458, SRR3750210, SRR3767545, SRR3775504
* Moraxella bovis* *.‡	2	SRR11012145, SRR7431214
* Proteus vulgaris* *,†	5	ERR4014498, ERR4014630, SRR10728100, SRR13191024, SRR13191127

*RTX toxin-producing bacteria.

†Human pathogens.

‡Bovine pathogens.

### Screening of *apxIV* in short-read data

The presence of the *apxIV* gene in trimmed reads of *A *. *pleuropneumoniae* and non-* A *. *pleuropneumoniae* bacteria was analysed by ariba v2.14.6 [19], which mapped and assembled the reads with default settings against the *apxIV* dataset. Gene hits were reported when the alignment identity was ≥90 %. The trimmed reads positive for *apxIV* were used for *de novo* genome assembly.

### *De novo* genome assembly and quality assessment

The trimmed reads were *de novo* assembled using Unicycler v0.4.4 with an Illumina-only assembly pipeline [32]. Assembly statistics were analysed using quast v5.0.2 [33], and the quality of the genome assemblies was assessed. Genome assemblies with a minimum assembly length of 2 Mbp and a maximum contig number of 200 were subjected to manual inspection for the number and accumulative length of large contigs. Genome assemblies that passed the quality assessment were subjected to KL typing.

### KL typing

The genome assemblies were analysed by Kaptive v0.7.3 against the *A *. *pleuropneumoniae* KL database with the default setting, and the best matching reference KL for each query genome assembly and a corresponding confidence level based on a blastn search and the number of genes were reported [15]. The match confidence was divided into six levels, namely, 'perfect', 'very high', 'high', 'good', 'low' and 'none', and the criteria for each level have been described elsewhere [14]. Except for the confidence level of none, all the other five levels were acceptable for serovar determination.

## Results

### *A*. *pleuropneumoniae* KL database establishment

For KL typing of the genome assemblies, 20 KL sequences were used as reference sequences in the *A *. *pleuropneumoniae* KL database covering all known serovars 1 to 19 (Table 1). Each KL type was designated according to the serovar information of the reference sequences. To coordinate with Kaptive, each KL type was designated as KL and a number, e.g. KL01 to KL19, and a KL reference sequence was assigned to each KL type. The KL harbouring the insertion mobile element ISApl*1*, denoted ‘KL07-I’, was from *A *. *pleuropneumoniae* serovar 7 reference strain AP76 [22], while KL07 represented the modified reference sequence of KL07-I lacking ISApl*1*. The number of coding sequences in each KL ranged from 10 to 17 (Table 1). Based on a correspondence between KL type and serovar information of the reference sequence, the *A *. *pleuropneumoniae* serovar could be predicted accordingly, e.g. the query genome with a KL type of ‘KLx’ was predicted as ‘serovar x’.

### Screening of *apxIV* in WGS reads of *A *. *pleuropneumoniae* and non-* A *. *pleuropneumoniae* bacterial species

**Fig. 2.**
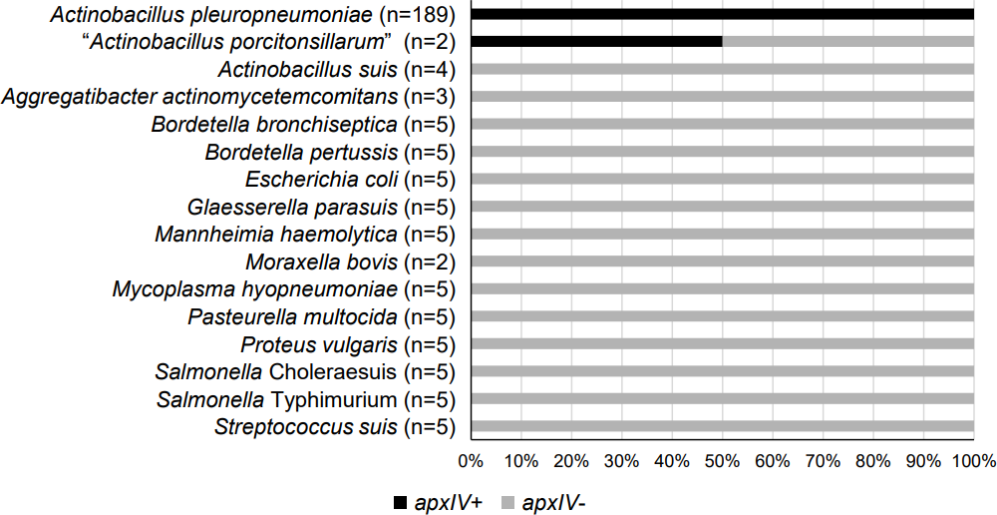
Screening of *apxIV* using WGS short-read data. The trimmed WGS short-read data of 189 *A *. *pleuropneumoniae* and those from 14 non-* A *. *pleuropneumoniae* bacterial species were analysed by ariba against the *apxIV* dataset. The presence of *apxIV* was reported, and the percentages of *apxIV*-positive samples in each bacterial species were calculated.

To assist with *A *. *pleuropneumoniae* typing and distinguish this species from other bacterial species, *apxIV* was used as an *A *. *pleuropneumoniae *-specific genetic marker [20]. The presence or absence of the *apxIV* gene in WGS reads of 189 *A *. *pleuropneumoniae* samples and those of 66 samples from 14 non-* A *. *pleuropneumoniae* bacterial species (Table 2) was screened using ariba against the *apxIV* dataset (Supplementary data S1). The presence of *apxIV* was detected in all the *A *. *pleuropneumoniae* samples tested (Fig. 2), indicating a sensitivity of 100 % (189/189) using the WGS-based identification of *A *. *pleuropneumoniae *. Moreover, the WGS reads from porcine, bovine and human bacterial pathogens, except for one “*Actinobacillus porcitonsillarum*” sample (SRA run accession number ERR200087), indicated that these organisms were *apxIV* negative (65/66). The *apxIV*-positive “*A. porcitonsillarum*” was subjected to further analysis. The results showed that the *apxIV* screening strategy distinguished the WGS reads of *A *. *pleuropneumoniae* from those of the non-* A *. *pleuropneumoniae* bacterial species.

### *De novo* genome assembly of *A *. *pleuropneumoniae* WGS reads

After confirming the presence of *apxIV* in *A *. *pleuropneumoniae* short-read data, the trimmed reads were subjected to *de novo* genome assembly, and the assembly quality was assessed. A total of 105 of the 189 *A *. *pleuropneumoniae* assemblies passed quality assessment with a median contig number of 62 and a median assembly length of 2.26 Mbp. In this study, 32 % (31/98) and 81 % (74/91) of the genome assemblies derived from the paired-end 75 and 100 bp reads, respectively, passed quality assessment.

### KL typing of the *A *. *pleuropneumoniae* genome assembly and interpretation

**Fig. 3.**
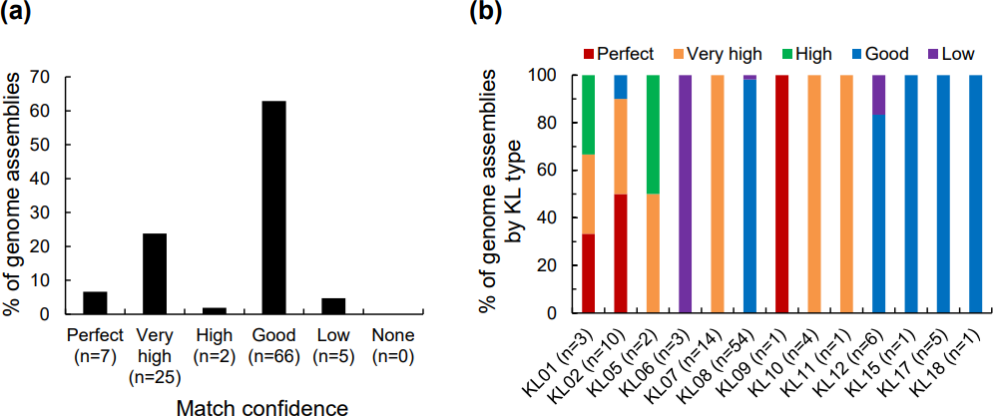
*In silico* KL typing of *A *. *pleuropneumoniae* genome assemblies. The 105 *A *. *pleuropneumoniae* genome assemblies that passed the quality assessment were subjected to KL typing by Kaptive against the *A *. *pleuropneumoniae* KL database. The match confidence and KL types of the genome assemblies were reported by Kaptive. (**a**) Distribution of the match confidence of the assigned KL types of the 105 genomes. (**b**) Distribution of the match confidence by KL types.

The 105 *A *. *pleuropneumoniae* genome assemblies were analysed by Kaptive analysis for KL type and serovar prediction. The results showed that the match confidence of 95 % of the tested genomes (100/105) was good to perfect (Fig. 3a), indicating that a KL type was assigned to these genomes with high confidence. Thirteen KL types, including KL types 01, 02, 05–12, 15, 17 and 18, were assigned to the tested genomes (Fig. 3b). The most assigned KL types were KL08, with 53 good matches and a low match, and KL07, with 14 very high matches. The KL types of the 105 genome assemblies and the detailed results reported by Kaptive, including match confidence, coverage, identity, discrepancy in KL length and a full list of identified genes with identities, missing genes and extra genes, are shown in Tables 3 and S1.

**Table 3.** KL types and predicted serovars of the 105 *A *. *pleuropneumoniae* genome assemblies

KL type/predicted serovar	No. of test genomes	Serovar information from the literature (*n*) [reference]	Correspondence
1	3	1 (3) [50]	+
2	10	2 (9) [9, 50], K2:O7 (1) [50]	+
5	2	5 (2) [50]	+
6	3*	6 (2) [9, 50]	+
7	14	7 (14) [9, 50]	+
8	54*	8 (53) [9, 49, 50]	+
9	1	9 (1) [50]	+
10	4	10 (4) [50]	+
11	1	11 (1) [50]	+
12	6	12 (6) [50]	+
15	1	15 (1) [50]	+
17	5	nd (5) [50]	na
18	1	nd (1) [50]	na

+, A match between the serovar predicted based on KL type and that recorded in the literature.

*One genome lacking serovar information in the literature.

na, not applicable; nd, not determined.

We reviewed the Kaptive analysis results in detail and found that when a perfect or very high match was obtained, it represented an exact match or very close match to the KL reference sequence, e.g. the genomes of strains 405I and MIDG3457 (SRA run numbers ERR200067 and ERR200079) were typed as KL09 and KL11, respectively (Table S1).

Furthermore, we reviewed the gene hits in the Kaptive results (Table S1). The percentage of expected genes in the locus identified by Kaptive was at least 87 %. Missing genes in the best-matched locus reported by Kaptive usually resulted from missing pieces of assembly or low gene identity, which was frequently accompanied by homologous genes with higher identity identified as other genes outside the best-matched locus. It was noted that there was a 256 bp deletion in the *cps2A* gene in the *A *. *pleuropneumoniae* strain MIDG3426 (SRA run accession number ERR200042), indicating the difference in KL sequences between the *A *. *pleuropneumoniae* strain MIDG3426 and KL02 reference sequence. As we observed, all of the five low matched samples, including three KL06 isolates, a KL08 isolate and a KL12 isolate, lacked *lysA* in the KL, which might be due to low-quality sequencing or sequence assembly of the KL, especially the region around the *lysA* gene.

### Serovar prediction and confirmation

To examine the correspondence between KL type and serovar, we searched the serovar information of the WGS reads based on the SRA run accession numbers and strain names in the literature and compared them with the typing results from Kaptive. The predicted serovars of 97 tested genomes according to the assigned KL types, including KL01, KL02, KL05 to KL12, and KL15, were consistent with the serovar information documented in the literature (Table 3). The results showed an exact match (100%, 97/97) in the KL typing for *A *. *pleuropneumoniae* serovar prediction. Two genomes of strains MIDG2379 and Br384 (SRA run accession numbers ERR200009 and ERR200068) typed as KL06 and KL08, respectively, lacked serovar information in the literature. The genomes of *A *. *pleuropneumoniae* strains MIDG3419 to MIDG3422 and MIDG3440 (SRA run accession numbers ERR200035 to ERR200038 and ERR200050) and that of strain MIDG3435 (SRA run accession number ERR200049), of which serovars were previously undetermined, were typed as KL17 and KL18, respectively.

### KL typing of the *apxIV*-positive “*A. porcitonsillarum”* strain MIDG3255

The WGS reads of the *apxIV*-positive “*A. porcitonsillarum*” strain MIDG3255 (SRA run accession number ERR200087) were *de novo* assembled and subjected to KL typing. The strain was classified as KL03 with a very high match. Moreover, all 14 genes in the KL were identified. *cps3F* shared 99.8 % identity with the KL03 reference, and the remaining genes showed 100 % identity with the reference. The length of the KL of the “*A. porcitonsillarum*” strain MIDG3255 was the same as that of the KL reference sequence of KL03. The detailed results reported by Kaptive are provided in Table S2. Furthermore, the G+C content of the genome assembly of “*A. porcitonsillarum*” strain MIDG3255 was 41.1%, which is more similar to that of *A *. *pleuropneumoniae* serovar 3 strain JL03 (41.2 %; GenBank accession number CP000687.1) than that of “*A. porcitonsillarum*” strain 9953 L55 (39.7 %; GenBank accession number CP029206.1). Collectively, the results show that the “*A. porcitonsillarum*” strain MIDG3255 is a misclassification of *A *. *pleuropneumoniae* serovar 3.

## Discussion

In the present study, we established an *in silico* analysis strategy to predict *A *. *pleuropneumoniae* serovars. This strategy combines *apxIV* screening of WGS short reads using ariba against the *apxIV* dataset to distinguish *A *. *pleuropneumoniae* and non-* A *. *pleuropneumoniae* bacterial species, and KL typing of genome assembly using Kaptive against the *A *. *pleuropneumoniae* KL database. This study is, to the best of our knowledge, the first to demonstrate that a genome-based approach is able to predict *A *. *pleuropneumoniae* serovars and to provide detailed information on the KL for further investigation.

The KL typing of *A *. *pleuropneumoniae* was highly accurate in this study, as we observed that Kaptive classified the 105 tested *A *. *pleuropneumoniae* genome assemblies into 13 KL types, including KL types 01, 02, 05–12, 15, 17 and 18. KL types 04, 13, 14, 16 and 19 were not identified in our study due to the unavailability of public WGS short-read data for serovar 4, 13, 14, 16 and 19 isolates. Nevertheless, the high discriminatory power of *in silico* KL typing was shown by the distinction between KL9 and KL11.

Among the 105 *A *. *pleuropneumoniae* genomes used for Kaptive analysis, the serovar information for 97 genomes can be found in the literature. Based on KL types, the predicted serovars of the 97 genomes were exactly the same as those recorded in the literature, indicating that *in silico* KL typing may serve as an alternative *A *. *pleuropneumoniae* typing method. Previously nontypable or potentially mistyped isolates may be classified through our *in silico* typing method, as exemplified that six nontypable *A *. *pleuropneumoniae* isolates were predicted as serovars 17 and 18. Furthermore, the *apxIV*-positive “*A. porcitonsillarum*” strain MIDG3255 was predicted to be *A *. *pleuropneumoniae* serovar 3. Analysis of the KL information was sufficient for serovar prediction without referring to gene loci associated with lipopolysaccharide biosynthesis. Nevertheless, several reports have indicated that some *A *. *pleuropneumoniae* isolates were designated as K:O serovars, including K1:O7, K2:O7, K19:O3 and K19:O4 [4, 34–36]. For further detection of K:O-serovar isolates of *A *. *pleuropneumoniae *, an expanding typing pipeline can be developed with a database of biosynthesis gene loci for the outer core of lipopolysaccharides. Collectively, *in silico* KL typing can be used in retrospective studies or routine molecular typing of *A *. *pleuropneumoniae* isolates. As reported in this and previous studies, Kaptive can be applied to genotyping or genomic characterization of *Acinetobacter baumannii *, *A *. *pleuropneumoniae *, *Klebsiella michiganensis *, *K. pneumoniae* and *K. variicola* [16–18, 37].

As Kaptive analysis provides detailed information on the KL, we recommend a manual reviewing of the result, including coverage, identity, discrepancy in KL length, and a full list of identified genes with identities and missing genes, to identify variants or potential new KLs and to understand the difference between the KL in the query genome and the KL reference sequence. In our study, lack of identification of *cps* genes was rarely accompanied by a length discrepancy exceeding 100 bp between the full-length KL extracted from the query genome and the reference sequence of the best-matched locus. If an exception occurred, it was worthy of further investigation, e.g. the KL in *A *. *pleuropneumoniae* strain MIDG3426 with a 256 bp deletion in the *cps2A* gene compared with the KL02 reference. This suggests that the KL in the strain MIDG3426 may be a potential new KL or a locus variant of serovar 2; however, more biological evidence is needed to confirm the variation in the antigenicity of CPS.

Although *in silico* typing has advantages, including high accuracy and potential for obtaining variant information in routine molecular typing and retrospective analysis of *A *. *pleuropneumoniae* isolates, it might have some limitations. One limitation is the dependence on the quality of WGS reads. WGS reads with appropriate read length, coverage and accuracy are the basis for *de novo* assembly [38]. Based on the SRA metadata of the *A *. *pleuropneumoniae* WGS reads analysed in this study, we suggested a minimum depth of 120× and 60× for paired-end 75 and 100 bp sequencing, respectively, and a genome size of 2.2 Mbp for *in silico A. *pleuropneumoniae** typing. The contiguity and completeness of the genome assembly could also affect KL typing. A genome assembly with low contiguity might lead to discontinuous KL sequences in multiple contigs. Incompleteness of the genome might result from sequencing or assembly processes and lead to a poor match gene in Kaptive analysis. Low-quality genome assembly would lead to incorrect assignment of KL type; high-quality genome assembly would result in successful KL typing for serovar prediction.

During analysis, Kaptive evaluates any input genome, and the best-matched locus is given as the locus type for which the largest fraction of the locus has a blast hit against the assembly. In our unpublished data, by subjecting genome assemblies of non-* A *. *pleuropneumoniae* bacterial species to Kaptive analysis, KL types were assigned even though the identity was extremely low, leading to incorrect interpretation of the KL type. Therefore, we suggest that identification of the *A *. *pleuropneumoniae *-specific *apxIV* gene is essential prior to *in silico* KL typing.

The species-specific *apxIV* gene is clinically important for *A *. *pleuropneumoniae* identification in molecular typing [20]. In the present genomic approach, *A *. *pleuropneumoniae* WGS reads were accurately distinguished from those of the other 14 bacterial species of porcine, bovine and human pathogens by screening for the presence of the *apxIV* gene in WGS reads by ariba. ariba was originally used to identify antimicrobial-resistance genes from short-read data in *Enterococcus faecium *, *Shigella sonnei* and *Neisseria gonorrhoeae* [19], and was applied to find the *A *. *pleuropneumoniae *-specific *apxIV* gene in our study. In a previous study, detection of *apxIV* with PCR could be used to distinguish *A *. *pleuropneumoniae* from other bacterial species, including *Actinobacillus equuli *, *Actinobacillus rossii *, *Actinobacillus suis *, *Actinobacillus minor *, *Actinobacillus porcinus *, *Haemophilus* spp. and *Mannheimia haemolytica* [20]. Furthermore, *Actinobacillus lignieresii* is closely affiliated with *A *. *pleuropneumoniae* [39], and whether *A. lignieresii* can be distinguished from *A *. *pleuropneumoniae* by our approach remains unknown. Because of the lack of *A. lignieresii* WGS data in short-read format in the NCBI SRA database, we were unable to examine *A. lignieresii* for the presence of *apxIV* by ariba, as ariba works on paired-end short reads. Alternatively, we analysed the genome assemblies of three *A. lignieresii* isolates (GenBank accession numbers GCA_900444945.1, GCA_900444935.1 and GCA_900635785.1) by blast. Compared to *apxIV* of *A *. *pleuropneumoniae *, the *A. lignieresii* genomes showed an identity of 70–72 %, which was below the threshold (90%) required to identify the *apxIV* gene by the ariba tool. Therefore, we speculate that *A. lignieresii* would be distinguished as a non-* A *. *pleuropneumoniae* bacterium using our approach. Taken together, results showed that *A *. *pleuropneumoniae* could be distinguished from a total of 19 bacterial species by detection of *apxIV* in this and previous studies [20], demonstrating the consistent detection of *apxIV* as an *A *. *pleuropneumoniae *-specific marker. Nevertheless, identification of bacterial species could be achieved by analysis of the 16S ribosomal DNA (rDNA) gene [40, 41]. da Costa *et al.* reported that *A *. *pleuropneumoniae* was distinguished from *A. minor*, *A. porcinus* and *Pasteurella* spp. by 16S rDNA sequencing [42], indicating a potential use for 16S rDNA in the identification of *A *. *pleuropneumoniae* and phylogenetic analysis among closely related *Actinobacillus* species.

Collectively, the results of the present study show that *A *. *pleuropneumoniae* serovars can be predicted with a genomic approach through analyses of *apxIV* and KLs. *In silico* KL typing with Kaptive provides detailed genetic information and has advantages in the identification of potential variants or new KLs. As more KL sequences of new and regional prevalent serovars of *A *. *pleuropneumoniae* are obtained, the *A *. *pleuropneumoniae* KL database can be updated and expanded easily to ensure accurate assignment of KL types for *A *. *pleuropneumoniae* serovar prediction. This approach for *in silico* typing can be used for routine diagnosis, genomic surveillance and retrospective analysis of *A *. *pleuropneumoniae *.

## Supplementary Data

Supplementary material 1

Supplementary material 2
