# Supplementary material for: In silico capsule locus typing for serovar prediction of Actinobacillus pleuropneumoniae
Source: Microb Genom. 2022 Apr 11;8(4):000780. doi: 10.1099/mgen.0.000780 (PMC9453067; doi:10.1099/mgen.0.000780)
Supplement: Supplementary material 1 [file mgen-8-0780-s001.pdf]

Supplementary information

In silico capsule locus typing for serovar prediction of *Actinobacillus pleuropneumoniae*

Siou-Cen Li[1,2], Jing-Fang Huang[2], Yu-Ting Hung[2], Hsiu-Hui Wu[2], Jyh-Perng Wang[2], Jiunn-Horng Lin[2], Zeng-Weng Chen[2,*], and Shih-Ling Hsuan[1,*]

[1]Graduate Institute of Veterinary Pathobiology, College of Veterinary Medicine, National Chung Hsing University, Taichung, Taiwan, R.O.C.
[2]Animal Technology Research Center, Agricultural Technology Research Institute, Miaoli, Taiwan, R.O.C.

*Corresponding authors

Supplementary information includes two Supplementary data and two Supplementary tables.

This file includes two Supplementary data.
Two Supplementary tables are provided in an Excel file.

Supplementary data S1. The *apxIV* dataset. (a) The reference sequence in FASTA format. (b) The associated metadata in tab-separated values format.

(a)

```
>apxIVA.2.0.AF021919.1.1132-1641.0
ATGACAAAATTAACTATGCAAGATGTGACCAATTTATATTTATATAAAACGAAAACTCTACCTAAAGATA
GATTGGATGATTCACTTATTTCTGAAATAGGAAAAGGAGATGATGATATTGATAGAAAAGAATTTATGGT
GGGGCCGGGACGTTTTGTGACCGCTGATAACTTTAGCGTTGTAAGAGATTTTTTTAATGCTGGGAAATCA
CGCATTATTGCGCCGCAAGTCCCGCCTATTCGTTCACAGCAGGAAAAAATCTTGGTCGGTTTAAAACCGG
GCAAATATTCCAAAGCGCAGATATTGGAAATGCTGGGTTATACGAAAGGCGGAGAAGTGGTAAATGGCAT
GTTTGCCGGTGAAGTCCAGACATTAGGCTTTTATGACGATGGCAAAGGGGATTTACTCGAACGCGCCTAT
ATCTGGAATACCACAGGATTTAAAATGAGCGACAATGCCTTTTTTGTTATAGAAGAATCAGGCAAACGCT
ATATTGAAAACTTTGGTATTGAACCTCTTGGTAAGCAAGAAGATTTTGATTTTGTCGGCGGCTTTTGGTC
TAACTTAGTGAATCGTGGTTTGGAAAGTATTATCGACCCATCCGGTATCGGTGGAACGGTAAACCTTAAC
TTTACCGGCGAGGTGGAAACCTACACGTTAGACGAAACAAGGTTTAAAGCGGAAGCGGCGAAGAAAAGCC
ATTGGAGTTTAGTGAATGCGGCGAAAGTATACGGCGGTTTAGACCAAATTATTAAAAAACTATGGGACAG
TGGCTCAATTAAGCATTTATATCAAGATAAAGATACGGGCAAATTAAAACCGATTATTTACGGCACGGCC
GGCAACGACAGTAAGATTGAAGGCACTAAAATCACCCGTAGGATTGCGGGTAAAGAAGTTACGCTTGATA
TTGCCAATCAGAAAATTGAAAAAGGCGTGTTAGAGAAATTGGGGCTGTCTGTTAGTGGTTCGGATATCAT
TAAATTGTTGTTTGGAGCATTGACTCCAACTTTAAATAGAATGTTGCTATCACAACTTATCCAGTCTTTT
TCCGATAGCTTGGCTAAACTTGATAATCCCTTAGCCCCTTACACTAAAAATGGCGTGGTTTATGTCACCG
GCAAAGGGAATGATGTGCTTAAAGGAACTGAACATGAGGATTTGTTTCTCGGTGGTGAGGGGAATGATAC
TTATTATGCGAGAGTAGGCGATACAATTGAAGACGCCGACGGCAAAGGTAAAGTCTATTTTGTGAGAGAA
AAAGGGGTACCTAAGGCGGATCCTAAGCGGGTAGAGTTTAGCGAGTACATAACGAAAGAAGAAATAAAAG
AGGTTGAAAAGGGGTTATTAACTTACGCAGTTTTAGAAAATTATAATTGGGAAGAGAAAACGGCGACTTT
CGCTCATGCGACTATGCTTAATGAGCTTTTTACTGATTATACTAATTATCGTTATGAAGTTAAAGGACTA
AAATTGCCCGCCGTTAAAAAGTTAAAAAGTCCGTTGGTGGAGTTTACAGCTGATTTATTAACTGTTACGC
CTATTGACGAAAACGGAAAAGCACTTAGCGAAAAAAGTATTACGGTTAAAAATTTTAAAAATGGTGATTT
AGGAATAAGGTTGTTGGATCCTAATAGCTATTATTATTTCCTTGAAGGCCAAGATACGGGTTTTTATGGT
CCTGCTTTTTATATTGAACGAAAAAACGGTGGCGGCGCTAAAAATAACTCGTCGGGAGCAGGAAATAGCA
AAGATTGGGGCGGGAACGGGCATGGAAATCACCGAAATAATGCCTCCGACCTGAATAAACCGGACGGAAA
TAATGGGAATAACCAAAATAACGGAAGCAATCAAGATAATCATAGCGATGTGAATGCGCCAAATAACCCG
GGACGTAACTATGATATTTACGATCCTTTAGCTTTAGATTTAGATGGAGATGGGCTTGAAACCGTGTCGA
TGAACGGGCGACAAGGCGCGTTATTCGATCATGAAGGAAAAGGTATTCGTACCGCAACGGGCTGGCTCGC
TGCGGATGACGGTTTTTTAGTGTTAGATCGTAACCAAGACGGCATTATTAATGATATAAGCGAGTTATTT
AGTAATAAAAATCAACTTTCCGACGGCAGTATTTCTGCACACGGTTTTGCGACATTAGCCGATTTGGATA
CAAACCAAGATCAGCGTATCGACCAAAATGATAAGCTGTTTTCTAAACTCCAAATTTGGCGGGATTTAAA
TCAAAACGGTTTTAGTGAAGCGAATGAGCTGTTTAGCTTAGAAAGTTTGAATATTAAATCTTTACATACC
GCCTATGAAGAGCGTAATGATTTTCTAGCGGGCAATAATATCCTTGCTCAGCTTGGGAAGTATGAAAAAA
```

CGGACGGTACTTTTGCACAAATGGGCGATTTAAATTTCAGTTTTAACCCGTTTTATAGCCGATTTACCGA
AGCGTTAAATTTAACCGAGCAACAACGTCGCACAATTAATCTAACCGGCACCGGTCGGGTTCGGGATTTG
CGTGAAGCCGCCGCACTTTCTGAGGAGTTGGCTGCTTTATTACAACAGTACACTAAGGCCTCCGATTTTC
AGGCACAACGAGAATTATTGCCTGCCATTTTAGATAAATGGGCGGCAACGGATTTACAGTATCAACATTA
TGATAAAACATTACTTAAAACGGTAGAAAGTACCGATAGTAGTGCTTCTGTCGTTAGAGTCACGCCTTCT
CAATTAAGTAGTATACGCAATGCAAAGCATGATCCTACCGTTATGCAAAACTTTGAACAGAGTAAGGCAA
AAATTGCGACTTTAAATTCGCTCTACGGGTTAAATATCGATCAACTTTATTACACGACGGATAAAGACAT
TCGCTATATTACTGATAAAGTGAATAATATGTATCAAACAACCGTAGAACTTGCCTACCGTTCTTTACTT
TTACAAACGCGTTTGAAGAAATATGTTTATAGCGTTAATGCGAAACAATTCGAAGGGAAATGGGTAACCG
ATTATTCTCGTACTGAAGCCTTATTTAACTCTACTTTTAAACAATCGCCTGAAAATGCATTATATGATTT
AAGCGAATACCTTTCTTTCTTTAACGATCCTACGGAATGGAAAGAAGGGCTATTACTGTTAAGCCGTTAT
ATAGATTATGCTAAAGCACAAGGATTTTATGAAAACTGGGCGGCTACTTCTAACTTAACTATTGCCCGTT
TAAGAGAGGCTGGAGTAATTTTTGCAGAATCGACGGATTTAAAAGGCGATGAAAAAAATAATATTTTGTT
AGGTAGCCAAAAAGATAATAACTTATCGGGTAGTGCAGGTGATGATCTACTTATCGGCGGAGAGGGTAAT
GATACGTTAAAAGGCAGCTACGGTGCAGACACCTATATCTTTAGCAAAGGACACGGACAGGATATCGTTT
ATGAAGATACCAATAATGATAACCGCGCAAGAGATATCGACACCTTAAAATTTACCGATGTGAATTATGC
GGAAGTGAAGTTTCGACGAGTAGATAATGACTTAATGTTATTCGGTTATCATGATACGGATTCGGTCACG
GTAAAATCCTTCTACAGCCATGTAGATTATCAATTTGACAAATTGGAGTTTGCTGACCGCAGTATAACTC
GCGATGAACTGATTAAAGCAGGGCTTCATCTATACGGCACCGATGGCAATGATGATATAAAGGATCATGC
GGATTGGGACAGCATTTTGGAAGGCGGCAAAGGCAACGATATTCTAAGAGGTGGCTACGGTGCGGACACC
TATATCTTTAGCAAAGGACACGGACAGGATATCGTTTATGAAGATACCAATAATGATAACCGCGCAAGAG
ATATCGACACCTTAAAATTTACTGATGTGAATTATGCGGAAGTGAAATTCCGACGAGTAGATAATGACTT
AATGTTATTCGGTTATCATGATACGGATTCGGTCACGATAAAATCCTTCTACAACCATGTAGATTATCAA
TTTGACAAATTGGAATTTGCTGACCGCAGTATAACTCGTGATGAACTAGGTAAACAAGGTATGGCATTAT
TTGGCACTGACGGTGATGATAATATCAACGACTGGGGACGTAACTCGGTGATTGATGCCGGTGCGGGTAA
TGATACGGTTAATGGCGGTAATGGCGATGACACCCTCATCGGCGGCAAAGGTAATGATATTCTAAGAGGT
GGCTACGGTGCGGACACCTATATCTTTAGCAAAGGACACGGACAGGATATCGTTTATGAAGATACCAATA
ATGATAACCGCGCAAGAGATATCGACACCTTAAAATTTACCGATGTGAATTATGCGGAAGTGAAATTCCG
ACGAGTAGATAATGACTTAATGTTATTCGGTTATCATGATACGGATTCGGTCACGGTAAAATCCTTCTAC
AGCCATGTAGATTATCAATTTGACAAATTGGAGTTTGCTGACCGCAGTATAACTCGCGATGAACTGATTA
AAGCAGGGCTTCATCTATACGGCACCGATGGCAATGATGATATAAAGGATCATGCGGATTGGGACAGCAT
TTTGGAAGGCGGCAAAGGCAACGATATTCTAAGAGGTGGCTACGGTGCGGACACCTATATCTTTAGCAAA
GGACACGGACAGGATATCGTTTATGAAGATACCAATAATGATAACCGAGCAAGAGATATCGACACCTTAA
AATTTACTGATGTGAATTATGCGGAAGTGAAATTCCGACGAGTAGATAATGACTTAATGTTATTCGGTTA
TCATGATACGGATTCGGTCACGATAAAATCCTTCTACAACCATGTAGATTATCAATTTGACAAATTGGAA
TTTGCTGACCGCAGTATAACTCGTGATGAACTAGGTAAACAAGGTATGGCATTATTTGGCACTGACGGTG
ATGATAATATCAACGACTGGGGACGTAACTCGGTGATTGATGCCGGTGCGGGTAATGATACGGTTAATGG
CGGTAATGGCGATGACACCCTCATCGGCGGCAAAGGTAATGATATTCTAAGAGGTGGCTACGGTGCGGAC
ACCTATATCTTTAGCAAAGGACACGGACAGGATATCGTTTATGAAGATACCAATAATGATAACCGCGCAA

GAGATATCGACACCTTAAAATTTACTGATATTAATTTATCCGAACTTTGGTTTAGCCGAGAAAATAACGA

TTTGATTATTAAATCATTATTAAGTGAGGATAAAGTCACGGTTCAAAATTGGTATTCACACCAAGATCAT

AAAATAGAAAATATTCGTTTATCGAATGAGCAAACGTTGGTGAGCACTCAGGTGGAGAAGATGGTTGAGT

CGATGGCCGGCTTTGCTCAGAAGCACGGAGGAGAGATATCTCTTGTGTCGCTTGAAGAGGTAAAACAATA

TATCAATAGCTTAACAGCTGCTTTATAA

(b)

apxIVA.2.0.AF021919.1.1132-1641.0	1	0	.	.	ApxIVA

Supplementary data S2. The App KL database in GenBank format.

LOCUS **CP029003.1** 15240 bp DNA linear BCT 20-APR-2021

DEFINITION Actinobacillus pleuropneumoniae serovar 1 str. 4074

capsular polysaccharide gene locus, complete sequence.

ACCESSION CP029003 REGION: complement(1845704..1860943)

VERSION CP029003.1

KEYWORDS .

SOURCE Actinobacillus pleuropneumoniae serovar 1 str. 4074

ORGANISM Actinobacillus pleuropneumoniae serovar 1 str. 4074

Bacteria; Proteobacteria; Gammaproteobacteria; Pasteurellales;

Pasteurellaceae; Actinobacillus.

REFERENCE 1 (bases 1 to 15240)

AUTHORS Xu,Z., Chen,X., Li,L., Li,T., Wang,S., Chen,H. and Zhou,R.

TITLE Comparative genomic characterization of Actinobacillus

pleuropneumoniae

JOURNAL J. Bacteriol. 192 (21), 5625-5636 (2010)

PUBMED 20802045

REFERENCE 2 (bases 1 to 15240)

AUTHORS Xu,Z., Zhou,R. and Chen,H.

TITLE Direct Submission

JOURNAL Submitted (04-MAY-2010) College of Veterinary Medicine, Huazhong

Agricultural University, Shizishan Street 1, Wuhan 430070, China

REFERENCE 3 (bases 1 to 15240)

AUTHORS Li,L.

TITLE Direct Submission

JOURNAL Submitted (22-APR-2018) College of Veterinary Medicine, Huazhong

Agricultural University, Shizishan Street No.1, Wuhan, Hubei

430070, China

FEATURES Location/Qualifiers

source 1..15240

/organism="Actinobacillus pleuropneumoniae serovar 1 str.

4074"

/mol_type="genomic DNA"

/strain="4074"

/serovar="1"

/note="K locus: KL01"

/type_material="type strain of Actinobacillus

pleuropneumoniae"

/db_xref="taxon:228399"

CDS 1..1458

/gene="modF"

/locus_tag="APPSER1_08725"

/inference="COORDINATES: similar to AA

sequence:RefSeq:WP_012263300.1"

/note="Derived by automated computational analysis using

gene prediction method: Protein Homology."

/codon_start=1

/transl_table=11

/product="molybdate ABC transporter ATP-binding protein

ModF"

/protein_id="AWG96012.1"

/translation="MPNINIQNALFSLAQHNKLSIESLEINTHDFWVIVGGNGSGKTA

FAQALHNSLSLYSGEYQNSFQHIALLSFEQQQKIIEQIFKHRNNDMVSPDDFGLTARQ

IILNGSERTQLCEEYAAKLRIQPLLDRPFIQLSTGESRKVLFCQMLVSEPDLLILDEP

FEGLDQASVTYWQEVMAQLGKQMAVVLISNRFNDIPDCATHIALLDNLQLILQGERQE

IEQQAVYSQLKFAEQNVNAPLPESATPLIQLPPNTNPFELKNVMIRYGEKTIIDDLTW

TVAPKQHWWIKGPNGAGKSTLLSIIAGDHPQSYANYVHLFGRQRGSGETIWDIKKNIG

YVSSQLHMDYRVNCSALDVILSGFFDSIGVYQQVPSALQLKAMEWLERLHLANLAKKP

FRSLSWGQQRLLLITRAMVKHPPILILDEPLQGLDGVNRKLVKQFIEQLVTNSQTQLL

FVSHQDADAPNCITHLFEFVPQTNGGYRYVQTALN"

CDS complement(1517..2167)

/gene="cpxA"

/locus_tag="APPSER1_08720"

/inference="COORDINATES: similar to AA

sequence:RefSeq:WP_005618062.1"

/note="Derived by automated computational analysis using

gene prediction method: Protein Homology."

/codon_start=1

/transl_table=11

/product="ABC transporter ATP-binding protein"

/protein_id="AWG96011.1"

/translation="MISVKNVSKDYYTRSGKKTVLQDINFELKKGEKIGILGRNGAGK

STLIRLLSGVEPPTSGTIERNMSISWPLAFSGAFQGSLTGMDNLRFICRIYNADIEYV

KAFTEEFSELGDYLYEPVKKYSSGMKARLAFALSLSVEFDCYLIDEVIAVGDSRFAAK

CKHELFEKRKDRSIILVSHSPSAMKSYCDNAMVLDKGIMYKFENMDEAYKFYNSTL"

CDS complement(2164..2961)

/gene="cpxB"

/locus_tag="APPSER1_08715"

/inference="COORDINATES: similar to AA

sequence:RefSeq:WP_005598930.1"

/note="Derived by automated computational analysis using

gene prediction method: Protein Homology."

/codon_start=1

/transl_table=11

/product="ABC transporter permease"

/protein_id="AWG96010.1"

/translation="MQYGDQTTFRQSLAIQGRVIGALLMREIITRYGRKNLGFLWLFV

EPLLLTLFIVLMWKFIRADRVSDLNIIAFVITGYPMAMMWRNASNRTIGAISGNLSLL

YHRNVRVLDTLLARVILEVAGATIAQIIIMALVILLGWIEMPKDTFYMVMAWVLMAFF

ALGLGLIICSIAQKFEAFGKIWGTLSFVLLPLSGAFFFVHALPSQAQQYATLIPMIHG

TEMFRHGYFGDSVITYESISYLVICDVAMLLFGLIMVKNFSKGIEPQ"

CDS complement(2961..4118)

/gene="cpxC"

/locus_tag="APPSER1_08710"

/inference="COORDINATES: similar to AA

sequence:RefSeq:WP_005608964.1"

/note="Derived by automated computational analysis using

gene prediction method: Protein Homology."

/codon_start=1

/transl_table=11

/product="capsule biosynthesis protein"

/protein_id="AWG96009.1"

/translation="METPIATSPAEKLQKPIKQKKSWLKKLNPLFWVTVAIPTVLSAF

YFGSVASDIYISESSFVVRSPKNQTALTGVGALLQGSGFSRAQDDTYTVQEYMHSRTA

LEQLMKDLPIREYYENQGDIIARFNGFGLNNSKEAFYKYFRDRLSVDFDSVSGIASLR

IRAFNAEEGQQINQKLLAEGETLINRLNERARKDTISFAEQAVKEAENNVNATASDLS

KYRIKNKIFDLPAQSGVQLSLISSLKSELIRVETQLAQLQSITPDNPQVDALLMRQKS

LRKEIDEQSKQLSSNSNSSIAIQTADYQRLVLANELAQQQLTAALTSLQNTKNEADRQ

QLYLEVISQPSKPDWAEEPYRLYNILATFFIGLMLYGVLSLLIASVREHKN"

CDS complement(4144..5328)

/gene="cpxD"

/locus_tag="APPSER1_08705"

/inference="COORDINATES: similar to AA

sequence:RefSeq:WP_011848622.1"

/note="Derived by automated computational analysis using

gene prediction method: Protein Homology."

/codon_start=1

/transl_table=11

/product="polysaccharide export protein"

/protein_id="AWG96008.1"

/translation="MKLIKLRLLLSLGLVASLAACSSLPTSGPSHSAILEANSQSSDK

PLPEVNVVELDNGLVQQLYQTQQSQQFSGFLGTVGSAGYAGAVNVGDVLEISIWEAPP

AVLFGGTFSSEGQGSGHLTQLPAQMVNQNGTVTVPFVGNIRVAGKTPEAIQSQIIGAL

QRKANHPQALVKIANNNSADVTVIRQGNSIRMPLTANNERVLDAVAAVGGTTENIEDV

TVKLTRGSEVKTLAFETLISDPAQNIMLRAGDVVSLLNTPYSFTGLGAVGNNQQMKFS

SKGITLAEAIGKMGGLIDTRSDPRGVFVFRHVPFAQLSLEQQAQWQAKGYAIGMDVPT

VYRVNLLEPQSMFLLQRFPMQDKDIVYVSNAPLSEFQKFLRMIFSITSPVTSTTNAVR

AY"

CDS 5562..6674

/gene="cps1A"

/locus_tag="APPSER1_08700"

/inference="COORDINATES: similar to AA

sequence:RefSeq:WP_005605524.1"

/note="Derived by automated computational analysis using

gene prediction method: Protein Homology."

/codon_start=1

/transl_table=11

/product="capsule biosynthesis protein CapC"

/protein_id="AWG96007.1"

/translation="MNKMNRKFSKLLKNPHIFFRDFLNKKYPIKNTELPFSESEEANL

IEANQKLDKIIQKNTLQQTNIDVVFTWVDGSDPSWQAKYSQYAPNYQAKSALYATDIA

RFEDHNELYYSVHAVLKYMPWVRHIFIITDNQKPKWLDETRQEKITLIDHQDIIDKEY

LPTFNSHVIEAFLHKIPNLSENFIYFNDDVFIARELQAEHFFQANGIASIFMSEKSLT

QMRNRGTITPTLSASEYSIRLLNKYYNTNIDSPLVHTYIPLKKSMYELAWRRYEKEIL

GFLPNKLRTNNDLNFANFLIPWLMYFEGKAMPKIDICYYFNIRSPNALTQYKKLLNKK

NIGEQPNSFCANDFNSQKSINNYQNQLFSFLNSYYS"

CDS 6685..10428

/gene="cps1B"

/locus_tag="APPSER1_08695"

/inference="COORDINATES: protein

motif:HMM:PF04464.12,HMM:PF13641.4"

/note="Derived by automated computational analysis using

gene prediction method: Protein Homology."

/codon_start=1

/transl_table=11

/product="glycosyl transferase"

/protein_id="AWG96006.1"

/translation="MNKVKRKFRKLLRDPKLFFSDMYFKHSIKIKKHLPVKYEGKHQF

TIVSAVYNVEKYLDDFFDSIVKQNLSFKKHIQIILVDDGSKDSSANIIKKWQKKYPNN

IHYYYKENGGQASARNLGLKYVQTEWVTFIDPDDFLSLNYFLEVDKKLSEHKNIAMIV

CNLLFFMEKKEIITDKHPLKFRFEKDVNCLSIKDLNNNLNLSVATSFFRTSVIQGNQL

LFDNRVKPNFEDGKFISDYLFELQHYNALFLKKPVYFYRKREDGTSTLDTSWQKPEKY

KNVLEYGFIPMLQKYHNKLSYVPNNIQKTALYDMYWYIQYLLNRPEKIRFLSEKDQAK

FYQLYDKVFEYIDVENIMQFNIAGAWFFHKVGMIGAFKNQRPPFQIAYIENIDREKKQ

ILISYFTYFDDCNSFRLNGRDTLPVYQKTVTNTFNEKLFTYEKRSWIPFEKEDDILTI

SLNGLMMRISVKGTLFSKGISINKILSAFTPQAKYLTDGSWLLMDRETKADDNAEHFY

RYMQTHHPEQRCYFVLNKSSIDWQRLKKDKFNLVEFGSIEYERRLEKASKIISSHLEA

HINNYFGDNYDFSKKFIFLQHGITKDDLSQWFNTKKNLSGVITATIPEYNSIVEELNK

YKIGKKETFLTGFPRHDKLLSGNIKGAKTILIVPTWRHYIMGTQIGKGANTRELNKAF

MTTNYAKAWYNLLHSQELKNLIKNLGYKVIFAPHPNIEPYLNEFNIPQYIDVWKSAIS

RESMQSLFQQSNLLITDYSSIAFEMAFLGKQTIYYQFDKEEFRSGIHTYQQGYFEYEK

DGFGPVAETLDDLFIHLDKFVNGENDYINIYQSRIQKTFKYRDTNNCQRVYEAIINLD

MPDKDINKNIILNALESAYKAQDWNLVISRAEALLAEIPNHSFAKSVLFEAVIASNNK

EKMQVLLASPLLNQQEKAVLQATICSQKLAWQDVLNHLKGIILSNEQLLVLSLKANAY

LHNAKGTQKTANKLSKIIDKNKKYLFKAWVAFANQDWISVISLLESKLSELSKKDLDL

YLPELLLARAYCQLNDFTSSHNCLVAFERHSAAFPLSRIEIAHLAYARRNYTKCIDQL

NKCFAKELDNLPAESLEEYALSLLKGNNTKEFEKLVESSLSEKFKDRTFFKKEYVSFL

VKNQLWKKLVKYASNWALQDKEKFNYPLMLAHYRLGDIAYVYQNHIKPTEEHPYEYWE

LIAETALLYEDIDLSKYCYRGIISIFPNKNKQQNLSIFFEKFI"

CDS 10464..11684

/gene="cps1C"

/locus_tag="APPSER1_08690"

/inference="COORDINATES: similar to AA

sequence:RefSeq:WP_005820271.1"

/note="Derived by automated computational analysis using

gene prediction method: Protein Homology."

/codon_start=1

/transl_table=11

/product="hypothetical protein"

/protein_id="AWG96005.1"

/translation="MLKEQTYKASNGVSIIYKEKKNKFDFKHLIFVFSGFLNSTPGNY

DFGNALNDCPANVIWINDNFEGMYSYYLCVNMLFSVEDAVTEFIYHKVNELGLTFDNI

TVTGFSKGGSAALYYGLKLPVANIVATVPQTKIGSYVVKHWKHVAEHMMGKITPTRVS

HLDKLITQKLKQDKKLDRNIYLLTSESDIQFPTEIVTILNDLRKYSNFNLLKTFSCFA

REHNQIKSHHTALLLGIYYSLASEATPRFNNGEVNFFGIQPLPPKEITGEPFIDLRKA

EIKDNLLFVDGVAILRGYDLVEYSDVEYQLIFKSERNNIVKNLAKTHKPQLTRELFDG

ENLVIYDKGWFTTYQYKGIDISDIPPGKYSVYININLSDKSACLPLTTKLNKIMTNNK

CTLHVVNNIVYFEK"

CDS 11735..12778

/gene="cps1D"

/locus_tag="APPSER1_08685"

/inference="COORDINATES: protein motif:HMM:PF00132.22"

/note="Derived by automated computational analysis using

gene prediction method: Protein Homology."

/codon_start=1

/transl_table=11

/product="acyltransferase"

/protein_id="AWG96004.1"

/translation="MNIKEIYKKIKNELPVEYDIKIYQEQSIYWEKIINSPHSKDKYN

LAISLNIKDKIIYLYRRDKEELKDINEIIPNFRLSERNNVFFHNIENYSLNESCEIFY

ILVKNAEDYLKNILIQNTKQKNVISGDYYDDMDNKISAPIGLVNVHFQFLGSNNKILI

SPKANLKNTFIECRGNNNTIIIDENVRMVGHWRLGFGCTLKIGKNSSSTNPVYITVAE

NTQLTIGEDCMFATNNQIRTDDAHPIYDVNTGKRVNMSKDIQIGDHVWIGYGATILSG

SAIGSGSVIGAGSIVRNKFPNNCVIAGTPAKVVKKDIFWERPLLLNMSEEVVYSEEER

RQKNYCKNTMETE"

CDS complement(13015..13194)

/locus_tag="APPSER1_08680"

/inference="COORDINATES: ab initio prediction:GeneMarkS+"

/note="Derived by automated computational analysis using

gene prediction method: GeneMarkS+."

/codon_start=1

/transl_table=11

/product="hypothetical protein"

/protein_id="AWG96003.1"

/translation="MKKTFLSKALIAIAACSVFFSATAANAEAGKWRKTGFHFLGLYQ

YCYTAYPYDCMWLRP"

CDS 13836..13949

/locus_tag="APPSER1_08675"

/inference="COORDINATES: similar to AA

sequence:RefSeq:WP_005639040.1"

/note="Derived by automated computational analysis using

gene prediction method: Protein Homology."

/codon_start=1

/transl_table=11

/product="hypothetical protein"

/protein_id="AWG96002.1"

/translation="MKKLLLAVLIASFGLAACGVKGPLYFPEQQPAQQQTK"

CDS 14080..14475

/note="similar to the nucleotide

sequence:CP022715.1 complement(652054..652449)"

/pseudo

/codon_start=1

/product="diaminopimelate decarboxylase"

CDS 14406..14654

/locus_tag="APPSER1_08665"

/inference="COORDINATES: similar to AA

sequence:RefSeq:WP_005621122.1"

/note="incomplete; partial on complete genome; missing

start; Derived by automated computational analysis using

gene prediction method: Protein Homology."

/pseudo

/codon_start=1

/transl_table=11

/product="nucleoside-diphosphate sugar epimerase"

CDS complement(14698..15240)

/gene="ydeN"

/locus_tag="APPSER1_08660"

/inference="COORDINATES: similar to AA

sequence:RefSeq:WP_005621124.1"

/note="Derived by automated computational analysis using

gene prediction method: Protein Homology."

/codon_start=1

/transl_table=11

/product="serine hydrolase family protein"

/protein_id="AWG96001.1"

/translation="MKKVYVTHGYTANPTRNWFPWLKNELEKLGWECECLAMPNSDQP

NPQAWLEHHQNTLQLDENTLLIGHSLGCIALLNYLAVTQQKVKTAIFVSGFYEKLPTL

PELDSFADFYANQTACLPQKSYVISALNDVVVPHSFSDRLAQYLQADYIRLATGGHFI

DREGVTELPVLLELLKQILK"

ORIGIN

1 atgccaaata tcaacatcca gaacgcctta ttttcccttg ctcaacacaa taaactctcg

61 attgaatcac tggaaatcaa tactcacgat ttctgggtga ttgtcggcgg taacggctcg

121 ggcaaaaccg ctttcgccca agcgctacat aattcacttt cgttatattc gggtgaatat

181 caaaatagtt tccagcatat cgctttactt tccttcgagc agcaacaaaa aatcatcgag

241 caaatcttta aacaccgtaa caacgatatg gtttcaccgg atgatttcgg tttaaccgcc

301 cgtcaaatta tcttaaacgg tagcgaaaga acgcaattat gcgaggaata tgcggctaaa

361 ttacgtatcc agccgttatt agatcgcccg tttattcagc tctccaccgg cgaaagccgc

421 aaagtgttat tttgccaaat gttagtcagc gaaccggatt tattgatttt agatgagcct

481 tttgaagggt tagaccaagc ctcggtcact tattggcagg aagtgatggc acaactcggt

541 aagcaaatgg cggtggtact gatttccaac cgttttaatg atattcccga ctgtgccaca

601 catattgctt tactggataa cttacaactg attttacaag gcgaacgtca agagattgaa

661 caacaagcgg tctattctca gctaaaattt gcagaacaga atgtgaatgc accgttgccg

721 gagagtgcca caccgctgat tcaactccca ccgaatacta atccgtttga actgaaaaac

781 gtaatgatcc gttacggcga aaaaacgatt attgatgatc taacttggac ggttgcccca

841 aaacaacatt ggtggattaa aggcccgaac ggagcaggaa aatcgacctt actttctatt

901 attgccggcg atcatccgca atcttacgct aattatgtgc atttattcgg tcgtcagcgt

961 ggttcgggcg aaacgatttg ggatataaag aaaaatatcg gctatgtgag cagccaatta

1021 catatggatt atcgggtgaa ttgctctgcg ttagacgtga ttttatccgg ctttttgat

1081 tcaatcggcg tttatcaaca agtaccgagt gccttacagc taaaagcaat ggaatggctg

1141 gaacgcttgc atttagccaa tctggcgaaa aaaccgttcc gttcactttc gtgggggcaa

1201 caacggttat tattgattac tcgtgctatg gtaaaacacc cgccgattct gattttagac

1261 gaaccgctgc aaggtttgga cggtgtaaac cgcaaattgg ttaaacaatt tatcgaacag

1321 cttgtgacta atagtcaaac ccagttgcta tttgtttcgc accaagatgc ggacgcccca

1381 aattgtatca cccatttatt tgaatttgtt ccgcaaacta acggtggtta ccgttatgtg

1441 cagacggcgt taaattaggt ttttgacctt taaaggaaat ccccctcttt agtaaagagg

1501 ggggatgtgt gggactttaa agcgttgaat tatagaactt ataagcctcg tccatattct

1561 caaatttata cataatccct ttatctaata ccattgcatt atcgcaataa gacttcattg

1621 ctgacggact atgcgaaacc aaaataatcg aacgatcttt gcgcttttca aataattcat

1681 gtttacattt tgccgcaaag cgagagtcac ctaccgcaat tacctcatca attaagtagc

1741 aatcaaactc taccgaaagc gacaaagcaa aggcaagtcg ggctttcatg ccggaggaat

1801 atttcttaac cggctcatat aaataatcac ccaattcgga aaattcttcg gtaaaggctt

1861 taacgtattc aatatccgca ttatatatac ggcaaataaa gcgtaaatta tccataccgg

1921 ttaaactgcc ttggaacgcc ccgctgaaag cgagcggcca agatatcgac atattacgtt

1981 cgatagtacc tgatgttggc ggctcaacac cacttaacaa acggattagc gttgatttcc

2041 ctgcaccgtt acgccctaaa ataccgattt tctcgccttt tttcagctca aaattaatat

2101 cttgcaatac ggttttttta ccgcttcgag tatagtaatc tttactcaca ttttttacgc

2161 taatcattgc ggttcgattc ctttactgaa gttttttacc ataatgagcc caaaaagtaa

2221 catggctaca tcacatatta cgagatagct tatactttca tatgtgataa cactgtcgcc

2281 aaaataaccg tgacgaaaca tttccgtgcc gtgaatcatc ggtattaagg ttgcatattg

2341 ttgagcttgg cttggtagcg catgcacaaa gaaaaatgcg cctgaaagag gtaaaagaac

2401 aaagcttaat gttccccaga ttttgccaaa tgcttcaaat ttttgtgcaa tagaacaaat

2461 aatcaagcct aatcctaatg caaaaaatgc cattaatacc cacgccataa ccatataaaa

2521 cgtatctttc ggcatttcta tccagcctaa taaaatgact aatgccataa taatgatttg

2581 ggcaatcgtt gcacccgcta cctcaagtat gacacgagcc agtaaggtat ctaatacgcg

2641 aacattacga tgataaagaa gactcaagtt accggaaatt gcaccgatag tgcggtttga

2701 tgcattacgc cacatcattg ccattggata accggtaatc acaaaagcaa taatatttaa

2761 atcggaaacg cgatccgctc ggataaattt ccacatcaaa acgataaata aagtgagtaa

2821 tagcggctca acaaacagcc ataaaaaacc caaatttttt cgtccgtaac gcgtaataat

2881 ttcccgcatg agtaatgcac cgattactct cccttgaatg gcgagagatt ggcggaaagt

2941 tgtttgatca ccgtattgca ttagtttttg tgctctctta cgcttgcaat taataaactt

3001 aatacaccat aaagcatcag accgataaag aatgtcgcta aaatattata taagcgataa

3061 ggctcttccg cccagtccgg tttgcttggc tgactgatta cttctaaata aagttgctgg

3121 cgatccgctt catttttcgt attttgtaat gaggttaatg ctgcggtcaa ttgttgctgt

3181 gccagctcgt ttgcaagtac taagcgttgg taatcggcag tttgaatagc aatagagcta

3241 ttactgttac tggaaagctg ttttgattgc tcatcgattt ccttacgtaa actttttgg

3301 cgcataagca atgcatcaac ttgcgggttg tccggtgtaa tagattgcaa ttgagccaat

3361 tgtgtttcta cacgaatcaa ttcgcttttt aagctggaaa ttaatgaaag ttgtacgccg

3421 gattgtgccg gtaaatcaaa gattttattt ttgatacggt atttacttaa gtcgcttgcc

3481 gttgcattta cattattttc cgcttcctta accgcttgtt ccgcaaatga aatggtatct

3541 tttcttgcac gttcgtttaa acggttgatg agtgtttcac cttcggcaag taatttttga

3601 ttaatttgtt gtccctcttc tgcattaaaa gcacgaatac gtaagctggc aataccggat

3661 acagaatcga aatcaacact taagcgatct cggaaatatt tgtaaaacgc ttctttacta

3721 ttatttaaac caaatccatt aaagcgagcg ataatatcgc cttgattctc atagtattca

3781 cgtattggta ggtctttcat taactgttct aatgccgtac gagaatgcat atattcttgt

3841 acggtataag tatcatcttg agcacgagaa aatccggaac cttgtaataa ggccccgaca

3901 ccggttaaag cggtctgatt tttaggagat cttacaacga agcttgattc cgaaatataa

3961 atatcggaag caacagaacc gaaataaaag gctgataata ccgtcggaat cgctacagtt

4021 acccaaaata acggattaag cttttttaac caactttttt tctgtttaat cggtttctgt

4081 agtttttctg ctggactggt agcaataggt gtttccatct tttgtcctta tacattcaat

4141 atattaatag gcacgaacgg cattggtcgt actggtaacc ggcgaagtaa tcgagaaaat

4201 cattctcaag aatttttgga attcagacaa cggcgcattt gaaacataca caatatcttt

4261 atcttgcatt gggaaacgct gtaataaaaa catggattgc ggctcaagta agttcacacg

4321 ataaaccgtt ggtacatcca ttcctatagc gtagcctttta gcttgccatt gtgcttgttg

4381 ttccaaactc aattgtgcaa aaggcacgtg acggaatacg aaaaccccctc tcggatccga

4441 acgagtatca attaaaccgc ccatcttacc gatagcttcg gcaagcgtaa ttcctttact

4501 tgagaatttc atttgctggt tgttacccac cgcacctaaa ccggtaaaac tataaggtgt

4561 gtttagcagt gaaacaacat cgccagcacg taacataata ttttgtgccg gatcggaaat

4621 taacgtttcg aatgcgagtg tttttacttc agaaccacgg gttagcttga ccgtcacatc

4681 ttcaatgttt tccgttgttc cgcctactgc agcaaccgca tctaatacac gttcattatt

4741 agcggttaat ggcatacgaa tactattgcc ttgacgaata accgtaacat cagcagagtt

4801 attattcgca attttgacta atgcttgcgg atgattcgct ttgcgctgta gtgctccaat

4861 aatttgagac tgaatcgctt ccggtgtttt gcctgcgaca cgaatgttac ccacgaacgg

4921 cacggtaacc gtaccgtttt gattaaccat ttgtgccggt aattgcgtta aatgcccgct

4981 accttgtccc tcagaactaa aagtaccgcc aaacagcacc gccggcggag cttcccaaat

5041 tgatatttca agtacatcac ccacattgac cgcaccggca tagcccgcgc tgcctactgt

5101 gcctaaaaat ccggaaaatt gttggctttg ctgagtttga tacaactgtt gaactaaacc

5161 gttatccagt tccaccacat ttacttccgg taagggttta tccgaacttt gtgaattagc

5221 ctctaagatc gcactatggc tagggcctga agtgggtaag cttgagcagg cagccaaact

5281 agcaaccagc cccaaagaaa ggagtaatct aagtttgatg agtttcatct aatttctctt

5341 caatatatta aggaataaca actatatagg tatgtcttaa aatccacata aagattgatt

5401 ttaataagtt acctaatcaa gagaaattaa atataaaaaa tttacaaaaa agcaataatg

5461 cgtataaaaa aacatcattt gtaaagaaag taaatagaga ggagggttca acagataagc

5521 attataatcc aagatttaca taaaatataa ttgataatat aatgaacaaa atgaatagaa

5581 aattttctaa gttactaaaa aatccacata tttttttag ggattttcta aataaaaagt

5641 accctataaa aaatacggaa cttcccttct cagaatctga agaagctaac ttaatagaag

5701 caaaccaaaa attagataag attatccaaa agaatacgtt gcaacaaact aatattgatg

5761 tggtatttac ttgggtagat ggttctgatc cttcatggca agctaaatat tcccaatatg

5821 caccaaatta tcaagcgaaa tccgctctat atgcaacgga tatcgcccga tttgaagatc

5881 ataatgaatt atattattca gtacatgctg tacttaaata tatgccttgg gttaggcata

5941 tatttattat aacagataat caaaagccaa agtggctgga tgagacgaga caagaaaaaa

6001 ttacactaat cgatcatcaa gatattatag ataaagaata tcttccaacg tttaattccc

6061 atgttattga agcattttta cataaaattc ctaatttaag cgagaatttt atctatttta

6121 atgatgatgt ttttattgca cgagaactac aagctgaaca ctttttccaa gcaaatggta

6181 ttgcctctat atttatgtcg gaaaaaagcc tcactcaaat gcgtaacaga ggaactatta

6241 caccgactct ttctgcttcg gaatatagta ttcgcttact aaacaaatat tacaatacaa

6301 atattgactc accacttgta cacacttata tcccattgaa aaaaagtatg tatgaattgg

6361 catggcggcg ttatgagaaa gaaattcttg gatttttacc caataaatta agaacaaata

6421 acgatttaaa ttttgcaaac tttcttattc cttggttaat gtatttcgaa gggaaagcaa

6481 tgcctaaaat agatatttgt tattatttta atattagatc tccaaatgca cttacacaat

6541 ataaaaaact tttaaataaa aaaaacatag gcgaacagcc taattcattt tgcgcaaatg

6601 attttaatag tcaaaaaagt attaacaact atcaaaatca attgttttct tttttaaact

6661 cctattacag ttaaggataa tataatgaat aaagtaaaac gtaaatttag aaaattactg

6721 cgtgatccta agttgttttt tagtgatatg tatttcaaac attctataaa aataaaaaaa

6781 catttacctg ttaaatatga aggaaaacat caatttacga ttgtttccgc tgtatataat

6841 gtagaaaaat atcttgatga tttctttgat agtatcgtta aacaaaattt atcatttaaa

6901 aaacacatac agattatctt agttgatgac ggctcaaaag attcatcagc aaacatcatc

6961 aaaaaatggc aaaaaaaata tccaaataat atccactatt attataaaga aaatggtggg

7021 caagcctctg ctcgtaattt aggactaaaa tacgtacaaa cagaatgggt taccttttatt

7081 gatccagatg attttcttag cctaaattat ttcttagaag tagataaaaa gttatcagaa

7141 cataaaaata ttgcaatgat tgtatgtaat ctattatttt ttatggaaaa gaaagaaatt

7201 attactgata aacatccttt aaaatttaga tttgaaaaag atgttaattg tttatcaatt

7261 aaagatctta ataataattt aaacttatct gtagcaacaa gtttctttag aacctctgta

7321 atacaaggta atcaactatt atttgataat agagtaaaac caaattttga agatggtaaa

7381 tttatttctg attatttatt cgaattacaa cactataatg ctttattttt aaagaaacct

7441 gtctattttt atcgaaaacg tgaagatggt acttcaactt tagatacttc ttggcaaaag

7501 cctgagaaat ataaaaacgt actagagtat ggttttattc caatgttaca gaaataccat

7561 aacaaactat catatgttcc taataacatt caaaaaacgg ctctttatga tatgtattgg

7621 tatattcaat atctattaaa tagaccagaa aaaataagat tcctatctga aaaagatcaa

7681 gctaaatttt atcaactcta tgataaagta ttcgaatata ttgatgtaga aaatattatg

7741 caatttaata ttgcaggagc ttggttcttt cataaagtag gtatgattgg agcatttaaa

7801 aatcaaagac ctcctttca aattgcatat atagaaaata ttgaccgtga gaaaaagcaa

7861 attcttatta gttactttac ttattttgat gattgtaatt cctttaggtt aaatggtaga

7921 gatacattac ctgtttacca aaaaacagta acaaatactt ttaatgagaa attatttacc

7981 tatgaaaaaa gaagctggat tccttttgaa aaagaggatg atattttaac tatttcttta

8041 aatggtctaa tgatgagaat atctgttaaa ggtactcttt tcagtaaagg tatttctatt

8101 aataaaatct tatcagcatt tacaccgcaa gctaaatatt taaccgatgg cagttggctt

8161 ttaatggata gagaaacaaa agcagatgat aatgctgaac atttctaccg ttatatgcag

8221 actcatcatc ctgagcaaag atgttacttt gttttgaata agagctcaat tgactggcaa

8281 agattgaaaa aagataaatt taatttagtt gaatttggct ctattgaata tgaaagacga

8341 ttagaaaaag caagtaaaat tattagtagc catttagagg ctcatattaa taattatttt

8401 ggcgacaatt atgattttag taaaaaattt atatttttac agcatggtat aactaaagat

8461 gatttatctc aatggttcaa tactaaaaag aatttatctg gagtaattac ggcgactatt

8521 cctgaatata actcaatagt agaagaacta aataaatata aaattggtaa aaaggaaaca

8581 tttttaacag gatttcctcg ccatgataaa ttactatctg gaaatataaa aggagctaag

8641 acaattctca tcgtacctac atggcgacat tatattatgg ggactcaaat tggaaaagga

8701 gccaatacac gcgagctaaa taaagccttt atgacaacaa attatgctaa agcttggtat

8761 aatttattac atagtcagga attaaaaaat ttaattaaaa atttaggata taaagttatt

8821 tttgcaccac accctaatat tgaaccatat ttaaatgagt ttaacatccc ccaatatatt

8881 gatgtgtgga aaagtgcaat atcaagagaa agtatgcaaa gtttattcca acaatcaaat

8941 ctattgatta cggactattc atctattgca tttgaaatgg catttctagg aaaacaaaca

9001 atctattacc aatttgataa agaggaattt agatctggaa ttcatacata tcaacaagga

9061 tactttgaat acgagaaaga tggatttggt cctgtagctg aaacattaga tgatttattt

9121 attcacctag ataaattcgt aaacggtgaa aatgattaca taaatattta tcaatctcgt

9181 atacaaaaaa catttaaata tcgggatacg aataattgcc aacgtgttta tgaagctatt

9241 attaacttag atatgccgga taaagatatt aataaaaata ttatcttaaa tgctttagaa

9301 tctgcttaca aagctcaaga ctggaattta gttatctctc gtgctgaagc tttattagca

9361 gaaataccta atcattcatt tgctaagagt gtgttatttg aagcagtgat agcttcaaac

9421 aataaagaga aaatgcaagt actactagct tctcctttat taaatcaaca agaaaaagca

9481 gtcctacaag ccactatatg ttctcaaaaa ttagcatggc aagatgtgtt aaatcattta

9541 aaaggaatca tactttcaaa tgaacaatta ctagtattat cattaaaagc taatgcctat

9601 ttacataatg caaaaggtac tcaaaaaaca gcaaataagt tatcaaaaat tattgataaa

9661 aataaaaagt accttttcaa agcttgggtt gcttttgcaa accaagattg gattagcgta

9721 atctcattac ttgaaagtaa gctctctgag ttaagtaaaa aagacttaga tttatatttg

9781 cctgagctat tgcttgctcg agcttactgt caattaaatg actttacctc ctcacataat

9841 tgtttagttg catttgagag acactctgct gcatttccat tgtctcgaat cgaaatagca

9901 cacctagcat atgcaagacg aaattataca aaatgtattg atcaacttaa taaatgcttt

9961 gctaaagaac ttgacaatct acctgcagag agtttagagg aatatgctct ttccttactt

10021 aaaggaaata acactaagga atttgaaaaa ttagtagaaa gttctctaag tgagaagttt

10081 aaagatcgaa ctttctttaa aaaagaatat gtttcctttc ttgttaaaaa tcagttatgg

10141 aaaaaattag ttaaatatgc tagcaattgg gcattacaag ataaagaaaa attcaattat

10201 ccattaatgc ttgctcatta tagattagga gatattgcat atgtttatca gaatcatata

10261 aaaccgactg aagaacatcc ctatgaatat tgggaattaa ttgctgaaac agccctatta

10321 tatgaagata ttgatctatc taaatattgt tatagaggaa taatttctat cttccctaac

10381 aaaaataagc aacaaaatct ttctatcttt tttgaaaaat tcatctaaat atatagcccc

10441 tcaaggggct tttaaggaca tttatgctaa aagagcaaac atataaagca agtaatggtg

10501 tttctattat ttacaaggaa aagaaaaata aattcgattt taaacatcta atttttgttt

10561 tttcaggatt tttaaatagt acacctggaa attatgattt tggaaatgcc ttaaacgatt

10621 gtcccgcaaa tgtaatttgg attaatgata acttcgaagg aatgtattct tattatttat

10681 gtgttaatat gctttttttcc gtagaagatg ccgttacgga atttatttat cataaagtaa

10741 atgaattagg attaactttc gataatataa ctgttacagg gttttcaaag ggaggatctg

10801 cagctctata ttatgggctg aaattgcctg tagccaatat cgttgcaact gtacctcaaa

10861 caaaaatagg tagttatgta gttaagcatt ggaaacacgt agcagaacac atgatgggaa

10921 aaattacacc aacaagagtt tctcatttag ataaattaat tactcaaaag ttaaaacaag

10981 ataaaaaatt agatcgaaac atttacttat taacatcaga atcagacatt caatttccaa

11041 ctgaaattgt aactattctt aatgacttaa gaaaatacag taactttaat ttattaaaaa

11101 cattttcttg ttttgcaaga gagcataatc aaattaaatc tcatcataca gcattattat

11161 taggaattta ttattcctta gcctctgaag ctactcctag atttaataac ggcgaagtca

11221 atttctttgg tattcaacct ctccctccaa aagaaataac tggagagcca tttatcgatc

11281 tacgtaaggc agaaattaaa gataatttgc tatttgttga tggagttgca atcttaagag

11341 gttatgattt ggtagaatat tctgatgtgg aatatcagtt aatttttaaa tcagaaagga

11401 ataatattgt taaaaacttg gctaagacac ataaaccgca attaacaaga gagctgtttg

11461 atggagaaaa tctagttatt tatgataagg gttggtttac cacatatcaa tataaaggta

11521 ttgatatttc tgatattcct ccaggtaaat atagtgtata tattaacatt aacttatcgg

11581 ataaatcagc ttgcttgcca ctgacaacaa agttaaataa gatcatgacg aataacaaat

11641 gtacattaca tgtagtaaat aacattgttt attttgaaaa ataacaataa agctaggcta

11701 accttagtct agcttttatt ttacggagaa tataatgaat attaaagaaa tatataaaaa

11761 gataaagaat gaattaccag ttgagtatga tataaaaata tatcaagaac aatccattta

11821 ttgggagaaa ataatcaact cacctcattc aaaagataaa tataacctag caataagttt

11881 aaatattaaa gacaaaataa tttatttata tagacgagat aaagaagaat taaaagacat

11941 aaatgaaata atacctaact ttagactgtc agaaagaaat aatgtattct tccataatat

12001 agaaaactat tccttaaatg aatcatgtga gatattctat attttagtta aaaatgccga

12061 agattaccta aaaaatatcc taatacaaaa tacaaaacaa aagaacgtta tttcaggaga

12121 ctattatgat gatatggata ataaaatttc agcacctata gggttagtca acgtacattt

12181 ccaattttta ggatcaaaca ataaaattct tatttcccca aaagcaaatt taaaaaatac

12241 atttatagaa tgccgaggta ataataatac tattataatt gatgagaatg taagaatggt

12301 agggcattgg cggttaggtt ttggctgcac cttaaaaatt ggtaagaact ctagtagcac

12361 aaatcctgtt tatattactg ttgcagaaaa tactcaacta actataggtg aagactgtat

12421 gtttgcaaca aataatcaaa ttcgtactga tgatgctcat ccaatttacg atgtaaacac

12481 aggcaaacga gttaatatgt ctaaagatat tcaaataggc gaccatgttt ggattggata

12541 tggagcaaca attttatctg gctcggcaat aggctccggc tcagttatcg gagcaggctc

12601 aattgtacga aacaagtttc ctaataattg tgttatagcg ggtacccccg caaaagtggt

12661 aaaaaaagat attttttggg aaagaccatt actcttgaat atgagcgaag aggttgtgta

12721 ttccgaagaa gaaagaagac agaaaaacta ctgtaaaaat actatggaaa cagagtaata

12781 ggttatattt ttaattatta tacatcatga gttaattttt ataactcatg atctccttaa

12841 agtgagttat tatgaataat caatttatcc cgacagatgt gactaagata tcttatgaaa

12901 aaataagcgg cttactacaa gctagaaaaa tcaatgttta tcatcctatt caagcacctc

12961 agacaataat agaccgctta ttttattcat actcttatat aaaattaggg tttattatgg

13021 gcgtaaccac atacaatcat aaggatatgc ggtatagcaa tattgatata atccgagaaa

13081 gtggaagcct gtttttctcc acttacctgc ttctgcattt gcagcagtag cagagaaaaa

13141 gactgaacac gcagcaattg cgataagtgc ttttgataaa aatgttttct tcataaaatg

13201 ctccttaatg tagcaataca acaatgacta aaatgcacac aactttagat taagtaatat

13261 gcaacttcat tttattatta atttgtataa ttgaaattag accacattat tactattttt

13321 aatgaacaaa gaataagaaa ttatgtctaa attgtctgtt taaaataagc cttttatatt

13381 taatcttatt taaaatccat tcaatattta tatactccac tcaatttaac attatcttaa

13441 tcaaatttat tttttcgttt tttagacatt aagctgttat tttcttcgtt atttaattct

13501 gccaaatctc ccctatcccc tctttactaa agaggggggat ttcctttaga tattaacaag

13561 ctatgatctt tcgaatagct ttggtctctc aatagattca ggtgaatgat cgatatataa

13621 ggaaaataaa aacattcgtc gaagacgagc gccatcgtaa agaaatttgg agccacagca

13681 tggctcactc agccgtaggc tgatcgtaac cacgtacggc ttgccgtgcg tggctgttgg

13741 gtgattattt aaaaacaggc gaaacggctt ttttactttt tcattaccgc aatttgtttt

13801 agaatcaacg gacgatttac tctcaaggat ttatgatgaa aaaattactt ttggcggtgt

13861 taatcgcatc gttcgggttg gctgcctgcg gtgtaaaagg cccgctctat tttcccgagc

13921 agcaaccggc tcaacaacaa acaaaataat tgctaaccac ggtaattcaa aatctacgta

13981 taacaagcgg tcggatttat ccgatttttt gtaaatacaa gtcatcaatc aatacggtac

14041 accggcttat atctattctc gtgctacgct tgagcgtcat tggcacgcct cggttcgggc

14101 tttgatattg tgtcgcaagg cgaacttgaa cgtgtacttg ccgccggcgg cgagccgagc

14161 aaagtggtat tttccggtgt ggcaaaatca catagcgaaa ttcaacgtgc attggaagtc

14221 ggcattcgtt gttttaatat cgaatccatc gccgagttac accgcattaa tgaagttgcc

14281 ggtcaattag gtaaaatcgc accgatttca ttgcgtgtaa atccggatgt ggatgcacat

14341 actcaccctt atatttccac cggtttaaaa gaaaataaat ttggggtaag cgtaacgaac

14401 gagggaaaca agtactggga gaagcgttac gagaaaaaac acaggcggaa aacctactgc

14461 gtgaaagtgc gctaaattgg acgattctcc gcccgtgcgg gctgaatacg gacgaaggcg

14521 aaacctttcg tttaattgaa aatgcggctg aactgcccgg cagttatatg agccgtaaag

14581 cattagccaa tgcggtcttg tccatactta acagtgaaaa cacaaactat aaaatcttct

14641 cagtctgtgc ctaacttcac aatccctttc actttggcac aagccatccg cttgtgccta

14701 tttcaaaatt tgttttaaca gttccaataa aaccggtaat tccgttactc cctctcgatc

14761 gataaaatgc ccgcccgttg ccaagcgaat ataatccgct tgtaagtatt gcgctaatcg

14821 atcgctgaac gaatggggaa cgacaacatc atttaatgca gatatgacgt aagacttttg

14881 cggtaaacaa gcggtctgat ttgcataaaa atctgcaaag ctatctaatt ccggcaaagt

14941 tggtaatttc tcataaaagc cggaaacaaa aattgccgtt tttacttttt gctgcgttac

15001 cgcaagataa ttcagtaacg caatgcagcc caaactatgt ccgatgagta aggtattttc

15061 atctaattga agtgtatttt ggtgatgttc cagccatgct tgcggattcg gctgatcgga

15121 attcggcatc gctaaacatt cacattccca tcctaatttt tccaattcgt ttttaagcca

15181 cggaaaccaa tttcttgtcg ggttcgccgt ataaccgtgc gttacatata cttttttcat

//

LOCUS **ADXN01000030.1** 17051 bp DNA linear BCT 20-APR-2021

DEFINITION Actinobacillus pleuropneumoniae serovar 2 str. 4226

capsular polysaccharide gene locus, complete sequence.

ACCESSION ADXN01000030 REGION: 55555..72605

VERSION ADXN01000030.1

KEYWORDS .

SOURCE Actinobacillus pleuropneumoniae serovar 2 str. 4226

ORGANISM Actinobacillus pleuropneumoniae serovar 2 str. 4226

Bacteria; Proteobacteria; Gammaproteobacteria; Pasteurellales;

Pasteurellaceae; Actinobacillus.

REFERENCE 1 (bases 1 to 17051)

AUTHORS Zhan,B., Angen,O., Hedegaard,J., Bendixen,C. and Panitz,F.

TITLE Draft genome sequences of Actinobacillus pleuropneumoniae serotypes

2 and 6

JOURNAL J. Bacteriol. 192 (21), 5846-5847 (2010)

PUBMED 20802047

REFERENCE 2 (bases 1 to 17051)

AUTHORS Zhan,B., Hedegaard,J., Andersen,P.K., Bendixen,C. and Panitz,F.

TITLE Direct Submission

JOURNAL Submitted (01-JUL-2010) Department of Genetics and Biotechnology,

Faculty of Agricultural Sciences, Aarhus University, Blichers Alle

20, Tjele 8830, Denmark

FEATURES Location/Qualifiers

source 1..17051

/organism="Actinobacillus pleuropneumoniae serovar 2 str.

4226"

/mol_type="genomic DNA"

/submitter_seqid="Contig36"

/strain="4226"

/serovar="2"

/note="K locus: KL02"

/db_xref="taxon:754254"

CDS 1..1458

/gene="modF"

/locus_tag="APP2_2151"

/codon_start=1

/transl_table=11

/product="putative molybdenum transport ATP-binding

protein ModF"

/protein_id="EFL77928.1"

/translation="MSNINIQNALFSLAQHNKLSIESLEINTHDFWVIVGGNGSGKTA

FAQALHNSLSLYSGEYQNSFQHIALLSFEQQQKIIEQIFKHRNNDMVSPDDFGLTARQ

IILNGSEKMQLCEEYAAKLRIQPLLDRPFIQLSTGESRKVLFCQMLVSEPDLLILDEP

FEGLDQASVAYWQDVMAQLGKQMAVVLISNRFNDIPDCATHIALLDNLQLILQGERQA

IEQQAVYSQLKFAEQNVNAPLLESATPLIQLPPNTNPFELKNVMIRYGEKTIIDDLTW

TVAPKQHWWIKGPNGAGKSTLLSIITGDHPQAYANYVHLFGRQRGSGETIWDIKKNIG

YVSSQLHMDYRVNCSALDVILSGFFDSIGVYQQVPSALQLKVMEWLERLHLANLAKKP

FRSLSWGQQRLLLITRAMVKHPPILILDEPLQGLDGVNRKLVKQFIEQLVTNSQTQLL

FVSHQDADAPNCITYLFEFVPQTNGGYRYVQTALN"

CDS complement(1657..2307)

/gene="cpxA"

/locus_tag="APP2_2152"

/codon_start=1

/transl_table=11

/product="capsule polysaccharide export transport system

ATP-binding protein"

/protein_id="EFL77929.1"

/translation="MISVKNVSKDYYTRSGKKTVLQDINFELKKGEKIGILGRNGAGK

STLIRLLSGVEPPTSGTIERNMSISWPLAFSGAFQGSLTGMDNLRFICRIYNADIEYV
KAFTEEFSELGDYLYEPVKKYSSGMKARLAFALSLSVEFDCYLIDEVIAVGDSRFAAK
CKHELFEKRKDRSIILVSHSPSAMKSYCDNAMVLDKGIMYKFENMDEAYKFYNSTL"

CDS complement(2304..3101)
/gene="cpxB"
/locus_tag="APP2_2153"
/codon_start=1
/transl_table=11
/product="capsule polysaccharide export inner-membrane
protein"
/protein_id="EFL77930.1"
/translation="MQYGDQTTFRQSLAIQGRVIGALLMREIITRYGRKNLGFLWLFV
EPLLLTLFIVLMWKFIRADRVSDLNIIAFVITGYPMAMMWRNASNRTIGAISGNLSLL
YHRNVRVLDTLLARVILEVAGATIAQIIIMALVILLGWIEMPKDTFYMVMAWVLMAFF
ALGLGLIICSIAQKFEAFGKIWGTLSFVLLPLSGAFFFVHALPSQAQQYATLIPMIHG
TEMFRHGYFGDSVITYESISYLVICDVAMLLFGLIMVKNFSKGIEPQ"

CDS complement(3101..4258)
/gene="cpxC"
/locus_tag="APP2_2154"
/codon_start=1
/transl_table=11
/product="capsule polysaccharide export transport system
permease protein"
/protein_id="EFL77931.1"
/translation="METPIATSPAEKLQKPVKQKKSRFKKLNPLFWITVAIPTVLSAF
YFGSVASDIYISESSFVVRSPKNQTALTGVGALLQGSGFSRSQDDTYTVQEYMRSRTA
LEQLMQGLPVREYYENQGDIIARFNGFGLNNSKEAFYKYFRDRLSVDFDSVSGIASLR
IRAFNAEEGQQINQKLLAEGETLINRLNERARKDTISFAEQAVTEAENNVNETANALS
KYRIKNKIFDLPAQSGVQLSLISSLKSELIRVETQLAQLQSITPDNPQVDALLMRQKS
LRKEIDEQSKQLSSNSNSSIAIQTADYQRLVLANELAQQQLTAALTSLQNTKNEADRQ
QLYLEVISQPSKPDWAEEPYRLYNILATFFIGLMLYGVLSLLIASVREHKN"

CDS complement(4284..5471)
/gene="cpxD"
/locus_tag="APP2_2155"
/codon_start=1
/transl_table=11
/product="HexD, capsule biosynthetic locus protein"
/protein_id="EFL77932.1"
/translation="MEIKKYNSIIGLALTTLFLSACSSLPTSGPSHSAILEANSQSSD

KPLPEVNVVELDNGLVQQLYQTQQSQQFSGFLGTVGSAGYAGAVNVGDVLEISIWEAP

PAVLFGGTFSSEGQGSGHLTQLPAQMVNQNGTVTVPFVGNIRVAGKTPETIQSQIVGA

LQRKANQPQALVKIANNNSADVTVIRQGNSIRMPLTANNERVLDAVAAVGGTTENIED

VTVKLTRGSEVKTLAFETLISDPAQNIMLRAGDVVSLLNTPYSFTGLGAVGNNQQMKF

SSKGITLAEAIGKMGGLIDTRSDPRGVFVFRHVPFAQLSLEQQAQWQAKGYAIGMDVP

TVYRVNLLEPQSMFLLQRFPMQDKDIVYVSNAPLSEFQKFLRMIFSITSPVTSTTNAV

RAY"

CDS 5656..6792

/gene="cps2A"

/locus_tag="APP2_2156"

/codon_start=1

/transl_table=11

/product="Cps2A"

/protein_id="EFL77933.1"

/translation="MKIAFIAWNSFQVLHFKPLLQALPCALLIIEKRRRSVPICKDIL

RDINNNIAYIRHTDIYAKIDGNFDVLVAQTTFEQLYLFHRTKIALLQYGYAKEPYNYG

TWRAFADLNLVYGNYAYERISYFSPTKITGCPRYDLWYQPLFHQKAKENYARVLDTSK

KTIVYAPSWGELSSFKLYIEEITKLSLFYNVLVKLHHNTLLLANKHQNYEKLYPNLHF

FYEGEDLLSLISVADIVISDFSGAIFDAIFCKKPVILFSIPLVDQPKLDKFSLEIAHR

SALGYEVSSPERVAITVEKALTEQKLADKMLYQQLFMGNENATQQVIDALQQLVDGKY

SLSQQQLYVRQTEKLLNIEKIKQQKNKKQSFNKIRQISKKLIKK"

CDS 6846..7274

/gene="cps2B"

/locus_tag="APP2_2157"

/codon_start=1

/transl_table=11

/product="glycerol-3-phosphate cytidylyltransferase"

/protein_id="EFL77934.1"

/translation="MKKVLTYGTFDLLHHGHIRLLERARSLGDHLTVAISTDQFNLGK

GKVCAYTYEERAHILKAIRYVDEVIPETNWEQKVEDVKNHEIDVFVMGDDWEGKFDFL

ADYCEVVYLPRTPDISTTQVKKMLAKKDLAAGQKQIHEKE"

CDS 7276..8427

/gene="cps2C"

/locus_tag="APP2_2158"

/codon_start=1

/transl_table=11

/product="Cps7C"

/protein_id="EFL77935.1"

/translation="MLMFQILKKKLPTLQRVLRESDSQCSLLSYWYGLNLLSALERAD

HVHVRKLADKMLNKGINIGHYFLAQSYFLCGEYTLAEQAVKKIPNFTKIPEVVFLYSD
ILIKCQRREEAWLLLEQCALLNKRKKVWIHLTNLVNTEADYRHLEQHIDKVRTTTPYL
KSDLLIHQRTNAALRAGLTETALALTELNPLPKQAKVKKKTTAYSDKLAAIALADLKK
VLDHKKIPFFLISGTLLGCIREGKLLGHDKDIDVGVWDEYSYEELANCLSTSGYFYVV
PTRTKHLVMLRHVNGIAIDVFIHYRESNDYWHAGVKIKWHNSPFNLVYTNFLGQQYLI
PENYDLYLTENYGDWRTPKTQFDSAFDTPNMEVINEVEMQVYINKYYKE"

CDS 8430..12293

/gene="cps2D"

/locus_tag="APP2_2159"

/codon_start=1

/transl_table=11

/product="hypothetical protein"

/protein_id="EFL77936.1"

/translation="MKKAFYKATRLLNTPVIYWKGLHFYQKKDWEKAKYYFELAVQKK
PEHAYSNFKLGMCFFKQGVWDKAYHYISIATNLAPEILQWQVQLRQSEVRIRLKKHKK
KSVIGKTSVHKIVEEQQKINIDEIKRITSSSANDIAEQIIIDALEKEPENASLYAELA
SFQNKQNKLWQSVDSWGEAISRDSVHAEWFYQYGIVLEKLGHFLQASKAYEQAKSLSM
KENLSDLYFRLGFVNENQGHDNEIDLEVAKQAYGLAIQADRKLRAKDFGIGVFHEHRR
DWGRAIIAYKAQLEITPNNPELLYRLGFAYDRNYQFGQAENIYKEALSLKQKPEWRFR
LGFVQERQNKFDQATINYEQAATERKQYTPYWFYRSAYTLEKQGLYEKASKMYLKLRK
NTELALKNKIHNKEAVEKLIFILNKKHQYDASSAESWFDLGTYYEFLNDWEQAELAYY
QAVSRSEILNSLGYYRLAFVQLKQNKYEEACENFRNYRVMQRPHGVNEDYLSKDIGFA
EAASYSEYYNVLKIKDKTILYESFSGQGMSCNPYALFLYLLNHQEYKSWTHIWVVNNI
DNISSEYKKQHNIIFVSRGSDSYLRYLATAKVLINNSNFPPYFIRKPEQKFLSTWHGT
PFKTLGRDMEGRFFEHKNLTRNIFQSTHLLSPNAHTSKILYDRHEIKEIYTGKLIESG
YPRIDMTLSLTEEEKLELREKLGVLNNEKLVFYAPTWRGTHGDIEFDYDKLKSDLNKL
SKLKGAKVIFRGHSLLQEALSKINLDITVAPDELDTNKILGVTDILITDYSSVLFDYL
PTLKPLVLYMYDIKEYTEERGLYFSENELPGEKCYNIDELVKTLTYLLENNITSVSVE
DNKVAEFAPHDDGNVSEKVINALFSDDYTDLKIINDIPENKRSLLIYGGPFMGNGITT
SVINLIANIDRSKYTITLVIDPGSIEKEVGRLIQFEKLPQDINVVARVGRMDMDLEER
YIHGLNNQHYELQSSVAQDILWNSWEKEYQRIFGNAKFDSLIQFEGYNRFWSGVFTSI
KNKKSSIYMHNSMEEEYRLKYPYLKSIFYYCSLADKVISVSELTMKLNQDKLSDRFNI
PLSKFDYSDNLQQPEKIKVLARDELLEQDKAYFNTEDKVFLTIGRLSIEKDHAKLINS
FANVVKKYPKTQLLIIGDGSLRYPLVQQIKQLGLEKNVHLLGLRANPFPLLKKADCFI
LPSNHEGQPMTLFEAMILEKMIIATDIVGSRSALEGRSGYLVENSVSGLEKGMLDYIS
GSLPLVTYDINEYQKQAINKFYSIV"

CDS 12304..13572

/gene="cps2E"

/locus_tag="APP2_2160"

/codon_start=1

/transl_table=11

/product="hypothetical protein"

/protein_id="EFL77937.1"

/translation="MKHSVIGIRTYQWTNEEEVLHKRLLEYFACDSIFIVVDEINKKE

VKFPDYVNKIVLNEEFLDSEGILSSHPTQKGIGWLCGDYFYYALREKVDSKFYWLIEP

DVGFTFDSLSKFFIRFEECDDDALVQSFQKAPEDWMWKNPAELISPQGYKSFFPLTRL

SKRAIDDCKKARKLLTEQLKKNKFDINQYPNDEALVATVIGNNELLSIKNLRTFFPKS

FKYFTYMQNISVFPKANEILPLNQVLHPVRDINYASNILVKKLEKELFSSTEISDFLQ

KFLISSDDYEDFSKEVLRKSQNILIQMLERNESSFKNYRLILEKVLDLYPNLSDNSHV

WIWKDKVLVLDYSFLDNIFTLEFDFSKENLVCNVFTRKGNINLIFLINQSKKNIKNNK

IEVFAEPIGDIRLSIDKGVSYFYSLIRDFY"

CDS 13582..14925

/gene="cps2F"

/locus_tag="APP2_2161"

/codon_start=1

/transl_table=11

/product="hypothetical protein"

/protein_id="EFL77938.1"

/translation="MDNCFKWSVVSTVKASIELIHNFINYYKGIGADKIYLYLDDVND

KDILNEYQKDKNVIITLCDDAYWNIDYGFDNFKFIGRPESIEERQKHNLCHVLKFCET

EWLLSVDIDELIFSEYAISDLLLTIPENVFSLRIKPYEATYELCEPKSIKEVFLTKYF

KHREKRFDDFFWNSVLPKESFHREGFFGHTVGKAFLRTSKDLKVPGIHNQYPLDSSLI

DNFWFKEIKLLHFEALTPDIFIQKNINRANNIFKVSQLGRLESERVKYIADLYKQSGV

EGLYKLYSSMHVLSSNVIESAKKLFLLEEIDINKQGHRNIGNYLTTIHNTIIVYNEEV

NKVQSIEFNAIDSNNYSPVFFNFDINKGIGYFFFIKDKISYYLFPDENGYLVSYPSRK

SIFYGVEVLDKINTQFAIFVLSSKLYLTITPKGEVRFRAEQVQAWEKISLLDDVN"

CDS 14970..15185

/gene="cps2G"

/locus_tag="APP2_2162"

/codon_start=1

/transl_table=11

/product="hypothetical protein"

/protein_id="EFL77939.1"

/translation="MKIFITDGAGFIGSAVVRYLIQQTNHSVVSLLDKLTYAGNLVSL

QAVENNPRYAFEQVDICDKSVLENIFM"

CDS 15883..16437

/locus_tag="APP2_2163"

/codon_start=1

/transl_table=11

/product="hypothetical protein"

/protein_id="EFL77940.1"

/translation="MFKKITLFSFIALIAGCSSSSQPEAFPGEFANADYVLSDKDAQR

WVVASHQAEQCIYPNLTRIQQQAFSKEDSYIHSQYVFFYPLEEIIGEQYVKIIQDDEK

SMGYAQYLFKKFRDNQEFEPLADKQCLVLREKAKNDLAVVKGQYKSGMVEETTSEAKN

ADGVATNQNKFFFDIIKWGSMLLL"

CDS complement(16509..17051)

/gene="ydeN"

/locus_tag="APP2_2164"

/codon_start=1

/transl_table=11

/product="esterase of the alpha/beta hydrolase fold"

/protein_id="EFL77941.1"

/translation="MKKVYVTHGYTANPTRNWFPWLKNELEKLGWECECLAMPNSDQP

NPQAWLEHHQNTLQLDENTLLIGHSLGCIALLNYLAVTQQKVKTAIFVSGFYEKLPAL

PELDSFADFYANQTACLPQKSYVISALNDTVVPHSFSDRLAQYLQADYIRLETGGHFV

DREGVTELPKLLELIKQISN"

ORIGIN

1 atgtcaaaca tcaacatcca gaatgcctta ttttcccttg ctcaacacaa taaactgtcg

61 attgaatcgc tggaaatcaa tactcacgat ttctgggtga ttgtcggcgg taacggctcg

121 ggtaaaactg ctttcgccca agcgctacat aattcacttt cactatattc gggcgaatat

181 caaaacagct tccaacatat cgctttactt tccttcgagc agcaacaaaa aatcatcgag

241 caaatcttta aacaccgtaa caatgatatg gtttcgcctg acgatttcgg tttaaccgcc

301 cgtcaaatta tcttaaacgg tagcgaaaaa atgcaattat gcgaggaata tgcggctaaa

361 ttacgtattc agccgttatt agatcgcccg tttattcagc tctccaccgg tgaaagtcgc

421 aaggtgttat tttgccaaat gttagttagc gaaccggatt tgctgatttt agatgagcct

481 tttgaggggt tagaccaagc ctcggtcgct tattggcaag acgtgatggc acaactcggc

541 aagcaaatgg cggtggtact gatttccaac cgttttaatg atattcccga ctgtgccaca

601 catattgctt tactggataa cttacaactg attttacaag gcgaacgcca agcgattgag

661 caacaagcgg tctattctca gctaaaattt gcagaacaga atgtgaatgc accgttgctg

721 gagagtgcca caccgctgat tcaactccca ccgaatacta atccgtttga actgaaaaac

781 gtaatgatcc gttacggcga aaaaacgatt attgatgatc taacttggac agttgcccca

841 aaacaacatt ggtggattaa aggcccgaac ggagcaggga aatcgacctt gctttcgatt

901 attaccggcg atcatccgca agcctatgct aattacgtac atttattcgg acgtcagcgt

961 ggctccggcg aaacgatttg ggatattaag aaaaatatcg gctatgtaag tagtcaatta

1021 catatggatt atcgggtgaa ttgttcggcg ttagatgtga ttttatctgg cttttttgat

1081 tcaatcggtg tttatcaaca agtgccaagt gccttacagc tcaaagtaat ggaatggtta

1141 gaacgcttgc atttagccaa tctggcgaaa aaaccgttcc gttcactttc gtgggggcaa

1201 caacggttat tattgattac tcgtgctatg gtaaaacacc cgccgattct gattttagac

1261 gaaccgctgc aaggtttgga cggtgtaaac cgcaaattgg ttaaacaatt tatcgaacag

1321 cttgtgacta atagtcaaac ccagttgcta tttgtttcgc accaagatgc ggacgcccca

1381 aattgcatca cgtatttatt tgaatttgtt ccgcaaacta acggtggtta ccgttatgtg

1441 cagacggcgt taaattaggt ttttgacctt taaaggaaat ccccctcttt agtaaagagg

1501 gattagggga gatttgtcaa tagagagata tgaaattgaa tagaatttca ttttttatat

1561 ttataaaagc gttaattagc atatttcttc gctaattcat tctgtcaaat ctcctcctgc

1621 ccctctttgc taaagagggg agatatgtgc gggactttaa agcgttgaat tatagaactt

1681 ataagcctcg tccatattct caaatttata cataatccct ttatctaata ccattgcatt

1741 atcgcaataa gacttcattg ctgacggact atgcgaaacc aaaataatcg aacgatcttt

1801 gcgcttttca aataattcat gtttacattt tgccgcaaag cgagagtcac ctaccgcaat

1861 tacctcatca attaagtagc aatcaaactc taccgaaagc gacaaagcaa aggcaagtcg

1921 ggctttcatg ccggaggaat atttcttaac cggctcatat aaataatcac ccaattcgga

1981 aaattcttcg gtaaaggctt taacgtattc aatatccgca ttatatatac ggcaaataaa

2041 gcgtaaatta tccataccgg ttaaactgcc ttggaacgcc ccgctgaaag cgagcggcca

2101 agatatcgac atattacgtt cgatagtacc tgatgttggc ggctcaacac cacttaacaa

2161 acggattagc gttgatttcc ctgcaccgtt acgccctaaa ataccgattt tctcgccttt

2221 tttcagctca aaattaatat cttgcaatac ggttttttta ccgcttcgag tatagtaatc

2281 tttactcaca ttttttacgc taatcattgc ggttcgattc ctttactgaa gtttttttacc

2341 ataatgagcc caaaaagtaa catggctaca tcacatatta cgagatagct tatactttca

2401 tatgtgataa cactgtcgcc aaaataaccg tgacgaaaca tttccgtgcc gtgaatcatc

2461 ggtattaagg ttgcatattg ttgagcttgg cttggtagcg catgcacaaa gaaaaatgcg

2521 cctgaaagag gtaaaagaac aaagcttaat gttccccaga ttttgccaaa tgcttcaaat

2581 ttttgtgcaa tagaacaaat aatcaagcct aatcctaatg caaaaaatgc cattaatacc

2641 cacgccataa ccatataaaa cgtatctttc ggcatttcta tccagcctaa taaaatgact

2701 aatgccataa taatgatttg ggcaatcgtt gcacccgcta cctcaagtat gacacgagcc

2761 agtaaggtat ctaatacgcg aacattacga tgataaagaa gactcaagtt accggaaatt

2821 gcaccgatag tgcggtttga tgcattacgc cacatcattg ccattggata accggtaatc

2881 acaaaagcaa taatatttaa atcggaaacg cgatccgctc ggataaattt ccacatcaaa

2941 acgataaata aagtgagtaa tagcggctca acaaacagcc ataaaaaacc caaatttttt

3001 cgtccgtaac gcgtaataat ttcccgcatg agtaatgcac cgattactct cccttgaatg

3061 gcgagagatt ggcggaaagt tgtttgatca ccgtattgca ttagtttttg tgctctctta

3121 cgcttgcaat taataaactt aatacaccat aaagcatcag accgataaag aatgtcgcta

3181 aaatattata taagcgataa ggctcttccg cccagtccgg tttgcttggc tgactgatta

3241 cttctaaata aagttgctgg cgatccgctt catttttcgt attttgtaat gaggttaatg

3301 ctgcggtcaa ttgttgctgt gccagctcgt ttgcaagtac taagcgttgg taatcggcag

3361 tttgaatagc aatagagcta ttactgttac tggaaagctg ttttgattgc tcgtcaattt

3421 ccttacgtaa acttttttgg cgcataagca atgcatcaac ttgcgggttg tccggtgtaa

3481 tagattgcaa ttgagccaat tgtgtttcca cacgaatcaa ctcgcttttt aagcttgaaa

3541 ttaatgaaag ttgtacgccc gattgtgccg gtaaatcaaa aattttattt ttgatacggt

3601 atttacttaa agcatttgcc gtttcgttta cattattttc cgcttctgta accgcttgtt

3661 ccgcaaatga aatggtatct tttcttgcac gttcgtttaa acggttaata agcgtttcac

3721 cttcggcaag gagtttttga ttaatttgtt gcccctcttc cgcgttaaat gctcgaatac

3781 gtaagctggc gataccggaa acagagtcaa agtccacact taagcgatct cggaaatatt

3841 tataaaacgc ttctttactg ttatttaatc cgaatccatt aaagcgagcg ataatatcgc

3901 cctgattttc ataatattcg cgaacaggta atccttgcat tagttgctca agagcggtac

3961 gagaacgcat atattcctgt acggtataag tatcgtcttg cgaacgagaa aaacctgaac

4021 cttgcaataa ggcgcctacg cctgtcagag cggtctgatt tttaggcgat ctaacgacaa

4081 aacttgattc cgaaatataa atatcagaag cgaccgaacc gaaataaaac gctgatagca

4141 cggtagggat tgcgacagtt atccaaaata atggattgag ttttttaaag cggctttttt

4201 tctgtttaac cggtttctgt aatttttctg ctggactggt agcaataggt gtttccatct

4261 tttgtcctta tacattcaat atattaatag gcacgaacgg cattggtcgt actggtaacc

4321 ggcgaagtaa ttgagaaaat cattctcaag aatttttgga attcagacaa cggcgcgttt

4381 gaaacataca caatatcttt atcttgcatt gggaaacgct gtaataaaaa catggattgc

4441 ggctcaagta agttcacacg ataaaccgtt ggtacatcca ttcctatagc gtagccttta

4501 gcttgccatt gtgcttgttg ttccaaactc aattgtgcaa aaggcacgtg acggaatacg

4561 aaaacccctc tcggatccga acgagtatca attaaaccgc ccatcttacc gatagcttcg

4621 gcaagcgtaa ttcctttact tgagaatttc atttgctggt tgttacccac agcgcctaaa

4681 ccggtaaaac tataaggcgt gtttagcaac gaaacgacat cgccggcacg taacataata

4741 ttttgcgccg gatcggaaat taacgtttcg aatgcgagtg tttttacttc agaaccacgg

4801 gttagcttga ccgtcacatc ttcaatattt tccgttgtac cgcctactgc cgcaaccgca

4861 tctaatacgc gttcattatt agcggttaat ggcatacgaa tactattacc ttgacgaata

4921 accgtaacat ccgcagagtt attattcgca attttgacta atgcttgcgg ctgattcgct

4981 ttacgctgca atgccccaac aatttgagac tgaatcgttt caggtgtttt acctgcgaca

5041 cgaatatttc ccacaaacgg cacggttacc gtcccgtttt gattaaccat ttgtgccggt

5101 aattgcgtta aatgcccgct accttgtcct tcggaactaa aagtaccgcc aaacaacact

5161 gccggcggcg cttcccaaat tgaaatttca agtacatcac ccacattgac tgcaccggca

5221 tagcccgcgc tgcctactgt gcctaaaaat ccggaaaatt gttggctttg ctgagtttga

5281 tacaactgtt gaactaaacc gttatccagt tccaccacat ttacttccgg taagggttta

5341 tccgaacttt gtgaattagc ctctaagatc gcactatggc tagggcctga agttgggagg

5401 cttgagcaag cagaaaggaa caatgttgtt aaagctaacc ctatgattga attatatttt

5461 ttaatttcca tcttgtaaaa aggctctatt gaaaaagtgt gtaaaatgaa tataaactat

5521 acataattat agatacaaca ctataattat atattataat ctaatatctc gttttttaaa

5581 acgataacct aaaatattac ttttctatag aagaataggc attattttaa taaatataat

5641 taggtatgaa tgttgatgaa aatagcattt atcgcttgga atagttttca ggtgttacat

5701 ttcaagccct tattacaagc tttaccgtgt gcattattaa ttattgaaaa acggagacgt

5761 agtgtaccaa tctgtaagga tattttgcga gatataaaca ataatatcgc ttatatccgc

5821 catacggata tatatgcaaa aattgatggg aattttgatg ttctagttgc tcaaactact

5881 ttcgagcaac tttatttgtt tcaccgcacc aaaattgcat tgcttcaata cggatatgct

5941 aaggaaccgt ataactacgg cacttggaga gcatttgcag atctaaattt ggtttatggg

6001 aattatgcct atgaacgtat ttcctatttc tctccaacta aaataaccgg ttgtccacga

6061 tacgatttat ggtatcagcc tttatttcat caaaaagcga aagaaaatta tgcgagagta

6121 ttagatacga gtaagaaaac gattgtatat gcaccaagtt ggggagaatt atccagcttt

6181 aaattatata tagaagaaat tacgaaatta tctttatttt acaacgtgtt agtaaaatta

6241 caccataaca cgcttttatt agcaaacaag catcagaatt atgaaaaatt gtatccgaat

6301 ttacattttt tctatgaagg tgaagatctt ctctcactta tttcggtagc cgacattgtt

6361 atttccgatt ttagcggtgc gatctttgac gcaattttct gtaaaaaacc agtaatatta

6421 ttttctatcc cattagtaga tcaacccaaa ttagataaat ttagtttgga gatagcccat

6481 cgttcggcgt tgggatatga ggtttcttca cctgaacgag tagctataac ggtggagaaa

6541 gcactaacag agcaaaaatt agcagataaa atgctatatc aacagctttt tatgggtaat

6601 gaaaatgcaa cacaacaagt gatagatgct ctacaacagc tcgttgatgg aaaatattcc

6661 ttatcgcaac aacagttata tgtgcggcaa acagagaagt tattaaatat tgaaaaaata

6721 aagcaacaga aaaataaaaa acaatctttt aataagataa ggcagatttc taaaaagtta

6781 attaaaaaat aaatttagac attttatttt ttatatcttt ttacttaata atacggagtt

6841 taggaatgaa aaaagtatta acctatggaa cctttgattt attacaccat gggcatattc

6901 gtttattaga aagagcaaga tcattaggcg atcaccttac tgttgctatt tctaccgatc

6961 aatttaactt aggaaaaggc aaagtatgcg cttatactta cgaagagaga gcgcatattt

7021 taaaagcaat ccgttatgtg gatgaagtaa ttcctgaaac aaactgggag caaaaagttg

7081 aggatgtaaa aaatcacgaa attgacgtat ttgtaatggg tgatgactgg gaaggcaaat

7141 ttgacttctt agcagattac tgcgaagtgg tttatttacc gagaacccct gatatttcaa

7201 cgactcaggt aaaaaaaatg cttgcgaaaa aagatctcgc agccggacaa aaacaaatac

7261 acgaaaaaga gtaatttgtt gatgttccaa atcttaaaaa agaagttacc aactttgcaa

7321 agagttctga gagaaagtga ttctcagtgt tctttgctta gttattggta tgggcttaat

7381 ctactttccg cactggaaag ggcagatcac gttcacgtaa gaaaacttgc cgataaaatg

7441 cttaataaag gtattaatat cggacactat tttttagcac agagttattt tctatgtgga

7501 gaatatacgc tagcggagca agcggttaaa aaaatcccaa attttactaa aatacctgaa

7561 gttgtttttt tatattcaga tattcttatc aaatgccagc gtagagaaga agcttggcta

7621 ctattagaac aatgcgcttt actcaataaa agaaaaaaag tgtggataca tctaacaaat

7681 ttagtaaata ctgaggcgga ttatcgacac ttagaacaac atattgacaa agtaagaaca

7741 actacacctt acttgaagtc tgatttgtta attcatcaaa gaacaaatgc ggcattaaga

7801 gcaggtttaa cagaaacggc attagctcta acagagctta accctttgcc aaagcaagca

7861 aaagttaaga aaaaaacaac cgcttatagt gataaattag cggcaattgc gctagcggat

7921 ctaaagaaag tattagatca caaaaaaata cctttctttc tgattagcgg cacgttgcta

7981 ggttgtattc gagaaggaaa attattaggg catgataaag atattgatgt cggcgtttgg

8041 gatgagtact cctacgaaga attagcaaac tgtttatcta catcgggata cttttacgta

8101 gtaccaactc gaacaaaaca tttagtcatg ttaagacatg ttaatggtat cgcaattgat

8161 gtgtttatcc attatcgcga atctaatgat tattggcacg caggtgtcaa aataaaatgg

8221 cataactcgc cattcaactt ggtatataca aattttctcg gacaacaata tttaatacct

8281 gaaaattacg atttatacct aacagaaaat tacggtgatt ggcgtacgcc aaaaactcaa

8341 tttgatagtg catttgatac gccaaatatg gaagttatca atgaagtgga aatgcaagta

8401 tatataaata aatattataa ggaataaaaa tgaagaaggc tttttataaa gcaacacgct

8461 tattaaatac tcctgtgatt tattggaaag gtttacattt ttatcaaaaa aaggattggg

8521 aaaaagcgaa gtattatttt gaattagctg ttcagaagaa gcccgaacat gcatatagta

8581 attttaaatt aggaatgtgc ttttttaagc aaggagtgtg ggataaagca tatcactata

8641 tatctattgc aacaaatctt gctccagaga ttttgcagtg gcaagtgcaa ctacgtcaat

8701 ctgaagttcg tatacgattg aaaaagcata agaaaaaatc agttattgga aaaacatcag

8761 tacataaaat cgtagaggaa caacaaaaaa taaatataga tgaaataaaa cgtattacga

8821 gttcttcagc aaatgatatt gctgaacaaa ttattatcga tgctttggaa aaagaacctg

8881 aaaatgcttc cctttatgca gaattagcat cttttcagaa taagcaaaat aaactatggc

8941 aatcagtcga ttcatggggt gaggcaatta gtcgagattc tgtccatgca gaatggttct

9001 atcagtatgg gatagtatta gaaaaattag gacattttct ccaagcatct aaagcatatg

9061 agcaagctaa atctctttct atgaaagaga atttatctga tctctatttc cgtttagggt

9121 ttgtaaatga aaatcaagga catgataatg aaattgatct tgaagtagct aaacaggctt

9181 atggcttagc aattcaagcg gatcgaaaac ttagagcaaa ggattttggc attggtgtat

9241 ttcatgaaca tcgtagagat tggggaagag ctattattgc gtataaagct caattagaaa

9301 ttacgcctaa taatccagag ttattatatc gtttaggctt tgcttatgat cgcaattatc

9361 agtttggaca agctgaaaat atatataagg aagctctatc gttaaaacaa aaaccagaat

9421 ggcgtttccg attaggcttt gtacaagaaa gacaaaataa atttgatcaa gccactataa

9481 attatgaaca agcagccaca gagagaaaac aatatacacc atattggttt tatcgctcag

9541 catatacttt agaaaagcaa ggattatatg aaaaggcatc aaagatgtat ttaaaattaa

9601 gaaagaatac agaattagcc ttaaaaaata aaattcataa taaagaagca gtagaaaaat

9661 taatttttat tttaaataaa aagcatcaat acgatgcatc atcagctgaa tcatggtttg

9721 atttaggaac atattatgaa tttctaaatg attgggaaca agctgaatta gcttattatc

9781 aagcagtatc tcgtagcgaa attttaaatt cattggggta ttacagatta gcatttgttc

9841 aactcaaaca aaataagtat gaagaagcat gtgaaaactt tagaaattat cgtgtaatgc

9901 aacgcccaca cggggtaaat gaagattacc tttcaaaaga tattggattt gcagaagctg

9961 cttcatacag tgagtattat aatgttctta aaattaaaga taaaactatc ttatatgaga

10021 gtttttctgg tcaaggaatg agctgtaatc catatgcatt atttttatat ttattaaatc

10081 atcaagaata taaatcttgg actcatattt gggttgttaa taatatagat aatatttctt

10141 ctgaatataa aaaacaacat aatattattt ttgtatctcg tggaagtgat agttatttac

10201 gttatttggc aacagcaaaa gtacttatta ataatagtaa ttttcctcca tattttatta

10261 gaaaacctga acagaagttt ttaagtacat ggcacggaac gccatttaaa acattaggaa

10321 gagatatgga aggacgattt tttgaacata aaaatttaac tagaaatata tttcaatcta

10381 ctcatttatt aagtccaaat gcccatacat caaagattct ttatgatcgc catgagatta

10441 aagaaatcta tacaggaaag ttaattgaat caggctatcc tcgaattgat atgacattgt

10501 cattaactga agaggagaaa ttggagttaa gagagaagtt aggtgtttta aataatgaaa

10561 aattagtatt ttatgctcca acttggcggg ggactcacgg agatattgag tttgattatg

10621 ataaattaaa atcagattta aataagctta gtaaattaaa aggtgctaaa gttattttta

10681 gagggcattc attgcttcag gaggcattat ctaaaattaa tctagatatc acagttgccc

10741 ctgatgagtt agatacgaat aagattcttg gtgtaacaga tatattaatt actgattatt

10801 caagtgtttt atttgattat ctaccaacat taaagccatt agttctttat atgtatgaca

10861 tcaaagaata tacggaagaa cgaggattat acttttcaga aaatgaatta cctggagaga

10921 aatgctataa catagatgaa ttagttaaaa cattaacgta cttattagag aataatatta

10981 cgtcagtatc agttgaggat aataaagtag ctgagtttgc tcctcatgat gacggtaatg

11041 tttcagaaaa agttattaat gccttatttt cagatgacta tacagatctg aaaattatta

11101 atgatattcc agaaaataaa aggtctttgc tcatttatgg tggtcctttt atgggtaatg

11161 gaataacgac atcggtaatt aatcttattg ctaatattga ccgttcaaag tatacgataa

11221 cgttagtaat tgatccaggt tctattgaaa aagaggtcgg tcgattaata caatttgaaa

11281 aattaccgca agatataaat gttgttgccc gtgttgggag aatggatatg gatttagaag

11341 aaagatatat tcatggctta aataatcaac attatgaatt acaatcttca gtagctcaag

11401 atattttatg gaactcttgg gagaaggaat atcaacgcat atttggcaat gctaaatttg

11461 attcattaat tcaatttgaa ggatataatc gtttttggtc cggagtattt acatctatta

11521 agaataagaa gtctagtatt tatatgcata attcgatgga agaagaatat agattaaaat

11581 atccgtattt aaaatctatt ttctattatt gttcattagc agataaggta atttcagttt

11641 ctgaattaac aatgaaatta aatcaggata agttatcaga tagatttaat attccactat

11701 caaaatttga ttatagtgat aacttacagc aaccagaaaa aataaaagtt ttggctagag

11761 atgaactctt agaacaagat aaagcatatt ttaatacaga ggacaaagta tttttaacaa

11821 tagggcgttt atcaatagaa aaggatcatg ctaagctaat taatagtttt gccaacgttg

11881 ttaaaaaata tcctaaaact cagctattaa ttattggaga tggttcatta agatatcctc

11941 tagtacaaca aattaaacag ttagggctag aaaaaaatgt gcatctatta gggctaagag

12001 cgaatccatt cccattacta aagaaagcag attgctttat tctgccgtct aatcatgaag

12061 gtcagccaat gacattattt gaagcaatga ttctagagaa aatgattatt gcaacagata

12121 ttgtaggatc aagaagtgct ttagaaggac gttctggtta tttagtagaa aattcagtta

12181 gtggcttgga gaaaggaatg ctagattata tttctggatc attgcctctt gtaacttatg

12241 atattaatga gtaccaaaaa caggcaatta ataaatttta cagtattgta taaggggtaa

12301 ttaatgaaac attcagtaat tggtattaga acataccaat ggactaacga agaagaagta

12361 ttacataagc gtttattaga gtactttgct tgtgattcaa tttttatagt tgtagatgaa

12421 attaataaga aggaagttaa atttccagat tatgtgaata aaatagtatt gaatgaagaa

12481 ttcttagatt cagaaggtat tttatcgagc cacccaaccc aaaaaggaat tggctggtta

12541 tgtggcgact atttttatta tgcattaaga gaaaaagttg attctaagtt ctattggctt

12601 attgaacctg atgtaggatt tacttttgat tctctatcca agttttttat cagattcgaa

12661 gagtgtgatg atgatgctct ggttcaaagc ttccaaaaag ctccagagga ttggatgtgg

12721 aaaaatccag cagaattaat ttctccacaa ggatataaaa gtttttccc tctaactaga

12781 ttatcaaaaa gagcgattga tgattgtaag aaggcacgta aattactaac tgaacaatta

12841 aagaaaaata aatttgatat taaccaatat ccaaatgatg aagcgttagt tgcaacagtt

12901 attggtaata atgaattatt atcaatcaaa aacttgagaa ctttttttcc taaaagtttt

12961 aaatatttta catatatgca aaatatttct gttttcccaa aagctaacga aattttgcca

13021 ttaaatcagg tattacatcc tgtaagagat ataaattacg caagtaatat tcttgtaaag

13081 aaactagaaa aggagttatt ttcttcaacg gaaatatctg atttccttca aaagtttctt

13141 atatcgtcag atgattatga ggacttttct aaagaagtat taagaaagtc tcaaaacatt

13201 cttatccaaa tgttagagag aaatgaatct tcttttaaaa actacaggct cattttagaa

13261 aaagttttag atttgtatcc taatttatca gataatagcc atgtatggat atggaaagat

13321 aaagtattgg ttttagatta ttcatttttg gataatatat tcacactaga atttgatttt

13381 tcaaaagaaa atttagtttg taatgttttt acaagaaaag ggaatataaa cctaatattc

13441 ttaattaatc aatcaaagaa gaatattaaa aataataaaa tagaagtttt tgctgaacct

13501 attggagaca taagattatc tattgataaa ggcgtttctt atttttattc tttaatacgt

13561 gatttttatt aggagggtat tatggataat tgttttaaat ggagtgttgt ttctacagta

13621 aaagctagta ttgaattgat acataatttc attaactatt ataaaggtat tggtgcagat

13681 aaaatttatt tatatttaga tgatgtgaat gataaggata ttctgaatga ataccaaaaa

13741 gataaaaaatg taataatcac attatgcgat gatgcttact ggaatataga ttatggattt

13801 gataacttta agtttatcgg tcgtccagag agtattgagg aaagacaaaa acataattta

13861 tgccatgtat tgaagttttg tgaaacagag tggttattaa gtgttgatat tgatgagtta

13921 attttctcag aatacgcaat atctgattta ttattgacta tacccgagaa tgttttttca

13981 ttaagaatta aaccttatga agctacatat gaattatgcg agcctaaatc aattaaagaa

14041 gtatttctaa caaaatattt taagcataga gaaaaacgtt ttgatgattt tttttggaat

14101 tcagttcttc ctaaagagtc ctttcataga gaaggctttt ttgggcatac agttgggaaa

14161 gcatttttaa gaacatctaa agatctgaaa gttccaggaa tacataatca atacccttta

14221 gattctagct taatcgataa tttttggttt aaggagatta aattacttca ttttgaagca

14281 ttaacgccag atatttttat acagaaaaat ataaaccgtg ctaataatat atttaaagta

14341 tctcaattag gcagattaga gtcagaaaga gtgaaatata tagcagactt atataaacaa

14401 agtggagttg agggactata taaattatat tcgtctatgc atgttttatc ttcgaatgta

14461 attgaaagtg caaaaaaact ttttttatta gaagaaattg atattaataa gcaagggcat

14521 aggaatattg gtaactatct tacaacgata cataatacta ttattgtata taatgaagaa

14581 gtaaataagg tacaaagcat tgagtttaat gccatagatt caaataatta ctcacctgtt

14641 ttttttaact ttgatattaa taagggaata ggatatttct tttttattaa agataagatt

14701 agttattatt tatttcctga tgaaaatgga tatcttgttt cttatccatc tagaaaatct

14761 attttctacg gcgtagaagt attagataag ataaatacac aatttgctat ttttgttctt

14821 agctcgaaat tgtatttaac aattactcca aagggggaag taaggtttag agcagaacaa

14881 gttcaagctt gggaaaaaat atcgttatta gatgatgtta actagaaaaa tagtgttttt

14941 aaattaactg attttagatg ttatctaata tgaaaatctt catcacagat ggagcggggt

15001 ttattgggtc agcggtggtt cgttatctta tccaacaaac aaaccatagt gtggttagtt

15061 tgttggataa attaacgtat gcagggaatt tagtttcctt gcaagcggtt gaaaataatc

15121 ctcgctatgc ttttgaacag gttgatattt gtgataaatc agtattggaa aatatattta

15181 tgtaatatca acctgatttt gtaatgcatt tagctgtgga aaaccatgtt gatcgagcaa

15241 tcacagctct gctactttta ttgaaactaa tattgaagtg atatatttta tttgtatttt

15301 tttgaagggt ttgtaccaaa caagccgaat ggttttgcaa aatatgaaga tttgtttact

15361 gttgtatcag accgtctaaa acatgataaa cgctatatag ttaatgcgag gaaaattttt

15421 cgtaatttag tatcaaatta cctatttaca aactgttaaa tttacaggat ttttttgcag

15481 atttaatcgc ttgttgttta tagcaaaaaa taaaatcagt gtgaatggta ctagcataag

15541 atattgcttc gctaatttat tctgtcaaat tttcctcatc ccctcttttc tagagaggaa

15601 gtgcaaaata aataagttat aaggtttcaa atagttttag tcagaaacaa actctatcat

15661 agaaagattt aacggttata atgtggctaa ctcagctgta tatctatcaa gagtagattt

15721 ctatagcttc tactttatag tagcgtccta gaatgaataa gaaggtttcc atttgctaag

15781 taattacgat aaagagttct tttcttcatt aaatattgct taatctagtg atagaatatc

15841 aattagaata tatctatttt ctatcaagag taaaaacata ctatgttcaa aaaaatcaca

15901 ttattcagtt ttattgcgtt aatcgccggt tgttcttctt cctcacaacc ggaagctttc

15961 ccgggggagt ttgcgaatgc ggattatgtg ttatcggata aagatgccca gcgttgggtg

16021 gtggcgagcc atcaggcgga gcagtgtatt tatccgaact tgacgcggat tcagcaacaa

16081 gcgtttagta aggaagattc atatattcat tcgcaatacg tatttttcta tccgttggaa

16141 gaaattatcg gcgagcagta tgtaaagatt atccaagacg atgaaaaatc tatgggatat

16201 gcgcaatact tgtttaagaa attcagagat aatcaggaat tcgagccgtt agcggataag

16261 caatgtcttg tgttacgaga aaaagcgaag aacgatttag cggtcgtaaa agggcagtat

16321 aagagcggaa tggttgaaga aacgacgtcc gaagctaaaa atgcggacgg cgtggcgacc

16381 aatcaaaata aattcttctt tgatattatc aaatggggtt cgatgctatt actgtaattt

16441 gcggttagtg tgacgttaaa gagtataaaa acgagctgtc ggtaaatcgt tatcggcagc

16501 tctttttatt agtttgatat ttgtttgatt aactccaata acttcggtaa ttccgttact

16561 ccctctcgat cgacaaaatg cccgcctgtt tccaagcgaa tatagtcggc ttgtaggtat

16621 tgtgctaacc gatcactgaa cgaatggggt acaaccgtat catttaatgc ggatatgacg

16681 taagacttct gtggtaaaca agcggtctga tttgcataaa aatctgcaaa gctatctaat

16741 tccggcaaag ccggtaattt ctcataaaag ccggaaacaa aaattgccgt ttttactttt

16801 tgctgcgtta ccgccagata attcagtaac gcaatgcagc ccaaactatg tccgatgagt

16861 aaggtatttt catctaattg aagtgtattt tggtgatgtt ccagccatgc ttgcggattc

16921 ggctgatcgg aattcggcat cgctaaacat tcacattccc atcctaattt ttccaattcg

16981 tttttaagcc acggaaacca atttcttgtc gggttcgccg tataaccgtg cgttacatat

17041 actttttca t

//

LOCUS **CP000687.1** 15691 bp DNA linear BCT 20-APR-2021

DEFINITION Actinobacillus pleuropneumoniae serovar 3 str. JL03

capsular polysaccharide gene locus, complete sequence.

ACCESSION CP000687 REGION: complement(1762065..1777755)

VERSION CP000687.1

KEYWORDS .

SOURCE Actinobacillus pleuropneumoniae serovar 3 str. JL03

ORGANISM Actinobacillus pleuropneumoniae serovar 3 str. JL03

Bacteria; Proteobacteria; Gammaproteobacteria; Pasteurellales;

Pasteurellaceae; Actinobacillus.

REFERENCE 1 (bases 1 to 15691)

AUTHORS Xu,Z., Zhou,Y., Li,L., Zhou,R., Xiao,S., Wan,Y., Zhang,S., Wang,K.,

Li,W., Li,L., Jin,H., Kang,M., Dalai,B., Li,T., Liu,L., Cheng,Y.,

Zhang,L., Xu,T., Zheng,H., Pu,S., Wang,B., Gu,W., Zhang,X.L.,

Zhu,G.F., Wang,S., Zhao,G.P. and Chen,H.

TITLE Genome Biology of Actinobacillus pleuropneumoniae JL03, an Isolate

of Serotype 3 Prevalent in China

JOURNAL PLoS ONE 3 (1), E1450 (2008)

PUBMED 18197260

REMARK Publication Status: Online-Only

REFERENCE 2 (bases 1 to 15691)

AUTHORS Xu,Z., Zhou,Y., Li,L., Zhou,R., Wan,Y., Zhang,S., Wang,K., Li,W.,

Li,L., Jin,H., Kang,M., Dalai,B., Li,T., Liu,L., Xiao,S., Wang,S.,

Zhao,G.-P. and Chen,H.

TITLE Direct Submission

JOURNAL Submitted (01-MAY-2007) College of Veterinary Medicine, Laboratory

of Animal Virology, Huazhong Agricultural University, Wuhan, Hubei

430070, China

FEATURES Location/Qualifiers

source 1..15691

/organism="Actinobacillus pleuropneumoniae serovar 3 str.

JL03"

/mol_type="genomic DNA"

/strain="JL03"

/serovar="3"

/note="K locus: KL03"

/db_xref="taxon:434271"

CDS 1..1458

/gene="modF"

/locus_tag="APJL_1619"

/codon_start=1

/transl_table=11

/product="molybdate transport system ATP-binding protein"

/protein_id="ABY70171.1"

/translation="MPNINIQNALFSLAQHNKLSIESLEINTHDFWVIVGGNGSGKTA

FAQALHNSLSLYSGEYQNSFQHIALLSFEQQQKIIEQIFKHRNNDMISPDDFGLTARQ

IILNGSEKTQLCEEYAAKLRIQPLLERPFIQLSTGESRKVLFCQMLVSEPDLLILDEP

FEGLDQASVAYWQDVMAQLGKQMAVVLISNRFNDIPDCATHIALLDNLQLILQGERQE

IEQQAVYSQLKFAEQNVNAPLPESATPLIQLPPNTNPFELKNVMIRYGEKTIIDDLTW

TVAPKQHWWIKGQNGAGKSTLLSIIAGDHPQSYANYVHLFGRQRGSGETIWDIKKNIG

YVSSQLHMDYRVNCSALDVILSGFFDSIGVYQQVPSALQLKAMEWLERLHLANLAKKP

FRSLSWGQQRLLLITRAMVKHPPILILDEPLQGLDGVNRKLVKQFIEQLVTNSQTQLL

FVSHQDADAPNCITHLFEFVPQTNGGYRYVQTALN"

CDS complement(1657..2307)

/gene="cpxA"

/locus_tag="APJL_1618"

/codon_start=1

/transl_table=11

/product="capsule polysaccharide export transport system

ATP-binding protein"

/protein_id="ABY70170.1"

/translation="MISVKNVSKDYYTRSGKKTVLQDINFELKKGEKIGILGRNGAGK

STLIRLLSGVEPPTSGTIERNMSISWPLAFSGAFQGSLTGMDNLRFICRIYNADIEYV

KAFTEEFSELGDYLYEPVKKYSSGMKARLAFALSLSVEFDCYLIDEVIAVGDSRFAAK

CKHELFEKRKDRSIILVSHSPSAMKSYCDNAMVLDKGIMYKFENMDEAYKFYNSTL"

CDS complement(2304..3029)

/gene="cpxB"

/locus_tag="APJL_1617"

/codon_start=1

/transl_table=11

/product="capsule polysaccharide export transport system

permease protein"

/protein_id="ABY70169.1"

/translation="MREIITRYGRKNLGFLWLFVEPLLLTLFIVLMWKFIRADRVSDL

NIIAFVITGYPMAMMWRNASNRTIGAISGNLSLLYHRNVRVLDTLLARVILEVAGATI

AQIIIMALVILLGWIEMPKDTFYMVMAWVLMAFFALGLGLIICSIAQKFEAFGKIWGT

LSFVLLPLSGAFFFVHALPSQAQQYATLIPMIHGTEMFRHGYFGDSVITYESISYLVI

CDVAMLLFGLIMVKNFSKGIEPQ"

CDS complement(3101..4258)

/gene="cpxC"

/locus_tag="APJL_1616"

/codon_start=1

/transl_table=11

/product="capsule polysaccharide export transport system

permease protein"
/protein_id="ABY70168.1"
/translation="METPIATSPAEKLQKPVKQKKSRFKKLNPLFWITVAIPTVLSAF
YFGSVASDIYISESSFVVRSPKNQTALTGVGALLQGSGFSRSQDDTYTVQEYMRSRTA
LEQLMQGLPVREYYENQGDIIARFNGFGLNNSKEAFYKYFRDRLSVDFDSVSGIASLR
IRAFNAEEGQQINQKLLAEGETLINRLNERARKDTISFAEQAVTEAENNVNETANALS
KYRIKNKIFDLPAQSGVQLSLISSLKSELIRVETQLAQLQSITPDNPQVDALLMRQKS
LRKEIDEQSKQLSSNSNSSIAIQTADYQRLVLANELAQQQLTAALTSLQNTKNEADRQ
QLYLEVISQPSKPDWAEEPYRLYNILATFFIGLMLYGVLSLLIASVREHKN"

CDS complement(4284..5471)
/gene="cpxD"
/locus_tag="APJL_1615"
/codon_start=1
/transl_table=11
/product="HexD, capsule biosynthetic locus protein"
/protein_id="ABY70167.1"
/translation="MEIKKYNSIIGLALTTLFLSACSSLPTSGPSHSAILEANSQSSD
KPLPEVNVVELDNGLVQQLYQTQQSQQFSGFLGTVGSAGYAGAVNVGDVLEISIWEAP
PAVLFGGTFSSEGQGSGHLTQLPAQMVNQNGTVTVPFVGNIRVAGKTPEAIQSQIIGA
LQRKANHPQALVKIANNNSADVTVIRQGNSIRMPLTANNERVLDAVAAVGGTTENIED
VTVKLTRGSEVKTLAFETLISDPAQNIMLRAGDVVSLLNTPYSFTGLGAVGNNQQMKF
SSKGITLAEAIGKMGGLIDTRSDPRGVFVFRHVPFAQLSLEQQAQWQAKGYAIGMDVP
TVYRVNLLEPQSMFLLQRFPMQDKDIVYVSNAPLSEFQKFLRMIFSITSPVTSTTNAV
RAY"

CDS 5650..6792
/gene="cps3A"
/locus_tag="APJL_1614"
/codon_start=1
/transl_table=11
/product="Cps2A"
/protein_id="ABY70166.1"
/translation="MLMKIAFIAWNSFQVLHFKPLLQALPCALLIIEKRRRSVPICKD
ILRDINNNIAYIRHTDIYAKIDGNFDVLVAQTTFEQLYLFHRTKIALLQYGYAKEPYN
YGTWRAFADLNLVYGNYAYERISYFSPTKITGCPRYDLWYQPLFHQKAKENYARVLDT
SKKTIVYAPSWGELSSFKLYIEEITKLSLFYNVLVKLHHNTLLLANKHQNYEKLYPNL
HFFYEGEDLLSLISVADIVISDFSGAIFDAIFCKKPVILFSIPLVDQPKLDKFSLEIA
HRSALGYEVSSPERVAITVEKALTEQKLADKMLYQQLFMGNENATQQVIDALQQLVDG
KYSLSQQQLYVRQTEKLLNIEKIKQQKNKKQSFNKIRQISKKLIKK"

CDS 6846..7274

/gene="cps3B"

/locus_tag="APJL_1613"

/codon_start=1

/transl_table=11

/product="Glycerol-3-phosphate cytidylyltransferase"

/protein_id="ABY70165.1"

/translation="MKKVLTYGTFDLLHHGHIRLLERARSLGDHLTVAISTDQFNLGK

GKVCAYTYEERAHILKAIRYVDEVIPETKWEQKIDDVKNHEIDVFVMGDDWEGKFDFL

ADYCEVVYLPRTPDISTTQVKKMLAKKDLATGQKQIHEKE"

CDS 7276..8424

/gene="cps3C"

/locus_tag="APJL_1612"

/codon_start=1

/transl_table=11

/product="Cps7C"

/protein_id="ABY70164.1"

/translation="MLMFQILQKHLPTLQRVLREGYSQHSLLAYWYGLSLLTALEQAN

HPQVRKLADKMLNKGINIGHYFLAQSYFLCGEYTLAEQAVKKIPNFTKIPEVVFLYSD

ILIKCQRREEAWLLLEQCALLNKRKKVWIHLTNLVNTEADYRHLEQHIDKVRTTTPYL

KSDLLIHQRTNAALRAGLTETALALTELNPLPKQAKVKKKTTAYSDKLAAIALADLKK

VLDHKKIPFFLISGTLLGCIREGKLLGHDKDIDVGVWDEYSYEELANCLSTSGYFYVV

PTRTKHLVMLRHVNGIAIDVFIHYREPNDYWHAGVKIKWHNSPFNLVYTNFLGQQYLI

PENYDLYLTENYGDWRTPKTKFDSAFDTPNMEVINEVEMKVYVNKQRE"

CDS 8429..10270

/gene="cps3D"

/locus_tag="APJL_1611"

/codon_start=1

/transl_table=11

/product="teichoic acid biosynthesis protein"

/protein_id="ABY70163.1"

/translation="MKHNVKLSSVPSLLHGLYLYKHQQYQKAQEIFNKILEKQPQNAY

LNFRYGMALYKDKKWDEANHFIQKAVELAPEKEAWKKQLATTERYKHDSSKVKVAENK

KKLEANPNSPEYIWEYAISLIDSKQYWLAQFQLEKYIELKPNSEQAFHQLGIVSEKLA

NYEQAFIYFQKASQFAPLNRNYKYRMGYNLEKLGKLNEAQKCYSLVIDMSHPTDEVAQ

FGIGALHAKRGLWDMALSAYLQHQIQSNSQNPQLYYRIGIAYERLYQWTKSATTFEQA

IILSEIMNANWCFKCGQAYERAENFEKSAEFYQEAVKRSDNYNDYWWYRLALMLEKLG

KYEQSVVAFQNSRRRKLAYAVNPKDVIKHKEEEFLSYYTEYYETLELDEKLVLIESFF

GGNISCNPYAILSYMLENNYDYTYVVVIKDGTVIPDNLKFNRNIIFIKRGSDAYLRYL

CTAKYLINNVSFPYYFIRKEGQVYLNTWHGTPMKTLGKDIKSPFMDHANVSRNFLQAT

HIISPNRHTTDVILEQYDVKDLFSGKLAETGYPRIDLSFNLTDKRRNEIAEKLGFSNN
KPVVFYAPTWRGTSQSKDFDTSKLQYDLRKLKSNKYNLIFRGHHLVE"

CDS 10475..11845

/gene="cps3D'"

/locus_tag="APJL_1610"

/codon_start=1

/transl_table=11

/product="hypothetical protein"

/protein_id="ABY70162.1"

/translation="MSGTVCTTITDVKKTILEHISSGKSNVSEQDIQKYSYLDDGQAT
KRTVEFMLDKDDSCIYKYERRKSDVFFEGPFIPNGISRSFLNLMASIKDSGKNITLLI
NGSDIAQDQKRLEEFNNLPSNITVLSRVGRTPMTLEELWVRNKFEETYQIYSESFTNT
LLKVYKREVRRLLGNSSFDNAIHFEGYSLFWVLLFSQINAKKHIIYQHNDKYKEWKGR
FPYLEGVFNSYVFFDQIVSVSEKTMENNILNLSKEFNIPEIKFTFCNNPINIQQILSS
AEENIEMESEFTLFNGQKFINIGRMSHEKDQLKLIEAFYEAKKAHVNIRLFILGDGVL
KQDLINKIKDLSLEDSVYLLGQKKNPFPYLKQADVFILSSNHEGQPMVLLESLTLGTP
IIATDIVGNRSILGENYGTLVENNKDGLVQGINAYMEKGGRKDKFDPYEYQNDAMAKF
YSLLAN"

CDS 11864..12901

/gene="cps3E"

/locus_tag="APJL_1609"

/codon_start=1

/transl_table=11

/product="hypothetical protein"

/protein_id="ABY70161.1"

/translation="MLDFFKIEFNENTKYEELNIDFNAKEIYIDKSTIGKDEKEKLNY
NFLAVGRANKVAKKLISDLANNSYFLTAHANGISVFTKFIDGESFLPYFKNKGVKVYN
QVFYTLDKPIEYDINNRLIVIFSPVSDLPFNASIDRRMFFKDFPNIKENTPKNTYVLR
IADIGGVLGSAYLNTISDNKIEERVQELINKIQQDLLISELDTVFYGFGKGATGALYH
GIKMHSKTLAIDPLVSDEYYLKEFNDMHFTQGIFDKSKKDKFLQLFNEFKGRDLRNVT
IITSINSEQFKYISEIMNLLEPEVNLITLNNKNITESKDIINEGNNIILSLINSLLYG
FNFNVNLNTNY"

CDS 12910..14415

/gene="cps3F"

/locus_tag="APJL_1608"

/codon_start=1

/transl_table=11

/product="hypothetical protein"

/protein_id="ABY70160.1"

/translation="MAIIQLIDTLGLGRGGLTKAIYERVTYLSQIEKVTLVVTGLQFD
VQRVASLLKEQKQIPEELEVVGLFDFAFGSEKLEISPPIALDKSNLYKTIEATDSSNI
YRYFDEQGRFLGLENYSTDNTLNFLEVHSKEFPHICRARQIYDSNGHVRCVRYFDYTW
KPRFETVFESSGNPIYSCWLTETGNRYRIISFGKENKIAKICSDFYQLRADILYKIIE
QYPNSILISDEPTTIAFISRDFGFKKYIREGIGYIHTTHTYQSAGVDKLKPWFFDYKL
NSGVLSLILSTNEIQAVELRKVIAGANEQNVKSLPHSIEIKTDTQIKKFPTGRLLFLG
RLSDEKRVDLVIKGFSVALRKMPNLTLDIVGDGPLMSAHKQLVKDLNISKSVIFHGYS
LDVNSWFKQADCHFLVSKFEGFGLVLIEGLSNACPCIVSPCKYGPDQVIENNINGIRV
SATPEEIGKAIIKLYSNNTLATLSEGALKTSYKYSKLEWKQKWNDIISSTI"

CDS 14523..15077
/locus_tag="APJL_1607"
/codon_start=1
/transl_table=11
/product="hypothetical protein"
/protein_id="ABY70159.1"
/translation="MSKKITLLSLAVFIAGCSSAPQPEAFPGEFANADYVLSDKDAQR
WVVASHQAEQCIYPNLTRIQQQAFSKEDSYIHSQYVFFYPLEEIIGEQYVKIIQDDEK
SMGYAQYLFKKFRDNQEFEPLADKQCLVLREKAKNDLAVVKGQYKSGMVEETKSEAKN
ADGVATNQNKFFFDIIKWGSMLLL"

CDS complement(15149..15691)
/gene="ydeN"
/locus_tag="APJL_1606"
/codon_start=1
/transl_table=11
/product="predicted esterase of the alpha/beta hydrolase
fold"
/protein_id="ABY70158.1"
/translation="MKKVYVTHGYTANPTRNWFPWLKNELEKLGWECECLAMPNSDQP
NPQAWLEHHQNTLQLDENTLLIGHSLGCIALLNYLAVTQQKVKTAIFVSGFYEKLPTL
PELDSFADFYANQTACLPQKSYVISALNDVVVPHSFSDRLAQYLQADYIRLATGGHFV
DREGVTELPELLELIKQISN"

ORIGIN

1 atgccaaata tcaacatcca gaacgcctta ttttcccttg ctcaacacaa taaactctcg
61 attgaatcac tggaaatcaa tactcacgat ttctgggtga ttgtcggcgg taacggctcg
121 ggcaaaaccg ctttcgccca agcgctacat aattcacttt cgttatattc gggtgaatat
181 caaaatagtt tccagcatat cgctttactt tccttcgagc agcaacaaaa aatcatcgag
241 caaatcttta aacaccgtaa caacgatatg atttcaccgg atgatttcgg tttaaccgcc
301 cgtcaaatta tcttaaacgg tagcgaaaaa acgcaattat gcgaagaata tgcagctaaa
361 ttacgtattc agccgttatt agaacgcccg tttattcagc tatccactgg cgaaagtcgc

421 aaagtgctat tttgccaaat gttagttagc gaaccggatt tgctgatttt agatgagcct

481 tttgaggggt tagaccaagc ctcggtcgct tattggcaag acgtgatggc acaactcggc

541 aagcaaatgg cggtggtact gatttccaac cgttttaatg atattcccga ctgtgccaca

601 catattgctt tactggataa cttacaactg attttacaag gcgaacgtca agagattgaa

661 caacaagcgg tctattctca gctaaaattt gcagaacaga atgtgaatgc accgttgccg

721 gagagtgcca caccgctgat tcaactccca ccgaatacta atccgtttga actgaaaaac

781 gtaatgatcc gttacggcga aaaaacgatt attgatgatc taacttggac ggttgcccca

841 aaacaacatt ggtggattaa aggccagaac ggagcaggaa aatcgacctt actttctatt

901 attgccggcg atcatccgca atcttacgct aattatgtgc atttattcgg tcgtcagcgt

961 ggttcgggcg aaacgatttg ggatataaag aaaaatatcg gctatgtgag cagccaatta

1021 catatggatt atcgggtgaa ttgctctgcg ttagacgtga ttttatccgg ctttttgat

1081 tcaatcggcg tttatcaaca agtaccgagt gccttacagc taaaagcaat ggaatggctg

1141 gaacgcttgc atttagccaa tctggcgaaa aaaccgttcc gttcactttc gtgggggcaa

1201 caacggttat tattgattac tcgtgctatg gtaaaacacc cgccgattct gattttagac

1261 gaaccgctgc aaggtttgga cggtgtaaac cgcaaattgg ttaaacaatt tatcgaacag

1321 cttgtgacta atagtcaaac ccagttgcta tttgtttcgc accaagatgc ggacgcccca

1381 aattgtatca cccatttatt tgaatttgtt ccgcaaacta acggtggtta ccgttatgtg

1441 cagacggcgt taaattaggt ttttgacctt taaaggaaat ccccctcttt agtaaagagg

1501 gattaggga gatttgtcaa tagagagata tgaaattgaa tagaacttca tttttatat

1561 ttataaaagc gttaattagc atatttcttc gctaattcat tctgtcaaat ctcctcctgc

1621 ccctctttgc taaagagggg agatatgtgc gggactttaa agcgttgaat tatagaactt

1681 ataagcctcg tccatattct caaatttata cataatccct ttatctaata ccattgcatt

1741 atcgcaataa gacttcattg ctgacggact atgcgaaacc aaaataatcg aacgatcttt

1801 gcgcttttca aataattcat gtttacattt tgccgcaaag cgagagtcac ctaccgcaat

1861 tacctcatca attaagtagc aatcaaactc taccgaaagc gacaaagcaa aggcaagtcg

1921 ggctttcatg ccggaggaat atttcttaac cggctcatat aaataatcac ccaattcgga

1981 aaattcttcg gtaaaggctt taacgtattc aatatccgca ttatatatac ggcaaataaa

2041 gcgtaaatta tccataccgg ttaaactgcc ttggaacgcc ccgctgaaag cgagcggcca

2101 agatatcgac atattacgtt cgatagtacc tgatgttggc ggctcaacac cacttaacaa

2161 acggattagc gttgatttcc ctgcaccgtt acgccctaaa ataccgattt tctcgccttt

2221 tttcagctca aaattaatat cttgcaatac ggttttttta ccgcttcgag tatagtaatc

2281 tttactcaca ttttttacgc taatcattgc ggttcgattc ctttactgaa gtttttttacc

2341 ataatgagcc caaaaagtaa catggctaca tcacatatta cgagatagct tatactttca

2401 tatgtgataa cactgtcgcc aaaataaccg tgacgaaaca tttccgtgcc gtgaatcatc

2461 ggtattaagg ttgcatattg ttgagcttgg cttggtagcg catgcacaaa gaaaaatgcg

2521 cctgaaagag gtaaaagaac aaagcttaat gttccccaga ttttgccaaa tgcttcaaat

2581 ttttgtgcaa tagaacaaat aatcaagcct aatcctaatg caaaaaatgc cattaatacc

2641 cacgccataa ccatataaaa cgtatctttc ggcatttcta tccagcctaa taaaatgact

2701 aatgccataa taatgatttg ggcaatcgtt gcacccgcta cctcaagtat gacacgagcc

2761 agtaaggtat ctaatacgcg aacattacga tgataaagaa gactcaagtt accggaaatt

2821 gcaccgatag tgcggtttga tgcattacgc cacatcattg ccattggata accggtaatc

2881 acaaaagcaa taatatttaa atcggaaacg cgatccgctc ggataaattt ccacatcaaa

2941 acgataaata aagtgagtaa tagcggctca acaaacagcc ataaaaaacc caaatttttt

3001 cgtccgtaac gcgtaataat ttcccgcatg agtaatgcac cgattactct cccttgaatg

3061 gcgagagatt ggcggaaagt tgtttgatca ccgtattgca ttagtttttg tgctctctta

3121 cgcttgcaat taataaactt aatacaccat aaagcatcag accgataaag aatgtcgcta

3181 aaatattata taagcgataa ggctcttccg cccagtccgg tttgcttggc tgactgatta

3241 cttctaaata aagttgctgg cgatccgctt catttttcgt attttgtaat gaggttaatg

3301 ctgcggtcaa ttgttgctgt gccagctcgt ttgcaagtac taagcgttgg taatcggcag

3361 tttgaatagc aatagagcta ttactgttac tggaaagctg ttttgattgc tcatcgattt

3421 ccttacgtaa actttttttgg cgcataagca atgcatcaac ttgtgggttg tccggtgtaa

3481 tagattgcaa ttgagccaat tgtgtttcta cacgaatcaa ttcgcttttt aggctggaaa

3541 ttaatgaaag ttgtacgccg gattgtgccg gtaaatcaaa gattttattt ttgatacggt

3601 atttacttaa agcatttgcc gtttcgttta cattattttc cgcttctgta accgcttgtt

3661 ccgcaaatga aatggtatct tttcttgcac gttcgtttaa acggttaata agcgtttcac

3721 cttcggcaag taatttttga ttaatttgtt gcccctcttc cgcgttaaat gctcgaatac

3781 gtaagctggc gataccggaa acagagtcaa agtccacact taagcgatct cggaaatatt

3841 tataaaacgc ttctttactg ttatttaatc cgaatccatt aaagcgagcg ataatatcgc

3901 cctgattttc ataatattcg cgaacaggta atccttgcat tagttgctca agagcggtac

3961 gagaacgcat atattcctgt acggtataag tatcgtcttg cgaacgagaa aaacctgaac

4021 cttgcaataa ggcgcctacg cctgtcagag cggtctgatt tttaggcgat ctaacgacaa

4081 aacttgattc cgaaatataa atatcagaag cgaccgaacc gaaataaaac gctgatagca

4141 cggtagggat tgcgacagtt atccaaaata atggattgag ttttttaaag cggctttttt

4201 tctgtttaac cggtttctgt aatttttctg ctggactggt agcaataggt gtttccatct

4261 tttgtcctta tacattcaat atattaatag gcacgaacgg cattggtcgt actggtaacc

4321 ggcgaagtaa ttgagaaaat cattctcaag aatttttgga attcagacaa cggcgcattt

4381 gaaacataca caatatcttt atcttgcatt gggaaacgct gtaataaaaa catggattgc

4441 ggctcaagta agttcacacg ataaaccgtt ggtacatcca ttcctatagc gtagccttta

4501 gcttgccatt gtgcttgttg ttccaaactc aattgtgcaa aaggcacgtg acggaatacg

4561 aaaacccctc tcggatccga acgagtatca attaaaccgc ccatcttacc gatagcttcg

4621 gcaagcgtaa ttcctttact tgagaatttc atttgctggt tgttacccac agcgcctaaa

4681 ccggtaaaac tataaggcgt gtttagcaac gaaacaacat cgccagcacg taacataata

4741 ttttgtgccg gatcggaaat taacgtttcg aatgcgagtg tttttacttc agaaccacgg

4801 gttagcttga ccgtcacatc ttcaatgttt tccgttgttc cgcctactgc agcaaccgca

4861 tctaatacac gttcattatt agcggttaat ggcatacgaa tactattgcc ttgacgaata

4921 accgtaacat cagcagagtt attattcgca attttgacta atgcttgcgg atgattcgct

4981 ttgcgctgta gtgctccaat aatttgagac tgaatcgctt ccggtgtttt gcctgcgaca

5041 cgaatgttac ccacgaacgg cacggtaacc gtaccgtttt gattaaccat ttgtgccggt

5101 aattgcgtta aatgcccgct accttgtccc tcagaactaa aagtaccgcc aaacagcacc

5161 gccggcggag cttcccaaat tgatatttca agtacatcac ccacattgac cgcaccggca

5221 tagcccgcgc tgcctactgt gcctaaaaat ccggaaaatt gttggctttg ctgagtttga

5281 tacaactgtt gaactaaacc gttatccagt tccaccacat ttacttccgg taagggttta

5341 tccgaacttt gtgaattagc ctctaagatc gcactatggc tagggcctga agttgggagg

5401 cttgagcaag cagaaaggaa caatgttgtt aaagctaacc ctatgattga attatatttt

5461 ttaatttcca tcttgtaaaa aggctctatt gaaaaagtgt gtaaaatgaa tataaactat

5521 acataattat agataaaatg ctataattat atattatagt ctaatatctc gtttttaaa

5581 acgataacct aaaatattac ttttctatag aagaataggc attattttaa taaatataat

5641 taggtatgaa tgttgatgaa aatagcattt atcgcttgga atagttttca ggtgttacat

5701 ttcaagccct tattacaagc tttaccgtgt gcattattaa ttattgaaaa acggagacgt

5761 agtgtaccaa tctgtaagga tattttgcga gatataaaca ataatatcgc ttatatccgc

5821 catacggata tatatgcaaa aattgatggg aattttgatg ttctagttgc tcaaactact

5881 ttcgagcaac tttatttgtt tcaccgcacc aaaattgcat tgcttcaata cggatatgct

5941 aaggaaccgt ataactacgg cacttggaga gcatttgcag atctaaattt ggtttatggg

6001 aattatgcct atgaacgtat ttcctatttc tctccaacta aaataaccgg ttgtccacga

6061 tacgatttat ggtatcagcc tttatttcat caaaaagcga aagaaaatta tgcgagagta

6121 ttagatacga gtaagaaaac gattgtatat gcaccaagtt ggggagaatt atccagcttt

6181 aaattatata tagaagaaat tacgaaatta tctttatttt acaacgtgtt agtaaaatta

6241 caccataaca cgcttttatt agcaaacaag catcagaatt atgaaaaatt gtatccgaat

6301 ttacattttt tctatgaagg tgaagatctt ctctcactta tttcggtagc cgacattgtt

6361 atttccgatt ttagcggtgc gatctttgac gcaattttct gtaaaaaacc agtaatatta

6421 ttttctatcc cattagtaga tcaacccaaa ttagataaat ttagtttgga gatagcccat

6481 cgttcggcgt tgggatatga ggtttcttca cctgaacgag tagctataac ggtggagaaa

6541 gcactaacag agcaaaaatt agcagataaa atgctatatc aacagctttt tatgggtaat

6601 gaaaatgcaa cacaacaagt gatagatgct ctacaacagc tcgttgatgg aaaatattcc

6661 ttatcgcaac aacagttata tgtgcggcaa acagagaagt tattaaatat tgaaaaaata

6721 aagcaacaga aaaataaaaa acaatctttt aataagatac gacagatttc taaaaagtta

6781 attaaaaaat aaatttagac attttatttt ttatatcttt ttacttaata atacggagtt

6841 taggaatgaa aaaagtatta acctatggaa cctttgattt attacaccat gggcatattc

6901 gtttattaga aagagcaaga tcattaggcg atcaccttac tgttgctatt tctaccgatc

6961 aatttaactt aggaaaaggc aaagtatgcg cttatactta cgaagagaga gcgcatattt

7021 taaaagcaat ccgttatgtg gatgaagtaa ttcctgaaac taagtgggaa caaaaaatcg

7081 atgatgtaaa aaatcatgaa atcgatgtat ttgttatggg ggatgattgg gaaggtaaat

7141 tcgacttctt agcagattat tgcgaagtag tttatttacc tagaactcct gatatttcaa

7201 ccactcaagt aaaaaaaatg ctcgcgaaaa aagatctcgc aaccggacaa aaacaaattc

7261 acgaaaaaga gtaatttgtt aatgtttcaa atcctacaaa agcatttacc gaccttgcaa

7321 agagttctga gggagggtta ctcccagcat tctttgcttg cttattggta tgggcttagt

7381 ctacttactg ctcttgaaca ggcgaatcat cctcaagtaa gaaaacttgc cgataaaatg

7441 cttaataaag gtattaatat cggacactat tttttagcac agagttattt tctatgtgga

7501 gaatatacgc tagcggagca agcggttaaa aaaatcccaa attttactaa aatacctgaa

7561 gttgtttttt tatattcaga tattcttatc aaatgccagc gtagagaaga agcttggcta

7621 ctattagaac aatgcgcttt actcaataaa agaaaaaaag tgtggataca tctaacaaat

7681 ttagtaaata ctgaggcgga ttatcgacac ttagaacaac atattgacaa agtaagaaca

7741 actacacctt acttgaagtc tgatttgtta attcatcaaa gaacaaatgc ggcattaaga

7801 gcaggtttaa cagaaacggc attagctcta acagagctta acccttttgcc aaagcaagca

7861 aaagttaaga aaaaaacaac cgcttatagt gataaattag cggcaattgc gctagcggat

7921 ctaaagaaag tattagatca caaaaaaata cctttctttc tgattagcgg cacgttgcta

7981 ggttgtattc gagaaggaaa attattaggg catgataaag atattgatgt cggcgtttgg

8041 gatgagtact cctacgaaga attagcaaac tgtttatcta catcgggata cttttacgta

8101 gtaccaactc gaacaaaaca tttagtcatg ttaagacatg ttaatggtat tgcaattgat

8161 gtgtttattc attatcgcga acctaatgac tattggcacg ctggtgtcaa aataaaatgg

8221 cataattcac catttaattt ggtatataca aatttccttg ggcaacaata tttaatacct

8281 gaaaattatg atttatacct aacggaaaat tatggtgact ggcgtacgcc aaaaactaaa

8341 tttgatagcg cctttgatac gccaaatatg gaagttatca atgaggtgga aatgaaggtt

8401 tatgttaata aacaacgaga atgaaaaaat gaaacataat gtgaaactat catctgtgcc

8461 aagcttacta cacggcctat atttatataa gcaccaacaa tatcaaaaag cacaggaaat

8521 ttttaataaa atcttagaga aacaacctca aaatgcatat ctaaatttta gatatggcat

8581 ggctttatat aaagataaga aatgggatga agcaaatcac tttattcaaa aagctgttga

8641 gcttgctcct gaaaaagaag catggaaaaa gcaactagca acaactgagc gatataagca

8701 cgattcatca aaagttaaag ttgccgaaaa taagaagaaa ttagaagcaa atccgaatag

8761 cccagaatat atttgggagt acgctatatc tttaattgat agtaagcaat attggttagc

8821 acaattccaa ctagaaaaat atattgagct aaaaccaaat tctgaacagg catttcatca

8881 attaggtatt gtatctgaaa aattggcaaa ttatgaacaa gcattcatct atttccaaaa

8941 agcaagtcaa tttgcccctc taaaccgtaa ctataaatat cgtatggggt ataacctaga

9001 aaaactcgga aaactaaacg aggctcaaaa atgctactcg ctcgtcatag atatgagtca

9061 tcctacagat gaagtagcac aatttggtat tggagctttg catgctaaac gtggcttgtg

9121 ggatatggcg ctatcagcat atttacaaca ccaaatacaa tctaactctc agaatcccca

9181 gctatattat cgcattggca tagcctatga gcgcttatat cagtggacaa aatcggctac

9241 cacatttgaa caagcaatta ttttatctga aataatgaat gccaattggt gctttaagtg

9301 cggacaagcc tatgaaagag ctgaaaattt tgagaaatca gcagagtttt accaagaagc

9361 tgttaagaga tcggataatt acaatgatta ttggtggtat cgcttagctt taatgttaga

9421 gaaactagga aaatatgagc aatctgtggt tgctttccaa aactctagac gacgtaaact

9481 tgcttatgca gttaatccga aagatgttat taaacataaa gaagaagaat ttctttcata

9541 ctatactgaa tattatgaaa ccttagaatt agatgagaaa ttagtattaa ttgaaagttt

9601 ctttggtggg aatattagtt gtaatccgta tgcaattcta tcttatatgt tagaaaataa

9661 ttatgactat acttatgtag ttgtaattaa agatggaaca gttattcctg acaatctcaa

9721 atttaataga aatattatct ttattaaacg aggttcagac gcatatttac gttatttatg

9781 cacagcgaaa taccttatta ataatgttag cttcccttat tattttatta gaaaagaagg

9841 tcaggtttat ttaaatactt ggcatggtac gccaatgaag acattaggga aagatattaa

9901 aagtccattt atggaccacg caaatgtcag ccgaaatttt ttacaagcaa cacatattat

9961 ttcgcctaat cgtcatacaa ctgatgttat tttagagcaa tatgatgtta aggacttatt

10021 tagtgggaaa ttagctgaaa caggttatcc aagaattgat ttatcattca atttaacaga

10081 taaaagacgt aacgaaatcg cagagaagtt aggattctca aataataaac ctgttgtatt

10141 ctatgcacca acttggagag ggacatccca atcaaaagat tttgacacct ctaaattaca

10201 gtacgactta agaaaactta aatcaaataa atataatctg atttttagag gacatcactt

10261 agtagaataa ttattagaaa ctattaattt agatgtaact gttgccccaa aagatataga

10321 cagtaatgaa ttacttggtt tctgcgatct attaattact gactattcaa gtattattta

10381 tgatttctta gcattaagta aaccagcaat tagctatatt tatgactatg aagaatatga

10441 tgctgaacga gggttatatt taaaacctac ggaaatgtcg gggactgttt gtacaacaat

10501 tacagatgtt aaaaagacga tcttagaaca tatttcttca ggtaaaagta acgtctctga

10561 acaagatatt caaaagtatt cttatttaga tgatggtcaa gctactaaac gtactgttga

10621 atttatgtta gataaagatg attcttgtat ttataaatat gaacgcagaa aatcagatgt

10681 attctttgaa ggaccattta ttccaaatgg aatttcccgt tcattcttaa atttgatggc

10741 atcaattaaa gattcaggaa aaaatattac tttattaatt aatggttctg atatagcaca

10801 agatcaaaaa cgtttagaag aatttaataa cttaccatca aatattactg ttctttccag

10861 agtaggaaga acacctatga ctttagagga attatgggta agaaataaat ttgaagaaac

10921 atatcaaata tattctgaat catttactaa tactttatta aaagtatata aacgagaggt

10981 tcgtagatta ttaggtaatt cttcatttga taatgccatt catttcgaag gatattcttt

11041 attctgggta ttattatttt ctcaaattaa tgcaaaaaaa catattattt atcaacataa

11101 cgataaatat aaagaatgga aagggcgttt cccatattta gagggggtat ttaacagtta

11161 tgtatttttt gatcagattg tttctgtatc agaaaaaaca atggagaata atatattaaa

11221 tctttcaaaa gaatttaata tacctgaaat aaaatttact ttctgcaata accctatcaa

11281 cattcaacaa atcctctcta gtgcagagga gaatattgaa atggaaagtg agtttacttt

11341 attcaatggc cagaagttta ttaatattgg cagaatgtcg cacgagaaag atcaattaaa

11401 attaattgaa gcgttctatg aagcgaaaaa agcacacgtt aatatcagat tatttattct

11461 tggcgatggc gtattaaaac aagatttaat taataagatt aaagatctct cactagaaga

11521 tagtgtatat cttcttggac agaagaaaaa tccattccct tatttaaaac aggcggatgt

11581 atttattcta tcttctaacc acgagggaca accaatggtt ctactagaat ccctcacact

11641 aggaacacct attattgcta ctgatattgt aggtaaccgc agtattttag gtgagaatta

11701 tggaacgtta gtagaaaaca acaaagacgg attagttcaa ggtattaatg cttatatgga

11761 gaaaggtggt cgtaaagata aatttgatcc atatgaatat caaaatgatg ccatggctaa

11821 gttttactca ctactagcaa attaaaaaga taaggaatga ataatgctag atttttttaa

11881 aattgaattt aacgaaaata ctaaatatga agaattaaat atagatttta atgcaaagga

11941 aatttatatt gacaaatcaa ctattggtaa ggatgaaaag gaaaaattaa attataattt

12001 tttagctgtt ggtagagcaa ataaagttgc caagaaatta atttctgatt tagctaataa

12061 ttcttatttt ttgacggccc atgctaatgg tatatctgtt tttactaagt ttattgatgg

12121 cgaatcattt cttccatatt ttaagaataa aggcgtaaag gtatacaatc aggtttttta

12181 tacactagat aagcctattg aatatgatat aaataatcga cttatcgtta tttttcacc

12241 tgtttcagat ttacctttta atgcatctat agatagaaga atgtttttta aagactttcc

12301 taatataaaa gagaatactc ctaaaaacac ttatgtttta cgaatagcag atatcggtgg

12361 agttcttggt agtgcatatc taaatactat ttcggataat aaaatagagg aaagagtaca

12421 agaattaatc aataaaatac aacaagattt attaatctct gaattggaca ctgtatttta

12481 cgggtttggt aagggagcga ccggagctct ttatcatgga ataaaaatgc attctaaaac

12541 attagctata gaccctttag tatctgatga gtattatctc aaagagttta atgatatgca

12601 ttttactcaa ggtatttttg ataaaagtaa aaaggataaa tttttacaac tatttaatga

12661 attcaaaggt cgagatttaa gaaatgtaac aattattaca tctattaact ctgaacagtt

12721 taaatacatt agtgagataa tgaatttatt agagcctgaa gttaatctta taactttaaa

12781 taacaaaaac ataaccgaat cgaaagatat aataaatgaa ggaaataata taatattatc

12841 attaataaac tctttattat atggatttaa ctttaacgtc aacttaaata ctaattatta

12901 gggaaaaata tggccattat tcaacttatt gataccttag gtcttggtcg aggaggatta

12961 accaaagcga tatatgagag agttacatat ctttcacaaa tagaaaaagt aactttagtt

13021 gttacaggat tacaatttga tgtacaaaga gtcgcaagcc ttctaaagga gcagaaacag

13081 attcctgaag aactcgaagt tgtaggatta ttcgattttg cttttggttc tgagaaatta

13141 gaaatttcgc caccaattgc actagataaa tcaaatctct ataagacgat tgaagctaca

13201 gattcatcca atatttatcg gtattttgat gaacaaggtc gcttccttgg tcttgaaaat

13261 tacagcacag acaatacact aaattttctt gaagttcatt ctaaagaatt tcctcatatt

13321 tgtagagccc gccaaattta cgatagtaac ggccatgtaa gatgtgtgag atattttgat

13381 tatacatgga aacctagatt cgaaacagtt tttgaatcta gtggaaatcc tatctatagt

13441 tgttggttaa ctgaaacggg gaatcgttat agaattattt cctttggtaa agaaaataaa

13501 atagccaaga tttgttctga tttctatcaa ttaagagctg atattttata taaaattatt

13561 gagcaatacc ctaattcaat attaatatct gatgaaccaa caactatagc attcatcagt

13621 agagacttcg gctttaagaa atatattaga gaaggtatcg gttatatcca tacaactcac

13681 acatatcaat cggcaggagt agataaactt aagccttggt tctttgatta taaattaaat

13741 tcaggagtat tatcattaat tctatcgaca aacgaaattc aagctgtaga attaagaaag

13801 gtgattgctg gtgcgaatga gcaaaatgta aaatctctac cgcattcaat agaaattaaa

13861 actgatactc aaatcaaaaa atttcctacg gggcgcttat tatttctagg tagattaagt

13921 gatgaaaaac gagtagacct tgttattaaa gggtttagtg ttgctttgag aaaaatgcct

13981 aacctgacac tagatattgt aggggatggc cctttaatgt ctgcacataa acaattagta

14041 aaagatctta atatttctaa gtctgttata tttcatggat attctttaga tgtaaattca

14101 tggtttaagc aagccgattg ccatttctta gtgtcaaagt ttgaaggctt tggcttagtt

14161 cttattgaag gattatctaa tgcttgccct tgtatcgtat ctccatgtaa atatggacct

14221 gatcaagtaa ttgaaaataa cataaatggg attagagtat ctgcaactcc agaagaaata

14281 ggaaaggcaa tcataaaact atatagtaat aatacattag ctacactttc cgaaggtgca

14341 ttgaaaactt cctataagta ttctaaatta gagtggaaac agaaatggaa tgatattatt

14401 tcttctacta tataaatatc ttttatttag ggcttagtaa ttgtgcaaat aagcagtcaa

14461 tttttttaat ttgaaaaatt tagacagctt ctgttattaa ttcttttata taaaattata

14521 tcatgtccaa aaaaatcact ttattgagtc ttgccgtatt catcgcaggt tgttcctctg

14581 ctccgcagcc ggaagctttc ccgggggagt ttgcgaatgc ggattatgtg ttatcggata

14641 aagatgccca gcgttgggtg gtggcgagcc atcaggcgga gcagtgtatt tatccgaatt

14701 taacgcgtat tcagcagcaa gcgtttagca aggaagattc atatattcat tcgcaatacg

14761 tatttttcta tccgctggaa gaaattatcg gcgagcagta tgtaaagatt atccaagacg

14821 atgaaaaatc tatgggatat gcgcaatact tgtttaagaa attcagagat aatcaggaat

14881 tcgagccgtt agcggataag caatgtcttg tgttgcgaga aaaagcgaag aacgatttag

14941 cggtcgtaaa agggcagtat aagagcggaa tggttgaaga aacgaagtcc gaagctaaaa

15001 atgcggacgg cgtggcgacc aatcaaaata aattcttctt tgatattatc aaatggggtt

15061 cgatgctatt actgtaattt gcggttagtg tgatgttaaa gagtataaaa acgagctgtc

15121 ggtaaatcgt tatcggcagc tctttttatt agtttgatat ttgtttgatt aactccaata

15181 actccggtaa ttccgttact ccctctcgat cgacaaaatg cccgcccgtt gccaagcgaa

15241 tataatccgc ttgtaagtat tgcgctaatc gatcgctgaa cgaatgggga acgacaacat

15301 catttaatgc agatatgacg taagactttt gcggtaaaca agcggtctga tttgcataaa

15361 aatctgcaaa gctatctaat tccggcaaag ttggtaattt ctcataaaag ccggaaacaa

15421 aaattgccgt ttttactttt tgctgcgtta ccgccagata attcagtaac gcaatgcagc

15481 ccaaactatg tccgatgagt aaggtatttt catctaattg aagtgtattt tggtgatgtt

15541 ccagccatgc ttgcggattc ggctgatcgg aattcggcat cgctaaacat tcacattccc

15601 atcctaattt ttccaattcg tttttaagcc acggaaacca atttcttgtc gggttcgccg

15661 tataaccgtg cgttacatat actttttca t

//

LOCUS **LS483358.1** 12770 bp DNA linear BCT 20-APR-2021

DEFINITION Actinobacillus pleuropneumoniae strain NCTC11384
capsular polysaccharide gene locus, complete sequence.

ACCESSION LS483358 REGION: complement(1809743..1822512)

VERSION LS483358.1

KEYWORDS .

SOURCE Actinobacillus pleuropneumoniae

ORGANISM Actinobacillus pleuropneumoniae
Bacteria; Proteobacteria; Gammaproteobacteria; Pasteurellales;
Pasteurellaceae; Actinobacillus.

REFERENCE 1

AUTHORS Doyle,S.

CONSRTM Pathogen Informatics

TITLE Direct Submission

JOURNAL Submitted (08-JUN-2018) WTSI, Pathogen Informatics, Wellcome Trust
Sanger Institute, CB10 1SA, United Kingdom

FEATURES Location/Qualifiers

source 1..12770

/organism="Actinobacillus pleuropneumoniae"

/mol_type="genomic DNA"

/strain="NCTC11384"

/note="K locus: KL04"

/serovar="not available: to be reported later"

/db_xref="taxon:715"

/chromosome="1"

CDS 1..1458

/gene="modF"

/locus_tag="NCTC11384_01732"

/EC_number="3.6.3.-"

/inference="ab initio prediction:Prodigal:2.60"

/inference="similar to AA sequence:RefSeq:YP_001054275.1"

/codon_start=1

/transl_table=11

/product="molybdenum transport ATP-binding protein ModF"

/protein_id="SQF65376.1"

/translation="MSNINIQNALFSLAQHNKLSIESLEINTHDFWVIVGGNGSGKTA

FAQALHNSLSLYSGEYQNSFQHIALLSFEQQQKIIEQIFKHRNNDMVLPDDFGLTARQ

IILNGSERTQLCEEYAAKLRIQPLLDRPFIQLSTGESRKVLFCQMLVSEPDLLILDEP

FEGLDQASVTYWQEVMAQLGKQMAVVLISNRFNDIPDCATHIALLDNLQLILQGERQE

IEQQAVYSQLKFAEQNVNAPLPESAAPLIQLPPNTNPFELKNVMIRYGEKNIIDDLTW

TVAPKQHWWIKGPNGAGKSTLLSIITGDHPQSYANYVHLFGRQRGSGETIWDIKKNIG

YVSSQLHMDYRVNCSALDVILSGFFDSIGVYQQVPSALQLKAMEWLERLHLANLAKKP

FRSLSWGQQRLLLITRAMVKHPPILILDEPLQGLDGVNRKLVKQFIEQLVTNSQTQLL

FVSHQDADAPNCITHLFEFVPQTNGGYRYVQTALN"

CDS complement(1657..2307)

/gene="cpxA"

/locus_tag="NCTC11384_01731"

/inference="ab initio prediction:Prodigal:2.60"

/inference="similar to AA sequence:RefSeq:YP_001652614.1"

/codon_start=1

/transl_table=11

/product="capsule polysaccharide export transport system

ATP-binding protein"

/protein_id="SQF65375.1"

/translation="MISVKDVSKRYHTNSGWKTVLQNINFDLHKGEKIGILGRNGAGK

STLIRLMSGVEPPTTGKIERHMSISWPLAFSGAFQGSLTGMDNLRFICRIYNADFEYV
KAFTEEFSELGDYLYEPVKKYSSGMKARLAFALSLSVEFDCYLIDEVIAVGDSRFAAK
CKYELFEKRKDRSIILVSHSPSAMKEYCDNAMVLDKGIMHKFENMDDAYKFYNSTP"

CDS complement(2304..3101)
/gene="cpxB"
/locus_tag="NCTC11384_01730"
/inference="ab initio prediction:Prodigal:2.60"
/inference="similar to AA sequence:RefSeq:YP_006818391.1"
/codon_start=1
/transl_table=11
/product="capsule polysaccharide export inner-membrane
protein"
/protein_id="SQF65374.1"
/translation="MQYGDQTTFRQSLAIQGRVIYALLMREIITRYGRKNLGFLWLFI
EPLLLTFLIVLMWKFFRADKVSTLNIVAFTITGYPMAMMWRNVSNRAIGSISANLSLL
YHRNVRVLDTIFARMLLEVAGATVAQIIITAVLVFIGWIDPPKDVFYMVLAWTLMAFF
AFGLGLIICSLAQKIEVFGKIWNTLSFVLLPLSGAFFFVHSLPSQIREIAQWVPIISG
TEMFRHGYFGDLVPTYENIGFLVVCDLAMLLLGLILVRNFSKGIEPQ"

CDS complement(3101..4240)
/gene="cpxC"
/locus_tag="NCTC11384_01729"
/inference="ab initio prediction:Prodigal:2.60"
/inference="similar to AA sequence:RefSeq:YP_006818390.1"
/codon_start=1
/transl_table=11
/product="polysaccharide transport protein"
/protein_id="SQF65373.1"
/translation="MVAITAEKPNKPKKKSLLRKLNPLLWLTVIFPTLFSAIYFGMFA
SDIYVSESSFVVRSPRNQSSLSGVGALLQGTGFTRSQDDTYSVQEYMRSRTALEQLQT
ELPVRDFYATKGDILSRFNGFGLNDTQEAFFRYFKDRLSIDVDSVSGIATLRVQAFEA
SEGQQLNEKLLRLGEDLINRLNARGRKDTLEFAAQAVQEAEKNVNETAEALSKYRIKN
KIFDLPAQSGVQLSLISSLKSELIRVETQLAQLQSITPDNPQVDALLMRQKSLRKEID
EQSKQLSGNTGSSVANQSADYQRLVLANELAQQQLTAAMASLHNTRGEADRQQLYLEV
ISQPSKPDWALAPSRLYNIIATFIIGLMLYGIFGLLITSVREHKN"

CDS complement(4290..5474)
/gene="cpxD"
/locus_tag="NCTC11384_01728"
/inference="ab initio prediction:Prodigal:2.60"
/inference="similar to AA sequence:RefSeq:YP_001652611.1"

/codon_start=1

/transl_table=11

/product="HexD, capsule biosynthetic locus protein"

/protein_id="SQF65372.1"

/translation="MKLIKLRLLLSLGLVASLAACSSLPTSGPSHSAILEANSQNSDK

PLPEVNLVELDNGLVQQLYQTQQSQQFSGFLGTAGGAGYAGAVNVGDVLEISIWEAPP

AVLFGGTFSSEGQGSGHLTQLPAQMVNQNGTVTVPFVGNIRVAGKTPEAIQSQIIGAL

QRKANHPQALVKIANNNSADVTVIRQGNSIRMPLTANNERVLDAVAAVGGTTENIEDV

TVKLTRGSQVKTLAFETLISDPAQNIMLRAGDVVSLLNTPYSFTGLGAVGNNQQMKFS

SKGITLAEAIGKMGGLIDTRSDPRGVFVFRHVPFAQLSLDQQAQWQAKGYAIGMDVPT

VYRVNLLEPQSMFLLQRFPMQDKDIVYVSNAPLSEFQKFLRMIFSVTSPITSTTNSIR

SY"

CDS 5697..6809

/gene="cps4A"

/locus_tag="NCTC11384_01727"

/EC_number="2.7.-.-"

/inference="ab initio prediction:Prodigal:2.60"

/inference="similar to AA sequence:RefSeq:YP_006818388.1"

/codon_start=1

/transl_table=11

/product="capsular polysaccharide phosphotransferase LcbA"

/protein_id="SQF65371.1"

/translation="MNKMNRKFSKLLKNPHIFFRDFLNKKYPIKNTELPFSESEEANL

IEANQKLDKIIQKNTLQQANIDVVFTWVDGSDPSWQAKYSQYAPNYQAKSALYATDIA

RFEDHNELYYSVHAVLKYIPWVRHIFIITDNQKPKWLDETKQEKITLIDHQDIIDEEY

LPTFNSHVIEAFLHKIPNLSENFIYFNDDVFIARELQAEHFFQANGIASIFMSEKSLT

QMRNRGTITPTLSASEYSIRLLNKYYNTNIDSPLVHTYIPLKKSMYELAWRRYEKEIL

GFLPNKLRTNNDLNFANFLIPWLMYFEGKAMPKIDICYYFNIRSPNALTQYKKLLNKK

NIGEQPNSFCANDFNSQKSINNYQNQLFSFLSSYYS"

CDS 6820..9414

/gene="cps4B"

/locus_tag="NCTC11384_01726"

/EC_number="2.7.8.12"

/inference="ab initio prediction:Prodigal:2.60"

/inference="similar to AA sequence:RefSeq:YP_006818479.1"

/codon_start=1

/transl_table=11

/product="Putative lipooligosaccharide biosynthesis

protein"

/protein_id="SQF65370.1"

/translation="MNKVKRKFRKLLRDPKLFFSDFWFKHSIKLEKFLSIKHYGNNEF

TIVSAIYNVEKYLDQYFNSIFKQTLLFKNNINIICVDDGSTDKSAEIIEKYRKKYPQN

IKYIYKENGGQASARNLGIKYVTTKWVTFIDPDDFISRNYFELVDDFIEKNTNLSLVS

CPFIFYFEDKNIYKDSHPLNFRFKNGEYISPIKSLDKHIQLSVNSAFFRTAVIKKNNI

QFGEIRPNFEDAKFVGDYLLSVNQENLIGFMKDVSYFYRKRSDQSSTLDTAWKNPLLY

SQVLENGCLALCERSATQKGFVPKYIQRAVLYHLSWYFKYLINNKDKLNFLTDKEQTY

FLELLHKIFRYIDVQTIMDFELAGTWFFQRVAWLGYFKKVEPDFQIVYIDSIDRENKQ

ILFYYYTFSDMPNEKFYLGKNELEPIDSKLRSFDFMGNNFVYERRIWLPYSDKDKKLL

FRFKINGQEPRISLAGKQHKSGLPIHTFLRDMPVKKYTHIEDFWIIMDRDVQADDNGE

HFYRYMMNNHPEQKIYFAINRNSNDWGRLKREGFNLIDFKSNEFKTLVSQCSRLISSH

IDEYIINPFKDHFEFTKKFIFLQHGVTHNDLSDWLNSKKILSCIITATPDEYNHISEN

KSRYKYSTKETILTGFPRHDALLRGNKTETRTILIMPTWRNSILGKNAKGNERSINSE

FMNTQYAKAWGAILSSPILEKLANQYDFEVIFAPHKNIEPYLDLFNIPKYIKQWKASE

GNIQKLFQNSKFMITDYSSVAFEMGYLNKTVLYYQFDKDSFFSGGHAFKRGYFSYEQH

GFGPVVYTEEEFFINLENILKNNGNPSEIYKSRIAQTFPFQDGKCCERVYFAIQNLTT

LYSYTEKA"

CDS 9414..10457

/gene="cps4C"

/locus_tag="NCTC11384_01725"

/inference="ab initio prediction:Prodigal:2.60"

/codon_start=1

/transl_table=11

/product="glycosyltransferase, SP_1767 family"

/protein_id="SQF65369.1"

/translation="MKSVILDVLKNTYSQNYYCKKIDVYKNVVFIDIKTQYFYFALEM

ANEKDIELIFRDDDSLQKANHYFNYPFQKGNKIYVCNFCTNNLFYMNEELSLNNMQTL

INRISHIISVFINIENTSLVRLSESNILLKNIKENSDLYLSNQVYLDMSNFMEDKLLS

IKDTIQYIIDNELSIARFGDGEIRCMVTRNGCSFQNHNWKLMNELREMCLEKNDKLLI

CYPSLMVDNAFWSPFWNKFWSKCKFYLRQNIIGDSFITRPEAFYFHSSLIVEKWKEVW

NNKNVCFITGENSRFNTKHYIFNNIKTENYIYSKPKNAYDDIDNIISKCTQLKDIDIF

LIALGPTGTAHTV"

CDS 11160..11273

/locus_tag="NCTC11384_01724"

/inference="ab initio prediction:Prodigal:2.60"

/codon_start=1

/transl_table=11

/product="Predicted small periplasmic lipoprotein"

/protein_id="SQF65368.1"

/translation="MKKLFLAALIASFGLAACGVKGPLYFPEQQPAQQQTK"

CDS 11400..12005

/gene="lysA_2"

/locus_tag="NCTC11384_01723"

/EC_number="4.1.1.20"

/inference="ab initio prediction:Prodigal:2.60"

/inference="similar to AA sequence:RefSeq:YP_001652547.1"

/codon_start=1

/transl_table=11

/product="diaminopimelate decarboxylase"

/protein_id="SQF65367.1"

/translation="MNHFNYKNQQLFAEDVSVSDIINQYGTPAYIYSRATLERRWHAF

DKAFGSHPHLICFAVKSNSNIALLNVMARLGSGFDIVSQGELERVLAAGGEPSKVVFS

GVAKSHSEIQRALEVGIRCFNIESIAELHRINEVAGQLGKIAPISLRVNPDVDAHTHP

YISTGLKENKFGVSVTNEGNKYWEKRYEKKHRRKTYCVKVR"

CDS complement(12228..12770)

/gene="ydeN"

/locus_tag="NCTC11384_01722"

/EC_number="3.-.-.-"

/inference="ab initio prediction:Prodigal:2.60"

/inference="similar to AA sequence:RefSeq:YP_001652602.1"

/codon_start=1

/transl_table=11

/product="alpha/beta hydrolase superfamily esterase"

/protein_id="SQF65366.1"

/translation="MRKVYITHGYTANPSRNWFPWLKNELEKLGWECECLAMPNSDQP

NPQAWLEHHRNTLQLDENTLLIGHSLGCIALLNYLAVTQQKVKTAIFVSGFYEQLPHL

PELDEFANFYTNQTACLPQKSYVISALNDVVVPHSFSDRLAQYLQADYIRLATGGHFV

DREGVTELPVLLELLKQILK"

ORIGIN

1 atgtcaaaca tcaacatcca gaatgcctta ttttcccttg ctcaacacaa taaactctcg

61 attgaatcac tggaaatcaa tactcacgat ttctgggtga ttgtcggcgg taacggctcg

121 ggcaaaaccg ctttcgccca agcgctacat aattcacttt cgttatattc gggtgaatat

181 caaaatagtt tccagcatat cgctttactt tccttcgagc agcaacaaaa aatcatcgag

241 caaatcttta aacaccgtaa caacgatatg gttttaccgg atgatttcgg tttaaccgcc

301 cgtcaaatta tcttaaacgg tagcgaaaga acgcaattat gcgaggaata tgcggctaaa

361 ttacgtatcc agccgttatt agatcgcccg tttattcagc tctccaccgg cgaaagccgc

421 aaagtgttat tttgccaaat gttagtcagc gaaccggatt tattgatttt agatgagcct

481 tttgaagggt tggatcaagc ctcggtcact tattggcagg aagtgatggc acaactcggt

541 aagcaaatgg cggtggtact gatttccaac cgttttaatg atattcccga ctgtgccaca

601 catattgctt tactggataa cttacaactg attttacaag gcgaacgcca agagattgaa

661 caacaagcgg tctattctca gctaaaattt gcagaacaga atgtgaatgc accgttgccg

721 gagagtgccg caccgctgat tcaactccca ccgaatacta atccgtttga actgaaaaac

781 gtgatgattc gttacggcga aaaaaacatt atcgatgatc taacttggac ggttgcccca

841 aaacaacatt ggtggattaa aggcccgaac ggagcaggaa aatcgacctt actttctatt

901 attaccggcg atcatccgca atcttatgct aactacgtgc atttattcgg tcgtcagcgt

961 ggctcgggcg aaaccatttg ggatatcaag aaaaatatcg gctatgtgag cagccaatta

1021 catatggatt atcgggtgaa ttgctctgcg ttagacgtga ttttatccgg cttttttgat

1081 tcaatcggcg tttatcaaca agtaccgagt gccttacagc tcaaagcaat ggaatggctg

1141 gaacgcttgc atttagccaa tctggcgaaa aaaccgttcc gttcactttc gtgggggcaa

1201 caacgtttat tattgattac tcgtgctatg gtaaaacatc cgccgattct gattttagac

1261 gaaccgctac aaggcttgga cggtgtaaac cgcaaattgg ttaaacagtt tatcgagcag

1321 ttggtcacta acagccaaac ccagttgcta tttgtttcgc accaagatgc ggacgcccca

1381 aattgtatca cccatttatt tgaatttgtt ccgcaaacta acggtggtta ccgttatgtg

1441 cagacggcgt taaattaggt tttgaccttt taaaggaaat ccccctcttt agtaaagagg

1501 gattagggga gatttgtcaa tagagagata tgaaattgaa tagaacttca ttttttatat

1561 ttataaaagc gttaattagc atatttcttc gctaattcat tctgtcaaat ctcctcctgc

1621 ccctctttgc taaagagggg agatatgtgc gggactttaa ggcgttgaat tatagaactt

1681 ataagcatcg tccatatttt caaatttatg cataatgcct ttatcaagca ccatcgcatt

1741 atcacaatat tccttcattg ctgacggact gtgcgacact aaaatgatcg aacgatcttt

1801 gcgtttctcg aataattcgt acttacattt tgccgcaaag cgagagtcac ctaccgcaat

1861 tacctcatca attaagtagc aatcaaactc aactgaaagc gataaagcaa acgcaagtcg

1921 ggctttcata ccggaagaat atttcttcac cggctcatat aaataatcgc ctaattcaga

1981 aaattcctcg gtaaacgctt taacatactc aaaatcggcg ttataaattc ggcaaataaa

2041 gcgtaaatta tccataccgg ttaaactgcc ttgaaatgca ccgctgaaag cgagcggcca

2101 agaaattgac atatggcgct caatcttacc ggttgttggc ggctcaacac cgctcattaa

2161 gcgaattaag gttgacttac ctgcaccgtt acgccccaga ataccgattt tttcgccttt

2221 atgcaggtca aaattgatat tctgtaacac ggttttccaa ccgctattag tatgatagcg

2281 cttgctcaca tcttttacac tgatcattgc ggttcaattc ctttactgaa gtttcgcacg

2341 agaatcagcc ccaataacag cattgccaaa tcgcatacta ctaaaaaacc gatattttca

2401 tacgttggga ctaaatcgcc aaaatagccg tgacgaaaca tttccgtacc actaattatc

2461 ggcacccatt gggcaatttc acgaatttgt gatggtagtg aatgtacgaa gaaaaatgca

2521 ccagacagcg gtaataatac aaaacttaac gtgttccaaa ttttgccgaa cacctcaatc

2581 ttttgtgcta acgaacaaat aatcagacct aacccaaagg caaaaaatgc cattaaagtc

2641 caagccagca ccatgtaaaa cacatctttc ggcggatcta tccagccgat aaaaactaaa

2701 accgccgtta taataatctg ggcaacagtc gcaccggcaa cttccaataa catacgagca

2761 aaaatcgtat ctaatacccg tacgttacgg tgatatagaa ggcttaagtt agcggaaatt

2821 gagccaattg ctcgatttga tacatttcgc cacatcattg ccatcggata gccggtaatc

2881 gtaaacgcta caatatttaa cgtagaaacc ttatccgctc ggaaaaactt ccacattaac

2941 acaatcaaaa acgttaacag caacggctca ataaacaacc ataaaaagcc taagttctta

3001 cgcccatagc gggtaataat ctcccgcatc aacagcgcat aaatcacacg cccctgaatg

3061 gctaacgatt gtcgaaacgt tgtttggtca ccatattgca ttagttttta tgctctctca

3121 cacttgtgat taataaaccg aaaattccat ataacattaa accgataata aaagtcgcaa

3181 taatgttata taaacggctt ggtgccaaag cccagtccgg tttacttggt tgactgataa

3241 cttctaaata aagttgctga cggtcggctt cgcctcgagt gttatgtaat gatgccattg

3301 ctgcggtcag ttgttgctgc gctaattcat tagctaacac taaacgctga taatccgccg

3361 attgattcgc aaccgagcta cccgtattac ccgaaagctg ttttgactgc tcatcaattt

3421 ctttacgtaa acttttttgg cgcatcagta gtgcatcaac ctgagggtta tccggtgtaa

3481 ttgactgcaa ctgcgccaat tgcgtttcca cacgaatcag ttcacttttc agactcgaaa

3541 ttaaagaaag ctgtacgccc gattgtgccg gcagatcaaa aattttattt ttgatacgat

3601 atttacttaa tgcttccgca gtttcattaa cattttttc cgcttcttgc accgcttgag

3661 cagcaaactc gagcgtatct ttacgacctc gagcatttaa acggttaatc aagtcttcac

3721 ccagtcttaa caatttctca tttaactgct gcccctcact cgcttcaaac gcttgtacac

3781 gtaaagtcgc aatccctgat acggaatcca catcaatgct taaacgatct ttaaaatagc

3841 ggaaaaacgc ttcttgcgta tcatttaaac cgaaaccgtt aaaacggctc agaatatcgc

3901 cttttgttgc atagaaatca cgaaccggta attcggtttg taactgttct aatgccgtac

3961 gagaacgcat atattcttgc actgaatacg tgtcgtcttg cgaacgagta aagcctgttc

4021 cttgtaacaa cgctcccacg cccgaaagcg aagactgatt acgtggcgaa cgcaccacaa

4081 aacttgattc ggaaacataa atatccgaag caaacatccc aaaataaatg gctgaaaata

4141 atgttggaaa aataaccgtt aaccaaagca atggatttaa ctttcttaat aagctttttt

4201 tcttaggttt attcggtttt tctgcggtaa tcgctaccat tatcctttcc tctaaataga

4261 tttatcgtta taaggtctat tgaccgcaat taatagctac gaatactgtt agtagtactc

4321 gtaatcgggg atgttactga gaaaatcatt ctcaagaatt tttggaattc agacaacggc

4381 gcatttgaaa catacacaat atctttatct tgcattggga aacgctgtaa taaaaacatg

4441 gattgcggct caagtaagtt cacacgataa accgttggta catccattcc tatagcgtag

4501 cctttagctt gccattgtgc ttgttgatct aaactcaatt gtgcaaaagg cacatggcga

4561 aacacaaata cccctctcgg atccgaacgg gtatcaatta aaccgcccat cttaccgata

4621 gcttcggcaa gcgtaatacc tttacttgag aatttcattt gttggttatt acccaccgca

4681 cctaaaccag taaagctata aggtgtattc aacagcgaca ccacatcgcc ggcacgtaac

4741 ataatatttt gtgccggatc agaaattagg gtttcaaacg ctaatgtttt gacttgcgaa

4801 ccacgagtta atttaaccgt cacatcttca atattttccg ttgtcccgcc taccgctgcc

4861 actgcatcta atacacgttc attattagcg gttaatggca tacgaatact attgccttga

4921 cgaataaccg taacatcagc agagttatta ttcgcaattt tgactaatgc ttgcggatga

4981 ttcgctttgc gctgtagtgc tccaataatt tgagactgaa tcgcttccgg cgttttaccc

5041 gctacacgaa tattaccgac aaacggcacg gtaaccgtac cgttttggtt aaccatttgc

5101 gccggtaatt gggttaaatg cccactaccc tgaccttcag aactgaaagt accgccaaac

5161 aacactgccg gcggcgcttc ccaaattgaa atttcaagaa catcccccac attgaccgca

5221 ccggcatatc cagcaccgcc agccgtgcct aaaaagccgg aaaattgctg actttgctga

5281 gtctgataca actgctgaac taagccatta tctaactcca ctaaattaac ttccggtaaa

5341 ggtttatctg aattctggga attagcctct aaaatcgcac tatggctagg acctgaagtc

5401 gggaggcttg agcaggcagc caaactagca accagcccca aagaaaggag taatctaagt

5461 ttgatgagtt tcatctaatt tctcttcaat atattaagga ataacaacta tataggtatg

5521 tcttaaaatc cacataaaga ttgattttaa taagttacct aatcaagaga aattaaatat

5581 aaaaaattta caaaaaagca ataatgcgta taaaaaaaca tcatttgcaa agaaagtaaa

5641 tagagaggag agttcaacag ataagcatta taatctaaaa tataattgat aatataatga

5701 acaaaatgaa tagaaaattt tctaagttac taaaaaatcc acatattttt tttagggatt

5761 ttctaaataa aaagtaccct ataaaaaata cggaacttcc cttctcagaa tctgaagaag

5821 ctaacttaat agaagcaaac caaaaattag ataagattat ccaaaagaat acgttgcaac

5881 aagctaatat tgatgtggta tttacttggg tagatggttc tgatccttca tggcaagcta

5941 aatattccca atatgcacca aattatcaag cgaaatccgc tctatatgca acggatatcg

6001 cccgatttga agatcataat gaattatatt attcagtaca tgctgtactt aaatatatac

6061 cttgggttag gcatatattt attataacag ataatcaaaa gccaaagtgg ttggatgaga

6121 cgaaacaaga aaaaattaca ctaatcgatc atcaagatat tatagatgaa gaatatcttc

6181 caacgtttaa ttcccatgtt attgaagcat ttttacataa aattcctaat ttaagcgaga

6241 attttatcta ttttaatgat gatgtcttta ttgcacgaga actacaagct gaacactttt

6301 tccaagcaaa tggtattgcc tctatattta tgtcggaaaa aagcctcact caaatgcgta

6361 acagaggaac tattacaccg actctttctg cttcggaata tagtattcgc ttactaaaca

6421 aatattacaa tacaaatatt gactcaccac tcgtacacac ttatatccca ttgaaaaaaa

6481 gtatgtatga attggcatgg cggcgttatg agaaagaaat tcttggattt ttacccaata

6541 aattaagaac aaataacgat ttaaattttg caaactttct tattccttgg ttaatgtatt

6601 tcgaagggaa agcaatgcct aaaatagata tttgttatta ttttaatatt agatctccaa

6661 atgcacttac acaatataaa aaacttttaa ataaaaaaaa cataggcgaa cagcctaatt

6721 cattttgcgc aaatgatttt aatagtcaaa aaagtattaa caactatcaa aatcaattgt

6781 tttctttttt aagctcctat tacagttaag gataatataa tgaataaagt aaaacgtaaa

6841 tttagaaaat tactacgaga tcctaagttg ttttttagtg acttttggtt taaacatagt

6901 ataaaactag aaaaattcct atcgataaaa cactatggca ataatgaatt cactattgta

6961 tctgctattt ataatgttga gaaatatcta gatcaatatt tcaatagtat attcaagcaa

7021 accttacttt tcaaaaataa tattaatatc atttgtgtag atgatggttc tactgacaaa

7081 tctgcggaaa tcatagaaaa atacagaaaa aaatatcctc aaaacattaa atatatctat

7141 aaagagaatg gagggcaagc ttctgctaga aatttaggca taaaatacgt tacgacaaaa

7201 tgggtcacat ttattgatcc tgatgacttt atttcaagaa attattttga attagtcgat

7261 gattttattg aaaaaaatac taatttatct ctagtaagct gtccatttat tttctatttc

7321 gaagataaaa atatatacaa agacagtcac cctttaaatt ttagatttaa aaatggtgaa

7381 tatatttcac ctataaaatc tttagataaa catattcaat tatcggtaaa tagtgcattt

7441 tttagaacag cagttatcaa gaaaaacaat attcaatttg gtgaaataag acccaatttt

7501 gaagatgcta aatttgtagg cgattattta ctttctgtaa accaagaaaa tttaattggt

7561 tttatgaaag atgttagtta tttttatcgc aaaagatctg atcaatcttc tactctagat

7621 acagcttgga aaaatccttt actttattcg caagttttag agaatggttg cttagcgtta

7681 tgtgagcgct ctgcgactca aaaaggtttt gtcccaaaat atattcaaag agcggtttta

7741 tatcatctat cttggtactt taaatacctt atcaataata aagataagtt aaacttttta

7801 actgataaag aacaaacata ttttttagaa ctattacata aaatttttcg atacatcgat

7861 gttcaaacta tcatggattt tgaattagct ggcacatggt ttttccaaag agttgcttgg

7921 cttggatact ttaaaaaagt agaacctgat tttcaaatag tctatataga tagtatagat

7981 cgcgagaata agcaaatttt attttattac tacacatttt cagatatgcc taatgagaaa

8041 ttctacttag gcaaaaatga actagaacct atagattcaa aacttagatc ttttgatttt

8101 atgggaaata atttcgttta tgaaagaaga atatggcttc catattctga taaagataaa

8161 aaacttttat ttagatttaa aataaatggg caagaaccta gaatttcttt agctggaaaa

8221 caacataaga gtggattacc tattcataca ttcttaagag atatgcctgt aaaaaaatat

8281 acgcacattg aagatttctg gataattatg gatagagatg tgcaagccga tgataatggt

8341 gaacattttt atcgatatat gatgaataat catcctgaac aaaaaaatata ctttgcgata

8401 aatagaaact ctaatgactg gggaaggtta aagcgagaag gatttaatct tattgatttc

8461 aaaagcaatg aatttaaaac tctcgtttcc caatgtagta gattaattag tagccatata

8521 gatgagtata tcattaaccc atttaaagac cattttgaat ttacaaaaaa atttatattt

8581 ctgcaacatg gagttactca caatgatcta tctgactggc tgaattcaaa aaaaatatta

8641 tcttgtataa taacggccac accagatgaa tataaccata tctctgagaa taaaagtaga

8701 tataaatatt ccactaaaga aactattctg actggctttc cgagacatga tgctttatta

8761 cgaggaaata aaacagaaac aagaactatt ttaatcatgc caacatggcg aaatagcata

8821 ttaggaaaaa atgccaaagg aaatgaaaga tcaataaatt cagaatttat gaatactcaa

8881 tatgccaaag catggggggc tattctttct agccctatat tagaaaagtt agctaatcaa

8941 tatgattttg aagtaatttt tgctcctcat aaaaacatag agccatattt agacttattc

9001 aacataccta aatatataaa acaatggaaa gcatctgaag gaaatattca gaaattattt

9061 caaaactcaa aatttatgat cactgattat tcttctgtag cttttgaaat ggggtactta

9121 aataaaaccg ttctatatta tcaatttgat aaggacagtt tctttagtgg tggacatgcc

9181 ttcaaaagag gctatttctc atatgaacaa catgggtttg gtcctgttgt gtatacagag

9241 gaagaattct ttattaatct agaaaatatc ctaaaaaata atgggaatcc atcagaaata

9301 tacaaaagta gaatagcaca aacattccct ttccaagatg gaaaatgttg tgaaagggtg

9361 tattttgcta tacaaaactt aaccacactt tattcataca cggagaaagc ctaatgaaaa

9421 gcgttattct tgatgttcta aaaaacacat acagtcaaaa ctactattgt aaaaagatcg

9481 atgtatataa gaacgttgtt tttattgata taaaaaccca atacttctat tttgcgctag

9541 aaatggcaaa tgaaaaagat attgaactta tctttagaga tgacgatagt ctacaaaaag

9601 ctaatcacta ttttaactat cctttccaaa aaggaaataa aatttatgtg tgcaattttt

9661 gcacaaacaa tctattttat atgaatgaag aactttcatt aaataatatg caaactctaa

9721 taaatagaat aagtcatatt attagtgttt tcattaatat tgagaataca tcattggtta

9781 gattatcaga atcaaatata cttctaaaaa atattaaaga aaactcagac ttatatttat

9841 ctaatcaagt atatctggat atgagtaatt ttatggaaga taaattgcta tctattaaag

9901 acactataca gtatattatc gataatgaat taagtattgc acgttttgga gatggtgaaa

9961 ttagatgcat ggtaactaga aatggttgta gttttcaaaa tcataattgg aaattaatga

10021 acgaacttag ggagatgtgt ttagaaaaaa atgataaact attaatttgt taccctagtt

10081 taatggttga taatgcattt tggtcccctt tttggaataa gttctggtct aaatgtaaat

10141 tttatttaag acaaaatatt attggtgatt catttattac tcgtccagaa gcattttact

10201 ttcatagttc cttaattgtc gaaaaatgga aagaagtttg gaataataag aatgtatgtt

10261 ttattacagg tgaaaactcc cgatttaata ccaaacatta tatttttaat aatataaaaa

10321 cagaaaacta catttattct aaacctaaaa atgcatatga tgatatagac aatatcattt

10381 caaaatgcac acaattaaaa gatatagaca tattcctaat agccttaggg cctaccggta

10441 cagcacatac cgtttaagta aactaggtta tagagcgtta gatattgggc atttgaataa

10501 cagctatgac actgtattta ataaggctcc tagacctgaa ttaatttcag ctaaataata

10561 cattgtaaag ccaattattt tcattaaaac aagacattaa atagcaagcg gtcaaatttt

10621 tcttaaaact tgcaaagttt aggaaaaata agaccgctta ttttattcat attcttatat

10681 aaaattagag tttattatga gcgtagccac atataattga taaagatatg cggtatagca

10741 atattaatac aaccctatca atttcatatt atcttaatca aatttatttt ttcgtttttt

10801 aaatattaaa ctattattta cttctgcaaa tctcccctat cccctcttta ctaaagaggg

10861 ggatttcctt tagatattaa caagctatga tctttcgaat agctttggtc tctcaataga

10921 ttcaggtgaa tgatcgatat ataaggaaaa taaaaacatt cgtcaaagac gagcgccatc

10981 gtaaagaaat ttggagccac agcatggctc actcagccgt aggctgatcg taaccacgta

11041 cggcttgccg tgcgtggcta ttgggtgatt atttaaaaac aggcgaaacg gcttttttac

11101 tttttcatta ccgcaatttg ttttagaatc aacggacgat ttactctcaa ggatttatga

11161 tgaaaaaatt atttttagcg gcgttaatcg catcgttcgg gttggctgcc tgcggtgtaa

11221 aaggcccgct ttattttccc gagcagcaac cggctcaaca gcaaacaaaa taatttctaa

11281 ccacggcaat tgagaagcac ctgtgtaaca agcggttaaa tttatccgat tttttgcaaa

11341 tacgagtgta gctattttgc cgtggtttta tttttcaaac acaacggaca agtaaataaa

11401 tgaatcattt caactataaa aaccaacaac tttttgcgga agacgtttcc gtttcagaca

11461 tcatcaatca atacggtacg cctgcttata tctattctcg tgcaacgctt gagcgccgct

11521 ggcacgcttt tgataaagcg tttggctcgc acccgcactt aatttgtttt gcggtgaaat

11581 ccaattccaa tattgcttta ttaaatgtga tggcacgcct cggttcgggc tttgatattg

11641 tgtcgcaagg cgaacttgaa cgtgtacttg ccgccggcgg cgagccgagc aaagtggtat

11701 tttccggtgt ggcaaaatca catagcgaaa ttcaacgtgc attggaagtc ggcattcgtt

11761 gctttaatat cgaatccatc gccgagttac accgcattaa tgaagttgcc ggtcaattag

11821 gtaaaatcgc accgatttca ttgcgtgtaa atccggatgt ggatgcacat actcaccctt

11881 atatttccac cggtttaaaa gaaaataaat ttggggtaag cgtaacgaac gagggaaaca

11941 agtactggga gaagcgttac gagaaaaaac acaggcggaa aacctactgc gtgaaagtgc

12001 gctaaattgg acaattctcc gcccgtgcgg gctgaatacg gacgaaggcg aaacctttcg

12061 tttaattgaa aatgcggctg aactgcccag cagttatatg agccgtaaag cattagccaa

12121 tgcggtcttg tccgtactta acagtgaaaa caaaaactat aaaatcttct cagtctgtgc

12181 ctaacttcac aatccctttc actttggcac aagccatccg cttgtgccta tttcaaaatt

12241 tgttttaaca gttccaataa aaccggtaat tccgttactc cctctcgatc gacaaaatgc

12301 ccgcccgttg ccaagcgaat ataatccgct tgtaagtatt gcgctaatcg atcgctgaac

12361 gaatggggaa cgacaacatc atttaatgca gatatgacgt aagacttttg cggtaaacaa

12421 gcggtttgat ttgtataaaa atttgcaaac tcatccaatt ccggtaaatg cggtaattgt

12481 tcataaaaac cggaaacaaa aatcgccgtt tttacttttt gttgcgtcac cgcaagataa

12541 ttcagtaacg caatgcagcc caaactatgt ccgatgagta aggtattttc atctaactga

12601 agtgtatttc ggtgatgttc aagccacgct tgcggattcg gctgatcgga attcggcatc

12661 gctaaacatt cacattccca tcctaatttt tccaattcgt ttttcagcca cggaaaccaa

12721 tttcggctcg ggttcgccgt ataaccgtgc gtgatatata cttttctcat

//

LOCUS **CP000569.1** 13518 bp DNA linear BCT 20-APR-2021

DEFINITION Actinobacillus pleuropneumoniae L20 serotype 5b
capsular polysaccharide gene locus, complete sequence.

ACCESSION CP000569 REGION: complement(1801340..1814857)

VERSION CP000569.1

KEYWORDS .

SOURCE Actinobacillus pleuropneumoniae serovar 5b str. L20

ORGANISM Actinobacillus pleuropneumoniae serovar 5b str. L20
Bacteria; Proteobacteria; Gammaproteobacteria; Pasteurellales;
Pasteurellaceae; Actinobacillus.

REFERENCE 1 (bases 1 to 13518)

AUTHORS Foote,S.J., Bosse,J.T., Bouevitch,A.B., Langford,P.R., Young,N.M.
and Nash,J.H.

TITLE The complete genome sequence of Actinobacillus pleuropneumoniae L20
(serotype 5b)

JOURNAL J. Bacteriol. 190 (4), 1495-1496 (2008)

PUBMED 18065534

REFERENCE 2 (bases 1 to 13518)

AUTHORS Foote,S.J., Bosse,J.T., Bouevitch,A.B., Langford,P.R., Young,N.M.
and Nash,J.H.E.

TITLE Direct Submission

JOURNAL Submitted (09-FEB-2007) Institute for Biological Sciences, National
Research Council of Canada, 100 Sussex Drive, Ottawa, Ontario K1A
0R6, Canada

FEATURES Location/Qualifiers

source 1..13518

/organism="Actinobacillus pleuropneumoniae serovar 5b str.
L20"

/mol_type="genomic DNA"

/strain="L20"

/serotype="5b"

/note="K locus: KL05"

/db_xref="taxon:416269"

CDS 1..1473

/gene="modF"

/locus_tag="APL_1586"

/inference="similar to AA

sequence:UniProtKB/Swiss-Prot:P31060"

/codon_start=1

/transl_table=11

/product="putative molybdenum transport ATP-binding

protein"

/protein_id="ABN74670.1"

/translation="MPNINIQNALFSLAQHNKLSIESLEINTHDFWVIVGGNGSGKTA

FAQALHNSLSLYSGEYQNSFQHIALLSFEQQQKIIEQIFKHRNNDMISPDDFGLTARQ

IILNGSEKTQLCEEYEAKLRIQPLLDRPFIQLSTGESRKVLFCQMLVSEPDLLILDEP

FEGLDQASVAYWQDVMAQLGKQMAVVLISNRFNDIPNCATHIALLDNLQLILQGERQA

IEQQAVYSQLKFAEQNVNAPLPDSAAPLIQLPPNTNPFELKNVMIRYGEKTIIDDLTW

TVAPKQHWWIKGPNGAGKSTLLSIITGDHPQSYANSVHLFGRQRGSGETIWDIKKNIG

YVSSQLHMDYRVNCSALDVILSGFFDSIGVYQQVPSALQLKAMEWLERLHLANLAKKP

FRSLSWGQQRLLLITRAMVKHPPILILDEPLQGLDGVNRKLVKQFIEQLVTNSQTQLL

FVSHQDADAPNCITHLFEFVPQENGGYRYVQTALNQIENA"

CDS complement(1821..2471)

/gene="cpxA"

/locus_tag="APL_1585"

/EC_number="3.6.3.38"

/inference="similar to AA sequence:INSD:AAD30163.1"

/note="pubmed:9169799"

/codon_start=1

/transl_table=11

/product="ATP-binding protein"

/protein_id="ABN74669.1"

/translation="MISVKNVSKDYYTRSGKKTVLQDINFELKKGEKIGILGRNGAGK

STLIRLLSGVEPPTSGTIERNMSISWPLAFSGAFQGSLTGMDNLRFICRIYNADIEYV

KAFTEEFSELGDYLYEPVKKYSSGMKARLAFALSLSVEFDCYLIDEVIAVGDSRFAAK

CKHELFEKRKDRSIILVSHSPSAMKEYCDNAMVLDKGIMYKFENMDEAYKFYNSTL"

CDS complement(2468..3265)

/gene="cpxB"

/locus_tag="APL_1584"

/inference="similar to AA sequence:INSD:AAB64444.1"

/note="pubmed:9169799"

/codon_start=1

/transl_table=11

/product="capsule polysaccharide export inner-membrane protein"

/protein_id="ABN74668.1"

/translation="MQYGDQTTFRQSLAIQGRVIGALLMREIITRYGRKNLGFLWLFV
EPLLLTLFIVLMWKFIRADRVSDLNIIAFVITGYPMAMMWRNASNRTIGAISGNLSLL
YHRNVRVLDTLLARVILEVAGATIAQIIIMALVILLGWIEMPKDTFYMVMAWVLMAFF
ALGLGLIICSIAQKFEAFGKIWGTLSFVLLPLSGAFFFVHALPSQAQQYATLIPMIHG
TEMFRHGYFGDSVITYESISYLVICDVAMLLFGLIMVKNFSKGIEPQ"

CDS complement(3265..4422)

/gene="cpxC"

/locus_tag="APL_1583"

/inference="similar to AA sequence:INSD:AAB64443.1"

/note="pubmed:9169799"

/codon_start=1

/transl_table=11

/product="capsule polysaccharide export inner-membrane protein"

/protein_id="ABN74667.1"

/translation="METTITASPTEKLQKPVKQKKSWLKKLNPLFWVTVAIPTVLSAF
YFGSVASDIYISESSFVVRSPQNQTALTGVGALLQGSGFSRAQDDTYTVQEYMHSRTA
LEQLMKDLPIREYYENQGDIIARFNGFGLNNSKEAFYKYFRDRLSVDFDSVSGIASLR
IRAFNAEEGQQINQKLLAEGETLINRLNERARKDTISFAEQAVTEAENNVNETANALS
KYRIKNKIFDLPAQSGVQLSLISSLKSELIRVETQLAQLQSITPDNPQVDALLMRQKS
LRKEIDEQSKQLSSNSNSSIAIQTADYQRLVLANELAQQQLTAALTSLQNTKNEADRQ
QLYLEVISQPSKPDWAEEPYRLYNILATFFIGLMLYGVLSLLIASVREHKN"

CDS complement(4448..5632)

/gene="cpxD"

/locus_tag="APL_1582"

/inference="similar to AA sequence:INSD:AAB64442.1"

/note="pubmed:9169799"

/codon_start=1

/transl_table=11

/product="capsule polysaccharide export protein"

/protein_id="ABN74666.1"

/translation="MKLIKLRLLLSLGLVASLAACSSLPTSGPSHSAILEANSQNSDK

PLPEVNLVELDNGLVQQLYQTQQSQQFSGFLGTAGGAGYAGAVNVGDVLEISIWEAPP

AVLFGGTFSSEGQGSGHLTQLPAQMVNQNGTVTVPFVGNIRVAGKTPEAIQSQIVGAL

QRKANQPQVLVKIANNNSADVTVIRQGNSIRMPLSANNERVLDAVAAVGGTTENIEDV

TVKLTRGSQVKTLAFETLISDPAQNIMLRAGDVVSLLNTPYSFTGLGAVGNNQQMKFS

SKGITLAEAIGKMGGLIDTRSDPRGVFVFRHVPFSQLSLDQQTQWGAKGYGMGMDVPT

VYRVNLLEPQSLFLLQRFPMQDKDIVYVSNAPLSEFQKFLRMIFSITSPVTSTTNAIR

AY"

CDS 5961..6926

/gene="cps5A"

/locus_tag="APL_1581"

/inference="similar to AA sequence:INSD:AAC26630.1"

/note="pubmed:9632602"

/codon_start=1

/transl_table=11

/product="putative glycosyltransferase"

/protein_id="ABN74665.1"

/translation="MSSIMTRPIINHVMSRDIQSGIFSSILEYFTDFGSNEFQHIVSV

SPIPEAKVYHYHRPHLEEKLLPNSVCTVHHDLNDPDPWHAKYRFIPRYMEAGAIICLN

YTQKEILISQGLPEHKLFVIPHGYNQKVLFPKKIKEISSTDKITLGIASRRYGRRVKG

DAYLFELAKRLNPDHFKFIFVGKDRQYSALEMQDLGFEAQVYERLPYRMFQSFYNNID

VLLMCSSHEGGPANIPEALATGTPIFSSNIGIPKDVVINYKNGLILTLDPDIDAEQIN

FICLEKPNIFENILDFSLKQSPSLAISWEKCIQQNILVYKKIIKG"

CDS 6929..8509

/gene="cps5B"

/locus_tag="APL_1580"

/inference="similar to AA sequence:INSD:AAC26631.1"

/note="pubmed:9632602"

/codon_start=1

/transl_table=11

/product="region 2 capsular polysaccharide biosynthesis

protein"

/protein_id="ABN74664.1"

/translation="MSISILVPDSLHINKRNFSSFFSWIEKNKINIHFENNNKDWISL

YGVYDSKLGILYEKIDILTKIEEEELFAFCVYDLNIFNICRAELLSLVATRPEWYNED

YPNNLREIYKKLYTNNRSELLQNMAAAWYWVDFWKKRLSELKQFSHCCVFSGGLIYQK

SLIELLKYTPTKVMVMESLFTGNEYYCEERYSSIANNSDIKHLAIFNSYKKTFSSKSE

YDKERMKAINKFLLMKNKNVQQPTDSEILVFKQQKPIITIIGQVINDFSVLEYKGRGL

STIKIYKELISKLSENGFNVVLKTHPWEEKKNNIRTSLTKNIIEEFLKNLTENQQECI

KIVDHYSIKKLFKQSDFIISLNSQGLLEAAFDGIKPIQLGNAFYGKKGFTYDYDFLDI

DQLVNDLVVNKLTPTLSLEEFDLFEEFITILLQKHAVSIHASGVSVLSRKFNLPTIIP

LVENVPKEKSKTTLPTQKDVVKKENTTIVNMVELPKVVPQSDKNRKYQKFRNNPRQFF

ADSRNPVIRSLMYFFPYK"

CDS 8524..9672

/gene="cps5C"

/locus_tag="APL_1579"

/inference="similar to AA sequence:INSD:AAC26632.1"

/note="pubmed:9632602"

/codon_start=1

/transl_table=11

/product="region 2 capsular polysaccharide biosynthesis

protein"

/protein_id="ABN74663.1"

/translation="MLKKYQPFDLRKINEGHSSNAKLVLHSEACNIDAKISKFFCSQD

DINLENFIATFTDNYKAPEVYTAILKNCCITPRAPKLPRYWGIFFEENNIPYYVFNFL

SSEAKKRFNDAKYFYLEDDDRISLDLEKVNKIHISGLSVWFYTFRNVDHFLRECLPSL

VTLKQMGYDFSALNFICPEILPDIIDFLCDFGIDRRNIISLDYQWLSFDELIIPCFST

FGHLHTPTKYYTEITNYPIINSKSREDIKRIYVSRANAKMRRVINEDVIVSELIARGF

SIIEPGNYSKLEQREIFSNAEVVIGPHGMGIANSVFSKNLKAILEIMNTDYHRISYFR

TAQLKGCLYAAYYVNPLPLDASYNNNYGDIVIHKTKFLNFLDSILKEI"

CDS 9721..10581

/gene="kdsA"

/locus_tag="APL_1578"

/EC_number="2.5.1.55"

/inference="similar to AA

sequence:UniProtKB/Swiss-Prot:O68662"

/codon_start=1

/transl_table=11

/product="2-dehydro-3-deoxyphosphooctonate aldolase"

/protein_id="ABN74662.1"

/translation="MTILNKIVKVGNIEVANDKPFTLFGGMNVLESRDMAMRVCEQYV

EVTNKLGVPYVFKASFDKANRSSIHSYRGPGMEEGLKIFQELKDTFGVSIITDVHEIY

QCKPVAEVVDIIQLPAFLARQTDLVEAMARTGVVINVKKPQFLSPGQMGNIVEKIAEC

GNENVILCDRGTNFGYDNLVVDMLGFNIMKKVSKGCPVIFDVTHSLQCRDPFGAASGG

RRDQVTELARSGMAIGLAGLFLEAHPDPNSAKCDGPSALPLSKLEAFVSQMKAIDDLV

KSFEEIDTSR"

CDS 10627..11385

/gene="kdsB"

/locus_tag="APL_1577"

/EC_number="2.7.7.38"

/inference="similar to AA

sequence:UniProtKB/Swiss-Prot:Q7VNY6"

/codon_start=1

/transl_table=11

/product="3-deoxy-manno-octulosonate cytidylyltransferase"

/protein_id="ABN74661.1"

/translation="MKFTIIIPARYASTRLPRKPLLDILGKPMIQHVWERAKQAGGHR

VIIATDHSEIAEVVTRFGGEVCLTSDKHSSGTERLAEVVSKMNISDDEIIVNVQGDEP

LIPPCIIKQVAENLDNHQVNMATLAVKLTQRDELFNPNVVKVLSDKNGMALYFSRAAI

PFARDNFPDCSDDFVTQNQYLRHIGIYAYRAGFIKQYVQWQPTALEQLESLEQLRALW

NGEKIHLDIALETPEVGVDTQEDLERVRLILSNK"

CDS 11397..12332

/gene="kpsF"

/locus_tag="APL_1576"

/inference="similar to AA

sequence:UniProtKB/Swiss-Prot:P45313"

/codon_start=1

/transl_table=11

/product="arabinose-5-phosphate isomerase"

/protein_id="ABN74660.1"

/translation="MNYLASARETLSLYTQAIDSLHNRLSTEFNQAIEMILSCEGRLV

VAGIGKSGLVGQKMVATFASTGTPSFFLHPTEAFHGDLGMLKPIDIVILISNSGETDD

VNKLIPSLKGFGNKIIAMTGNSHSTLAQHADIILNIGVEKEACTNNLAPTTSTLVTMA

LGDALAIALIKARNFQAMDFARFHPGGSLGRKLLCTVKDVMIRSLPIVSPTAIFSECL

NIMNEGRIGVALVMEHDCLLGIITDGDIRRLLADKGANSLLMTADQIMTKNPKTILES

TFLAKAEEEMKSLHVHSLVVMNEENRVVGIFEFSN"

CDS 12350..12904

/locus_tag="APL_1575"

/inference="similar to AA

sequence:UniProtKB/Swiss-Prot:P44085"

/codon_start=1

/transl_table=11

/product="hypothetical protein"

/protein_id="ABN74659.1"

/translation="MFKKITLFSFIALIAGCSSSSQLETFPGEFANADYVLSDKDAQR

WVVASRQVEQCIYPNLTRIQQQAFSKEDSYIHSQYVFFYPLEEIIGEQYVKIIQDDEK

SMGYAQYLFKKFRDNQEFEPLADKQCLVLREKAKNDLAVVKGQYKSGMVEETKSEAKN

ADGVATNQNKFFFDIIKWGSMLLL"

CDS complement(12976..13518)

/gene="ydeN"

/locus_tag="APL_1574"

/inference="similar to AA sequence:RefSeq:YP_089573.1"

/codon_start=1

/transl_table=11

/product="hypothetical protein"

/protein_id="ABN74658.1"

/translation="MKKVYVTHGYTANPTRNWFPWLKNELEKLGWECECLVMPNSDQP

NPQAWLEHHQNTLQLDENTLLIGHSLGCIALLNYLAVTQQKVKTAIFVSGFYEKLPTL

PELDSFADFYANQTACLPQKSYVISALNDVVVPHSFSDRLAQYLQADYIRLATGGHFV

DREGVTELPVLLELIKQISN"

ORIGIN

1 atgccaaata tcaacatcca gaacgcctta ttttcccttg ctcaacacaa taaactgtcg

61 attgaatcgc tggaaatcaa tactcacgat ttctgggtga ttgtcggcgg taacggctcg

121 ggtaaaactg ctttcgccca agcgctacat aattcacttt cactatattc gggcgaatat

181 caaaacagct tccaacatat cgctttactt tccttcgagc agcaacaaaa aatcatcgag

241 caaatcttta aacaccgtaa caacgatatg atttcaccgg atgatttcgg tttaacagca

301 cgtcaaatta tcttaaacgg tagcgaaaaa acgcaattat gcgaggaata tgaggctaaa

361 ttacgcattc aaccgttatt agatcgcccg tttattcagc tctccaccgg cgaaagtcgc

421 aaagtgttat tttgccaaat gttggttagt gaaccggatt tattgattct agatgagcct

481 ttcgaagggt tagaccaagc ctcggtcgct tattggcaag acgttatggc gcaactcggc

541 aagcaaatgg cggtggttct gatttctaac cgctttaacg atattcccaa ctgtgctacg

601 catatcgctt tattggataa tctacaactg attttacaag gcgaacgcca agcgattgag

661 caacaagcgg tctattctca gctaaaattt gcagaacaga atgtgaatgc gccgttaccg

721 gacagtgccg caccgctgat tcagctccca ccgaatacca atccgttcga actgaaaaac

781 gtgatgattc gttacggcga aaaaaccatt attgatgatc taacttggac ggttgcccca

841 aaacaacatt ggtggattaa aggcccgaac ggagccggca aatcgacctt actttcgatt

901 attaccggcg atcatccgca gtcttatgcc aattctgtgc atttattcgg tcgtcagcgt

961 ggctccggcg aaacgatttg ggatattaag aaaaatatcg gctatgtaag tagtcaatta

1021 catatggatt atcgggtgaa ttgttcggcg ttagatgtga ttttatccgg cttttttgat

1081 tcaatcggtg tttaccaaca agtgccaagt gccttacagc tcaaagcaat ggaatggctg

1141 gaacgcttac atttagccaa tctggcgaaa aaaccgttcc gttcactttc gtgggggcaa

1201 caacgtttat tattgattac tcgagccatg gtaaagcatc cgccgattct aattttagac

1261 gaacctctgc aaggcttgga tggtgtaaac cgcaaattgg ttaaacagtt tatcgagcag

1321 ttggtcacta acagccaaac ccagttgcta tttgtttctc atcaagatgc ggacgcccca

1381 aattgcatca cccatttatt tgaatttgtc ccgcaagaga atggtggtta tcgatacgta

1441 cagacggctt tgaatcaaat agaaaacgcc tgagatttaa ccacggaaaa cacagattgc

1501 acggagttta atgaatcggt cagttgacgt aatgtttgga taatattgtt tttttcaggt

1561 ttttcttatc tcgtaaggaa ctaataaaac tttaatcgga aactgcaact actcgcaata

1621 tcataatttg ttaaccttta aaggaaatcc ccctctttag taaagagggg ttaggggaga

1681 tttgtcaata gagatatgaa attgaataga acttcatttt ttatatttat aaaagcgtta

1741 attagcatat ttcttcgcta atttattctg tcaaatctcc cccagcccct ctttgctaaa

1801 gaggggggat atgtgggact ttaaagcgtt gaattataga acttgtaagc ctcgtccata

1861 ttctcaaatt tatacataat ccctttatcc aataccattg cattatcgca atattctttc

1921 attgcagacg ggctatgtga aaccaaaata atcgaacgat ctttgcgctt ttcaaataat

1981 tcatgtttac attttgccgc aaaacgggaa tcgcccacag caatcacttc atcaattaag

2041 tagcaatcaa actctaccga aagtgacaaa gcaaaggcaa gtcgggcttt cataccggag

2101 gaatatttct taaccggctc atataaataa tcacccaatt cggaaaattc ttcggtaaag

2161 gctttaacgt attcaatatc cgcattatat atacggcaaa taaagcgtaa attatccata

2221 ccggttaaac tgccttggaa cgccccgctg aaagcgagcg gccaagatat cgacatatta

2281 cgttcgatag tacctgatgt tggcggctca acaccactta acaaacggat tagcgttgat

2341 ttccctgcac cgttacgccc taaaataccg attttctcgc ctttttcag ctcaaaatta

2401 atatcttgca atacggtttt tttaccgctt cgagtatagt aatctttact cacatttttt

2461 acgctaatca ttgcggttcg attcctttac tgaagttttt taccataatg agcccaaaaa

2521 gtaacatggc tacatcacat attacgagat agcttatact ttcatatgtg ataacactgt

2581 cgccaaaata accgtgacga aacatttccg tgccgtgaat catcggtatt aaggttgcat

2641 attgttgagc ttggcttggt agcgcatgca caaagaaaaa tgcgcctgaa agaggtaaaa

2701 gaacaaagct taatgttccc cagattttgc caaatgcttc aaatttttgt gcaatagaac

2761 aaataatcaa gcctaatcct aatgcaaaaa atgccattaa tacccacgcc ataaccatat

2821 aaaacgtatc tttcggcatt tctatccagc ctaataaaat gactaatgcc ataataatga

2881 tttgggcaat cgttgcacct gctacttcaa gtatgacacg agccagtaag gtatctaata

2941 cgcgaacatt acgatgataa agaagactca agttaccgga aattgcaccg atagtgcggt

3001 ttgacgcatt acgccacatc atggccattg gataaccggt aatcacaaaa gcaataatat

3061 ttaaatcgga aacgcgatcc gctcggataa atttccacat caaaacgata aataaagtga

3121 gtaatagcgg ctcaacaaac agccataaaa aacccaaatt ttttcgtccg taacgcgtaa

3181 taatttcccg catgagtaat gcaccgatta ctctcccttg aatggcgaga gattggcgga

3241 aagttgtttg atcaccgtat tgcattagtt tttgtgctct cttacgcttg caattaataa

3301 acttaataca ccataaagca tcagaccgat aaagaatgtc gctaaaatat tatataagcg

3361 ataaggctct tccgcccagt ccggtttgct tggctgactg attacttcta aataaagttg

3421 ctggcgatcc gcttcatttt tcgtattttg taatgaggtt aatgctgcgg tcaattgttg

3481 ctgtgccagc tcgtttgcaa gtactaagcg ttggtaatcg gcagtttgaa tagcaataga

3541 gctattactg ttactggaaa gctgttttga ttgctcatcg atttccttac gtaaactttt

3601 ttggcgcata agcaatgcat caacttgtgg gttgtccggt gtaatagatt gcaattgagc

3661 caattgtgtt tctacacgaa tcaattcgct ttttaggctg gaaattaatg aaagttgtac

3721 gccggattgt gccggtaaat caaagatttt atttttgata cggtatttac ttaaagcatt

3781 tgccgtttcg tttacattat tttccgcttc tgtaaccgct tgttccgcaa atgaaatggt

3841 atcttttctt gcacgttcgt ttaaacggtt aataagcgtt tcaccttcgg caagtaattt

3901 ttgattaatt tgttgcccct cttccgcgtt aaatgctcga atacgtaagc tggcgatacc

3961 ggaaacagag tcaaagtcca cacttaagcg atctcggaaa tatttataaa acgcttcttt

4021 actattattt aaaccaaatc cattaaagcg agcgataata tcgccttgat tctcatagta

4081 ttcacgtatt ggcaagtctt tcattaactg ttctagtgcc gtacgagaat gcatatattc

4141 ttgtacggta taagtatcat cttgagctcg agaaaatccg gaaccttgta ataaggcacc

4201 gacaccggtt aaagcggtct gattttgagg agatcttaca acgaagcttg attccgaaat

4261 ataaatatcg gaagcaacag aaccgaaata aaaggctgat aataccgtag gaatcgctac

4321 agttacccaa aataacggat taagcttttt taaccaactt tttttctgtt taaccggttt

4381 ttgtagtttt tctgtcggac ttgccgtaat agttgtttcc atattttatc cttataaatt

4441 caatatatta ataggcacga atagcattag tcgtacttgt aaccggcgaa gtaatcgaga

4501 aaatcattct caagaatttt tggaattcgg acaacggtgc atttgataca tagacaatat

4561 ctttatcttg catcgggaag cgttgtaata aaaacagtga ttgcggctca agtaaattca

4621 cacgataaac cgtcggtaca tccataccca taccatagcc tttcgctccc cattgtgttt

4681 gctgatctaa acttaattga gaaaaaggca catgacggaa gacgaatacc cctctcggat

4741 ccgaacgagt atcaattagg ccacccatct taccgatagc ttcggcaagc gtaattcctt

4801 tacttgagaa tttcatttgc tggttgttac ccaccgcacc taaaccggta aagctataag

4861 gcgtgtttag caacgaaacg acatcgccgg cacgtaacat aatattttgc gccggatcgg

4921 aaattagagt ttcaaacgct aatgttttga cttgcgagcc acgagttaat tttacggtaa

4981 cgtcttcaat attttcagtt gtaccgccta ctgctgcaac agcatctaac acacgttcgt

5041 tattcgcact cagcggcata cgaatactgt taccctgacg aataaccgta acatccgcag

5101 agttattatt cgcaattttt actaatactt gtggctgatt cgctttacgt tgcaatgccc

5161 caacaatttg agactgaatc gcttccggtg ttttacctgc aacacgaata ttacccacaa

5221 acggcacagt aaccgtaccg ttttggttaa ccatttgcgc cggtaattgc gttaaatgcc

5281 cgctaccttg cccttcagaa ctaaaagtac cgccaaacaa cactgccggt ggcgcttccc

5341 aaattgaaat ttcaagaaca tcccccacat tgaccgcacc ggcatatcca gcaccgccag

5401 ccgtgcctaa aaagccggaa aattgctgac tttgctgagt ctgatacaac tgctgaacta

5461 agccattatc taactccact aaattaactt ccggtaaagg tttatctgag ttctgggaat

5521 tagcctctaa aatcgcacta tggctagggc ctgaagtggg taagcttgag caggcagcca

5581 aactagcaac cagccccaaa gaaaggagta atctaagttt gatgagtttc atctaatttc

5641 tcttcaatat attaaggaat aacaactata taggtatgtc ttaaaatcca cataaagatt

5701 gattttaata agttacctaa tcaagagaaa ttaaatataa gaaatttaca aacaaattaa

5761 aaaatgtatt tttttcaaa aaaagtaaat caagaggggc gttatacaga taaacattat

5821 aatttaaaag ccatataaaa tacggagttt cccctagata gttgataaat ttctcattta

5881 tatttatgaa attccgatga aaaatttatc aactatctag ggtaactcca taacgtattc

5941 gtatttcagg agtattttta atgtctagca taatgactcg tcctataatt aatcatgtaa

6001 tgtctagaga tattcaaagt ggcatattta gttctatttt agaatatttt actgattttg

6061 gttccaatga atttcaacat attgtcagtg tatctccaat acctgaagct aaagtttatc

6121 actatcaccg tccacaccta gaagaaaaat tattacctaa ttctgtttgt acagtacatc

6181 atgacctcaa tgatccagat ccttggcatg ctaagtatag atttattcct agatatatgg

6241 aagctggggc tataatttgt ttaaattaca ctcaaaaaga aattttaata tctcagggac

6301 ttccggaaca taagttattt gtgattcctc acggatataa tcaaaaagta ttatttccta

6361 agaaaattaa agaaatatca agtacagata aaattacctt aggaattgct tcacggagat

6421 atggtagaag agtaaaagga gatgcatatt tatttgaatt agcaaaaaga ttaaatccag

6481 accattttaa atttattttt gttggtaaag atagacaata tagtgcctta gaaatgcaag

6541 atctaggatt tgaagctcaa gtatatgaaa gattgccata tagaatgttt caaagttttt

6601 ataataatat tgatgtacta cttatgtgta gtagtcatga aggtggacct gcaaatatcc

6661 ccgaagcatt agctactggg acacctatat tttcatctaa cataggtata cctaaggatg

6721 ttgttattaa ttataagaat gggttgattc taaccttaga tccagatata gatgctgaac

6781 agattaattt tatttgcctt gaaaaaccaa atatatttga aaatatatta gatttttcac

6841 taaaacagtc tccaagttta gcaatttctt gggagaaatg tattcaacaa aatattttag

6901 tttataaaaa aataattaag ggttaattat gtccatttct attctagtac ctgattcttt

6961 acacattaac aaaagaaact ttagttcatt cttcagttgg attgagaaaa ataaaataaa

7021 tatccatttt gaaaataata ataaagattg gatttcatta tatggtgttt acgattcaaa

7081 attgggtatt ctatatgaga aaatagatat tcttactaag attgaagaag aggaattatt

7141 tgctttttgt gtttatgatc taaatatttt caatatttgt agagctgaat tattatcttt

7201 agtagccaca agacccgaat ggtataatga agattatcct aataacttaa gagaaatata

7261 caaaaaactc tatactaata atcgaagtga attattgcaa aacatggctg ctgcttggta

7321 ttgggttgat ttctggaaaa aacgcctatc tgagttaaaa caattctctc attgttgtgt

7381 attttcagga ggtttaattt atcaaaaatc tttgattgag ttattaaaat atactccaac

7441 taaagttatg gttatggaaa gcctatttac aggaaacgaa tattattgtg aggaacgtta

7501 ttcatcaatt gctaataata gcgatattaa acatttagct atttttaact cttataaaaa

7561 aacatttagt tcaaaaagtg aatatgataa ggaacgaatg aaagctatta ataagttcct

7621 attaatgaaa aataagaatg tccaacaacc tactgattct gaaatattag tatttaaaca

7681 acaaaaacca ataattacta ttattggaca agtgataaat gatttttcag tcctagaata

7741 taaagggaga ggactatcaa caattaaaat ctataaagaa cttatatcta aactatcaga

7801 gaatggattt aatgtagtat taaaaactca cccttgggaa gagaaaaaaa ataatatccg

7861 tacatcttta actaaaaata taatagaaga atttctaaaa aatctaactg agaatcaaca

7921 agaatgtata aaaatagttg atcactattc aataaagaaa ttatttaaac aatctgattt

7981 tattattagt ttaaattctc aagggctcct tgaagctgca tttgatggta taaaacctat

8041 acagttaggt aatgcttttt atggaaaaaa aggattcacg tacgattatg actttttaga

8101 tattgatcaa ttggtaaatg acttagtagt aaataaactt actccaacac tatctttaga

8161 agagtttgat ttgttcgaag agttcattac tatattatta caaaagcatg ctgtttctat

8221 tcacgcctct ggcgtaagtg ttttatctag aaaatttaat ttacctacta ttataccatt

8281 agtagaaaat gtccctaagg agaagtctaa aacaacatta cctactcaaa aagatgtggt

8341 aaaaaaggaa aatacaacaa ttgttaatat ggttgagtta cctaaagtag ttccacaaag

8401 tgataagaat aggaaatatc aaaaatttag aaacaatcct cgacaattct ttgcagattc

8461 taggaatcca gttattagaa gtttaatgta ttttttccct tataaataat ataggtctaa

8521 tttatgttaa aaaaatatca gccttttgat ttaagaaaaa taaatgaagg ccactctagt

8581 aatgctaagt tagttttaca ttctgaggcc tgtaatatag atgctaaaat ctctaagttt

8641 ttctgttcac aagatgacat taatttagaa aactttattg caacatttac tgataactat

8701 aaagcaccag aagtatatac ggcgatttta aagaattgtt gtattacacc tagagcacct

8761 aagctaccaa gatattgggg gatatttttt gaagaaaata atattcccta ctatgtattt

8821 aattttttaa gttctgaagc taaaaaaaga ttcaatgatg ctaaatattt ttacttagaa

8881 gatgatgata gaatttccct tgatttagag aaagttaata aaattcatat ttcagggctt

8941 agtgtatggt tttatacttt tcgtaatgta gatcattttt tacgagaatg tttgccttca

9001 ttagttacat taaaacagat gggatatgac tttagtgctt tgaactttat ttgtcctgaa

9061 atattacctg atattattga ttttctatgt gattttggga tagatagaag aaatattatc

9121 agccttgatt atcaatggtt atcgtttgat gaattaatta ttccttgttt tagtacgttt

9181 ggacatctgc atacaccaac aaaatattat actgaaatta ctaattaccc tattattaac

9241 tctaaatcta gagaggatat aaaaagaatt tatgtatcta gagcgaatgc taaaatgcgt

9301 agagtcataa atgaagatgt tattgtttcc gaactgattg ctaggggctt ttctatcatt

9361 gaacctggaa attattctaa gctggaacaa cgagagattt tttcaaatgc ggaagtagtt

9421 attggtccac atgggatggg aatagctaat agtgtttttt cgaaaaatct aaaagcaatt

9481 ttagaaataa tgaatactga ctatcacaga attagttatt ttagaactgc tcaattaaaa

9541 ggttgtttat atgctgctta ctatgtaaat cctttaccat tagatgcttc atataataat

9601 aattatggag atatagttat acataaaaca aaatttctca attttttaga ttcaatatta

9661 aaagaaatat aattaaagaa ctttaatttt agatttactt tactaagtgg aattaaaatc

9721 atgacaattt taaataaaat cgtaaaagtt ggcaatattg aagtggcgaa tgacaagccg

9781 tttactttat tcggtggtat gaacgtatta gaaagccgtg atatggcaat gcgtgtctgt

9841 gaacaatacg tagaagtgac caacaaactg ggtgtgcctt atgtatttaa agcctctttc

9901 gataaagcga atcgctcgtc aattcactct taccgaggac cgggtatgga agaaggttta

9961 aaaatcttcc aagaattaaa agatacgttc ggtgtgagca ttattactga cgtacacgaa

10021 atttatcaat gtaaaccggt agcggaagtg gtggatatta tccagttacc ggcattctta

10081 gctcgccaaa cagatttagt cgaagcaatg gcacgtaccg gtgtggtgat taacgtgaaa

10141 aaaccacaat ttttaagccc gggtcaaatg ggtaatatcg tagaaaaaat tgcagaatgc

10201 ggtaacgaaa acgtaattct ttgtgaccgt ggtactaact tcggctacga taatttagtg

10261 gtggatatgc tcggctttaa cattatgaaa aaagtatcaa aaggctgtcc ggtcattttt

10321 gacgtgactc actcactaca atgccgtgac ccgttcggtg cagcatcagg cggtcgccgt

10381 gatcaagtaa ccgaattggc tcgcagcggt atggcaattg gtttagccgg tttattcctt

10441 gaagcgcatc cggatccgaa cagtgcgaaa tgtgacggtc cgtcagccct accgttatca

10501 aaattagaag cattcgttag ccaaatgaaa gcgattgatg atttagttaa atcatttgaa

10561 gaaattgata catcaagata agaaatactt cccctcttaa ttagagggga tttttatgag

10621 gtttttatga agtttactat tattatcccg gctcgttatg cttcgactcg cttacctaga

10681 aaaccgttat tggatatttt aggtaagccg atgatccaac acgtttggga aagagcaaag

10741 caagcgggag gacatcgagt aattattgca acagatcatt ctgagattgc tgaggttgtt

10801 actcgctttg gtggagaagt ttgcctaact tcggataaac attcatccgg aacagaacgt

10861 ttagctgaag ttgttagtaa aatgaatatt agtgatgatg agattattgt taatgttcaa

10921 ggcgatgagc ctctcattcc accgtgtatt atcaaacaag ttgcagagaa tttagataat

10981 catcaagtaa atatggcaac attagcggtt aaattaactc aaagagatga attatttaat

11041 cctaatgtag taaaagtact ttcagataaa aatggtatgg cgttatattt ttctcgtgca

11101 gctattccat ttgctagaga taacttccct gattgttcag atgattttgt tactcaaaac

11161 cagtatcttc gtcatatagg tatttatgct tatcgtgcag gcttcattaa gcagtatgta

11221 caatggcaac cgacggcttt agaacagcta gaatctttag agcagttaag ggctttgtgg

11281 aatggagaaa agatacactt agatattgca ttagaaacgc ctgaagttgg tgttgatacg

11341 caagaagatt tagagcgagt tcgtttaatt ttatcaaata aataaggcta taaaatatga

11401 attacttagc aagtgctaga gaaacattat ctttatatac tcaagcaata gatagtttac

11461 ataatcgctt atctactgag tttaatcaag ctattgaaat gattttatct tgtgagggac

11521 gtttagttgt tgctggtatt ggtaaatcag gtttagttgg tcagaaaatg gttgctactt

11581 ttgcttctac gggaacacca agtttctttt tacatccaac cgaggcattt cacggtgatt

11641 tggggatgtt aaaacccatc gatatcgtca ttcttatttc aaatagtggt gaaactgatg

11701 acgtaaataa attaattcct agcctgaaag gttttggtaa taaaattatt gcaatgacag

11761 gtaattctca ttcaacccta gcacagcatg ctgatattat tttaaatatt ggcgtagaaa

11821 aagaagcctg tacaaataat ctggctccaa caacgtccac attagtaact atggcactag

11881 gagatgcact tgcgattgct ttaattaaag caagaaactt ccaagctatg gactttgctc

11941 gttttcatcc gggtggcagt ttagggcgaa aattactctg tacggttaaa gatgtaatga

12001 ttagatcatt accaatagtt tctccaacgg caatatttag cgaatgttta aatataatga

12061 atgaaggtcg aataggcgtt gctctagtta tggaacatga ctgtttactg ggaattatta

12121 ctgatgggga tattcgtcgt ttattagctg ataaaggagc taatagtttg ttaatgacag

12181 ctgatcaaat tatgacgaag aatcctaaaa ctatcctaga aagtactttt cttgctaaag

12241 cggaagaaga gatgaaatca ttgcatgtac attcattagt tgtaatgaat gaagaaaaca

12301 gagttgtagg tatatttgaa ttttcaaatt agaattatta taggatatta tgttcaaaaa

12361 aatcacttta ttcagtttta tcgcgttaat cgccggttgc tcttcttcct cacaactgga

12421 aactttccct ggggagtttg cgaatgcgga ttatgtgtta tcggacaagg atgcccagcg

12481 ttgggtggtg gcgagccgtc aggtggagca gtgtatttat ccgaacttga cgcggattca

12541 gcagcaagcg tttagcaagg aagattcata tattcattcg caatacgtat ttttctatcc

12601 gttggaagaa attatcggcg agcagtatgt aaaaaattatc caagacgatg aaaaatctat

12661 gggatatgcg caatacttgt ttaagaaatt cagagataat caggaattcg agccgttagc

12721 ggataagcaa tgtcttgtgt tgcgagaaaa agcgaagaac gatttagcgg tcgtaaaagg

12781 gcagtataag agcggaatgg ttgaagaaac gaagtccgaa gctaaaaatg cggacggcgt

12841 ggcgaccaat caaaataaat tcttctttga tattatcaaa tggggttcga tgctattact

12901 gtaatttgcg gttagtgtga cgttaaagag tataaaaacg agctgtcggt aaatcgttat

12961 cggcagctct ttttattagt ttgatatttg tttgattaac tccaataaaa ccggtaattc

13021 cgttactccc tctcgatcga caaaatgccc gcccgttgcc aagcgaatat aatccgcttg

13081 taagtattgc gctaatcgat cgctgaacga atggggaacg acaacatcat ttaatgcaga

13141 tatgacgtaa gacttttgcg gtaaacaagc ggtctgattt gcataaaaat ctgcaaagct

13201 atctaattcc ggcaaagttg gtaatttctc ataaaagccg gaaacaaaaa ttgccgtttt

13261 tactttttgc tgcgttaccg ccagataatt cagtaacgca atgcagccca aactatgtcc

13321 gatgagtaag gtattttcat ctaattgaag tgtattttgg tgatgttcca gccatgcttg

13381 cggattcggc tgatcggaat tcggcatcac taaacattca cattcccatc ctaatttttc

13441 caattcgttt ttaagccacg gaaaccaatt tcttgtcggg ttcgccgtat aaccgtgcgt

13501 tacatatact tttttcat

//

LOCUS **MZ450073** 18270 bp DNA linear 20-APR-2021

DEFINITION Actinobacillus pleuropneumoniae strain ATCC 33590 capsular

polysaccharide gene locus, complete sequence.

ACCESSION

VERSION

KEYWORDS .

SOURCE Actinobacillus pleuropneumoniae

ORGANISM Actinobacillus pleuropneumoniae

Unclassified.

FEATURES Location/Qualifiers

source 1..18270

/organism="Actinobacillus pleuropneumoniae"

/mol_type="genomic DNA"

/strain="ATCC 33590"

/serovar="6"

/note="K locus: KL06"

CDS 1..852

/gene="modF_1"

/codon_start=1

/transl_table=11

/product="ABC transporter ATP-binding protein ModF"

/protein_id="########.[1]"

/translation="MPNINIQNALFSLAQHNKLSIESLEINTHDFWVIVGGNGSGKTA

FAQALHNSLSLYSGEYQNSFQHIALLSFEQQQKIIEQIFKHRNNDMVSPDDFGLTARQ

IILNGSEKMQLCEEYAAKLRIQPLLDRPFIQLSTGESRKVLFCQMLVSEPDLLILDEP

FEGLDQASVAYWQDVMAQLGKQMAVVLISNRFNDIPDCATHIALLDNLQLILQGERQE

IEQQAVYSQLKFAEQNVNAPLPESATPLIQLPPNTNPFELKNVMIRYGEKTIIDDLTW

TVAPKQH"

CDS 937..1473

/gene="modF_2"

/codon_start=1
/transl_table=11
/product="ABC transporter ATP-binding protein ModF"
/protein_id="########.[1]"
/translation="MHLFGRQRGSGETIWDIKKNIGYVSSQLHMDYRVNCSALDVILS
GFFDSIGVYQQVPSALQLKVMEWLERLHLASLAKKPFRSLSWGQQRLLLITRAMVKHP
PILILDEPLQGLDGVNRKLVKQFIEQLVTNSQTQLLFVSHQDADAPNCITHLFEFVPQ
ENGGYRYVQTALNQIENA"

CDS complement(1823..2473)
/gene="cpxA"
/codon_start=1
/transl_table=11
/product="Polysialic acid transport ATP-binding protein
KpsT"
/protein_id="########.[1]"
/translation="MISVKNVSKDYYTRSGKKTVLQDINFELKKGEKIGILGRNGAGK
STLIRLLSGVEPPTSGTIERNMSISWPLAFSGAFQGSLTGMDNLRFICRIYNADIDYV
KAFTEEFSELGDYLYEPVKKYSSGMKARLAFALSLSVEFDCYLIDEVIAVGDSRFAAK
CKHELFEKRKDRSIILVSHSPSAMKSYCDNAMVLDKGIMYKFENMDEAYKFYNSTL"

CDS complement(2470..3267)
/gene="cpxB"
/codon_start=1
/transl_table=11
/product="Polysialic acid transport protein KpsM"
/protein_id="########.[1]"
/db_xref="COG:COG1682"
/translation="MQYGDQTTFRQSLAIQGRVIGALLMREIITRYGRKNLGFLWLFV
EPLLLTLFIVLMWKFIRADRVSDLNIIAFVITGYPMAMMWRNASNRTIGAISGNLSLL
YHRNVRVLDTLLARVILEVAGATIAQIIIMALVILLGWIEMPKDTFYMVMAWVLMAFF
ALGLGLIICSIAQKFEAFGKIWGTLSFVLLPLSGAFFFVHALPSQAQQYATLIPMIHG
TEMFRHGYFGDSVITYESISYLVICDVAMLLFGLIMVKNFSKGIEPQ"

CDS complement(3267..4424)
/gene="cpxC"
/product="hypothetical protein"
/protein_id="########.[1]"
/translation="METPIATSPAEKLQKPVKQKKSRFKKLNPLFWITVAIPTVLSAF
YFGSVASDIYISESSFVVRSPKNQTALTGVGALLQGSGFSRSQDDTYTVQEYMRSRTA
LEQLMQGLPVREYYENQGDIIARFNGFGLNNSKEAFYKYFRDRLSVDFDSVSGIASLH
IRAFNAEEGQQINQKLLAEGETLINRLNERARKDTISFAEQAVTEAENNVNETANALS

KYRIKNKIFDLPAQSGVQLSLISSLKSELIRVETQLAQLQSITPDNPQVDALLMRQKS
LHKEIDEQSKQLSSNSNSSIAIQTADYQRLVLANELAQQQLTAALTSLQNTKNEADRQ
QLYLEVISQPSKPDWAEEPYRLYNILATFFIGLMLYGVLSLLIASVREHKN"

CDS complement(4450..5637)
/gene="cpxD"
/codon_start=1
/transl_table=11
/product="hypothetical protein"
/protein_id="########.[1]"
/translation="MEIKKYNSIIGLALTTLFLSACSILPTSGPSHSAILEANSQNSD
KPLPEVNLVELDNGLVQQLYQTQQSQQFSGFLGTVSSAGYAGAVNVGDVLEISIWEAP
PAVLFGGTFSSEGQGSGHLTQLPAQMVNQNGTVTVPFVGNIRVAGKTPETIQSQIVGA
LQRKANQPQALVKIANNNSADVTVIRQGNSIRMPLTANNERVLDAVAAVGGTTENIED
VTVKLTRGSEVKTLAFETLISDPAQNIMLRAGDVVSLLNTPYSFTGLGAVGNNQQMKF
SSKGITLAEAIGKMGGLIDTRSDPRGVFVFRHVPFAQLSLEQQAQWQAKGYAIGMDVP
TVYRVNLLEPQSMFLLQRFPMQDKDIVYVSNAPLSEFQKFLRMIFSITSPVTSTTNAV
RAY"

CDS 5816..6958
/gene="cps6A"
/codon_start=1
/transl_table=11
/product="hypothetical protein"
/protein_id="########.[1]"
/translation="MLMKIAFIAWNSFQVLHFKPLLQALPCALLIIEKRRRSVPICKD
ILRDINNNIAYIRHTDIYAKIDGNFDVLVAQTTFEQLYLFRRTKIALLQYGYAKEPYN
YGTWRAFADLNLVYGNYAYERISYFSPTKITGCPRYDLWYQPLFHQKAKENYARVLDT
SKKTIVYAPSWGELSSFKLYIEEITKLSLFYNVLVKLHHNTLLLANKHQNYEKLYPNL
HFFYEGEDLLSLISVADIVISDFSGAIFDAIFCKKPVILFSIPLVNQPKLDKFSLEIA
HRSALGYEVSSPERVAITVEKALTEQKLADKMLYQQLFTGNENATQQVIDALQQLAEG
KYTLSQQQLYVRQTEKLLNIEKIKQQKNKKQSFNKIRQISKRLIKK"

CDS 7011..7439
/gene="cps6B"
/codon_start=1
/transl_table=11
/product="Glycerol-3-phosphate cytidylyltransferase"
/protein_id="########.[1]"
/translation="MKKVLTYGTFDLLHHGHIRLLERARSLGDHLTVAISTDQFNLGK
GKVCAYTYEERAHILKAIRYVDEVIPETNWEQKVEDVKNHEIDVFVMGDDWEGKFDFL
ADYCEVVYLPRTPDISTTQVKKMLAKKDLAAGQKQIHEKE"

CDS 7447..8589

/gene="cps6C"

/codon_start=1

/transl_table=11

/product="hypothetical protein"

/protein_id="########.[1]"

/translation="MFQILKKKLPTLQRVLRESDSQCSLLSYWYGFNLLSALERADHV
HVRKLADKMLNKGINIGHYFLAQSYFLCGEYTLAEQAVKKIPNFTKIPEVVFLYSDIL
IKCQRREEAWLLLEQCALLNKRKKVWIHLTNLVNTEADYRHLEQHIDKVRTTTPYLKS
DLLIHQRTNAALRAGLTETALALTELNPLPKQAKVKKKTTAYSDKLAAIALADLKKVL
DHKKIPFFLISGTLLGCIREGKLLGHDKDIDVGVWDEYSYEELANCLSTSGYFYVVPT
RTKHLVMLRHVNGIAIDVFIHYRESNDYWHAGVKIKWHNSPFNLVYTNFLGQQYLIPE
NYDLYLTENYGDWRTPKTKFDSAFDTPNMEVINEVEMKVYISKIIK"

CDS 8601..11558

/gene="cps6D"

/codon_start=1

/transl_table=11

/product="UDP-N-acetylglucosamine--peptide
N-acetylglucosaminyltransferase GtfA subunit"

/protein_id="########.[1]"

/translation="MKFLKNSYHNVIAPKGYHRGLVLYRKKQWTEALSCFEAAYSTSP
LHAKNTFKLGLCHLKLGNFSEAHSFIAKALEIAPYNTHWKKQLQQAERHLNNTYSSPH
KITTVVTRMKQSGISQSIGTAIRKTVLLIPSDYNHRVMADISSFIQYYKDKFDVYIIL
RELPEDIVYKNTHVLVKNGTSFGEYLKFTADYVIDSGTMNYSYRITDTNKWVSVWHGI
PYKKMFVDLDIKHISTAIRYDLAYDSMISMSNFYTDTFLRKAMRYDGEILQLGCAKMD
NLFSSISTSNADKANALRNELGLPNNKKVILYAPEFREVGELYFPFDPNKLLSHLGEE
YCLLTLLPFKGYIKQAENNIYYISDLDNKDALLIADLLISDYHELIYTFDRYNKPAVL
IQYDYESFVKQHTSRKQELEILASRKYVAQEAEELYQFNWNLLKRYSTHATLPEYLDS
SYIKHKLGIPFDKKIILYAPTYRKAGAVQLPFDPNTLLNYLDNDYVLITKMHYLNYLA
NTYNDVIDCTSHENMAELMKIADILISDYSSLVLDFAVLNKPIILFQYDYDEYMKQRG
VYFNFGDYLPKEQIIRTEFELYTLNWNKLNSDNSKIINEFYPLEDGKSTQRIVDKINF
NADLRFSKDIIFLVNDLNQIGGVHSFLKNMAKYYKQKYNSRIYVIAIKEFAEANSEYH
LLESEYIDFKLSSQYLNGACANILQNTDGIVISLQFSAHMYFQKYLTNAKSVLMFHGD
VKDMISRELYGPHLDWLNKGKLYNYQKLLLLTQSAVDLLKPHLNPEIQDKLGFMHNSI
DEEFSPIKQNKKYQLNTAVISRLDADKNIFAIIDLGKTILTQNSNIVVNIYGDGALKD
EFIAEINRHGLEHILKVRGFESNKAKIFSENNSLLLMSKSEGFGLVILEAYAYGKPVI
VFDSFTAAKEVVKHNQSGFLLPYGDYENVVKAIENSKNIKLKDIEMLFNNFSNPTVFA
KWDSLILSLDQTT"

CDS 11571..12602

/gene="cps6E"

/codon_start=1

/transl_table=11

/product="hypothetical protein"

/protein_id="########.[1]"

/translation="MKLLKKLFGRNKLEQPLISILVPCYNSRKTLPATLKSIQQSNYK

NLDVMIVDDGHEVTVEDIVSSFNDPRFRYFYKKNEGLGLTRNFGIDNAKGEFIFFLDS

DDLIYPDAFSNLINYMLENNLDVVSGVTVRRDFETNVESEWFRSLYKTNKISTFENRL

SQFDDSLSTNKLYRVKTLQEHNIYFETGLYEDKLFTAKLYSKIAKIGLISNRIYVWLV

YGNATSITTSKSISNFKGRMTAINNLWQYIPELRKAYQVAFYMNHDLLIYLREFKFYS

EEEKNEIYDIAYEFIQKHKKYVYKRLITQSLNRACLDALYIGDRDKFIYTSNILSNLF

QEELKRQHI"

CDS 12623..14089

/gene="cps6F"

/codon_start=1

/transl_table=11

/product="UDP-N-acetylglucosamine--peptide

N-acetylglucosaminyltransferase GtfA subunit"

/protein_id="########.[1]"

/translation="MAKNYVLTNCIPIGYGGLTTALLNRSAQISSVSKEPVKILTFRD

REFPKAVSKHLTERNGLRNGVTVTNIYDWLRENELTCGGMSFDKNKHFYTSLDLNAPN

STLDIEDGTVLRQLISEDNINGWVQVNHLRKDGSPAVIDNRRNGQRCLIVCDKQGKPH

RVFSYSWPFYHAWLDALFGDDHIDIIVDNDHEARFLTDYKRPNVSIIHYFHGTHCNDQ

GKVLPYAEFVFSRLEHFDLFVFPTFSQCYHAQQMFPHYSGFAYVPHALPILTNQKNEV

KRERNTFVVASRLEAIKRLDHAVLAIGAANKTSDGLKLHFLGHGSLIDSLQQQSKMLG

DIAIFHGHCSNVPDKLQKFSFYLLTSISEGLPVALLEAMQAGCLPIAYNINFGPSDVI

IHGVNGWLVEPGDVQALTKTIEMVSKIPENELEYMRENARNTAKQYSISRVLSDWKMC

KLQARKRRAEKLSLEIWKNKLTTELKDKIRKEMLNETN"

CDS 14076..14945

/gene="cps6G"

/codon_start=1

/transl_table=11

/product="hypothetical protein"

/protein_id="########.[1]"

/translation="MKPTNIIEKLSIRNNTFFIEGLAYIQGYNSPEFSNLYKYLCIIN

THTEELLEYELGSIRRYELKNQNYKGNIYDYTASGTATKNFKGIKINNLKEGLYEIKI

SVSDNNIERNYQSINFNSSEIDKFDFDDYFEYRLFKNQNKIYLAKRKLIGRAPISDYF

INIEKEWTKKQTMHIEGAFVIPGIDITEFNQARYYLIAQKAITQKQYSFALGQIKKAG

LGEKINNLQGSYNACYYATKMLKGIDMSALEFGFYDLYISLSYKSEVFTVKLNKQLEI

GHQLLKLVDNVEE"

CDS complement(15102..15890)

/codon_start=1

/transl_table=11

/product="hypothetical protein"

/protein_id="########.[1]"

/translation="MDYSVGKRTYQQLANKYQCSVRTIQRYLEKAPKAFLKAPQNKYL

NLIIDTTFFHRDFGVMVFIDSLSTKVIYHQIVKTEKDIYYKKAINHLREKGYIIQSIT

CDGRRGLLKDLMNTPTQMCQFHLVAIVMRALRKKHQSHAGRELKTIIKTLKVSSKNEF

YLKIHHWKIKHKAFLEERSDKQNEKGKYPYKHRNVRSAYTSIKRYMDYIFTYEKYPEL

NIEKTTNRIEGLFKELKDKLRPHSGLTRKHKILFIQDFLNKKSR"

CDS 16900..17505

/gene="lysA"

/codon_start=1

/transl_table=11

/product="Diaminopimelate decarboxylase"

/protein_id="########.[1]"

/translation="MNHFNYKNQQLFAEDVSVSDIINQYGTPAYIYSRATLERHWHAF

DKAFGAHPHLICFAVKSNSNIALLNVMVRLGSGFDIVSQGELERVLAAGGEPSKVVFS

GVAKSHSEIQRALEVGIRCFNIESIAELHRINEVAGQLGKIAPISLRVNPDVDAHTHP

YISTGLKENKFGVSVTNEGNKYWEKRYEKKHRRKTYCVKVR"

CDS complement(17728..18270)

/gene="ydeN"

/codon_start=1

/transl_table=11

/product="Putative hydrolase YdeN"

/protein_id="########.[1]"

/translation="MKKVYVTHGYTANPTRNWFPWLKNELEKLGWECECLAMPNSDQP

NPQAWLEHHKNTLKLNEETVLIGHSLGCIALLNYLAVTQQKVKTAIFVSGFYEQLPHL

PELDKFANFYANQTACLPEKSYVIAALNDVVVPHSFSDRLAQYLQADYIRLATGGHFI

DREGVTELPVLLELLKQILK"

ORIGIN

1 atgccaaata tcaacatcca gaacgcctta ttttcccttg ctcaacacaa taaactctcg

61 attgaatcac tggaaatcaa tactcacgat ttctgggtga ttgtcggcgg taacggctcg

121 ggcaaaaccg ctttcgccca agcgctacat aattcacttt cgttatattc gggtgaatat

181 caaaatagtt tccagcatat cgctttactt tccttcgagc agcaacaaaa aatcatcgag

241 caaatcttta aacaccgtaa caacgatatg gtttcaccgg atgatttcgg tttaaccgcc

301 cgtcaaatta tcttaaacgg tagcgaaaaa atgcaattat gcgaggaata tgcggctaaa

361 ttacgtattc agccgttatt agatcgcccg tttattcagc tctccaccgg cgaaagtcgc

421 aaggtgttat tttgccaaat gttagttagc gaaccggatt tgctgatttt agatgagcct

481 tttgaggggt tagaccaagc ctcggtcgct tattggcaag acgtgatggc acaactcggc

541 aagcaaatgg cggtggtact gatttccaac cgttttaatg atattcccga ctgtgccaca

601 catattgctt tactggataa cttacaactg attttacaag gcgaacgcca agagattgaa

661 caacaagcgg tctattctca gctaaaattt gcagaacaga atgtgaatgc accgttgccg

721 gagagtgcca caccgctgat tcaactccca ccgaatacta atccgtttga actgaaaaac

781 gtaatgatcc gttacggcga aaaaacgatt attgatgatc taacttggac ggttgcccca

841 aaacaacatt agtggattaa aggcccgaac ggagcaggaa aatcgacctt actttctatt

901 attaccggcg atcatccgca atcttacgct aattatgtgc atttattcgg tcgtcagcgt

961 ggctcgggcg aaaccatttg ggatatcaag aaaaatatcg gctatgtgag cagccaatta

1021 catatggatt atcgggtgaa ttgttcggcg ttagatgtga ttttatctgg cttttttgat

1081 tcaatcggtg tttaccaaca agtgccaagt gccttacagc tcaaagtaat ggaatggtta

1141 gaacgcttac atttagccag tctggcgaaa aaaccgttcc gttcactttc gtggggacaa

1201 caacgtttat tattgattac tcgtgccatg gtaaaacatc cgccgattct gattttagac

1261 gaaccgctac aaggcttaga cggcgtaaac cgcaaattgg ttaaacagtt tatcgaacag

1321 cttgtgacta atagtcaaac ccagttgcta tttgtttcgc accaagatgc ggacgcccca

1381 aattgcatca cgcatttatt tgaatttgtt ccgcaagaga atggtggtta tcgttatgta

1441 cagacggctt tgaatcaaat agaaaacgcc taagatttaa ccacggaaaa cacggattac

1501 acggagttta atgaatcggt cagttgacgt aatgtttgga taatattgtt tttttcaggt

1561 ttttcttatc tcgtaaggaa ctaataaaac tttaatcgga aactgcaact actcgcaata

1621 tcataatttg ttaaccttta aaggaaatcc ccctctttag taaagagggg ttaggggaga

1681 tttgtcaata gagatatgaa attgaataga acttcatttt ttatatttat aaaagcgtta

1741 attagcatat ttcttcgcta attcattctg tcaaatctcc tcctgcccct ctttgctaaa

1801 gaggggagat atgtgtggga ctttaaagcg ttgaattata gaacttataa gcctcgtcca

1861 tattctcaaa tttatacata atccctttat ctaataccat tgcattatcg caataagact

1921 tcattgctga cggactatgc gaaaccaaaa taatcgaacg atctttgcgc ttttcaaata

1981 attcatgttt acattttgcc gcaaagcgag agtcacctac cgcaattacc tcatcaatta

2041 agtagcaatc aaactcaact gaaagcgata aagcaaacgc aagtcgggct ttcataccgg

2101 aagaatattt cttcaccggc tcatataaat aatcgcctaa ttcggaaaac tcttcggtaa

2161 atgctttaac atagtcaata tccgcattat agatacggca aataaagcgt aaattatcca

2221 taccggttaa actgccttgg aacgccccgc tgaaagcgag cggccaagat atcgacatat

2281 tacgttcgat agtacctgat gttggtggtt caacaccact taacaaacgg attagcgttg

2341 atttccctgc accgttacgc cctaaaatac cgattttctc gcctttttttc agctcaaaat

2401 taatatcttg caatacggtt tttttaccgc ttcgagtata gtaatcttta ctcacatttt

2461 ttacgctaat cattgcggtt cgatcccttt actgaagttt tttaccataa tgagcccaaa

2521 aagtaacatg gctacatcac atatgacgag ataacttata ctttcatatg tgataacact

2581 gtcgccaaaa taaccgtgac gaaacatttc cgtgccgtga atcatcggta ttaaggttgc

2641 atattgttga gcttggcttg gtagcgcatg cacaaagaaa aatgcgcctg aaagaggtaa

2701 aagaacaaag cttaatgttc cccaaatttt accgaaggct tcaaattttt gtgcaataga

2761 acaaataatc aagcctaatc ctagtgcaaa aaatgccatt aatacccacg ccataaccat

2821 ataaaacgta tctttcggca tttctatcca gcctaataaa atgactaatg ccataataat

2881 gatttgggca atcgttgcac ctgctacttc aagtatgaca cgagccagta aggtatctaa

2941 tacgcgaacg ttacgatgat aaagaagact caaattaccg gaaattgcac cgatagtgcg

3001 gtttgatgca ttacgccaca tcattgccat tggataacca gtaatcacaa aagcaataat

3061 atttaaatcg gaaacgcgat ccgctcggat aaatttccac atcaaaacga taaataaagt

3121 gagtaatagc ggctcaacaa acagccataa aaaacccaag ttttttcgtc cgtaacgcgt

3181 aataatttcc cgcatgagta acgcaccgat tactctccct tgaatggcga gagattggcg

3241 gaaagttgtt tgatcaccgt attgcattag tttttatgct ctcttacgct tgcaattaat

3301 aaacttaata caccataaag catcagaccg ataaagaatg tcgctaaaat attatataag

3361 cgataaggct cttccgccca gtccggtttg cttggctgac tgattacttc taaataaagt

3421 tgctggcgat ccgcttcatt tttggtattt tgcaaagaag ttaaagctgc ggttaattgt

3481 tgctgcgcca gctcgtttgc aagtactaag cgttggtaat cggcagtttg aatagcaata

3541 gagctattac tattactgga aagctgtttt gattgctcgt caatttcctt atgtaaactc

3601 ttttggcgca ttaataacgc atcaacttgc gggttatccg gcgtaatcga ttgtaattgc

3661 gctaattggg tttctacacg aatcaactcg ctttttaagc ttgaaattaa tgaaagttgt

3721 acgccggatt gtgccggtaa atcaaaaatt ttatttttga tacggtattt acttaaagca

3781 tttgccgttt cgtttacatt attttccgct tctgtaaccg cttgttccgc aaatgaaatg

3841 gtatcttttc ttgcacgttc gtttaaacgg ttaataagcg tttcaccttc ggcaaggagt

3901 ttttgattaa tttgttgccc ctcttccgcg ttaaatgctc gaatatgtaa gctggcgata

3961 ccggaaacag agtcaaagtc cacacttaag cgatctcgga aatatttata aaacgcttct

4021 ttactgttat ttaatccgaa tccattaaag cgagcgataa tatcgccctg attttcataa

4081 tattcgcgaa caggtaatcc ttgcattagt tgctcaagag cggtacgaga acgcatatat

4141 tcctgtacgg tataagtatc gtcttgcgaa cgagaaaaac ctgaaccttg caataaggcg

4201 cctacgcctg tcagagcggt ctgattttta ggcgatctaa cgacaaaact tgattccgaa

4261 atataaatat cagaagcgac cgaaccgaaa taaaacgctg atagcacggt agggattgcg

4321 acagttatcc aaaataatgg attgagtttt ttaaagcggc tttttttctg tttaaccggt

4381 ttctgtaatt tttctgctgg actggtagca ataggtgttt ccatcttttg tccttataca

4441 ttcaatatat taataggcac gaacggcatt ggtcgtactg gtaaccggcg aagtaattga

4501 gaaaatcatt ctcaagaatt tttggaattc agacaacggc gcatttgaaa catacacaat

4561 atctttatct tgcattggga aacgctgtaa taaaaacatg gattgcggct caagtaagtt

4621 cacacgataa accgttggta catccattcc tatagcgtag cctttagctt gccattgtgc

4681 ttgttgttcc aaactcaatt gtgcaaaagg cacgtgacgg aatacgaaaa cccctctcgg

4741 atccgaacga gtatcaatta aaccgcccat cttaccgata gcttcggcaa gcgtaattcc

4801 tttacttgag aatttcattt gctggttgtt acccacagcg cctaaaccgg taaaactata

4861 aggcgtgttt agcaacgaaa cgacatcgcc ggcacgtaac ataatatttt gcgccggatc

4921 ggaaattaac gtttcgaatg cgagtgtttt tacttcagaa ccacgggtta gcttgaccgt

4981 cacatcttca atattttccg ttgtaccgcc tactgccgca accgcatcta atacgcgttc

5041 attattagcg gttaatggca tacgaatact attaccttga cgaataaccg taacatcagc

5101 agagttatta ttcgcaattt tgactaatgc ttgcggctga ttcgctttac gctgcaatgc

5161 cccaacaatt tgagactgaa tcgtttcagg tgttttacct gcgacacgaa tatttcccac

5221 aaacggcacg gttaccgtcc cgttttgatt aaccatttgt gccggtaatt gcgttaaatg

5281 cccgctacct tgtccttcgg aactaaatgt accgccaaat aatactgccg gcggcgcttc

5341 ccaaattgaa atttcaagta catcacccac attgactgca ccggcatagc ccgcgctgct

5401 tactgtgcct aaaaatccgg aaaattgttg gctttgctga gtctgataca actgctgaac

5461 taagccatta tctaactcca ctaaattaac ttccggtaaa ggtttatctg agttctggga

5521 attagcctct aaaatcgcac tatggctagg gcctgaagtt gggaggattg agcaagcaga

5581 aaggaacaat gttgttaaag ctaaccctat gattgaatta tattttttaa tttccatctt

5641 gtaaaaaggc tctattgaaa aagtgtgtaa aatgaatata aactatacat aattatagat

5701 acaatactat aattatatat tatagtctaa tatctcgttt tttaaaacga taacctaaaa

5761 tattactttt ctatagaaga ataggcatta ttttaataaa tataattagg tatgaatgtt

5821 gatgaaaata gcatttatcg cttggaatag ttttcaggtg ttacatttca agcccttatt

5881 acaagcttta ccgtgtgcat tattaattat tgaaaaacgg agacgtagtg taccaatctg

5941 taaggatatt ttgcgagata taaacaataa tatcgcttat atccgccata cggatatata

6001 tgcaaaaatt gatgggaatt ttgatgttct agttgctcaa actactttcg agcaacttta

6061 tttgtttcgc cgcaccaaaa ttgcattgct tcaatacgga tatgctaagg aaccgtataa

6121 ctacggcact tggagagcat ttgcagatct aaatttggtt tatgggaatt atgcctatga

6181 acgtatttcc tatttctctc caactaaaat aaccggttgt ccacgatacg atttatggta

6241 tcagccttta tttcatcaaa aagcgaaaga aaattatgcg agagtattag atacgagtaa

6301 gaaaacgatt gtatatgcac caagttgggg agaattatcc agctttaaat tatatataga

6361 agaaattacg aaattatctt tattttacaa cgtgttagta aaattacacc ataacacgct

6421 tttattagca aacaagcatc agaattatga aaaattgtat ccgaatttac attttttcta

6481 tgaaggtgaa gatcttctct ctcttatttc ggtagccgac attgttattt ccgattttag

6541 cggtgcgatc tttgacgcaa ttttctgtaa aaaaccagta atattatttt ctatcccatt

6601 agtaaatcaa cccaaattag ataaatttag tttggagata gcccatcgtt cggcgttggg

6661 atatgaggtt tcttcacctg aacgagtagc tataacggtg gagaaagcac taacagagca

6721 aaaattagca gataaaatgc tatatcaaca gctttttaca ggtaatgaaa atgcaacaca

6781 acaagtgata gacgctctac aacagcttgc tgagggtaag tatacattat ctcaacagca

6841 gttatatgtg cgacaaacag aaaaattatt aaatattgaa aaaataaagc agcaaaaaaa

6901 taaaaaacag tctttcaata aaataagaca gatttctaaa agattaatta aaaaataatt

6961 tttcatattt tctttttata ttttttatt tgacaatacg gagtttagga atgaaaaaag

7021 tattaaccta tggaaccttt gatttattac accatgggca tattcgttta ttagaaagag

7081 caagatcatt aggcgatcac cttactgttg ctatttctac cgatcaattt aacttaggaa

7141 aaggcaaagt atgcgcttat acttacgaag agagagcgca tattttaaaa gcaatccgtt

7201 atgtggatga agtaattcct gaaacaaact gggagcaaaa agttgaggat gtaaaaaatc

7261 acgaaattga cgtatttgta atgggtgatg actgggaagg caaatttgac ttcttagcag

7321 attactgcga agtggtttat ttaccgagaa cccctgatat ttcaacgact caggtaaaaa

7381 aaatgcttgc gaaaaaagat ctcgcagccg gacaaaaaca aatacacgaa aaagagtaat

7441 ttgttgatgt tccaaatctt aaaaaagaag ttaccaactt tgcaaagagt tctgagagaa

7501 agtgattctc agtgttcttt gcttagttat tggtatgggt ttaatctact ttccgcactg

7561 gaaagggcag atcacgttca cgtaagaaaa cttgccgata aaatgcttaa taaaggtatt

7621 aatatcggac actatttttt agcacagagt tattttctat gtggagaata tacgctagcg

7681 gagcaagcgg ttaaaaaaat cccaaatttt actaaaatac ctgaagttgt ttttttatat

7741 tcagatattc ttatcaaatg ccagcgtaga gaagaagctt ggctactatt agaacaatgc

7801 gctttactca ataaaagaaa aaaagtgtgg atacatctaa caaatttagt aaatactgag

7861 gcggattatc gacacttaga acaacatatt gacaaagtaa gaacaactac accttacttg

7921 aagtctgatt tgttaattca tcaaagaaca aatgcggcat taagagcagg tttaacagaa

7981 acggcattag ctctaacaga gcttaaccct ttgccaaagc aagcaaaagt taagaaaaaa

8041 acaaccgctt atagtgataa attagcggca attgcgctag cggatctaaa gaaagtatta

8101 gatcacaaaa aaataccttt ctttctgatt agcggcacgt tgctaggttg tattcgagaa

8161 ggaaaattat tagggcatga taaagatatt gatgtcggcg tttgggatga gtactcctac

8221 gaagaattag caaactgttt atctacatcg ggatactttt acgtagtacc aactcgaaca

8281 aaacatttag tcatgttaag acatgttaat ggtatcgcaa ttgatgtgtt tatccattat

8341 cgcgaatcta atgattattg gcacgcaggt gtcaaaataa aatggcataa ctcgccattc

8401 aacttggtat atacaaattt tctcggacaa caatatttaa tacctgaaaa ttacgattta

8461 tacctaacag aaaattacgg tgattggcgt acgccaaaaa ctaaatttga tagcgccttt

8521 gatacgccaa atatggaagt tatcaatgag gtggaaatga aggtttatat atctaaaata

8581 ataaaataag gaataaatga atgaaatttt tgaaaaactc ttatcataat gtgattgctc

8641 ctaaagggta tcatcgagga ttagttttat acagaaaaaa acaatggact gaagctttat

8701 cttgctttga agctgcttat agcacatctc ctttacatgc caaaaatacc tttaagctag

8761 gattatgtca ccttaaatta gggaatttct cagaggctca ttcatttatt gctaaagctc

8821 ttgaaatagc tccatataat acacattgga aaaagcagct ccaacaggct gaacgccacc

8881 ttaataacac atactcatct ccacataaaa tcactactgt tgtaactaga atgaaacaaa

8941 gtggtattag ccagtctatt ggtaccgcta tcagaaaaac agtactactt attccatcag

9001 actataacca tcgagtaatg gctgatattt catcatttat ccaatactat aaagataaat

9061 ttgacgtata tattatatta cgtgaattac ctgaagacat tgtgtataaa aatactcatg

9121 tattagtcaa gaatggaact tcatttggtg aatatttaaa atttactgca gattatgtta

9181 ttgactcagg tacaatgaac tatagctacc gtattactga tactaataaa tgggtttcgg

9241 tatggcacgg tatcccttat aaaaaaatgt tcgttgactt agatataaaa catatctcaa

9301 cagctatccg ctatgatctt gcctatgaca gtatgatttc aatgtctaat ttttatactg

9361 atacattttt acgtaaagca atgcgctatg acggtgaaat attacaactt ggctgtgcca

9421 aaatggataa tttattttca tctatttcta caagcaacgc tgataaagcc aatgccttac

9481 gtaatgaatt aggtttacct aataataaaa aggttatttt atatgctcct gaatttagag

9541 aggtaggtga actttatttc ccatttgatc ctaataaatt attatctcat ttaggtgaag

9601 agtattgttt acttacttta ttacctttta aaggatatat caaacaagca gaaaataata

9661 tctactatat ttccgattta gataataagg atgccctttt aattgctgat ttattaatta

9721 gcgattacca cgaattaatc tatacatttg atagatataa taagcctgca gttctaatac

9781 agtacgacta tgaatcattt gtaaaacaac atacttccag aaaacaagag ctagaaatac

9841 tagcatctag aaaatatgtt gcacaagaag cggaagaatt atatcaattt aattggaatc

9901 tactaaaaag atatagtaca catgctactt tacccgaata tcttgatagc tcatatataa

9961 agcataaatt aggcattccg tttgataaga agattatttt atatgcacca acttaccgta

10021 aagctggtgc cgtacaattg ccctttgacc caaatacatt actaaattac ttggataatg

10081 attatgtatt aatcacaaaa atgcattacc taaattattt agctaataca tataacgacg

10141 taattgactg tacctcacat gaaaatatgg cagagctaat gaaaatcgca gatatcttaa

10201 tcagcgacta ttcatcatta gttcttgact tcgctgtatt aaataaacca atcattctat

10261 tccaatatga ttatgatgaa tatatgaaac aacgaggagt ttacttcaat tttggagact

10321 atttacctaa agagcaaatt attcgaactg aatttgaatt atatacatta aactggaata

10381 agcttaactc ggacaatagt aagattatta atgaatttta tccacttgaa gatggaaaat

10441 ccacccaacg tattgtagat aaaataaatt ttaatgcaga tttaagattc agcaaggata

10501 ttattttctt agtaaatgat ttaaatcaaa ttggtggtgt ccactcattt ttaaagaata

10561 tggcaaaata ctataagcag aagtataact ctcgcattta tgttattgct attaaagaat

10621 ttgcagaagc aaactctgaa tatcacttat tagaaagcga atatattgac tttaaattat

10681 ctagccaata tttaaatggt gcttgtgcta atattttaca aaatacagat ggcattgtaa

10741 tttcattaca gttttctgct catatgtatt tccaaaaata tttaaccaac gctaaatcag

10801 ttctgatgtt ccatggtgat gttaaagata tgatttccag agaactttat ggaccacatt

10861 tagattggtt aaataaaggc aaactctata actatcaaaa gttattattg cttacacaat

10921 cagctgtaga cttacttaaa cctcatttaa atcctgagat tcaagataaa ttaggattta

10981 tgcataactc tattgatgag gaatttagtc caattaaaca gaataaaaaa tatcaattaa

11041 atactgcagt aattagccgt ctggatgcgg ataaaaatat atttgccatc attgaccttg

11101 gaaaaacaat tcttactcag aactcgaata ttgtagtaaa tatctatggt gatggagctt

11161 taaaagatga atttatagct gaaattaatc gtcatggtct agaacatatt ttaaaagtga

11221 gaggctttga aagtaataaa gctaaaattt tctcagaaaa taattcttta ttattaatga

11281 gcaaatcgga aggttttggt ctcgtgatac ttgaggccta tgcttatggc aaaccagtga

11341 ttgtttttga ttcatttaca gcagctaaag aagttgtaaa acataatcag tcaggatttt

11401 tattacctta cggtgattat gagaatgtag ttaaagccat tgaaaatagt aaaaatatta

11461 agttaaagga tattgaaatg ctctttaaca acttctctaa tccaactgtt tttgctaaat

11521 gggatagctt aatcctatct ttagatcaaa caacgtaaag gaaatatagc atgaaactat

11581 taaaaaaatt attcggccgc aataaattag aacagccttt aatttcgatt cttgtacctt

11641 gttacaattc tcgtaaaaca ttgcctgcaa cattaaaatc tattcaacaa tcaaattata

11701 aaaacttaga tgtaatgatt gttgatgatg ggcatgaagt gactgtagag gatattgtta

11761 gttcatttaa cgaccctcgc tttcgttatt tttataaaaa aaatgaaggc ttaggcttaa

11821 ctagaaattt tggtattgac aatgcgaaag gcgaatttat cttcttttta gactcagatg

11881 atctaattta tcctgatgct ttctcaaatt taataaacta tatgctggaa aacaatttag

11941 atgtagtatc tggtgttaca gtacgtcgcg actttgaaac taatgtggaa agtgagtggt

12001 ttagatcatt atacaaaacc aataaaatta gtacttttga gaatagatta tctcaatttg

12061 atgattcgtt atcgacaaat aaattatatc gtgtaaaaac ccttcaagaa cataatatat

12121 attttgaaac gggtttatat gaggataagc tgtttaccgc taaattatat tctaaaatag

12181 ccaaaatagg tttgattagt aatagaatat atgtttggtt agtttatggt aatgcaacta

12241 gtattacaac atctaaatct ataagcaatt ttaaaggaag aatgacagca attaataatt

12301 tatggcaata catccctgaa ttacgtaaag catatcaagt tgctttctac atgaaccatg

12361 atttattaat ttatctcaga gaatttaagt tctactctga agaagagaaa aatgaaatat

12421 acgatattgc ttatgaattc atccaaaaac ataagaaata tgtttataaa aggttaatta

12481 cacaaagctt aaatagagct tgcttagatg cactatatat aggagataga gataaattta

12541 tctatacttc aaatatttta tctaacttgt tccaagaaga attaaagaga caacatattt

12601 aaaattttaa gagaatatta atatggcaaa aaattatgta ttaacaaatt gtattcctat

12661 tggttatggt ggtttaacta cagcgttatt aaatcgttct gctcaaatct catcagtttc

12721 taaagaacct gttaaaattt taacgtttcg cgatagagaa ttccctaaag cagtttcgaa

12781 acatttaaca gaaagaaacg gattacggaa tggtgttact gttactaata tttatgactg

12841 gcttcgtgaa aatgagttaa cttgcggtgg catgtctttt gataaaaata aacattttta

12901 tacatcatta gatttgaatg ctccaaatag cactctagac atagaggatg gtactgtttt

12961 aaggcaatta atatctgaag ataatattaa tggctgggtt caagttaatc atttgcgaaa

13021 agatggctca cctgcagtaa ttgataaccg ccgaaatgga caaagatgct tgattgtctg

13081 tgataaacaa ggaaaacctc atagagtatt ttcttattct tggccgtttt atcatgcttg

13141 gcttgatgcc ctatttggag atgatcatat tgatattatt gttgataatg atcatgaggc

13201 acgttttcta acagattata aacgtccgaa tgtcagtatt attcactatt ttcatggaac

13261 tcattgcaat gaccaaggga aagttttgcc atatgcggaa tttgtctttt caagattaga

13321 gcactttgat ctatttgtat ttcctacttt ctctcaatgc tatcatgctc aacaaatgtt

13381 ccctcattat tctggttttg cctatgtacc gcatgcatta cctattttaa ctaatcagaa

13441 aaatgaagtg aaaagagaaa gaaacacttt tgtagttgct tcaagattag aagctattaa

13501 gcgattagat catgcagttt tagctattgg agctgcgaat aaaacttcag acggattaaa

13561 attacatttc ttggggcacg gaagtttaat agattcctta cagcaacaat caaaaatgct

13621 aggagatatt gctatatttc atggccattg ttccaatgtt cccgataagc ttcaaaaatt

13681 cagtttttat ttactcacta gtatctctga aggcttacct gtagcattac ttgaagcaat

13741 gcaagctggg tgcttaccaa tcgcttataa tattaatttt ggtccgagtg atgtaattat

13801 tcacggggta aatggttggc ttgttgaacc aggcgatgtt caggcattaa caaagactat

13861 tgagatggtt agtaaaatac ctgaaaacga attagagtat atgcgagaaa atgctcgtaa

13921 tactgctaaa caatattcta ttagccgtgt attatctgat tggaagatgt gtaaacttca

13981 agctagaaaa cgcagagctg aaaaattatc tttagaaata tggaaaaata agctaactac

14041 cgaattgaaa gataaaatta gaaaggaaat gttaaatgaa accaactaat ataattgaaa

14101 aattatcgat aagaaataac actttttta tcgaaggctt agcttatatc caaggatata

14161 actcaccaga gttcagcaat ctttataaat acttatgtat aataaatact cacactgagg

14221 aattattgga atatgaatta ggtagcatta gacggtatga gttaaagaat caaaactata

14281 aaggaaatat ctatgattat actgcatcag gaactgcaac taaaaatttt aagggaatca

14341 agataaataa tcttaaagaa ggactatatg aaataaaaat ctctgtttca gataataata

14401 ttgaacgcaa ctatcaaagt ataaacttta attcatctga aatagataaa tttgactttg

14461 atgattattt tgaatatcgc ttatttaaaa atcaaaataa gatttattta gcaaaaagaa

14521 agctaatagg aagagctcct atttccgatt attttattaa tattgaaaaa gaatggacta

14581 aaaagcaaac aatgcatatt gaaggtgcat ttgtaatccc tggtattgat attactgaat

14641 ttaatcaagc cagatactat ttaattgccc aaaaagcgat tacacagaag caatactcct

14701 ttgcattagg acagatcaaa aaagctggac taggagaaaa aatcaataat ctacaaggtt

14761 cttataatgc ctgctattat gcaacaaaaa tgctaaaagg tattgatatg agtgcgctag

14821 aattcggatt ctatgattta tatatttctt taagctacaa aagcgaagtc tttactgtta

14881 aattaaataa acagttagaa attggacatc aactcctgaa attagttgat aacgttgagg

14941 agtaggctct tatcttaacg aaagatagtt attactcttc ctaagagact tcccaaaaaa

15001 gcggtcaaat ttttctggca tagtagacaa aaaggttggt attttgtcga actatgcctc

15061 tattggacaa aagggttggt aatgattatg tttagaaatt tttaccgact ctttttattt

15121 aaaaagtcct gtataaacaa tatcttatgc tttctcgtta atccgctatg tgggcgtaat

15181 ttgtctttta attctttaaa taatccttct attcgatttg tcgtcttttc aatatttaac

15241 tcaggatact tttcatacgt aaaaatataa tccatataac gttttatact tgtataagca

15301 cttctcacat tacgatgttt ataaggatat tttccttttt cattctgttt atctgaccgt

15361 tcctctaaaa aggctttatg ttttattttc cagtgatgta tttttaaata aaattcattt

15421 tttgaactga cttttaacgt ctttattatc gtttttaatt ctcttcctgc gtgcgattga

15481 tgcttttttc ttaatgctct catcacgatt gctactaaat gaaattggca catttgcgtg

15541 ggcgtattca ttaaatcttt caataagcca cgcctgccat cacaggtaat tgattgaatg

15601 atatagcctt tttctcttaa atgattgatg gctttcttgt aataaatatc tttttccgtt

15661 ttcacaattt ggtgataaat cactttggtt gaaaggctat caataaaaac catcacgcca

15721 aaatcacgat gaaagaaagt agtatcaata attaagttta aatatttatt ttgaggggct

15781 tttaagaagg ctttaggagc cttttctaaa tatcgttgaa tcgttcttac tgaacactga

15841 tatttattag cgagttgttg atatgttcgt tttccaacag aataatccaa ccaaatttgg

15901 gtcgaattta atttagatgt taaggtaaat attttattgc aagctgaaca tttataacgt

15961 tgaagcttat ttttaaagcc atacttttgt gttttggaag attgacaaaa agggcaagtt

16021 tttattcata tttcaaaaag gtaggttaaa gccttgtact ataaggcttt aacccacttt

16081 ttaccaacct ttttgtctac tatgccattt ttctaaaaaa ttgcaaagtt taggaaaaat

16141 aagaccgctt attttattca tactcttata taaaattaga gtttattatg ggcgtagcca

16201 cgtacaatgg taaggatatg ctgtatagca atattaatac aatcctatca atttcatatt

16261 atcttaatca gatttatttt ttcgtttttt agacattaag ctgttatttt cttcgttatt

16321 taattctgcc aaatctcccc tatcccctct ttactaaaga gggggatttc ctttagatat

16381 taacaagcta tgatctttcg aatagctttg gtctctcaat agattcaggt gaatgatcga

16441 tatataagga aaataaaaac attcgtcaaa gacgagcgcc atcgtaaaga aatttggagc

16501 cacagcatgg ctcactcagc cgtaggctga tcgtaaccac gtacggcttg ccgtgcgtgg

16561 ctgttgggtg attatttaaa aacaggcgaa acggcttttt tactttttca ttaccgcaat

16621 ttgttttaga atcaacggac gatttactct caaggattta tgatgaaaaa attattttta

16681 gcggcgttaa tcgcatcgtt cgggttggct gcctgcggtg taaaaggtcc gctttatttt

16741 cccgagcagc aaccggctca acaacaaaca aaataattgc taaccacggt aattcaaaat

16801 cacacgtata acaagcggtc gaatttattc gattttttgt aaatacaagt gtagcgagtt

16861 tgccgtggtt ttattttttc aaacacaacg gacaaattaa tgaatcattt caattataaa

16921 aaccaacaac tttttgcgga agacgtttcc gtttcagata tcatcaatca atacggtaca

16981 ccggcttata tctattctcg tgctacgctt gagcgtcatt ggcacgcttt tgataaagca

17041 ttcggcgcac acccgcactt gatttgcttt gcggtaaaat ccaattccaa tatcgcttta

17101 ttaaatgtaa tggtgcgcct cggttcgggc tttgatattg tgtcgcaagg cgaacttgaa

17161 cgtgtacttg ccgccggcgg cgagccgagc aaagtggtat tttccggtgt ggcaaaatca

17221 catagcgaaa ttcaacgtgc attggaagtc ggcattcgtt gttttaatat cgaatccatc

17281 gccgagttac accgcattaa tgaagtcgcc ggtcaattag gtaaaatcgc accgatttca

17341 ttgcgtgtaa atccggatgt ggatgcgcac actcaccctt atatttccac cggtttaaaa

17401 gaaaataaat ttggggtaag cgtaacgaac gagggaaaca agtactggga gaagcgttac

17461 gagaaaaaac acaggcggaa aacctactgc gtgaaagtgc gctaaattgg acgattctcc

17521 gcccgtgcgg gctgaataca acagaaggcg aaaccttccg tttaattgaa aatgcggctg

17581 aactgcccgg cagttatatg agccgtaaag cattagccaa tgcggtcttg tccgtactta

17641 acagtgaaaa cacaaaccat aaaatcttct cagtctgtgc ctaacttcac aatccctttc

17701 actttggcac aagccatccg cttgtgctta tttcaaaatt tgttttaaca gttccaataa

17761 aaccggtaat tccgttactc cctctcgatc gataaaatgc ccgcctgttg ccaagcgaat

17821 ataatccgct tgtaagtatt gcgctaatcg atcgctgaac gaatggggaa cgacaacatc

17881 atttaatgcg gcaatcacat aagacttttc tggtaagcaa gcggtctgat ttgcataaaa

17941 atttgcaaac ttatccaatt ccggtaaatg cggtaattgt tcataaaaac cggaaacaaa

18001 aatcgccgtt tttacttttt gttgcgtcac cgccagataa ttcagtaacg caatacaacc

18061 caaactatgc ccgatcaaca ccgtctcttc atttaatttc agtgtatttt tatgatgttc

18121 cagccacgct tgcggattcg gttgatcgga attcggcatc gctaaacatt cacattccca

18181 tcctaatttt tccaattcgt ttttaagcca cggaaaccaa tttcttgtcg ggttcgccgt

18241 ataaccgtgc gttacatata ctttttcat

//

LOCUS **CP001091.1** 16427 bp DNA linear BCT 20-APR-2021

DEFINITION Actinobacillus pleuropneumoniae serovar 7 str. AP76
capsular polysaccharide gene locus, complete sequence.

ACCESSION CP001091 REGION: complement(1825226..1841652)

VERSION CP001091.1

KEYWORDS .

SOURCE Actinobacillus pleuropneumoniae serovar 7 str. AP76

ORGANISM Actinobacillus pleuropneumoniae serovar 7 str. AP76
Bacteria; Proteobacteria; Gammaproteobacteria; Pasteurellales;
Pasteurellaceae; Actinobacillus.

REFERENCE 1 (bases 1 to 16427)

AUTHORS Buettner,F., Martinez-Arias,R., Goesmann,A., Baltes,N.,
Tegetmeyer,H., Singh,M. and Gerlach,G.F.

TITLE Genome and proteome analysis of A. pleuropneumoniae serotype 7

JOURNAL Unpublished

REFERENCE 2 (bases 1 to 16427)

AUTHORS Linke,B.

TITLE Direct Submission

JOURNAL Submitted (21-MAY-2008) Centrum fuer Biotechnologie, Universitaet

Bielefeld, Universitaetsstrasse 27, Bielefeld 33615, Germany

FEATURES Location/Qualifiers

source 1..16427

/organism="Actinobacillus pleuropneumoniae serovar 7 str.

AP76"

/mol_type="genomic DNA"

/strain="AP76"

/serovar="7"

/note="K locus: KL07-I"

/db_xref="taxon:537457"

CDS 1..1473

/gene="modF"

/locus_tag="APP7_1648"

/codon_start=1

/transl_table=11

/product="putative molybdenum transport ATP-binding

protein"

/protein_id="ACE62300.1"

/translation="MPNINIQNALFSLAQHNKLSIESLEINTHDFWVIVGGNGSGKTA

FAQALHNSLSLYSGEYQNSFQHIALLSFEQQQKIIEQIFKHRNNDMISPDDFGLTARQ

IILNGSEKTQLCEEYAAKLRIQPLLDRPFIQLSTGESRKVLFCQMLVSEPDLLILDEP

FEGLDQASVAYWQDVMAQLGKQMAVVLISNRFNDIPDCATHIALLDNLQLILQGERQE

IEQQAVYSQLKFAEQNVNAPLPESATPLIQLPPNTNPFELKNVMIRYGEKTIIDDLTW

TVAPKQHWWIKGPNGAGKSTLLSIIAGDHPQSYANYVHLFGRQRGSGETIWDIKKNIG

YVSSQLHMDYRVNCSALDVILSGFFDSIGVYQQVPSALQLKAMEWLERLHLANLAKKP

FRSLSWGQQRLLLITRAMVKHPPILILDEPLQGLDGVNRKLVKQFIEQLVTNSQTQLL

FVSHQDADAPNCITHLFEFVPQENGGYRYVQTALNKTENT"

CDS complement(1822..2472)

/gene="cpxA"

/locus_tag="APP7_1647"

/EC_number="3.6.3.38"

/codon_start=1

/transl_table=11

/product="ATP-binding protein"

/protein_id="ACE62299.1"

/translation="MISVKNVSKDYYTRSGKKTVLQDINFELKKGEKIGILGRNGAGK

STLIRLLSGVEPPTSGTIERHMSISWPLAFSGAFQGSLTGMDNLRFICRIYNADIDYV

KAFTEEFSELGDYLYEPVKKYSSGMKARLAFALSLSVEFDCYLIDEVIAVGDSRFAAK

CKHELFEKRKDRSIILVSHSPSAMKSYCDNAMVLDKGIMYKFENMDEAYKFYNSTL"

CDS complement(2469..3266)

/gene="cpxB"

/locus_tag="APP7_1646"

/codon_start=1

/transl_table=11

/product="capsule polysaccharide export inner-membrane

protein"

/protein_id="ACE62298.1"

/translation="MQYGDQTTFRQSLAIQGRVIGALLMREIITRYGRKNLGFLWLFV

EPLLLTLFIVLMWKFIRADRVSDLNIIAFVITGYPMAMMWRNASNRTIGAISGNLSLL

YHRNVRVLDTLLARVLLEVAGATIAQIIIMALLILLGWIDMPKDTFYMIIAWVLMAFF

ALGLGLIICSIAQKFEAFGKIWGTLSFVLLPLSGAFFFVHALPSQAQQYATLIPMIHG

TEMFRHGYFGDSVITYESISYLVICDVAMLLFGLIMVKNFSKGIEPQ"

CDS complement(3266..4423)

/gene="cpxC"

/locus_tag="APP7_1645"

/codon_start=1

/transl_table=11

/product="capsule polysaccharide export inner-membrane

protein"

/protein_id="ACE62297.1"

/translation="METPIATSPAEKLQKPVKQKKSRFKKLNPLFWITVAIPTVLSAF

YFGSVASDIYISESSFVVRSPKNQTALTGVGALLQGSGFSRSQDDTYTVQEYMRSRTA

LEQLMQGLPVREYYENQGDIIARFNGFGLNNSKEAFYKYFRDRLSVDFDSVSGIASLR

IRAFNAEEGQQINQKLLAEGETLINRLNERARKDTISFAEQAVTEAENNVNETANALS

KYRIKNKIFDLPAQSGVQLSLISSLKSELIRVETQLAQLQSITPDNPQVDALLMRQKS

LRKEIDEQSKQLSSNSNSSIAIQTADYQRLVLANELAQQQLTAALTSLQNTKNEADRQ

QLYLEVISQPSKPDWAEEPYRLYNILATFFIGLMLYGVLSLLIASVREHKN"

CDS complement(4449..5636)

/gene="cpxD"

/locus_tag="APP7_1644"

/codon_start=1

/transl_table=11

/product="capsule polysaccharide export protein"
/protein_id="ACE62296.1"
/translation="MEIKKYNSIIGLALTTLFLSACSSLPTSGPSHSAILEANSQSSD
KPLPEVNVVELDNGLVQQLYQTQQSQQFSGFLGTVGSAGYAGAVNVGDVLEISIWEAP
PAVLFGGTFSSEGQGSGHLTQLPAQMVNQNGTVTVPFVGNIRVAGKTPETIQSQIVGA
LQRKANQPQALVKIANNNSADVTVIRQGNSIRMPLTANNERVLDAVAAVGGTTENIED
VTVKLTRGSEVKTLAFETLISDPAQNIMLRAGDVVSLLNTPYSFTGLGAVGNNQQMKF
SSKGITLAEAIGKMGGLIDTRSDPRGVFVFRHVPFAQLSLEQQAQWQAKGYAIGMDVP
TVYRVNLLEPQSMFLLQRFPMQDKDIVYVSNAPLSEFQKFLRMIFSITSPVTSTTNAV
RAY"

CDS 5833..6807
/locus_tag="APP7_1643"
/function="Transposase and inactivated derivatives, IS30
family"
/codon_start=1
/transl_table=11
/product="Transposase for insertion sequence element
ISApl1"
/protein_id="ACE62295.1"
/translation="MMSTSYRHLTINEREKIMILLAQGKKQAEIAKALGRSSSTISRE
LKRHALESYSATNAQNSYLKHRQNSKAQRKLEQPEYFNLVQEKFLTENWSPEQISARL
KLEKSELSISYSTIYRGIYSGLFDIGERKASRKLRHKGKTRHTKNHHEKRGKIQISNH
LNDRPISAQNRSRFGHWEADTVLGKAGGACLLTLTERKSRFELVKKIPAKKAEAVQKA
MIELLDSHILRSITPDRGKEFAQHRLVTEALGVEFYFPEPHQPWTRGTNENTNGLLRE
YFPKHQDINQWSEVDIQQVINKLNLRPRKCLGWKTPYEVYFKKSLHLV"

CDS 6887..8029
/gene="cps7A"
/locus_tag="APP7_1642"
/function="Putative glycosyl/glycerophosphate transferases
involved in teichoic acid biosynthesis
TagF/TagB/EpsJ/RodC"
/codon_start=1
/transl_table=11
/product="Glycerophosphotransferase"
/protein_id="ACE62294.1"
/translation="MLMKIAFIAWNSFQVLHFKPLLQALPCALLIIEKRRRSVPICKD
ILRDINNNIAYIRHTDIYAKIDGNFDVLVAQTTFEQLYLFHRTKIALLQYGYAKEPYN
YGTWRAFADLNLVYGNYAYERISYFSPTKITGCPRYDLWYQPLFHQKAKENYARVLDT
SKKTIVYAPSWGELSSFKLYIEEITKLSLFYNVLVKLHHNTLLLANKHQNYEKLYPNL

HFFYEGEDLLSLISVADIVISDFSGAIFDAIFCKKTVVLLSISLVNQPKLDKFSLEIA

YRSKLGYEVFSPDQVAITVARALTEPKLVDETLYQQLFMHNKDATQQVINALQQLAEG

KYTLSQQQLYVRQTEKLLNIEKIKQQKNKKQSFNKIRQISKRLIKK"

CDS 8082..8510

/gene="cps7B"

/locus_tag="APP7_1641"

/function="Cytidylyltransferase"

/codon_start=1

/transl_table=11

/product="glycerol-3-phosphate cytidylyltransferase"

/protein_id="ACE62293.1"

/translation="MKKVLTYGTFDLLHHGHIRLLERARSLGDHLTVAISTDQFNLGK

GKVCAYTYEERAHILKAIRYVDEVIPETNWEQKVEDVKNHEIDVFVMGDDWEGKFDFL

ADYCEVVYLPRTPDISTTQVKKMLAKKDLAAGQKQIHEKE"

CDS 8518..9654

/gene="cps7C"

/locus_tag="APP7_1640"

/codon_start=1

/transl_table=11

/product="conserved hypothetical protein"

/protein_id="ACE62292.1"

/translation="MFQILQKHLPTLQRVLREGYSQHSLLAYWYGLSLLTALEQANHP

QVRKLAEKMINKGINIGHYFLAQSYFLCGEYDLAEQAVKKIKNFVKIPEVVFLYADIL

VKCKRKEEAWQLLEQCALLNKRKKVWIHLTNLVNTEADYRHLEQHIDKVRTTTPYLKS

DLLIHQRTNAALRAGLTETALALTELNPLPKQAKVKKKTTAYNDKLAAIALADLKKVL

DHKKIPFFLISGTLLGCIREGKLLGHDKDIDVGVWDEYSYEELANCLSTSGYFYVVPT

RTKHLVMLRHVNGIAIDVFIHYREPNDYWHAGVKIKWHNSPFNLVYTNFLGQQYLIPE

NYDLYLTENYGDWRTPKTKFDSAFDTPNMEVINEAEMKIYIFRK"

CDS 9666..13499

/gene="cps7D"

/locus_tag="APP7_1639"

/function="Putative glycosyl/glycerophosphate transferases

involved in teichoic acid biosynthesis

TagF/TagB/EpsJ/RodC"

/codon_start=1

/transl_table=11

/product="Putative CDP-glycerol:glycerophosphate"

/protein_id="ACE62291.1"

/translation="MKKKFYKAIYLFNAPIIYWRGLKYYQKKEWKKAEKYFQLAVTRK

PDHAYSNFKLGMCFFKQKMWDQAYNYISIAVNLAPEMPLWKVQLRQSAAQLQVKKRRK
SVKRNSENQISTPEELKKVTGISANEVAEQILIELLEKEPENPTIYAELALIQSKQAK
LWQAVDSWTEAINRKSDNAMYFYQYGIVLEKLGHYARASEAYKTSLNLNPPNKILADL
FFRLGFVNKHQGHDNILNIELADEAYTKAIQADKKLKSKDFGIGVFYEERRDWQQAAI
AYENQIGKTIKEAELFYRIGFAYDRNYEWELAEINYQKALSITERPEWRFRLGLVLEK
QKKFIQATKNYEKAAIERKQYTPYWFYRCAYTLEQQGLYERASKMYLKLRKDPSLITK
ASHSSVNDLILALNIKHNYDTTSAHSWFELGSSYELIQNWEKSEYAYSQAIARSNELV
SLWYYRLGFVQIAQGKYLQACESLRSYRVMQRPHGVNEDILSKDISYAEVATYNEYYN
ILNIKEQTVLYESFSGQGMSCNPLALFLYLFNHNEYKNWTHIWVINDTSNIPEEYRKY
DNVIFIRRGSDSYLRYLATTKILINNSNFPPYFIRKPEQKFLSTWHGTPFKTLGRDME
GRFFEHKNLTRNIFQSTHLLSPNAHTSKILYERHDIKEIYTGRLIESGYPRIDMTLSL
AKEEKIELREKLGVLNNEKLVFYAPTWRGIHGDIEFDYEKLQSDLNKLSKLEGAKVVF
RGHSLLQEALSKINLGITVVPDELDTNKILSVTDILITDYSSVLFDYLPTLKPLVLYM
YDIKEYTEERGLYFSENELPGEKCYNINELVKTLTYLLENNITSVSFEDSKVAQFAPH
DDGNVSEKVINALFLDDYTDLKVINDIPENKKSLLIYGGPFMGNGITTSVINLISNID
RSKYTVTLVIDPGSIEKEAGRLRQFEKLPKDINVVARVGRMNMDLEERYIHGLNNQHY
ELQSSVARGILQDSWEKEYQRIFGNAKFDSLIQFEGYNRFWSGVFTSIQNKKSSIYMH
NSMEEEYRLKYPYLKSMFYYCSLANKVISVSELTMELNKDKLADKFGILSSKFDYSDN
LQQPEKIRKLADEPLLLDDEIYFKTPGKVFLTIGRLSIEKDHAKLINSFAKLIKYYPD
SKLLIIGDGSLKYALTQQIKELKLDNNVYLLGLRTNPFPLLKNADCFILPSNHEGQPM
TLFEAMILGKMIIATDIVGSRSALEGRSGYLVENSVDGLLKGMSDFLEGKLSLITFDI
NEYQEQAINRFYNVI"

CDS 13534..15147

/gene="cps7E"

/locus_tag="APP7_1638"

/codon_start=1

/transl_table=11

/product="hypothetical protein"

/protein_id="ACE62290.1"

/translation="MNIITISPNSLVIPENFDLRRINARPDEFIDPTYMDKYDNTSIF
YDIFESNNKIYLIGPPLLNLSPIINSCYIIFDNGREEKVSINSKLLERGQLSWIDLKE
IKYKPVSLRFDFSIFSISYKGNKNVIVDIGKDVNDEFNDAKSLMTLQLNNKLEWIHDW
AKYYNKVHDVDTIVIYDNNSTNYKLDDISNSLLSITNLKNIVVVPWNFKYGPQGKPWT
GPNTPWDSDFCQIGALQHMRFRFSLKSKGFINADIDELIIPLKEVNIFDALENSEVGV
IGVEGNTIEGHLSNHMMKAEGVPHFYHFWERKVHISGGTRKWAGSPSKWDDETVQTTA
HWVRGISYKADSRFSIGHFRQINDGWKIQSRTIEYSGKDILRPDFSLIGAMSVAFPNE
IPNILLVNALKDAEQRIQLLEKGKEDEYSKLQSYIKLLTHERIVWDKIWIWKGNVLVF
ETRCSLGKIAFDIVISNNNVQLNVSVRDTKYQEDFFEVVFRYLGTDFSILSNGKGLKA
YSLKRENISFEEIATLISKKILIFYKILN"

CDS 15259..15813

/locus_tag="APP7_1637"

/codon_start=1

/transl_table=11

/product="hypothetical protein"

/protein_id="ACE62289.1"

/translation="MSKKITLLSLAVFIAGCSSAPQPEAFPGEFANADYVLSDKDAQR

WVVASHQAEQCIYPNLTRIQQQAFSKEDSYIHSQYVFFYPLEEIIGEQYVKIIQDDEK

SMGYAQYLFKKFRDNQEFEPLADKQCLVLREKAKNDLAVVKGQYKSEMVEETKSEAKN

ADGVATNQNKFFFDIIKWGSMLLL"

CDS complement(15885..16427)

/gene="ydeN"

/locus_tag="APP7_1636"

/codon_start=1

/transl_table=11

/product="hypothetical protein"

/protein_id="ACE62288.1"

/translation="MKKVYVTHGYTANPTRNWFPWLKNELEKLGWECECLAMPNSDQP

NPQAWLEHHQNTLQLDENTLLIGHSLGCIALLNYLAVTQQKVKTAIFVSGFYEKLPTL

PELDSFADFYANQTACLPQKSYVISALNDVVVPHSFSDRLAQYLQADYIRLATGGHFI

DREGVTELPELLELIKQISN"

ORIGIN

1 atgccaaata tcaacatcca gaacgcctta ttttcccttg ctcaacacaa taaactgtcg

61 attgaatcgc tggaaatcaa tactcacgat ttctgggtga ttgtcggcgg taacggctcg

121 ggtaaaactg ctttcgccca agcgctacat aattcacttt cactatattc gggcgaatat

181 caaaacagct tccaacatat cgctttactt tccttcgagc agcaacaaaa aatcatcgag

241 caaatcttta aacaccgtaa caacgatatg atttcaccgg atgatttcgg tttaaccgcc

301 cgtcaaatta tcttaaacgg tagcgaaaaa acgcaattat gcgaagaata tgcagctaaa

361 ttacgtattc agccgttatt agatcgcccg tttattcagc tatccaccgg cgaaagtcgc

421 aaagtgctat tttgccaaat gttagttagc gaaccggatt tgctgatttt agatgagcct

481 tttgaggggt tagaccaagc ctcggtcgct tattggcaag acgtgatggc acaactcggc

541 aagcaaatgg cggtggtact gatttccaac cgttttaatg atattcccga ctgtgccaca

601 catattgctt tactggataa cttacaactg attttacaag gcgaacgcca agagattgaa

661 caacaagcgg tctattctca gctaaaattt gcagaacaga atgtgaatgc accgttgccg

721 gagagtgcca caccgctgat tcaactccca ccgaatacta atccgtttga actgaaaaac

781 gtaatgatcc gttacggcga aaaaacgatt attgatgatc taacttggac ggttgcccca

841 aaacaacatt ggtggattaa aggcccgaac ggagcaggaa aatcgacctt actttctatt

901 attgccggcg atcatccgca atcttacgct aattatgtgc atttattcgg tcgtcagcgt

961 ggttcgggcg aaacgatttg ggatataaag aaaaatatcg gctatgtgag cagccaatta

1021 catatggatt atcgggtgaa ttgctctgcg ttagacgtga ttttatctgg cttttttgat

1081 tcaatcggcg tttatcaaca agtaccgagt gccttacagc taaaagcaat ggaatggctg

1141 gaacgcttgc atttagccaa tctggcgaaa aaaccgttcc gttcactttc gtgggggcaa

1201 caacggttat tattgattac tcgtgccatg gtaaaacatc cgccgattct gattttagac

1261 gaaccgctgc aaggcttgga cggtgtaaac cgcaaattgg ttaaacagtt tatcgagcag

1321 ttggtcacta acagccaaac ccagttgcta tttgtttcgc accaagatgc ggacgcccca

1381 aattgcatca cccatttatt tgaatttgtc ccgcaagaga atggtggtta tcgttatgta

1441 cagacggctt tgaataaaac agaaaacacc tgagttcaac cacggaaaac acggattgca

1501 cggagtttaa tgaatcggtc agttgacgta atgtttggat aatattgttt ttttcaggtt

1561 tttcttatct cgtaaggaac taataaaact ttaatcggaa actgcaacta ctcgcaatat

1621 cataatttgt taacctttaa aggaaatccc cctctttagt aaagaggggt taggggagat

1681 ttgtcaatag agatatgaaa ttgaatagaa cttcattttt tatatttata aaagcgttaa

1741 ttagcatatt tcttcgctaa ttcattctgt caaatctcct cctgcccctc tttgctaaag

1801 aggggagata tgtgtgggac tttaaagcgt tgaattatag aacttataag cctcgtccat

1861 attctcaaat ttatacataa tccctttatc taataccatt gcattatcgc aataagactt

1921 cattgctgac ggactatgcg aaaccaaaat aatcgaacga tctttgcgct tttcaaataa

1981 ttcatgttta cattttgccg caaagcgaga gtcacctacc gcaattacct catcaattaa

2041 gtagcaatca aactcaactg aaagcgataa agcaaacgca agtcgggctt tcataccgga

2101 agaatatttc ttcaccggct catataaata atcgcctaat tcggaaaact cttcggtaaa

2161 tgctttaaca tagtcaatat ccgcattata gatacggcaa ataaagcgta aattatccat

2221 accggttaaa ctgccttgaa acgccccgct gaaagcgagc ggccaagata tcgacatgtg

2281 acgttcgata gtacctgatg ttggcggttc aacaccactt aacaaacgga ttagcgttga

2341 tttccctgca ccgttacgcc ctaaaatacc gattttctcg ccttttttca gctcaaaatt

2401 aatatcttgc aatacggttt ttttaccgct tcgagtatag taatctttac tcacattttt

2461 tacgctaatc attgcggttc gattccttta ctgaagtttt ttaccataat gagcccaaaa

2521 agtaacatgg ctacatcaca tatgacgaga taacttatac tttcatatgt gataacactg

2581 tcgccaaaat aaccgtgacg aaacatttcc gtgccgtgaa tcatcggtat taaggttgca

2641 tattgttgag cttggcttgg tagcgcatgc acaaagaaaa atgcgcctga aagaggtaaa

2701 agaacaaagc ttaatgttcc ccagattttg ccaaatgctt caaatttttg tgcaatggag

2761 caaatgatca agccgagccc taaagcaaag aatgccatta atacccatgc tattatcata

2821 tagaacgtat ctttcggcat atcaatccaa cccaataata ttaataatgc cataataatg

2881 atttgggcaa ttgttgcgcc tgctacttcc agtaggactc gagcgagtaa agtatccaat

2941 acacgtacgt tacgatgata aagaagactc aaatttcctg atattgctcc gatagttcta

3001 tttgaagcat tacgccacat cattgccatt ggataaccgg taatcacaaa agcaataata

3061 tttaaatcgg aaacgcgatc cgctcggata aatttccaca tcaaaacgat aaataaagtg

3121 agtaatagcg gctcaacaaa cagccataaa aaacccaaat tttttcgtcc gtaacgcgta

3181 ataatttccc gcatgagtaa tgcaccgatt actctccctt gaatggcgag agattggcgg

3241 aaagttgttt gatcaccgta ttgcattagt ttttgtgctc tcttacgctt gcaattaata

3301 aacttaatac accataaagc atcagaccga taaagaatgt cgctaaaata ttatataagc

3361 gataaggctc ttccgcccag tccggtttgc ttggctgact gattacttct aaataaagtt

3421 gctggcgatc cgcttcattt ttcgtatttt gtaatgaggt taatgctgcg gtcaattgtt

3481 gctgtgccag ctcgtttgca agtactaagc gttggtaatc ggcagtttga atagcaatag

3541 agctattact gttactggaa agctgttttg attgctcatc gatttcctta cgtaaacttt

3601 tttggcgcat aagcaatgca tcaacttgcg ggttatccgg cgtaatcgat tgtaattgcg

3661 ctaattgggt ttccacacga atcaactcgc tttttaagct tgaaattaat gaaagttgta

3721 cgcccgattg tgccggtaaa tcaaaaattt tatttttgat acggtattta cttaaagcat

3781 ttgctgtttc gtttacatta ttttccgctt ctgtaaccgc ttgttccgca aatgaaatgg

3841 tatcttttct tgcacgttcg tttaaacggt taataagcgt ttcaccttcg gcaaggagtt

3901 tttgattaat ttgttgcccc tcttccgcgt taaatgctcg aatacgtaag ctggcgatac

3961 cggaaacaga gtcaaagtcc acacttaagc gatctcggaa atatttataa aacgcttctt

4021 tactgttatt taatccgaat ccattaaagc gagcgataat atcgccctga ttttcataat

4081 attcgcgaac aggtaatcct tgcattagtt gctcaagagc ggtacgagaa cgcatatatt

4141 cctgtacggt ataagtatcg tcttgcgaac gagaaaaacc tgaaccttgc aataaggcgc

4201 ctacgcctgt cagagcggtc tgatttttag gcgatctaac gacaaaactt gattccgaaa

4261 tataaatatc agaagcgacc gaaccgaaat aaaacgctga tagcacggta gggattgcga

4321 cagttatcca aaataatgga ttgagttttt taaagcggct ttttttctgt ttaaccggtt

4381 tctgtaattt ttctgctgga ctggtagcaa taggtgtttc catcttttgt ccttatacat

4441 tcaatatatt aataggcacg aacggcattg gtcgtactgg taaccggcga agtaattgag

4501 aaaatcattc tcaagaattt ttggaactca gacaacggcg catttgaaac atacacaata

4561 tctttatctt gcattgggaa acgctgtaat aaaaacatgg attgcggctc aagtaagttc

4621 acacgataaa ccgttggtac atccattcct atagcgtagc ctttagcttg ccattgtgct

4681 tgttgttcta aactcaattg tgcaaaaggc acgtgacgga atacgaaaac ccctctcgga

4741 tccgaacgag tatcaattag accccccatc ttaccgatag cttcggcaag cgtaattcct

4801 ttacttgaga atttcatttg ctggttattg cccaccgcac ctaaaccggt aaaactataa

4861 ggtgtgttta gcagtgaaac aacatcgccg gcacgtaaca taatattttg tgccggatcg

4921 gaaattaacg tttcgaatgc gagtgttttt acttcagaac cacgggttag cttgaccgtc

4981 acatcttcaa tgttttccgt tgttccgcct actgcagcaa ccgcatctaa tacacgttca

5041 ttattagcgg ttaatggcat acgaatacta ttgccttgac gaataaccgt aacatcagca

5101 gagttattat tcgcaatttt gactaatgct tgcggctgat tcgctttacg ctgcaatgcc

5161 ccaacaattt gagactgaat cgtttcaggt gttttacctg cgacacgaat atttcccaca

5221 aacggcacgg ttaccgtccc gttttgatta accatttgtg ccggtaattg cgttaaatgc

5281 ccgctacctt gtccttcgga actaaaagta ccgccaaaca acactgccgg cggcgcttcc

5341 caaattgaaa tttcaagtac atcacccaca ttgactgcac cggcatagcc cgcgctgcct

5401 actgtgccta aaaatccgga aaattgttgg ctttgctgag tttgatacaa ctgttgaact

5461 aaaccgttat ccagttccac cacatttact tccggtaagg gtttatccga actttgtgaa

5521 ttagcctcta agatcgcact atggctaggg cctgaagttg ggaggcttga gcaagcagaa

5581 aggaacaatg ttgttaaagc taaccctatg attgaattat attttttaat ttccatcttg

5641 taaaaaggct ctattgaaaa agtgtgtaaa atgaatataa actatacata attatagata

5701 caacactata attatatatt ataatctaat atctcgtttt ttaaaagctg aatttacaat

5761 ccaagtgcaa caaaaaaaga agtactcatc aactagaata taattttgtt tccacacaaa

5821 aaccacttcc aaatgatgag tacttcctac cgacatctta caataaacga gcgagaaaag

5881 ataatgattt tactcgcaca gggcaaaaaa caagcagaaa ttgccaaagc actgggacgt

5941 agctccagca ccatttctcg cgagctgaaa cgacacgctc tagaaagcta cagtgcaacg

6001 aacgcacaaa acagctattt gaagcatcgt caaaatagca aagcacagcg caaattagag

6061 cagcctgaat atttcaattt ggtgcaagaa aagtttctga cagaaaactg gtcgcccgaa

6121 caaatcagcg cacgattaaa attggaaaaa tctgaattat ccattagtta ttcaaccatt

6181 tatcgtggta tttattcagg gttgtttgat ataggcgaac gcaaagccag tcgcaaactg

6241 cgccacaaag gcaaaacacg gcatacaaaa aatcatcatg aaaaacgtgg caaaattcag

6301 atatccaacc atttgaacga ccgtcccatt tcggcgcaaa atcgcagtcg ctttggacat

6361 tgggaagccg ataccgtact gggtaaagcg ggtggagctt gtttgctgac gctgacggaa

6421 cgcaaaagtc gttttgagtt ggtgaagaaa attcctgcca aaaaagccga agcagtccaa

6481 aaagccatga ttgaattgct ggattcacat atattgcggt caattacgcc agaccgtggt

6541 aaagaatttg cccaacatcg tttggtaaca gaagcactgg gtgtagaatt ttacttcccc

6601 gagccgcatc aaccgtggac acggggaacg aatgaaaata caaatgggtt acttcgtgaa

6661 tactttccga agcaccaaga catcaatcag tggagcgaag ttgatattca acaggtgatc

6721 aataaactga atttacgacc acgtaaatgt ttaggttgga aaacacctta tgaagtttac

6781 ttcaaaaaat cgttgcactt ggtttgacaa ttcaagaacg ataacctaaa atattacttt

6841 tctatagaag aataggcatt attttaataa atataattag gtatgaatgt tgatgaaaat

6901 agcatttatc gcttggaata gttttcaggt gttacatttc aagcccttat tacaagcttt

6961 accgtgtgca ttattaatta ttgaaaaacg gagacgtagt gtaccaatct gtaaggatat

7021 tttgcgagat ataaacaata atatcgctta tatccgccat acggatatat atgcaaaaat

7081 tgatgggaat tttgatgttc tagttgctca aactactttc gagcaacttt atttgtttca

7141 ccgcaccaaa attgcattgc ttcaatacgg atatgctaag gaaccgtata actacggcac

7201 ttggagagca tttgcagatc taaatttggt ttatgggaat tatgcctatg aacgtatttc

7261 ctatttctct ccaactaaaa taaccggttg tccacgatac gatttatggt atcagccttt

7321 atttcatcaa aaagcgaaag aaaattatgc gagagtatta gatacgagta agaaaacgat

7381 tgtatatgca ccaagttggg gagaattatc cagctttaaa ttatatatag aagaaattac

7441 gaaattatct ttattttaca acgtgttagt aaaattacac cataacacgc ttttattagc

7501 aaacaagcat cagaattatg aaaaattgta tccgaattta cattttttct atgaaggtga

7561 agatcttctc tcacttattt cggtagccga cattgttatt tccgatttta gcggtgcgat

7621 ctttgacgca attttttgta aaaaaacagt agtgttactt tcgatatcgc tagtgaatca

7681 accaaaacta gataaattta gtttagagat agcttatcgt tcaaagttag gatatgaggt

7741 tttttcgccg gatcaagtag ctataacagt ggcacgagca cttacagagc cgaaattagt

7801 agatgaaacg ctgtatcaac agctttttat gcataacaag gatgcaacac agcaagtaat

7861 aaatgcttta caacagcttg ctgagggtaa gtatacatta tctcaacagc agttatatgt

7921 gcgacaaaca gaaaaaattat taaatattga aaaaataaag cagcaaaaaa ataaaaaaca

7981 gtctttcaat aaaataagac agatttctaa aagattaatt aaaaaaataat ttttcatatt

8041 ttctttttat attttttat ttgacaatac ggagtttagg aatgaaaaaa gtattaacct

8101 atggaacctt tgatttatta caccatgggc atattcgttt attagaaaga gcaagatcat

8161 taggcgatca ccttactgtt gctatttcta ccgatcaatt taacttagga aaaggcaaag

8221 tatgcgctta tacttacgaa gagagagcgc atattttaaa agcaatccgt tatgtggatg

8281 aagtaattcc tgaaacaaac tgggagcaaa aagttgagga tgtaaaaaat cacgaaattg

8341 acgtatttgt aatgggtgat gactgggaag gcaaatttga cttcttagca gattactgcg

8401 aagtggttta tttaccgaga acccctgata tttcaacgac tcaggtaaaa aaaatgcttg

8461 cgaaaaaaga tctcgcagcc ggacaaaaac aaattcacga aaaagagtaa tttgttaatg

8521 tttcaaatcc tacaaaagca tttaccgacc ttgcaaagag ttctgaggga gggttactcc

8581 cagcattctt tgcttgctta ttggtatggg cttagtctac ttactgctct tgaacaggcg

8641 aatcatcctc aagtaagaaa actggctgag aaaatgatca ataaaggtat taatatcggg

8701 cattattttt tagcacaaag ttatttctta tgtggagaat atgatttagc ggaacaagcg

8761 gtcaaaaaaa tcaaaaattt tgtaaaaata cccgaagttg ttttttata tgcggacatt

8821 ctcgttaaat gcaaacgtaa agaagaggct tggcaattat tagaacaatg cgctttactc

8881 aataaaagaa aaaaagtgtg gatacatcta acaaatttag taaatactga ggcggattat

8941 cgacacttag aacaacatat tgacaaagta agaacaacta caccttactt gaagtctgat

9001 ttgttaattc atcaaagaac aaatgcagca ttaagggctg gtttaacaga aacggcatta

9061 gcactaacag aacttaaccc tttgccaaag caagcaaaag tgaagaaaaa aacaaccgct

9121 tataatgata aattagcggc aattgcgcta gcggatctaa agaaagtatt agatcacaaa

9181 aaaatacctt tctttctgat tagcggcacg ttgctaggtt gtattcgaga aggaaaatta

9241 ttagggcatg ataaagatat tgatgtcggc gtttgggatg agtactccta cgaagaatta

9301 gcaaactgtt tatctacatc gggatacttt tacgtagtac caactcgaac aaaacattta

9361 gtcatgttaa gacacgttaa tggtatcgca attgatgtgt ttattcatta tcgcgaacct

9421 aatgactatt ggcacgctgg tgtcaaaata aaatggcata attcaccatt taatttggta

9481 tatacaaatt tccttgggca acaatattta atacctgaaa attatgattt atacctaacg

9541 gaaaattatg gtgactggcg tacgccaaaa acaaaattcg acagtgcttt tgatactcct

9601 aatatggaag taatcaatga agctgaaatg aaaatatata tattcagaaa ataataaggt

9661 tatatatgaa gaaaaaattt tataaagcaa tttatctttt taatgctcct attatttatt

9721 ggcgaggatt gaagtattat cagaaaaaag aatggaaaaa agctgagaaa tattttcaat

9781 tagcagttac ccgtaaacct gatcatgcat atagtaattt caaactaggt atgtgctttt

9841 ttaaacagaa aatgtgggat caagcataca actatatttc tattgccgta aatcttgccc

9901 cagaaatgcc tctatggaaa gtccaattac gtcagtctgc agcccaatta caagtgaaga

9961 agcgcagaaa aagtgtgaag agaaattctg aaaatcagat aagtacacct gaagagttaa

10021 aaaaagtgac aggcatttct gctaatgagg tcgcggaaca aattttaatt gaattattag

10081 aaaaagaacc agaaaatcct acaatttatg cggaactcgc attaattcaa agtaaacagg

10141 ctaaactgtg gcaggcagta gattcttgga ctgaagctat aaatcgtaaa tctgataatg

10201 ctatgtattt ctatcagtat ggaattgttt tagaaaaatt agggcattat gctagagcat

10261 ctgaagcata taaaacatct cttaatttaa atcctccaaa taaaatactt gcagatttgt

10321 tctttcgttt aggatttgta aataagcatc aaggtcatga taatatactg aatattgaac

10381 tggcagatga agcttatact aaagctattc aggctgataa aaaacttaaa tcaaaggatt

10441 ttggtatagg agttttctat gaggaacgta gagattggca acaagcggct atagcgtatg

10501 aaaatcaaat tgggaaaaca ataaaagaag ctgagttatt ttatcgtata ggttttgcat

10561 atgatcgtaa ttatgagtgg gaattggctg aaataaatta tcaaaaggct ttatctatta

10621 cagagcgccc agaatggcgt ttccgtttag gtcttgtatt agagaaacag aagaaattta

10681 ttcaagctac aaagaattat gaaaaagcag ctatagagag aaaacaatat actccgtatt

10741 ggttctatcg ttgcgcttat acgttagagc agcaggggct atatgaaaga gcctcaaaaa

10801 tgtatttgaa attacgtaaa gatccctcat taattaccaa agcaagtcat tcatctgtaa

10861 atgatcttat tttggcatta aatataaaac ataattatga tactacgtca gctcattcat

10921 ggtttgaatt gggttcctct tatgaattaa tccagaactg ggagaagtcg gaatatgctt

10981 attctcaagc tattgctaga agtaatgagt tagtttcgct atggtattat cgtttagggt

11041 ttgttcaaat tgctcaaggt aagtatttgc aagcatgcga gagtttaaga agttatcgag

11101 tgatgcaacg tccgcatgga gtaaatgaag acattctatc aaaagatatt agctacgcag

11161 aagtagccac atataatgaa tactataata ttttaaatat aaaagaacaa actgttctat

11221 atgaaagttt ttctgggcaa ggaatgagtt gtaatccatt agccttattt ttatatctat

11281 ttaatcataa tgaatataag aattggactc atatttgggt cattaatgat actagtaata

11341 ttcctgaaga atataggaaa tatgataatg taatctttat tagaagagga agtgatagtt

11401 atttacgtta tttagcaact actaagatat taattaataa tagcaatttt cctccatatt

11461 ttattagaaa acctgaacag aagtttttaa gtacatggca cggaactcca tttaaaacat

11521 taggaagaga tatggaggga agattttttg aacataaaaa tctaaccaga aatatatttc

11581 aatctactca tttattaagt ccaaatgctc atacctcaaa gattctttat gagcgccatg

11641 atattaaaga aatctatact ggaagactaa ttgaatcagg ttatcctcga attgatatga

11701 cattgtcatt agcaaaagag gagaaaattg agttaagaga gaagttaggt gttttaaata

11761 acgaaaaatt agtattttat gcaccgacct ggagaggtat tcatggggac atagaattcg

11821 attatgagaa gttgcaatca gatttaaata aacttagcaa attagaaggt gctaaggttg

11881 tttttagagg gcattcatta cttcaggaag cattatccaa aattaatcta ggtattactg

11941 ttgttcctga tgaattagat acgaataaga ttcttagtgt aactgatata ttaattactg

12001 attattcaag tgttttattt gattatttac caacattaaa gccattagtt ctttatatgt

12061 atgatatcaa agaatataca gaagaacgag gattatactt ttcagaaaat gaattacctg

12121 gagaaaaatg ctataacata aatgaattag tgaagacatt aacgtattta ttagagaata

12181 atattacctc tgtgtcgttt gaagatagta aagtggctca atttgctcct catgatgatg

12241 gtaatgtttc agaaaaagtc attaatgctt tatttttaga tgactataca gatctaaaag

12301 ttattaatga tattccagaa aataaaaagt ctttgctcat ttatggtggt ccttttatgg

12361 gtaatggaat aactacatcg gtaattaatc ttatttctaa tattgatcgt tcaaaatata

12421 cggtaacatt agtaattgat ccaggctcta ttgagaaaga ggcaggacga ttaagacaat

12481 ttgaaaaatt accgaaagat ataaacgtag ttgctcgtgt tggacgaatg aatatggatt

12541 tagaagaaag atatattcat ggattaaata atcagcatta tgaattgcaa tcttcggtag

12601 ctcgaggtat tttacaggat tcttgggaga aggaatatca gcgcatattt ggcaatgcta

12661 aatttgattc attgattcag tttgaagggt ataaccgttt ttggtctgga gtgtttacat

12721 ctattcagaa taagaaatct agtatttata tgcataattc tatggaagaa gaatatcgac

12781 taaaatatcc atatttaaaa tcaatgtttt attattgttc tttagcaaat aaagtgatat

12841 ctgtgtcgga attaactatg gaattaaata aagataagtt agcagataag tttggtattt

12901 tatcaagtaa atttgattat agtgataatt tacagcagcc agagaaaatt aggaagctag

12961 ctgatgagcc cttgctatta gatgatgaga tatattttaa aacgccagga aaggtgtttt

13021 taacaatagg tagactatca atagagaagg accacgctaa gttaattaat agttttgcaa

13081 aacttataaa atattatccc gatagtaaat tattaattat tggagatggc tcacttaaat

13141 atgctctaac tcagcaaatt aaagagttaa aattagataa taatgtatat ttactagggt

13201 taaggacaaa tccattcccg ttattgaaga atgctgactg ttttatatta ccttcaaacc

13261 atgagggaca acctatgact ttatttgaag ctatgatttt aggaaaaatg attattgcaa

13321 cagatattgt tggatcaagg agtgcactag aaggaagatc aggttattta gttgagaatt

13381 ctgtagatgg attgttaaaa ggaatgtcag acttcctaga aggaaaatta tcattaatta

13441 cttttgatat aaacgagtat caagagcaag caattaatag attttataat gttatttaag

13501 cattgtattt taataaaatc aggagatttt aatatgaata ttattactat aagtcctaat

13561 tcgttggtta taccagaaaa ttttgattta agacggataa atgctagacc cgatgagttt

13621 atagatccta catatatgga taaatatgat aatacatcta ttttttatga tatatttgag

13681 agtaataata agatatattt aattggtcct ccattgttaa atttatcgcc tattattaat

13741 agttgctata ttattttcga taatggtaga gaagaaaaag taagcataaa ttccaagctc

13801 ttagagagag gtcaattaag ttggattgat ttaaaagaaa tcaagtataa accagtatct

13861 ttaagatttg acttctctat ttttagtatt tcttataaag gaaataagaa tgttattgtt

13921 gatataggaa aggatgttaa tgatgaattt aacgatgcaa aatctttaat gacattacag

13981 ctaaataata aattggaatg gattcatgat tgggccaaat actataataa agtacatgat

14041 gtagatacaa tagttatata tgataataat tctactaatt ataaactgga tgatatatct

14101 aattctttgt taagtataac taacttaaaa aatattgttg ttgttccttg gaattttaaa

14161 tatggtcctc aagggaaacc ttggactggg ccaaatactc cttgggactc cgatttttgt

14221 caaattgggg cattacaaca tatgagattt agattttcac taaaatctaa aggttttatt

14281 aatgctgata tagatgaact aattattcct ttgaaagaag ttaatatttt tgatgcactt

14341 gaaaatagtg aggttggagt aatcggagtt gaaggaaata caattgaagg tcatctctca

14401 aatcatatga tgaaagctga aggggttccc catttttatc atttttggga gagaaaagtc

14461 catattagcg ggggaacacg aaaatgggca ggttctccaa gtaaatggga tgatgaaaca

14521 gttcaaacaa cagctcattg ggtaagagga atttcatata aagctgatag tcgtttttca

14581 ataggacatt tccgtcaaat aaatgatgga tggaaaattc aaagtagaac gatcgaatat

14641 agtggtaaag atattttgag acctgatttt tcattgattg gagccatgtc agttgctttt

14701 cctaatgaga tacctaatat tttattagtt aatgcattaa aagatgcaga acagagaatc

14761 caattattag aaaaaggtaa agaagacgaa tattcaaaat tacaatctta cattaagtta

14821 ttgacacatg agcgtattgt ttgggataaa atttggatat ggaaagggaa tgttttagta

14881 tttgaaacaa gatgctcatt ggggaaaata gcatttgata tcgttatatc aaataataat

14941 gtccaattaa atgtttcagt aagggatact aaatatcaag aagatttctt tgaagtagta

15001 tttagatatt tgggaacgga ttttagtatt ttatctaatg gaaaaggatt gaaagcatat

15061 tctttaaaga gagaaaatat tagtttcgag gagattgcaa cattaataag taaaaaaata

15121 ttaatatttt ataaaatatt aaattaatat ttaattgagt aattagtatt ttaaaaacta

15181 actcaaatta agtaataaag ttgttatttt aatattgcta tctattatac tttctaaggt

15241 taagagtcaa aacatatcat gtccaaaaaa atcactttat tgagtcttgc cgtattcatc

15301 gcaggttgtt cctctgctcc gcagccggaa gctttcccag gggagtttgc gaatgcggat

15361 tatgtgttat cggataaaga tgcccagcgt tgggtggtgg cgagccatca ggcggagcag

15421 tgtatttatc cgaatttaac gcgtattcag cagcaagcgt ttagcaagga agattcatat

15481 attcattcgc aatacgtatt tttctatccg ctggaagaaa ttatcggcga gcagtatgta

15541 aagattatcc aagacgatga aaaatctatg ggatatgcgc aatacttgtt taagaaattc

15601 agagataatc aggaattcga gccgttagcg gataagcaat gtcttgtgtt gcgagaaaaa

15661 gcgaagaacg atttagcggt cgtaaaaggg cagtataaga gcgaaatggt tgaagaaacg

15721 aagtccgaag ctaaaaatgc ggacggcgtg gcgaccaatc aaaataaatt cttctttgat

15781 attatcaaat ggggttcgat gctattactg taatttgcgg ttagtgtgat gttaaagagt

15841 ataaaaacga gctgtcggta aatcgttatc ggcagctctt tttattagtt tgatatttgt

15901 ttgattaact ccaataactc cggtaattcc gttactccct ctcgatcgat aaaatgcccg

15961 cccgttgcca agcgaatata atccgcttgt aagtattgcg ctaatcgatc gctgaacgaa

16021 tggggaacga caacatcatt taatgcagat atgacgtaag acttttgcgg taaacaagcg

16081 gtctgatttg cataaaaatc tgcaaagcta tctaattccg gcaaagttgg taatttctca

16141 taaaagccgg aaacaaaaat tgccgttttt actttttgct gcgttaccgc aagataattc

16201 agtaacgcaa tgcagcccaa actatgtccg atgagtaagg tattttcatc taattgaagt

16261 gtattttggt gatgttccag ccatgcttgc ggattcggct gatcggaatt cggcatcgct

16321 aaacattcac attcccatcc taatttttcc aattcgtttt taagccacgg aaaccaattt

16381 cttgtcgggt tcgccgtata accgtgcgtt acatatactt ttttcat

//

LOCUS **CP001091.1b** 15355 bp DNA linear BCT 20-APR-2021

DEFINITION KL07 reference sequence for Actinobacillus pleuropneumoniae serovar 7

ACCESSION .

VERSION .

KEYWORDS .

SOURCE Actinobacillus pleuropneumoniae

ORGANISM Actinobacillus pleuropneumoniae

Bacteria; Proteobacteria; Gammaproteobacteria; Pasteurellales;

Pasteurellaceae; Actinobacillus.

REFERENCE 1 (bases 1 to 15355)

AUTHORS .

TITLE .

JOURNAL Unpublished

FEATURES Location/Qualifiers

source 1..15355

/organism="Actinobacillus pleuropneumoniae"

/mol_type="DNA"

/serovar="7"

/note="K locus: KL07"

CDS 1..1473

/gene="modF"

/locus_tag="APP7_1648"

/codon_start=1

/transl_table=11

/product="putative molybdenum transport ATP-binding protein"

/protein_id="ACE62300.1"

/translation="MPNINIQNALFSLAQHNKLSIESLEINTHDFWVIVGGNGSGKTA
FAQALHNSLSLYSGEYQNSFQHIALLSFEQQQKIIEQIFKHRNNDMISPDDFGLTARQ
IILNGSEKTQLCEEYAAKLRIQPLLDRPFIQLSTGESRKVLFCQMLVSEPDLLILDEP
FEGLDQASVAYWQDVMAQLGKQMAVVLISNRFNDIPDCATHIALLDNLQLILQGERQE
IEQQAVYSQLKFAEQNVNAPLPESATPLIQLPPNTNPFELKNVMIRYGEKTIIDDLTW
TVAPKQHWWIKGPNGAGKSTLLSIIAGDHPQSYANYVHLFGRQRGSGETIWDIKKNIG
YVSSQLHMDYRVNCSALDVILSGFFDSIGVYQQVPSALQLKAMEWLERLHLANLAKKP
FRSLSWGQQRLLLITRAMVKHPPILILDEPLQGLDGVNRKLVKQFIEQLVTNSQTQLL
FVSHQDADAPNCITHLFEFVPQENGGYRYVQTALNKTENT"

CDS complement(1822..2472)

/gene="cpxA"

/locus_tag="APP7_1647"

/EC_number="3.6.3.38"

/codon_start=1

/transl_table=11

/product="ATP-binding protein"

/protein_id="ACE62299.1"

/translation="MISVKNVSKDYYTRSGKKTVLQDINFELKKGEKIGILGRNGAGK
STLIRLLSGVEPPTSGTIERHMSISWPLAFSGAFQGSLTGMDNLRFICRIYNADIDYV
KAFTEEFSELGDYLYEPVKKYSSGMKARLAFALSLSVEFDCYLIDEVIAVGDSRFAAK
CKHELFEKRKDRSIILVSHSPSAMKSYCDNAMVLDKGIMYKFENMDEAYKFYNSTL"

CDS complement(2469..3266)

/gene="cpxB"

/locus_tag="APP7_1646"

/codon_start=1

/transl_table=11

/product="capsule polysaccharide export inner-membrane

protein"

/protein_id="ACE62298.1"

/translation="MQYGDQTTFRQSLAIQGRVIGALLMREIITRYGRKNLGFLWLFV

EPLLLTLFIVLMWKFIRADRVSDLNIIAFVITGYPMAMMWRNASNRTIGAISGNLSLL

YHRNVRVLDTLLARVLLEVAGATIAQIIIMALLILLGWIDMPKDTFYMIIAWVLMAFF

ALGLGLIICSIAQKFEAFGKIWGTLSFVLLPLSGAFFFVHALPSQAQQYATLIPMIHG

TEMFRHGYFGDSVITYESISYLVICDVAMLLFGLIMVKNFSKGIEPQ"

CDS complement(3266..4423)

/gene="cpxC"

/locus_tag="APP7_1645"

/codon_start=1

/transl_table=11

/product="capsule polysaccharide export inner-membrane

protein"

/protein_id="ACE62297.1"

/translation="METPIATSPAEKLQKPVKQKKSRFKKLNPLFWITVAIPTVLSAF

YFGSVASDIYISESSFVVRSPKNQTALTGVGALLQGSGFSRSQDDTYTVQEYMRSRTA

LEQLMQGLPVREYYENQGDIIARFNGFGLNNSKEAFYKYFRDRLSVDFDSVSGIASLR

IRAFNAEEGQQINQKLLAEGETLINRLNERARKDTISFAEQAVTEAENNVNETANALS

KYRIKNKIFDLPAQSGVQLSLISSLKSELIRVETQLAQLQSITPDNPQVDALLMRQKS

LRKEIDEQSKQLSSNSNSSIAIQTADYQRLVLANELAQQQLTAALTSLQNTKNEADRQ

QLYLEVISQPSKPDWAEEPYRLYNILATFFIGLMLYGVLSLLIASVREHKN"

CDS complement(4449..5636)

/gene="cpxD"

/locus_tag="APP7_1644"

/codon_start=1

/transl_table=11

/product="capsule polysaccharide export protein"

/protein_id="ACE62296.1"

/translation="MEIKKYNSIIGLALTTLFLSACSSLPTSGPSHSAILEANSQSSD

KPLPEVNVVELDNGLVQQLYQTQQSQQFSGFLGTVGSAGYAGAVNVGDVLEISIWEAP

PAVLFGGTFSSEGQGSGHLTQLPAQMVNQNGTVTVPFVGNIRVAGKTPETIQSQIVGA

LQRKANQPQALVKIANNNSADVTVIRQGNSIRMPLTANNERVLDAVAAVGGTTENIED

VTVKLTRGSEVKTLAFETLISDPAQNIMLRAGDVVSLLNTPYSFTGLGAVGNNQQMKF

SSKGITLAEAIGKMGGLIDTRSDPRGVFVFRHVPFAQLSLEQQAQWQAKGYAIGMDVP

TVYRVNLLEPQSMFLLQRFPMQDKDIVYVSNAPLSEFQKFLRMIFSITSPVTSTTNAV

RAY"

CDS 5815..6957

/gene="cps7A"

/locus_tag="APP7_1642"

/function="Putative glycosyl/glycerophosphate transferases

involved in teichoic acid biosynthesis

TagF/TagB/EpsJ/RodC"

/codon_start=1

/transl_table=11

/product="Glycerophosphotransferase"

/protein_id="ACE62294.1"

/translation="MLMKIAFIAWNSFQVLHFKPLLQALPCALLIIEKRRRSVPICKD

ILRDINNNIAYIRHTDIYAKIDGNFDVLVAQTTFEQLYLFHRTKIALLQYGYAKEPYN

YGTWRAFADLNLVYGNYAYERISYFSPTKITGCPRYDLWYQPLFHQKAKENYARVLDT

SKKTIVYAPSWGELSSFKLYIEEITKLSLFYNVLVKLHHNTLLLANKHQNYEKLYPNL

HFFYEGEDLLSLISVADIVISDFSGAIFDAIFCKKTVVLLSISLVNQPKLDKFSLEIA

YRSKLGYEVFSPDQVAITVARALTEPKLVDETLYQQLFMHNKDATQQVINALQQLAEG

KYTLSQQQLYVRQTEKLLNIEKIKQQKNKKQSFNKIRQISKRLIKK"

CDS 7010..7438

/gene="cps7B"

/locus_tag="APP7_1641"

/function="Cytidylyltransferase"

/codon_start=1

/transl_table=11

/product="glycerol-3-phosphate cytidylyltransferase"

/protein_id="ACE62293.1"

/translation="MKKVLTYGTFDLLHHGHIRLLERARSLGDHLTVAISTDQFNLGK

GKVCAYTYEERAHILKAIRYVDEVIPETNWEQKVEDVKNHEIDVFVMGDDWEGKFDFL

ADYCEVVYLPRTPDISTTQVKKMLAKKDLAAGQKQIHEKE"

CDS 7446..8582

/gene="cps7C"

/locus_tag="APP7_1640"

/codon_start=1

/transl_table=11

/product="conserved hypothetical protein"

/protein_id="ACE62292.1"

/translation="MFQILQKHLPTLQRVLREGYSQHSLLAYWYGLSLLTALEQANHP

QVRKLAEKMINKGINIGHYFLAQSYFLCGEYDLAEQAVKKIKNFVKIPEVVFLYADIL

VKCKRKEEAWQLLEQCALLNKRKKVWIHLTNLVNTEADYRHLEQHIDKVRTTTPYLKS

DLLIHQRTNAALRAGLTETALALTELNPLPKQAKVKKKTTAYNDKLAAIALADLKKVL

DHKKIPFFLISGTLLGCIREGKLLGHDKDIDVGVWDEYSYEELANCLSTSGYFYVVPT

RTKHLVMLRHVNGIAIDVFIHYREPNDYWHAGVKIKWHNSPFNLVYTNFLGQQYLIPE

NYDLYLTENYGDWRTPKTKFDSAFDTPNMEVINEAEMKIYIFRK"

CDS 8594..12427

/gene="cps7D"

/locus_tag="APP7_1639"

/function="Putative glycosyl/glycerophosphate transferases

involved in teichoic acid biosynthesis

TagF/TagB/EpsJ/RodC"

/codon_start=1

/transl_table=11

/product="Putative CDP-glycerol:glycerophosphate"

/protein_id="ACE62291.1"

/translation="MKKKFYKAIYLFNAPIIYWRGLKYYQKKEWKKAEKYFQLAVTRK

PDHAYSNFKLGMCFFKQKMWDQAYNYISIAVNLAPEMPLWKVQLRQSAAQLQVKKRRK

SVKRNSENQISTPEELKKVTGISANEVAEQILIELLEKEPENPTIYAELALIQSKQAK

LWQAVDSWTEAINRKSDNAMYFYQYGIVLEKLGHYARASEAYKTSLNLNPPNKILADL

FFRLGFVNKHQGHDNILNIELADEAYTKAIQADKKLKSKDFGIGVFYEERRDWQQAAI

AYENQIGKTIKEAELFYRIGFAYDRNYEWELAEINYQKALSITERPEWRFRLGLVLEK

QKKFIQATKNYEKAAIERKQYTPYWFYRCAYTLEQQGLYERASKMYLKLRKDPSLITK

ASHSSVNDLILALNIKHNYDTTSAHSWFELGSSYELIQNWEKSEYAYSQAIARSNELV

SLWYYRLGFVQIAQGKYLQACESLRSYRVMQRPHGVNEDILSKDISYAEVATYNEYYN

ILNIKEQTVLYESFSGQGMSCNPLALFLYLFNHNEYKNWTHIWVINDTSNIPEEYRKY

DNVIFIRRGSDSYLRYLATTKILINNSNFPPYFIRKPEQKFLSTWHGTPFKTLGRDME

GRFFEHKNLTRNIFQSTHLLSPNAHTSKILYERHDIKEIYTGRLIESGYPRIDMTLSL

AKEEKIELREKLGVLNNEKLVFYAPTWRGIHGDIEFDYEKLQSDLNKLSKLEGAKVVF

RGHSLLQEALSKINLGITVVPDELDTNKILSVTDILITDYSSVLFDYLPTLKPLVLYM

YDIKEYTEERGLYFSENELPGEKCYNINELVKTLTYLLENNITSVSFEDSKVAQFAPH

DDGNVSEKVINALFLDDYTDLKVINDIPENKKSLLIYGGPFMGNGITTSVINLISNID

RSKYTVTLVIDPGSIEKEAGRLRQFEKLPKDINVVARVGRMNMDLEERYIHGLNNQHY

ELQSSVARGILQDSWEKEYQRIFGNAKFDSLIQFEGYNRFWSGVFTSIQNKKSSIYMH

NSMEEEYRLKYPYLKSMFYYCSLANKVISVSELTMELNKDKLADKFGILSSKFDYSDN

LQQPEKIRKLADEPLLLDDEIYFKTPGKVFLTIGRLSIEKDHAKLINSFAKLIKYYPD

SKLLIIGDGSLKYALTQQIKELKLDNNVYLLGLRTNPFPLLKNADCFILPSNHEGQPM

TLFEAMILGKMIIATDIVGSRSALEGRSGYLVENSVDGLLKGMSDFLEGKLSLITFDI

NEYQEQAINRFYNVI"

CDS 12462..14075

/gene="cps7E"

/locus_tag="APP7_1638"

/codon_start=1

/transl_table=11

/product="hypothetical protein"

/protein_id="ACE62290.1"

/translation="MNIITISPNSLVIPENFDLRRINARPDEFIDPTYMDKYDNTSIF

YDIFESNNKIYLIGPPLLNLSPIINSCYIIFDNGREEKVSINSKLLERGQLSWIDLKE

IKYKPVSLRFDFSIFSISYKGNKNVIVDIGKDVNDEFNDAKSLMTLQLNNKLEWIHDW

AKYYNKVHDVDTIVIYDNNSTNYKLDDISNSLLSITNLKNIVVVPWNFKYGPQGKPWT

GPNTPWDSDFCQIGALQHMRFRFSLKSKGFINADIDELIIPLKEVNIFDALENSEVGV

IGVEGNTIEGHLSNHMMKAEGVPHFYHFWERKVHISGGTRKWAGSPSKWDDETVQTTA

HWVRGISYKADSRFSIGHFRQINDGWKIQSRTIEYSGKDILRPDFSLIGAMSVAFPNE

IPNILLVNALKDAEQRIQLLEKGKEDEYSKLQSYIKLLTHERIVWDKIWIWKGNVLVF

ETRCSLGKIAFDIVISNNNVQLNVSVRDTKYQEDFFEVVFRYLGTDFSILSNGKGLKA

YSLKRENISFEEIATLISKKILIFYKILN"

CDS 14187..14741

/locus_tag="APP7_1637"

/codon_start=1

/transl_table=11

/product="hypothetical protein"

/protein_id="ACE62289.1"

/translation="MSKKITLLSLAVFIAGCSSAPQPEAFPGEFANADYVLSDKDAQR

WVVASHQAEQCIYPNLTRIQQQAFSKEDSYIHSQYVFFYPLEEIIGEQYVKIIQDDEK

SMGYAQYLFKKFRDNQEFEPLADKQCLVLREKAKNDLAVVKGQYKSEMVEETKSEAKN

ADGVATNQNKFFFDIIKWGSMLLL"

CDS complement(14813..15355)

/gene="ydeN"

/locus_tag="APP7_1636"

/codon_start=1

/transl_table=11

/product="hypothetical protein"

/protein_id="ACE62288.1"

/translation="MKKVYVTHGYTANPTRNWFPWLKNELEKLGWECECLAMPNSDQP

NPQAWLEHHQNTLQLDENTLLIGHSLGCIALLNYLAVTQQKVKTAIFVSGFYEKLPTL

PELDSFADFYANQTACLPQKSYVISALNDVVVPHSFSDRLAQYLQADYIRLATGGHFI

DREGVTELPELLELIKQISN"

ORIGIN

1 atgccaaata tcaacatcca gaacgcctta ttttcccttg ctcaacacaa taaactgtcg

61 attgaatcgc tggaaatcaa tactcacgat ttctgggtga ttgtcggcgg taacggctcg

121 ggtaaaactg ctttcgccca agcgctacat aattcacttt cactatattc gggcgaatat

181 caaaacagct tccaacatat cgctttactt tccttcgagc agcaacaaaa aatcatcgag

241 caaatcttta aacaccgtaa caacgatatg atttcaccgg atgatttcgg tttaaccgcc

301 cgtcaaatta tcttaaacgg tagcgaaaaa acgcaattat gcgaagaata tgcagctaaa

361 ttacgtattc agccgttatt agatcgcccg tttattcagc tatccaccgg cgaaagtcgc

421 aaagtgctat tttgccaaat gttagttagc gaaccggatt tgctgatttt agatgagcct

481 tttgaggggt tagaccaagc ctcggtcgct tattggcaag acgtgatggc acaactcggc

541 aagcaaatgg cggtggtact gatttccaac cgttttaatg atattcccga ctgtgccaca

601 catattgctt tactggataa cttacaactg attttacaag gcgaacgcca agagattgaa

661 caacaagcgg tctattctca gctaaaattt gcagaacaga atgtgaatgc accgttgccg

721 gagagtgcca caccgctgat tcaactccca ccgaatacta atccgtttga actgaaaaac

781 gtaatgatcc gttacggcga aaaaacgatt attgatgatc taacttggac ggttgcccca

841 aaacaacatt ggtggattaa aggcccgaac ggagcaggaa aatcgacctt actttctatt

901 attgccggcg atcatccgca atcttacgct aattatgtgc atttattcgg tcgtcagcgt

961 ggttcgggcg aaacgatttg ggatataaag aaaaatatcg gctatgtgag cagccaatta

1021 catatggatt atcgggtgaa ttgctctgcg ttagacgtga ttttatctgg cttttttgat

1081 tcaatcggcg tttatcaaca agtaccgagt gccttacagc taaaagcaat ggaatggctg

1141 gaacgcttgc atttagccaa tctggcgaaa aaaccgttcc gttcactttc gtgggggcaa

1201 caacggttat tattgattac tcgtgccatg gtaaaacatc cgccgattct gattttagac

1261 gaaccgctgc aaggcttgga cggtgtaaac cgcaaattgg ttaaacagtt tatcgagcag

1321 ttggtcacta acagccaaac ccagttgcta tttgtttcgc accaagatgc ggacgcccca

1381 aattgcatca cccatttatt tgaatttgtc ccgcaagaga atggtggtta tcgttatgta

1441 cagacggctt tgaataaaac agaaaacacc tgagttcaac cacggaaaac acggattgca

1501 cggagtttaa tgaatcggtc agttgacgta atgtttggat aatattgttt ttttcaggtt

1561 tttcttatct cgtaaggaac taataaaact ttaatcggaa actgcaacta ctcgcaatat

1621 cataatttgt taacctttaa aggaaatccc cctctttagt aaagaggggt taggggagat

1681 ttgtcaatag agatatgaaa ttgaatagaa cttcattttt tatatttata aaagcgttaa

1741 ttagcatatt tcttcgctaa ttcattctgt caaatctcct cctgcccctc tttgctaaag

1801 aggggagata tgtgtgggac tttaaagcgt tgaattatag aacttataag cctcgtccat

1861 attctcaaat ttatacataa tccctttatc taataccatt gcattatcgc aataagactt

1921 cattgctgac ggactatgcg aaaccaaaat aatcgaacga tctttgcgct tttcaaataa

1981 ttcatgttta cattttgccg caaagcgaga gtcacctacc gcaattacct catcaattaa

2041 gtagcaatca aactcaactg aaagcgataa agcaaacgca agtcgggctt tcataccgga

2101 agaatatttc ttcaccggct catataaata atcgcctaat tcggaaaact cttcggtaaa

2161 tgctttaaca tagtcaatat ccgcattata gatacggcaa ataaagcgta aattatccat

2221 accggttaaa ctgccttgaa acgccccgct gaaagcgagc ggccaagata tcgacatgtg

2281 acgttcgata gtacctgatg ttggcggttc aacaccactt aacaaacgga ttagcgttga

2341 tttccctgca ccgttacgcc ctaaaatacc gattttctcg cctttttca gctcaaaatt

2401 aatatcttgc aatacggttt ttttaccgct tcgagtatag taatctttac tcacattttt

2461 tacgctaatc attgcggttc gattccttta ctgaagtttt ttaccataat gagcccaaaa

2521 agtaacatgg ctacatcaca tatgacgaga taacttatac tttcatatgt gataacactg

2581 tcgccaaaat aaccgtgacg aaacatttcc gtgccgtgaa tcatcggtat taaggttgca

2641 tattgttgag cttggcttgg tagcgcatgc acaaagaaaa atgcgcctga aagaggtaaa

2701 agaacaaagc ttaatgttcc ccagattttg ccaaatgctt caaatttttg tgcaatggag

2761 caaatgatca agccgagccc taaagcaaag aatgccatta atacccatgc tattatcata

2821 tagaacgtat ctttcggcat atcaatccaa cccaataata ttaataatgc cataataatg

2881 atttgggcaa ttgttgcgcc tgctacttcc agtaggactc gagcgagtaa agtatccaat

2941 acacgtacgt tacgatgata aagaagactc aaatttcctg atattgctcc gatagttcta

3001 tttgaagcat tacgccacat cattgccatt ggataaccgg taatcacaaa agcaataata

3061 tttaaatcgg aaacgcgatc cgctcggata aatttccaca tcaaaacgat aaataaagtg

3121 agtaatagcg gctcaacaaa cagccataaa aaacccaaat tttttcgtcc gtaacgcgta

3181 ataatttccc gcatgagtaa tgcaccgatt actctccctt gaatggcgag agattggcgg

3241 aaagttgttt gatcaccgta ttgcattagt ttttgtgctc tcttacgctt gcaattaata

3301 aacttaatac accataaagc atcagaccga taaagaatgt cgctaaaata ttatataagc

3361 gataaggctc ttccgcccag tccggtttgc ttggctgact gattacttct aaataaagtt

3421 gctggcgatc cgcttcattt ttcgtatttt gtaatgaggt taatgctgcg gtcaattgtt

3481 gctgtgccag ctcgtttgca agtactaagc gttggtaatc ggcagtttga atagcaatag

3541 agctattact gttactggaa agctgttttg attgctcatc gatttcctta cgtaaacttt

3601 tttggcgcat aagcaatgca tcaacttgcg ggttatccgg cgtaatcgat tgtaattgcg

3661 ctaattgggt ttccacacga atcaactcgc tttttaagct tgaaattaat gaaagttgta

3721 cgcccgattg tgccggtaaa tcaaaaattt tatttttgat acggtattta cttaaagcat

3781 ttgctgtttc gtttacatta ttttccgctt ctgtaaccgc ttgttccgca aatgaaatgg

3841 tatctttttct tgcacgttcg tttaaacggt taataagcgt ttcaccttcg gcaaggagtt

3901 tttgattaat ttgttgcccc tcttccgcgt taaatgctcg aatacgtaag ctggcgatac

3961 cggaaacaga gtcaaagtcc acacttaagc gatctcggaa atatttataa aacgcttctt

4021 tactgttatt taatccgaat ccattaaagc gagcgataat atcgccctga ttttcataat

4081 attcgcgaac aggtaatcct tgcattagtt gctcaagagc ggtacgagaa cgcatatatt

4141 cctgtacggt ataagtatcg tcttgcgaac gagaaaaacc tgaaccttgc aataaggcgc

4201 ctacgcctgt cagagcggtc tgatttttag gcgatctaac gacaaaactt gattccgaaa

4261 tataaatatc agaagcgacc gaaccgaaat aaaacgctga tagcacggta gggattgcga

4321 cagttatcca aaataatgga ttgagttttt taaagcggct tttttctgt ttaaccggtt

4381 tctgtaattt ttctgctgga ctggtagcaa taggtgtttc catcttttgt ccttatacat

4441 tcaatatatt aataggcacg aacggcattg gtcgtactgg taaccggcga agtaattgag

4501 aaaatcattc tcaagaattt ttggaactca gacaacggcg catttgaaac atacacaata

4561 tctttatctt gcattgggaa acgctgtaat aaaaacatgg attgcggctc aagtaagttc

4621 acacgataaa ccgttggtac atccattcct atagcgtagc ctttagcttg ccattgtgct

4681 tgttgttcta aactcaattg tgcaaaaggc acgtgacgga atacgaaaac ccctctcgga

4741 tccgaacgag tatcaattag accccccatc ttaccgatag cttcggcaag cgtaattcct

4801 ttacttgaga atttcatttg ctggttattg cccaccgcac ctaaaccggt aaaactataa

4861 ggtgtgttta gcagtgaaac aacatcgccg gcacgtaaca taatattttg tgccggatcg

4921 gaaattaacg tttcgaatgc gagtgttttt acttcagaac cacgggttag cttgaccgtc

4981 acatcttcaa tgttttccgt tgttccgcct actgcagcaa ccgcatctaa tacacgttca

5041 ttattagcgg ttaatggcat acgaatacta ttgccttgac gaataaccgt aacatcagca

5101 gagttattat tcgcaatttt gactaatgct tgcggctgat tcgctttacg ctgcaatgcc

5161 ccaacaattt gagactgaat cgtttcaggt gttttacctg cgacacgaat atttcccaca

5221 aacggcacgg ttaccgtccc gttttgatta accatttgtg ccggtaattg cgttaaatgc

5281 ccgctacctt gtccttcgga actaaaagta ccgccaaaca acactgccgg cggcgcttcc

5341 caaattgaaa tttcaagtac atcacccaca ttgactgcac cggcatagcc cgcgctgcct

5401 actgtgccta aaaatccgga aaattgttgg ctttgctgag tttgatacaa ctgttgaact

5461 aaaccgttat ccagttccac cacatttact tccggtaagg gtttatccga actttgtgaa

5521 ttagcctcta agatcgcact atggctaggg cctgaagttg ggaggcttga gcaagcagaa

5581 aggaacaatg ttgttaaagc taaccctatg attgaattat attttttaat ttccatcttg

5641 taaaaaggct ctattgaaaa agtgtgtaaa atgaatataa actatacata attatagata

5701 caacactata attatatatt ataatctaat atctcgtttt ttaaaacgat aacctaaaat

5761 attacttttc tatagaagaa taggcattat tttaataaat ataattaggt atgaatgttg

5821 atgaaaatag catttatcgc ttggaatagt tttcaggtgt tacatttcaa gcccttatta

5881 caagctttac cgtgtgcatt attaattatt gaaaaacgga gacgtagtgt accaatctgt

5941 aaggatattt tgcgagatat aaacaataat atcgcttata tccgccatac ggatatatat

6001 gcaaaaattg atgggaattt tgatgttcta gttgctcaaa ctactttcga gcaactttat

6061 ttgtttcacc gcaccaaaat tgcattgctt caatacggat atgctaagga accgtataac

6121 tacggcactt ggagagcatt tgcagatcta aatttggttt atgggaatta tgcctatgaa

6181 cgtatttcct atttctctcc aactaaaata accggttgtc cacgatacga tttatggtat

6241 cagcctttat ttcatcaaaa agcgaaagaa aattatgcga gagtattaga tacgagtaag

6301 aaaacgattg tatatgcacc aagttgggga gaattatcca gctttaaatt atatatagaa

6361 gaaattacga aattatcttt attttacaac gtgttagtaa aattacacca taacacgctt

6421 ttattagcaa acaagcatca gaattatgaa aaattgtatc cgaatttaca ttttttctat

6481 gaaggtgaag atcttctctc acttatttcg gtagccgaca ttgttatttc cgattttagc

6541 ggtgcgatct ttgacgcaat tttttgtaaa aaaacagtag tgttactttc gatatcgcta

6601 gtgaatcaac caaaactaga taaatttagt ttagagatag cttatcgttc aaagttagga

6661 tatgaggttt tttcgccgga tcaagtagct ataacagtgg cacgagcact tacagagccg

6721 aaattagtag atgaaacgct gtatcaacag ctttttatgc ataacaagga tgcaacacag

6781 caagtaataa atgctttaca acagcttgct gagggtaagt atacattatc tcaacagcag

6841 ttatatgtgc gacaaacaga aaaattatta aatattgaaa aaataaagca gcaaaaaaat

6901 aaaaaaacagt ctttcaataa aataagacag atttctaaaa gattaattaa aaaataattt

6961 ttcatatttt ctttttatat ttttttattt gacaatacgg agtttaggaa tgaaaaaagt

7021 attaacctat ggaacctttg atttattaca ccatgggcat attcgtttat tagaaagagc

7081 aagatcatta ggcgatcacc ttactgttgc tatttctacc gatcaattta acttaggaaa

7141 aggcaaagta tgcgcttata cttacgaaga gagagcgcat attttaaaag caatccgtta

7201 tgtggatgaa gtaattcctg aaacaaactg ggagcaaaaa gttgaggatg taaaaaatca

7261 cgaaattgac gtatttgtaa tgggtgatga ctgggaaggc aaatttgact tcttagcaga

7321 ttactgcgaa gtggtttatt taccgagaac ccctgatatt tcaacgactc aggtaaaaaa

7381 aatgcttgcg aaaaaagatc tcgcagccgg acaaaaacaa attcacgaaa aagagtaatt

7441 tgttaatgtt tcaaatccta caaaagcatt taccgacctt gcaaagagtt ctgagggagg

7501 gttactccca gcattctttg cttgcttatt ggtatgggct tagtctactt actgctcttg

7561 aacaggcgaa tcatcctcaa gtaagaaaac tggctgagaa aatgatcaat aaaggtatta

7621 atatcgggca ttatttttta gcacaaagtt atttcttatg tggagaatat gatttagcgg

7681 aacaagcggt caaaaaaatc aaaaattttg taaaaatacc cgaagttgtt tttttatatg

7741 cggacattct cgttaaatgc aaacgtaaag aagaggcttg gcaattatta gaacaatgcg

7801 ctttactcaa taaaagaaaa aaagtgtgga tacatctaac aaatttagta aatactgagg

7861 cggattatcg acacttagaa caacatattg acaaagtaag aacaactaca ccttacttga

7921 agtctgattt gttaattcat caaagaacaa atgcagcatt aagggctggt ttaacagaaa

7981 cggcattagc actaacagaa cttaaccctt tgccaaagca agcaaaagtg aagaaaaaaa

8041 caaccgctta taatgataaa ttagcggcaa ttgcgctagc ggatctaaag aaagtattag

8101 atcacaaaaa aatacctttc tttctgatta gcggcacgtt gctaggttgt attcgagaag

8161 gaaaattatt agggcatgat aaagatattg atgtcggcgt ttgggatgag tactcctacg

8221 aagaattagc aaactgttta tctacatcgg gatactttta cgtagtacca actcgaacaa

8281 aacatttagt catgttaaga cacgttaatg gtatcgcaat tgatgtgttt attcattatc

8341 gcgaacctaa tgactattgg cacgctggtg tcaaaataaa atggcataat tcaccattta

8401 atttggtata tacaaatttc cttgggcaac aatatttaat acctgaaaat tatgatttat

8461 acctaacgga aaattatggt gactggcgta cgccaaaaac aaaattcgac agtgcttttg

8521 atactcctaa tatggaagta atcaatgaag ctgaaatgaa aatatatata ttcagaaaat

8581 aataaggtta tatatgaaga aaaaatttta taaagcaatt tatcttttta atgctcctat

8641 tatttattgg cgaggattga agtattatca gaaaaaagaa tggaaaaaag ctgagaaata

8701 ttttcaatta gcagttaccc gtaaacctga tcatgcatat agtaatttca aactaggtat

8761 gtgctttttt aaacagaaaa tgtgggatca agcatacaac tatatttcta ttgccgtaaa

8821 tcttgcccca gaaatgcctc tatggaaagt ccaattacgt cagtctgcag cccaattaca

8881 agtgaagaag cgcagaaaaa gtgtgaagag aaattctgaa aatcagataa gtacacctga

8941 agagttaaaa aaagtgacag gcatttctgc taatgaggtc gcggaacaaa ttttaattga

9001 attattagaa aaagaaccag aaaatcctac aatttatgcg gaactcgcat taattcaaag

9061 taaacaggct aaactgtggc aggcagtaga ttcttggact gaagctataa atcgtaaatc

9121 tgataatgct atgtatttct atcagtatgg aattgtttta gaaaaattag ggcattatgc

9181 tagagcatct gaagcatata aaacatctct taatttaaat cctccaaata aaatacttgc

9241 agatttgttc tttcgtttag gatttgtaaa taagcatcaa ggtcatgata atatactgaa

9301 tattgaactg gcagatgaag cttatactaa agctattcag gctgataaaa aacttaaatc

9361 aaaggatttt ggtataggag ttttctatga ggaacgtaga gattggcaac aagcggctat

9421 agcgtatgaa aatcaaattg ggaaaacaat aaaagaagct gagttatttt atcgtatagg

9481 ttttgcatat gatcgtaatt atgagtggga attggctgaa ataaattatc aaaaggcttt

9541 atctattaca gagcgcccag aatggcgttt ccgtttaggt cttgtattag agaaacagaa

9601 gaaatttatt caagctacaa agaattatga aaaagcagct atagagagaa aacaatatac

9661 tccgtattgg ttctatcgtt gcgcttatac gttagagcag caggggctat atgaaagagc

9721 ctcaaaaatg tatttgaaat tacgtaaaga tccctcatta attaccaaag caagtcattc

9781 atctgtaaat gatcttattt tggcattaaa tataaaacat aattatgata ctacgtcagc

9841 tcattcatgg tttgaattgg gttcctctta tgaattaatc cagaactggg agaagtcgga

9901 atatgcttat tctcaagcta ttgctagaag taatgagtta gtttcgctat ggtattatcg

9961 tttagggttt gttcaaattg ctcaaggtaa gtatttgcaa gcatgcgaga gtttaagaag

10021 ttatcgagtg atgcaacgtc cgcatggagt aaatgaagac attctatcaa aagatattag

10081 ctacgcagaa gtagccacat ataatgaata ctataatatt ttaaatataa aagaacaaac

10141 tgttctatat gaaagttttt ctgggcaagg aatgagttgt aatccattag ccttattttt

10201 atatctattt aatcataatg aatataagaa ttggactcat atttgggtca ttaatgatac

10261 tagtaatatt cctgaagaat ataggaaata tgataatgta atctttatta gaagaggaag

10321 tgatagttat ttacgttatt tagcaactac taagatatta attaataata gcaattttcc

10381 tccatatttt attagaaaac ctgaacagaa gtttttaagt acatggcacg gaactccatt

10441 taaaacatta ggaagagata tggagggaag atttttgaa cataaaaatc taaccagaaa

10501 tatatttcaa tctactcatt tattaagtcc aaatgctcat acctcaaaga ttctttatga

10561 gcgccatgat attaaagaaa tctatactgg aagactaatt gaatcaggtt atcctcgaat

10621 tgatatgaca ttgtcattag caaaagagga gaaaattgag ttaagagaga agttaggtgt

10681 tttaaataac gaaaaattag tattttatgc accgacctgg agaggtattc atggggacat

10741 agaattcgat tatgagaagt tgcaatcaga tttaaataaa cttagcaaat tagaaggtgc

10801 taaggttgtt tttagagggc attcattact tcaggaagca ttatccaaaa ttaatctagg

10861 tattactgtt gttcctgatg aattagatac gaataagatt cttagtgtaa ctgatatatt

10921 aattactgat tattcaagtg ttttatttga ttatttacca acattaaagc cattagttct

10981 ttatatgtat gatatcaaag aatatacaga agaacgagga ttatactttt cagaaaatga

11041 attacctgga gaaaaatgct ataacataaa tgaattagtg aagacattaa cgtatttatt

11101 agagaataat attacctctg tgtcgtttga agatagtaaa gtggctcaat ttgctcctca

11161 tgatgatggt aatgtttcag aaaaagtcat taatgcttta tttttagatg actatacaga

11221 tctaaaagtt attaatgata ttccagaaaa taaaaagtct ttgctcattt atggtggtcc

11281 ttttatgggt aatggaataa ctacatcggt aattaatctt atttctaata ttgatcgttc

11341 aaaatatacg gtaacattag taattgatcc aggctctatt gagaaagagg caggacgatt

11401 aagacaattt gaaaaattac cgaaagatat aaacgtagtt gctcgtgttg gacgaatgaa

11461 tatggattta gaagaaagat atattcatgg attaaataat cagcattatg aattgcaatc

11521 ttcggtagct cgaggtattt tacaggattc ttgggagaag gaatatcagc gcatatttgg

11581 caatgctaaa tttgattcat tgattcagtt tgaagggtat aaccgttttt ggtctggagt

11641 gtttacatct attcagaata agaaatctag tatttatatg cataattcta tggaagaaga

11701 atatcgacta aaatatccat atttaaaatc aatgttttat tattgttctt tagcaaataa

11761 agtgatatct gtgtcggaat taactatgga attaaataaa gataagttag cagataagtt

11821 tggtatttta tcaagtaaat ttgattatag tgataattta cagcagccag agaaaattag

11881 gaagctagct gatgagccct tgctattaga tgatgagata tattttaaaa cgccaggaaa

11941 ggtgttttta acaataggta gactatcaat agagaaggac cacgctaagt taattaatag

12001 ttttgcaaaa cttataaaat attatcccga tagtaaatta ttaattattg gagatggctc

12061 acttaaatat gctctaactc agcaaattaa agagttaaaa ttagataata atgtatattt

12121 actagggtta aggacaaatc cattcccgtt attgaagaat gctgactgtt ttatattacc

12181 ttcaaaccat gagggacaac ctatgacttt atttgaagct atgattttag gaaaaatgat

12241 tattgcaaca gatattgttg gatcaaggag tgcactagaa ggaagatcag gttatttagt

12301 tgagaattct gtagatggat tgttaaaagg aatgtcagac ttcctagaag gaaaattatc

12361 attaattact tttgatataa acgagtatca agagcaagca attaatagat tttataatgt

12421 tatttaagca ttgtatttta ataaaatcag gagattttaa tatgaatatt attactataa

12481 gtcctaattc gttggttata ccagaaaatt ttgatttaag acggataaat gctagacccg

12541 atgagtttat agatcctaca tatatggata aatatgataa tacatctatt ttttatgata

12601 tatttgagag taataataag atatatttaa ttggtcctcc attgttaaat ttatcgccta

12661 ttattaatag ttgctatatt attttcgata atggtagaga agaaaaagta agcataaatt

12721 ccaagctctt agagagaggt caattaagtt ggattgattt aaaagaaatc aagtataaac

12781 cagtatcttt aagatttgac ttctctattt ttagtatttc ttataaagga aataagaatg

12841 ttattgttga tataggaaag gatgttaatg atgaatttaa cgatgcaaaa tctttaatga

12901 cattacagct aaataataaa ttggaatgga ttcatgattg ggccaaatac tataataaag

12961 tacatgatgt agatacaata gttatatatg ataataattc tactaattat aaactggatg

13021 atatatctaa ttctttgtta agtataacta acttaaaaaa tattgttgtt gttccttgga

13081 attttaaata tggtcctcaa gggaaacctt ggactgggcc aaatactcct tgggactccg

13141 atttttgtca aattggggca ttacaacata tgagatttag attttcacta aaatctaaag

13201 gttttattaa tgctgatata gatgaactaa ttattccttt gaaagaagtt aatatttttg

13261 atgcacttga aaatagtgag gttggagtaa tcggagttga aggaaataca attgaaggtc

13321 atctctcaaa tcatatgatg aaagctgaag gggttcccca tttttatcat ttttgggaga

13381 gaaaagtcca tattagcggg ggaacacgaa aatgggcagg ttctccaagt aaatgggatg

13441 atgaaacagt tcaaacaaca gctcattggg taagaggaat ttcatataaa gctgatagtc

13501 gtttttcaat aggacatttc cgtcaaataa atgatggatg gaaaattcaa agtagaacga

13561 tcgaatatag tggtaaagat attttgagac ctgatttttc attgattgga gccatgtcag

13621 ttgcttttcc taatgagata cctaatattt tattagttaa tgcattaaaa gatgcagaac

13681 agagaatcca attattagaa aaaggtaaag aagacgaata ttcaaaatta caatcttaca

13741 ttaagttatt gacacatgag cgtattgttt gggataaaat ttggatatgg aaagggaatg

13801 ttttagtatt tgaaacaaga tgctcattgg ggaaaatagc atttgatatc gttatatcaa

13861 ataataatgt ccaattaaat gtttcagtaa gggatactaa atatcaagaa gatttctttg

13921 aagtagtatt tagatatttg ggaacggatt ttagtatttt atctaatgga aaaggattga

13981 aagcatattc tttaaagaga gaaaatatta gtttcgagga gattgcaaca ttaataagta

14041 aaaaaaatatt aatattttat aaaatattaa attaatattt aattgagtaa ttagtatttt

14101 aaaaactaac tcaaattaag taataaagtt gttattttaa tattgctatc tattatactt

14161 tctaaggtta agagtcaaaa catatcatgt ccaaaaaaat cactttattg agtcttgccg

14221 tattcatcgc aggttgttcc tctgctccgc agccggaagc tttcccaggg gagtttgcga

14281 atgcggatta tgtgttatcg gataaagatg cccagcgttg ggtggtggcg agccatcagg

14341 cggagcagtg tatttatccg aatttaacgc gtattcagca gcaagcgttt agcaaggaag

14401 attcatatat tcattcgcaa tacgtatttt tctatccgct ggaagaaatt atcggcgagc

14461 agtatgtaaa gattatccaa gacgatgaaa aatctatggg atatgcgcaa tacttgttta

14521 agaaattcag agataatcag gaattcgagc cgttagcgga taagcaatgt cttgtgttgc

14581 gagaaaaagc gaagaacgat ttagcggtcg taaaagggca gtataagagc gaaatggttg

14641 aagaaacgaa gtccgaagct aaaaatgcgg acggcgtggc gaccaatcaa aataaattct

14701 tctttgatat tatcaaatgg ggttcgatgc tattactgta atttgcggtt agtgtgatgt

14761 taaagagtat aaaaacgagc tgtcggtaaa tcgttatcgg cagctctttt tattagtttg

14821 atatttgttt gattaactcc aataactccg gtaattccgt tactccctct cgatcgataa

14881 aatgcccgcc cgttgccaag cgaatataat ccgcttgtaa gtattgcgct aatcgatcgc

14941 tgaacgaatg gggaacgaca acatcattta atgcagatat gacgtaagac ttttgcggta

15001 aacaagcggt ctgatttgca taaaaatctg caaagctatc taattccggc aaagttggta

15061 atttctcata aaagccggaa acaaaaaattg ccgtttttac tttttgctgc gttaccgcaa

15121 gataattcag taacgcaatg cagcccaaac tatgtccgat gagtaaggta ttttcatcta

15181 attgaagtgt attttggtga tgttccagcc atgcttgcgg attcggctga tcggaattcg

15241 gcatcgctaa acattcacat tcccatccta atttttccaa ttcgttttta agccacggaa

15301 accaatttct tgtcgggttc gccgtataac cgtgcgttac atatactttt ttcat

//

LOCUS **LN908249.1** 18614 bp DNA linear BCT 20-APR-2021

DEFINITION Actinobacillus pleuropneumoniae serovar 8 isolate MIDG2331
capsular polysaccharide gene locus, complete sequence.

ACCESSION LN908249 REGION: complement(1845319..1863932)

VERSION LN908249.1

KEYWORDS .

SOURCE Actinobacillus pleuropneumoniae serovar 8

ORGANISM Actinobacillus pleuropneumoniae serovar 8
Bacteria; Proteobacteria; Gammaproteobacteria; Pasteurellales;
Pasteurellaceae; Actinobacillus.

REFERENCE 1

AUTHORS Chaudhuri,Roy.

TITLE Direct Submission

JOURNAL Submitted (18-NOV-2015) UNIVERSITY OF SHEFFIELD, Department of
Molecular Biology and Biotechnology, University of Sheffield, Firth
Court, Western Bank, Sheffield, S10 2TN, United Kingdom

FEATURES Location/Qualifiers

source 1..18614

/organism="Actinobacillus pleuropneumoniae serovar 8"

/mol_type="genomic DNA"

/serovar="8"

/note="K locus: KL08"

/isolate="MIDG2331"

/isolation_source="pig lung"

/host="Sus scrofa"

/db_xref="taxon:754345"

/chromosome="I"

CDS 1..1458

/gene="modF"

/locus_tag="MIDG2331_01774"

/inference="ab initio prediction:Prodigal:2.6"

/inference="similar to AA sequence:ABY70171.1"

/codon_start=1

/transl_table=11

/product="molybdate transport system ATP-binding protein"

/protein_id="CUU53011.1"

/translation="MSNINIQNALFSLAQHNKLSIESLEINTHDFWVIVGGNGSGKTA

FAQALHNSLSLYSGEYQNSFQHIALLSFEQQQKIIEQIFKHRNNDMISPDDFGLTARQ

IILNGSEKTQLCEEYAAKLRIQPLLDRPFIQLSTGESRKVLFCQMLVSEPDLLILDEP

FEGLDQASVAYWQDVMAQLGKQIAVVLISNRFNDIPDCATHIALLDNLQLILQGERQE

IEQQAVYSQLKFAEQNVSAPLPESAIPLIQLPPNTNPFELKNVMIRYGEKTIIDDLTW

TVAPKQHWWIKGPNGAGKSTLLSIIAGDHPQSYANYVHLFGRQRGSGETIWDIKKNIG

YVSSQLHMDYRVNCSALDVILSGFFDSIGVYQQVPSALQLKAMEWLERLHLANLAKKP

FRSLSWGQQRLLLITRAMVKHPPILILDEPLQGLDGVNRKLVKQFIEQLVTNSQTQLL

FVSHQDADAPNCITHLFEFVPQENGGYQYVQTVLN"

CDS complement(1655..2305)

/gene="cpxA"

/locus_tag="MIDG2331_01773"

/inference="ab initio prediction:Prodigal:2.6"

/inference="similar to AA sequence:ABY70170.1"

/codon_start=1

/transl_table=11

/product="capsule polysaccharide export transport system

ATP-binding protein"

/protein_id="CUU53010.1"

/translation="MISVKNVSKDYYTRSGKKTVLQNINFDLHKGEKIGILGRNGAGK

STLIRLMSGVEPPTTGTIERHMSISWPLAFSGAFQGSLTGMDNLRFICRIYNADIDYV

KAFTEEFSELGDYLYEPVKKYSSGMKARLAFALSLSVEFDCYLIDEVIAVGDSRFAAK

CKYELFEKRKDRSIILVSHSPSAMKEYCDNAMVLDKGIMHKFENMDDAYKFYNSTL"

CDS complement(2302..3099)

/gene="cpxB"

/locus_tag="MIDG2331_01772"

/inference="ab initio prediction:Prodigal:2.6"

/inference="similar to AA sequence:ABY70169.1"

/codon_start=1

/transl_table=11

/product="capsule polysaccharide export transport system

permease protein"

/protein_id="CUU53009.1"

/translation="MQYGDQTTFRQSLAIQGRVIGALLMREIITRYGRKNLGFLWLFV

EPLLLTLFIVLMWKFIRADRVSDLNIIAFVITGYPMAMMWRNASNRTIGAISGNLSLL

YHRNVRVLDTLLARVILEVAGATIAQIIIMALVILLGWIEMPKDTFYMVMAWVLMAFF

ALGLGLIICSIAQKFEAFGKIWGTLSFVLLPLSGAFFFVHALPSQAQQYATLIPMIHG

TEMFRHGYFGDNIITYESISYLVICDLAMLLIGLIMVKNFSKGIEPQ"

CDS complement(3099..4256)

/gene="cpxC"

/locus_tag="MIDG2331_01771"

/inference="ab initio prediction:Prodigal:2.6"

/inference="similar to AA sequence:ABY70168.1"

/codon_start=1

/transl_table=11

/product="capsule polysaccharide export transport system

permease protein"

/protein_id="CUU53008.1"

/translation="METPIATSPAEKLQKPVKQKKSRFKKLNPLFWITVAIPTVLSAF

YFGSVASDIYISESSFVVRSPKNQTALTGVGALLQGSGFSRAQDDTYTVQEYMHSRTA

LEQLMKDLPIREYYENQGDIIARFNGFGLNNSKEAFYKYFRDRLSVDFDSVSGIASLR

IRAFNAEEGQQINQKLLAEGETLINRLNERARKDTISFAEQAVKEAENNVNATASDLS

KYRIKNKIFDLPAQSGVQLSLISSLKSELIRVETQLAQLQSITPDNPQVDALLMRQKS

LRKEIDEQSKQLSGNSNSSIATQTADYQRLVLANELAQQQLTAALTSLQNTKNEADRQ

QLYLEVISQPSKPDWAEEPYRLYNILATFFIGLMLYGVLSLLIASVREHKN"

CDS complement(4282..5469)

/gene="cpxD"

/locus_tag="MIDG2331_01770"

/inference="ab initio prediction:Prodigal:2.6"

/inference="similar to AA sequence:ABY70167.1"

/codon_start=1

/transl_table=11

/product="HexD, capsule biosynthetic locus protein"

/protein_id="CUU53007.1"

/translation="MEIKKYNSIIGLALTTLFLSACSSLPTSGPSHSAILEANSQSSD

KPLPEVNVVELDNGLVQQLYQTQQSQQFSGFLGTVGSAGYAGAVNVGDVLEISIWEAP

PAVLFGGTFSSEGQGSGHLTQLPAQMVNQNGTVTVPFVGNIRVAGKTPEAIQSQIIGA

LQRKANHPQALVKIANNNSADVTVIRQGNSIRMPLTANNERVLDAVAAVGGTTENIED

VTVKLTRGSEVKTLAFETLISDPAQNIMLRAGDVVSLLNTPYSFTGLGAVGNNQQMKF

SSKGITLAEAIGKMGGLIDTRSDPRGVFVFRHVPFAQLSLEQQAQWQAKGYAIGMDVP

TVYRVNLLEPQSMFLLQRFPMQDKDIVYVSNAPLSEFQKFLRMIFSITSPVTSTTNAV

RAY"

/note="predicted cleavage at residue 34"

CDS 5648..6790

/gene="cps8A"

/locus_tag="MIDG2331_01769"

/inference="ab initio prediction:Prodigal:2.6"

/inference="similar to AA sequence:ABY70166.1"

/codon_start=1

/transl_table=11

/product="Cps2A"

/protein_id="CUU53006.1"

/translation="MLMKIAFIAWNSFQVLHFKPLLQALPCALLIIEKRRRSVPICKD

ILRDINNNIAYIRHTDIYAKIDGNFDVLVAQTAFEQLYLFRHTKIALLQYGYAKEPHN

YGTWRALADLNLVYGSYAYERISYFSPTEITGCPRYDLWYLPSFHQKAKENYAKVLDT

NKKTIVYAPSWGELSSLPLYIEEIKKLSSSYNVLVKLHHNTLLLANKHQNYESLYPNL

HFFYESEDLLSLISVADIVISDFSGAIFDAIFCKKTVVLLSISLVNQPKLDKFSLEIA

YRSKLGYEVFSPDQVAITVARALTEPKLVDETLYQQLFMHNKDATQQVINALQQLAEG

KYTLSQQQLYVRQTEKLLNIEKIKQQKNKKQSFNKIRQISKRLIKK"

CDS 6843..7271

/gene="cps8B"

/locus_tag="MIDG2331_01768"

/inference="ab initio prediction:Prodigal:2.6"

/inference="similar to AA sequence:ABY70165.1"

/codon_start=1

/transl_table=11

/product="Glycerol-3-phosphate cytidylyltransferase"

/protein_id="CUU53005.1"

/translation="MKKVLTYGTFDLLHHGHIRLLERARSLGDHLTVAISTDQFNLGK

GKVCAYTYEERAHILKAIRYVDEVIPETKWEQKIDDVKNHEIDVFVMGDDWEGKFDFL

ADYCEVVYLPRTPDISTTQVKKMLAKKDLATGQKQIHEKE"

CDS 7279..8421

/gene="cps8C"

/locus_tag="MIDG2331_01767"

/inference="ab initio prediction:Prodigal:2.6"

/inference="similar to AA sequence:ABY70164.1"

/codon_start=1

/transl_table=11

/product="Cps7C"

/protein_id="CUU53004.1"

/translation="MFQILQKHLPTLQRVLREGYSQHSLLAYWYGLSLLTALEQANHP
QVRKLAEKMINKGINIGHYFLAQSYFLCGEYDLAEQAVKKIKNFVKIPEVVFLYADIL
VKCKRKEEAWQLLEQCALLNKRKKVWIYLANLVNTIADFQRLEQHIEKVRTTTPHLKF
ELLIHQRTNAALRAGLTETALALTELNPLPKQAKVKKKTTAYNDKLAAIALADLKKVL
DHKKIPFFLISGTLLGCIREGKLLGHDKDIDIGVWDKYSYEELANCLSTSGYFYVVPT
RTNHLVMLRHVNGIAIDVFIHYRESNDYWHAGVKIKWHNSPFNLVYTNFLGQQYLIPE
NYDLYLTENYGDWRTPKTQFDSAFDTPNMEVINEVEMKVYISKIQK"

CDS 8441..11398

/gene="cps8D"

/locus_tag="MIDG2331_01766"

/inference="ab initio prediction:Prodigal:2.6"

/inference="similar to AA sequence:ABY70163.1"

/codon_start=1

/transl_table=11

/product="teichoic acid biosynthesis protein"

/protein_id="CUU53003.1"

/translation="MKFLKNSYHNVIAPKGYHRGLVLYRKKQWTEALSCFEAAYSTSP
LHAKNTFKLGLCHLKLGNFSEAHSFIAKALEIAPYNTHWRKQLQQAERHFNNTYSSPH
KITTVVTRMKQSGISQSIGTAIRKTVLLIPSDYNHRVMADISSFIQYYKDKFDVYIIL
RELPEDIVYKNTHVLVKNGTSFGEYLKFTADYVIDSGTMNYSYRITDTNKWVSVWHGI
PYKKMFVDFDIKNLATAIRYDLAYDSMVSMSNFYTDTFLRKAMRYDGEILQLGCAKID
NLFSSISTSNADKVNALRNELGLPNNKKVILYAPEFREVGELYFPFDPNKLLSHLGEE
YCLLTLLPFKGYIEQAENNIYYISDLDNKDALLIADLLISDYHELIYTFDRYNKPAVL
IQYDYESFVKQHTSRKQELEILASRKYVAKEANELYQFNWNLLKRYSKQSTLPEYLDS
SYIKHKLGIPFDKKIVLYAPTFRKAGAVQLPFDPNTLLNYLDNDYVLITKMHYLNYLA
NTYNGVIDCTSHENMAELMKIADILISDYSSLVLDFAVLNKPIILFQYDYDEYMKQRG
VYFNFGDYLPKEQIIRTEFELYTLNWNKLNSDNSKIINEFYPLEDGKSTQRIVDKINF
NADLRFSKDIIFLVNDLNQIGGVHSFLKNMAKYYKQKYNSRIYVIAIKEFAEANSEYH
LLESEYIDFKLSSQYLNGACANILQNTDGIVISLQFSAHMYFQKYLTNAKSVLMFHGD

VKDMISRELYGPHLDWLNKGKLYNYQKLLLLTQSALDLLKPHLNPEIQDKLGFMHNSI
DEEFSPIKQNKKHQLNTAVISRLDADKNIFAMIDLGKEILAQNSNVVVNIYGDGALKD
EFIAEITRHGLEHILKVRGFESNKSKIFSENNSLLLMSKSEGFPLVLLEAYAYGKPVI
VFDSFTAAKEIVKHNQSGFLLPYGDYGNVVKAIENSKNIKLKDIEMIFNNFSNPTVFA
KWDSLILALEQTA"

CDS 11411..12445

/gene="cps8E"

/locus_tag="MIDG2331_01765"

/inference="ab initio prediction:Prodigal:2.6"

/inference="similar to AA sequence:ABY70040.1"

/codon_start=1

/transl_table=11

/product="glycosyltransferase"

/protein_id="CUU53002.1"

/translation="MKLLKKLFGRNKLEQPLISILVPCYNSRKTLPATLKSIQQSNYK
NLDVMIVDDGHEVTVEDIVSSFNDPRFRYFYKKNEGLGLTRNFGIDNAKGEFIFFLDS
DDLIYPDAFSNLINYMLENNLDVVSGVTVRRDFETNVESEWCRALYRSKKISTFENRL
SLFDDALSTNKLYRLSMLREKDIRFETGLYEDKVFTAKLYSLVDRIGLIDNRVYIWFI
YGSQTSISTSKSVSNFKGRMAAINNLWQYIPEMRKTYQIAFYMNHDLLIYLREFEFYS
EEEKNEIYNIAYEFIHRHKKYIYNRLIPNSWNRTCLDALCEGNKEKFIYTANTLSKVF
QEELSRKQRV"

CDS 12470..13717

/gene="cps8F"

/locus_tag="MIDG2331_01764"

/inference="ab initio prediction:Prodigal:2.6"

/codon_start=1

/transl_table=11

/product="hypothetical protein"

/protein_id="CUU53001.1"

/translation="MSKTVIGIRTHQWTINEERLYKQLGEYFKSENIYFIVDETKEII
QFPQHINKISLNKELLTKIKILSNHPNPKGLGWLCGDYFYYAFKRNIQADYYWLIESD
VDFTFPNLGNFFQKFEQIEDDALLYNFGPAMNSWAWTQRGQLIDPTVYQAFFPLSRLS
GKAIDSCLTERQKLTHHFIENKVDLYQYPNDESLVATAVMKANLKVAKLNDFWKDSFK
FFTYRNQIIIPNAKNLIEKNQVLHPSRSPETFANTLNYEIVNLLSTSKSIPDLINRVF
VNPVDVEKIINQLKNKVLLDIEKLLKTRANYLYFLNFIKQLLDTKASEKSDKFAYKSW
VYRDSTLVLDVYINSENIICNTFTRKGNDQFIYKMSEKYDISSNENKLRIFDYSLCHD
KLNEEITNSLSLFFHSLEEFYFK"

CDS 13898..15598

/gene="cps8G"

/locus_tag="MIDG2331_01763"

/inference="ab initio prediction:Prodigal:2.6"

/codon_start=1

/transl_table=11

/product="hypothetical protein"

/protein_id="CUU53000.1"

/translation="MQLTHKSKIQVEIINGYKISYILKPSRKDIAHLVVLFNGYRHYG

WDFDKSINFFKCNSLMIVDIFKDEQSCYLGSSGELHFSDVVACLIERVLNRLYLTKDD

CTLLGASKGGFAALFIGIKYDFPNIVSSAPVAHIGSWMKNYNQNIATHVMGNNYNEDN

IAYYNNLLFTEIENSKNLNKSIYFFLSRNDHFYHEYGQKELLDKLESKYKNLNVFYTE

SELAFQHNQVTSYFLQEILSVTNLLSQKVYPTLNKHFIDDTSYNSIVLPNIRSKAILS

HRKHSILLKEESINKISMIKIIDGKLFVEGLLYIKNYNSETYKDLNKYISFNSIINNT

MTEYLLGTVPKIEQTRELYEDYLFNYSAAGTATLNFKGIDLSSLENGTYKLNISVTKS

NTDRDYKSLVLDKQINHKYIYKDCEYYLVSRKNDTFLTKRNILGDKNISSLFTLNSYW

VRESRFHIEGEYIIKGLAMPDFHIGNYYLVAKNLTSNETYSYLLGQVKKNDLSKKLND

IYGNYVSCYYATMKFEGIDTQIWENGEYELYVSLSHNNEIFSEKLNKKLLVNDKQCEF

"

CDS 15679..16590

/gene="cps8H"

/locus_tag="MIDG2331_01762"

/inference="ab initio prediction:Prodigal:2.6"

/codon_start=1

/transl_table=11

/product="hypothetical protein"

/protein_id="CUU52999.1"

/translation="MSLFFKKPNNKKLKLHINKIDNLHISENRLFFIEGLSYIEGVNS

PDYTYLSKNLKFINLATNVEFEYPLGITQKKDMSNTLYGDKYFDYTAAGTATMGFKGI

DVNHLEEGLYEVQISVSENKEERNYQSINFTAGHLDKYASNDYFEYRLFKNQNKIYLA

KRKLIGRNPISDYFISIEKEWIKEKTMHIEGAFVIPGIDITEFNQARYYLIAQKAITQ

KQYSFALGQIKKAGLGEKINNLQGSYNACYYATKMLKGIDMSALEFGFYDLYISLSYK

SEVFTVKLNKQLEIGHQLLKLVDNIEE"

CDS 17210..17323

/locus_tag="MIDG2331_01761"

/inference="ab initio prediction:Prodigal:2.6"

/codon_start=1

/transl_table=11

/product="hypothetical protein"

/protein_id="CUU52998.1"

/translation="MKKLLLAVLIASFGLAACGVKGPLYFPEQQPAQQQTK"

/locus_tag="MIDG2331_01760"

CDS 17454..17849

/gene="lysA_2"

/locus_tag="MIDG2331_01760"

/inference="ab initio prediction:Prodigal:2.6"

/inference="similar to AA sequence:ABY70103.1"

/codon_start=1

/transl_table=11

/product="diaminopimelate decarboxylase"

/protein_id="CUU52997.1"

/translation="MARLGSGFDIVSQGELERVLAAGGEPSKVVFSGVAKSHSEIQRA

LEVGIRCFNIESIAELHRINEVAGQLGKIAPISLRVNPDVDAHTHPYISTGLKENKFG

VSVTNEVNKYWEKRYEKKHRRKTYCVKVR"

CDS complement(18072..18614)

/gene="ydeN"

/locus_tag="MIDG2331_01759"

/inference="ab initio prediction:Prodigal:2.6"

/inference="similar to AA sequence:ABY70158.1"

/codon_start=1

/transl_table=11

/product="putative esterase of the alpha/beta hydrolase

fold protein"

/protein_id="CUU52996.1"

/translation="MKKVYVTHGYTANPTRNWFPWLKNELEKLGWECECLAMPNSDQP

NPQAWLEHHQNTLQLDENTLLIGHSLGCIALLNYLAVTQQKVKTAIFVSGFYEQLPHL

PELDEFANFYTNQTACLPEKSYVIAALNDVVVPHSFSDRLAQYLQADYIRLATGGHFV

DREGVTELPVLLELLKQILK"

ORIGIN

1 atgtcaaaca tcaacatcca gaatgcctta ttttcccttg ctcaacacaa taaactgtcg

61 attgaatcgc tggaaatcaa tactcacgat ttctgggtga ttgtcggcgg taacggctcg

121 ggtaaaactg ctttcgccca agcgctacat aattcacttt cactatattc gggcgaatat

181 caaaacagct tccaacatat cgctttactt tccttcgagc agcaacaaaa aatcatcgag

241 caaatcttta aacaccgtaa caacgatatg atttcaccgg atgatttcgg tttaactgcc

301 cgtcaaatta tcttaaacgg tagcgaaaaa acgcaattat gcgaagaata tgcagctaaa

361 ttacgtattc agccgttatt agatcgcccg tttattcagc tatccaccgg cgaaagtcgc

421 aaagtgctat tttgccaaat gttagttagc gaaccggatt tgctgatttt agatgagcct

481 tttgaagggt tagaccaagc ctcggtcgct tattggcaag atgttatggc acaactcggt

541 aagcaaatag cggtggtact gatttctaac cgctttaacg atattcccga ctgtgccaca

601 catattgctt tactggataa cttacaactg attttacaag gcgaacgcca agagattgaa

661 caacaagcgg tctattctca gctaaaattt gcagaacaga atgtgagtgc accgttgccg

721 gagagtgcca taccgctgat tcaactccca ccgaatacta atccgtttga actgaaaaac

781 gtaatgatcc gttacggcga aaaaacgatt attgatgatc taacttggac ggttgcccca

841 aaacaacatt ggtggattaa aggcccgaac ggagcaggaa aatcgacctt actttctatt

901 attgccggcg atcatccgca atcttacgct aattatgtgc atttattcgg tcgtcagcgt

961 ggttcgggcg aaacgatttg ggatataaag aaaaatatcg gctatgtgag cagccaatta

1021 catatggatt atcgggtgaa ttgctctgcg ttagacgtga ttttatccgg cttttttgat

1081 tcaatcggcg tttatcaaca agtaccgagt gccttacagc taaaagcaat ggaatggctg

1141 gaacgcttgc atttagccaa tctggcgaaa aaaccgttcc gttcactttc gtgggggcaa

1201 caacggttat tattgattac tcgtgctatg gtaaaacacc cgccgattct gattttagac

1261 gaaccgctgc aaggtttgga cggtgtaaac cgcaaattgg ttaaacaatt tatcgaacag

1321 cttgtgacta atagtcaaac ccagttgcta tttgtttcgc accaagatgc ggacgcccca

1381 aattgcatca cgcatttatt tgaatttgtt ccgcaagaga atggtggtta tcaatacgta

1441 cagacggtgt taaattagat tgttaacctt taaaggaaat ccccctcttt agtaaagagg

1501 ggttagggga gatttgtcaa tagagatatg aaattgaata gaacttcatt ttttatattt

1561 ataaaagcgt taattagcat atttcttcgc taatttattc tgtcaaatct cccccagccc

1621 ctctttgcta aagaggggag atgtatgtgg actattaaag cgttgagtta tagaacttat

1681 aagcatcgtc catattttcg aatttatgca taatgccttt atctaatacc atcgcattat

1741 cacaatattc cttcattgct gacggactgt gcgacactaa aatgatcgaa cgatctttgc

1801 gtttctcgaa taattcgtac ttacattttg ccgcaaagcg agagtcgcct accgcaatca

1861 cttcatcgat taagtaacaa tcaaactcca ctgaaagcga caaggcaaag gcaagtcggg

1921 ctttcatacc ggaggaatat ttcttaaccg gctcatataa ataatcgcct aattcggaaa

1981 attcctcggt aaaggcttta acgtagtcaa tatccgcatt atagatacgg caaataaagc

2041 gtaaattatc cataccggtt aaactgcctt gaaacgcccc gctgaaagcg agcggccaag

2101 atatcgacat gtgacgttcg atcgtaccgg ttgtcggcgg ctcaacaccg ctcattaagc

2161 gaattaaggt tgatttaccc gcgccgttac gccctaaaat accgattttt tcacctttat

2221 gtagatcaaa attaatattt tgtaacacgg tttttttacc gcttcgagta tagtaatctt

2281 tactcacatt ttttacgcta atcattgcgg ttcgattcct ttactgaagt ttttgaccat

2341 gattaagccg attaataaca tggctagatc acatattacg agataactta tgctttcata

2401 agtgataatg ttgtcaccga aataaccgtg gcgaaacatt tccgtgccgt gaatcatcgg

2461 tattaaggtt gcatattgtt gagcttggct tggtagcgca tgcacaaaga aaaatgcgcc

2521 tgaaagaggt aaaagaacaa agcttaatgt tccccagatt ttgccaaatg cttcaaattt

2581 ttgtgcaata gaacaaataa tcaagcctaa tcctaatgca aaaaatgcca ttaataccca

2641 cgccataacc atataaaacg tatctttcgg catttctatc cagcctaata aaatgactaa

2701 tgccataata atgatttggg caatcgttgc acccgctacc tcaagtatga cacgagccag

2761 taaggtatct aatacgcgaa cattacgatg ataaagaaga ctcaagttac cggaaattgc

2821 accgatagtg cggtttgatg cattacgcca catcattgcc attggataac cagtaatcac

2881 aaaagcaata atatttaaat cggaaacgcg atccgctcgg ataaatttcc acatcaaaac

2941 gataaataaa gtgagtaata gcggctcaac aaacagccat aaaaaaccca aattttttcg

3001 tccgtaacgc gtaataattt cccgcatgag taatgcaccg attactctcc cttgaatggc

3061 gagagattgg cggaaagttg tttgatcacc gtattgcatt agtttttgtg ctctcttacg

3121 cttgcaatta ataaacttaa tacaccataa agcatcagac cgataaagaa tgtcgctaaa

3181 atattatata agcgataagg ctcttccgcc cagtccggtt tgcttggctg actgattact

3241 tctaaataaa gttgctggcg atccgcttca tttttcgtat tttgtaatga ggttaatgct

3301 gcggtcaatt gttgctgtgc cagctcgttt gcaagtacta agcgttgata atcggcagtt

3361 tgagtagcaa tcgagctatt actgttaccg gaaagctgtt ttgattgctc atcgatttcc

3421 ttacgtaaac ttttttggcg cataagcaat gcatcaactt gtgggttgtc cggtgtaata

3481 gattgcaatt gagccaattg tgtttctaca cgaatcaatt cgctttttag gctggatatt

3541 aatgaaagtt gtacgccgga ttgtgccggt aaatcaaaga ttttattttt gatacgatat

3601 ttacttaagt cgcttgccgt tgcgtttaca ttattttccg cttccttaac cgcttgttcc

3661 gcaaatgaaa tggtatcttt tcttgcacgt tcgtttaaac ggttgatgag tgtttcacct

3721 tcggcaagta atttttgatt aatttgttgt ccctcttctg cattaaaagc acgaatacgt

3781 aagctggcaa taccggatac agaatcgaaa tcaacactta agcgatctcg gaaatatttg

3841 taaaacgctt ctttactatt atttaaacca aatccattaa agcgagcgat aatatcgcct

3901 tgattctcat agtattcacg tattggtagg tctttcatta actgttctaa tgccgtacga

3961 gaatgcatat attcttgtac ggtataagta tcatcttgag cacgagaaaa tccggaacct

4021 tgtaataagg ccccgacacc ggttaaagcg gtctgatttt taggcgatct aacgacaaaa

4081 cttgattccg aaatataaat atcagaagcg accgaaccga aataaaacgc tgatagcacg

4141 gtagggattg cgacagttat ccaaaataat ggattgagtt ttttaaagcg gctttttttc

4201 tgtttaaccg gtttctgtaa tttttctgct ggactggtag caataggtgt ttccatcttt

4261 tgtccttata cattcaatat attaataggc acgaacggca ttggtcgtac tggtaaccgg

4321 cgaagtaatt gagaaaatca ttctcaagaa tttttggaat tcagacaacg gcgcatttga

4381 aacatacaca atatctttat cttgcattgg gaaacgctgt aataaaaaca tggattgcgg

4441 ctcaagtaag ttcacacgat aaaccgttgg tacatccatt cctatagcgt agcctttagc

4501 ttgccattgt gcttgttgtt ccaaactcaa ttgtgcaaaa ggcacgtgac ggaatacgaa

4561 aacccctctc ggatccgaac gagtatcaat taaaccgccc atcttaccga tagcttcggc

4621 aagcgtaatt cctttacttg agaatttcat ttgctggttg ttacccacag cgcctaaacc

4681 ggtaaaacta taaggcgtgt ttagcaacga aacaacatca ccagcacgta acataatatt

4741 ttgtgccgga tcggaaatta acgtttcgaa tgcgagtgtt tttacttcag aaccacgggt

4801 tagcttgacc gtcacatctt caatgttttc cgttgttccg cctactgcag caaccgcatc

4861 taatacacgt tcattattag cggttaatgg catacgaata ctattgcctt gacgaataac

4921 cgtaacatca gcagagttat tattcgcaat tttgactaat gcttgcggat gattcgcttt

4981 gcgctgtagt gctccaataa tttgagactg aatcgcttcc ggtgttttgc ctgcgacacg

5041 aatgttaccc acgaacggca cggtaaccgt accgttttga ttaaccattt gtgccggtaa

5101 ttgcgttaaa tgcccgctac cttgtccctc agaactaaaa gtaccgccaa acagcaccgc

5161 cggcggagct tcccaaattg atatttcaag tacatcaccc acattgaccg caccggcata

5221 gcccgcgctg cctactgtgc ctaaaaatcc ggaaaattgt tggctttgct gagtttgata

5281 caactgttga actaaaccgt tatccagttc caccacattt acttccggta agggtttatc

5341 cgaactttgt gaattagcct ctaagatcgc actatggcta gggcctgaag ttgggaggct

5401 tgagcaagca gaaaggaaca atgttgttaa agctaaccct atgattgaat tatatttttt

5461 aatttccatc ttgtaaaaag gctctattga aaaagtgtgt aaaatgaata taaactatac

5521 ataattatag atacaatgct ataattatat attatagtct aatatctcgt tttttaaaac

5581 gataacctaa aatattactt ttctatagaa gaataggcat tattttaata aatataatta

5641 ggtatgaatg ttgatgaaaa tagcatttat cgcttggaat agttttcagg tgttacattt

5701 caagccctta ttacaagctt taccgtgtgc attattaatt attgaaaaac ggagacgtag

5761 tgtaccaatc tgtaaggata ttttgcgaga tataaacaat aatatcgctt atatccgcca

5821 tacggatata tatgcaaaaa ttgatgggaa ttttgatgtt ttagttgcgc aaacggcttt

5881 tgaacaactt tatttatttc gccacacaaa aattgctttg cttcaatatg ggtatgctaa

5941 ggagcctcat aactatggta cttggagagc attagcagat ctaaatttgg tttatgggag

6001 ttacgcctat gaacgaattt cttatttttc tccgactgaa ataacaggct gtccgcgata

6061 tgatttatgg tatttacctt catttcatca gaaagcgaaa gaaaattatg caaaagtatt

6121 agatacgaat aagaaaacga ttgtctatgc gccgagttgg ggagaattat ccagcctccc

6181 attgtatata gaagaaatca agaaattatc ttcatcttac aacgtgttag taaaattaca

6241 ccataacacg cttttattag ctaataaaca tcagaattat gaaagtttgt atccgaattt

6301 acattttttc tatgaaagtg aagatcttct atcacttatt tcggtagctg atattgttat

6361 ttctgatttt agcggagcaa tctttgatgc tattttttgt aaaaaaacag tagtgttact

6421 ttcgatatcg ctagtgaatc aaccaaaact agataaattt agtttagaga tagcttatcg

6481 ttcaaagtta ggatatgagg ttttttcgcc ggatcaagta gctataacag tggcacgagc

6541 acttacagag ccgaaattag tagatgaaac gctgtatcaa cagcttttta tgcataacaa

6601 ggatgcaaca cagcaagtaa taaatgcttt acaacagctt gctgagggta agtatacatt

6661 atctcaacag cagttatatg tgcgacaaac agaaaaaatta ttaaatattg aaaaaataaa

6721 gcagcaaaaa aataaaaaac agtctttcaa taaaataaga cagatttcta aaagattaat

6781 taaaaaataa tttttcatat tttcttttta tattttttta tttgacaata cggagtttag

6841 gaatgaaaaa agtattaact tatggaactt ttgatttgtt acatcatgga catattcgtt

6901 tattagaaag agcgagatca ttaggagatc atctcaccgt tgccatttcg acggaccagt

6961 ttaatctagg taaaggaaaa gtatgtgctt atacttacga agagagagca catattttaa

7021 aagcaatacg ttatgttgac gaagtaattc ctgaaactaa gtgggaacaa aaaatcgatg

7081 atgtaaaaaa tcatgaaatc gatgtatttg ttatgggggga tgattgggaa ggtaaattcg

7141 acttcttagc agattattgc gaagtagttt atttacctag aactcctgat atttcaacca

7201 ctcaagtaaa aaaaatgctc gcgaaaaaag atctcgcaac cggacaaaaa caaattcacg

7261 aaaaagagta atttgttaat gtttcaaatc ctacaaaagc atttaccgac cttgcaaaga

7321 gttctgaggg agggttactc ccagcattct ttgcttgctt attggtatgg gcttagtcta

7381 cttactgctc ttgaacaggc gaatcatcct caagtaagaa aactggctga gaaaatgatc

7441 aataaaggta ttaatatcgg gcattatttt ttagcacaaa gttatttctt atgtggagaa

7501 tatgatttag cggaacaagc ggtcaaaaaa atcaaaaatt ttgtaaaaat acccgaagtt

7561 gtttttttat atgcggacat tctcgttaaa tgcaaacgta aagaagaggc ttggcaatta

7621 ttagaacaat gcgctttact taataaaaga aaaaaagttt ggatatatct ggcaaattta

7681 gtaaatacta ttgcggattt tcaacgttta gaacagcata ttgaaaaagt aagaacaacg

7741 acacctcacc taaaatttga attattaatt caccaaagaa caaatgcagc attaagggct

7801 ggtttaacag aaacggcatt agcactaaca gaacttaacc ctttgccaaa gcaagcaaaa

7861 gtgaagaaaa aaacaaccgc ttataatgat aaattagcgg caattgcact agcggatctc

7921 aaaaaagtat tagatcacaa aaaaatacct ttctttctga ttagcggtac tctgctaggt

7981 tgtattcgag aaggaaaatt attagggcat gataaagata ttgatatcgg cgtttgggat

8041 aagtactcct acgaagaatt agcaaactgt ttatctacat cgggatattt ttatgtagta

8101 ccaacccgca caaatcattt agtcatgtta agacacgtta atggtatcgc aattgatgtg

8161 tttatccatt atcgcgaatc taatgattat tggcacgcag gtgtcaaaat aaaatggcat

8221 aactcgccat tcaacttggt atatacaaat tttctcggac aacaatattt aatacctgaa

8281 aattacgatt tatacctaac agaaaattac ggtgattggc gtacgccaaa aactcaattt

8341 gatagtgcat ttgatacgcc aaatatggaa gttatcaatg aagtggaaat gaaggtttat

8401 atatctaaaa tacaaaaata ataaaataag gaataaatga atgaaatttt tgaaaaactc

8461 ttatcataat gtgattgctc ctaaagggta tcatcgagga ttagttttat acagaaaaaa

8521 acaatggact gaagctttat cttgctttga agctgcttat agcacatctc ctttacatgc

8581 caaaaatacc tttaagctag gattatgtca ccttaaatta gggaatttct cagaggctca

8641 ttcatttatt gctaaagctc ttgaaatagc tccatataat acgcattgga gaaagcagct

8701 ccaacaggct gaacgccact ttaataacac atactcatct ccacataaaa tcactactgt

8761 tgtaactaga atgaaacaaa gtggtattag ccagtctatt ggtaccgcta tcagaaaaac

8821 agtactactc attccatcag actataacca tcgagtaatg gctgatattt catcatttat

8881 ccaatactat aaagataaat ttgacgtata tattatatta cgtgaattac ccgaagatat

8941 tgtgtataaa aatactcatg tattagtcaa gaatggaact tcatttggtg aatatttaaa

9001 atttactgca gattatgtta ttgactcagg cacaatgaac tatagctacc gtattactga

9061 tactaataaa tgggtttcgg tatggcacgg tattccttat aaaaaaatgt tcgttgactt

9121 tgacattaaa aatctagcaa cagcaatccg ctatgatctt gcctatgaca gcatggtttc

9181 aatgtctaat ttttatactg atacattttt acgtaaagca atgcgctatg acggtgaaat

9241 attacaactt ggttgtgcca aaattgataa tttattttca tctatttcta caagcaacgc

9301 cgataaagtc aatgccttac gtaatgaatt aggtttacct aataataaaa aggttatttt

9361 atatgctcct gaatttagag aagtaggtga actttatttc ccatttgatc ctaataaatt

9421 attatctcat ttaggtgaag agtattgttt acttacttta ttacctttta aaggatatat

9481 cgaacaagca gaaaataata tctactatat ttccgattta gataataagg atgccctttt

9541 aattgctgat ttattaatta gtgattatca cgaattaatc tatacatttg atagatataa

9601 taagcctgca gttctaatac agtacgacta tgaatcattt gtaaaacaac atacttccag

9661 aaaacaagag ctagaaatac tagcatctag aaaatatgtt gcaaaagaag cgaacgaatt

9721 atatcaattt aattggaatc tactaaaaag atatagtaaa cagtctactt tacccgaata

9781 tcttgatagc tcatatataa agcataaatt aggtattcca tttgataaga aaattgtttt

9841 atatgcacca actttccgca aagctggtgc cgtacaattg ccttttgacc caaatacatt

9901 actaaattac ttagataatg attatgtatt gatcacaaaa atgcattacc taaattattt

9961 agctaataca tataacggcg ttattgactg tacctcacat gaaaatatgg cagagctaat

10021 gaaaatcgct gatatcttaa tcagcgacta ttcatcatta gttcttgact tcgctgtatt

10081 aaataaacca atcattctat tccagtatga ttatgatgaa tatatgaaac aacgaggagt

10141 ttacttcaat tttggagatt atttacctaa agagcaaatt attcgaactg aatttgaatt

10201 atatacatta aactggaata agcttaactc ggacaatagt aagattatta atgaatttta

10261 tccacttgaa gatggaaaat ccacccagcg tattgtagat aaaataaatt ttaatgcaga

10321 tttaagattc agcaaggata ttattttctt agtaaatgat ttaaatcaaa ttggtggtgt

10381 ccactcattt ttaaagaata tggcaaaata ctataagcag aagtataact ctcgcattta

10441 tgttattgct attaaagaat ttgcagaagc aaactctgaa tatcacttat tagaaagcga

10501 atatattgac tttaaattat ctagccaata tttaaatggt gcttgtgcta atattttaca

10561 aaatacagat ggcattgtaa tttcattaca gttttctgct catatgtatt tccaaaaata

10621 tttaaccaac gctaaatcag ttctgatgtt ccatggcgat gttaaagata tgatttccag

10681 agagctttat gggccacatt tagattggtt aaataaaggc aaactctata actatcaaaa

10741 gttattattg cttacacaat cagctttaga cttacttaaa cctcatttaa atcctgagat

10801 ccaagataaa ttaggattta tgcataattc tattgatgaa gaatttagtc caattaaaca

10861 gaataaaaaa catcaattaa atactgcggt aattagccgt ctagatgcag ataaaaatat

10921 ttttgcaatg attgaccttg gaaaagagat tcttgctcaa aattcaaatg ttgttgtaaa

10981 tatctatggt gatggagctt taaaagatga atttatagct gaaattactc gtcatggtct

11041 agaacatatt ttaaaagtaa gaggctttga aagtaataaa tctaaaattt tctcagaaaa

11101 taattcctta ttattaatga gtaaatcaga gggctttcct cttgttttac tagaagccta

11161 tgcttatggc aagcctgtga ttgtttttga ttcatttaca gctgctaaag aaattgtaaa

11221 acataatcag tcaggatttt tattacctta cggcgattat gggaatgtag ttaaagccat

11281 tgagaatagt aaaaatatta agttgaagga tattgaaatg atctttaaca atttctctaa

11341 tccaactgtg tttgctaaat gggatagctt aatcctagct ttagagcaaa cagcgtaaag

11401 gagatacagt atgaaactat taaaaaaatt attcggccgc aataaattag aacagccttt

11461 aatttcgatt cttgtacctt gttacaattc tcgtaaaaca ttgcctgcaa cattaaaatc

11521 tattcaacaa tcaaattata aaaacttaga tgtaatgatt gttgatgatg ggcatgaggt

11581 gactgtagag gatattgtta gttcatttaa cgatcctcgc tttcgttatt tttataaaaa

11641 aaatgaaggc ttaggcttaa ctagaaattt tggtattgac aatgcgaaag gcgaatttat

11701 cttcttttta gactcagatg atctaattta tcctgatgct ttctcaaatt taataaacta

11761 tatgctggaa aacaatttag atgtggtatc tggtgttaca gtacgtcgcg actttgaaac

11821 taatgtagaa agtgaatggt gtagggcatt atatagatct aaaaaaatta gtacatttga

11881 aaatagatta tccttatttg atgatgcttt atctacaaat aaattatacc gattatcgat

11941 gttaagagaa aaagatattc gttttgaaac tggattatat gaagataaag tttttactgc

12001 aaaattatat tctctagtag atcgtatagg gttaattgat aatagggtat atatctggtt

12061 tatatatgga tcacaaacaa gtatatctac atcaaaatcc gttagtaatt ttaagggaag

12121 aatggctgct attaataatc tatggcagta tattcctgaa atgcgaaaga cataccaaat

12181 agcgttttat atgaaccacg atttattaat ttatttacgt gaatttgaat tttactctga

12241 agaagaaaag aatgaaattt acaatatcgc ttatgaattc atacataggc acaagaaata

12301 tatctacaat agattaatac caaatagctg gaatagaact tgtcttgatg cgctatgtga

12361 aggaaataaa gaaaaattta tttatactgc aaatactcta tcaaaagtat tccaagaaga

12421 gttaagtaga aaacaaagag tctaatatta atctaaggag taaaaactta tgagtaagac

12481 ggttattggt attaggactc atcaatggac tatcaatgaa gaaagattat ataaacaatt

12541 aggggaatat tttaaatctg aaaatatcta ctttattgtt gatgaaacga aagaaattat

12601 tcaatttcct cagcatataa ataaaatctc tttaaataaa gagctattga ccaaaattaa

12661 aattttaagt aatcatccaa atcctaaagg cttgggctgg ctatgtggtg actactttta

12721 ttatgctttc aaacgaaata ttcaggctga ttactattgg ttaattgaat ctgacgtaga

12781 ttttacattt cctaatttag gtaatttctt tcagaaattt gagcaaattg aagatgatgc

12841 attattatat aattttggtc ctgcaatgaa ctcatgggct tggactcaaa gaggacaatt

12901 gattgatcct actgtttatc aagcattttt tccattaagc cgattatcgg gaaaagctat

12961 agattcttgt ttaacagaac gtcaaaagtt gacccatcat tttatagaaa ataaagtaga

13021 tttataccaa tacccaaatg atgagtcatt ggtagcaact gctgtaatga aagctaattt

13081 gaaggtagca aaattaaatg acttttggaa agatagcttt aaatttttca cataccgtaa

13141 tcaaattatt atacctaatg caaagaatct tattgaaaag aatcaggtat tacatccaag

13201 ccgttctcca gaaacgtttg ctaatactct aaattacgaa atagtgaatt tacttagtac

13261 ttctaaaagt attcctgact taataaatag agttttcgta aatcctgtag atgtggaaaa

13321 aataattaat caattaaaga ataaagtttt acttgatatt gaaaaactat taaagactcg

13381 tgcaaattat ctttattttc ttaactttat taaacagcta ttagacacaa aagcatctga

13441 gaaaagtgat aagtttgcat acaaatcatg ggtatatcga gattccactt tagtattaga

13501 tgtttacatt aattcagaaa atattatatg taatactttt acacggaaag gaaatgacca

13561 atttatatat aaaatgtcag aaaaatatga tatttcaagc aatgaaaata aattaagaat

13621 ttttgattat tcgttatgcc atgataaatt aaatgaagaa ataaccaatt cactttcatt

13681 attcttccat tcattagaag agttttattt taaataggaa tttacccaga cacataagaa

13741 atatatttat aatagattaa tccccaatag ctggaatata gcttgcttag atgcattata

13801 taagagatag agataaattc atctatattt ctaatatttt atctaaatta ttccaggagg

13861 agttaagtaa gaagcaaaag gtttaaaagg taaaatcatg caattaacac ataaatcaaa

13921 aatccaagta gaaattatta atggttataa aattagttat atccttaagc caagtcgaaa

13981 agatattgca catttagttg ttctttttaa tggatataga cattacggat gggattttga

14041 taaatctata aatttcttca aatgtaattc tttgatgatt gtagatatat ttaaggatga

14101 gcaatcctgt tatttaggca gttctggaga acttcatttt tctgatgtag ttgcttgttt

14161 aatagaaaga gtattaaatc gtttatatct tactaaagat gattgtactc ttcttggagc

14221 ttctaaaggt ggatttgcag ctttatttat tggaataaaa tatgattttc caaatattgt

14281 atcttctgct ccagtagccc acattggctc atggatgaaa aattataatc aaaacatcgc

14341 cacacatgtt atgggaaaca attataatga agataatatc gcatattata ataatttatt

14401 attcacagaa atagaaaact caaagaattt aaataaatca atatacttct ttttatcaag

14461 aaacgatcat ttttatcatg aatatggaca aaaagaatta ctcgataaat tagaatctaa

14521 atacaagaat ttaaatgttt tctatacaga atctgagtta gcatttcaac ataatcaagt

14581 aacttcttat tttttacaag aaatactatc tgtaacaaat ttattatcac agaaagtata

14641 cccgacttta aataaacatt ttattgacga tactagctac aatagtattg tactgcctaa

14701 cataagaagc aaagcaattc ttagtcatag aaaacattct atcttattaa aagaagaaag

14761 cattaataaa attagcatga tcaagattat agatggaaag ctgtttgtag aaggactatt

14821 gtatataaaa aattataatt ctgaaactta taaagactta aacaaatata tttcttttaa

14881 tagcattatt aataatacaa tgacagaata ccttttaggt actgtaccta aaattgaaca

14941 aacaagagaa ttatatgaag attatttatt taattactct gcagcaggaa ctgcaacatt

15001 aaattttaaa ggcattgatt tatcatcatt agaaaatggt acttataaac taaatatctc

15061 tgtaacaaaa tcaaacactg atagagatta taaaagctta gttttagata aacagatcaa

15121 tcataaatat atttataaag attgtgaata ttatttagtt agtagaaaaa atgatacttt

15181 tcttaccaaa agaaatatat tgggagataa aaatatttcg tccctattta cattaaacag

15241 ttattgggtg agagaaagta ggtttcacat cgaaggtgaa tatatcataa aagggcttgc

15301 catgcctgat tttcatattg gaaattatta tttagtagct aaaaatctaa cgagtaatga

15361 aacatattct tatttattag gacaggtaaa aaagaatgac ttatctaaga aattaaatga

15421 catatatggt aattatgtgt cctgttatta tgctaccatg aaattcgaag gcatagatac

15481 ccagatatgg gagaatggag aatatgagtt atatgtatcg ttgagtcata acaacgagat

15541 ttttagtgaa aaactaaata aaaaattatt agttaacgat aaacaatgtg aattttaata

15601 ttcactttat cataaatctt atataagaca ataaatttat ttatggtcta taattgaaat

15661 tattactagg aatatagcat gagtttattt tttaagaaac caaataataa aaaactaaaa

15721 ttacacataa acaagataga taacctacat atttcagaaa ataggttatt ttttattgaa

15781 ggattgtctt atatagaagg ggttaattcc cctgactata cttatttatc aaaaaaattta

15841 aagtttatta acttagcaac aaatgttgag tttgaatatc cccttggtat aacccagaaa

15901 aaggacatgt caaatacatt atatggagat aaatattttg actatactgc agcaggtact

15961 gcaacaatgg gatttaaagg tatagatgtt aaccatcttg aagaagggtt atatgaagtt

16021 cagatttctg tatctgaaaa taaagaagaa cgaaattatc aaagtatcaa ctttactgca

16081 ggtcatttag ataaatatgc ctcaaatgat tattttgagt atcgcttatt taaaaatcaa

16141 aataagattt atttagcgaa aagaaagtta atcggaagaa atcctatttc tgattatttt

16201 attagtattg aaaaagaatg gatcaaagaa aaaacaatgc atattgaagg tgcatttgta

16261 atccctggta ttgatattac tgaatttaat caagccagat actatttaat tgcccaaaaa

16321 gcgattacac agaagcaata ctcctttgca ttaggacaga tcaaaaaagc tggactagga

16381 gaaaaaatca ataatctaca aggttcttat aatgcctgct attatgcaac aaaaatgcta

16441 aaaggtattg atatgagtgc gctagaattc ggattctatg atttatatat ttctttaagc

16501 tacaaaagcg aagtctttac tgttaaatta aataaacagt tagaaattgg acatcaactc

16561 ctgaaattag ttgataacat tgaggagtag gctcttatct taacgaaaga tagttattac

16621 tcttcctaag agacttccca aaaaagcggt caaatttttc taaaaaattg caaagtttag

16681 gaaaaataag accgcttatt ttattcatac tcttatataa aattagagtt tattatgggc

16741 gtagccacgt acaatggtaa ggatatgctg tatagcaata ttaatacaat cctatcaatt

16801 tcatattatc ttaatcagat ttatttttc gttttttaga cattaagctg ttattttctt

16861 cgttatttaa ttctgccaaa tctcccctat cccctcttta ctaaagaggg ggatttcctt

16921 tagatattaa caagctatga tctttcgaat agctttggtc tctcaataga ttcaggtgaa

16981 tgatcgatat ataaggaaaa taaaaacatt cgtcgaagac gagcgccatc gtaaagaaat

17041 ttggagccac agcatggctc actcagccgt aggctgatcg taaccacgta cggcttgccg

17101 tgcgtggctg ttgggtgatt atttaaaaac aggcgaaacg gcttttttac tttttcatta

17161 ccgcaatttg ttttagaatc aacggacgat ttactctcaa ggatttatga tgaaaaaatt

17221 acttttggcg gtgttaatcg catcgttcgg gttggctgcc tgcggtgtaa aaggcccgct

17281 ctattttccc gagcagcaac cggctcaaca acaaacaaaa taattgctaa ccacggtaat

17341 tcaaaatcta cgtataacaa gcggtcggat ttatccgatt ttttgtaaat acaagtcatc

17401 aatcaatacg gtacaccggc ttatatctat tctcgtgcta cgcttgagcg tcattggcac

17461 gcctcggttc gggctttgat attgtgtcgc aaggcgaact tgaacgtgta cttgccgccg

17521 gcggcgagcc gagcaaagtg gtattttccg gtgtggcaaa atcacatagc gaaattcaac

17581 gtgcattgga agtcggcatt cgttgtttta atatcgaatc catcgccgag ttacaccgca

17641 ttaatgaagt tgccggtcaa ttaggtaaaa tcgcaccgat ttcattgcgt gtaaatccgg

17701 atgtggatgc acatactcac ccttatattt ccaccggttt aaaagaaaat aaatttgggg

17761 taagcgtaac gaacgaggta aacaagtact gggagaagcg ttacgagaaa aaacacaggc

17821 ggaaaaccta ctgcgtgaaa gtgcgctaaa ttggacgatt ctccgcccgt gcgggctgaa

17881 tacggacgaa ggcgaaacct ttcgtttaat tgaaaatgcg gctgaactgc ccggcagtta

17941 tatgagccgt aaagcattag ccaatgcggt cttgtccgta cttaacagtg aaaacacaaa

18001 ctataaaatc ttctcagtct gtgcctaact tcacaatccc tttcactttg gcacaagcca

18061 tccgcttgtg cctatttcaa aatttgtttt aacagttcca ataaaaccgg taattccgtt

18121 actccctctc gatcgacaaa atgcccgccc gttgccaagc gaatataatc cgcttgtaag

18181 tattgcgcta atcgatcgct gaacgaatgg gggacgacaa catcatttaa tgcggcaatc

18241 acataagact tttctggtaa gcaagcggtt tgatttgtat aaaaatttgc aaactcatcc

18301 aattccggta aatgcggtaa ttgttcataa aaaccggaaa caaaaattgc cgtttttact

18361 ttttgctgcg ttaccgccag ataattcagt aacgcaatgc agcccaaact atgtccgatg

18421 agtaaggtat tttcatctaa ttgaagtgta ttttggtgat gttccagcca tgcttgcgga

18481 ttcggctgat cggaattcgg catcgctaaa cattcacatt cccatcctaa tttttccaat

18541 tcgtttttaa gccacggaaa ccaatttctt gtcgggttcg ccgtataacc gtgcgttaca

18601 tatacttttt tcat

//

LOCUS **ADOI01000049.1** 15315 bp DNA linear BCT 20-APR-2021

DEFINITION Actinobacillus pleuropneumoniae serovar 9 str. CVJ13261
capsular polysaccharide gene locus, complete sequence.

ACCESSION ADOI01000049 REGION: complement(37672..52986)

VERSION ADOI01000049.1

KEYWORDS .

SOURCE Actinobacillus pleuropneumoniae serovar 9 str. CVJ13261

ORGANISM Actinobacillus pleuropneumoniae serovar 9 str. CVJ13261
Bacteria; Proteobacteria; Gammaproteobacteria; Pasteurellales;
Pasteurellaceae; Actinobacillus.

REFERENCE 1 (bases 1 to 15315)

AUTHORS Xu,Z., Chen,X., Li,L., Li,T., Wang,S., Chen,H. and Zhou,R.

TITLE Comparative genomic characterization of Actinobacillus

pleuropneumoniae

JOURNAL J. Bacteriol. (2010) In press

PUBMED 20802045

REMARK Publication Status: Available-Online prior to print

REFERENCE 2 (bases 1 to 15315)

AUTHORS Xu,Z., Zhou,R. and Chen,H.

TITLE Direct Submission

JOURNAL Submitted (04-MAY-2010) College of Veterinary Medicine, Huazhong

Agricultural University, Shizishan Street 1, Wuhan 430070, China

FEATURES Location/Qualifiers

source 1..15315

/organism="Actinobacillus pleuropneumoniae serovar 9 str.

CVJ13261"

/mol_type="genomic DNA"

/submitter_seqid="contig00033"

/strain="CVJ13261"

/serovar="9"

/note="K locus: KL09"

/db_xref="taxon:754258"

/note="Coverage of the contig is 16.28X"

CDS 1..1458

/gene="modF"

/locus_tag="appser9_17200"

/note="similar to ABC-type molybdenum transport system,

ATPase component/photorepair protein PhrA COG1119;

similar to ABC transporter related protein of Mannheimia

haemolytica serotype A2 str. OVINE UniRef

RepID=UPI0001BCFBC9"

/codon_start=1

/transl_table=11

/product="ABC transporter protein"

/protein_id="EFM93524.1"

/translation="MPNINIQNALFSLAQHNKLSIESLEINTHDFWVIVGGNGSGKTA

FAQALHNSLSLYSGEYQNSFQHIALLSFEQQQKIIEQIFKHRNNDMVSPDDFGLTARQ

IILNGSERTQLCEEYAAKLRIQPLLDRPFIQLSTGESRKVLFCQMLVSEPDLLILDEP

FEGLDQASVTYWQEVMAQLGKQMAVVLISNRFNDIPDCATHIALLDNLQLILQGERQE

IEQQAVYSQLKFAEQNVNAPLPESATPLIQLPPNTNPFELKNVMIRYGEKTIIDDLTW
TVAPKQHWWIKGPNGAGKSTLLSIIAGDHPQSYANYVHLFGRQRGSGETIWDIKKNIG
YVSSQLHMDYRVNCSALDVILSGFFDSIGVYQQVPSALQLKAMEWLERLHLANLAKKP
FRSLSWGQQRLLLITRAMVKHPPILILDEPLQGLDGVNRKLVKQFIEQLVTNSQTQLL
FVSHQDADAPNCITHLFEFVPQTNGGYRYVQTALN"

CDS complement(1517..2167)
/gene="cpxA"
/locus_tag="appser9_17190"
/note="similar to ABC-type polysaccharide/polyol phosphate
transport system, ATPase component COG1134;
similar to ATP-binding protein bexA of Bacteria UniRef
RepID=BEXA_HAEIN"
/codon_start=1
/transl_table=11
/product="ATP-binding protein bexA"
/protein_id="EFM93523.1"
/translation="MISVKNVSKDYYTRSGKKTVLQDINFELKKGEKIGILGRNGAGK
STLIRLLSGVEPPTSGTIERNMSISWPLAFSGAFQGSLTGMDNLRFICRIYNADIEYV
KAFTEEFSELGDYLYEPVKKYSSGMKARLAFALSLSVEFDCYLIDEVIAVGDSRFAAK
CKHELFEKRKDRSIILVSHSPSAMKSYCDNAMVLDKGIMYKFENMDEAYKFYNSTL"

CDS complement(2164..2961)
/gene="cpxB"
/locus_tag="appser9_17180"
/note="similar to ABC-type polysaccharide/polyol phosphate
export systems, permease component COG1682;
similar to Capsule polysaccharide export inner-membrane
protein bexB of Proteobacteria UniRef RepID=BEXB2_HAEIN"
/codon_start=1
/transl_table=11
/product="Capsule polysaccharide export inner-membrane
protein bexB"
/protein_id="EFM93522.1"
/translation="MQYGDQTTFRQSLAIQGRVIGALLMREIITRYGRKNLGFLWLFV
EPLLLTLFIVLMWKFIRADRVSDLNIIAFVITGYPMAMMWRNASNRTIGAISGNLSLL
YHRNVRVLDTLLARVILEVAGATIAQIIIMALVILLGWIEMPKDTFYMVMAWVLMAFF
ALGLGLIICSIAQKFEAFGKIWGTLSFVLLPLSGAFFFVHALPSQAQQYATLIPMIHG
TEMFRHGYFGDSVITYESISYLVICDVAMLLFGLIMVKNFSKGIEPQ"

CDS complement(2961..4118)
/gene="cpxC"

/locus_tag="appser9_17170"

/note="similar to Capsule polysaccharide export protein

COG3524;

similar to Capsule polysaccharide export inner-membrane

protein ctrB of Proteobacteria UniRef RepID=CTRB_NEIMA"

/codon_start=1

/transl_table=11

/product="Capsule polysaccharide export inner-membrane

protein ctrB"

/protein_id="EFM93521.1"

/translation="METPIATSPAEKLQKPIKQKKSWLKKLNPLFWVTVAIPTVLSAF

YFGSVASDIYISESSFVVRSPKNQTALTGVGALLQGSGFSRAQDDTYTVQEYMHSRTA

LEQLMKDLPIREYYENQGDIIARFNGFGLNNSKEAFYKYFRDRLSVDFDSVSGIASLR

IRAFNAEEGQQINQKLLAEGETLINRLNERARKDTISFAEQAVKEAENNVNATASDLS

KYRIKNKIFDLPAQSGVQLSLISSLKSELIRVETQLAQLQSITPDNPQVDALLMRQKS

LRKEIDEQSKQLSSNSNSSIAIQTADYQRLVLANELAQQQLTAALTSLQNTKNEADRQ

QLYLEVISQPSKPDWAEEPYRLYNILATFFIGLMLYGVLSLLIASVREHKN"

CDS complement(4144..5331)

/gene="cpxD"

/locus_tag="appser9_17160"

/note="similar to Periplasmic protein involved in

polysaccharide export, contains SLBB domain of b-grasp

fold COG1596;

similar to Capsule polysaccharide export protein bexD of

Proteobacteria UniRef RepID=BEXD_HAEIN"

/codon_start=1

/transl_table=11

/product="Capsule polysaccharide export protein bexD"

/protein_id="EFM93520.1"

/translation="MEIKKYNSIIGLALTTLFLSACSSLPTSGPSHSAILEANSQSSD

KPLPEVNVVELDNGLVQQLYQTQQSQQFSGFLGTVASAGYAGAVNVGDVLEISIWEAP

PAVLFGGTFSSEGQGSGHLTQLPAQMVNQNGTVTVPFVGNIRVAGKTPETIQSQIVGA

LQRKANQPQALVKIANNNSADVTVIRQGNSIRMPLTANNERVLDAVAAVGGTTENIED

VTVKLTRGSEVKTLAFETLISDPAQNIMLRAGDVVSLLNTPYSFTGLGAVGNNQQMKF

SSKGITLAEAIGKMGGLIDTRSDPRGVFVFRHVPFSQLSLDQQAQWGSKGYGMGMDVP

TVYRVNLLEPQSLFLLQRFPMQDKDIVYVSNAPLSEFQKFLRMIFSITSPVTSTTNAV

RAY"

CDS 5510..6652

/gene="cps9A"

/locus_tag="appser9_17150"

/note="similar to Cps2A of Actinobacillus pleuropneumoniae

UniRef RepID=B0BRQ7_ACTPJ"

/codon_start=1

/transl_table=11

/product="Cps2A"

/protein_id="EFM93519.1"

/translation="MLMKIAFIAWNSFQVLHFKPLLQALPCALLIIEKRRRSVPICKD

ILRDINNNIAYIRHTDIYAKIDGNFDVLVAQTTFEQLYLFHRTKIALLQYGYAKEPYN

YGTWRAFADLNLVYGNYAYERISYFSPTKITGCPRYDLWYQPLFHQKAKENYARVLDT

SKKTIVYAPSWGELSSFKLYIEEITKLSLFYNVLVKLHHNTLLLANKHQNYEKLYPNL

HFFYEGEDLLSLISVADIVISDFSGAIFDAIFCKKTVVLLSISLVNQPKLDKFSLEIA

YRSKLGYEVFSPDQVAITVARALTEPKLVDETLYQQLFMHNKDATQQVINALQQLAEG

KYTLSQQQLYVRQTEKLLNIEKIKQQKNKKQSFNKIRQISKRLIKK"

CDS 6705..7133

/gene="cps9B"

/locus_tag="appser9_17140"

/note="similar to Cytidylyltransferase COG0615;

similar to Glycerol-3-phosphate cytidylyltransferase of

Bacteria UniRef RepID=TARD_BACSU"

/codon_start=1

/transl_table=11

/product="Glycerol-3-phosphate cytidylyltransferase"

/protein_id="EFM93518.1"

/translation="MKKVLTYGTFDLLHHGHIRLLERARSLGDHLTVAISTDQFNLGK

GKVCAYTYEERAHILKAIRYVDEVIPETNWEQKVEDVKNHEIDVFVMGDDWEGKFDFL

ADYCEVVYLPRTPDISTTQVKKMLAKKDLAAGQKQIHEKE"

CDS 7213..8286

/gene="cps9C"

/locus_tag="appser9_17130"

/note="similar to Cps7C of Actinobacillus pleuropneumoniae

UniRef RepID=B0BRQ5_ACTPJ"

/codon_start=1

/transl_table=11

/product="Cps7C"

/protein_id="EFM93517.1"

/translation="MLAYWYGLSLLTALEQANHPQVRKLAEKMINKGINIGHYFLAQS

YFLCGEYDLAEQAVKKIKNFVKIPEVVFLYADILVKCKRKEEAWQLLEQCALLNKRKK

VWIHLTNLVNTEADYRHLEQHIDKVRTTTPYLKSDLLIHQRTNAALRAGLTETALALT

ELNPLPKQAKVKKKTTAYNDKLAAIALADLKKVLDHKKIPFFLISGTLLGCIREGKLL
GHDKDIDVGVWDEYSYEELANYLATSGYFYVVPTRTKHLVMLRHVNGIAIDVFIHYRE
PNDYWHAGVKIKWHNSPFNLVYKDFLGQQYLIPENYDLYLTENYGDWRTPKTKFDSAF
DTPNMEVINEAEMQIYVIKKREL"

CDS 8261..11704

/gene="cps9D"

/locus_tag="appser9_17120"

/note="similar to Putative glycosyl/glycerophosphate
transferases involved in teichoic acid biosynthesis
TagF/TagB/EpsJ/RodC COG1887;
similar to Teichoic acid biosynthesis protein of
Actinobacillus pleuropneumoniae serovar 3 str. JL03 UniRef
RepID=B0BRQ4_ACTPJ"

/codon_start=1

/transl_table=11

/product="Teichoic acid biosynthesis protein"

/protein_id="EFM93516.1"

/translation="MLLKSENFKMKHNVKLSSLPSLLQGLYLYKHHQYQKAQKIFSKI
LEKQPKNAYLNFRYGMSLYKDKKWNEANYFIQKAVELAPEQESWKKQLATTERYKNDS
SKIKVAENKKNLEKQPNSPEYIWEYAVSLIDSKQYWLAQFQLEKYIELRPDSERAFYQ
LGIVAEKLANYEQAFSYFQKASQFDPLNRNYKYRMGYNLEKLGHLDQARMCYDLVTSL
SNAGDEVIDFGIGALHAKRGLWDLAVSAYLEFQVKTGSQNPELFYRIGLANERLYQWA
ESATAFEQAVTLSEVINANWCFKCGQAYERAGNFEKSAEFYQEAVKRSDHYNDYWWYR
LALVLEKLGKYEQSVSAFQNSRRRKLAYSVNPKNVIKHKEEEYLSYYTEYYETLELDE
KLVLIESFFGGNISCNPYAILSYMLGNNYDYTYVVVIKDGTVIPDNLKFNRKIIFIKR
GSDAYLRYLCTAKYLINNVSFPYYFIRKEGQIYLNTWHGTPMKTLGKDIKNPFMDHAN
VSRNFLQATHIISPNRHTTDIILEQYDVKDLFSGKLAETGYPRIDLAFNLTGKRREEI
KEKLGLSNKKPVVFYAPTWRGTSQSKDFDTTKLQSDLKKLKSDKYNLIFRGHHLVEQL
LETINLDVIVAPKDIDSNELLGFCDLLITDYSSIIYDFLALNKPAISYIYDYEEYDAE
RGLYLKPEEMSGTVCTTITDVKNAILENIALGKTNVSEQDINKYSYLDDGKATKRTVE
FMFDRDDSCVYKYERKKSDIFFEGPFIPNGISRSFLNLMASIKDSEKNITLLINGADI
AQDQKRLAEFDNLPSNVTVLSRVGRTPMTLEELWVRNKFEETYQMYSESFTETLLKVY
KREVRRLLGDSLFENAIHFEGYSLFWVLLFSQINAKKHIIYQHNDKYKEWKGRFPYLE
GVFNSYVFFDQIVSVSEKTMENNILNLSKSFNIPKEKFTFCNNPINIQQILSSAEEDI
EMESEFTSFNGQKFINIGRMSHEKDQLKLIEAFCEAKKVHANIRLFILGDGVLKQDLT
NKIKELSLEKDVYLLGQKKNPFPYLKQADVFILSSNHEGQPMVLLESLTLGTPIIATD
IVGNRSILGDNYGVLVENSKDGLVKGINIYMEQGGRKDSFDPYEYQNDAMAKFYSLLT
N"

CDS 11722..12762

/gene="cps9E"

/locus_tag="appser9_17110"

/note="similar to Putative uncharacterized protein of

Actinobacillus pleuropneumoniae serovar 3 str. JL03 UniRef

RepID=B0BRQ2_ACTPJ"

/codon_start=1

/transl_table=11

/product="hypothetical protein"

/protein_id="EFM93515.1"

/translation="MLDFFRIKFDQNTKCDELNIDLSVKEIYIDKSAIGKDEKEKLNH

NFLAVGRGNIEAKKLIAKLSNNKYYLTAHSNGISIFTKFSDGKSFISHFKNKNVKVWN

DTFYTLDEPLEKDTSINRLLVVFSSIADLAFNASIERRMFFTNFPSVGKYIPKNTYIL

RIADIGGVLGSFYLNNNADMKFENKVESLIRKVQMECSVPNEYTVLYGTSKGATGALY

HGVNMKLKTLAVDPIVHDEHYIKKFNDLHFVQNVFPESKQEKFSKLFQNNKDENLSHI

KLVTSKNSEQFNYISEIVFSESTTVCSYIFNNPNIKGHTDMGEHTLNFVTAMLNNLLY

DIDVKSNLITVY"

CDS 12801..12974

/locus_tag="appser9_17100"

/codon_start=1

/transl_table=11

/product="hypothetical protein"

/protein_id="EFM93514.1"

/translation="MANKVYKFALATMLINLKETNNDQYDNVIVYHDGFSQTELDDLS

LLEPRVKFIEYTF"

CDS 12937..14019

/gene="cps9F"

/locus_tag="appser9_17090"

/codon_start=1

/transl_table=11

/product="Lipopolysaccharide biosynthesis protein,

LPS:glycosyltransferase"

/protein_id="EFM93513.1"

/translation="MNLGLSLSNIHFEKWNQEHSVLLGDKANNFLNRFSHLAWSKYKI

IEQLEHYEKVLYLDLDMLVSGSLEGLFKLDGIAWRNGANFGEKFSTNGLKVKNIAEVS

NIPESTPAPNGGLLCVTNTIDWKKNLIEAKYFIERFINDFSFALDELAFSYIYVKNNL

KLTQLSAYEYNVLPQHLNPSCKVIHFIGQFKPWKDLIIQAEFPKWFEYYQKACLLTDN

RISSEQVQEYPKDGKYLTKRLNEQRWFSFLREANLNIPQSLRLRYVFDNEWLIFECNS

KVYYEFKFHQYAQGFLMGMWVKDLYKEPEIKREIDNLVGKNPRLFRLHEDHRGLYIYS

TKFSALEIAPVFKDFYERTSKLFNIK"

CDS 14147..14701

/locus_tag="appser9_17080"

/note="similar to Putative uncharacterized protein of

Mannheimia haemolytica UniRef RepID=A7JQX8_PASHA"

/codon_start=1

/transl_table=11

/product="hypothetical protein"

/protein_id="EFM93512.1"

/translation="MFKKITLFSFIALIAGCSSSSQPEAFPGEFANADYVLSDKDAQR

WVVASRQAEQCIYPNLTRIQQQAFSKEDSYIHSQYVFFYPLEEIIGEQYVKIIQDDEK

SMGYAQYLFKKFRDNQEFEPLADKQCLVLREKAKNDLAVVKGQYKSGMVEETTSEAKN

ADGVATNQNKFFFDIIKWGSMLLL"

CDS complement(14773..15315)

/gene="ydeN"

/locus_tag="appser9_17070"

/note="similar to Predicted esterase of the alpha/beta

hydrolase fold COG3545;

similar to Putative uncharacterized protein of

Actinobacillus minor UniRef RepID=B9CWM4_9PAST"

/codon_start=1

/transl_table=11

/product="hypothetical protein"

/protein_id="EFM93511.1"

/translation="MKKVYVTHGYTANPTRNWFPWLKNELEKLGWECECLAMPNSDQP

NPQAWLEHHQNTLQLDENTLLIGHSLGCIALLNYLAVTQQKVKTAIFVSGFYEKLPTL

PELDSFADFYANQTACLPQKSYVISALNDVVVPHSFSDRLAQYLQADYIRLATGGHFI

DREGVTELPELLELIKQISN"

ORIGIN

1 atgccaaata tcaacatcca gaacgcctta ttttcccttg ctcaacacaa taaactctcg

61 attgaatcac tggaaatcaa tactcacgat ttctgggtga ttgtcggcgg taacggctcg

121 ggcaaaaccg ctttcgccca agcgctacat aattcacttt cgttatattc gggtgaatat

181 caaaatagtt tccagcatat cgctttactt tccttcgagc agcaacaaaa aatcatcgag

241 caaatcttta aacaccgtaa caacgatatg gtttcaccgg atgatttcgg tttaaccgcc

301 cgtcaaatta tcttaaacgg tagcgaaaga acgcaattat gcgaggaata tgcggctaaa

361 ttacgtatcc agccgttatt agatcgcccg tttattcagc tctccaccgg cgaaagccgc

421 aaagtgttat tttgccaaat gttagtcagc gaaccggatt tattgatttt agatgagcct

481 tttgaagggt tagaccaagc ctcggtcact tattggcagg aagtgatggc acaactcggt

541 aagcaaatgg cggtggtact gatttccaac cgttttaatg atattcccga ctgtgccaca

601 catattgctt tactggataa cttacaactg attttacaag gcgaacgtca agagattgaa

661 caacaagcgg tctattctca gctaaaattt gcagaacaga atgtgaatgc accgttgccg

721 gagagtgcca caccgctgat tcaactccca ccgaatacta atccgtttga actgaaaaac

781 gtaatgatcc gttacggcga aaaaacgatt attgatgatc taacttggac ggttgcccca

841 aaacaacatt ggtggattaa aggcccgaac ggagcaggaa aatcgacctt actttctatt

901 attgccggcg atcatccgca atcttacgct aattatgtgc atttattcgg tcgtcagcgt

961 ggttcgggcg aaacgatttg ggatataaag aaaaatatcg gctatgtgag cagccaatta

1021 catatggatt atcgggtgaa ttgctctgcg ttagacgtga ttttatccgg cttttttgat

1081 tcaatcggcg tttatcaaca agtaccgagt gccttacagc taaaagcaat ggaatggctg

1141 gaacgcttgc atttagccaa tctggcgaaa aaaccgttcc gttcactttc gtgggggcaa

1201 caacggttat tattgattac tcgtgctatg gtaaaacacc cgccgattct gattttagac

1261 gaaccgctgc aaggtttgga cggtgtaaac cgcaaattgg ttaaacaatt tatcgaacag

1321 cttgtgacta atagtcaaac ccagttgcta tttgtttcgc accaagatgc ggacgcccca

1381 aattgtatca cccatttatt tgaatttgtt ccgcaaacta acggtggtta ccgttatgtg

1441 cagacggcgt taaattaggt ttttgacctt taaaggaaat ccccctcttt agtaaagagg

1501 ggggatgtgt gggactttaa agcgttgaat tatagaactt ataagcctcg tccatattct

1561 caaatttata cataatccct ttatctaata ccattgcatt atcgcaataa gacttcattg

1621 ctgacggact atgcgaaacc aaaataatcg aacgatcttt gcgcttttca aataattcat

1681 gtttacattt tgccgcaaag cgagagtcac ctaccgcaat tacctcatca attaagtagc

1741 aatcaaactc taccgaaagc gacaaagcaa aggcaagtcg ggctttcatg ccggaggaat

1801 atttcttaac cggctcatat aaataatcac ccaattcgga aaattcttcg gtaaaggctt

1861 taacgtattc aatatccgca ttatatatac ggcaaataaa gcgtaaatta tccataccgg

1921 ttaaactgcc ttggaacgcc ccgctgaaag cgagcggcca agatatcgac atattacgtt

1981 cgatagtacc tgatgttggc ggctcaacac cacttaacaa acggattagc gttgatttcc

2041 ctgcaccgtt acgccctaaa ataccgattt tctcgccttt tttcagctca aaattaatat

2101 cttgcaatac ggttttttta ccgcttcgag tatagtaatc tttactcaca ttttttacgc

2161 taatcattgc ggttcgattc ctttactgaa gtttttttacc ataatgagcc caaaaagtaa

2221 catggctaca tcacatatta cgagatagct tatactttca tatgtgataa cactgtcgcc

2281 aaaataaccg tgacgaaaca tttccgtgcc gtgaatcatc ggtattaagg ttgcatattg

2341 ttgagcttgg cttggtagcg catgcacaaa gaaaaatgcg cctgaaagag gtaaaagaac

2401 aaagcttaat gttccccaga ttttgccaaa tgcttcaaat ttttgtgcaa tagaacaaat

2461 aatcaagcct aatcctaatg caaaaaatgc cattaatacc cacgccataa ccatataaaa

2521 cgtatctttc ggcatttcta tccagcctaa taaaatgact aatgccataa taatgatttg

2581 ggcaatcgtt gcacccgcta cctcaagtat gacacgagcc agtaaggtat ctaatacgcg

2641 aacattacga tgataaagaa gactcaagtt accggaaatt gcaccgatag tgcggtttga

2701 tgcattacgc cacatcattg ccattggata accggtaatc acaaaagcaa taatatttaa

2761 atcggaaacg cgatccgctc ggataaattt ccacatcaaa acgataaata aagtgagtaa

2821 tagcggctca acaaacagcc ataaaaaacc caaatttttt cgtccgtaac gcgtaataat

2881 ttcccgcatg agtaatgcac cgattactct cccttgaatg gcgagagatt ggcggaaagt

2941 tgtttgatca ccgtattgca ttagtttttg tgctctctta cgcttgcaat taataaactt

3001 aatacaccat aaagcatcag accgataaag aatgtcgcta aaatattata taagcgataa

3061 ggctcttccg cccagtccgg tttgcttggc tgactgatta cttctaaata aagttgctgg

3121 cgatccgctt catttttcgt attttgtaat gaggttaatg ctgcggtcaa ttgttgctgt

3181 gccagctcgt ttgcaagtac taagcgttgg taatcggcag tttgaatagc aatagagcta

3241 ttactgttac tggaaagctg ttttgattgc tcatcgattt ccttacgtaa actttttgg

3301 cgcataagca atgcatcaac ttgcgggttg tccggtgtaa tagattgcaa ttgagccaat

3361 tgtgtttcta cacgaatcaa ttcgcttttt aagctggaaa ttaatgaaag ttgtacgccg

3421 gattgtgccg gtaaatcaaa gattttattt ttgatacggt atttacttaa gtcgcttgcc

3481 gttgcattta cattattttc cgcttcctta accgcttgtt ccgcaaatga aatggtatct

3541 tttcttgcac gttcgtttaa acggttgatg agtgtttcac cttcggcaag taatttttga

3601 ttaatttgtt gtccctcttc tgcattaaaa gcacgaatac gtaagctggc aataccggat

3661 acagaatcga aatcaacact taagcgatct cggaaatatt tgtaaaacgc ttctttacta

3721 ttatttaaac caaatccatt aaagcgagcg ataatatcgc cttgattctc atagtattca

3781 cgtattggta ggtctttcat taactgttct aatgccgtac gagaatgcat atattcttgt

3841 acggtataag tatcatcttg agcacgagaa aatccggaac cttgtaataa ggccccgaca

3901 ccggttaaag cggtctgatt tttaggagat cttacaacga agcttgattc cgaaatataa

3961 atatcggaag caacagaacc gaaataaaag gctgataata ccgtcggaat cgctacagtt

4021 acccaaaata acggattaag cttttttaac caactttttt tctgtttaat cggtttctgt

4081 agtttttctg ctggactggt agcaataggt gtttccatct tttgtcctta tacattcaat

4141 atattaatag gcacgaacgg cattggtcgt actggtaacc ggcgaagtaa tcgagaaaat

4201 cattctcaag aatttttgga attcagacaa cggtgcattt gatacataga caatatcttt

4261 atcttgcatc gggaagcgtt gtaataaaaa tagtgattgc ggctcaagta aattcacacg

4321 ataaaccgtc ggtacatcca tacccatacc atagcctttc gatccccatt gcgcttgctg

4381 atctaaactc aattgagaaa aaggaacatg acggaagacg aatacccctc tcggatcgga

4441 acgggtatcg attaagcctc ccatcttacc gatagcttcg gcaagcgtaa tacctttact

4501 tgagaatttc atttgctggt tgttacccac cgcacctaaa ccggtaaaac tataaggtgt

4561 gtttagcagt gaaacaacat cgccagcacg taacataata ttttgtgccg gatcggaaat

4621 taacgtttcg aatgcgagtg tttttacttc agaaccacgg gttagcttga ccgtcacatc

4681 ttcaatgttt tccgttgttc cgcctactgc agcaaccgca tctaatacac gttcattatt

4741 agcggttaat ggcatacgaa tactattgcc ttgacgaata accgtaacat cagcagagtt

4801 attattcgca attttgacta atgcttgcgg ctgattcgct ttacgctgca atgccccaac

4861 aatttgagac tgaatcgttt caggtgtttt acctgcgaca cgaatatttc ccacaaacgg

4921 cacggttacc gtcccgtttt gattaaccat ttgtgccggt aattgcgtta aatgcccgct

4981 accttgtcct tcggaactaa aagtaccgcc aaacaacact gccggcggcg cttcccaaat

5041 tgaaatttca agtacatcac ccacattgac tgcaccggca tagcccgcgc tggctactgt

5101 gcctaaaaat ccggaaaatt gttggctttg ctgagtttga tacaactgtt gaactaaacc

5161 gttatccagt tccaccacat ttacttccgg taagggttta tccgaacttt gtgaattagc

5221 ctctaagatc gcactatggc tagggcctga agttgggagg cttgagcaag cagaaaggaa

5281 caatgttgtt aaagctaacc ctatgattga attatatttt ttaatttcca tcttgtaaaa

5341 aggctctatt gaaaaagtgt gtaaaatgaa tataaactat acataattat agatacaaca

5401 ctataattat atattataat ctaatatctc gttttttaaa acgataacct aaaatattac

5461 ttttctatag aagaataggc attattttaa taaatataat taggtatgaa tgttgatgaa

5521 aatagcattt atcgcttgga atagttttca ggtgttacat ttcaagccct tattacaagc

5581 tttaccgtgt gcattattaa ttattgaaaa acggagacgt agtgtaccaa tctgtaagga

5641 tattttgcga gatataaaca ataatatcgc ttatatccgc catacggata tatatgcaaa

5701 aattgatggg aattttgatg ttctagttgc tcaaactact ttcgagcaac tttatttgtt

5761 tcaccgcacc aaaattgcat tgcttcaata cggatatgct aaggaaccgt ataactacgg

5821 cacttggaga gcatttgcag atctaaattt ggtttatggg aattatgcct atgaacgtat

5881 ttcctatttc tctccaacta aaataaccgg ttgtccacga tacgatttat ggtatcagcc

5941 tttatttcat caaaaagcga aagaaaatta tgcgagagta ttagatacga gtaagaaaac

6001 gattgtatat gcaccaagtt ggggagaatt atccagcttt aaattatata tagaagaaat

6061 tacgaaatta tctttatttt acaacgtgtt agtaaaatta caccataaca cgcttttatt

6121 agcaaacaag catcagaatt atgaaaaatt gtatccgaat ttacattttt tctatgaagg

6181 tgaagatctt ctctcactta tttcggtagc cgacattgtt atttccgatt ttagcggtgc

6241 gatctttgac gcaatttttt gtaaaaaaac agtagtgtta ctttcgatat cgctagtgaa

6301 tcaaccaaaa ctagataaat ttagtttaga gatagcttat cgttcaaagt taggatatga

6361 ggttttttcg ccggatcaag tagctataac agtggcacga gcacttacag agccgaaatt

6421 agtagatgaa acgctgtatc aacagctttt tatgcataac aaggatgcaa cacagcaagt

6481 aataaatgct ttacaacagc ttgctgaggg taagtataca ttatctcaac agcagttata

6541 tgtgcgacaa acagaaaaat tattaaatat tgaaaaaata aagcagcaaa aaaataaaaa

6601 acagtctttc aataaaataa gacagatttc taaaagatta attaaaaaat aatttttcat

6661 attttctttt tatatttttt tatttgacaa tacggagttt aggaatgaaa aaagtattaa

6721 cctatggaac ctttgattta ttacaccatg ggcatattcg tttattagaa agagcaagat

6781 cattaggcga tcaccttact gttgctattt ctaccgatca atttaactta ggaaaaggca

6841 aagtatgcgc ttatacttac gaagagagag cgcatatttt aaaagcaatc cgttatgtgg

6901 atgaagtaat tcctgaaaca aactgggagc aaaaagttga ggatgtaaaa aatcacgaaa

6961 ttgacgtatt tgtaatgggt gatgactggg aaggcaaatt tgacttctta gcagattact

7021 gcgaagtggt ttatttaccg agaacccctg atatttcaac gactcaggta aaaaaaatgc

7081 ttgcgaaaaa agatctcgca gccggacaaa aacaaattca cgaaaaagag taatttgtta

7141 atgtttcaaa tcctacaaaa gcatttaccg accttgcaaa gagttctgag ggagggttac

7201 tcccagcatt ctttgcttgc ttattggtat gggcttagtc tacttactgc tcttgaacag

7261 gcgaatcatc ctcaagtaag aaaactggct gagaaaatga tcaataaagg tattaatatc

7321 gggcattatt ttttagcaca aagttatttc ttatgtggag aatatgattt agcggaacaa

7381 gcggtcaaaa aaatcaaaaa ttttgtaaaa atacccgaag ttgttttttt atatgcggac

7441 attctcgtta aatgcaaacg taaagaagag gcttggcaat tattagaaca atgcgcttta

7501 ctcaataaaa gaaaaaaagt gtggatacat ctaacaaatt tagtaaatac tgaggcggat

7561 tatcgacact tagaacaaca tattgacaaa gtaagaacaa ctacacctta cttgaagtct

7621 gatttgttaa ttcatcaaag aacaaatgca gcattaaggg ctggtttaac agaaacggca

7681 ttagcactaa cagaacttaa ccctttgcca aagcaagcaa aagtgaagaa aaaaacaacc

7741 gcttataatg ataaattagc ggcaattgcg ctagcggatc taaagaaagt attagatcac

7801 aaaaaaatac ctttctttct gattagcggc acgttgctag gttgtattcg agaaggaaaa

7861 ttattagggc atgataaaga tattgatgta ggtgtttggg acgaatattc ctacgaagag

7921 ctagcaaatt atttagcaac ttcaggttat ttttatgttg tacccacccg aacaaaacat

7981 ttagtaatgc tacggcacgt aaacggcatt gctattgatg tgtttattca ttatcgagaa

8041 cctaatgact attggcacgc cggtgtcaaa ataaaatggc ataactcgcc atttaactta

8101 gtgtataaag attttctcgg acaacaatat ttaatacctg aaaattacga tttataccta

8161 acagaaaatt acggtgattg gcgcacgcca aaaactaaat ttgatagcgc ctttgatacg

8221 ccaaatatgg aagtgatcaa tgaagctgaa atgcaaattt atgttattaa aaagcgagaa

8281 ctttaaaatg aaacataatg taaaattatc ttctctgcca agtttattac aaggcttata

8341 tttatataaa catcaccaat atcaaaaagc acagaaaatt tttagcaaaa ttttagaaaa

8401 gcaacctaaa aatgcctatc taaatttcag atatggaatg tctttatata aagataaaaa

8461 atggaatgag gcaaattatt ttattcaaaa agcagttgag cttgctccag aacaagagtc

8521 gtggaaaaaa caattagcaa caacagaacg ttataaaaat gattcatcaa aaattaaagt

8581 tgctgaaaat aagaagaatt tagaaaaaca accaaatagc ccagaatata tttgggaata

8641 tgctgtatct ttaattgata gtaaacaata ttggttagca caattccaat tagaaaaata

8701 tattgaatta agacctgact ccgaacgtgc tttttaccag ttaggaattg ttgcggaaaa

8761 attagcgaat tatgaacaag cattttcata ttttcaaaaa gcaagccaat ttgacccgct

8821 taatcgtaat tataaatatc gtatggggta caatctcgaa aaacttggac atttagatca

8881 agcgagaatg tgctatgacc ttgttacgag tttgagtaat gcgggtgatg aagttattga

8941 ttttggcatt ggggcattac acgctaaacg agggctatgg gacttagctg tatctgctta

9001 tttagaattc caagttaaaa caggttctca aaacccagaa ttattttacc gcattggtct

9061 tgctaatgag cgtttatatc aatgggctga atcagcaacc gcttttgagc aagcagttac

9121 gttatctgaa gtaattaatg caaattggtg tttcaaatgt ggacaagctt atgaaagagc

9181 tgggaatttt gagaaatctg ctgaatttta ccaagaagca gttaagagat ccgatcatta

9241 taatgattat tggtggtatc gcttagcttt agtattagag aaactcggta aatatgaaca

9301 atctgttagt gctttccaaa actcaagacg acgtaaactt gcttattccg ttaatcccaa

9361 aaatgttatt aagcataaag aagaagagta tctttcatac tacacagaat attatgaaac

9421 cttagaatta gatgagaaat tagtattaat tgaaagtttc tttggtggaa atattagttg

9481 taatccatac gctattctat cttatatgct aggaaataat tatgactata cttatgttgt

9541 tgtaattaaa gatggaactg ttatccctga taacctcaaa tttaatagaa aaattatttt

9601 tattaaacga ggatcagatg cgtatttacg ttatttatgt acagctaaat atcttattaa

9661 taatgttagt ttcccttatt attttattag aaaagagggt cagatttatt taaatacttg

9721 gcacggtacg ccaatgaaga cattaggaaa agatattaag aatccattta tggatcacgc

9781 taatgtgagt cgaaacttct tgcaagcaac acatattatt tctcctaatc gtcatacaac

9841 tgatattatt ttagagcaat atgatgttaa ggatttattt agtggaaaat tagccgaaac

9901 aggttatccg agaattgatt tggcatttaa tctgacaggt aagagacgtg aagaaatcaa

9961 agaaaaattg gggttatcta ataaaaaacc tgttgtattc tacgcgccaa cgtggcgagg

10021 cacatctcaa tcaaaagatt ttgatacaac gaaattacaa agtgatttaa aaaaacttaa

10081 atcagataaa tataatctga tttttagagg gcatcatcta gtagaacaat tattagaaac

10141 tattaattta gatgtaattg ttgctccgaa agatattgat agtaatgaat tactaggttt

10201 ctgtgattta ttgattactg actactcaag tattatttat gatttcttag cattaaataa

10261 accagcaatt agttatattt atgactatga agaatatgat gctgaacgag gtttatattt

10321 aaaacctgag gaaatgtcgg gtacggtttg cacaacgatt acagatgtta aaaatgctat

10381 tctagaaaat attgctttgg gtaaaactaa tgtatctgaa caagatatta ataaatattc

10441 ttatttagat gatggtaaag ctactaagcg tacggttgaa tttatgtttg atcgtgatga

10501 ttcttgtgtt tataagtatg agcgtaagaa atcagatata ttctttgaag gaccatttat

10561 tccaaatgga atttctcgtt cattcttaaa tttgatggca tcaattaaag attcagaaaa

10621 aaatattaca ttattaatta atggtgcaga tatagcacaa gatcaaaaac gtctagcaga

10681 atttgataat ctaccgtcaa atgttacggt tctctctaga gtgggtagaa ctcctatgac

10741 cttagaagaa ttatgggtga gaaataagtt tgaagaaacg tatcagatgt attcggagtc

10801 atttaccgag actttgttaa aagtatataa acgagaagtg cgtagattgt tgggtgattc

10861 actatttgaa aatgctattc attttgaggg ttattcatta ttttgggtat tattgttctc

10921 tcaaattaat gcgaaaaaac atattattta tcaacataat gataaatata aggaatggaa

10981 aggacgtttc ccgtatttag agggtgtatt caatagctac gtatttttg accagattgt

11041 ttctgtgtca gaaaaaacta tggagaataa tatattaaat ctttcaaaat catttaatat

11101 acctaaagaa aaatttactt tctgtaataa tcctatcaac attcagcaaa ttctctctag

11161 tgcagaggaa gatattgaaa tggaaagtga atttacctca ttcaatggtc agaagtttat

11221 caatattggt agaatgtcgc acgagaaaga tcaattaaaa ttaattgagg ctttctgtga

11281 agctaaaaaa gtacacgcta atattagatt atttattcta ggagatggcg tattaaaaca

11341 ggatttaact aataaaatta aagagctttc attagaaaaa gatgtgtatc ttcttggaca

11401 gaagaaaaat ccattccctt atttaaaaca agcggatgta tttattcttt cttcaaacca

11461 cgaaggacaa ccaatggttc tattagaatc tcttacatta ggtactccaa ttattgctac

11521 cgatattgta ggtaatcgca gtattctagg agataattat ggagtgttag tcgaaaatag

11581 taaggatgga ttagttaaag gtattaatat ttatatggag caaggtggtc gtaaagatag

11641 ttttgatccg tatgaatatc aaaatgatgc tatggcgaag ttttattcat tattaacaaa

11701 ttgaaatatt aaggataaat aatgttagat tttttcagaa ttaaatttga tcaaaacact

11761 aaatgtgatg agttaaatat cgatttatca gtaaaagaaa tttatattga taaatcggct

11821 attggtaaag atgaaaaaga aaagttaaat cataatttct tagcagtagg acgtggtaat

11881 attgaggcta agaaacttat cgctaagttg tcaaataaca aatattattt aacagctcat

11941 tctaatggta tatctatttt cactaagttt agtgatggta aatcatttat ttcacatttt

12001 aagaacaaga atgtaaaagt atggaatgat actttttata cattggatga gcctttagaa

12061 aaagatacat caataaatcg tttattagta gttttttcat ctattgcaga tttagcattt

12121 aatgcttcta ttgaaagacg aatgtttttt actaattttc cgagtgtagg aaaatatatt

12181 cctaagaata catatatttt acgcattgca gatattggtg gggtcttagg aagcttttat

12241 ttgaataata atgcggatat gaagtttgaa aataaggttg aaagtttaat tagaaaagtt

12301 caaatggaat gttccgtacc taatgaatat actgttttat acggtacatc taaaggtgca

12361 acaggcgcac tttaccacgg tgttaatatg aaattaaaaa cgttggcagt tgatccgatt

12421 gtccacgatg aacattatat taaaaagttt aatgatcttc attttgtgca aaatgttttc

12481 cctgaatcaa aacaggaaaa attttcaaaa ctatttcaga ataataagga cgagaattta

12541 agtcatatta aattagttac gtctaagaat tctgaacaat ttaactatat tagcgaaatt

12601 gtattttcag aaagtacgac tgtatgttca tatatattta ataacccgaa tattaaagga

12661 catacagata tgggagaaca tacactaaac tttgttactg ctatgttaaa taacctacta

12721 tatgatattg atgttaaatc aaatttaatt actgtttatt aatctgagac ataaaaatga

12781 ataaaacagc aatatgttta atggcaaata aggtatataa atttgcttta gcaacaatgc

12841 ttattaattt aaaagaaact aataacgatc aatatgataa tgttattgtt tatcacgatg

12901 gattttctca aactgaactt gatgatttat ctttacttga acctagggtt aagtttatcg

12961 aatatacatt ttgaaaaatg gaatcaagaa cactcagtgc tattaggtga taaggctaat

13021 aactttctta acaggttttc tcatttagct tggtctaaat ataaaattat tgagcagtta

13081 gaacactatg aaaaagttct atatctagat ttagatatgc ttgtttctgg ttcattagaa

13141 ggactcttca aattagatgg aattgcttgg agaaatggcg caaactttgg agaaaaattt

13201 tcaactaatg gtcttaaagt taagaatatt gctgaagtaa gtaatattcc tgaatctaca

13261 ccagcaccta atggtgggct tttgtgtgta acaaatacta tcgattggaa aaagaatcta

13321 atagaagcaa aatattttat agaaagattt attaatgatt tttcttttgc attagatgaa

13381 ttagcatttt cttatatata tgtaaaaaat aacttaaaat taactcaatt atccgcttat

13441 gaatataatg ttttacctca gcatttaaat ccaagttgta aggttattca ttttattggg

13501 caatttaaac cgtggaagga tcttatcatt caagcagaat ttccaaaatg gtttgaatat

13561 tatcaaaagg cttgtttact tactgataat aggatatctt ctgagcaagt ccaagagtat

13621 cccaaagatg ggaaatattt aactaaaaga ttaaatgaac agcgttggtt tagttttttg

13681 agagaagcga atttaaatat ccctcaatca ttaagacttc gatatgtatt tgataatgaa

13741 tggcttattt ttgaatgtaa ttcaaaagtt tattatgagt ttaaattcca tcaatatgct

13801 caaggatttc ttatgggtat gtgggttaaa gacttatata aagagccaga aattaagcga

13861 gaaattgata acttagtggg taagaatcct agactattta gattacacga agatcatcgt

13921 ggtctatata tttattctac aaaattttca gctttagaaa ttgcaccggt ttttaaagat

13981 ttctatgaaa gaacatctaa gctatttaat ataaaatgag ttaaagaaga tttccattgc

14041 taagtaatta cgataaagag ttcttttctt cattaaatat tgcttaatct agtgatagaa

14101 tatcaattag aatatatcta ttttctatca agagtaaaaa catactatgt tcaaaaaaat

14161 cacattattc agttttattg cgttaatcgc cggttgttct tcttcctcac aaccggaagc

14221 tttcccgggg gagtttgcga atgcggatta tgtgttatcg gataaagatg cccagcgttg

14281 ggtggtggct agccgtcagg cggagcagtg tatttatccg aacttgacgc ggattcagca

14341 acaagcgttt agtaaggaag attcatatat tcattcgcaa tacgtatttt tctatccgtt

14401 ggaagaaatt atcggcgagc agtatgtaaa gattatccaa gacgatgaaa aatctatggg

14461 atatgcgcaa tacttgttta agaaattcag agataatcag gaattcgagc cgttagcgga

14521 taagcaatgt cttgtgttac gagaaaaagc gaagaacgat ttagcggtcg taaaagggca

14581 gtataagagc ggaatggttg aagaaacgac gtccgaagct aaaaatgcgg acggcgtggc

14641 gaccaatcaa aataaattct tctttgatat tatcaaatgg ggttcgatgc tattactgta

14701 atttgcggtt agtgtgatgt taaagagtat aaaaacgagc tgtcggtaaa tcgttatcgg

14761 cagctctttt tattagtttg atatttgttt gattaactcc aataactccg gtaattccgt

14821 tactccctct cgatcgataa aatgcccgcc cgttgccaag cgaatataat ccgcttgtaa

14881 gtattgcgct aatcgatcgc tgaacgaatg gggaacgaca acatcattta atgcagatat

14941 gacgtaagac ttttgcggta aacaagcggt ctgatttgca taaaaatctg caaagctatc

15001 taattccggc aaagttggta atttctcata aaagccggaa acaaaaattg ccgtttttac

15061 tttttgctgc gttaccgcaa gataattcag taacgcaatg cagcccaaac tatgtccgat

15121 gagtaaggta ttttcatcta attgaagtgt attttggtga tgttccagcc atgcttgcgg

15181 attcggctga tcggaattcg gcatcgctaa acattcacat tcccatccta atttttccaa

15241 ttcgttttta agccacggaa accaatttct tgtcgggttc gccgtataac cgtgcgttac

15301 atatactttt ttcat

//

LOCUS **ADOJ01000030.1** 15428 bp DNA linear BCT 20-APR-2021

DEFINITION Actinobacillus pleuropneumoniae serovar 10 str. D13039

capsular polysaccharide gene locus, complete sequence.

ACCESSION ADOJ01000030 REGION: complement(37488..52915)

VERSION ADOJ01000030.1

KEYWORDS .

SOURCE Actinobacillus pleuropneumoniae serovar 10 str. D13039

ORGANISM Actinobacillus pleuropneumoniae serovar 10 str. D13039

Bacteria; Proteobacteria; Gammaproteobacteria; Pasteurellales;

Pasteurellaceae; Actinobacillus.

REFERENCE 1 (bases 1 to 15428)

AUTHORS Xu,Z., Chen,X., Li,L., Li,T., Wang,S., Chen,H. and Zhou,R.

TITLE Comparative genomic characterization of Actinobacillus

pleuropneumoniae

JOURNAL J. Bacteriol. (2010) In press

PUBMED 20802045

REMARK Publication Status: Available-Online prior to print

REFERENCE 2 (bases 1 to 15428)

AUTHORS Xu,Z., Zhou,R. and Chen,H.

TITLE Direct Submission

JOURNAL Submitted (04-MAY-2010) College of Veterinary Medicine, Huazhong

Agricultural University, Shizishan Street 1, Wuhan 430070, China

FEATURES Location/Qualifiers

source 1..15428

/organism="Actinobacillus pleuropneumoniae serovar 10 str.

D13039"

/mol_type="genomic DNA"

/submitter_seqid="contig00005"

/strain="D13039"

/serovar="10"

/note="K locus: KL10"

/db_xref="taxon:754259"

/note="Coverage of the contig is 21.13X"

CDS 1..1458

/gene="modF"

/locus_tag="appser10_16510"

/note="similar to ABC-type molybdenum transport system,
ATPase component/photorepair protein PhrA COG1119;
similar to ABC transporter related protein of Mannheimia
haemolytica serotype A2 str. OVINE UniRef
RepID=UPI0001BCFBC9"

/codon_start=1

/transl_table=11

/product="ABC transporter protein"

/protein_id="EFM95763.1"

/translation="MPNINIHNALFSLAQHNKLSIESLEINTHDFWVIVGGNGSGKTA
FAQALHNSLSLYSGEYQNSFQHIALLSFEQQQKIIEQIFKHRNNDMISPGDFGLTARQ
IILNGSEKTQLCEEYAAKLRIQPLLDRPFIQLSTGESRKVLFCQMLVSEPDLLILDEP
FEGLDQASVAYWQDVMAQIGKQMAVVLISNRFNDIPDCATHIALLDNLQLILQGERQE
IEQQAVYSQLKFAEQNVNAPLPDSAAPLIQLPPNTNPFELKNVMIRYGEKTIIDDLTW
TVAPKQHWWIKGPNGAGKSTLLSIITGDHPQSYANYVHLFGRQRGSGETIWDIKKNIG
YVSSQLHMDYRVNCSALDVILSGFFDSIGVYQQVPSALQLKAMEWLERLHLANLAKKP
FRSLSWGQQRLLLITRAMVKHPPILILDEPLQGLDGVNRKLVKQFIEQLVTNSQTQLL
FVSHQDADAPNCITHLFEFVPQENGGYQYVQTVLN"

CDS complement(1657..2307)

/gene="cpxA"

/locus_tag="appser10_16500"

/note="similar to ABC-type polysaccharide/polyol phosphate
transport system, ATPase component COG1134;
similar to ATP-binding protein bexA of Bacteria UniRef
RepID=BEXA_HAEIN"

/codon_start=1

/transl_table=11

/product="ATP-binding protein bexA"

/protein_id="EFM95762.1"

/translation="MISVKNVSKDYYTRSGKKTVLQDINFELKKGEKIGILGRNGAGK
STLIRLLSGVEPPTSGTIERNMSISWPLAFSGAFQGSLTGMDNLRFICRIYNADIEYV
KAFTEEFSELGDYLYEPVKKYSSGMKARLAFALSLSVEFDCYLIDEVIAVGDSRFAAK
CKYELFEKRKDRSIILVSHSPSAMKEYCDNAMVLNKGIMHKFENMDDAYKFYNSTP"

CDS complement(2304..3101)
/gene="cpxB"
/locus_tag="appser10_16490"
/note="similar to ABC-type polysaccharide/polyol phosphate
export systems, permease component COG1682;
similar to Capsule polysaccharide export inner-membrane
protein bexB of Proteobacteria UniRef RepID=BEXB2_HAEIN"
/codon_start=1
/transl_table=11
/product="Capsule polysaccharide export inner-membrane
protein bexB"
/protein_id="EFM95761.1"
/translation="MQYGDQTTFRQSLAIQGRVIGALLMREIITRYGRQNLGFLWLFI
EPLSMTLFMVAIWKFMRADQVSSLNIIAFTITGYPLMMMWRNASNRAIGAVTANLSLL
YHRNVQVLDTIFSRMLLEIAGATTAQILIIVIFIALDLIDIPKDPFYMLISWFLMIFF
AFGLGLIICSIAQKSEVFGKIWGTVSFIMMPLSGVFIFVHNIPQHLQSIALWFPMIHG
TEMFRHGYFGDSVITYESISYLVICDVAMLLFGLIMVKNFSKGIEPQ"

CDS complement(3101..4258)
/gene="cpxC"
/locus_tag="appser10_16480"
/note="similar to Capsule polysaccharide export protein
COG3524;
similar to Capsule polysaccharide export inner-membrane
protein ctrB of Proteobacteria UniRef RepID=CTRB_NEIMA"
/codon_start=1
/transl_table=11
/product="Capsule polysaccharide export inner-membrane
protein ctrB"
/protein_id="EFM95760.1"
/translation="METTIMATPTEKLQKPVKQKKSWLKKLNPLFWVTVAIPTVLSAF
YFGSVASDIYISESSFVVRSPQNQTALTGVGALLQGSGFSRAQDDTYTVQEYMHSRTA
LEQLMKDLPIREYYENQGDIIARFNGFGLNNSKEAFYKYFRDRLSVDFDSVSGIASLR
IRAFNAEEGQKINEKLLAEGETLINRLNERARKDTISFAEQAVKEAENNVNATASDLS
KYRIKNKIFDLPAQSGVQLSLISSLKSELIRVETQLAQLQSITPDNPQVDALLMRQKS
LRKEIDEQSKQLSSNSNSSIAIQTADYQRLVLANELAQQQLTAALTSLQNTKNEADRQ

QLYLEVISQPSKPDWAEEPYRLYNILATFFIGLMLYGVLSLLIASVREHKN"

CDS complement(4284..5456)

/gene="cpxD"

/locus_tag="appser10_16470"

/note="similar to Periplasmic protein involved in

polysaccharide export, contains SLBB domain of b-grasp

fold COG1596;

similar to Capsule polysaccharide export protein bexD of

Proteobacteria UniRef RepID=BEXD_HAEIN"

/codon_start=1

/transl_table=11

/product="Capsule polysaccharide export protein bexD"

/protein_id="EFM95759.1"

/translation="MLKKFTFPLASTLIIACSSLPTSGPSHSAILESNSQNSDKPLPE

VNVVELDNGLVQQLYQTQQSQQFSGFLGTVGSAGYAGAVNVGDVLEISIWEAPPAVLF

GGTFSSEGQGSGHLTQLPAQMVNQNGTVTVPFVGNIRVAGKTPEAIQSQIIGALQRKA

NHPQALVKIANNNSADVTVIRQGNSIRMPLTANNERVLDAVAAVGGTTENIEDVTVKL

TRGSEVKTLAFETLISDPAQNIMLRAGDVVSLLNTPYSFTGLGAVGNNQQMKFSSKGI

TLAEAIGKMGGLIDTRSDPRGVFVFRHVPFAQLSLEQQAQWQAKGYAIGMDVPTVYRV

NLLEPQSMFLLQRFPMQDKDIVYVSNAPLSEFQKFLRMIFSITSPVTSTTNAIRAY"

CDS 5648..7006

/gene="cps10A"

/locus_tag="appser10_16460"

/note="similar to Mannose-1-phosphate guanylyltransferase

COG0836;

similar to GDP-mannose pyrophosphorylase of

Gammaproteobacteria UniRef RepID=Q9EXY5_ECOLX"

/codon_start=1

/transl_table=11

/product="GDP-mannose pyrophosphorylase"

/protein_id="EFM95758.1"

/translation="MIRSTVVPVILSGGDGTRLWPLSRQKMPKQFIKFPSGESLFQKT

LHRLDNIDCETPFIITNIQHQSTISHQLQQINKEGFIILEPFRKDTCAAITLAAIQAR

KLYSSSKKIKLLILSSDHYIKDDTVFAQLINNISKSEIGLVSIGIKPTSPHTGYGYIQ

RGKELDEYLTKIEKFHEKPSLDKAHEYIQDGNYLWNSGIFLFDLGIFISELERFSPDI

LLACEQALEGSCIENNIIKVDPIAFQKSPSISIDYAVMEKTSYGSIAVLDTQWSDIGS

WDALAALEKADEDNNVVLGKALAYKTTNSLVYSSGRLVTTLGIDNVAVIETPDAVAVI

HKDYFQENKNVVNDLNNKSYSEVIESSKVIYSWGEEEYIEKNNKYCIKKIFIYPNRSI

HIDSGHFTIINGKLEACSPKKLNKFLLKGTFISVNKDDYNLFINRSNEISELIYIKSE

RL"

CDS 7008..8627

/gene="cps10B"

/locus_tag="appser10_16450"

/note="similar to Capsular polysaccharide biosynthesis

protein COG4421;

similar to Acetyltransferase (Isoleucine patch

superfamily) protein of Actinobacillus minor 202 UniRef

RepID=C8KY83_9PAST"

/codon_start=1

/transl_table=11

/product="Acetyltransferase (Isoleucine patch superfamily)

protein"

/protein_id="EFM95757.1"

/translation="MYKLNEFIENISNEHISIIGKPILFKDSNIISDGKNNKIVFGSN

VKVINCNIRLIGDNNLFFIGDFVTIRGNYLLDTGSEIRIGSRTIFNFVVDIEARESRK

IIIGENCLFSRCRIQTSDVHSIFDRDSLKRINFAKDVYIDSNVWVAFDALICKGSHIR

QNSVIGARSLIAGKFPENSIIAGNPAKVIRKNILWDTKLLDTLDLKEKNMNIITFSEL

LRKRQNDGLKLNPIDSLSMEYKSDHHKNLKDPVLGVVKENDCILTKNFFVLTKDNLFI

KDISYEKYNDYAKDITYEEIIEEPCFLVGGDTNYYHNLINWIPRLFLYEALNLNCKIV

VNSSFSKRQLEVISSIFPYIKDKIVKVNKNIKFKCLYIPNFFLNPIHSPYAIRNLRMR

LFTLYREEIVQPLFPTKFIISRSDASTRKIVNEQELFESLKDFGFSLISLDKLTFIEQ

INLFYHAEMVISPHGASLTNLLWCNHRPRVVEIINEHYTKVFWSLGVLCGVKNYDVFC

GKVIIDKSVNIHRNIEVDIDLLLSKIKKYLS"

CDS 8631..10073

/gene="cps10C"

/locus_tag="appser10_16440"

/codon_start=1

/transl_table=11

/product="hypothetical protein"

/protein_id="EFM95756.1"

/translation="MKVLLQCGDHFENQSRVFLLAKELKQLGVEPVILMYKEKQGNLF

LSNSIKVVYLSSYLSKVKSLKQDIDFSTKIYNDLKIIDFINIEVKRRPRIGWPSQVKK

TSQQVYKYIIAINNILGDINPDHIVVWNGFTGYVANILRVLASEKNIDSSFIERGLLK

DSIFIDVKGVNGNSTLTDLSDYPPANLKLAEYVDKLFIQKAISNTSDKSLLPENIKGK

RVIFFPLQVQLDTNIIMYCKYNTMREVFFEIYSHLNNDDVIFVVRPHPEEDVETLSNL

PNWDNLIVSTDLDLNFWLENSDLIVTINSTVGLEALLKGKPVICLGKSIYSSLPCLSK

YNHILDDRGKILCNVSGYLGYLLTHNLIVKDSIYNTNVIKSIFKFNDKLGESNFIIDT

LSKKDIPFCKVFLDFSLSSKLNLTYRKNSEVITLEWIEKILLSYFKDKEISYVRNIDD

ANIVITDKFYSDLKFQDSKIYIDIYGVALN"

CDS 10083..11606

/gene="cps10D"

/locus_tag="appser10_16430"

/codon_start=1

/transl_table=11

/product="hypothetical protein"

/protein_id="EFM95755.1"

/translation="MILLYFNSNYTKQQYLKILSICQKHKVACLGNSEYIGDFSQFYY

NDIKNIELEKISKILVIDNNIQRKILPNNLLVSLFSIRAFEIEWLSVDGTIEDREKNK

PNTLFIFPGSIIPLSMGSHQRAFNFLYNLSMKGVIFDVLIPSNNKLDKVALKSALKSV

ASNVYFYRNKPKKFTKLNTLKRGIEKRVRTLINKDASLSDLFSERAYRKPTESLKRWV

NSLYLAKDYENIIVSYAWLLDSIQYIEHLRDDFNLICDTHDVQFYRNQNILSRKERLF

FNKDLEKQKEVNLLNKCDYVISISDMDKKLLEENINSKVIPIYPGFDYIKVPVKQRPV

GRPIYFGFIGGSMSANVIALRYVIEHWWPVIKKHSPDSHLYIAGSICNDPSIRELCFF

EKNIELLGFVKDIFSFYNKFEVSLNPVLVSGGLNFKSVEAVCAGKHLFTNTLGKDCLS

TDFPCIIIDDPAQIIQHMNQIEFNFSDDKKRRIASQAKALEIFGNKNHQKSLAKLLG"

CDS 11637..12491

/gene="kdsA"

/locus_tag="appser10_16420"

/note="similar to 3-deoxy-D-manno-octulosonic acid (KDO)

8-phosphate synthase COG2877;

similar to 2-dehydro-3-deoxyphosphooctonate aldolase of

Bacteria UniRef RepID=KDSA_MANSM"

/codon_start=1

/transl_table=11

/product="2-dehydro-3-deoxyphosphooctonate aldolase"

/protein_id="EFM95754.1"

/translation="MNNKIVKVGNIEVANDKPFTLFGGMNVLESRDMAMRVCEQYVEV

TNKLGVPYVFKASFDKANRSSIHSYRGPGMEEGLKIFQELKDTFSVSIITDVHEIYQC

KPVAEVVDIIQLPAFLARQTDLVEAMARTGAVINVKKPQFLSPGQMGNIVEKIAECSN

ENVILCDRGTNFGYDNLVVDMLGFNIMKKVSKGCPVIFDVTHSLQCRDPFGAASGGRR

DQVTELARSGMAIGLAGLFLEAHPDPNNAKCDGPSALPLSKLEAFVSQMKAIDDLVKS

FEEIDTSR"

CDS 12537..13295

/gene="kdsB"

/locus_tag="appser10_16410"

/note="similar to CMP-2-keto-3-deoxyoctulosonic acid

synthetase COG1212;

similar to 3-deoxy-manno-octulosonate cytidylyltransferase

of Gammaproteobacteria UniRef RepID=KDSB_HAES1"

/codon_start=1

/transl_table=11

/product="3-deoxy-manno-octulosonate cytidylyltransferase"

/protein_id="EFM95753.1"

/translation="MKFTIIIPARYASIRLPRKPLLDILGKPMIQHVWERAKQAGGHR

VIIATDHSEIAEVVTRFGGEVCLTSDKHSSGTERLAEVVSKMNISDDEIIVNVQGDEP

LIPPCIIKQVAENLDNHQVNMATLAVKLTQRDELFNPNVVKVLSDKNGMALYFSRAAI

PFARDNFPDCSDDFVTQNQYLRHIGIYAYRAGFIKQYVQWQPTALEQLESLEQLRALW

NGEKIHLDIALETPEVGVDTQEDLERVRLILSNK"

CDS 13307..14242

/gene="kpsF"

/locus_tag="appser10_16400"

/note="similar to Predicted sugar phosphate isomerase

involved in capsule formation COG0794;

similar to Probable phosphosugar isomerase HI1678 of

Bacteria UniRef RepID=Y1678_HAEIN; phosphosugar isomerase

HI1678"

/codon_start=1

/transl_table=11

/product="phosphosugar isomerase"

/protein_id="EFM95752.1"

/translation="MNYLASARETLSLYTQAIDSLHNRLSTEFNQAIEMILSCEGRLV

VAGIGKSGLVGQKMVATFASTGTPSFFLHPTEAFHGDLGMLKPIDIVILISNSGETDD

VNKLIPSLKGFGNKIIAMTGNSHSTLAQHADIILNIGVEKEACPNNLAPTTSTLVTMA

LGDALAIALIKARNFQAMDFARFHPGGSLGRKLLCTVKDVMIRSLPIVSPTAIFSECL

NIMNEGRIGVALVMEHDCLLGIITDGDIRRLLADKGANSLLMTADQIMTKNPKTILES

TFLAKAEEEMKSLHVHSLVVMNEENRVVGIFEFSN"

CDS 14260..14814

/locus_tag="appser10_16390"

/note="similar to Putative uncharacterized protein of

Mannheimia haemolytica UniRef RepID=A7JQX8_PASHA"

/codon_start=1

/transl_table=11

/product="hypothetical protein"

/protein_id="EFM95751.1"

/translation="MFKKITLFSFIALIAGCSSSSQLETFPGEFANADYVLSDKDAQR

WVVASRQVEQCIYPNLTRIQQQAFSKEDSYIHSQYVFFYPLEEIIGEQYVKIIQDDEK

SMGYAQYLFKKFRDNQEFEPLADKQCLVLREKAKNDLAVVKGQYKSGMVEETKSEAKN

VDGVATNQNKFFFDIIKWGSMLLL"

CDS complement(14886..15428)

/gene="ydeN"

/locus_tag="appser10_16380"

/note="similar to Predicted esterase of the alpha/beta

hydrolase fold COG3545;

similar to Putative uncharacterized protein of

Actinobacillus minor UniRef RepID=B9CWM4_9PAST"

/codon_start=1

/transl_table=11

/product="hypothetical protein"

/protein_id="EFM95750.1"

/translation="MKKVYVTHGYTANPTRNWFPWLKNELEKLGWECECLVMPNSDQP

NPQAWLEHHQNTLQLDENTLLIGHSLGCIALLNYLAVTQQKVKTAIFVSGFYEKLPTL

PELDSFADFYANQTACLPQKSYVISALNDVVVPHSFSDRLAQYLQADYIRLATGGHFV

DREGVTELPVLLELIKQISN"

ORIGIN

1 atgccaaaca tcaacatcca taacgcctta ttttcccttg ctcaacacaa taaactgtcg

61 attgaatcac tggaaatcaa tactcacgat ttttgggtaa ttgtcggcgg taacggctcg

121 ggtaaaactg ctttcgccca agcgctacat aattcacttt cactatattc gggcgaatat

181 caaaacagct tccaacatat cgctttactt tccttcgagc agcaacaaaa aatcatcgag

241 caaatcttta aacaccgtaa caacgatatg atttcaccgg gtgatttcgg tttaaccgcc

301 cgtcaaatta tcttaaacgg tagcgaaaaa acgcaattat gcgaagaata tgcagctaaa

361 ttacgtattc agccgttatt agatcgcccg tttattcagc tatccaccgg cgaaagtcgc

421 aaagtgctat tttgccaaat gttagtcagc gaaccggatt tgctgatttt agatgagcct

481 tttgaggggt tagaccaagc ctcggtcgct tattggcaag acgtgatggc acaaatcggc

541 aagcaaatgg cggtggtact gatttccaac cgttttaatg atattcccga ctgtgccaca

601 catattgctt tactggataa cttacaactg attttacaag gcgaacgcca agagattgaa

661 caacaagcgg tctattctca gctaaaattt gcagaacaga atgtgaatgc accgttgccg

721 gacagtgccg caccgctgat tcaactccca ccgaatacta atccgtttga actgaaaaac

781 gtgatgattc gttacggcga aaaaaccatt atcgatgatc taacttggac ggttgcccca

841 aaacaacatt ggtggattaa aggtccgaac ggagcaggaa aatcgacctt actttctatt

901 attaccggcg atcatccgca atcttacgct aattatgtgc atttattcgg tcgtcagcgt

961 ggctcgggag aaacaatttg ggatataaag aaaaatatcg gctatgtgag cagccaatta

1021 catatggatt atcgggtgaa ttgctctgcg ttagacgtaa ttttatccgg ttttttgat

1081 tcaatcggcg tttatcaaca agtaccgagt gcgttacagc tcaaagcaat ggaatggcta

1141 gaacgcttgc atttagccaa tctggcgaaa aaaccgttcc gttcactttc gtgggggcaa

1201 caacgtttat tattgattac tcgtgccatg gtaaaacatc cgccgatttt gattttagac

1261 gaaccgctgc aaggtttgga cggtgtaaac cgcaaattgg ttaaacagtt tatcgaacag

1321 ttggtcacca atagccaaac tcagctgtta tttgtttcgc accaagatgc ggacgcccca

1381 aattgtatca cccatttatt tgaatttgtt ccgcaagaga atggtggtta tcaatacgta

1441 cagacggtgt taaattagat tgttaacctt taaaggaaat ccccctcttt agtaaagagg

1501 gattagggga gatttgtcaa tagagagata tgaaattgaa tagaacttca tttttatat

1561 ttataaaagc gttaattagc atatttcttc gctaattcat tctgtcaaat ctcctcctgc

1621 ccctctttgc taaagagggg agatatgtgc gggactttaa ggcgttgaat tatagaactt

1681 ataagcatcg tccatatttt caaatttatg cataatgcct ttattaagca ccatcgcatt

1741 atcacaatat tccttcattg ctgacggact gtgcgacact aaaatgatcg aacgatcttt

1801 gcgtttctcg aataattcgt acttacattt tgccgcaaag cgagagtcac ctaccgcaat

1861 tacctcatca attaagtagc aatcaaactc aactgaaagc gataaagcaa acgcaagtcg

1921 ggctttcata ccggaggaat atttcttaac cggctcatat aaataatcac ccaattcgga

1981 aaattcctcg gtaaaggctt taacatattc aatatccgca ttatagatac ggcaaataaa

2041 acgtaaatta tccataccgg ttaaactgcc ttgaaacgcc ccgctgaaag cgagcggcca

2101 agatatcgac atattacgtt cgatagtacc cgatgttggc ggctcaacac cacttaacaa

2161 acggattagc gttgattttc ctgcaccgtt acgccctaaa ataccgattt tctcgccttt

2221 tttcagctca aaattaatat cttgcaatac ggttttttta ccgcttcgag tatagtaatc

2281 tttactcaca ttttttacgc taatcattgc ggttcgattc ctttactgaa gtttttacc

2341 ataatgagcc caaaaagtaa catagctaca tcacatatga cgagataact tatactttca

2401 tatgtgataa cactgtcgcc aaaataaccg tgacgaaaca tttccgttcc gtgaatcatt

2461 ggaaaccata gtgctatgga ttgtaggtgc tgaggaatat tatggacaaa aataaagaca

2521 cctgagagag gcatcattat aaaactaact gttccccaaa tttttccaaa tacctcagat

2581 ttttgagcaa tagaacaaat aattaatcct aatccaaatg caaagaaaat cataagaaac

2641 catgaaatta acatataaaa cggatcctta ggtatatcaa ttagatctaa tgcaataaat

2701 ataactataa tcagaatttg tgctgtggtt gctccagcaa tttccaataa cattctagaa

2761 aatatagtgt ctagaacttg aacatttcta tgataaagta aacttaaatt ggcagtaaca

2821 gctccaatag ctcgatttga agcattacgc cacatcatca ttaatggata tcctgtaatt

2881 gtaaatgcaa taatatttaa cgaagaaact tggtcagcac gcatgaattt ccatattgca

2941 accataaata atgtcattga tagaggctca ataaatagcc atagaaaccc tagattttgt

3001 cgcccataac gagtgatgat ttcccgcatg agtaatgcac cgattactct cccttgaatg

3061 gcgagagatt ggcggaaagt tgtttgatca ccgtattgca ttagtttttg tgctctctta

3121 cgcttgcaat taataaactt aatacaccat aaagcatcag accgataaag aatgttgcta

3181 aaatattata taagcgataa ggctcttccg cccagtccgg tttgcttggc tgactgatta

3241 cttctaaata aagttgctgg cgatccgctt catttttcgt attttgtaat gaggttaatg

3301 ctgcggtcaa ttgttgctgt gccagctcat ttgcaagtac taagcgttgg taatcggcag

3361 tttgaatagc aatagagcta ttactgttac tggaaagctg ttttgattgc tcatcgattt

3421 ccttacgtaa acttttttgg cgcataagca atgcatcaac ttgtgggttg tccggtgtaa

3481 tagattgcaa ttgagccaat tgtgtttcta cacgaatcaa ttcgcttttt aagctggaaa

3541 ttaatgaaag ttgtacgccg gattgtgccg gtaaatcaaa gattttattt ttgatacggt

3601 atttacttaa gtcgcttgcc gttgcattta cattattttc cgcttcctta accgcttgtt

3661 ccgcaaatga aatggtatct tttcttgctc tttcatttaa acggttgatg agtgtttcac

3721 cttcggcaag taatttttca ttaatttttt gcccctcttc cgcattaaaa gcacgaatac

3781 gtaagctggc aataccggat acagaatcga aatcaacact taagcgatct cggaaatatt

3841 tgtaaaacgc ttctttacta ttatttaaac caaatccatt aaagcgagcg ataatatcgc

3901 cttgattctc atagtattca cgtattggca agtctttcat taactgttct agtgccgtac

3961 gagaatgcat atattcttgt acggtataag tatcatcttg agctcgagaa aatccggaac

4021 cttgtaataa ggcaccgaca ccggttaaag cggtctgatt ttgaggagat cttacaacga

4081 agcttgattc cgaaatataa atatcggaag caacagaacc gaaataaaag gctgataata

4141 ccgtaggaat cgctacagtt acccaaaata acggattaag cttttttaac caactttttt

4201 tctgtttaac cggtttttgt agtttttctg tcggagttgc cataatagtt gtttccatat

4261 tttatcctta taaattcaat atattaatag gcacgaatag cattagtcgt actggtaacc

4321 ggcgaagtaa tcgagaaaat cattctcaag aatttttgga attcagacaa cggcgcattt

4381 gaaacataca caatatcttt atcttgcatt gggaaacgct gtaataaaaa catggattgc

4441 ggctcaagta agttcacacg ataaaccgtt ggtacatcca ttcctatagc gtagccttta

4501 gcttgccatt gtgcttgttg ttccaaactc aattgtgcaa aaggcacgtg acggaatacg

4561 aaaacccctc tcggatccga acgagtatca attaaaccgc ccatcttacc gatagcttcg

4621 gcaagcgtaa ttcctttact tgagaatttc atttgctggt tgttacccac cgcacctaaa

4681 ccggtaaaac tataaggtgt gtttagcagt gaaacaacat cgccggcacg taacataata

4741 ttttgtgccg gatcggaaat taacgtttcg aatgcgagtg tttttacttc agaaccacgg

4801 gttagcttga ccgtcacatc ttcaatgttt tccgttgttc cgcctactgc agcaaccgca

4861 tctaatacac gttcattatt agcggttaat ggcatacgaa tactattgcc ttgacgaata

4921 accgtaacat cagcagagtt attattcgca attttgacta atgcttgcgg atgattcgct

4981 ttgcgctgta gtgctccaat aatttgagac tgaatcgctt ccggtgtttt gcctgcgaca

5041 cgaatgttac ccacgaacgg cacggtaacc gtaccgtttt gattaaccat ttgtgccggt

5101 aattgcgtta aatgcccgct accttgtccc tcagaactaa aagtaccgcc aaacagcacc

5161 gccggcggag cttcccaaat tgatatttca agtacatcac ccacattgac cgcaccggca

5221 tagcccgcgc tgcctactgt gcctaaaaat ccggaaaatt gttggctttg ctgagtttga

5281 tacaactgtt gaactaaacc gttatccagt tccaccacat ttacttccgg taagggttta

5341 tccgaatttt gggaattaga ttctaaaatt gcgctatggc tagggcctga agttggaaga

5401 gaactgcaag caataattag tgtactggct aaaggaaatg taaatttttt taacatattt

5461 gatgtagtag tcatattgat atttcagctt gtatggtttt ttaaaatagt tgtactctac

5521 ttcaaaaaag tagatactag gcagagtttt attgatttct atctatgttt agttaagttt

5581 acctgtttaa aaatattgat agtatagcat aaattctaat aaataatatt ttaaggacgt

5641 taatataatg attcgtagta cagtggttcc tgtgattttg tctggtggtg atggaacaag

5701 gttatggcct ctttctaggc aaaaaatgcc aaagcagttt ataaaatttc catcaggaga

5761 gtcgctattc caaaaaacat tacatagatt agataatatt gattgtgaaa cgccttttat

5821 tatcacaaat attcagcacc aatctactat ttcgcatcaa ttacagcaaa ttaataaaga

5881 gggttttatt attttagagc ctttccgtaa agatacctgt gctgcaataa cattagcggc

5941 aattcaagca agaaaattat atagttcatc taaaaaaata aaattattaa ttttatcatc

6001 ggatcattat attaaagatg atactgtatt tgctcagtta attaataata ttagtaagtc

6061 tgaaattgga ttagtttcta taggtattaa acctacctct cctcatacgg gatatggtta

6121 tattcaaaga ggaaaagaat tggatgaata tttaactaaa attgaaaaat ttcatgagaa

6181 gcctagccta gataaggcac atgaatatat tcaagatggt aattacttat ggaatagtgg

6241 aatattttta tttgatttag gtatatttat atctgaatta gaaagatttt ctcctgatat

6301 tttattagct tgtgagcaag cattagaagg ttcttgtatt gaaaataata ttattaaagt

6361 tgatccgata gcgtttcaaa agagtcctag tatttctatt gattatgctg taatggaaaa

6421 gacttcttac ggttctatag ctgtattaga tactcaatgg tctgatattg gttcttggga

6481 tgccctcgct gcattagaaa aagcagatga agataataat gttgtactag gtaaagctct

6541 agcatataaa acaactaatt cattagttta ttcatccggc agattagtta ctacattggg

6601 tattgataat gttgctgtta tcgaaacgcc tgatgctgta gcagttatac ataaagacta

6661 ctttcaagag aataaaaatg tagtaaatga cttgaataat aaaagttata gtgaagtaat

6721 tgaaagttca aaggttatat attcttgggg agaagaggaa tatatagaaa agaataataa

6781 atattgtatt aaaaagatat ttatttatcc aaatcgctct atacatattg attcaggtca

6841 tttcaccatt ataaatggta aacttgaagc ttgttcacct aaaaaactta ataagttcct

6901 actaaaagga acatttattt cagtaaataa ggatgattat aatttattta taaatagaag

6961 taatgaaatt tcagagttaa tttatattaa gtcggaaaga ttatagtatg tataaactta

7021 atgaatttat agaaaatata agtaatgagc atatatccat aataggaaaa cctatcttat

7081 ttaaggatag taatatcatt tcggatggga aaaataataa gatagtattt ggttctaatg

7141 taaaagttat taactgtaat attagattaa ttggagataa taaccttttc tttatagggg

7201 attttgttac tataagagga aattatttat tagatacagg atcggaaatt cgtatcggat

7261 ctagaacgat tttcaatttt gttgttgata ttgaggctag agaatcaaga aaaataataa

7321 taggagaaaa ttgcctattt tctcgttgta ggattcagac tagtgatgtt cattctatat

7381 ttgataggga tagtttaaaa agaataaact ttgctaagga tgtttatata gatagtaatg

7441 tttgggtggc ttttgatgct ttaatttgta aaggtagtca tattagacag aattctgtta

7501 ttggtgctag gagcttgatt gcgggtaaat ttcctgaaaa tagtattatt gcaggtaatc

7561 cagctaaagt tattagaaag aatattcttt gggatactaa attattagat acattagatt

7621 taaaagagaa gaatatgaat attattactt ttagtgaatt actccgaaaa agacaaaatg

7681 atggtttaaa gttaaatcct attgattcat taagtatgga gtataaatcg gatcatcata

7741 aaaatttgaa agatccagta ttaggcgtag taaaagaaaa tgattgtatt ttaactaaaa

7801 atttctttgt tcttactaaa gataatttat ttataaaaga tattagttat gaaaaataca

7861 atgattatgc taaggatata acttatgagg aaataattga ggaaccttgc tttttagttg

7921 gtggagatac taattattat cataatctta ttaattggat tccaaggtta tttttatatg

7981 aggcattaaa tctaaattgt aaaatagtag ttaattcatc attctcaaaa agacaattgg

8041 aagttattag ctctattttt ccttatatta aagataaaat tgttaaagta aataaaaaca

8101 ttaaatttaa atgtttatat attccaaatt ttttcttaaa tccaatacat agtccatatg

8161 caatacggaa tcttagaatg agattattta ctttgtatag agaagaaata gttcagccac

8221 tttttccaac taaatttata attagtagaa gtgatgcttc tacaagaaaa attgtaaatg

8281 aacaagaatt atttgagagc ttaaaggatt ttggattttc tttaatatca ttggataagt
8341 taacttttat agaacaaatc aatttgtttt atcatgcaga aatggttatt tctccacatg
8401 gagcatctct tacaaacctc ttatggtgta atcatcgtcc aagagtagtt gagattataa
8461 atgaacatta taccaaggtt ttttggagtt taggtgtttt atgtggtgtt aagaactatg
8521 atgtattttg tggaaaagtc attatagata agtcagtgaa tattcataga aatatagaag
8581 ttgatataga cttattactt agtaaaatta agaaatattt gagttaagat atgaaagtat
8641 tattacaatg tggagatcac tttgaaaatc aatctagagt atttttactt gctaaagagt
8701 taaaacaatt aggggttgaa cctgttattt taatgtataa ggagaagcaa ggaaatttat
8761 tcctatctaa tagtattaaa gttgtttatt tatcttctta tttatctaaa gttaagagtc
8821 taaaacaaga tatagatttt agcactaaaa tctataatga tttaaaaatt attgatttta
8881 ttaacattga ggttaaacgt agacctagaa taggctggcc aagtcaagtt aagaaaacta
8941 gtcaacaagt ctataagtat attatagcta ttaataatat tttaggagat attaatccag
9001 atcatattgt tgtttggaat ggctttacag gctatgttgc taatatttta agagtcttag
9061 cgtcagaaaa aaatatagat tcttctttta tagagagagg attactaaaa gattctattt
9121 ttattgatgt taaaggcgtt aatggaaact caacattgac tgatttaagt gattatcctc
9181 cggccaattt aaaattggca gagtatgtag ataaattatt tattcagaaa gcaataagta
9241 atacttctga taagtccttg ttgccagaga atataaaagg aaaaagagta atatttttcc
9301 cactacaagt tcaattagat accaatatta ttatgtattg taaatacaat acaatgaggg
9361 aggtattttt tgagatatat tcccatttaa ataatgatga tgtcatattt gtcgttagac
9421 ctcatccgga agaagatgtt gaaacattat ccaatttacc taattgggat aatttaatcg
9481 tatcaactga tttagattta aatttttggt tagaaaatag tgatcttata gttactatta
9541 atagtactgt tggattagag gctttattga aagggaagcc tgtaatttgt ttaggaaaat
9601 caatttattc ttcgttgcca tgtttatcta aatacaatca tattttagat gacagaggaa
9661 aaatcttatg taatgtgagt gggtatctag gatatctttt aacacataat ttaattgtaa
9721 aagatagtat atataacact aatgttatta aatctatttt taaatttaat gataagttgg
9781 gagagtctaa ttttatcata gatacattaa gtaaaaaaga catacctttc tgtaaagtgt
9841 ttttagactt ttctttatct agtaagttga atttgacata tagaaaaaac tcagaggtaa
9901 ttacattaga atggatcgag aaaattctct tatcttattt taaagataaa gagatatctt
9961 atgttaggaa tatagatgat gctaatatag taattacaga taagttttac tcagatttaa
10021 aatttcaaga tagtaagatt tatatagata tatatggcgt agcattaaat tagggtttta
10081 taatgatttt attatatttc aattcaaatt atactaaaca acaatattta aaaatattaa
10141 gcatatgtca gaaacataaa gtagcttgtt taggtaactc agaatatatt ggtgatttta
10201 gtcaatttta ttataatgat ataaagaata ttgaactaga aaaaattagt aagatattag
10261 ttatagataa taatattcaa agaaaaatat tgcctaataa ccttttggtt tcattatttt
10321 ctattagagc atttgaaata gaatggctat ctgtagatgg aactattgaa gatagagaaa
10381 aaaataaacc caatactctt tttattttcc caggaagtat tatacctcta tcaatgggtt
10441 ctcatcaaag ggcttttaat tttctttata acttgtccat gaaaggagtt atatttgatg
10501 tacttattcc atctaataat aaattagata aggtagcatt aaaaagtgca ttaaaatctg
10561 tcgctagtaa tgtttatttt tatagaaata aaccaaaaaa atttacaaag ttaaatactc

10621 tgaaaagagg aattgaaaag agagttagaa ctttaattaa taaagatgct tcattatctg

10681 atttattttc tgaaagggca tatagaaagc cgacagaatc tttgaagaga tgggtaaata

10741 gtttatattt agctaaagat tatgagaata ttatagtatc ttatgcgtgg ctattggatt

10801 ctattcaata tatagaacat ttacgagatg attttaattt aatttgtgat acgcacgatg

10861 tacagttcta tagaaatcaa aatatattaa gtcgaaaaga gcgattgttt tttaacaaag

10921 atttagaaaa gcaaaaagaa gtaaacttat taaataagtg cgattatgta attagtattt

10981 cagatatgga taagaaatta ttagaggaaa atattaattc caaagtaatt ccgatatatc

11041 caggatttga ttatataaaa gtccctgtaa aacaacgtcc ggttggacga cctatttatt

11101 ttggctttat tggtggctct atgtctgcta atgtaattgc attaagatat gtgatagagc

11161 attggtggcc tgtaattaag aagcattctc cagatagtca tttatatatt gcaggatcaa

11221 tatgcaatga tccttcaatc agagagctat gttttttga aaaaaatata gaattattag

11281 gatttgtgaa ggacattttt agcttttata ataagttcga agtaagttta aaccccgttc

11341 tagtatcggg aggattaaat ttcaaaagtg ttgaggctgt ttgtgctggc aaacacttat

11401 tcactaatac attaggaaaa gattgtctaa gtactgattt tccttgtatc atcattgatg

11461 atccggcaca aattattcaa catatgaacc aaattgaatt taatttttct gacgataaga

11521 aaagaagaat agcttcgcaa gcgaaagctt tagaaatttt tggtaataaa aatcaccaaa

11581 aaagtttagc taaattgctt gggtgatgat tatttaacta tattaggata atataaatga

11641 acaacaaaat cgtaaaagtt ggcaatattg aagtggcgaa tgacaagccg tttactttat

11701 tcggtggtat gaacgtgtta gaaagccgtg atatggcaat gcgtgtctgt gaacaatacg

11761 tagaggtgac gaacaaactg ggtgtgcctt atgtatttaa ggcttctttc gataaagcaa

11821 accgctcgtc aattcactct tatcgtggac cgggtatgga agaaggttta aaaatcttcc

11881 aagaattaaa agatacgttc agtgtgagca ttattactga cgtacacgaa atctatcaat

11941 gtaaaccggt agcggaagtg gtggatatta tccagttacc agcgttctta gctcgccaaa

12001 cggatttagt cgaagcaatg gcacgtactg gtgcggtaat caacgtgaaa aaacctcaat

12061 ttttaagccc gggtcaaatg ggtaatatcg tagaaaaaat tgcagaatgc agtaacgaaa

12121 acgtaattct ttgcgaccgt ggtactaact tcggctacga taatttagtg gtggatatgc

12181 tcggctttaa cattatgaaa aaagtatcaa aaggctgtcc ggtcattttt gacgtaactc

12241 actcgttaca atgtcgtgat ccattcggtg cagcatccgg tggtcgccgt gatcaagtaa

12301 ccgaattagc tcgtagcggt atggctattg gtttagccgg tttattcctt gaggctcatc

12361 cggatccaaa caatgcgaaa tgtgacggtc cgtctgcttt accgttatca aaattagaag

12421 cattcgttag ccaaatgaaa gcgattgatg atttagttaa atcatttgaa gaaattgata

12481 catcaagata agaaatactt cccctcttaa ttagagggga tttttatgag gtttttatga

12541 agtttactat tattatcccg gctcgttatg cttcgattcg cttacctaga aaaccgttat

12601 tggatatttt aggtaaaccg atgatccaac acgtttggga aagagcaaag caagcgggag

12661 gacatcgagt aattattgca acagatcatt ctgagattgc tgaggttgtt actcgctttg

12721 gtggagaagt ttgcctaact tcggataaac attcatccgg aacagaacgt ttagctgaag

12781 ttgttagtaa aatgaatatt agtgatgatg agattattgt taatgttcaa ggcgatgagc

12841 ctctcattcc accgtgtatt atcaaacaag ttgcagagaa tttagataat catcaagtaa

12901 atatggcaac attagcggtt aaattaactc aaagagatga attatttaat cctaatgtag

12961 taaaagtact ttcagataaa aatggtatgg cgttatattt ttctcgtgca gctattccat

13021 ttgctagaga taacttccct gattgttcag atgattttgt tactcaaaac cagtatcttc

13081 gtcatatagg tatttatgct tatcgtgcag gcttcattaa gcagtatgta caatggcaac

13141 cgacggcttt agaacagcta gaatctttag agcagttaag ggctttgtgg aatggagaaa

13201 agatacactt agatattgca ttagaaacgc ctgaagttgg tgttgatacg caagaagatt

13261 tagagcgagt tcgtttaatt ttatcaaata aataaggcta taaaatatga attacttagc

13321 aagtgctaga gaaacattat ctttatatac tcaagcaata gatagtttac ataatcgctt

13381 atctactgag tttaatcaag ctattgaaat gattttatct tgtgagggac gtttagttgt

13441 tgctggtatt ggtaaatcag gtttagttgg tcagaaaatg gttgctactt ttgcttctac

13501 gggaacacca agtttctttt tacatccaac cgaggcattt cacggtgatt tggggatgtt

13561 aaaacccatc gatatcgtca ttcttatttc aaatagtggt gaaactgatg acgtaaataa

13621 attaattcct agcctgaaag gttttggtaa taaaattatt gcaatgacag gtaattctca

13681 ttcaacccta gcacagcatg ctgatattat tttaaatatt ggcgtagaaa aagaagcctg

13741 tccaaataat ctggctccaa caacgtccac attagtaact atggcactag gagatgcact

13801 tgcgattgct ttaattaaag caagaaactt ccaagctatg gactttgctc gttttcatcc

13861 gggtggcagt ttagggcgaa aattactctg tacggttaaa gatgtaatga ttagatcatt

13921 accaatagtt tctccaacgg caatatttag cgaatgttta aatataatga atgaaggtcg

13981 aataggcgtt gctctagtta tggaacatga ctgtttactg ggaattatta ctgatgggga

14041 tattcgtcgt ttattagctg ataaaggagc taatagtttg ttaatgacag ctgatcaaat

14101 tatgacgaag aatcctaaaa ctatcctaga aagtactttt cttgctaaag cggaagaaga

14161 gatgaaatca ttgcatgtac attcattagt tgtaatgaat gaagaaaaca gagttgtagg

14221 tatatttgaa ttttcaaatt agaattatta taggatatta tgttcaaaaa aatcacttta

14281 ttcagtttta tcgcgttaat cgccggttgc tcttcttcct cacaactgga aactttccct

14341 ggggagtttg cgaatgcgga ttatgtgtta tcggacaagg atgcccagcg ttgggtggtg

14401 gcgagccgtc aggtggagca gtgtatttat ccgaacttga cgcggattca gcagcaagcg

14461 tttagcaagg aagattcata tattcattcg caatacgtat ttttctatcc gttggaagaa

14521 attatcggcg agcagtatgt aaaaattatc caagacgatg aaaaatctat gggatatgcg

14581 caatacttgt ttaagaaatt cagagataat caggaattcg agccgttagc ggataagcaa

14641 tgtcttgtgt tgcgagaaaa agcgaagaac gatttagcgg tcgtaaaagg gcagtataag

14701 agcggaatgg ttgaagaaac gaagtccgaa gctaaaaatg tggacggcgt ggcgaccaat

14761 caaaataaat tcttctttga tattatcaaa tggggttcga tgctattact gtaatttgcg

14821 gttagtgtga cgttaaagag tataaaaacg agctgtcggt aaatcgttat cggcagctct

14881 ttttattagt ttgatatttg tttgattaac tccaataaaa ccggtaattc cgttactccc

14941 tctcgatcga caaaatgccc gcccgttgcc aagcgaatat aatccgcttg taagtattgc

15001 gctaatcgat cgctgaacga atggggaacg acaacatcat ttaatgcaga tatgacgtaa

15061 gacttttgcg gtaaacaagc ggtctgattt gcataaaaat ctgcaaagct atctaattcc

15121 ggcaaagttg gtaatttctc ataaaagccg gaaacaaaaa ttgccgtttt tactttttgc

15181 tgcgttaccg ccagataatt cagtaacgca atgcagccca aactatgtcc gatgagtaag

15241 gtattttcat ctaattgaag tgtattttgg tgatgttcca gccatgcttg cggattcggc

15301 tgatcggaat tcggcatcac taaacattca cattcccatc ctaatttttc caattcgttt

15361 ttaagccacg gaaaccaatt tcttgtcggg ttcgccgtat aaccgtgcgt tacatatact

15421 tttttcat

//

LOCUS ADOK01000031.1 15314 bp DNA linear BCT 20-APR-2021

DEFINITION Actinobacillus pleuropneumoniae serovar 11 str. 56153 ,

capsular polysaccharide gene locus, complete sequence.

ACCESSION ADOK01000031 REGION: complement(37672..52985)

VERSION ADOK01000031.1

KEYWORDS .

SOURCE Actinobacillus pleuropneumoniae serovar 11 str. 56153

ORGANISM Actinobacillus pleuropneumoniae serovar 11 str. 56153

Bacteria; Proteobacteria; Gammaproteobacteria; Pasteurellales;

Pasteurellaceae; Actinobacillus.

REFERENCE 1 (bases 1 to 15314)

AUTHORS Xu,Z., Chen,X., Li,L., Li,T., Wang,S., Chen,H. and Zhou,R.

TITLE Comparative genomic characterization of Actinobacillus

pleuropneumoniae

JOURNAL J. Bacteriol. (2010) In press

PUBMED 20802045

REMARK Publication Status: Available-Online prior to print

REFERENCE 2 (bases 1 to 15314)

AUTHORS Xu,Z., Zhou,R. and Chen,H.

TITLE Direct Submission

JOURNAL Submitted (04-MAY-2010) College of Veterinary Medicine, Huazhong

Agricultural University, Shizishan Street 1, Wuhan 430070, China

FEATURES Location/Qualifiers

source 1..15314

/organism="Actinobacillus pleuropneumoniae serovar 11 str.

56153"

/mol_type="genomic DNA"

/submitter_seqid="contig00027"

/strain="56153"

/serovar="11"

/note="K locus: KL11"

/db_xref="taxon:754260"

/note="Coverage of the contig is 27.42X"

CDS 1..1458

/gene="modF"

/locus_tag="appser11_17320"

/note="similar to ABC-type molybdenum transport system,

ATPase component/photorepair protein PhrA COG1119;

similar to ABC transporter related protein of Mannheimia

haemolytica serotype A2 str. OVINE UniRef

RepID=UPI0001BCFBC9"

/codon_start=1

/transl_table=11

/product="ABC transporter protein"

/protein_id="EFM97848.1"

/translation="MPNINIQNALFSLAQHNKLSIESLEINTHDFWVIVGGNGSGKTA

FAQALHNSLSLYSGEYQNSFQHIALLSFEQQQKIIEQIFKHRNNDMVSPDDFGLTARQ

IILNGSERTQLCEEYAAKLRIQPLLDRPFIQLSTGESRKVLFCQMLVSEPDLLILDEP

FEGLDQASVTYWQEVMAQLGKQMAVVLISNRFNDIPDCATHIALLDNLQLILQGERQE

IEQQAVYSQLKFAEQNVNAPLPESATPLIQLPPNTNPFELKNVMIRYGEKTIIDDLTW

TVAPKQHWWIKGPNGAGKSTLLSIIAGDHPQSYANYVHLFGRQRGSGETIWDIKKNIG

YVSSQLHMDYRVNCSALDVILSGFFDSIGVYQQVPSALQLKAMEWLERLHLANLAKKP

FRSLSWGQQRLLLITRAMVKHPPILILDEPLQGLDGVNRKLVKQFIEQLVTNSQTQLL

FVSHQDADAPNCITHLFEFVPQTNGGYRYVQTALN"

CDS complement(1517..2167)

/gene="cpxA"

/locus_tag="appser11_17310"

/note="similar to ABC-type polysaccharide/polyol phosphate

transport system, ATPase component COG1134;

similar to ATP-binding protein bexA of Bacteria UniRef

RepID=BEXA_HAEIN"

/codon_start=1

/transl_table=11

/product="ATP-binding protein bexA"

/protein_id="EFM97847.1"

/translation="MISVKNVSKDYYTRSGKKTVLQDINFELKKGEKIGILGRNGAGK

STLIRLLSGVEPPTSGTIERNMSISWPLAFSGAFQGSLTGMDNLRFICRIYNADIEYV

KAFTEEFSELGDYLYEPVKKYSSGMKARLAFALSLSVEFDCYLIDEVIAVGDSRFAAK

CKHELFEKRKDRSIILVSHSPSAMKSYCDNAMVLDKGIMYKFENMDEAYKFYNSTL"

CDS complement(2164..2961)

/gene="cpxB"

/locus_tag="appser11_17300"

/note="similar to ABC-type polysaccharide/polyol phosphate

export systems, permease component COG1682;
similar to Capsule polysaccharide export inner-membrane
protein bexB of Proteobacteria UniRef RepID=BEXB2_HAEIN"
/codon_start=1
/transl_table=11
/product="Capsule polysaccharide export inner-membrane
protein bexB"
/protein_id="EFM97846.1"
/translation="MQYGDQTTFRQSLAIQGRVIGALLMREIITRYGRKNLGFLWLFV
EPLLLTLFIVLMWKFIRADRVSDLNIIAFVITGYPMAMMWRNASNRTIGAISGNLSLL
YHRNVRVLDTLLARVILEVAGATIAQIIIMALVILLGWIEMPKDTFYMVMAWVLMAFF
ALGLGLIICSIAQKFEAFGKIWGTLSFVLLPLSGAFFFVHALPSQAQQYATLIPMIHG
TEMFRHGYFGDSVITYESISYLVICDVAMLLFGLIMVKNFSKGIEPQ"

CDS complement(2961..4118)
/gene="cpxC"
/locus_tag="appser11_17290"
/note="similar to Capsule polysaccharide export protein
COG3524;
similar to Capsule polysaccharide export inner-membrane
protein ctrB of Proteobacteria UniRef RepID=CTRB_NEIMA"
/codon_start=1
/transl_table=11
/product="Capsule polysaccharide export inner-membrane
protein ctrB"
/protein_id="EFM97845.1"
/translation="METPIATSPAEKLQKPIKQKKSWLKKLNPLFWVTVAIPTVLSAF
YFGSVASDIYISESSFVVRSPKNQTALTGVGALLQGSGFSRAQDDTYTVQEYMHSRTA
LEQLMKDLPIREYYENQGDIIARFNGFGLNNSKEAFYKYFRDRLSVDFDSVSGIASLR
IRAFNAEEGQQINQKLLAEGETLINRLNERARKDTISFAEQAVKEAENNVNATASDLS
KYRIKNKIFDLPAQSGVQLSLISSLKSELIRVETQLAQLQSITPDNPQVDALLMRQKS
LRKEIDEQSKQLSSNSNSSIAIQTADYQRLVLANELAQQQLTAALTSLQNTKNEADRQ
QLYLEVISQPSKPDWAEEPYRLYNILATFFIGLMLYGVLSLLIASVREHKN"

CDS complement(4144..5331)
/gene="cpxD"
/locus_tag="appser11_17280"
/note="similar to Periplasmic protein involved in
polysaccharide export, contains SLBB domain of b-grasp
fold COG1596;
similar to Capsule polysaccharide export protein bexD of

Proteobacteria UniRef RepID=BEXD_HAEIN"

/codon_start=1

/transl_table=11

/product="Capsule polysaccharide export protein bexD"

/protein_id="EFM97844.1"

/translation="MEIKKYNSIIGLALTTLFLSACSSLPTSGPSHSAILEANSQSSD

KPLPEVNVVELDNGLVQQLYQTQQSQQFSGFLGTVASAGYAGAVNVGDVLEISIWEAP

PAVLFGGTFSSEGQGSGHLTQLPAQMVNQNGTVTVPFVGNIRVAGKTPETIQSQIVGA

LQRKANQPQALVKIANNNSADVTVIRQGNSIRMPLTANNERVLDAVAAVGGTTENIED

VTVKLTRGSEVKTLAFETLISDPAQNIMLRAGDVVSLLNTPYSFTGLGAVGNNQQMKF

SSKGITLAEAIGKMGGLIDTRSDPRGVFVFRHVPFSQLSLDQQAQWGSKGYGMGMDVP

TVYRVNLLEPQSLFLLQRFPMQDKDIVYVSNAPLSEFQKFLRMIFSITSPVTSTTNAV

RAY"

CDS 5510..6652

/gene="cps11A"

/locus_tag="appser11_17270"

/note="similar to Cps2A of Actinobacillus pleuropneumoniae

UniRef RepID=B0BRQ7_ACTPJ"

/codon_start=1

/transl_table=11

/product="Cps2A"

/protein_id="EFM97843.1"

/translation="MLMKIAFIAWNSFQVLHFKPLLQALPCALLIIEKRRRSVPICKD

ILRDINNNIAYIRHTDIYAKIDGNFDVLVAQTTFEQLYLFHRTKIALLQYGYAKEPYN

YGTWRAFADLNLVYGNYAYERISYFSPTKITGCPRYDLWYQPLFHQKAKENYARVLDT

SKKTIVYAPSWGELSSFKLYIEEITKLSLFYNVLVKLHHNTLLLANKHQNYEKLYPNL

HFFYEGEDLLSLISVADIVISDFSGAIFDAIFCKKTVVLLSISLVNQPKLDKFSLEIA

YRSKLGYEVFSPDQVAITVARALTEPKLVDETLYQQLFMHNKDATQQVINALQQLAEG

KYTLSQQQLYVRQTEKLLNIEKIKQQKNKKQSFNKIRQISKRLIKK"

CDS 6705..7133

/gene="cps11B"

/locus_tag="appser11_17260"

/note="similar to Cytidylyltransferase COG0615;

similar to Glycerol-3-phosphate cytidylyltransferase of

Bacteria UniRef RepID=TARD_BACSU"

/codon_start=1

/transl_table=11

/product="Glycerol-3-phosphate cytidylyltransferase"

/protein_id="EFM97842.1"

/translation="MKKVLTYGTFDLLHHGHIRLLERARSLGDHLTVAISTDQFNLGK
GKVCAYTYEERAHILKAIRYVDEVIPETNWEQKVEDVKNHEIDVFVMGDDWEGKFDFL
ADYCEVVYLPRTPDISTTQVKKMLAKKDLAAGQKQIHEKE"

CDS 7213..8286

/gene="cps11C"

/locus_tag="appser11_17250"

/note="similar to Cps7C of Actinobacillus pleuropneumoniae
UniRef RepID=B0BRQ5_ACTPJ"

/codon_start=1

/transl_table=11

/product="Cps7C"

/protein_id="EFM97841.1"

/translation="MLAYWYGLSLLTALEQANHPQVRKLAEKMINKGINIGHYFLAQS
YFLCGEYDLAEQAVKKIKNFVKIPEVVFLYADILVKCKRKEEAWQLLEQCALLNKRKK
VWIHLTNLVNTEADYRHLEQHIDKVRTTTPYLKSDLLIHQRTNAALRAGLTETALALT
ELNPLPKQAKVKKKTTAYNDKLAAIALADLKKVLDHKKIPFFLISGTLLGCIREGKLL
GHDKDIDVGVWDEYSYEELANYLATSGYFYVVPTRTKHLVMLRHVNGIAIDVFIHYRE
PNDYWHAGVKIKWHNSPFNLVYKDFLGQQYLIPENYDLYLTENYGDWRTPKTKFDSAF
DTPNMEVINEAEMQIYVIKKREL"

CDS 8261..11704

/gene="cps11D"

/locus_tag="appser11_17240"

/note="similar to Putative glycosyl/glycerophosphate
transferases involved in teichoic acid biosynthesis
TagF/TagB/EpsJ/RodC COG1887;
similar to Teichoic acid biosynthesis protein of
Actinobacillus pleuropneumoniae serovar 3 str. JL03 UniRef
RepID=B0BRQ4_ACTPJ"

/codon_start=1

/transl_table=11

/product="Teichoic acid biosynthesis protein"

/protein_id="EFM97840.1"

/translation="MLLKSENFKMKHNVKLSSLPSLLQGLYLYKHHQYQKAQKIFSKI
LEKQPKNAYLNFRYGMSLYKDKKWNEANYFIQKAVELAPEQESWKKQLATTERYKNDS
SKIKVAENKKNLEKQPNSPEYIWEYAVSLIDSKQYWLAQFQLEKYIELRPDSERAFYQ
LGIVAEKLANYEQAFSYFQKASQFDPLNRNYKYRMGYNLEKLGHLDQARMCYDLVTSL
SNAGDEVIDFGIGALHAKRGLWDLAVSAYLEFQVKTGSQNPELFYRIGLANERLYQWA
ESATAFEQAVTLSEVINANWCFKCGQAYERAGNFEKSAEFYQEAVKRSDHYNDYWWYR
LALVLEKLGKYEQSVSAFQNSRRRKLAYSVNPKNVIKHKEEEYLSYYTEYYETLELDE

KLVLIESFFGGNISCNPYAILSYMLGNNYDYTYVVVIKDGTVIPDNLKFNRKIIFIKR
GSDAYLRYLCTAKYLINNVSFPYYFIRKEGQIYLNTWHGTPMKTLGKDIKNPFMDHAN
VSRNFLQATHIISPNRHTTDIILEQYDVKDLFSGKLAETGYPRIDLAFNLTGKRREEI
KEKLGLSNKKPVVFYAPTWRGTSQSKDFDTTKLQSDLKKLKSDKYNLIFRGHHLVEQL
LETINLDVIVAPKDIDSNELLGFCDLLITDYSSIIYDFLALNKPAISYIYDYEEYDAE
RGLYLKPEEMSGTVCTTITDVKNAILENIALGKTNVSEQDINKYSYLDDGKATKRTVE
FMFDRDDSCVYKYERKKSDIFFEGPFIPNGISRSFLNLMASIKDSEKNITLLINGADI
AQDQKRLAEFDNLPSNVTVLSRVGRTPMTLEELWVRNKFEETYQMYSESFTETLLKVY
KREVRRLLGDSLFENAIHFEGYSLFWVLLFSQINAKKHIIYQHNDKYKEWKGRFPYLE
GVFNSYVFFDQIVSVSEKTMENNILNLSKSFNIPKEKFTFCNNPINIQQILSSAEEDI
EMESEFTSFNGQKFINIGRMSHEKDQLKLIEAFCEAKKVHANIRLFILGDGVLKQDLT
NKIKELSLEKDVYLLGQKKNPFPYLKQADVFILSSNHEGQPMVLLESLTLGTPIIATD
IVGNRSILGDNYGVLVENSKDGLVKGINIYMEQGGRKDSFDPYEYQNDAMAKFYSLLT
N"

CDS 11722..12762
/gene="cps11E"
/locus_tag="appser11_17230"
/note="similar to Putative uncharacterized protein of
Actinobacillus pleuropneumoniae serovar 3 str. JL03 UniRef
RepID=B0BRQ2_ACTPJ"
/codon_start=1
/transl_table=11
/product="hypothetical protein"
/protein_id="EFM97839.1"
/translation="MLDFFRIKFDQNTKCDELNIDLSVKEIYIDKSAIGKDEKEKLNH
NFLAVGRGNIEAKKLIAKLSNNKYYLTAHSNGISIFTKFSDGKSFISHFKNKNVKVWN
DTFYTLDEPLEKDTSINRLLVVFSSIADLAFNASIERRMFFTNFPSVGKYIPKNTYIL
RIADIGGVLGSFYLNNNADMKFENKVESLIRKVQMECSVPNEYTVLYGTSKGATGALY
HGVNMKLKTLAVDPIVHDEHYIKKFNDLHFVQNVFPESKQEKFSKLFQNNKDENLSHI
KLVTSKNSEQFNYISEIVFSESTTVCSYIFNNPNIKGHTDMGEHTLNFVTAMLNNLLY
DIDVKSNLITVY"

CDS 12777..14018
/gene="cps11F"
/locus_tag="appser11_17220"
/codon_start=1
/transl_table=11
/product="Lipopolysaccharide biosynthesis protein,
LPS:glycosyltransferase"
/protein_id="EFM97838.1"

/translation="MNKTAICLMANKVYKFALATMLINLKETNNDQYDNVIVYHDGFS
QTELDDLSLLEPRVKFIEYTFEKWNQEHSVLLGDKANNFLNRFSHLAWSKYKIIEQLE
HYEKVLYLDLDMLVSGSLEGLFKLDGIAWRNGANFGEKFSTNGLKVKNIAEVSNIPES
TPAPNGGLLCVTNTIDWKKNLIEAKYFIERFINDFSFALDELAFSYIYVKNNLKLTQL
SAYEYNVLPQHLNPSCKVIHFIGQFKPWKDLIIQAEFPKWFEYYQKACLLTDNRISSE
QVQEYPKDGKYLTKRLNEQRWFSFLREANLNIPQSLRLRYVFDNEWLIFECNSKVYYE
FKFHQYAQGFLMGMWVKDLYKEPEIKREIDNLVGKNPRLFRLHEDHRGLYIYSTKFSA
LEIAPVFKDFYERTSKLFNIK"

CDS 14146..14700

/locus_tag="appser11_17210"

/note="similar to Putative uncharacterized protein of
Mannheimia haemolytica UniRef RepID=A7JQX8_PASHA"

/codon_start=1

/transl_table=11

/product="hypothetical protein"

/protein_id="EFM97837.1"

/translation="MFKKITLFSFIALIAGCSSSSQPEAFPGEFANADYVLSDKDAQR
WVVASRQAEQCIYPNLTRIQQQAFSKEDSYIHSQYVFFYPLEEIIGEQYVKIIQDDEK
SMGYAQYLFKKFRDNQEFEPLADKQCLVLREKAKNDLAVVKGQYKSGMVEETTSEAKN
ADGVATNQNKFFFDIIKWGSMLLL"

CDS complement(14772..15314)

/gene="ydeN"

/locus_tag="appser11_17200"

/note="similar to Predicted esterase of the alpha/beta
hydrolase fold COG3545;
similar to Putative uncharacterized protein of
Actinobacillus minor UniRef RepID=B9CWM4_9PAST"

/codon_start=1

/transl_table=11

/product="hypothetical protein"

/protein_id="EFM97836.1"

/translation="MKKVYVTHGYTANPTRNWFPWLKNELEKLGWECECLAMPNSDQP
NPQAWLEHHQNTLQLDENTLLIGHSLGCIALLNYLAVTQQKVKTAIFVSGFYEKLPTL
PELDSFADFYANQTACLPQKSYVISALNDVVVPHSFSDRLAQYLQADYIRLATGGHFI
DREGVTELPELLELIKQISN"

ORIGIN

1 atgccaaata tcaacatcca gaacgcctta ttttcccttg ctcaacacaa taaactctcg

61 attgaatcac tggaaatcaa tactcacgat ttctgggtga ttgtcggcgg taacggctcg

121 ggcaaaaccg ctttcgccca agcgctacat aattcacttt cgttatattc gggtgaatat

181 caaaatagtt tccagcatat cgctttactt tccttcgagc agcaacaaaa aatcatcgag

241 caaatcttta aacaccgtaa caacgatatg gtttcaccgg atgatttcgg tttaaccgcc

301 cgtcaaatta tcttaaacgg tagcgaaaga acgcaattat gcgaggaata tgcggctaaa

361 ttacgtatcc agccgttatt agatcgcccg tttattcagc tctccaccgg cgaaagccgc

421 aaagtgttat tttgccaaat gttagtcagc gaaccggatt tattgatttt agatgagcct

481 tttgaagggt tagaccaagc ctcggtcact tattggcagg aagtgatggc acaactcggt

541 aagcaaatgg cggtggtact gatttccaac cgttttaatg atattcccga ctgtgccaca

601 catattgctt tactggataa cttacaactg attttacaag gcgaacgtca agagattgaa

661 caacaagcgg tctattctca gctaaaattt gcagaacaga atgtgaatgc accgttgccg

721 gagagtgcca caccgctgat tcaactccca ccgaatacta atccgtttga actgaaaaac

781 gtaatgatcc gttacggcga aaaaacgatt attgatgatc taacttggac ggttgcccca

841 aaacaacatt ggtggattaa aggcccgaac ggagcaggaa aatcgacctt actttctatt

901 attgccggcg atcatccgca atcttacgct aattatgtgc atttattcgg tcgtcagcgt

961 ggttcgggcg aaacgatttg ggatataaag aaaaatatcg gctatgtgag cagccaatta

1021 catatggatt atcgggtgaa ttgctctgcg ttagacgtga ttttatccgg cttttttgat

1081 tcaatcggcg tttatcaaca agtaccgagt gccttacagc taaaagcaat ggaatggctg

1141 gaacgcttgc atttagccaa tctggcgaaa aaaccgttcc gttcactttc gtgggggcaa

1201 caacggttat tattgattac tcgtgctatg gtaaaacacc cgccgattct gattttagac

1261 gaaccgctgc aaggtttgga cggtgtaaac cgcaaattgg ttaaacaatt tatcgaacag

1321 cttgtgacta atagtcaaac ccagttgcta tttgtttcgc accaagatgc ggacgcccca

1381 aattgtatca cccatttatt tgaatttgtt ccgcaaacta acggtggtta ccgttatgtg

1441 cagacggcgt taaattaggt ttttgacctt taaaggaaat ccccctcttt agtaaagagg

1501 ggggatgtgt gggactttaa agcgttgaat tatagaactt ataagcctcg tccatattct

1561 caaatttata cataatccct ttatctaata ccattgcatt atcgcaataa gacttcattg

1621 ctgacggact atgcgaaacc aaaataatcg aacgatcttt gcgcttttca aataattcat

1681 gtttacattt tgccgcaaag cgagagtcac ctaccgcaat tacctcatca attaagtagc

1741 aatcaaactc taccgaaagc gacaaagcaa aggcaagtcg ggctttcatg ccggaggaat

1801 atttcttaac cggctcatat aaataatcac ccaattcgga aaattcttcg gtaaaggctt

1861 taacgtattc aatatccgca ttatatatac ggcaaataaa gcgtaaatta tccataccgg

1921 ttaaactgcc ttggaacgcc ccgctgaaag cgagcggcca agatatcgac atattacgtt

1981 cgatagtacc tgatgttggc ggctcaacac cacttaacaa acggattagc gttgatttcc

2041 ctgcaccgtt acgccctaaa ataccgattt tctcgccttt tttcagctca aaattaatat

2101 cttgcaatac ggttttttta ccgcttcgag tatagtaatc tttactcaca ttttttacgc

2161 taatcattgc ggttcgattc ctttactgaa gttttttacc ataatgagcc caaaaagtaa

2221 catggctaca tcacatatta cgagatagct tatactttca tatgtgataa cactgtcgcc

2281 aaaataaccg tgacgaaaca tttccgtgcc gtgaatcatc ggtattaagg ttgcatattg

2341 ttgagcttgg cttggtagcg catgcacaaa gaaaaatgcg cctgaaagag gtaaaagaac

2401 aaagcttaat gttccccaga ttttgccaaa tgcttcaaat ttttgtgcaa tagaacaaat

2461 aatcaagcct aatcctaatg caaaaaatgc cattaatacc cacgccataa ccatataaaa

2521 cgtatctttc ggcatttcta tccagcctaa taaaatgact aatgccataa taatgatttg

2581 ggcaatcgtt gcacccgcta cctcaagtat gacacgagcc agtaaggtat ctaatacgcg

2641 aacattacga tgataaagaa gactcaagtt accggaaatt gcaccgatag tgcggtttga

2701 tgcattacgc cacatcattg ccattggata accggtaatc acaaaagcaa taatatttaa

2761 atcggaaacg cgatccgctc ggataaattt ccacatcaaa acgataaata aagtgagtaa

2821 tagcggctca acaaacagcc ataaaaaacc caaatttttt cgtccgtaac gcgtaataat

2881 ttcccgcatg agtaatgcac cgattactct cccttgaatg gcgagagatt ggcggaaagt

2941 tgtttgatca ccgtattgca ttagtttttg tgctctctta cgcttgcaat taataaactt

3001 aatacaccat aaagcatcag accgataaag aatgtcgcta aaatattata taagcgataa

3061 ggctcttccg cccagtccgg tttgcttggc tgactgatta cttctaaata aagttgctgg

3121 cgatccgctt catttttcgt attttgtaat gaggttaatg ctgcggtcaa ttgttgctgt

3181 gccagctcgt ttgcaagtac taagcgttgg taatcggcag tttgaatagc aatagagcta

3241 ttactgttac tggaaagctg ttttgattgc tcatcgattt ccttacgtaa actttttgg

3301 cgcataagca atgcatcaac ttgcgggttg tccggtgtaa tagattgcaa ttgagccaat

3361 tgtgtttcta cacgaatcaa ttcgcttttt aagctggaaa ttaatgaaag ttgtacgccg

3421 gattgtgccg gtaaatcaaa gattttattt ttgatacggt atttacttaa gtcgcttgcc

3481 gttgcattta cattattttc cgcttcctta accgcttgtt ccgcaaatga aatggtatct

3541 tttcttgcac gttcgtttaa acggttgatg agtgtttcac cttcggcaag taatttttga

3601 ttaatttgtt gtccctcttc tgcattaaaa gcacgaatac gtaagctggc aataccggat

3661 acagaatcga aatcaacact taagcgatct cggaaatatt tgtaaaacgc ttctttacta

3721 ttatttaaac caaatccatt aaagcgagcg ataatatcgc cttgattctc atagtattca

3781 cgtattggta ggtctttcat taactgttct aatgccgtac gagaatgcat atattcttgt

3841 acggtataag tatcatcttg agcacgagaa aatccggaac cttgtaataa ggccccgaca

3901 ccggttaaag cggtctgatt tttaggagat cttacaacga agcttgattc cgaaatataa

3961 atatcggaag caacagaacc gaaataaaag gctgataata ccgtcggaat cgctacagtt

4021 acccaaaata acggattaag cttttttaac caactttttt tctgtttaat cggtttctgt

4081 agtttttctg ctggactggt agcaataggt gtttccatct tttgtcctta tacattcaat

4141 atattaatag gcacgaacgg cattggtcgt actggtaacc ggcgaagtaa tcgagaaaat

4201 cattctcaag aatttttgga attcagacaa cggtgcattt gatacataga caatatcttt

4261 atcttgcatc gggaagcgtt gtaataaaaa tagtgattgc ggctcaagta aattcacacg

4321 ataaaccgtc ggtacatcca tacccatacc atagcctttc gatccccatt gcgcttgctg

4381 atctaaactc aattgagaaa aaggaacatg acggaagacg aatacccctc tcggatcgga

4441 acgggtatcg attaagcctc ccatcttacc gatagcttcg gcaagcgtaa tacctttact

4501 tgagaatttc atttgctggt tgttacccac cgcacctaaa ccggtaaaac tataaggtgt

4561 gtttagcagt gaaacaacat cgccagcacg taacataata ttttgtgccg gatcggaaat

4621 taacgtttcg aatgcgagtg tttttacttc agaaccacgg gttagcttga ccgtcacatc

4681 ttcaatgttt tccgttgttc cgcctactgc agcaaccgca tctaatacac gttcattatt

4741 agcggttaat ggcatacgaa tactattgcc ttgacgaata accgtaacat cagcagagtt

4801 attattcgca attttgacta atgcttgcgg ctgattcgct ttacgctgca atgccccaac

4861 aatttgagac tgaatcgttt caggtgtttt acctgcgaca cgaatatttc ccacaaacgg

4921 cacggttacc gtcccgtttt gattaaccat ttgtgccggt aattgcgtta aatgcccgct

4981 accttgtcct tcggaactaa aagtaccgcc aaacaacact gccggcggcg cttcccaaat

5041 tgaaatttca agtacatcac ccacattgac tgcaccggca tagcccgcgc tggctactgt

5101 gcctaaaaat ccggaaaatt gttggctttg ctgagtttga tacaactgtt gaactaaacc

5161 gttatccagt tccaccacat ttacttccgg taagggttta tccgaacttt gtgaattagc

5221 ctctaagatc gcactatggc tagggcctga agttgggagg cttgagcaag cagaaaggaa

5281 caatgttgtt aaagctaacc ctatgattga attatatttt ttaatttcca tcttgtaaaa

5341 aggctctatt gaaaaagtgt gtaaaatgaa tataaactat acataattat agatacaaca

5401 ctataattat atattataat ctaatatctc gtttttaaa acgataacct aaaatattac

5461 ttttctatag aagaataggc attattttaa taaatataat taggtatgaa tgttgatgaa

5521 aatagcattt atcgcttgga atagttttca ggtgttacat ttcaagccct tattacaagc

5581 tttaccgtgt gcattattaa ttattgaaaa acggagacgt agtgtaccaa tctgtaagga

5641 tattttgcga gatataaaca ataatatcgc ttatatccgc catacggata tatatgcaaa

5701 aattgatggg aattttgatg ttctagttgc tcaaactact ttcgagcaac tttatttgtt

5761 tcaccgcacc aaaattgcat tgcttcaata cggatatgct aaggaaccgt ataactacgg

5821 cacttggaga gcatttgcag atctaaattt ggtttatggg aattatgcct atgaacgtat

5881 ttcctatttc tctccaacta aaataaccgg ttgtccacga tacgatttat ggtatcagcc

5941 tttatttcat caaaaagcga aagaaaatta tgcgagagta ttagatacga gtaagaaaac

6001 gattgtatat gcaccaagtt ggggagaatt atccagcttt aaattatata tagaagaaat

6061 tacgaaatta tctttatttt acaacgtgtt agtaaaatta caccataaca cgcttttatt

6121 agcaaacaag catcagaatt atgaaaaatt gtatccgaat ttacattttt tctatgaagg

6181 tgaagatctt ctctcactta tttcggtagc cgacattgtt atttccgatt ttagcggtgc

6241 gatctttgac gcaatttttt gtaaaaaaac agtagtgtta ctttcgatat cgctagtgaa

6301 tcaaccaaaa ctagataaat ttagtttaga gatagcttat cgttcaaagt taggatatga

6361 ggttttttcg ccggatcaag tagctataac agtggcacga gcacttacag agccgaaatt

6421 agtagatgaa acgctgtatc aacagctttt tatgcataac aaggatgcaa cacagcaagt

6481 aataaatgct ttacaacagc ttgctgaggg taagtataca ttatctcaac agcagttata

6541 tgtgcgacaa acagaaaaat tattaaatat tgaaaaaata aagcagcaaa aaaataaaaa

6601 acagtctttc aataaaataa gacagatttc taaaagatta attaaaaaat aatttttcat

6661 attttctttt tatatttttt tatttgacaa tacggagttt aggaatgaaa aaagtattaa

6721 cctatggaac ctttgattta ttacaccatg ggcatattcg tttattagaa agagcaagat

6781 cattaggcga tcaccttact gttgctattt ctaccgatca atttaactta ggaaaaggca

6841 aagtatgcgc ttatacttac gaagagagag cgcatatttt aaaagcaatc cgttatgtgg

6901 atgaagtaat tcctgaaaca aactgggagc aaaaagttga ggatgtaaaa aatcacgaaa

6961 ttgacgtatt tgtaatgggt gatgactggg aaggcaaatt tgacttctta gcagattact

7021 gcgaagtggt ttatttaccg agaacccctg atatttcaac gactcaggta aaaaaaatgc

7081 ttgcgaaaaa agatctcgca gccggacaaa aacaaattca cgaaaaagag taatttgtta

7141 atgtttcaaa tcctacaaaa gcatttaccg accttgcaaa gagttctgag ggagggttac

7201 tcccagcatt ctttgcttgc ttattggtat gggcttagtc tacttactgc tcttgaacag

7261 gcgaatcatc ctcaagtaag aaaactggct gagaaaatga tcaataaagg tattaatatc

7321 gggcattatt ttttagcaca aagttatttc ttatgtggag aatatgattt agcggaacaa

7381 gcggtcaaaa aaatcaaaaa ttttgtaaaa atacccgaag ttgttttttt atatgcggac

7441 attctcgtta aatgcaaacg taaagaagag gcttggcaat tattagaaca atgcgcttta

7501 ctcaataaaa gaaaaaaagt gtggatacat ctaacaaatt tagtaaatac tgaggcggat

7561 tatcgacact tagaacaaca tattgacaaa gtaagaacaa ctacacctta cttgaagtct

7621 gatttgttaa ttcatcaaag aacaaatgca gcattaaggg ctggtttaac agaaacggca

7681 ttagcactaa cagaacttaa ccctttgcca aagcaagcaa aagtgaagaa aaaaacaacc

7741 gcttataatg ataaattagc ggcaattgcg ctagcggatc taaagaaagt attagatcac

7801 aaaaaaatac ctttctttct gattagcggc acgttgctag gttgtattcg agaaggaaaa

7861 ttattagggc atgataaaga tattgatgta ggtgtttggg acgaatattc ctacgaagag

7921 ctagcaaatt atttagcaac ttcaggttat ttttatgttg tacccacccg aacaaaacat

7981 ttagtaatgc tacggcacgt aaacggcatt gctattgatg tgtttattca ttatcgagaa

8041 cctaatgact attggcacgc cggtgtcaaa ataaaatggc ataactcgcc atttaactta

8101 gtgtataaag attttctcgg acaacaatat ttaatacctg aaaattacga tttataccta

8161 acagaaaatt acggtgattg gcgcacgcca aaaactaaat ttgatagcgc ctttgatacg

8221 ccaaatatgg aagtgatcaa tgaagctgaa atgcaaattt atgttattaa aaagcgagaa

8281 ctttaaaatg aaacataatg taaaattatc ttctctgcca agtttattac aaggcttata

8341 tttatataaa catcaccaat atcaaaaagc acagaaaatt tttagcaaaa ttttagaaaa

8401 gcaacctaaa aatgcctatc taaatttcag atatggaatg tctttatata aagataaaaa

8461 atggaatgag gcaaattatt ttattcaaaa agcagttgag cttgctccag aacaagagtc

8521 gtggaaaaaa caattagcaa caacagaacg ttataaaaat gattcatcaa aaattaaagt

8581 tgctgaaaat aagaagaatt tagaaaaaca accaaatagc ccagaatata tttgggaata

8641 tgctgtatct ttaattgata gtaaacaata ttggttagca caattccaat tagaaaaata

8701 tattgaatta agacctgact ccgaacgtgc tttttaccag ttaggaattg ttgcggaaaa

8761 attagcgaat tatgaacaag cattttcata ttttcaaaaa gcaagccaat ttgacccgct

8821 taatcgtaat tataaatatc gtatggggta caatctcgaa aaacttggac atttagatca

8881 agcgagaatg tgctatgacc ttgttacgag tttgagtaat gcgggtgatg aagttattga

8941 ttttggcatt ggggcattac acgctaaacg agggctatgg gacttagctg tatctgctta

9001 tttagaattc caagttaaaa caggttctca aaacccagaa ttattttacc gcattggtct

9061 tgctaatgag cgtttatatc aatgggctga atcagcaacc gcttttgagc aagcagttac

9121 gttatctgaa gtaattaatg caaattggtg tttcaaatgt ggacaagctt atgaaagagc

9181 tgggaatttt gagaaatctg ctgaatttta ccaagaagca gttaagagat ccgatcatta

9241 taatgattat tggtggtatc gcttagcttt agtattagag aaactcggta aatatgaaca

9301 atctgttagt gctttccaaa actcaagacg acgtaaactt gcttattccg ttaatcccaa

9361 aaatgttatt aagcataaag aagaagagta tctttcatac tacacagaat attatgaaac

9421 cttagaatta gatgagaaat tagtattaat tgaaagtttc tttggtggaa atattagttg

9481 taatccatac gctattctat cttatatgct aggaaataat tatgactata cttatgttgt

9541 tgtaattaaa gatggaactg ttatccctga taacctcaaa tttaatagaa aaattatttt

9601 tattaaacga ggatcagatg cgtatttacg ttatttatgt acagctaaat atcttattaa

9661 taatgttagt ttcccttatt attttattag aaaagagggt cagatttatt taaatacttg

9721 gcacggtacg ccaatgaaga cattaggaaa agatattaag aatccattta tggatcacgc

9781 taatgtgagt cgaaacttct tgcaagcaac acatattatt tctcctaatc gtcatacaac

9841 tgatattatt ttagagcaat atgatgttaa ggatttattt agtggaaaat tagccgaaac

9901 aggttatccg agaattgatt tggcatttaa tctgacaggt aagagacgtg aagaaatcaa

9961 agaaaaattg gggttatcta ataaaaaacc tgttgtattc tacgcgccaa cgtggcgagg

10021 cacatctcaa tcaaaagatt ttgatacaac gaaattacaa agtgatttaa aaaaacttaa

10081 atcagataaa tataatctga tttttagagg gcatcatcta gtagaacaat tattagaaac

10141 tattaattta gatgtaattg ttgctccgaa agatattgat agtaatgaat tactaggttt

10201 ctgtgattta ttgattactg actactcaag tattatttat gatttcttag cattaaataa

10261 accagcaatt agttatattt atgactatga agaatatgat gctgaacgag gtttatattt

10321 aaaacctgag gaaatgtcgg gtacggtttg cacaacgatt acagatgtta aaaatgctat

10381 tctagaaaat attgctttgg gtaaaactaa tgtatctgaa caagatatta ataaatattc

10441 ttatttagat gatggtaaag ctactaagcg tacggttgaa tttatgtttg atcgtgatga

10501 ttcttgtgtt tataagtatg agcgtaagaa atcagatata ttctttgaag gaccatttat

10561 tccaaatgga atttctcgtt cattcttaaa tttgatggca tcaattaaag attcagaaaa

10621 aaatattaca ttattaatta atggtgcaga tatagcacaa gatcaaaaac gtctagcaga

10681 atttgataat ctaccgtcaa atgttacggt tctctctaga gtgggtagaa ctcctatgac

10741 cttagaagaa ttatgggtga gaaataagtt tgaagaaacg tatcagatgt attcggagtc

10801 atttaccgag actttgttaa aagtatataa acgagaagtg cgtagattgt tgggtgattc

10861 actatttgaa aatgctattc attttgaggg ttattcatta ttttgggtat tattgttctc

10921 tcaaattaat gcgaaaaaac atattattta tcaacataat gataaatata aggaatggaa

10981 aggacgtttc ccgtatttag agggtgtatt caatagctac gtatttttg accagattgt

11041 ttctgtgtca gaaaaaacta tggagaataa tatattaaat ctttcaaaat catttaatat

11101 acctaaagaa aaatttactt tctgtaataa tcctatcaac attcagcaaa ttctctctag

11161 tgcagaggaa gatattgaaa tggaaagtga atttacctca ttcaatggtc agaagtttat

11221 caatattggt agaatgtcgc acgagaaaga tcaattaaaa ttaattgagg ctttctgtga

11281 agctaaaaaa gtacacgcta atattagatt atttattcta ggagatggcg tattaaaaca

11341 ggatttaact aataaaatta aagagctttc attagaaaaa gatgtgtatc ttcttggaca

11401 gaagaaaaat ccattccctt atttaaaaca agcggatgta tttattcttt cttcaaacca

11461 cgaaggacaa ccaatggttc tattagaatc tcttacatta ggtactccaa ttattgctac

11521 cgatattgta ggtaatcgca gtattctagg agataattat ggagtgttag tcgaaaatag

11581 taaggatgga ttagttaaag gtattaatat ttatatggag caaggtggtc gtaaagatag

11641 ttttgatccg tatgaatatc aaaatgatgc tatggcgaag ttttattcat tattaacaaa

11701 ttgaaatatt aaggataaat aatgttagat tttttcagaa ttaaatttga tcaaaacact

11761 aaatgtgatg agttaaatat cgatttatca gtaaaagaaa tttatattga taaatcggct

11821 attggtaaag atgaaaaaga aaagttaaat cataatttct tagcagtagg acgtggtaat

11881 attgaggcta agaaacttat cgctaagttg tcaaataaca aatattattt aacagctcat

11941 tctaatggta tatctatttt cactaagttt agtgatggta aatcatttat ttcacatttt

12001 aagaacaaga atgtaaaagt atggaatgat actttttata cattggatga gcctttagaa

12061 aaagatacat caataaatcg tttattagta gtttttcat ctattgcaga tttagcattt

12121 aatgcttcta ttgaaagacg aatgtttttt actaattttc cgagtgtagg aaaatatatt

12181 cctaagaata catatatttt acgcattgca gatattggtg gggtcttagg aagcttttat

12241 ttgaataata atgcggatat gaagtttgaa aataaggttg aaagtttaat tagaaaagtt

12301 caaatggaat gttccgtacc taatgaatat actgttttat acggtacatc taaaggtgca

12361 acaggcgcac tttaccacgg tgttaatatg aaattaaaaa cgttggcagt tgatccgatt

12421 gtccacgatg aacattatat taaaaagttt aatgatcttc attttgtgca aaatgttttc

12481 cctgaatcaa aacaggaaaa attttcaaaa ctatttcaga ataataagga cgagaattta

12541 agtcatatta aattagttac gtctaagaat tctgaacaat ttaactatat tagcgaaatt

12601 gtattttcag aaagtacgac tgtatgttca tatatattta ataacccgaa tattaaagga

12661 catacagata tgggagaaca tacactaaac tttgttactg ctatgttaaa taacctacta

12721 tatgatattg atgttaaatc aaatttaatt actgtttatt aatctgagac ataaaaatga

12781 ataaaacagc aatatgttta atggcaaata aggtatataa atttgcttta gcaacaatgc

12841 ttattaattt aaaagaaact aataacgatc aatatgataa tgttattgtt tatcacgatg

12901 gattttctca aactgaactt gatgatttat ctttacttga acctagggtt aagtttatcg

12961 aatatacttt tgaaaaatgg aatcaagaac actcagtgct attaggtgat aaggctaata

13021 actttcttaa caggttttct catttagctt ggtctaaata taaaattatt gagcagttag

13081 aacactatga aaaagttcta tatctagatt tagatatgct tgtttctggt tcattagaag

13141 gactcttcaa attagatgga attgcttgga gaaatggcgc aaactttgga gaaaaatttt

13201 caactaatgg tcttaaagtt aagaatattg ctgaagtaag taatattcct gaatctacac

13261 cagcacctaa tggtgggctt ttgtgtgtaa caaatactat cgattggaaa aagaatctaa

13321 tagaagcaaa atattttata gaaagattta ttaatgattt ttcttttgca ttagatgaat

13381 tagcattttc ttatatatat gtaaaaaata acttaaaatt aactcaatta tccgcttatg

13441 aatataatgt tttacctcag catttaaatc caagttgtaa ggttattcat tttattgggc

13501 aatttaaacc gtggaaggat cttatcattc aagcagaatt tccaaaatgg tttgaatatt

13561 atcaaaaggc ttgtttactt actgataata ggatatcttc tgagcaagtc caagagtatc

13621 ccaaagatgg gaaatattta actaaaagat taaatgaaca gcgttggttt agttttttga

13681 gagaagcgaa tttaaatatc cctcaatcat taagacttcg atatgtattt gataatgaat

13741 ggcttatttt tgaatgtaat tcaaaagttt attatgagtt taaattccat caatatgctc

13801 aaggatttct tatgggtatg tgggttaaag acttatataa agagccagaa attaagcgag

13861 aaattgataa cttagtgggt aagaatccta gactatttag attacacgaa gatcatcgtg

13921 gtctatatat ttattctaca aaattttcag ctttagaaat tgcaccggtt tttaaagatt

13981 tctatgaaag aacatctaag ctatttaata taaaatgagt taaagaagat ttccattgct

14041 aagtaattac gataaagagt tcttttcttc attaaatatt gcttaatcta gtgatagaat

14101 atcaattaga atatatctat tttctatcaa gagtaaaaac atactatgtt caaaaaaatc

14161 acattattca gttttattgc gttaatcgcc ggttgttctt cttcctcaca accggaagct

14221 ttcccggggg agtttgcgaa tgcggattat gtgttatcgg ataaagatgc ccagcgttgg

14281 gtggtggcta gccgtcaggc ggagcagtgt atttatccga acttgacgcg gattcagcaa

14341 caagcgttta gtaaggaaga ttcatatatt cattcgcaat acgtattttt ctatccgttg

14401 gaagaaatta tcggcgagca gtatgtaaag attatccaag acgatgaaaa atctatggga

14461 tatgcgcaat acttgtttaa gaaattcaga gataatcagg aattcgagcc gttagcggat

14521 aagcaatgtc ttgtgttacg agaaaaagcg aagaacgatt tagcggtcgt aaaagggcag

14581 tataagagcg gaatggttga agaaacgacg tccgaagcta aaaatgcgga cggcgtggcg

14641 accaatcaaa ataaattctt ctttgatatt atcaaatggg gttcgatgct attactgtaa

14701 tttgcggtta gtgtgatgtt aaagagtata aaaacgagct gtcggtaaat cgttatcggc

14761 agctcttttt attagtttga tatttgtttg attaactcca ataactccgg taattccgtt

14821 actccctctc gatcgataaa atgcccgccc gttgccaagc gaatataatc cgcttgtaag

14881 tattgcgcta atcgatcgct gaacgaatgg ggaacgacaa catcatttaa tgcagatatg

14941 acgtaagact tttgcggtaa acaagcggtc tgatttgcat aaaaatctgc aaagctatct

15001 aattccggca aagttggtaa tttctcataa aagccggaaa caaaaattgc cgtttttact

15061 ttttgctgcg ttaccgcaag ataattcagt aacgcaatgc agcccaaact atgtccgatg

15121 agtaaggtat tttcatctaa ttgaagtgta ttttggtgat gttccagcca tgcttgcgga

15181 ttcggctgat cggaattcgg catcgctaaa cattcacatt cccatcctaa tttttccaat

15241 tcgtttttaa gccacggaaa ccaatttctt gtcgggttcg ccgtataacc gtgcgttaca

15301 tatacttttt tcat

//

LOCUS **ADOL01000042.1** 11941 bp DNA linear BCT 20-APR-2021

DEFINITION Actinobacillus pleuropneumoniae serovar 12 str. 1096
capsular polysaccharide gene locus, complete sequence.

ACCESSION ADOL01000042 REGION: complement(37908..49848)

VERSION ADOL01000042.1

KEYWORDS .

SOURCE Actinobacillus pleuropneumoniae serovar 12 str. 1096

ORGANISM Actinobacillus pleuropneumoniae serovar 12 str. 1096
Bacteria; Proteobacteria; Gammaproteobacteria; Pasteurellales;
Pasteurellaceae; Actinobacillus.

REFERENCE 1 (bases 1 to 11941)

AUTHORS Xu,Z., Chen,X., Li,L., Li,T., Wang,S., Chen,H. and Zhou,R.

TITLE Comparative genomic characterization of Actinobacillus
pleuropneumoniae

JOURNAL J. Bacteriol. (2010) In press

PUBMED 20802045

REMARK Publication Status: Available-Online prior to print

REFERENCE 2 (bases 1 to 11941)

AUTHORS Xu,Z., Zhou,R. and Chen,H.

TITLE Direct Submission

JOURNAL Submitted (04-MAY-2010) College of Veterinary Medicine, Huazhong

Agricultural University, Shizishan Street 1, Wuhan 430070, China

FEATURES Location/Qualifiers

source 1..11941

/organism="Actinobacillus pleuropneumoniae serovar 12 str.

1096"

/mol_type="genomic DNA"

/submitter_seqid="contig00058"

/strain="1096"

/serovar="12"

/note="K locus: KL12"

/db_xref="taxon:754261"

/note="Coverage of the contig is 19.69X"

CDS 1..1473

/gene="modF"

/locus_tag="appser12_16200"

/note="similar to ABC-type molybdenum transport system,

ATPase component/photorepair protein PhrA COG1119;

similar to ABC transporter related protein of Mannheimia

haemolytica serotype A2 str. OVINE UniRef

RepID=UPI0001BCFBC9"

/codon_start=1

/transl_table=11

/product="ABC transporter protein"

/protein_id="EFN00073.1"

/translation="MPNINIHNALFSLAQHNKLSIESLEINTHDFWVIVGGNGSGKTA

FAQALHNSLSLYSGEYQNSFQHIALLSFEQQQKIIEQIFKHRNNDMVSPDDFGLTARQ

IILNGSEKMQLCEEYAAKLRIQPLLDRPFIQLSTGESRKVLFCQMLVSEPDLLILDEP

FEGLDQASVAYWQDVMAQLGKQMAVVLISNRFNDIPDCATHIALLDNLQLILQGERQE

IEQQAVYSQLKFAEQNVNAPLPESAAPLIQLPPNTNPFELKNVMIRYGEKNIIDDLTW

TVAPKQHWWIKGPNGAGKSTLLSIITGDHPQSYANYVHLFGRQRGSGETIWDIKKNIG

YVSSQLHMDYRVNCSALDVILSGFFDSIGVYQQVPSALQLKAMEWLERLHLANLAKKP

FRSLSWGQQRLLLITRAMVKHPPILILDEPLQGLDGVNRKLVKQFIEQLVTNSQTQLL

FVSHQDADAPNCITHLFEFVPQENGGYRYVQTALNQIENA"

CDS complement(1823..2473)

/gene="cpxA"

/locus_tag="appser12_16190"

/note="similar to ABC-type polysaccharide/polyol phosphate
transport system, ATPase component COG1134;
similar to ATP-binding protein bexA of Bacteria UniRef
RepID=BEXA_HAEIN"
/codon_start=1
/transl_table=11
/product="ATP-binding protein bexA"
/protein_id="EFN00072.1"
/translation="MISVKNVSKDYYTRSGKKTVLQDINFELKKGEKIGILGRNGAGK
STLIRLLSGVEPPTSGTIERNMSISWPLAFSGAFQGSLTGMDNLRFICRIYNADIEYV
KAFTEEFSELGDYLYEPVKKYSSGMKARLAFALSLSVEFDCYLIDEVIAVGDSRFAAK
CKHELFEKRKDRSIILVSHSPSAMKSYCDNAMVLDKGIMYKFENMDEAYKFYNSTF"

CDS complement(2470..3267)
/gene="cpxB"
/locus_tag="appser12_16180"
/note="similar to ABC-type polysaccharide/polyol phosphate
export systems, permease component COG1682;
similar to Capsule polysaccharide export inner-membrane
protein bexB of Proteobacteria UniRef RepID=BEXB2_HAEIN"
/codon_start=1
/transl_table=11
/product="Capsule polysaccharide export inner-membrane
protein bexB"
/protein_id="EFN00071.1"
/translation="MQYGDQTTFRQSLAIQGRVIGALLMREIITRYGRKNLGFLWLFV
EPLLLTLFIVLMWKFIRADRVSDLNIIAFVITGYPMAMMWRNASNRTIGAISGNLSLL
YHRNVRVLDTLLARVILEVAGATIAQIIIMALVILLGWIEMPKDTFYMVMAWVLMAFF
ALGLGLIICSIAQKFEAFGKIWGTLSFVLLPLSGAFFFVHALPSQAQQYATLIPMIHG
TEMFRHGYFGDSVITYESISYLVICDVAMLLFGLIMVKNFSKGIEPQ"

CDS complement(3267..4307)
/gene="cpxC"
/locus_tag="appser12_16170"
/note="similar to Capsule polysaccharide export protein
COG3524;
similar to Capsule polysaccharide export inner-membrane
protein ctrB of Proteobacteria UniRef RepID=CTRB_NEIMA"
/codon_start=1
/transl_table=11
/product="Capsule polysaccharide export inner-membrane

protein ctrB"

/protein_id="EFN00070.1"

/translation="MLSAFYFGSVASDIYISESSFVVRSPKNQTALTGVGALLQGSGF

SRAQDDTYTVQEYMHSRTALEQLMKDLPIREYYENQGDIIARFNGFGLNNSKEAFYKY

FRDRLSVDFDSVSGIASLRIRAFNAEEGQQINQKLLAEGETLINRLNERARKDTISFA

EQAVKEAENNVNATASDLSKYRIKNKIFDLPAQSGVQLSLISSLKSELIRVETQLAQL

QSITPDNPQVDALLMRQKSLRKEIDEQSKQLSSNSNSSIAIQTADYQRLVLANELAQQ

QLTAALTSLQNTKNEADRQQLYLEVISQPSKPDWAEEPYRLYNILATFFIGLMLYGVL

SLLIASVREHKN"

CDS complement(4449..5600)

/gene="cpxD"

/locus_tag="appser12_16160"

/note="similar to Periplasmic protein involved in

polysaccharide export, contains SLBB domain of b-grasp

fold COG1596;

similar to Capsule polysaccharide export protein bexD of

Proteobacteria UniRef RepID=BEXD_HAEIN"

/codon_start=1

/transl_table=11

/product="Capsule polysaccharide export protein bexD"

/protein_id="EFN00069.1"

/translation="MGLVASLAACSSLPTSGPSHTAVLEANSRNSDKPLPEVNLVELD

NGLVQQLYQTQQSQQFSGFLGTVGSAGYAGAVNVGDVLEISIWEAPPAVLFGGTFSSE

GQGSGHLTQLPAQMVNQNGTVTVPFVGNIRVAGKTPEAIQSQIIGALQRKANHPQALV

KIANNNSADVTVIRQGNSIRMPLTANNERVLDAVAAVGGTTENIEDVTVKLTRGSEVK

TLAFETLISDPAQNIMLRAGDVVSLLNTPYSFTGLGAVGNNQQMKFSSKGITLAEAIG

KMGGLIDTRSDPRGVFVFRHVPFAQLSLEQQAQWQAKGYAIGMDVPTVYRVNLLEPQS

MFLLQRFPMQDKDIVYVSNAPLSEFQKFLRMIFSITSPVTSTTNAVRAY"

CDS 5867..6982

/gene="cps12A"

/locus_tag="appser12_16150"

/note="similar to Capsular polysaccharide

phosphotransferase of Proteobacteria UniRef

RepID=LCBA_NEIMB"

/codon_start=1

/transl_table=11

/product="Capsular polysaccharide phosphotransferase"

/protein_id="EFN00068.1"

/translation="MNKMNRKFSKLLKNPHIFFRDFLNKKYPIKNTELPFSESEEANL

IEANQKLDKIIQKNTLQQANIDVVFTWVDGSDPSWQAKYSQYAPNYQAKSALYATDIA
RFEDHNELYYSVHAVLKYMPWVRHIFIITDNQKPKWLDETRQEKITLIDHQDIIDKEY
LPTFNSHVIEAFLHKIPNLSENFIYFNDDVFIARELQAEHFFQANGIASIFVSEKSLS
KMRDKGIITPTLSASEYSIRLLNKYYDTNIDSPLVHTYIPLKKSMYELAWLRYEKAIL
GFLPNKLRTNNDLNFANFLIPWLMYFEGKAMPKIDICYYFNIRSPNAISLYKKLLLKQ
QMGEEPNSFCANDFNSNYSIENYRNNLISTLNNYYKF"

CDS 7377..9617

/gene="cps12B"'

/locus_tag="appser12_16140"

/note="similar to Putative glycosyl/glycerophosphate
transferases involved in teichoic acid biosynthesis
TagF/TagB/EpsJ/RodC COG1887;
similar to Cps12B of Proteobacteria UniRef
RepID=Q69AA8_ACTPL"

/codon_start=1

/transl_table=11

/product="Cps12B"

/protein_id="EFN00067.1"

/translation="MKTEWVTFIDPDDFLDLNYFYLLNDTLEKYDHIGAFVTKFKLFK
EKFGTYHDGFQTDFCFTKPIRVLKANDMEDCVQFSSSSSVYRTDVIHKNKILFDEKLT
ASFEDTKFFYDYLYNIKESNILYIKDAIYNYRLRSNESSSSNSQWTKKAKYQEFFQFG
LLSVIKKYNENGTIPTFIQRLVLFSIIPYLQVAMINKKRIEDVLNESEINNLLQSIKE
CLSYVETNTLEKFYNSPGNYFWINAINNYFYNKLPIDKRVYINKVNLDENKVYFRFYG
IKGKTKFSLKVNNKHLKTSSERVIEYKLFSDNLINEYNICYHIEPNKKIEFLLDGEKA
KIYTDFKILSDKDTDFYKGYISKNNSLKNIALFIDSGYKADDNAEHLYEKLLKNKNLD
NFIDDHYYLLDKESEHWNRLILKGFNLVDIKSMKGVWLMKNAKYIFCSYLPGHLNEWA
THHSFKFQKFIFLQHGIITSNLSKPFNASYSQIYKMVISSKFEKSEILDDKFNYIFHS
NDLILSTIPRLDKLVNHKRNQSNKVKKILVCPTWRTSLGNINFNKKDAISSFKETSYI
KNWLGLLYSDKLRNYLEEGKIEISFLPHQNFHQLLEENSLNEKLFFDINENIRILNPK
KSSYQELFIDHDILITDFSSLHFDFATLQKDILYFQFDKDEFYGISHAYQKGLFNFEK
DGFGQVTYTLEELLDQLVILINSQNEKVVNGYKKRISNVFLPSLGDSCNYILKNVFTN
PKRNIN"

CDS 10332..10448

/locus_tag="appser12_16130"

/codon_start=1

/transl_table=11

/product="hypothetical protein"

/protein_id="EFN00066.1"

/translation="MMKKLFLAALIASFGLAACGVKGPLYFLEQQPAQQQTK"

CDS 10571..11176

/locus_tag="appser12_16120"

/note="similar to Diaminopimelate decarboxylase COG0019;

similar to Diaminopimelate decarboxylase of Proteobacteria

UniRef RepID=DCDA_HAEIN"

/codon_start=1

/transl_table=11

/product="Diaminopimelate decarboxylase"

/protein_id="EFN00065.1"

/translation="MNHFNYKNQQLFAEDVSVSDIINQYGTPAYIYSRATLERHWHAF

DKAFGAHPHLICFAVKSNSNIALLNVMARLGSGFDIVSQGELERVLAAGGKPSKVVFS

GVAKSHSEIQRALEVGIRCFNIESIAELHRINEVAGQLGKIAPISLRVNPDVDAHTHP

YISTGLKENKFGVSVTNEGNKYWEKRYEKKHRRKTYCVKVR"

CDS complement(11399..11941)

/gene="ydeN"

/locus_tag="appser12_16110"

/note="similar to Predicted esterase of the alpha/beta

hydrolase fold COG3545;

similar to Putative uncharacterized protein of

Actinobacillus minor UniRef RepID=B9CWM4_9PAST"

/codon_start=1

/transl_table=11

/product="hypothetical protein"

/protein_id="EFN00064.1"

/translation="MKKVYVTHGYTANPTRNWFPWLKNELEKLGWECECLAMPNSDQP

NPQAWLEHHQNTLQLDENTLLIGHSLGCIALLNYLAVTQQKVKTAIFVSGFYEKLPTL

PELDSFADFYANQTACLPQKSYVISALNDVVVPHSFSDRLAQYLQADYIRLATGGHFV

DREGVTELPVLLELLKQILK"

ORIGIN

1 atgccaaaca tcaacattca caacgcctta ttttcccttg ctcaacacaa taaactctcg

61 attgaatcgc tggaaatcaa tactcacgat ttctgggtga ttgtcggcgg taacggctcg

121 ggtaaaaccg ctttcgccca agcgctacac aattcacttt cgttatattc gggcgaatat

181 caaaatagtt tccagcatat cgctttactt tccttcgagc agcaacaaaa aatcatcgag

241 caaatcttta aacaccgtaa caatgatatg gtttcgcctg acgatttcgg tttaaccgct

301 cgtcaaatta tcttaaacgg tagcgaaaaa atgcaattat gcgaggaata tgcggctaaa

361 ttacgtattc agccgttatt agatcgcccg tttattcagc tctccaccgg cgaaagtcgc

421 aaggtgttat tttgccaaat gttagttagc gaaccggatt tgctgatttt agatgagcct

481 tttgaggggt tagaccaagc ctcggtcgct tattggcaag acgtgatggc acaactcggc

541 aagcaaatgg cggtggtact gatttccaac cgttttaatg atattcccga ctgtgccaca

601 catattgctt tactggataa cttacaactg attttacaag gcgaacgcca agagattgaa

661 caacaagcgg tctattctca gctaaaattt gcagaacaga atgtgaatgc accgttgccg

721 gagagtgccg caccgctgat tcaactccca ccgaatacta atccgtttga actgaaaaac

781 gtgatgattc gttacggcga aaaaaacatt atcgatgatc taacttggac ggttgcccca

841 aaacaacatt ggtggattaa aggtccgaac ggagcaggaa aatcgacctt actttctatt

901 attaccggcg atcatccgca atcttatgct aactacgtgc atttattcgg tcgtcagcgt

961 ggctcgggcg aaaccatttg ggatatcaag aaaaatatcg gctatgtgag cagccaatta

1021 catatggatt atcgggtgaa ttgctctgcg ttagacgtga ttttatccgg cttttttgat

1081 tcaatcggtg tttaccaaca agtgccaagt gcgttacagc tcaaagcaat ggaatggctg

1141 gaacgcttgc atttagccaa tctggcgaaa aaaccgttcc gttcactttc gtgggggcaa

1201 caacggttat tattgattac tcgtgctatg gtaaaacacc cgccgattct gattttagac

1261 gaaccgctac aaggcttgga cggtgtaaac cgcaaattgg ttaaacagtt tatcgagcag

1321 ttggtcacca atagccaaac tcagttgcta tttgtttcgc accaagatgc ggacgcccca

1381 aattgcatca cgcatttatt tgaatttgtt ccgcaagaga atggtggtta tcgctatgta

1441 cagacggctt tgaatcaaat agaaaacgcc taagatttaa ccacggaaaa cacggattac

1501 acggagttta atgaatcggt cagttgacgt aatgtttgga taatattgtt tttttcaggt

1561 ttttcttatc tcgtaaggaa ctaataaaac tttaatcgga aactgcaact actcgcaata

1621 tcataatttg ttaaccttta aaggaaatcc ccctctttag taaagagggg ttaggggaga

1681 tttgtcaata gagatatgaa attgaataga acttcatttt ttatatttat aaaagcgtta

1741 attagcatat ttcttcgcta attcattctg tcaaatctcc tcctgcccct ctttgctaaa

1801 gaggggagat atgtgcggga ctttaaaacg ttgaattata gaacttataa gcctcgtcca

1861 tattctcaaa tttatacata atccctttat ctaataccat tgcattatcg caataagact

1921 tcattgctga cggactatgc gaaaccaaaa taatcgaacg atctttgcgc ttttcaaata

1981 attcatgttt acattttgcc gcaaagcgag agtcacctac cgcaattacc tcatcaatta

2041 agtagcaatc aaactctacc gaaagcgaca aagcaaaggc aagtctggct ttcatgccgg

2101 aggaatattt cttaaccggc tcatataaat aatcacccaa ttcggaaaat tcttcggtaa

2161 aggctttaac gtattcaata tccgcattat atatacggca aataaagcgt aaattatcca

2221 taccggttaa actgccttgg aacgccccgc tgaaagcgag cggccaagat atcgacatat

2281 tacgttcgat agtacctgat gttggcggct caacaccact taacaaacgg attagcgttg

2341 atttccctgc accgttacgc cctaaaatac cgattttctc gcctttttc agctcaaaat

2401 taatatcttg caatacggtt tttttaccgc ttcgagtata gtaatcttta ctcacatttt

2461 ttacgctaat cattgcggtt cgattccttt actgaagttt tttaccataa tgagcccaaa

2521 aagtaacatg gctacatcac atattacgag atagcttata ctttcatatg tgataacact

2581 gtcgccaaaa taaccgtgac gaaacatttc cgtgccgtga atcatcggta ttaaggttgc

2641 atattgttga gcttggcttg gtagcgcatg cacaaagaaa aatgcgcctg aaagaggtaa

2701 aagaacaaag cttaatgttc cccagatttt gccaaatgct tcaaattttt gtgcaataga

2761 acaaataatc aagcctaatc ctaatgcaaa aaatgccatt aatacccacg ccataaccat

2821 ataaaacgta tctttcggca tttctatcca gcctaataaa atgactaatg ccataataat

2881 gatttgggca atcgttgcac ccgctacctc aagtatgaca cgagccagta aggtatctaa

2941 tacgcgaaca ttacgatgat aaagaagact caaattaccg gaaattgcac cgatagtgcg

3001 gtttgatgca ttacgccaca tcatggccat tggataaccg gtaatcacaa aagcaataat

3061 atttaaatcg gaaacgcgat ccgctcggat aaatttccac atcaaaacga taaataaagt

3121 gagtaatagc ggctcaacaa acagccacaa aaaccccaag ttttttcgtc cgtaacgcgt

3181 aataatttcc cgcatgagta acgcaccgat tactcttcct tgaatggcga gagattggcg

3241 gaaagttgtt tgatcaccat attgcattag ttttgtgct cccttacgct tgcaattaat

3301 aaacttaata caccataaag catcagaccg ataaagaatg tcgctaaaat attatataag

3361 cgataaggct cttccgccca gtccggtttg cttggctgac tgattacttc taaataaagt

3421 tgctggcgat ccgcttcgtt ttttgtattt tgcaaagagg ttaaggctgc ggtcaattgt

3481 tgctgtgcca gctcgtttgc aagtactaag cgttggtaat cggcagtttg aatagcaata

3541 gagctattac tgttactgga aagctgtttt gattgctcat cgatttcctt acgtaaactt

3601 ttttggcgca taagcaatgc atcaacttgt gggttgtccg gtgtaataga ttgcaattga

3661 gccaattgtg tttctacacg aatcaattcg ctttttaggc tggaaattaa tgaaagttgt

3721 acgccggatt gtgccggtaa atcaaagatt ttatttttga tacgatattt acttaagtcg

3781 cttgccgttg cgtttacatt attttccgct tccttaaccg cttgttccgc aaatgaaatg

3841 gtatcttttc ttgcacgttc gtttaaacgg ttgatgagtg tttcaccttc ggcaagtaat

3901 ttttgattaa tttgttgtcc ctcttctgca ttaaaagcac gaatacgtaa gctggcaata

3961 ccggatacag aatcgaaatc aacacttaag cgatctcgga aatatttgta aaacgcttct

4021 ttactattat ttaaaccaaa tccattaaag cgagcgataa tatcgccttg attctcatag

4081 tattcacgta ttggtaggtc tttcattaac tgttctaatg ccgtacgaga atgcatatat

4141 tcttgtacgg tataagtatc atcttgagca cgagaaaatc cggaaccttg taataaggcc

4201 ccgacaccgg ttaaagcggt ctgattttta ggcgatctaa cgacaaaact tgattccgaa

4261 atataaatat cagaagcgac cgaaccgaaa taaaacgctg atagcacggt agggattgcg

4321 acagttatcc aaaataatgg attgagtttt ttaaagcggc ttttttctgt ttaaccggtt

4381 tctgtaattt ttctgctgga ctggtagcaa taggtgtttc catcttttgt ccttatacat

4441 tcaatatatt aataggcacg aacggcattg gtcgtactgg taaccggcga agtaattgag

4501 aaaatcattc tcaagaattt ttggaattca gacaacggcg catttgaaac atacacaata

4561 tctttatctt gcattgggaa acgctgtaat aaaaacatgg attgcggctc aagtaagttc

4621 acacgataaa ccgttggtac atccattcct atagcgtagc ctttagcttg ccattgtgct

4681 tgttgttcca aactcaattg tgcaaaaggc acgtgacgga atacgaaaac ccctctcgga

4741 tccgaacgag tatcaattaa accgcccatc ttaccgatag cttcggcaag cgtaattcct

4801 ttacttgaga atttcatttg ctggttgtta cccaccgcac ctaaaccggt aaagctataa

4861 ggcgtgttta gcaacgaaac gacatcgccg gcacgtaaca taatattttg cgccggatcg

4921 gaaattaacg tttcgaatgc gagtgttttt acttcagaac cacgggttag cttgaccgtc

4981 acatcttcaa tgttttccgt tgttccgcct actgcagcaa ccgcatctaa tacacgttca

5041 ttattagcgg ttaatggcat acgaatacta ttgccttgac gaataaccgt aacatcagca

5101 gagttattat tcgcaatttt gactaatgct tgcggatgat tcgctttgcg ctgtagtgct

5161 ccaataattt gagactgaat cgcttccggt gttttgcctg cgacacgaat gttacccacg

5221 aacggcacgg taaccgtacc gttttgatta accatttgtg ccggtaattg cgttaaatgc

5281 ccgctacctt gtccctcaga actaaaagta ccgccaaaca gcaccgccgg cggagcttcc

5341 caaattgata tttcaagtac atcacccaca ttgaccgcac cggcatagcc cgcgctgcct

5401 actgtgccta aaaatccgga aaattgttgg ctttgctgag tttgatacaa ctgttgaact

5461 aaaccgttat ctaattccac taaattgact tccggtaacg gtttatccga atttcgggag

5521 ttagcttcta aaaccgcagt atggctaggg cctgaggtcg gaagacttga gcaggcagcc

5581 aaactagcaa ccagccccaa agaaaggagt aatctaagtt tgatgagttt catctaattt

5641 ctcttcaata tattaaggaa taacaactat ataggtatgt cttaaaatct atataaagat

5701 tgattttaat aagttaccta atcaagagaa attaaatata aaaaatttac aaaaaagcaa

5761 taatgcgtat aaaaaaacat catttgcaaa gaaagtaaat agagaggaga gttcaataga

5821 taagcattat aatccaagat ttatataaaa tataattgat aatataatga acaaaatgaa

5881 tagaaaattt tctaagttac taaaaaatcc acatattttt tttagggatt ttctaaataa

5941 aaagtaccct ataaaaaata cggaacttcc cttctcagaa tctgaagaag ctaacttaat

6001 agaagcaaac caaaaattag ataagattat ccaaaagaat acgttgcaac aagctaatat

6061 tgatgtggta tttacttggg tagatggttc tgatccttca tggcaagcta aatattccca

6121 atatgcacca aattatcaag cgaaatccgc tctatatgca acggatatcg cccgatttga

6181 agatcataat gaattatatt attcagtaca tgctgtactt aaatatatgc cttgggttag

6241 gcatatattt attataacag ataatcaaaa gccaaagtgg ttggatgaga cgagacaaga

6301 aaaaattaca ctaatcgatc atcaagatat tatagataaa gaatatcttc caacgtttaa

6361 ttcccatgtt attgaagcat ttttacataa aattcctaat ttaagcgaga attttatcta

6421 ttttaatgat gatgttttta ttgctcgaga attacaagct gaacactttt ttcaagcaaa

6481 tggaatagcc tctatttttg tgtcggaaaa aagtctctct aaaatgcgtg ataaaggtat

6541 tataacgccg actctttctg cttcggaata tagtattcgc ttattaaaca aatattacga

6601 tacaaatatt gactcacctc tcgtgcacac ttatatccca ttgaaaaaaa gtatgtatga

6661 attggcatgg ctgcgttatg agaaagcaat tcttggattt ttacccaata aattaagaac

6721 aaataacgat ttaaattttg caaactttct tattccttgg ttaatgtatt tcgaagggaa

6781 agcaatgcct aaaatagata tttgttatta ttttaatatt agatctccga atgcaatttc

6841 actatacaaa aaactactac ttaaacaaca gatgggagaa gaacctaact cattttgtgc

6901 aaacgatttt aacagtaatt attctattga aaattataga aataatctta tttccacttt

6961 aaataactat tataaatttt gagaacattt aacatgaata aaattagtaa aagaaaattt

7021 agaaaattaa aaaacactcc agggttattt tttaaggatt tttatacaaa caggttatta

7081 caattaagaa acttcatata tttaaatagt cgaaagaaaa aactaaattc taaaaaattt

7141 actattatca gtgctgttta taatgtgtca gaatatttgg atgattactt agaatcatta

7201 gtaaatcaaa gactagattt tgaaacaagt atagatgtta tcttagtaaa tgatggttct

7261 ccagatgact ctgaaataat tataaaaaaa tggattaaaa aatatccaaa taacattcac

7321 tatattaaga aaaaaatgga ggacaatctt ctgcaaggaa tctaggatta aagtttgtga

7381 aaactgaatg ggtaacattt attgatccag atgacttcct agacttaaat tatttttatt

7441 tattaaatga caccttagaa aaatatgatc atatcggggc atttgttact aaatttaaac

7501 tatttaaaga aaaattcgga acatatcatg atggattcca aaccgatttt tgctttacaa

7561 aaccaataag agtgttaaaa gcaaatgata tggaagattg tgtacaattt tcatccagct

7621 catctgttta tagaacagac gttattcaca aaaataaaat cttatttgat gaaaaattaa

7681 ctgcttcttt tgaagataca aagtttttct atgattattt atataatatt aaagaatcaa

7741 acattctgta tataaaagat gcaatatata attacagatt aagatcaaat gagtcatctt

7801 catccaatag ccaatggaca aaaaaagcta aataccaaga gtttttccaa ttcggtctac

7861 tatctgtaat taagaaatat aatgaaaatg gtactatacc tacatttatt caaagattag

7921 ttttattttc tataattcca tatttacaag ttgcgatgat aaataaaaaa agaattgagg

7981 atgtattaaa tgaatcagaa ataaacaatc tattacaatc gattaaagaa tgtttatctt

8041 atgttgaaac taatacttta gaaaaatttt ataactctcc gggaaattat ttctggatca

8101 acgctataaa caattacttt tataataaac ttcctattga taagagagtt tatatcaata

8161 aagtaaatct tgatgaaaac aaagtctact ttagattcta tggaataaaa ggtaaaacaa

8221 aattttcttt aaaggttaat aataaacact taaaaacaag ctcagaaaga gttatagaat

8281 ataagttatt ttcagacaat ttaattaatg aatataatat ttgttatcat atagaaccaa

8341 ataagaaaat agaattctta ttagatggag agaaagcaaa aatatataca gactttaaaa

8401 tattatctga caaagatact gacttttata aaggatatat aagtaagaat aattctctta

8461 agaatatagc attatttatt gattctggtt ataaagctga tgataatgct gagcatttat

8521 acgaaaaatt attaaaaaat aaaaacttag ataattttat tgatgatcac tactatttgt

8581 tagataaaga aagtgaacat tggaataggt taatattgaa aggatttaat ctagtagata

8641 taaaatctat gaaaggagta tggttaatga agaatgcaaa atacatattt tgttcatatt

8701 taccagggca tctgaatgaa tgggcaacac atcatagctt taagttccaa aaattcatat

8761 ttttacaaca tggtataatt acatcaaatt tatcaaaacc atttaatgca tcgtattcac

8821 aaatatataa gatggtaata tcaagtaaat ttgagaaaag tgagatattg gatgataaat

8881 ttaattatat ttttcatagt aatgatctaa ttttatctac aatcccaaga ttagataaat

8941 tagttaacca taagcgaaat cagagtaata aggtgaagaa gattcttgtt tgcccaacat

9001 ggagaacatc attaggtaat ataaacttta acaaaaagga tgccatatca tcatttaaag

9061 aaacaagtta tataaagaat tggttaggct tattatactc agacaaattg agaaactatc

9121 tagaagaagg gaaaatagaa atttctttcc taccacatca gaattttcat caattacttg

9181 aggaaaatag tttaaatgag aaactattct ttgatataaa tgaaaatata agaatactta

9241 acccgaagaa atcttcttat caagaattat ttattgatca tgatatatta attacagatt

9301 tctcatcatt acattttgac tttgcaacct tacagaaaga tattttatat tttcaattcg

9361 ataaagatga attctatgga atttcacatg cttaccaaaa aggattattt aattttgaaa

9421 aagacggatt tggtcaagta acatacactt tagaggaact attagatcaa ttagttatac

9481 taattaattc tcaaaatgaa aaggtagtta atgggtataa aaaaagaata tcgaatgtat

9541 ttttgcctag cttaggtgat tcttgtaact atattcttaa gaatgtattt actaatccaa

9601 aaagaaatat aaactaaaac attaagtagt caaagttaat agcctataaa gactttatca

9661 ttttgactac tgtttgtatc tgacattata ctaaataatt ttaaatacct tatacaaatc

9721 tttctgttat aagtaatttt ataacatact ataaaatata ctgatactaa taactttaac

9781 caagcggtca aatttttctt aaaacttgca aagtttagga agaataagac cgcttatttt

9841 attcatattc ttatataaaa ttagagttta ttatgagcgt agccacatat aattgataaa

9901 gatatgcggt atagcaatat taatacaacc ctatcaattt catattatct taatcaaatt

9961 tatttttcgt tttttaaata ttaaactatt atttacttct gcaaatctcc cctatcccct

10021 ctttactaaa gagggggatt tcctttagat attaacaagc tatgattttt cgaatagctt

10081 tggtctctca atagattcag gtaaatggtc gatatataag gaaaataaaa acattcgtta

10141 aagacgagcg ccatcttaga gaaatttgga gccacagcat ggctcactca gccgtaggct

10201 gatcgtaacc acgtacggct tgccgtgcgt ggctattggg tgattattta aaaacaggcg

10261 aaacggcttt tttacttttt cattaccgca atttgtttta gaatcaacgg acgatttact

10321 ctcaaggatt tatgatgaaa aaattatttt tagcggcgtt aatcgcatcg ttcgggttgg

10381 ctgcctgcgg tgtaaaaggc ccgctttatt ttctcgagca gcaaccggct caacaacaaa

10441 caaaataatt gctaaccacg gtaattcaaa atctacgtat aacaagcggt cgaatttatt

10501 cgattttttg taaatgcgag tgtagtgagt ttgccgtggt tttatttttt caaacacaac

10561 ggacaaatta atgaatcatt tcaattataa aaaccaacaa ctttttgcgg aagacgtttc

10621 tgtctccgac atcattaatc aatacggcac accggcttat atctattctc gcgctacgct

10681 tgagcgtcat tggcacgctt ttgataaagc attcggcgca cacccgcact tgatttgttt

10741 tgcggtgaaa tctaactcca atatcgcgct attaaatgta atggcacgcc tcggttcggg

10801 ttttgatatt gtgtcgcaag gtgagcttga acgtgtactt gctgcaggcg gtaagccgag

10861 caaagtggta ttttccggtg tggcaaagtc acatagtgaa attcaacgtg cattggaagt

10921 cggcattcgt tgttttaata tcgaatccat cgccgagtta caccgcatta atgaagtcgc

10981 cggtcaatta ggtaaaatcg caccgatttc attgcgtgta aatccggatg tggatgcgca

11041 cactcaccct tatatttcca ccggtttaaa agaaaataaa ttcggggtaa gcgtaacgaa

11101 cgagggaaac aagtactggg agaagcgtta cgagaaaaaa cacaggcgga aaacctactg

11161 cgtgaaagtg cgctaaattg gacgattctc cgcccgtgcg ggctgaatac aacagaaggc

11221 gaaaccttcc gtttaattga aaatgcggct gaactgcccg gcagttatat gagccgtaaa

11281 gcattagcca atgcggtctt gtccgtactt aacagtgaaa acacaaacca taaaatcttc

11341 tcagtctgtg cctaacttca caatcccttt cactttggca caagccatcc gcttgtgcct

11401 atttcaaaat ttgttttaac agttccaata aaaccggtaa ttccgttact ccctctcgat

11461 cgacaaaatg cccgcccgtt gccaagcgaa tataatccgc ttgtaagtat tgcgctaatc

11521 gatcgctgaa cgaatgggga acgacaacat catttaatgc agatatgacg taagactttt

11581 gcggtaaaca agcggtctga tttgcataaa aatctgcaaa gctatctaat tccggcaaag

11641 ttggtaattt ctcataaaag ccggaaacaa aaattgccgt ttttactttt tgctgcgtta

11701 ccgccagata attcagtaac gcaatgcagc ccaaactatg tccgatgagt aaggtatttt

11761 catctaattg aagtgtattt tggtgatgtt ccagccatgc ttgcggattc ggctgatcgg

11821 aattcggcat cgctaaacat tcacattccc atcctaattt ttccaattcg tttttaagcc

11881 acggaaacca atttcttgtc gggttcgccg tataaccgtg cgttacatat acttttttca

11941 t

//

LOCUS **MG868947.1** 14876 bp DNA linear BCT 16-MAR-2021

DEFINITION Actinobacillus pleuropneumoniae strain N-273 capsular polysaccharide gene locus, complete sequence.

ACCESSION MG868947

VERSION MG868947.1

KEYWORDS .

SOURCE Actinobacillus pleuropneumoniae

ORGANISM Actinobacillus pleuropneumoniae

Bacteria; Proteobacteria; Gammaproteobacteria; Pasteurellales;

Pasteurellaceae; Actinobacillus.

REFERENCE 1 (bases 1 to 14876)

AUTHORS Bosse,J.T., Li,Y., Fernandez Crespo,R., Lacouture,S.,

Gottschalk,M., Sarkozi,R., Fodor,L., Casas Amoribieta,M., Angen,O.,

Nedbalcova,K., Holden,M.T., Maskell,D.J., Tucker,A.W., Wren,B.W.,

Rycroft,A.N. and Langford,P.R.

CONSRTM BRaDP1T consortium

TITLE Comparative sequence analysis of the capsular polysaccharide loci

of Actinobacillus pleuropneumoniae serovars 1-18, and development

of two multiplex PCRs for comprehensive capsule typing

JOURNAL Vet. Microbiol. 220, 83-89 (2018)

PUBMED 29885806

REFERENCE 2 (bases 1 to 14876)

AUTHORS Bosse,J.T., Li,Y., Fernandez Crespo,R., Lacouture,S.,

Gottschalk,M., Sarkozi,R., Fodor,L., Angen,O., Nedbalcova,K.,

Holden,M.T.G., Maskell,D.J., Tucker,A.W., Wren,B.W., Rycroft,A.N.

and Langford,P.R.

TITLE Direct Submission

JOURNAL Submitted (26-JAN-2018) Medicine, Imperial College London, Norfolk

Place, London W2 1PG, UK

FEATURES Location/Qualifiers

source 1..14876

/organism="Actinobacillus pleuropneumoniae"

/mol_type="genomic DNA"

/strain="N-273"

/serovar="13"

/note="K locus: KL13"

/isolation_source="lung"

/host="pig"

/db_xref="taxon:715"

/country="Hungary"

CDS 1..1458

/gene="modF"

/codon_start=1

/transl_table=11

/product="putative molybdate ABC transporter ATP-binding

protein ModF"

/protein_id="AVY03701.1"

/translation="MPNINIHNALFSLAQHNKLSIESLEINTHDFWVIVGGNGSGKTA

FAQALHNSLSLYSGEYQNSFQHIALLSFEQQQKIIEQIFKHRNNDMVSPDDFGLTARQ

IILNGSERTQLCEEYAAKLRIQPLLDRPFIQLSTGESRKVLFCQMLVSEPDLLILDEP

FEGLDQASVTYWQEVMAQLGKQMAVVLISNRFNDIPDCATHIALLDNLQLILQGERQE

IEQQAVYSQLKFAEQNVNAPLPESATPLIQLPPNTNPFELKNVMIRYGEKTIIDDLTW

TVAPKQHWWIKGPNGAGKSTLLSIIAGDHPQSYANYVHLFGRQRGSGETIWDIKKNIG

YVSSQLHMDYRVNCSALDVILSGFFDSIGVYQQVPGALQLKAMEWLERLHLANLAKKP

FRSLSWGQQRLLLITRAMVKHPPILILDEPLQGLDGVNRKLVKQFIEQLVTNSQTQLL

FVSHQDADAPNCITHLFEFVPQTNGGYRYVQTALN"

CDS complement(1655..2305)

/gene="cpxA"

/codon_start=1

/transl_table=11

/product="capsular polysaccharide export protein CpxA"

/protein_id="AVY03702.1"

/translation="MISVKNVSKDYYTRSGKKTVLQDINFELKKGEKIGILGRNGAGK

STLIRLLSGVEPPTSGTIERNMSISWPLAFSGAFQGSLTGMDNLRFICRIYNADIEYV

KAFTEEFSELGDYLYEPVKKYSSGMKARLAFALSLSVEFDCYLIDEVIAVGDSRFAAK

CKHELFEKRKDRSIILVSHSPSAMKSYCDNAMVLDKGIMYKFENMDEAYKFYNSTL"

CDS complement(2302..3099)

/gene="cpxB"

/codon_start=1

/transl_table=11

/product="capsular polysaccharide export protein CpxB"

/protein_id="AVY03703.1"

/translation="MQYGDQTTFRQSLAIQGRVIGALLIREIITRYGRKNLGFLWLFV

EPLLLTLFIVLMWKFIRADRVSDLNIIAFVITGYPMAMMWRNASNRTIGAISGNLSLL

YHRNVRVLDTLLARVILEVAGATIAQIIIMALVILLGWIEMPKDTFYMVMAWVLMAFF

ALGLGLIICSIAQKFEAFGKIWGTLSFVLLPLSGAFFFVHALPSQAQQYATLIPMIHG

TEMFRHGYFGDSVITYESISYLVICDVAMLLFGLIMVKNFSKGIEPQ"

CDS complement(3099..4256)

/gene="cpxC"

/codon_start=1

/transl_table=11

/product="capsular polysaccharide export inner membrane

protein CpxC"

/protein_id="AVY03704.1"

/translation="METPIATSPAEKLQKPVKQKKSRFKKLNPLFWITVAIPTVLSAF

YFGSVASDIYISESSFVVRSPKNQTALTGVGALLQGSGFSRSQDDTYTVQEYMRSRTA

LEQLMQGLPVREYYENQGDIIARFNGFGLNNSKEAFYKYFRDRLSVDFDSVSGIASLR

IRAFNAEEGQQINQKLLAEGETLINRLNERARKDTISFAEQAVTEAENNVNETANALS

KYRIKNKIFDLPAQSGVQLSLISSLKSELIRVETQLAQLQSITPDNPQVDALLMRQKS

LRKEIDEQSKQLSSNSNSSIAIQTADYQRLVLANELAQQQLTAALTSLQNTKNEADRQ

QLYLEVISQPSKPDWAEEPYRLYNILATFFIGLMLYGVLSLLIASVREHKN"

CDS complement(4282..5469)

/gene="cpxD"

/codon_start=1

/transl_table=11

/product="capsular polysaccharide export protein CpxD"

/protein_id="AVY03705.1"

/translation="MEIKKYNSIIGLALTTLFLSACSSLPTSGPSHSAILEANSQSSD

KPLPEVNVVELDNGLVQQLYQTQQSQQFSGFLGTVGSAGYAGAVNVGDVLEISIWEAP

PAVLFGGTFSSEGQGSGHLTQLPAQMVNQNGTVTVPFVGNIRVAGKTPETIQSQIVGA

LQRKANQPQALVKIANNNSADVTVIRQGNSIRMPLTANNERVLDAVAAVGGTTENIED

VMVKLTRGSEVKTLAFETLISDPAQNIMLRAGDVVSLLNTPYSFTGLGAVGNNQQMKF

SSKGITLAEAIGKMGGLIDTRSDPRGVFVFRHVPFAQLSLEQQAQWQAKGYAIGMDVP

TVYRVNLLEPQSMFLLQRFPMQDKDIVYYVSNAPLSEFQKFLRMIFSITSPVTSTTNAV

RAY"

CDS 5648..6790

/gene="cps13A"

/codon_start=1

/transl_table=11

/product="CDP-glycerol:poly(glycerophosphate)

glycerophosphotransferase"

/protein_id="AVY03706.1"

/translation="MLMKIAFIAWNSFQVLHFKPLLQALPCALLIIEKRRRSVPICKD

ILRDINNNIAYIRHTDIYAKIDGNFDVLVAQTTFEQLYLFHRTKIALLQYGYAKEPYN

YGTWRAFADLNLVYGNYAYERISYFSPTKITGCPRYDLWYQPLFHQKAKENYARVLDT

SKKTIVYAPSWGELSSFKLYIEEITKLSLFYNVLVKLHHNTLLLANKHQNYEKLYPNL

HFFYEGEDLLSLISVADIVISDFSGAIFDAIFCKKPVILFSIPLVDQPKLDKFSLEIA

HRSALGYEVSSPERVAITVEKALTEQKLADKMLYQQLFMGNENATQQVIDALQQLVDG

KYSLSQQQLYVRQTEKLLNIEKIKQQKNKKQSFNKIRQISKKLIKK"

CDS 6844..7272

/gene="cps13B"

/codon_start=1

/transl_table=11

/product="glycerol-3-phosphate cytidylyltransferase"

/protein_id="AVY03707.1"

/translation="MKKVLTYGTFDLLHHGHIRLLERARSLGDHLTVAISTDQFNLGK

GKVCAYTYEERAHILKAIRYVDEVIPETNWEQKVEDVKNHEIDVFVMGDDWEGKFDFL

ADYCEVVYLPRTPDISTTQVKKMLAKKDLAAGQKQIHEKE"

CDS 7280..8425

/gene="cps13C"

/codon_start=1

/transl_table=11

/product="capsular polysaccharide biosynthesis protein

Cps13C"

/protein_id="AVY03708.1"

/translation="MFQILKKKLPTLQRVLRESDSQCSLLSYWYGLNLLSALERADHV

HVRKLADKMLNKGINIGHYFLAQSYFLCGEYTLAEQAVKKIPNFTKIPEVVFLYSDIL

IKCQRREEAWLLLEQCALLNKRKKVWIHLTNLVNTEADYRHLEQHIDKVRTTTPYLKS

DLLIHQRTNAALRAGLTETALALTELNPLPKQAKVKKKTTAYSDKLAAIALADLKKVL

DHKKIPFFLISGTLLGCIREGKLLGHDKDIDVGVWDEYSYEELANCLSTSGYFYVVPT

RTKHLVMLRHVNGIAIDVFIHYREPNDYWHAGVKIKWHNSPFNLVYTNFLGQQYLIPE

NYDLYLAENYGDWRTPKTKFDSAFDTPNMEVFDETEMIVYKYRKALM"

CDS 8436..12209

/gene="cps13D"

/codon_start=1

/transl_table=11

/product="putative glycosyltransferase"

/protein_id="AVY03709.1"

/translation="MKKFKKLLEISRYPLSYWRGMKFYRNRDWDRASIYFKKAVNVMP

MHPQSNFKLGMCYFKQRKWELAYQFISVAVDLLPSKEEWKVQLYQSQLKLNNINGIKL

TTSASLIEEELIRKRLETEKPTGKLYARLAELLHKQGKSWQEVDALQKAVELSPKNAQ

LYRRLGESLETMKRYEEAAFAYKTAIKLKGNKADYELFYQYGFCLEKIDAKQEDIIQA

YTLAIEKDDIDDSKKFGIGAIHERKGRWSEATDAYLTSFSNNPSNGELCYRVGFAYQR

CYDWDNAERYYLLALKLDTSNPNWYYQVGFVREKKGAFLEATEYYKYATNKKYTPYWM

YRLGLCLTKANKHKEATLAFLKTKKSFKEEHLEESELSIFLDDNKIDKLQEKLSLDYS

NLELWHKLSNIYFSRGDLVNAEKHFYQILLRTNEYNSDLYYKYGLILAKLGNFKRAAR

FLRNCRQIQTLHGLPDRKFNNDEGFRQAAIYSEYYDVLNVNKKIILFESFSGVAMSCN

PLAIFLEMKKDSRFDNFLFVWVINDITTVSDEYKKHQNVVFVQKDSDLYLRYLCHAYY

LVNNATFPPYFTRKKEQKYLNTWHGTPWKTLGKDIKNSFMELKNSQRNFLQSTHMLSP

NPHTTWVLADRYDIKEIYLGKFLEAGYPRIDLTLNISDDRKSELRRTLNIDPTKKVVL

YAPTWRGTLGSPEVEADKLISEIKALKDLGINLLFRGHYFVQKNAYESGIEQYIVPEF

INTNELLSIVDILITDYSSIGFDYMATGRPIVYYIDDYEEYKADRGLYFDYDKLPGEM

ATNINELKKAILNEVSSPKAHSLYPQAQKEFTPYENGQVSSRVINWFIHGLSDENEIN

ISSQEKKSILIFGGEFLPNGITTSIINLLNNIDYKKYTVSLLIDPNAISKEEKRLAQF

ARVSPKVNIIPRVGRMNRSIEDDWVEAKANQYKFVPKNFRAYFERAYNKEFRRIVGYS

KFDALVEFTGYSRFWAYLLGSAKIKNVVRTIYQHNDKYGEWTLRFPYLENTFSIYYMY

DHLMSVSKPTMDLNIKNLCERFSLDINKFDYCDNVQDPESTIIKSKEELSTEDEKYFE

NCKGKIFINLARLSPEKDQAKLIRSFRILVNKYPNSRLLILGDGPLYNDLSNLIKELN

LESNVFLVGIRFNPFPFLKRADCFVLSSNHEGQPMTLFEAMILEKPIIATDIVGSRSA

LEGRPGHLVPNSEEGLYQGLSDFIEGKLHFSHFDYNSYQNSALNMFYSKILSK"

CDS 12306..13598

/gene="cps13E"

/codon_start=1

/transl_table=11

/product="glycosyltransferase family 2 protein Cps13E"

/protein_id="AVY03710.1"

/translation="MKKLRVGIIGSCVSRLAFRSDFIPESKPFFDVIQYQFHTSLISV

MAQPIKYDYSKFKGREDEYAKEHLASELDKDGLNNLVASNPDILLIDFYPDVHFGISY

TEHSIITNKCWRYKRIEAFNELDIKGHLDPITSFDEYFKIWKENLSKFIEFMTSYLPN

TKIIIIGARFAELQNIDGVVSQINSEFDLAKRNKVWNIFDKYAVEAYKLDYLDLTSRY

MATNNHTHGLDPLHFERTYYSDFIIKLFNCVYSCKARNLSSLELLEIDQSQFLYQKGK

NAKKWNLLDSKVLNLWHHNKASAFNISDETITISVKGANKPIYNQLHSLPIEIGGRND

SYFQYKLSFDIFIKDLDELEDDSIFLLRTHKGKFTLWHKDAISSVMLKAKKLELTGGK

WEHVQVAMICKDRFLRVSPYLAQNGSVQWKNIKLERVA"

CDS 13708..14262

/gene="hypothetical"

/codon_start=1

/transl_table=11

/product="hypothetical protein"

/protein_id="AVY03711.1"

/translation="MSKKITLLSLAILIAGCSSSPQPEAFPGEFANADYVLSDKDAQR

WVVASHQAEQCIYPNLTRIQQQAFSKEDSYIHSQYVFFYPLEEIIGEQYVKIIQDDEK

SMGYAQYLFKKFRDNQEFEPLADKQCLVLREKAKNDLAVVKGQYKSGMVEETKSESKN

ADGVATNQNKFFFDIIKWGSMLLL"

CDS complement(14334..14876)

/gene="ydeN"

/codon_start=1

/transl_table=11

/product="putative hydrolase YdeN"

/protein_id="AVY03712.1"

/translation="MRKVYITHGYTANPSRNWFPWLKNELEKLGWECECLAMPNSDQP

NPQAWLEHHRNTLQLDENTLLIGHSLGCIALLNYLAVTQQKVKTAIFVSGFYEQLPHL

PELDEFANFYTNQTACLPQKSYVISALNDVVVPHSFSDRLAQYLQADYIRLATGGHFV

DREGVTELPELLELIKQISN"

ORIGIN

1 atgccaaaca tcaacattca caacgcctta ttttcccttg ctcaacacaa taaactgtcg

61 attgaatcgc tggaaatcaa tactcacgat ttctgggtga ttgtcggcgg taacggctcg

121 ggtaaaactg ctttcgccca agcgctacat aattcacttt cactatattc gggcgaatat

181 caaaacagct tccaacatat cgctttactt tccttcgagc agcaacaaaa aatcatcgag

241 caaatcttta aacaccgtaa caatgatatg gtttcgcctg acgatttcgg tttaaccgcc

301 cgtcaaatta tcttaaacgg tagcgaaaga acgcaattat gcgaggaata tgcggctaaa

361 ttacgtatcc agccgttatt agatcgcccg tttattcagc tctccaccgg cgaaagccgc

421 aaagtgttat tttgccaaat gttagtcagc gaaccggatt tattgatttt agatgagcct

481 tttgaagggt tagaccaagc ctcggtcact tattggcagg aagtgatggc acaactcggt

541 aagcaaatgg cggtggtact gatttccaac cgttttaatg atattcccga ctgtgccaca

601 catattgctt tactggataa cttacaactg attttacaag gcgaacgtca agagattgaa

661 caacaagcgg tctattctca gctaaaattt gcagaacaga atgtgaatgc accgttgccg

721 gagagtgcca caccgctgat tcaactccca ccgaatacta atccgtttga actgaaaaac

781 gtaatgatcc gttacggcga aaaaacgatt attgatgatc taacttggac ggttgcccca

841 aaacaacatt ggtggattaa aggcccgaac ggagcaggaa aatcgacctt actttctatt

901 attgccggcg atcatccgca atcttacgct aattatgtgc atttattcgg tcgtcagcgt

961 ggttcgggcg aaacgatttg ggatataaag aaaaatatcg gctatgtgag cagccaatta

1021 catatggatt atcgggtgaa ttgctctgcg ttagacgtga ttttatccgg tttttttgat

1081 tcaatcggcg tttatcaaca agtaccgggt gccttacagc taaaagcaat ggaatggctg

1141 gaacgcttgc atttagccaa tctggcgaaa aaaccgttcc gttcactttc gtgggggcaa

1201 caacggttat tattgattac tcgtgctatg gtaaaacacc cgccgattct gattttagac

1261 gaaccgctgc aaggtttgga cggtgtaaac cgcaaattgg ttaaacaatt tatcgaacag

1321 cttgtgacta atagtcaaac ccagttgcta tttgtttcgc accaagatgc ggacgcccca

1381 aattgtatca cccatttatt tgaatttgtt ccgcaaacta acggtggtta ccgttatgtg

1441 cagacggcgt taaattaggt ttttgacctt taaaggaaat ccccctcttt agtaaagagg

1501 gattagggga gatttgtcaa tagagagata tgaaattgaa tagaacttca ttttttatat

1561 ttataaaagc gttaattagc atatttcttc gctaattcat tctgtcaaat ctcctcctgc

1621 ccctctttag taaagagggg ggatgtgtgg gactttaaag cgttgaatta tagaacttat

1681 aagcctcgtc catattctca aatttataca taatcccttt atctaatacc attgcattat

1741 cgcaataaga cttcattgct gacggactat gcgaaaccaa aataatcgaa cgatctttgc

1801 gcttttcaaa taattcatgt ttacattttg ccgcaaagcg agagtcacct accgcaatta

1861 cctcatcaat taagtagcaa tcaaactcta ccgaaagcga caaagcaaag gcaagtcggg

1921 ctttcatgcc ggaggaatat ttcttaaccg gctcatataa ataatcaccc aattcggaaa

1981 attcttcggt aaaggcttta acgtattcaa tatccgcatt atatatacgg caaataaagc

2041 gtaaattatc cataccggtt aaactgcctt ggaacgcccc gctgaaagcg agcggccaag

2101 atatcgacat attacgttcg atagtacctg atgttggcgg ctcaacacca cttaacaaac

2161 ggattagcgt tgatttccct gcaccgttac gccctaaaat accgattttc tcgccttttt

2221 tcagctcaaa attaatatct tgcaatacgg tttttttacc gcttcgagta tagtaatctt

2281 tactcacatt ttttacacta atcattgcgg ttcgattcct ttactgaagt tttttaccat

2341 aatgagccca aaaagtaaca tggctacatc acatattacg agatagctta tactttcata

2401 tgtgataaca ctgtcgccaa aataaccgtg acgaaacatt tccgtgccgt gaatcatcgg

2461 tattaaggtt gcatattgtt gagcttggct tggtagcgca tgcacaaaga aaaatgcgcc

2521 tgaaagaggt aaaagaacaa agcttaatgt tccccagatt ttgccaaatg cttcaaattt

2581 ttgtgcaata gaacaaataa tcaagcctaa tcctaatgca aaaaatgcca ttaataccca

2641 cgccataacc atataaaacg tatctttcgg catttctatc cagcctaata aaatgactaa

2701 tgccataata atgatttggg caatcgttgc acccgctacc tcaagtatga cacgagccag

2761 taaggtatct aatacgcgaa cattacgatg ataaagaaga ctcaagttac cggaaattgc

2821 accgatagtg cggtttgatg cattacgcca catcattgcc attggataac cggtaatcac

2881 aaaagcaata atatttaaat cggaaacgcg atccgctcgg ataaatttcc acatcaaaac

2941 gataaataaa gtgagtaata gcggctcaac aaacagccat aaaaaaccca aattttttcg

3001 tccgtaacgc gtaataattt cccgtatgag taatgcaccg attactctcc cttgaatggc

3061 gagagattgg cggaaagttg tttgatcacc gtattgcatt agtttttgtg ctctcttacg

3121 cttgcaatta ataaacttaa tacaccataa agcatcagac cgataaagaa tgtcgctaaa

3181 atattatata agcgataagg ctcttccgcc cagtccggtt tgcttggctg actgattact

3241 tctaaataaa gttgctggcg atccgcttca tttttcgtat tttgtaatga ggttaatgct

3301 gcggtcaatt gttgctgtgc cagctcgttt gcaagtacta agcgttggta atcggcagtt

3361 tgaatagcaa tagagctatt actgttactg gaaagctgtt ttgattgctc gtcaatttcc

3421 ttacgtaaac ttttttggcg cataagcaat gcatcaactt gcgggttgtc cggtgtaata

3481 gattgcaatt gagccaattg tgtttccaca cgaatcaact cgctttttaa gcttgaaatt

3541 aatgaaagtt gtacgcccga ttgtgccggt aaatcaaaaa ttttattttt gatacggtat

3601 ttacttaaag catttgccgt ttcgtttaca ttattttccg cttctgtaac cgcttgttcc

3661 gcaaatgaaa tggtatcttt tcttgcacgt tcgtttaaac ggttaataag cgtttcacct

3721 tcggcaagga gtttttgatt aatttgttgc ccctcttccg cgttaaatgc tcgaatacgt

3781 aagctggcga taccggaaac agagtcaaag tccacactta agcgatctcg gaaatattta

3841 taaaacgctt ctttactgtt atttaatccg aatccattaa agcgagcgat aatatcgccc

3901 tgattttcat aatattcgcg aacaggtaat ccttgcatta gttgctcaag agcggtacga

3961 gaacgcatat attcctgtac ggtataagta tcgtcttgcg aacgagaaaa acctgaacct

4021 tgcaataagg cgcctacgcc tgtcagagcg gtctgatttt taggcgatct aacgacaaaa

4081 cttgattccg aaatataaat atcagaagcg accgaaccga aataaaacgc tgatagcacg

4141 gtagggattg cgacagttat ccaaaataat ggattgagtt ttttaaagcg gcttttttttc

4201 tgtttaaccg gtttctgtaa tttttctgct ggactggtag caataggtgt ttccatcttt

4261 tgtccttata cattcaatat attaataggc acgaacggca ttggtcgtac tggtaaccgg

4321 cgaagtaatt gagaaaatca ttctcaagaa tttttggaat tcagacaacg gcgcgtttga

4381 aacatacaca atatctttat cttgcattgg gaaacgctgt aataaaaaca tggattgcgg

4441 ctcaagtaag ttcacacgat aaaccgttgg tacatccatt cctatagcgt agcctttagc

4501 ttgccattgt gcttgttgtt ccaaactcaa ttgtgcaaaa ggcacgtgac ggaatacgaa

4561 aacccctctc ggatccgaac gagtatcaat taaaccgccc atcttaccga tagcttcggc

4621 aagcgtaatt cctttacttg agaatttcat ttgctggttg ttacccacag cgcctaaacc

4681 ggtaaaacta taaggcgtgt ttagcaacga aacgacatcg ccggcacgta acataatatt

4741 ttgcgccgga tcggaaatta acgtttcgaa tgcgagtgtt tttacttcag aaccacgggt

4801 tagcttgacc atcacatctt caatattttc cgttgtaccg cctactgccg caaccgcatc

4861 taatacgcgt tcattattag cggttaatgg catacgaata ctattacctt gacgaataac

4921 cgtaacatcc gcagagttat tattcgcaat tttgactaat gcttgcggct gattcgcttt

4981 acgctgcaat gccccaacaa tttgagactg aatcgtttca ggtgttttac ctgcgacacg

5041 aatatttccc acaaacggca cggttaccgt cccgttttga ttaaccattt gtgccggtaa

5101 ttgcgttaaa tgcccgctac cttgtccttc ggaactaaaa gtaccgccaa acaacactgc

5161 cggcggcgct tcccaaattg aaatttcaag tacatcaccc acattgactg caccggcata

5221 gcccgcgctg cctactgtgc ctaaaaatcc ggaaaattgt tggctttgct gagtttgata

5281 caactgttga actaaaccgt tatccagttc caccacattt acttccggta agggtttatc

5341 cgaactttgt gaattagcct ctaagatcgc actatggcta gggcctgaag ttgggaggct

5401 tgagcaagca gaaaggaaca atgttgttaa agctaaccct atgattgaat tatatttttt

5461 aatttccatc ttgtaaaaag gctctattga aaaagtgtgt aaaatgaata taaactatac

5521 ataattatag atacaatact ataattatat attataatct aatatctcgt tttttaaaac

5581 gataacctaa aatattactt ttctatagaa gaataggcat tattttaata aatataatta

5641 ggtatgaatg ttgatgaaaa tagcatttat cgcttggaat agttttcagg tgttacattt

5701 caagccctta ttacaagctt taccgtgtgc attattaatt attgaaaaac ggagacgtag

5761 tgtaccaatc tgtaaggata ttttgcgaga tataaacaat aatatcgctt atatccgcca

5821 tacggatata tatgcaaaaa ttgatgggaa ttttgatgtt ctagttgctc aaactacttt

5881 cgagcaactt tatttgtttc accgcaccaa aattgcattg cttcaatacg gatatgctaa

5941 ggaaccgtat aactacggca cttggagagc atttgcagat ctaaatttgg tttatgggaa

6001 ttatgcctat gaacgtattt cctatttctc tccaactaaa ataaccggtt gtccacgata

6061 cgatttatgg tatcagcctt tatttcatca aaaagcgaaa gaaaattatg cgagagtatt

6121 agatacgagt aagaaaacga ttgtatatgc accaagttgg ggagaattat ccagctttaa

6181 attatatata gaagaaatta cgaaattatc tttattttac aacgtgttag taaaattaca

6241 ccataacacg cttttattag caaacaagca tcagaattat gaaaaattgt atccgaattt

6301 acattttttc tatgaaggtg aagatcttct ctcacttatt tcggtagccg acattgttat

6361 ttccgatttt agcggtgcga tctttgacgc aattttctgt aaaaaaccag taatattatt

6421 ttctatccca ttagtagatc aacccaaatt agataaattt agtttggaga tagcccatcg

6481 ttcggcgttg ggatatgagg tttcttcacc tgaacgagta gctataacgg tggagaaagc

6541 actaacagag caaaaattag cagataaaat gctatatcaa cagcttttta tgggtaatga

6601 aaatgcaaca caacaagtga tagatgctct acaacagctc gttgatggaa aatattcctt

6661 atcgcaacaa cagttatatg tgcggcaaac agagaagtta ttaaatattg aaaaaataaa

6721 gcaacagaaa aataaaaaac aatcttttaa taagataagg cagatttcta aaaagttaat

6781 taaaaaataa atttagacat tttatttttt atatcttttt acttaataat acggagttta

6841 ggaatgaaaa aagtattaac ctatggaacc tttgatttat tacaccatgg gcatattcgt

6901 ttattagaaa gagcaagatc attaggcgat caccttactg ttgctatttc taccgatcaa

6961 tttaacttag gaaaaggcaa agtatgcgct tatacttacg aagagagagc gcatatttta

7021 aaagcaatcc gttatgtgga tgaagtaatt cctgaaacaa actgggagca aaaagttgag

7081 gatgtaaaaa atcacgaaat tgacgtattt gtaatgggtg atgactggga aggcaaattt

7141 gacttcttag cagattactg cgaagtggtt tatttaccga gaacccctga tatttcaacg

7201 actcaggtaa aaaaaatgct tgcgaaaaaa gatctcgcag ccggacaaaa acaaatacac

7261 gaaaaagagt aatttgttga tgttccaaat cttaaaaaag aagttaccaa ctttgcaaag

7321 agttctgaga gaaagtgatt ctcagtgttc tttgcttagt tattggtatg ggcttaatct

7381 actttccgca ctggaaaggg cagatcacgt tcacgtaaga aaacttgccg ataaaatgct

7441 taataaaggt attaatatcg gacactattt tttagcacag agttattttc tatgtggaga

7501 atatacgcta gcggagcaag cggttaaaaa aatcccaaat tttactaaaa tacctgaagt

7561 tgttttttta tattcagata ttcttatcaa atgccagcgt agagaagaag cttggctact

7621 attagaacaa tgcgctttac tcaataaaag aaaaaaagtg tggatacatc taacaaattt

7681 agtaaatact gaggcggatt atcgacactt agaacaacat attgacaaag taagaacaac

7741 tacaccttac ttgaagtctg atttgttaat tcatcaaaga acaaatgcgg cattaagagc

7801 aggtttaaca gaaacggcat tagctctaac agagcttaac cctttgccaa agcaagcaaa

7861 agttaagaaa aaaacaaccg cttatagtga taaattagcg gcaattgcgc tagcggatct

7921 aaagaaagta ttagatcaca aaaaaatacc tttctttctg attagcggca cgttgctagg

7981 ttgtattcga gaaggaaaat tattagggca tgataaagat attgatgtcg gcgtttggga

8041 tgagtactcc tacgaagaat tagcaaactg tttatctaca tcgggatact tttacgtagt

8101 accaactcga acaaaacatt tagtcatgtt aagacatgtt aatggtattg caattgatgt

8161 gtttattcat tatcgcgaac ctaatgacta ttggcacgct ggtgtcaaaa taaaatggca

8221 taattcacca tttaatttgg tatatacaaa tttccttggg caacaatatt taatacctga

8281 aaattatgat ttatacctag cagaaaatta tggtgactgg cgtacgccaa aacaaaatt

8341 cgacagtgct tttgatactc ctaatatgga agtatttgat gaaactgaga tgattgtata

8401 taaatataga aaagcattaa tgtagcaggg caattatgaa aaagtttaaa aaactattag

8461 aaatttctcg ttatccttta agttattggc gaggtatgaa gttttatcgg aatagagatt

8521 gggatagggc ttcaatttat tttaaaaaag cagttaatgt tatgccaatg catccacaaa

8581 gcaactttaa attgggtatg tgttatttca aacaacggaa atgggaactt gcatatcagt

8641 ttattagtgt tgccgtagat ctcttaccga gtaaagaaga atggaaggtg cagttatatc

8701 agtctcaact caaattaaat aatattaatg gtattaaatt aacgacatct gcatcattaa

8761 ttgaagaaga gttaattaga aaacgtttag aaacagagaa accaaccggt aagttatatg

8821 ctcgtttagc tgaattactt cataaacaag gaaagtcttg gcaagaagtt gatgctttac

8881 aaaaagcggt ggagctttct cctaaaaatg ctcagttata tcgtcgttta ggggaatctc

8941 tggaaacgat gaaacgttat gaggaggccg cttttgcata taaaactgca ataaaattga

9001 aaggaaataa agctgattat gaattatttt atcagtatgg gttctgttta gaaaagatag

9061 atgcaaaaca agaagatatt atccaagcct atactttggc aatagaaaaa gatgatatag

9121 atgattcaaa aaaattcggt attggcgcta ttcatgaacg gaaagggcgt tggtcagaag

9181 ctaccgatgc atatttaact tcgttttcta ataatccctc gaatggagaa ttatgctatc

9241 gagtggggtt tgcttatcaa cgttgttatg attgggataa tgctgagaga tattatttat

9301 tagctttgaa gttagataca tcgaatccta attggtatta tcaagttggg tttgtgagag

9361 agaagaaagg tgctttctta gaagcaacag aatattataa atatgctacg aataaaaaat

9421 atacacctta ttggatgtac cgtttaggtt tatgtttaac aaaagcgaat aagcataaag

9481 aagctacatt ggctttctta aaaactaaaa aatcatttaa agaagaacat ctagaagaaa

9541 gtgaattatc tatatttcta gatgataata aaatagataa attacaggaa aagttatctt

9601 tagattactc taatttagaa ctttggcata agttaagtaa tatttacttt tctcgtggag

9661 atttagtaaa tgcagagaaa catttttacc aaattctttt aagaacaaat gaatataata

9721 gtgacttata ttataaatat ggccttattt tggctaaatt aggtaatttt aaacgggctg

9781 ctcgtttcct tagaaactgt agacaaattc aaacacttca tggtcttcca gataggaagt

9841 ttaataatga tgaaggattt agacaggcgg ctatttatag tgagtattat gatgttttaa

9901 atgtaaataa gaaaattatt ttatttgaaa gtttttctgg tgttgcaatg tcttgcaatc

9961 ctttagctat atttttagaa atgaaaaaag atagccgttt tgataatttc ttatttgtat

10021 gggttattaa tgatatcacg acagtcagtg acgaatataa gaaacatcaa aatgttgtat

10081 ttgttcaaaa ggattcagat ctgtatttaa ggtatttatg tcatgcatat tatttagtta

10141 ataatgcaac attcccacct tattttacaa gaaagaaaga gcagaagtat ctaaatacat

10201 ggcatggtac tccatggaaa actttaggaa aagatattaa aaatagtttc atggagttaa

10261 aaaattccca gagaaacttt ttgcaaagta cacatatgtt aagcccaaat cctcatacga

10321 cttgggttct tgcagaccgt tacgatatta aagaaattta tctaggaaaa ttcttagagg

10381 caggttatcc aagaattgac ctaacattga atatatcaga tgatagaaag tcagagttac

10441 gcagaacttt aaatattgat cctactaaga aggtcgtatt atatgctccg acttggagag

10501 ggactttagg ttctccggaa gttgaggctg acaaattaat ttctgagatt aaagcattaa

10561 aagatttagg tattaattta ctatttaggg gacattactt tgttcagaag aatgcttatg

10621 aaagcggtat tgagcaatat atagttccgg agtttattaa tactaatgaa cttttatcaa

10681 tcgtagatat tttaattaca gattattcta gtattgggtt cgattatatg gctacgggta

10741 gaccgatagt ttattatatc gatgattatg aagagtataa ggcagataga ggtctatatt

10801 tcgattatga taaattgcca ggagaaatgg ctaccaatat caatgaattg aaaaaagcta

10861 ttttaaatga agtaagtagt cctaaagctc attcattata tccacaggct caaaaggaat

10921 ttacgcctta cgagaatggt caagtatcta gtagagtcat taattggttt attcatggtt

10981 tatctgatga aaatgaaatt aatatttcta gtcaagagaa gaaatctata ttaatttttg

11041 gtggtgaatt cttaccaaat ggaattacga cttctatcat taatttatta aataatattg

11101 attataagaa atatacagtt tccctattga ttgacccaaa tgctatttct aaagaagaaa

11161 agcgtttagc tcagtttgct cgtgtttctc caaaagtaaa tattattccg agagttggta

11221 gaatgaatcg gagtattgag gatgattggg tagaggctaa ggcaaatcaa tataaatttg

11281 ttccgaagaa cttccgagct tattttgaaa gagcatataa taaagaattc agacgtatag

11341 ttgggtattc taagtttgat gctcttgttg aatttactgg gtacagtaga ttttgggcat

11401 atttattagg ttctgcaaaa ataaaaaatg tagtaagaac aatttatcag cataatgata

11461 agtatgggga atggacttta cgattccctt atttagagaa tacgtttagt atttactata

11521 tgtatgatca tttaatgtca gtttctaaac caactatgga tctgaatatt aagaatctgt

11581 gcgagagatt tagtttagat ataaataaat ttgattactg cgataatgta caagatccag

11641 aatcaacaat tattaaatct aaagaagaac tttctacgga agatgagaaa tattttgaga

11701 attgtaaagg taaaattttt atcaatttgg caagactttc tccagaaaaa gaccaagcta

11761 aattaattag aagttttaga attctagtga ataaatatcc taattcaaga ttactgattc

11821 ttggagatgg gccattgtat aatgatttaa gtaatcttat caaagaacta aatttagaaa

11881 gtaatgtgtt tcttgttggt attaggttca atccatttcc attcttgaaa cgggctgatt

11941 gtttcgtatt gtcttcaaat catgaggggc aaccaatgac tttatttgaa gcaatgattt

12001 tagaaaaacc aattattgct actgatattg ttggctctag aagtgcttta gaaggacgtc

12061 caggtcattt agttcctaat tcggaagaag gattgtacca agggctatct gattttattg

12121 aagggaaatt acatttctct cattttgatt ataattctta tcaaaatagt gcattaaata

12181 tgttctatag taagattctt tctaaatagt ttaattttaa taaagcccat ttatgggctt

12241 tattattaag aaatatatta atttagtatt aggaaatatt atttattaat ttaggaaaat

12301 aagtcatgaa aaaactaaga gttggtatta ttggaagttg tgtatcgagg ttggcatttc

12361 gttcagattt catacctgaa agtaaaccct tttttgatgt aatccaatac cagtttcata

12421 catcattaat ttctgttatg gctcagccaa tcaaatatga ttattcaaaa tttaaaggta

12481 gagaagatga atatgcaaag gaacacctag caagtgaatt agataaagat gggctaaata

12541 atttagttgc ctctaatccg gatatattgc ttatagattt ttatccagat gttcattttg

12601 gaatatctta tactgaacat tcaattatta caaataagtg ttggagatat aaaagaattg

12661 aagcttttaa tgaattagat attaaaggac atttagatcc aataacttca tttgatgagt

12721 attttaaaat ctggaaagaa aatttatcta aatttattga gtttatgaca tcttatctac

12781 ctaatacaaa aataattatt attggtgcta gatttgcaga attacaaaat atagatggag

12841 ttgtgagtca gatcaattct gaattcgatt tagcgaaaag aaataaagta tggaatatat

12901 ttgacaagta tgcagtagaa gcttataaat tggattattt agaccttaca tcaagatata

12961 tggcaactaa taatcataca cacggcttag atcctttaca ttttgaaaga acatattatt

13021 ctgactttat tattaagtta tttaattgtg tttattcttg taaagctaga aacttaagta

13081 gtttagagct attagaaata gatcaaagtc agttcctata tcaaaagggc aagaatgcta

13141 agaaatggaa tttattagat agcaaggttt tgaatctttg gcatcataat aaagcatctg

13201 catttaatat ttctgatgaa acgattacta tttcggttaa gggagcaaat aaaccaatat

13261 ataatcaatt gcacagttta cctattgaaa taggtggtag aaatgattct tatttccaat

13321 ataagttgtc gtttgatatc tttattaaag atttggatga attggaggat gattcaattt

13381 tcttattaag gactcataaa ggaaaattta ctttatggca taaagatgcg atatcttcag

13441 taatgttaaa agctaaaaaa ctagaattaa ccggtggaaa atgggaacat gttcaagttg

13501 caatgatttg taaggataga ttcttacgag tatctccata tttagcacaa aatggctctg

13561 ttcaatggaa aaatatcaaa ttagagagag tagcatgatt ttttgaaccc taatgtataa

13621 aatttttctt aagatttttt aaaatttata agaagggata tgttatataa taaaaacctt

13681 taaatatatc aagagtaaaa acatattatg tccaaaaaaa tcactttatt gagtcttgcc

13741 atattaatcg caggttgctc ttcttccccg cagccggaag ctttcccggg ggagtttgcg

13801 aatgcggatt atgtgttatc ggataaggat gcgcagcgtt gggtggtggc gagccatcag

13861 gcggagcagt gtatttatcc gaacttgacg cggattcagc agcaagcgtt tagcaaggaa

13921 gattcatata ttcattcgca atacgtattt ttctatccgt tggaagaaat tatcggcgag

13981 cagtatgtga aaattatcca agacgatgaa aaatctatgg gatatgcgca atacttgttt

14041 aagaaattca gagataatca ggaattcgag ccgttagcgg ataagcaatg tcttgtgttg

14101 cgagaaaaag cgaagaacga tttagcggtc gtaaaagggc agtataagag cggaatggtt

14161 gaagaaacga agtccgaatc taaaaatgcg gacggcgtgg cgaccaatca aaataaattc

14221 ttctttgata ttatcaaatg gggttcgatg ctattactgt aatttgcggt tagtgtgatg

14281 ttaaagagta taaaaacgag ctgtcggtaa atcgttatcg gcagctcttt ttattagttt

14341 gatatttgtt tgattaactc caataactcc ggtaattccg ttactccctc tcgatcgaca

14401 aaatgcccgc ccgttgccaa gcgaatataa tccgcttgta agtattgcgc taatcgatcg

14461 ctgaacgaat ggggaacgac aacatcattt aatgcagata tgacgtaaga cttttgcggt

14521 aaacaagcgg tttgatttgt ataaaaattt gcaaactcat ccaattccgg taaatgcggt

14581 aattgttcat aaaaaccgga aacaaaaatc gccgttttta ctttttgttg cgtcaccgca

14641 agataattca gtaacgcaat gcagcccaaa ctatgtccga tgagtaaggt attttcatct

14701 aactgaagtg tatttcggtg atgttcaagc cacgcttgcg gattcggctg atcggaattc

14761 ggcatcgcta aacattcaca ttcccatcct aatttttcca attcgttttt cagccacgga

14821 aaccaatttc ggctcgggtt cgccgtataa ccgtgcgtga tatatacttt tctcat

//

LOCUS **MG868948.1** 18149 bp DNA linear BCT 16-MAR-2021

DEFINITION Actinobacillus pleuropneumoniae strain 3906 capsular polysaccharide
gene locus, complete sequence.

ACCESSION MG868948

VERSION MG868948.1

KEYWORDS .

SOURCE Actinobacillus pleuropneumoniae

ORGANISM Actinobacillus pleuropneumoniae
Bacteria; Proteobacteria; Gammaproteobacteria; Pasteurellales;
Pasteurellaceae; Actinobacillus.

REFERENCE 1 (bases 1 to 18149)

AUTHORS Bosse,J.T., Li,Y., Fernandez Crespo,R., Lacouture,S.,
Gottschalk,M., Sarkozi,R., Fodor,L., Casas Amoribieta,M., Angen,O.,
Nedbalcova,K., Holden,M.T., Maskell,D.J., Tucker,A.W., Wren,B.W.,
Rycroft,A.N. and Langford,P.R.

CONSRTM BRaDP1T consortium

TITLE Comparative sequence analysis of the capsular polysaccharide loci
of Actinobacillus pleuropneumoniae serovars 1-18, and development
of two multiplex PCRs for comprehensive capsule typing
JOURNAL Vet. Microbiol. 220, 83-89 (2018)
PUBMED 29885806
REFERENCE 2 (bases 1 to 18149)
AUTHORS Bosse,J.T., Li,Y., Fernandez Crespo,R., Lacouture,S.,
Gottschalk,M., Sarkozi,R., Fodor,L., Angen,O., Nedbalcova,K.,
Holden,M.T.G., Maskell,D.J., Tucker,A.W., Wren,B.W., Rycroft,A.N.
and Langford,P.R.
TITLE Direct Submission
JOURNAL Submitted (26-JAN-2018) Medicine, Imperial College London, Norfolk
Place, London W2 1PG, UK
FEATURES Location/Qualifiers
source 1..18149
/organism="Actinobacillus pleuropneumoniae"
/mol_type="genomic DNA"
/strain="3906"
/serovar="14"
/note="K locus: KL14"
/isolation_source="lung"
/host="pig"
/db_xref="taxon:715"
/country="Denmark"
CDS 1..1458
/gene="modF"
/codon_start=1
/transl_table=11
/product="putative molybdate ABC transporter ATP-binding
protein ModF"
/protein_id="AVY03713.1"
/translation="MPNINIQNALFSLAQHNKLSIESLEINTHDFWVIVGGNGSGKTA
FAQALHNSLSLYSGEYQNSFQHIALLSFEQQQKIIEQIFKHRNNDMVSPDDFGLTARQ
IILNGSEKMQLCEEYAAKLRIQPLLDRPFIQLSTGESRKVLFCQMLVSEPDLLILDEP
FEGLDQASVAYWQDVMAQLGKQMAVVLISNRFNDIPDCATHIALLDNLQLILQGERQE
IEQQAVYSQLKFAEQNVNAPLPESAAPLIQLPPNTNPFELKNVMIRYGEKNIIDDLTW
TVAPKQHWWIKGPNGAGKSTLLSIITGDHPQSYANYVHLFGRQRGSGETIWDIKKNIG
YVSSQLHMDYRVNCSALDVILSGFFDSIGVYQQVPSALQLKAMEWLERLHLANLAKKP
FRSLSWGQQRLLLITRAMVKHPPILILDEPLQGLDGVNRKLVKQFIEQLVTNSQTQLL

FVSHQDADAPNCITHLFEFVPQTNGGYRYVQTALN"

CDS complement(1657..2307)

/gene="cpxA"

/codon_start=1

/transl_table=11

/product="capsular polysaccharide export protein CpxA"

/protein_id="AVY03714.1"

/translation="MISVKNVSKDYYTRSGKKTVLQDINFELKKGEKIGILGRNGAGK

STLIRLLSGVEPPTSGTIERNMSISWPLAFSGAFQGSLTGMDNLRFICRIYNADIEYV

KAFTEEFSELGDYLYEPVKKYSSGMKARLAFALSLSVEFDCYLIDEVIAVGDSRFAAK

CKHELFEKRKDRSIILVSHSPSAMKSYCDNAMVLDKGIMYKFENMDEAYKFYNSTL"

CDS complement(2304..3101)

/gene="cpxB"

/codon_start=1

/transl_table=11

/product="capsular polysaccharide export protein CpxB"

/protein_id="AVY03715.1"

/translation="MQYGDQTTFRQSLAIQGRVIGALLMREIITRYGRKNLGFLWLFV

EPLLLTLFIVLMWKFIRADRVSDLNIIAFVITGYPMAMMWRNASNRTIGAISGNLSLL

YHRNVRVLDTLLARVILEVAGATIAQIIIMALVILLGWIEMPKDTFYMVMAWVLMAFF

ALGLGLIICSIAQKFEAFGKIWGTLSFVLLPLSGAFFFVHALPSQAQQYATLIPMIHG

TEMFRHGYFGDSVITYESISYLVICDVAMLLFGLIMVKNFSKGIEPQ"

CDS complement(3101..4258)

/gene="cpxC"

/codon_start=1

/transl_table=11

/product="capsular polysaccharide export inner membrane

protein CpxC"

/protein_id="AVY03716.1"

/translation="METTIMATPTEKLQKPVKQKKSWLKKLNPLFWVTVAIPTVLSAF

YFGSVASDIYISESSFVVRSPQNQTALTGVGALLQGSGFSRAQDDTYTVQEYMHSRTA

LEQLMKDLPIREYYENQGDIIARFNGFGLNNSKEAFYKYFRDRLSVDFDSVSGIASLR

IRAFNAEEGQQINQKLLAEGETLINRLNERARKDTISFAEQAVKEAENNVNATASDLS

KYRIKNKIFDLPAQSGVQLSLISSLKSELIRVETQLAQLQSITPDNPQVDALLMRQKS

LRKEIDEQSKQLSGNSNSSIAIQTADYQRLVLANELAQQQLTAALTSLQNTKNEADRQ

QLYLEVISQPSKPDWAEEPYRLYNILATFFIGLMLYGVLSLLIASVREHKN"

CDS complement(4284..5468)

/gene="cpxD"

/codon_start=1

/transl_table=11

/product="capsular polysaccharide export protein CpxD"

/protein_id="AVY03717.1"

/translation="MKLIKLRLLLSLGLVASLAACSSLPTSGPSHSAILEANSQNSDK

PLPEVNLVELDNGLVQQLYQTQQSQQFSGFLGTVSSAGYAGAVNVGDVLEISIWEAPP

AVLFGGTFSSEGQGSGHLTQLPAQMVNQNGTVTVPFVGNIRVAGKTPETIQSQIVGAL

QRKANQPQALVKIANNNSADVTVIRQGNSIRMPLTANNERVLDAVAAVGGTTENIEDV

TVKLTRGSEVKTLAFETLISDPAQNIMLRAGDVVSLLNTPYSFTGLGAVGNNQQMKFS

SKGLTLAEAIGKMGGLIDTRSDPRGVFVFRHVPFSQLSLDQQTQWGAKGYGMGMDVPT

VYRVNLLEPQSLFLLQRFPMQDKDIVYVSNAPLSEFQKFLRMIFSITSPVTSTTNAIR

AY"

CDS 5702..6814

/gene="cps14A"

/codon_start=1

/transl_table=11

/product="capsular polysaccharide phosphotransferase"

/protein_id="AVY03718.1"

/translation="MNKMNRKFSKLLKNPHIFFRDFLNKKYPIKNTELPFSESEEANL

IEANQKLDKIIQKNTLQQTNIDVVFTWVDGSDPSWQAKYSQYAPNYQAKSALYATDIA

RFEDHNELYYSVHAVLKYMPWVRHIFIITDNQKPKWLDETRQEKITLIDHQDIIDKEY

LPTFNSHVIEAFLHKIPNLSENFIYFNDDVFIARELQAEHFFQANGIASIFMSEKSLT

QMRDRGTITPTLSASEYSIRLLNKYYNTNIDSPLVHTYIPLKKSMYELAWQRYEKEIL

GFLPNKLRTNNDLNFANFLIPWLMYFEGKAMPKIDICYYFNIRSPNALTQYKKLLNKK

NIGEQPNSFCANDFNSQKSINNYQNQLFSFLNSYYS"

CDS 6825..8954

/gene="cps14B1"

/codon_start=1

/transl_table=11

/product="putative glycosyltransferase"

/protein_id="AVY03719.1"

/translation="MNKVKRKFRKLLRDPKLFFSDMYFKHSIKIKKHLPVKYEGKHQF

TIVSAVYNVEKYLDDFFDSIVKQNLSFKKYIQIILVDDGSKDSSANIIKKWQKKYPNN

IHYYYKENGGQASARNLGLKYVQTEWVTFIDPDDFISNNYFRELDNFLSLNNELVLVG

IPLIFYFEDKKIYKDTHPLKYRFSNGNKIFPTNDLENFIQLSASTALFKTRLINNIKF

DEEMKPSFEDAKFVMDYLITNRIIGKVGFISDIQYFYRKREDGSSTLDGAWQNKNLFS

KVLKHGCLSILESAEQQFGKIPTYIQRSVLYHLYWYFGRIVNNENALSHLTEYEKLEF

SQIVHRIFNKIDKSTIEKFNLGGAWFYHKVGFLGLFKKAEPSFQIAYIKKFDLIKNQV

LISFFSYQDVNYSLLINGEDRMPSFKKDIHYNFLNERFTNEYRIWLNVQELGNLSILL

NGKIAKLSFNGKWYNTLSMKVVREFYQSKSTKKENSWIFIDRDNQADDNAEHLYRYVM

NNHPEKDIYFALNKNSKDWDRLNKEGFKLLEFKSKEFENKLKNCSKIISSHIDGYITH

YFGDNGLLDKDYIFLQHGVTKDDMSPWLNTKENISIFITTTKDEYNSISEDGSPYRFT

KKEVKFLGFPRYDSLLSKNTFNTKNILVMPTWRQNIIGNSVNGSKRNFNSDFMETNYA

KHWHNFLNGDMVKKLVNQYGYNICSSS"

CDS 8926..9498

/gene="cps14B2"

/codon_start=1

/transl_table=11

/product="putative glycosyltransferase"

/protein_id="AVY03720.1"

/translation="MDIIFAPHPNIQEYLDVFTIPKYINTWRYSEGNIQKLFQEGMML

ITDYSSVAFDMAYLEKYTIYYQFDEKEVFSGSHTYRKGYFEYDKHGFGPVARTEDELN

AILERFLINKSDNFDYIYSSRIRNTFIHRDQDNCKRVYEAIVKLDSNIPDEQKFCYEI

ILESIKKAYQNEAWGVIYNRVNYLSKLNLI"

CDS 9756..10151

/gene="cps14B3"

/codon_start=1

/transl_table=11

/product="putative glycosyltransferase"

/protein_id="AVY03721.1"

/translation="MLNKVNENLYPIILAYVSASQKNWENVIFLLSKKISMFTKEELK

KYEPQLLLAKSYRHLKRYNEAHNMLVAFEKHTKDCSRCRIEISHLAYERADYKKCIDQ

LNKVFKFSLEYLPEESKRKYIESKNKLQK"

CDS 10192..11424

/gene="cps14C"

/codon_start=1

/transl_table=11

/product="capsular polysaccharide biosynthesis protein

Cps14C"

/protein_id="AVY03722.1"

/translation="MKEMNFNATNNIVVKYKHKKSKYDFNHVIFVFSGFLNASPGNYD

FSNALNDCPCDIIWINDEFEKMYTYYMCINMDFKVEEAITEFIYSKISELGLNKNHAT

LTGFSKGGSAALYYGLKLNFSNIVVSVPQMKIGSYIENNWKQVASHMMGKNYTIVDKN

YLDNILYKLLCQDTFLSRNIYLLTSEKDVQYSTEIVPYLSFFQKYTNFNLLKTHSAFV

REHNQVTSHHVPLLLSIYYALATDAIPTYSGGEVNFFGRLLFSDKNPTNEMVIDLRVA

KIINSHLFLEGVSFLQGNDLIEYSDVNYYLVLKLGESNIKLDLAKAHRPALTREFFNG

KSLTIYDKAWFTTYQYKGIDISVLPKGKYQLSLGIQLSKGLSKVSVLKDSRNIVRTDT

ENRYKLLSENNILYLEIL"

CDS 11438..13087

/gene="cps14D"

/codon_start=1

/transl_table=11

/product="capsular polysaccharide biosynthesis protein

Cps14D"

/protein_id="AVY03723.1"

/translation="MTHYQIENCLENWDFDAALEWHRYISDNNSDQIPNYCRYLADTG

HLDIAKSLIDSIKSNPVLYKKLSNDENFSVCRNLNSFFNKKLNEYANNPDYFCKLYMF

SLTGNIDKVFSLLTTYRGGHINSSTSAENNMIINFALNKLIEKNRLDVDISREIIIHL

ANSNKINNQRKKYLLKSMIDFIAKNHDLSKELFDLKHIYTIHIRLIPLIYAISNNENG

AKSLMSKVYSLIQDNNNLNMLNTEKPRIAICISGMFKSDLTNLKTIQTKLAIPLNADV

FIHTWDRQQDWMGDVRRYNFWPRVFNISNSLVPKNIQNLSFLEKNYSNVYSCLLSSVF

SSLDINQVKNNIISKSILIENENNFMREHHINDNFKSRETFNQIKMFYGLYKCFELAK

RKEDIEGFRYDYFIRLRADTIVNSNSISPEHLYALDNSSLAVPAGAGWGISDGFFYAN

RSVYERVISLWKKMKIANRLSPFEEFRDWDAHKLLGLWLLKNDIRPVPCKFSCGTIFG

GETLKVPGLLAALEKDNTQENRNKFPEETQWLMEFLKDKAK"

CDS 13149..13865

/gene="cps14E"

/codon_start=1

/transl_table=11

/product="capsular polysaccharide biosynthesis protein

Cps14E"

/protein_id="AVY03724.1"

/translation="MFIIPMAGLSSRFFKAGYTKPKYQLEIGNETVFSWSVRSFERYF

TTDKFIFIYRDVYETQDFLKQEIEKLGISDYELICLPEETLGQADTVYQGINHLPSDE

EIYIFNIDSKIIEFIKPEWVNECDGYLEVFKGEGDHWSFALAENNSKRVIRTTEKERI

SELCSDGLYYFKKKSIFESLFLNARKNNITSKNEYYIAPLYNDLIKQNGLVFYDLIEK

EDILFCGTPDEYITLLGNEK"

CDS 13862..14248

/gene="cps14F"

/codon_start=1

/transl_table=11

/product="capsular polysaccharide biosynthesis protein

Cps14F"

/protein_id="AVY03725.1"

/translation="MKRLIMDLDNTITLTENGDYRNAEPILDVIEKLQEYKKQGFEII

ISSSRNMRTYEGNVGKINVNTLPIIIDWLNRHNVPYDEIYVGKPWCGHDGFYVDDRAI

RPDEFAKMSYEEIRKLTKMDGEYDSN"

CDS 14235..15794

/gene="cps14G"

/codon_start=1

/transl_table=11

/product="capsular polysaccharide biosynthesis protein

Cps14G"

/protein_id="AVY03726.1"

/translation="MILINSAAYVNAEFRNEFGAIPPCFLPIGNRKLLTFQVNALREN

LGNDQHIVVSLPMNYSLSIDEKELIQNLNIQPVFVPEGISLGMAVLYVLNTVGHNEEV

LRLLHGDTLLNNIPLENDCIALVSTQEDYEWEVDSNTNIPLVWCGYFSFTSSQKFIKA

LATTQGDFVQSVHMYAKEEPNTIHKEVNEWYDLGHINTYFRSRSSITTQRAFNSLKIG

NGVVWKSGTPARKIEAEAHWFANLPAQLKRFTPQLINAGVLKGNPFYETEYLPILPLN

EIFVHGKNPVAFWEKVLNLITFYMSESRAHLPKENKDLLEKIHVDSLSLYSDKTYERL

EKYAEQSGIALDQSTRYNGIDLPSLREIATECVTRTLKLPEIPAIVHGDLCFSNIMYD

SRSNNIKVIDPRGLNIQQELTIYGNQSYDLAKLCHSFIGLYDFIIADSFKLEKSESLG

VKLHFNIDSRLEAIQDVFMRKTLLPNISNKDIIAPTILLFLSMIPLHFDKPHRQEAML

ANALRLYSEWL"

CDS 16542..16655

/gene="hypothetical"

/codon_start=1

/transl_table=11

/product="putative small periplasmic lipoprotein"

/protein_id="AVY03727.1"

/translation="MKKLLLAVLIASFGLAACGVKGPLYFPEQQPAQQQTK"

CDS 16779..17384

/gene="lysA"

/codon_start=1

/transl_table=11

/product="diaminopimelate decarboxylase"

/protein_id="AVY03728.1"

/translation="MNHFNYKNQQLFAEDVSVSDIINQHGTPAYIYSRDTLERHWHAF

DKAFGAHPHLICFAVKSNSNIALLNVMARLGSGFDIVSQGELERVLAAGGEPSKVVFS

GVAKSHSEIQRALEVGIRCFNIESIAELHRINEVAGQLGKIAPISLRVNPDVDAHTHP

YISTGLKENKFGVSVTNEGNKYWEKRYEKKHRRKTYCVKVR"

CDS complement(17607..18149)

/gene="ydeN"

/codon_start=1

/transl_table=11

/product="putative hydrolase YdeN"

/protein_id="AVY03729.1"

/translation="MKKVYVTHGYTANPTRNWFPWLKNELEKLGWECECLAMPNSDQP

NPQAWLEHHQNTLQLDENTLLIGHSLGCIALLNYLAVTQQKVKTAIFVSGFYEKLPTL

PELDSFADFYANQTACLPQKSYVISALNDVVVPHSFSDRLAQYLQADYIRLATGGHFV

DREGVTELPELLELLKQILK"

ORIGIN

1 atgccaaata tcaacatcca gaacgcctta ttttcccttg ctcaacacaa taaactgtcg

61 attgaatcgc tggaaatcaa tactcacgat ttctgggtga ttgtcggcgg taacggctcg

121 ggtaaaaccg ctttcgccca agcgctacac aattcacttt cgttatattc gggcgaatat

181 caaaatagtt tccagcatat cgctttactt tccttcgagc agcaacaaaa aatcatcgag

241 caaatcttta aacaccgtaa caatgatatg gtttcgcctg acgatttcgg tttaaccgct

301 cgtcaaatta tcttaaacgg tagcgaaaaa atgcaattat gcgaggaata tgcggctaaa

361 ttacgtattc agccgttatt agatcgcccg tttattcagc tctccaccgg cgaaagtcgc

421 aaggtgttat tttgccaaat gttagttagc gaaccggatt tgctgatttt agatgagcct

481 tttgaggggt tagaccaagc ctcggtcgct tattggcaag acgtgatggc acaactcggc

541 aagcaaatgg cggtggtact gatttccaac cgttttaatg atattcccga ctgtgccaca

601 catattgctt tactggataa cttacaactg attttacaag gcgaacgcca agagattgaa

661 caacaagcgg tctattctca gctaaaattt gcagaacaga atgtgaatgc accgttgccg

721 gagagtgccg caccgctgat tcaactccca ccgaatacta atccgtttga actgaaaaac

781 gtgatgattc gttacggcga aaaaaacatt atcgatgatc taacttggac ggttgcccca

841 aaacaacatt ggtggattaa aggtccgaac ggagcaggaa aatcgacctt actttctatt

901 attaccggcg atcatccgca atcttatgct aactacgtgc atttattcgg tcgtcagcgt

961 ggctcgggcg aaaccatttg ggatatcaag aaaaatatcg gctatgtgag cagccaatta

1021 catatggatt atcgggtgaa ttgctctgcg ttagacgtga ttttatccgg cttttttgat

1081 tcaatcggtg tttaccaaca agtgccaagt gcgttacagc tcaaagcaat ggaatggctg

1141 gaacgcttgc atttagccaa tctggcgaaa aaaccgttcc gttcactttc gtgggggcaa

1201 caacggttat tattgattac tcgtgctatg gtaaaacacc cgccgattct gattttagac

1261 gaaccgctgc aaggtttgga cggtgtaaac cgcaaattgg ttaaacaatt tatcgaacag

1321 cttgtgacta atagtcaaac ccagttgcta tttgtttcgc accaagatgc ggacgcccca

1381 aattgtatca cccatttatt tgaatttgtt ccgcaaacta acggtggtta ccgttatgtg

1441 cagacggcgt taaattaggt ttttgacctt taaaggaaat ccccctcttt agtaaagagg

1501 gattagggga gatttgtcaa tagagagata tgaaattgaa tagaacttca ttttttatat

1561 ttataaaagc gttaattagc atatttcttc gctaattcat tctgtcaaat ctcctcctgc

1621 ccctctttgc taaagagggg agatatgtgc gggactttaa agcgttgaat tatagaactt

1681 ataagcctcg tccatattct caaatttata cataatccct ttatctaata ccattgcatt

1741 atcgcaataa gacttcattg ctgacggact atgcgaaacc aaaataatcg aacgatcttt

1801 gcgcttttca aataattcat gtttacattt tgccgcaaag cgagagtcac ctaccgcaat

1861 tacctcatca attaagtagc aatcaaactc taccgaaagc gacaaagcaa aggcaagtct

1921 ggctttcatg ccggaggaat atttcttaac cggctcatat aaataatcac ccaattcgga

1981 aaattcttcg gtaaaggctt taacgtattc aatatccgca ttatatatac ggcaaataaa

2041 gcgtaaatta tccataccgg ttaaactgcc ttggaacgcc ccgctgaaag cgagcggcca

2101 agatatcgac atattacgtt cgatagtacc tgatgttggc ggctcaacac cacttaacaa

2161 acggattagc gttgatttcc ctgcaccgtt acgccctaaa ataccgattt tctcgccttt

2221 tttcagctca aaattaatat cttgcaatac ggttttttta ccgcttcgag tatagtaatc

2281 tttactcaca tttttacgc taatcattgc ggttcgattc ctttactgaa gttttttacc

2341 ataatgagcc caaaaagtaa catggctaca tcacatatta cgagatagct tatactttca

2401 tatgtgataa cactgtcgcc aaaataaccg tgacgaaaca tttccgtgcc gtgaatcatc

2461 ggtattaagg ttgcatattg ttgagcttgg cttggtagcg catgcacaaa gaaaaatgcg

2521 cctgaaagag gtaaaagaac aaagcttaat gttccccaga ttttgccaaa tgcttcaaat

2581 ttttgtgcaa tagaacaaat aatcaagcct aatcctaatg caaaaaatgc cattaatacc

2641 cacgccataa ccatataaaa cgtatctttc ggcatttcta tccagcctaa taaaatgact

2701 aatgccataa taatgatttg ggcaatcgtt gcacccgcta cctcaagtat gacacgagcc

2761 agtaaggtat ctaatacgcg aacattacga tgataaagaa gactcaagtt accggaaatt

2821 gcaccgatag tgcggtttga tgcattacgc cacatcattg ccattggata accggtaatc

2881 acaaaagcaa taatatttaa atcggaaacg cgatccgctc ggataaattt ccacatcaaa

2941 acgataaata aagtgagtaa tagcggctca acaaacagcc ataaaaaacc caaatttttt

3001 cgtccgtaac gcgtaataat ttcccgcatg agtaatgcac cgattactct tccttgaatg

3061 gcgagagatt ggcggaaagt tgtttgatca ccatattgca ttagtttttg tgctccctta

3121 cgcttgcaat taataaactt aatacaccat aaagcatcag accgataaag aatgtcgcta

3181 aaatattata taagcgataa ggctcttccg cccagtccgg tttgcttggc tgactgatta

3241 cttctaaata aagttgctgg cgatccgctt cgttttttgt attttgcaaa gaggttaagg

3301 ctgcggtcaa ttgttgctgt gccagctcgt ttgcaagtac taagcgttgg taatcggcag

3361 tttgaatagc aatagagcta ttactgttac cggaaagctg ttttgattgc tcatcgattt

3421 ccttacgtaa acttttttgg cgcataagca atgcatcaac ttgtgggttg tccggtgtaa

3481 tagattgcaa ttgagccaat tgtgtttcta cacgaatcaa ttcgcttttt aggctggaaa

3541 ttaatgaaag ttgtacgcca gattgtgccg gtaaatcaaa gattttattt ttgatacgat

3601 atttacttaa gtcgcttgcc gttgcgttta cattattttc cgcttcctta accgcttgtt

3661 ccgcaaatga aatggtatct tttcttgcac gttcgtttaa acggttgatg agtgtttcac

3721 cttcggcaag taatttttga ttaatttgtt gtccctcttc tgcattaaaa gcacgaatac

3781 gtaagctggc aataccggat acagaatcga aatcaacact taagcgatct cggaaatatt

3841 tgtaaaacgc ttctttacta ttatttaaac caaatccatt aaagcgagcg ataatatcgc

3901 cttgattctc atagtattca cgtattggta ggtctttcat taactgttct aatgccgtac

3961 gagaatgcat atattcttgt acggtataag tatcatcttg agcacgagaa aatccggaac

4021 cttgtaataa ggcaccgaca ccggttaaag cggtctgatt ttgaggagat cttacaacga

4081 agcttgattc cgaaatataa atatcagaag cgaccgaacc gaaataaaac gctgatagca

4141 cggtagggat tgcgacagtt acccaaaata acggattaag cttttttaac caactttttt

4201 tctgtttaac cggtttttgt agtttttctg tcggagttgc cataatagtt gtttccatat

4261 tttatcctta taaattcaat atattaatag gcacgaatag cattagtcgt acttgtaacc

4321 ggcgaagtaa tcgagaaaat cattctcaag aatttttgga attcggacaa cggtgcattt

4381 gatacataga caatatcttt atcttgcatc gggaagcgtt gtaataaaaa cagtgattgc

4441 ggctcaagta aattcacacg ataaaccgtc ggtacatcca tacccatacc atagcctttc

4501 gctccccatt gtgtttgctg atctaaactt aattgagaaa aaggcacatg acggaagacg

4561 aatacccctc tcggatctga acgggtatcg attaaacctc ccatcttacc gatagcttcg

4621 gcaagcgtaa gacctttact tgagaatttc atttgctggt tgttacccac cgcacctaaa

4681 ccggtaaaac tataaggtgt gtttagcagt gaaacaacat cgccagcacg taacataata

4741 ttttgtgccg gatcggaaat taacgtttcg aatgcgagtg tttttacttc agaaccacgg

4801 gttagcttga ccgtcacatc ttcaatgttt tccgttgttc cgcctactgc agcaaccgca

4861 tctaatacac gttcattatt agcggttaat ggcatacgaa tactattgcc ttgacgaata

4921 accgtaacat cagcagagtt attattcgca attttgacta atgcttgcgg ctgattcgct

4981 ttacgctgca atgccccaac aatttgagac tgaatcgttt caggtgtttt acctgcgaca

5041 cgaatatttc ccacaaacgg cacggttacc gtcccgtttt gattaaccat ttgtgccggt

5101 aattgcgtta aatgcccgct accttgtcct tcggaactaa atgtaccgcc aaataatact

5161 gccggcggcg cttcccaaat tgaaatttca agtacatcac ccacattgac tgcaccggca

5221 tagcccgcgc tgcttactgt gcctaaaaat ccggaaaatt gttggctttg ctgagtctga

5281 tacaactgct gaactaagcc attatctaac tccactaaat taacttccgg taaaggttta

5341 tctgagttct gggaattagc ctctaaaatc gcactatggc tagggcctga agtgggtaag

5401 cttgagcagg cagccaaact agcaaccagc cccaaagaaa ggagtaatct aagtttgatg

5461 agtttcatct aatttctctt caatatatta aggaataaca actatatagg tatgtcttaa

5521 aatccatata aagattgatt ttaataagtt acctaatcaa gagaaattaa atataaaaaa

5581 tttacaaaaa agcaataatg cgtataaaaa aacatcattt gcaaagaaag taaatagaga

5641 ggagggttca acagataagc attataatcc aagatttata taaaatataa ttgataatat

5701 aatgaacaaa atgaatagaa aattttctaa gttactaaaa aatccacata ttttttttag

5761 ggattttcta aataaaaagt accctataaa aaatacggaa cttcccttct cagaatctga

5821 agaagctaac ttaatagaag caaaccaaaa attagataag attatccaaa agaatacgtt

5881 gcaacaaact aatattgatg tggtatttac ttgggtagat ggttctgatc cttcatggca

5941 agctaaatat tcccaatatg caccaaatta tcaagcgaaa tccgctctat atgcaacgga

6001 tatcgcccga tttgaagatc ataatgaatt atattattca gtacatgctg tacttaaata

6061 tatgccttgg gttaggcata tatttattat aacagataat caaaagccaa agtggctgga

6121 tgagacgaga caagaaaaaa ttacactaat cgatcatcaa gatattatag ataaagaata

6181 tcttccaacg tttaattccc atgttattga agcattttta cataaaattc ctaatttaag

6241 cgagaatttt atctatttta atgatgatgt ctttattgca cgagaactac aagctgaaca

6301 ctttttccaa gcaaatggta ttgcctctat atttatgtcg gaaaaaagcc tcactcaaat

6361 gcgtgacaga ggaactatta caccgactct ttctgcttcg gaatatagta ttcgcttact

6421 aaacaaatat tacaatacaa atattgactc accactcgta cacacttata tcccattgaa

6481 aaaaagtatg tatgaattgg catggcagcg ttatgagaaa gaaattcttg gatttttacc

6541 caataaatta agaacaaata acgatttaaa ttttgcaaac tttcttattc cttggttaat

6601 gtatttcgaa gggaaagcaa tgcctaaaat agatatttgt tattatttta atattagatc

6661 tccaaatgca cttacacaat ataaaaaact tttaaataaa aaaaacatag gcgaacagcc

6721 taattcattt tgcgcaaatg attttaatag tcaaaaaagt attaacaact atcaaaatca

6781 attgttttct tttttaaact cctattacag ttaaggataa tataatgaat aaagtaaaac

6841 gtaaatttag aaaattactg cgtgatccta agttgttttt tagtgatatg tatttcaaac

6901 attctataaa aataaaaaaa catttacctg ttaaatatga aggaaaacat caatttacga

6961 ttgtttccgc tgtatataat gtagaaaaat atcttgatga tttctttgat agtatcgtta

7021 aacaaaattt atcatttaaa aaatacatac agattatctt agttgatgac ggctcaaaag

7081 attcatcagc aaacatcatc aaaaaatggc aaaaaaaata tccaaataat atccactatt

7141 attataaaga aaatggtggg caagcctctg ctcgcaattt aggactaaaa tacgtacaaa

7201 cagaatgggt tacctttatt gatccggatg attttattag caacaactac tttagagagc

7261 tagataattt cttaagcctg aataacgagt tagtcttagt tggaattccg ttaatatttt

7321 attttgaaga taaaaaaatc tataaagata ctcatccatt aaagtacaga ttctctaatg

7381 gtaataaaat ttttccaaca aatgatctag agaatttcat tcagctatct gccagtactg

7441 ctctatttaa aactagatta ataaataata ttaaattcga tgaggaaatg aaaccctcat

7501 ttgaagacgc taaatttgta atggattatc taataacaaa tagaattatt gggaaagtag

7561 gatttatttc tgatattcag tatttttacc gtaaacgaga agatggtagc tcaaccctgg

7621 atggcgcgtg gcaaaataaa aatctattta gtaaggtatt aaagcatggc tgcctctcta

7681 ttctagaatc agcagaacag caatttggta aaattccgac atatattcaa cgatcagtac

7741 tttatcattt atattggtat tttgggagaa tcgttaacaa tgagaatgct ctatctcatt

7801 taacagaata tgaaaaacta gaattctcac aaattgttca tagaatattt aataaaatag

7861 acaaaagtac tatagaaaaa tttaacttag gaggggcttg gttttatcat aaagtgggat

7921 ttttagggct attcaaaaaa gctgaaccta gtttccagat tgcctatatc aaaaaatttg

7981 atttaataaa aaatcaagta ctaattagct tcttctcgta ccaggacgta aactattcgc

8041 tacttattaa cggagaagat agaatgccta gctttaaaaa agatattcat tataactttt

8101 taaacgaacg ctttacaaat gaatatagaa tttggctaaa tgttcaagag ctaggaaatc

8161 tttctatttt actaaatggt aaaatagcaa aattatcttt taatggaaaa tggtataaca

8221 ctttatcgat gaaagttgtg cgagagtttt atcagagtaa atcaactaaa aaagaaaata

8281 gttggatctt tatagacaga gataatcaag ctgatgataa tgcagaacat ttatatagat

8341 atgtgatgaa taaccatcct gaaaaagata tatattttgc tttaaataaa aactcaaaag

8401 actgggatag attaaataaa gaagggttca aattactaga atttaaatct aaagaatttg

8461 aaaataaatt aaaaaattgc agtaaaatta ttagtagcca tatagatggg tatattactc

8521 attactttgg tgacaatggt ttattagata aagattatat atttctacaa catggagtaa

8581 caaaagatga tatgtcccct tggttaaata cgaaagaaaa tatatctatt tttattacaa

8641 caacaaaaga tgaatataac tctattagtg aagatggttc tccatatcga tttacaaaaa

8701 aagaagtaaa attcttaggt tttccaagat atgattcctt attatctaaa aatactttca

8761 atactaaaaa tattttagtt atgccaactt ggcgacaaaa tattataggt aattcagtaa

8821 atggttctaa acgaaacttc aattcagatt ttatggaaac aaattatgct aagcattggc

8881 ataatttctt aaatggagat atggtaaaaa aactagttaa tcaatatgga tataatattt

8941 gctcctcatc ctaatattca agaatatttg gatgtattta ctattcccaa atacattaat

9001 acatggcgtt attcagaagg aaatattcaa aaattattcc aagaaggaat gatgctaatt

9061 actgattatt catctgttgc atttgatatg gcttacctag aaaaatatac catttattat

9121 caatttgacg agaaagaagt tttttctggc tcgcatacat atagaaaagg atattttgaa

9181 tatgacaagc atggttttgg acctgtagcg agaacagaag atgaactaaa tgctatttta

9241 gaaagattct taattaataa atctgataat tttgattata tctactcctc tagaattaga

9301 aacaccttta ttcatagaga tcaagataat tgcaaaagag tctatgaagc tatagttaag

9361 ctagattcaa atataccaga tgagcaaaag ttttgttacg aaattatatt ggaatccata

9421 aaaaaagcat accagaatga agcttggggt gtaatctata atagagtaaa ttatttatct

9481 aaattaaatt taatttaatt agagaagaag ataaagaatg ggtaactaga atttatctag

9541 catcaataat aaaaattagg cattttaatg agatagaaaa atttatacct aataataaat

9601 atagcgatat aatcctatcg ctttactatg aaactcatca acatgagaag gccttagaat

9661 tattaagtca aagccctgaa cctgatttat tcatgctttt atactcttat tctaatttat

9721 atgacttttt caacgcaaaa aaaatatcta ttaagatgct taataaggtc aatgaaaatt

9781 tatacccaat tattttggcc tatgtatctg cgagtcaaaa aaattgggaa aatgtaatat

9841 tcttattaag taaaaaaata tccatgttta ctaaagagga actaaaaaaa tatgagccac

9901 aactattatt agctaaatca tataggcatt taaaaagata taatgaagcc cataatatgc

9961 tagttgcttt tgaaaaacat actaaagatt gctctagatg taggatagaa atttctcact

10021 tagcatatga acgtgcagat tataaaaaat gtatcgatca gttaaataag gtctttaaat

10081 tttctttaga atatcttcca gaagaaagca aaagaaaata tatcgaatca aagaataaat

10141 tacaaaaata aattattaat aataatttaa tcaaaatagg acattaatta aatgaaagaa

10201 atgaacttta atgctacaaa taatatagta gttaaatata agcataaaaa atccaaatat

10261 gatttcaatc acgtcatttt tgtattttca ggtttcttaa acgcatcacc tggaaactat

10321 gatttttcta atgcattaaa tgattgccct tgcgatatca tttggataaa tgatgagttt

10381 gaaaaaatgt acacatatta catgtgtatt aatatggatt ttaaagtgga agaagcgatc

10441 accgaattta tatatagcaa aatatctgag ttaggattga ataagaatca tgctactcta

10501 acaggatttt ccaaaggagg gagtgctgct ttatattatg gacttaaatt aaatttttct

10561 aatattgtag ttagtgttcc tcaaatgaaa attggtagtt atattgaaaa caattggaaa

10621 caagtagcat cccatatgat gggcaaaaac tatacaatag tagataagaa ttatttagat

10681 aatatcctat ataaactatt atgtcaggat acttttcttt cacgtaatat ctatctacta

10741 acatctgaaa aagatgtaca gtattcaacg gaaattgttc catatttaag ctttttccaa

10801 aaatatacta actttaattt attaaaaaca cactcagctt ttgttagaga acataatcag

10861 gttactagtc atcatgttcc attgttatta agtatttatt atgcattagc aaccgatgct

10921 atccctactt atagtggtgg agaggtgaat ttctttggac gtttattatt tagtgataaa

10981 aatcctacaa atgaaatggt catagattta cgtgtagcta aaattattaa tagtcattta

11041 tttttagaag gagtatcttt tttgcaagga aatgatctca ttgaatattc tgatgttaat

11101 tactatttag tattaaaatt aggagaatct aatattaaat tagatttagc taaagctcac

11161 agaccagcat tgacaagaga atttttcaat ggaaaatcat taactatata tgataaagct

11221 tggtttacaa cttatcaata taagggaata gatatatctg tattacctaa agggaaatat

11281 caattatcac tcggtattca attaagtaaa ggacttagta aagtttctgt gctaaaagat

11341 agtagaaata ttgttagaac agatacagaa aatagatata aactattatc agaaaataat

11401 attttatact tagaaatctt ataataagga atatgatatg actcactatc aaatagagaa

11461 ctgtttagaa aattgggatt ttgatgctgc tctagaatgg catagatata tctctgataa

11521 taattcggat caaataccaa actattgtag gtatttagca gacacgggac atttagatat

11581 agctaaatca ttaatagata gtataaaatc caatcctgtg ctatataaaa aattatcaaa

11641 tgatgaaaat ttttctgtct gccgtaattt gaattctttc tttaataaga aactaaatga

11701 atacgcaaac aatccagatt acttttgtaa actatatatg ttttctctta ctggcaatat

11761 tgataaagta ttttcactat taaccactta tagaggtgga catataaact cttctacctc

11821 tgcagaaaat aatatgatta ttaactttgc gttaaataag cttattgaga agaacaggtt

11881 agatgtagat atatcaagag aaattattat ccatcttgca aattcaaaca aaataaataa

11941 tcaaagaaaa aaatatttac taaaatctat gatagatttc attgccaaaa atcatgatct

12001 atctaaggag ctatttgatt taaagcatat ctatactatt catattaggc ttattccttt

12061 aatttatgcc atctcaaata atgaaaatgg tgcaaaatca ttaatgtcta aagtatattc

12121 attaattcaa gataataata atttgaatat gctaaataca gaaaaaccaa gaatagctat

12181 ctgtattagt ggtatgttta agtcagattt gaccaactta aaaactattc aaactaaact

12241 agcaatccct ctaaatgctg atgtttttat ccatacttgg gatcgacagc aagattggat

12301 gggagatgtt cgtagatata acttttggcc tagagtattt aatataagta attcgttagt

12361 gcctaaaaat atccaaaatt tatctttttt agaaaaaaac tacagcaatg tatactcatg

12421 tctcttatct tctgtatttt catcactcga tattaatcag gttaaaaata atataatttc

12481 taaaagtata ttgattgaaa atgaaaataa ttttatgcga gaacatcata taaatgataa

12541 tttcaaatct agagaaacat tcaatcagat aaaaatgttc tatgggctat ataaatgctt

12601 tgagttagct aagaggaaag aagatataga aggatttaga tatgattatt ttatacgctt

12661 acgagcagat actattgtaa attctaactc tatatctcca gaacatctat atgccttaga

12721 taatagctct ttagctgttc cagcaggtgc tggatggggt atatctgatg gtttctttta

12781 tgcaaataga agcgtatatg agcgagttat atccttatgg aaaaaaatga aaatagctaa

12841 tagattaagt ccttttgaag aattcagaga ttgggatgcg cataaattat tagggttatg

12901 gttgctaaaa aatgatataa gacctgtacc ttgtaaattc tcttgtggaa ctatttttgg

12961 tggtgaaact ttaaaagtac caggtctatt agcagcccta gaaaaagata atactcaaga

13021 gaatcgaaac aaattccctg aggaaactca atggttaatg gagtttctca aagataaagc

13081 taaataataa ataaaaggcg agatcacttc tcgccttttg gttttaattt ttaataaaag

13141 gatacaatat gtttattatt cccatggcag gattaagttc ccgctttttt aaggccggtt

13201 atacaaaacc gaaataccaa ttagaaattg gtaatgaaac agtattttca tggtctgtgc

13261 gatcttttga acgttacttt acaacagata aatttatctt tatctatcgt gatgtatatg

13321 aaacgcaaga cttcctaaag caagaaatag aaaaattagg tatctcagat tatgagttaa

13381 tctgtttacc agaagaaact ttagggcagg cagatactgt gtatcaaggt attaaccatt

13441 tacctagtga tgaggaaatt tatatattta atattgattc taaaattatt gaatttataa

13501 aaccagaatg ggtaaatgaa tgcgatggct atttagaagt ctttaaggga gaaggtgatc

13561 actggtcatt tgctcttgct gaaaataatt ctaaacgtgt aattcgcaca acagaaaaag

13621 aaagaatatc tgaactctgt agtgatggat tatattattt taaaaagaaa tccatttttg

13681 aatcattatt tttaaatgca agaaaaaata atataacatc aaagaatgaa tattatatag

13741 cccctcttta taatgatctc ataaaacaaa atggattggt tttttatgat cttattgaaa

13801 aagaagatat tttattttgt ggcacaccag atgaatatat tactttatta ggaaatgaaa

13861 aatgaagcgc ttaataatgg atttagataa tacaattact ctaacagaga atggtgatta

13921 tcgtaatgca gaaccaattt tagatgtaat tgagaaatta caagaatata agaaacaagg

13981 ctttgaaatt attattagtt caagtcgtaa tatgcgtact tatgaaggaa atgtgggaaa

14041 aattaatgtt aacacattac caattattat agattggcta aatcgccata atgtgccata

14101 tgatgaaatc tatgttggta aaccttggtg tgggcatgat ggcttttatg ttgatgatag

14161 agctatcaga ccagatgaat ttgctaaaat gagttatgaa gaaattcgaa aactaacaaa

14221 aatggatggt gaatatgatt ctaattaact ccgctgcata cgtgaatgct gaatttcgta

14281 atgagtttgg cgctattccc ccatgttttt taccaattgg taatcgtaaa ctattaactt

14341 ttcaagttaa tgctttacga gagaacctag gtaatgacca acatatcgtg gtttctttac

14401 ctatgaatta ttcactaagc attgatgaga aagaattaat tcagaattta aatattcagc

14461 ctgtttttgt acctgaagga atatcattag gtatggctgt tctctatgta ttgaacactg

14521 ttggtcataa tgaagaagta ctaagattac ttcatggaga tactctccta aataatattc

14581 ctctcgaaaa tgactgcatt gcacttgtca gtacacaaga agattacgaa tgggaagtgg

14641 attcaaatac aaatattcca ctcgtttggt gtggttactt ctcttttaca tcaagccaaa

14701 aatttattaa ggctcttgct acaacacagg gggattttgt ccaatctgtg catatgtatg

14761 ctaaggaaga accaaatacc attcataaag aggttaatga atggtatgac ttaggtcata

14821 ttaatactta ttttagatct cgttcttcta ttacgacaca aagagcattc aattcattaa

14881 aaattggaaa tggcgttgtt tggaaatctg gtacacctgc tagaaaaatt gaagctgaag

14941 cacattggtt tgccaatttg cccgcacaac taaaacgctt tactcctcaa ttaatcaatg

15001 ctggtgtact taagggaaat ccattttatg agacagaata tctcccaatt cttcctctaa

15061 atgagatttt cgttcatggg aaaaaccctg ttgctttttg ggaaaaggta ctcaatttaa

15121 ttacatttta tatgagtgaa tctcgagcac atttacctaa agaaaataaa gatctattgg

15181 agaaaataca cgtagattct ttatcactct attcagataa gacttatgaa cgattagaaa

15241 aatatgctga acaaagcggt atagctctag atcaatcgac tcgttataac ggtattgatt

15301 taccatcttt gagagaaatc gcaactgagt gtgtaacaag aacattaaaa ctacctgaaa

15361 tcccagctat agtacacggg gatttatgct tcagtaatat aatgtatgac tctcgtagca

15421 ataatattaa agttattgat ccgagaggat taaatattca gcaagaatta acaatctacg

15481 gaaaccaaag ctatgattta gcaaaacttt gccactcatt tattggttta tatgatttca

15541 ttattgcgga ttcgtttaaa ttagaaaaat cagaatcatt aggagtgaaa ttacacttta

15601 atattgattc tcgcttggaa gcaattcaag atgtatttat gagaaaaact ttattaccaa

15661 atattagtaa caaagatatt attgctccaa caatacttct tttcttgtca atgatcccat

15721 tgcatttcga taaaccacat aggcaagaag caatgttagc taatgcatta cgcttatatt

15781 ctgaatggtt ataataattt aatatttatt tatctttcta gaacaagcgg tcaaattttt

15841 cttaaaactt gcaaagttta ggaaaaataa gaccgcttat tttattcata ttcttatata

15901 aaattagagt ttattatgag cgtagccaca tataattgat aaagatatgc ggtatagcaa

15961 tattaataca atcctatcaa tttcatatta tcttaatcaa atttattttt tcgtttttta

16021 aatattaaac tattatttac ttctgcaaat ctcccctatc acctctttac taaagagggg

16081 gatttccttt agatataaac aagctatgat cgttcgaata gctttggtct ctcaatagat

16141 tcaggtaaat ggtcgatata taaggaaaat aaaaacattc gttaaagacg agcgccatct

16201 tagagaaatt tagagccaca gcgtggctca ctcagccgta ggctgatcgt aaccacgtac

16261 ggcttgccgt gcgtggctgt tacatcatga ggcgttttag ctacttcccc ctctccctgt

16321 ttttttcttc tagacgaaga aaaatctgtc cctctcccac aaggggcgag ggtgattatt

16381 tataagggca ttcgttaaag acgagtgcca tcgtaaagaa atttggagcc acagcgtggc

16441 tattgggtga ttatttaaaa acaggcgaaa cggctttttt actttttcat taccgcaatt

16501 tgttttagaa tcaacggacg atttactctc aaggatttat gatgaaaaaa ttacttttgg

16561 cggtgttaat cgcatcgttc gggttggctg cctgcggtgt aaaaggcccg ctttattttc

16621 ccgagcagca accggctcaa cagcaaacta aataatttct aaccacggta attacaaaat

16681 acctgtataa caagcagtcg gatttatccg attttttgta aatgcgagtg tcgcgagttt

16741 gccgtggttt tattttttca aacacaacgg acaaatcaat gaatcatttc aattataaaa

16801 accaacaact ttttgcggaa gacgtttccg tttcagatat catcaatcaa cacggtacgc

16861 ccgcttatat ctattctcgt gatacgcttg agcgtcattg gcacgctttt gataaagcat

16921 tcggcgcaca cccgcacttg atttgctttg cggtaaaatc caattccaat atcgctttat

16981 taaatgtaat ggcacgcctc ggttcgggct ttgatattgt gtcgcaaggc gaacttgaac

17041 gtgtacttgc cgccggcggc gagccgagca aagtggtatt ttccggtgtg gcaaaatcac

17101 atagcgaaat tcagcgtgca ttggaagtcg gcattcgttg ctttaatatc gaatccatcg

17161 ccgagttaca ccgcattaat gaagttgccg gtcaattagg taaaatcgca ccgatttcat

17221 tgcgtgtaaa tccggatgtg gatgcacata ctcaccctta tatttccacc ggtttaaaag

17281 aaaataaatt tggggtaagc gtaacgaacg agggaaacaa gtactgggag aagcgttacg

17341 agaaaaaaca caggcggaaa acctactgcg tgaaagtgcg ctaaattgga cgattctccg

17401 cccgtgcggg ctgaatacgg acgaaggcga aacctttcgt ttaattgaaa atgcggctga

17461 actgcccggc agttatatga gccgtaaagc attagccaat gcggtcttgt ccgtacttaa

17521 cagtgaaaac acaaactata aaatcttctc agtctgtgcc taacttcaca atccctttca

17581 ctttggcaca agccatccgc ttgtgcctat ttcaaaattt gttttaacag ttccaataac

17641 tccggtaatt ccgttactcc ctctcgatcg acaaaatgcc cgcccgttgc caagcgaata

17701 taatccgctt gtaagtattg cgctaatcga tcgctgaacg aatggggaac gacaacatca

17761 tttaatgcag atatgacgta agacttttgc ggtaaacaag cggtctgatt tgcataaaaa

17821 tctgcaaagc tatctaattc cggcaaagtt ggtaatttct cataaaagcc ggaaacaaaa

17881 attgccgttt ttactttttg ctgcgttacc gccagataat tcagtaacgc aatgcagccc

17941 aaactatgtc cgatgagtaa ggtattttca tctaattgaa gtgtattttg gtgatgttcc

18001 agccatgctt gcggattcgg ctgatcggaa ttcggcatcg ctaaacattc acattcccat

18061 cctaattttt ccaattcgtt tttaagccac ggaaaccaat ttcttgtcgg gttcgccgta

18121 taaccgtgcg ttacatatac ttttttcat

//

LOCUS **MG868949 .1** 12857 bp DNA linear BCT 16-MAR-2021

DEFINITION Actinobacillus pleuropneumoniae strain HS 143 capsular

polysaccharide gene locus, complete sequence.

ACCESSION MG868949

VERSION MG868949.1

KEYWORDS .

SOURCE Actinobacillus pleuropneumoniae

ORGANISM Actinobacillus pleuropneumoniae

Bacteria; Proteobacteria; Gammaproteobacteria; Pasteurellales;

Pasteurellaceae; Actinobacillus.

REFERENCE 1 (bases 1 to 12857)

AUTHORS Bosse,J.T., Li,Y., Fernandez Crespo,R., Lacouture,S.,

Gottschalk,M., Sarkozi,R., Fodor,L., Casas Amoribieta,M., Angen,O.,

Nedbalcova,K., Holden,M.T., Maskell,D.J., Tucker,A.W., Wren,B.W.,

Rycroft,A.N. and Langford,P.R.

CONSRTM BRaDP1T consortium

TITLE Comparative sequence analysis of the capsular polysaccharide loci

of Actinobacillus pleuropneumoniae serovars 1-18, and development

of two multiplex PCRs for comprehensive capsule typing

JOURNAL Vet. Microbiol. 220, 83-89 (2018)

PUBMED 29885806

REFERENCE 2 (bases 1 to 12857)

AUTHORS Bosse,J.T., Li,Y., Fernandez Crespo,R., Lacouture,S.,

Gottschalk,M., Sarkozi,R., Fodor,L., Angen,O., Nedbalcova,K.,

Holden,M.T.G., Maskell,D.J., Tucker,A.W., Wren,B.W., Rycroft,A.N.

and Langford,P.R.

TITLE Direct Submission

JOURNAL Submitted (26-JAN-2018) Medicine, Imperial College London, Norfolk

Place, London W2 1PG, UK

FEATURES Location/Qualifiers

source 1..12857

/organism="Actinobacillus pleuropneumoniae"

/mol_type="genomic DNA"

/strain="HS 143"

/serovar="15"

/note="K locus: KL15"

/isolation_source="lung"

/host="pig"

/db_xref="taxon:715"

/country="Australia"

CDS 1..1458

/gene="modF"

/codon_start=1

/transl_table=11

/product="putative molybdate ABC transporter ATP-binding

protein ModF"

/protein_id="AVY03730.1"

/translation="MPNINIQNALFSLAQHNKLSIESLEINTHDFWVIVGGNGSGKTA

FAQALHNSLSLYSGEYQNSFQHIALLSFEQQQKIIEQIFKHRNNDMVSPDDFGLTARQ

IILNGSERTQLCEEYAAKLRIQPLLDRPFIQLSTGESRKVLFCQMLVSEPDLLILDEP

FEGLDQASVTYWQEVMAQLGKQMAVVLISNRFNDIPDCATHIALLDNLQLILQGERQE

IEQQAVYSQLKFAEQNVNAPLLESATPLIQLPPNTNPFELKNVMIRYGEKTIIDDLTW

TVAPKQHWWIKGPNGAGKSTLLSIIAGDHPQSYANYVHLFGRQRGSGETIWDIKKNIG

YVSSQLHMDYRVNCSALDVILSGFFDSIGVYQQVPSALQLKAMEWLERLHLANLAKKP

FRSLSWGQQRLLLITRAMVKHPPILILDEPLQGLDGVNRKLVKQFIEQLVTNSQTQLL

FVSHQDADAPNCITHLFEFVPQTNGGYRYVQTALN"

CDS complement(1657..2307)

/gene="cpxA"

/codon_start=1

/transl_table=11

/product="capsular polysaccharide export protein CpxA"

/protein_id="AVY03731.1"

/translation="MISVKDVSKRYHTNSGWKTVLQNINFDLHKGEKIGILGRNGAGK

STLIRLMSGVEPPTTGKIERHMSISWPLAFSGAFQGSLTGMDNLRFICRIYNADFEYV

KAFTEEFSELGDYLYEPVKKYSSGMKARLAFALSLSVEFDCYLIDEVIAVGDSRFAAK

CKYELFEKRKDRSIILVSHSPSAMKEYCDNAMVLDKGIMHKFENMDDAYKFYNSTP"

CDS complement(2304..3101)

/gene="cpxB"

/codon_start=1

/transl_table=11

/product="capsular polysaccharide export protein CpxB"

/protein_id="AVY03732.1"

/translation="MQYGDQTTFRQSLAIQGRVIYALLMREIITRYGRKNLGFLWLFI

EPLLLTFLIVLMWKFFRADKVSTLNIVAFTITGYPMAMMWRNVSNRAIGSISANLSLL

YHRNVRVLDTIFARMLLEVAGATVAQIIITAVLVFIGWIDPPKDVFYMVLAWTLMAFF

AFGLGLIICSLAQKIEVFGKIWNTLSFVLLPLSGAFFFVHSLPSQIREIAQWVPMISG

TEMFRHGYFGDLVPTYENIGFLVVCDLAMLLLGLILVRNFSKGIEPQ"

CDS complement(3101..4240)

/gene="cpxC"

/codon_start=1

/transl_table=11

/product="capsular polysaccharide export inner membrane

protein CpxC"

/protein_id="AVY03733.1"

/translation="MVAITAEKPNKPKKKSLLRKLNPLLWLTVIFPTLFSAIYFGMFA

SDIYVSESSFVVRSPRNQSSLSGVGALLQGTGFTRSQDDTYSVQEYMRSRTALEQLQT

ELPVRDFYATKGDILSRFNGFGLNDTQEAFFRYFKDRLSIDVDSVSGIATLRVQAFEA

SEGQQLNEKLLRLGEDLINRLNARGRKDTLEFAAQAVQEAEKNVNETAEALSKYRIKN

KIFDLPAQSGVQLSLISSLKSELIRVETQLAQLQSITPDNPQVDALLMRQKSLRKEID

EQSKQLSGNTGSSVANQSADYQRLVLANELAQQQLTAAMASLHNTRGEADRQQLYLEV

ISQPSKPDWALAPSRLYNIIATFIIGLMLYGIFGLLITSVREHKN"

CDS complement(4290..5477)

/gene="cpxD"

/codon_start=1

/transl_table=11

/product="capsular polysaccharide export protein CpxD"

/protein_id="AVY03734.1"

/translation="MQSKKHNSIIGLTLAALLITACSILPTSGPSHSAILEANSKNSD

KPLPEVNLVELDNGLVQQLYQTQQSQQFSGFLGTVGGAGYAGAVNVGDVLEISIWEAP

PAVLFGGTFSSEGQGSGHLTQLPAQMVNQNGTVTVPFVGNIRVAGKTPEAIQSQIIGA

LQRKANHPQALVKIANNNSADVTVIRQGNSIRMPLTANNERVLDAVAAVGGTTENIED

VTVKLTRGSEVKTLAFETLISDPAQNIMLRAGDVVSLLNTPYSFTGLGAVGNNQQMKF

SSKGITLAEAIGKMGGLIDTRSDPRGVFVFRHVPFSQLSLDQQAQWGSKGYGMGMDVP

TVYRVNLLEPQSLFLLQRFPMQDKDIVYVSNAPLSEFQKFLRMIFSVTSPITSTTNSI

RSY"

CDS complement(5548..6690)

/gene="cps15C"

/codon_start=1

/transl_table=11

/product="capsular polysaccharide biosynthesis protein

Cps15C"

/protein_id="AVY03735.1"

/translation="MRNLDVKISKIELTEKNTLRVPPRKEEDQQPNYKELFDYHTLFS

DAFFSEENIELVGPPLLNLHNILLSGEIYIDEKNVTKEAIIQSEHRKCRVLLPKIPGA

KKVIIKFEDVLFEQEIQPDESDFFANHNVLVTQQKDNPLEWIGYWIAHHIKHHKINAV

LIYDNDSSLYDINQLKTFLSSIKGLEKICVVPWSIPYGVTGGDKQIWDSDFGQYQSWE

HALKRFVYSANCVVIGDVDELVIHKDGLSLPDILDSIDEPVITYKRRQIIEVASTEFT

NLPRMHNHTHLYEKERILYAPKYAFKPKKLSKKVHLLVHKVEGDKSKFSEELLGRHFG

ILRLHWRVGNFEPIELRDRSSYKTPLSEDMELFQSFNEVDLSWLKG"

CDS complement(6710..9241)

/gene="cps15B"

/codon_start=1

/transl_table=11

/product="putative glycosyltransferase"

/protein_id="AVY03736.1"

/translation="MFKRKFNKLRKDPILFFKDAYWNFRLKKQRNEKTSYGTYQYCII

CPTFNVDEYIDDFFQSIKNQTLDFTKHIHVIFVDDGSTDLSAKKIEKFKQKYPRNVTY

LYKTNGGLSSARNYGLDYLEDNNIYFDYVTFTDPDDILDKDFFKSLDNFFIKNKNCKI

ASSNLIYFYEHNNTFRDIHPLRFRYVKTHTVQNDFGNDLLLSAATSIYNLQFLYNLGV

RFDENVKPSFEDCKFNSQLLIVSPDVQVGFVKEARYLYRQRENNTSLMNNSWKKVGLF

TNVPKNGVLNILELANKTLGYVPIHIQRVALFHCIGYYRRLVNADYHVNFLTIQERQI

FKDFLKEIFKFIDVDTIIKCEFSNLDRKTKVGILGLYKGQALPIHYTYVNYIDKERKE

FCISYFSFNTDDTIKVTLDDLNIEITEEKFILNDFMGDKFSIERRFLFKYEDTNQNLA

ISINNRKVNLTCFTKGYTGGAVITELISSMNKNIIYPNLKDTWILMDRKDRGDDNAEH

FYRYLQKKHPEQNIFYAISKNCSDWKRLERDGFNLLDYGSSYFSRELKNCTYIVSSHL

FIWNHLVSKGLLSLASKKKIWLQHGVICNNNANVVNTKHVDFMVTSTKPEYNSIAANF

TDYNLLPSQVLLSGLPRHDSLIKKSKVVKKEKIILVMPTWRTWLNQDNISDSEYLRNW

VELLSSEKLNSLLNKYGYKIVFAPHQELNKYSHLFVENQYISIWNPSNNESMQELFLK

SSCMITDYSSVAFEMGFLNKCVFYYQFDQSEFYSKHYKKGYFDFYNDGFGPVAEQKVD

LLKQLESYLKSPQQMEKKYTSRMDVFAYKDGRSCERIYQRIMNHK"

CDS complement(9234..10415)

/gene="cps15A"

/codon_start=1

/transl_table=11

/product="capsular polysaccharide phosphotransferase"

/protein_id="AVY03737.1"

/translation="MNFILILFIIIHKFINQKWLNIVQKLKKLIKKPGIFLRDYLNKK

YPIKNIEQPYSELEEHILIEADEKLNHISARNSVIPFDIDVVFTWVDGSDLNWIGKFN

KFSPEYKERSALYATDSARFENHNELFYSVNSVLKNIPWVRHIFIVTDEQRPEWLNEC

YKHKVSVINHVDIIDKKYLPTFNSHVIESFLYKIPDLSENFIYFNDDVFVARPLEPEH

FFQHNGIASIFLADKSLLRMKDKGIITPTLSASEKCIKLLFRDYNTKIDSPLVHTYIP

LKKSIYELAWSRYKHEIEEFLPNRFRNNNDINFANFLIPWLMYLEGKAIPKRDICYYF

NIRSPHAITQYRKLLQKKKNGISPHSFCANDFNSKRSISGYHQKLINMLHLYYKDNDN

V"

CDS 11251..11364

/gene="hypothetical"

/codon_start=1

/transl_table=11

/product="putative small periplasmic lipoprotein"

/protein_id="AVY03738.1"

/translation="MKKLLLVALVASFGLAACGVKGPLYFPEQQPAQQQTK"

CDS 11487..12092

/gene="lysA"

/codon_start=1

/transl_table=11

/product="diaminopimelate decarboxylase"

/protein_id="AVY03739.1"

/translation="MNHFNYKNQQLFAEDVSVSDIINQHGTPAYIYSRATLERHWHAF

DKAFGAHPHLICFAVKSNSNIALLNVMARLGSGFDIVSQGELERVLAAGGEPSKVVFS

GVAKSHSEIQRALEVGIRCFNIESIAELHRINEVAGQLGKIAPISLRVNPDVDAHTHP

YISTGLKENKFGVSVTNEGNKYWEKRYEKKYRRKTYCVKVR"

CDS complement(12315..12857)

/gene="ydeN"

/codon_start=1

/transl_table=11

/product="putative hydrolase YdeN"

/protein_id="AVY03740.1"

/translation="MKKVYVTHGYTANPTRNWFPWLKNELEKLGWECECLAMPNSDQP

NPQAWLEHHQNTLQLDENTLLIGHSLGCIALLNYLAVTQQKVKTAIFVSGFYEKLPTL

PELDSFADFYANQTAYLPQKSYVISALNDTVVPHSFSDRLAQYLQADYIRLATGGHFI

DREGVTELPVLLELLKQILK"

ORIGIN

1 atgccaaata tcaacatcca gaacgcctta ttttcccttg ctcaacacaa taaactctcg

61 attgaatcac tggaaatcaa tactcacgat ttctgggtga ttgtcggcgg taacggctcg

121 ggcaaaaccg ctttcgccca agcgctacat aattcacttt cgttatattc gggtgaatat

181 caaaatagtt tccagcatat cgctttactt tccttcgagc agcaacaaaa aatcatcgag

241 caaatcttta aacaccgtaa caacgatatg gtttcaccgg atgatttcgg tttaaccgcc

301 cgtcaaatta tcttaaacgg tagcgaaaga acgcaattat gcgaggaata tgcggctaaa

361 ttacgtatcc agccgttatt agatcgcccg tttattcagc tctccaccgg cgaaagccgc

421 aaagtgttat tttgccaaat gttagtcagc gaaccggatt tattgatttt agatgagcct

481 tttgaagggt tagaccaagc ctcggtcact tattggcagg aagtgatggc acaactcggt

541 aagcaaatgg cggtggtact gatttccaac cgttttaatg atattcccga ctgtgccaca

601 catattgctt tactggataa cttacaactg attttacaag gcgaacgtca agagattgaa

661 caacaagcgg tctattctca gctaaaattt gcagaacaga atgtgaatgc accgttgctg

721 gagagtgcca caccgctgat tcaactccca ccgaatacta atccgtttga actgaaaaac

781 gtaatgatcc gttacggcga aaaaacgatt attgatgatc taacttggac ggttgcccca

841 aaacaacatt ggtggattaa aggcccgaac ggagcaggaa aatcgacctt actttctatt

901 attgccggcg atcatccgca atcttacgct aattatgtgc atttattcgg tcgtcagcgt

961 ggttcgggcg aaacgatttg ggatataaag aaaaatatcg gctatgtgag cagccaatta

1021 catatggatt atcgggtgaa ttgctctgcg ttagacgtga ttttatccgg cttttttgat

1081 tcaatcggcg tttatcaaca agtaccgagt gccttacagc taaaagcaat ggaatggctg

1141 gaacgcttgc atttagccaa tctggcgaaa aaaccgttcc gttcactttc gtgggggcaa

1201 caacggttat tattgattac tcgtgctatg gtaaaacacc cgccgattct gattttagac

1261 gaaccgctgc aaggtttgga cggtgtaaac cgcaaattgg ttaaacaatt tatcgaacag

1321 cttgtgacta atagtcaaac ccagttgcta tttgtttcgc accaagatgc ggacgcccca

1381 aattgtatca cccatttatt tgaatttgtt ccgcaaacta acggtggtta ccgttatgtg

1441 cagacggcgt taaattaggt ttttgacctt taaaggaaat ccccttcttt agtaaagagg

1501 gattagggga gatttgtcaa tagagagata tgaaattgaa tagaacttca ttttttatat

1561 ttataaaagc gttaattagc atatttcttc gctaattcat tctgtcaaat ctcctcctgc

1621 ccctctttgc taaagagggg agatatgtgc gggactttaa ggcgttgaat tatagaactt

1681 ataagcatcg tccatatttt caaatttatg cataatgcct ttatcaagca ccatcgcatt

1741 atcacaatat tccttcattg ctgacggact gtgcgacact aaaatgatcg aacgatcttt

1801 gcgtttctcg aataattcgt acttacattt tgccgcaaag cgagagtcac ctaccgcaat

1861 tacctcatca attaagtagc aatcaaactc aactgaaagc gataaagcaa acgcaagtcg

1921 ggctttcata ccggaagaat atttcttcac cggctcatat aaataatcgc ctaattcaga

1981 aaattcctcg gtaaacgctt taacatactc aaaatcggcg ttataaattc ggcaaataaa

2041 gcgtaaatta tccataccgg ttaaactgcc ttgaaatgca ccgctgaaag cgagcggcca

2101 agaaattgac atatggcgct caatcttacc ggttgttggc ggctcaacac cgctcattaa

2161 gcgaattaag gttgacttac ctgcaccgtt acgccccaga ataccgattt tttcgccttt

2221 atgcaggtca aaattgatat tctgtaacac ggttttccaa ccgctattag tatgatagcg

2281 cttgctcaca tcttttacac tgatcattgc ggttcaattc ctttactgaa gtttcgcacg

2341 agaatcagcc ccaataacag cattgccaaa tcgcatacta ctaaaaaacc gatattttca

2401 tacgttggga ctaaatcgcc aaaatagccg tgacgaaaca tttccgtacc actaatcatc

2461 ggcacccatt gggcaatttc acgaatttgt gatggtagtg aatgtacgaa gaaaaatgca

2521 ccagacagcg gtaataatac aaaacttaac gtgttccaaa ttttgccgaa cacctcaatc

2581 ttttgtgcta acgaacaaat aatcagacct aacccaaagg caaaaaatgc cattaaagtc

2641 caagccagca ccatgtaaaa cacatctttc ggcggatcta tccagccgat aaaaactaaa

2701 accgccgtta taataatctg ggcaacagtc gcaccggcaa cttccaataa catacgagca

2761 aaaatcgtat ctaatacccg tacgttacgg tgatatagaa ggcttaagtt agcggaaatt

2821 gagccaattg ctcgatttga tacatttcgc cacatcattg ccatcggata gccggtaatc

2881 gtaaacgcta caatatttaa cgtagaaacc ttatccgctc ggaaaaactt ccacattaac

2941 acaatcaaaa acgttaacag caacggctca ataaacaacc ataaaaagcc taagttctta

3001 cgcccatagc gggtaataat ctcccgcatc aacagcgcat aaatcacacg cccctgaatg

3061 gctaacgatt gtcgaaacgt tgtttggtca ccatattgca ttagttttta tgctctctca

3121 cacttgtgat taataaaccg aaaattccat ataacattaa accgataata aaagtcgcaa

3181 taatgttata taaacggctt ggtgccaaag cccagtccgg tttacttggt tgactgataa

3241 cttctaaata aagttgctga cggtcggctt cgcctcgagt gttatgtaat gatgccattg

3301 ctgcggtcag ttgttgctgc gctaattcat tagctaacac taaacgctga taatccgccg

3361 attgattcgc aaccgagcta cccgtattac ccgaaagctg ttttgactgc tcatcaattt

3421 ctttacgtaa acttttttgg cgcatcagta gtgcatcaac ctgagggtta tccggtgtaa

3481 ttgactgcaa ctgcgccaat tgcgtttcca cacgaatcag ttcacttttc agactcgaaa

3541 ttaaagaaag ctgtacgccc gattgtgccg gcagatcaaa aattttattt ttgatacgat

3601 atttacttaa cgcttccgca gtttcattaa catttttttc cgcttcttgc accgcttgag

3661 cagcaaactc gagcgtatct ttacgacctc gagcatttaa acggttaatc aagtcttcac

3721 ccagtcttaa caatttctca tttaactgct gcccctcact cgcttcaaac gcttgtacac

3781 gtaaagtcgc aatccctgat acggaatcca catcaatgct taaacgatct ttaaaatagc

3841 ggaaaaacgc ttcttgcgta tcatttaaac cgaaaccgtt aaaacggctc agaatatcgc

3901 cttttgttgc atagaaatca cgaaccggta attcggtttg taactgttct aatgccgtac

3961 gagaacgcat atattcttgc actgaatacg tgtcgtcttg cgaacgagta aagcctgttc

4021 cttgtaacaa cgctcccacg cccgaaagcg aagactgatt acgtggcgaa cgcaccacaa

4081 aacttgattc ggaaacataa atatccgaag caaacatccc aaaataaatg gctgaaaata

4141 atgttggaaa aataaccgtt aaccaaagca atggatttaa ctttcttaat aagctttttt

4201 tcttaggttt attcggtttt tctgcggtaa tcgctaccat tatcctttcc tctaaataga

4261 tttatcgtta taaggtctat tgaccgcaat taatagctac gaatactgtt agtagtactc

4321 gtaatcgggg atgttactga gaaaatcatt ctcaagaatt tttggaattc agacaacggt

4381 gcatttgata cataaacaat atctttatct tgcatcggga agcgttgtaa taaaaatagt

4441 gattgcggct caagtaaatt cacacgataa accgtcggta catccatacc cataccatag

4501 cctttcgatc cccattgcgc ttgctgatct aaactcaatt gagaaaaagg aacatgacgg

4561 aagacgaata cccctctcgg atcggaacgg gtatcgatta agcctcccat cttaccgata

4621 gcttcggcaa gcgtaatacc tttacttgag aatttcattt gctggttgtt acccaccgca

4681 cctaaaccgg taaaactata aggtgtgttt agcagtgaaa caacatcgcc agcacgtaac

4741 ataatatttt gtgccggatc ggaaattaac gtttcgaatg cgagtgtttt tacttcagaa

4801 ccacgggtta gcttgaccgt cacatcttca atgttttccg ttgttccgcc tactgcagca

4861 accgcatcta atacacgttc attattagcg gttaatggca tacgaatact attgccttga

4921 cgaataaccg taacatcagc agagttatta ttcgcaattt tgactaatgc ttgcggatga

4981 ttcgctttgc gctgtagtgc tccaataatt tgagactgaa tcgcttccgg tgttttgcct

5041 gcgacacgaa tgttacccac gaacggcacg gtaaccgtac cgttttgatt aaccatttgt

5101 gccggtaatt gcgttaaatg cccgctacct tgtccctcag aactaaaagt accgccaaac

5161 agcaccgctg gcggagcttc ccaaattgat atttcaagta catcacccac attgaccgca

5221 ccggcatatc ccgcaccgcc tacagtacct aaaaaaccgg aaaattgctg actttgctgc

5281 gtttggtaca actgttgcac caatccatta tctaattcca ccaaattcac ttccggcaac

5341 ggtttatctg aatttttgga attagcctct aaaatcgcac tatggctagg acctgaagtc

5401 gggaggattg agcaggctgt gattaataaa gcagctaaag ttaaccctat gattgaatta

5461 tgttttttttg attgcattat ttccactcag atacatagtt gcacttttca tacttcatga

5521 aaagtgcaac ttataattta gctaccacta gccttttaac caagataaat caacttcatt

5581 aaaactctgg aatagttcca tatcttcact aagaggggtt ttataagaac ttctatctct

5641 taattcaata ggttcaaaat taccaaccct ccaatgtaag cgaaggattc caaaatgcct

5701 tcctaacagt tcctctgaaa atttactttt atctccctct actttatgaa ctagcaaatg

5761 gaccttttta gaaagttttt taggtttaaa tgcatatttc ggagcatata atattctttc

5821 tttttcatat aaatgagtat ggttatgcat ccttggtaaa ttagtaaatt ccgtactagc

5881 aacttcaata atctgacgac gtttatatgt aataacaggc tcgtcaatac tatctaaaat

5941 atcaggaagt gataacccat ctttatgtat aactaattca tccacatcac caataactac

6001 acaatttgct gaatatacaa accgcttcag tgcatgttcc catgattgat attgtccgaa

6061 atcggaatcc caaatctgct tatctccccc cgtcacaccg taaggaatag accatggaac

6121 tacacatatt ttctctaacc ctttaatgga agaaagaaaa gtttttaact ggtttatatc

6181 atataaagaa ctgtcgttat catatatgag aacggcatta atcttatgat gcttaatatg

6241 gtgagcaatc caataaccaa tccattctaa agggttatct ttttgctgtg taaccaaaac

6301 attatgatta gcaaaaaaat cagattcatc aggttggatt tcctgttcaa ataaaacatc

6361 ctcaaatttt ataataactt tctttgcacc tggtattttt ggtaataata ctctacattt

6421 tctatgctct gattggataa tagcttcttt agtaacattt ttctcatcaa tatatatttc

6481 tccagacaat aaaatattat ggagatttaa caatggcggt cctaccaatt caatattttc

6541 ctcagaaaag aaggcatccg aaaagagggt atgataatca aacagctctt tataatttgg

6601 ttgttgatct tcctctttcc taggagggac tctcaatgta ttcttttctg ttagctctat

6661 tttactaatt ttaacatcta aatttctcat attttttcct aatttaaact tatttatgat

6721 tcatgattcg ttgataaatt cgctcacagg atcttccatc tttataggca aatacatcca

6781 ttctgcttgt gtatttcttc tccatttgtt gtggtgattt taaataactt tcaagttgtt

6841 taagcaaatc tactttctgt tccgctaccg gaccaaatcc atcattataa aaatcaaaat

6901 atcctttctt ataatgttta gaataaaatt cactttgatc aaattgataa taaaaaacac

6961 atttgtttaa aaagcccatt tcaaaagcta cgctagagta gtcagttatc atacaagatg

7021 attttaagaa tagttcttgc atactttcat tattagatgg attccatatt gatatatatt

7081 gattttcaac aaataaatgg gaatatttat ttagttcttg atgaggtgca aaaactattt

7141 tatatccata tttattaagt aaactattaa gtttttctga agataataac tctacccaat

7201 ttcgtaaata ctctgaatca gagatgttat cttgatttaa ccatgttctc caagttggca

7261 taacaagaat tattttttct tttttgacaa cttttgattt tttaattaaa gaatcatgtc

7321 ttggtaagcc acttaataat acttgactag gtaataaatt gtaatcagta aaattcgcag

7381 cgatagaatt gtattctggt tttgttgatg taaccatgaa atccacgtgt ttagtgttta

7441 caacatttgc attattatta cagataacac catgctgtaa ccatatcttt ttcttagaag

7501 ctaaagatag aagtccttta gatactaagt gattccaaat gaataaatgg gaactaacga

7561 tatatgtaca attttttaac tctcgagaaa aatagctact accataatct aaaagattaa

7621 atccatcacg ttctaaacgt ttccagtcag aacaattttt acttatagcg taaaaaaatat

7681 tttgttcagg atgttttttt tgtaggtaac gatagaaatg ttcagcatta tcatcccctc

7741 tatcttttct atccataaga atccaagtat cttttaagtt aggatagatt atattttttat

7801 tcatagaact aataagctca gtaataactg caccaccggt atacccttta gtaaaacatg

7861 ttaaatttac ctttctatta tttatagata ttgcaagatt ttgatttgtg tcttcatatt

7921 taaataaaaa acgcctctca atggaaaatt tatcacccat aaaatcattt aaaatgaact

7981 tctcttcagt tatttctata tttaaatcat ctaatgtaac ttttatcgta tcatccgtat

8041 taaatgagaa atagcttata caaaattctt ttctttcttt atctatatag ttcacataag

8101 tataatgaat aggcaatgcc tgacctttat atagccctaa tatcccaact tttgtttttc

8161 tatctaaatt cgagaactca cattttataa tagtatccac atcaataaat ttgaaaattt

8221 ctttaagaaa atctttaaaa atttgtcttt cttgaattgt aagaaagttc acatgatagt

8281 ctgcgttaac taatcttctg taatatccta tacaatggaa taaggctact ctttgaatat

8341 gtattggaac atatcctaaa gttttattag ctagctctaa tatattaagc acgccatttt

8401 tgggaacatt agtaaaaagc ccaacttttt tccaagaatt attcattaaa gaagtattat

8461 tctctctctg tctatataaa tatcttgctt ctttaacaaa accaacttgt acatcaggac

8521 taacaataag caattgacta ttaaatttac aatcttcaaa agacggcttc acattttcat

8581 caaaccgtac ccccaaatta tataaaaatt gcaagttata tatactagtt gcagcagata

8641 atagaagatc attgccaaaa tcattttgca cagtatgcgt ttttacatac ctaaacctaa

8701 gaggatgaat atctctaaag gtattattat gttcataaaa ataaatcaaa ttacttgatg

8761 ctattttaca attcttattt ttgataaaaa agttatcaag agacttaaaa aaatctttat

8821 ctaaaatatc atctggatct gtaaatgtta cgtagtcaaa atatatatta ttatcttcta

8881 agtaatctaa accataattc cttgcactag ataacccacc atttgtttta tataaatatg

8941 taacattacg agggtacttt tgtttgaact tttcaatttt ttttgctgat aagtctgtag

9001 aaccatcatc tacaaaaata acatggatat gtttagtgaa atccaaagtt tgattttta

9061 tactttgaaa aaaatcatct atatattcat ccacattaaa tgtagggcaa ataatacaat

9121 attggtatgt accatatgat gtttttcgt ttctttgttt ttttaatcta aagttccagt

9181 aagcatcttt aaaaaataag ataggatctt ttctcagctt attaaatttt cttttaaaca

9241 ttatcattat ccttataata taaatgaagc atatttatta gtttttggtg ataaccacta

9301 atacttctct tgctattaaa atcattcgca caaaacgaat gtggtgaaat tccatttttc

9361 ttcttttgta ataattttcg atactgtgtt atcgcatgtg gagaacgaat attaaagtaa

9421 tagcatatat ctcttttagg tatagccttt ccttctaaat acattaacca cggtataaga

9481 aaattggcaa aattaatatc attattattt ctaaatctgt ttggtaaaaa ctcctctatt

9541 tcatgtttat aacggctcca agctagttca tatatacttt ttttcaatgg aatatatgta

9601 tgtactagtg gagaatctat ttttgtatta taatctctga ataataactt tatgcatttc

9661 tcagatgctg ataaggtagg agttattatt cctttatctt tcattcttaa taagctttta

9721 tcagctaaga aaatagatgc tataccatta tgttgaaaaa aatgctcagg ttccagtggt

9781 ctagctacaa agacatcatc attaaaataa ataaaatttt cacttaaatc gggtatctta

9841 tataaaaatg attcaataac atgagagtta aatgtaggta aatatttttt atcgataata

9901 tccacatggt taattacact tactttatgt ttataacatt catttaacca ttctggtctt

9961 tgttcatctg ttacaataaa gatatgtctt acccatggta tattcttcaa tacactattt

10021 actgaataaa ataactcatt atgattctca aaccgtgctg aatctgtagc atatagcgcc

10081 gatcgttctt tatattcagg agaaaacttg ttaaactttc ctatccaatt aagatcacta

10141 ccatcaaccc acgtaaaaac aacatcaata tcaaaaggaa ttacagaatt tctagcactg

10201 atatgattta atttctcatc agcttctata agaatatgtt cttctaattc tgaataaggc

10261 tgttcaatgt tctttattgg gtatttttta tttaaatagt cacgcaaaaa aatacctggt

10321 ttttttatga gcttttttaa tttttgcaca atatttaacc atttttggtt tataaattta

10381 tggattataa tgaataaaat taatataaaa ttcatttact gtaatttata tgtaatatat

10441 tattaagaag agtaaattaa tgtataatag catattaaga aagaatagta aagaaccctc

10501 tctaaatata catttaaagc tatgaattta gagttagaaa gcaaactgta tccatttttt

10561 catcgcaagt gcatctaatt acttagatgt aggagaactt attaacgcat cctcagtaaa

10621 agtttgtttc aaaatgaggc tgaaaacctt atagactaaa cattaaaggt tttcacgatt

10681 gattagtagg tttattagat atagaaaaat ctcttatagc ctaaagattc tttatatcga

10741 aagtcaaaat aaaagcggtc aaatttttct taaaacttgc aaagtttagg aaaaatttga

10801 ccgcttattt tattcatact cttatataaa cattagagtt tattatgggc gtagccacat

10861 ataattgata aagatatgcg gtatagcaat attaatacaa tcctatcaat ttcatattat

10921 cttaatcaaa tttatttttt cgttttttaa atattaaact attatttact tctgcaaatc

10981 tcccctatcc cctctttact aaagaggggg atttccttta gatataaaca agctatgatc

11041 gttcgaatag ctttggtctc tcaatagatt caggtaaatg gtcgatatat aaggaaaata

11101 aaaacattcg ttaaagacga gcgccatctt agagaaattt agagccacag cgtggctcac

11161 tcagccgtag gctgatcgta accacgtacg gcttgccgtg cgtggctatt gggttagaat

11221 caacggacga tttactctca aggatttatg atgaaaaaat tacttttagt ggctttggtt

11281 gcctcgttcg ggttagcggc ctgcggtgta aaaggcccgc tttattttcc tgagcagcaa

11341 ccggctcaac agcaaacaaa ataatttcta accacggcaa ttgagaagca cctgtgtaac

11401 aagcggttaa atttatccga ttttttgcaa atacgaatgt agctattttg ccgtggtttt

11461 atttttcaaa cacaacggac aaattaatga atcatttcaa ttataaaaac caacaacttt

11521 ttgcggaaga cgtttccgtt tcagatatca tcaatcaaca cggtacgccc gcttatatct

11581 attctcgtgc tacgcttgag cgtcattggc acgcttttga taaagcattc ggcgcacacc

11641 cgcacttgat ttgttttgcg gtgaaatcta actccaatat cgcgctatta aatgtaatgg

11701 cacgcctggg ttcgggcttt gatattgtat cgcaaggtga acttgaacgt gtgcttgctg

11761 caggtggtga gccgagcaaa gtggtatttt ccggtgtggc aaaatcacac agtgaaattc

11821 agcgtgcgtt agaagttggc attcgttgct ttaatatcga atcaatcgcc gagctacacc

11881 gtattaatga agtcgccggt caattaggta aaatcgcacc gatttcattg cgtgtaaatc

11941 cggatgtgga tgcgcacact cacccttata tttccaccgg tttaaaagaa aataaatttg

12001 gggtaagcgt aacgaacgag ggaaacaagt actgggagaa gcgttacgag aaaaaataca

12061 ggcggaaaac ctactgcgtg aaagtgcgct aaattggacg attctccgcc cgtgcgggct

12121 gaatacggac gaaggcgaaa cctttcgttt aattgaaaat gcggctgaac tgcccagcag

12181 ttatatgagc cgtaaagcat tagccaatgc ggtcttgtcc gtacttaaca gtgaaaacac

12241 aaaccataaa atcttctcag tctgtgccta acttcacaat ccctttcact ttggcacaag

12301 ccatccgctt gtgcttattt caaaatttgt tttaacagtt ccaataaaac cggtaattcc

12361 gttactccct ctcgatcgat aaaatgcccg cctgttgcca agcgaatata atccgcttgt

12421 aggtattgtg ctaatcgatc actgaacgaa tggggtacaa ccgtatcatt taatgcggat

12481 atgacgtaag acttttgcgg taaataagcg gtctgatttg cataaaaatc tgcaaagcta

12541 tctaattccg gcaaagttgg taatttctca taaaagccgg aaacaaaaat tgccgttttt

12601 actttttgct gcgttaccgc cagataattc agtaacgcaa tgcagcccaa actatgtccg

12661 atgagtaagg tattttcatc taattgaagt gtattttggt gatgttccag ccatgcttgg

12721 ggattcggct gatcggaatt cggcatcgct aaacattcac attcccatcc taatttttcc

12781 aattcgtttt taagccacgg aaaccaattt cttgtcgggt tcgccgtata accgtgcgtt

12841 acatatactt ttttcat

//

LOCUS **MG868950.1** 15201 bp DNA linear BCT 16-MAR-2021

DEFINITION Actinobacillus pleuropneumoniae strain A-85/14 capsular
polysaccharide gene locus, complete sequence.

ACCESSION MG868950

VERSION MG868950.1

KEYWORDS .

SOURCE Actinobacillus pleuropneumoniae

ORGANISM Actinobacillus pleuropneumoniae
Bacteria; Proteobacteria; Gammaproteobacteria; Pasteurellales;
Pasteurellaceae; Actinobacillus.

REFERENCE 1 (bases 1 to 15201)

AUTHORS Bosse,J.T., Li,Y., Fernandez Crespo,R., Lacouture,S.,
Gottschalk,M., Sarkozi,R., Fodor,L., Casas Amoribieta,M., Angen,O.,
Nedbalcova,K., Holden,M.T., Maskell,D.J., Tucker,A.W., Wren,B.W.,
Rycroft,A.N. and Langford,P.R.

CONSRTM BRaDP1T consortium

TITLE Comparative sequence analysis of the capsular polysaccharide loci
of Actinobacillus pleuropneumoniae serovars 1-18, and development
of two multiplex PCRs for comprehensive capsule typing

JOURNAL Vet. Microbiol. 220, 83-89 (2018)

PUBMED 29885806

REFERENCE 2 (bases 1 to 15201)

AUTHORS Bosse,J.T., Li,Y., Fernandez Crespo,R., Lacouture,S.,
Gottschalk,M., Sarkozi,R., Fodor,L., Angen,O., Nedbalcova,K.,
Holden,M.T.G., Maskell,D.J., Tucker,A.W., Wren,B.W., Rycroft,A.N.
and Langford,P.R.

TITLE Direct Submission

JOURNAL Submitted (26-JAN-2018) Medicine, Imperial College London, Norfolk
Place, London W2 1PG, UK

FEATURES Location/Qualifiers

source 1..15201

/organism="Actinobacillus pleuropneumoniae"

/mol_type="genomic DNA"

/strain="A-85/14"

/serovar="16"

/note="K locus: KL16"

/isolation_source="lung"

/host="pig"

/db_xref="taxon:715"

/country="Hungary"

CDS 1..1473

/gene="modF"

/codon_start=1

/transl_table=11

/product="putative molybdate ABC transporter ATP-binding protein ModF"

/protein_id="AVY03741.1"

/translation="MPNINIHNALFSLAQHNKLSIESLEINTHDFWVIVGGNGSGKTA
FAQALHNSLSLYSGEYQNSFQHIALLSFEQQQKIIEQIFKHRNNDMISPDDFGLTARQ
IILNGSEKTQLCEEYAAKLRIQPLLDRPFIQLSTGESRKVLFCQMLVSEPDLLILDEP
FEGLDQASVAYWQDVMAQLGKQMAVVLISNRFNDIPDCATHIALLDNLQLILQGERQE
IEQQAVYSQLKFAEQNVNAPLPDSAAPLIQLPPNTNPFELKNVMIRYGEKNIIDDLTW
TVAPKQHWWIKGPNGAGKSTLLSIITGDHPQAYANYVHLFGRQRGSGETIWDIKKNIG
YVSSQLHMDYRVNCSALDVILSGFFDSIGVYQQVPSALQLKVMEWLERLHLASLAKKP
FRSLSWGQQRLLLITRAMVKHPPILILDEPLQGLDGVNRKLVKQFIEQLVTNSQTQLL
FVSHQDADAPNCITHLFEFVPQENGGYRYVQTALNQIENA"

CDS complement(1823..2473)

/gene="cpxA"

/codon_start=1

/transl_table=11

/product="capsular polysaccharide export protein CpxA"

/protein_id="AVY03742.1"

/translation="MISVKNVSKDYYTRSGKKTVLQDINFELKKGEKIGILGRNGAGK
STLIRLLSGVEPPTSGTIERNMSISWPLAFSGAFQGSLTGMDNLRFICRIYNADIDYV
KAFTEEFSELGDYLYEPVKKYSSGMKARLAFALSLSVEFDCYLIDEVIAVGDSRFAAK
CKHELFEKRKDRSIILVSHSPSAMKSYCDNAMVLDKGIMYKFENMDEAYKFYNSTL"

CDS complement(2470..3267)

/gene="cpxB"

/codon_start=1

/transl_table=11

/product="capsular polysaccharide export protein CpxB"

/protein_id="AVY03743.1"

/translation="MQYGDQTTFRQSLAIQGRVIGALLMREIITRYGRKNLGFLWLFV

EPLLLTLFIVLMWKFIRADRVSDLNIIAFVITGYPMAMMWRNASNRTIGAISGNLSLL

YHRNVRVLDTLLARVILEVAGATIAQIIIMALVILLGWIEMPKDTFYMVMAWVLMAFF

ALGLGLIICSIAQKFEAFGKIWGTLSFVLLPLSGAFFFVHALPSQAQQYATLIPMIHG

TEMFRHGYFGDSVITYESISYLVICDVAMLLFGLIMVKNFSKGIEPQ"

CDS complement(3267..4424)

/gene="cpxC"

/codon_start=1

/transl_table=11

/product="capsular polysaccharide export inner membrane

protein CpxC"

/protein_id="AVY03744.1"

/translation="METTITASPTEKLQKPVKQKKSWLKKLNPLFWVTVAIPTVLSAF

YFGSVASDIYISESSFVVRSPQNQTALTGVGALLQGSGFSRAQDDTYTVQEYMHSRTA

LEQLMKDLPIREYYENQGDIIARFNGFGLNNSKEAFYKYFRDRLSVDFDSVSGIASLR

IRAFNAEEGQQINQKLLAEGETLINRLNERARKDTISFAEQAVTEAENNVNETANALS

KYRIKNKIFDLPAQSGVQLSLISSLKSELIRVETQLAQLQSITPDNPQVDALLMRQKS

LHKEIDEQSKQLSSNSNSSIAIQTADYQRLVLANELAQQQLTAALTSLQNTKNEADRQ

QLYLEVISQPSKPDWAEEPYRLYNILATFFIGLMLYGVLSLLIASVREHKN"

CDS complement(4450..5610)

/gene="cpxD"

/codon_start=1

/transl_table=11

/product="capsular polysaccharide export protein CpxD"

/protein_id="AVY03745.1"

/translation="MKLQIYTLHLCILLSGCSSLPTTGPSLNEVAEINKNGQFEANLI

ELNIAIVNNLYHIQEKQNFSGFLGTAGGAGYAGAVNVGDVLEISIWEAPPAVLFGGTF

SSEGQGSGHLTQLPAQMVNQNGTVTVPFVGNIRVAGKTPEAIQSQIIGALQRKANQPQ

ALVKIANNNSADVTVIRQGNSIRMPLTANNERVLDAVAAVGGTTENIEDVTVKLTRGS

EVKTLAFETLISDPAQNIMLRAGDVVSLLNTPYSFTGLGAVGNNQQMKFSSKGLTLAE

AIGKMGGLIDTRSDPRGVFVFRHVPFSQLSLDQQTQWGAKGYGMGMDVPTVYRVNLLE

PQSLFLLQRFPMQDKDIVYVSNAPLSEFQKFLRMIFSITSPVTSTTNAVRAY"

CDS 5747..7147

/gene="cps16A"

/codon_start=1

/transl_table=11

/product="capsular polysaccharide biosynthesis protein

Cps16A"

/protein_id="AVY03746.1"

/translation="MLLFKYLNYIRYFLKIREKIKNKEFIYVLAKYKDFLPLNLKLFL

LYKTGLYNKIIENIDYKKYIYYDISKVALGYKFSYESNNAFLSKYRNNISFILGILPY

NPTLALDCLCHKKYYLLKLFILYHLGQKIDFVPNKINHPDLYLLCNNDSVNSNIENIN

KYLDYYNLSKIRFINKEANNFINNIITSQFSSPAQIDDLPLVSILMTTFNSEKWIEWS

INSLLSQTYSNIEIIIIDDSSTDSTISIIEKLKRKSDKIKLIKLSKNVGTYVAKNIGI

SLAKGEFITCQDSDDWAHPQKIMLQIYPLLENPNLVVTFSQWFRIYNDGKAYARGVFP

LTRLNPSSALFRKDIVIEKIGLWDCVRTGADSEFNARLKLVFGNELCLTIKKPLTIGT

HRKDSLMTSSDTGYINGISLERLNYWESWNLWHISEIEKGRVPYYLLNNKRPFNISDK

NKVDYKDIICNLCSIK"

CDS 7187..8884

/gene="cps16B"

/codon_start=1

/transl_table=11

/product="capsular polysaccharide biosynthesis protein

Cps16B"

/protein_id="AVY03747.1"

/translation="MKKIIIIGHSTSGYQSVEELFQVAGMSPALPSKRDGMTPQEIDS

VLKKVIFSPSHSSKELLPAKRYQSKRQAKKIAQKFDVVQSSIVPAETLINPIWDHLAL

DLMLGNLDQSFWGWSNPNALDTLEYWQRIDPEIYFVFVYDNPHSVLLQYTEEEILLLD

DKKITEKLDTWAKYNKKMLNAFEKFQDRSVLISSKQLIEFSENSIRTVYDQIQAPIDL

ESRELTLDLVRNNISSNNKYINTFLVECIINNYSDMILIYENLQESADLPYLSKMDIK

NGMVLQSWKEIIKDKIFFQSQIKEKNKLLSQYKEEYSIREKFESANKVILAQLHLTQD

LLEKELLIKQEMEKNSSIYRQRLDVYESDKIDLTSKLRSLENDKKELQSKLNNLENDN

KNLQSILEHVENSYNNVSNNVENIRAQLTLTETENQNLILQLHLTQDELEKQYSVISS

LQKPIYLGAADRIKSEMPYRLGKKMIEASKSLKGWLTMPCLLKKEANKVKEEQEKSEG

LKYINIEEYADFSDAEKVKKHLSYKLGVKLLKNIRKPYLWIFIPFSLLSTIYLFRKN"

CDS 8970..11006

/gene="cps16C"

/codon_start=1

/transl_table=11

/product="capsular polysaccharide biosynthesis protein

Cps16C"

/protein_id="AVY03748.1"

/translation="MSLKLANQLFQEGKYQQALVEYQKVKSDHPLYQHAQFNINLIHV

KLHQDIIPTNSISKLESNEQPLVSVVMPVFNVAPYLDASIMSVLNQSYTNIELIIVND

ASTDNGLNIINMYKNWDSRIKVVNLEFNTMGGAGIPSNIGVDNARGEYLAYADSDDIL

DKYAIQKMMESALKHEAEVIIADFSNFNNEIRVIENGYDKKNWNNIPLDEVFSPKDKS

EIFRLSPVPWRKLYKVSFLNNNKIRFPEGDYFYEDNPLHWFVLTKAKRVVLLDYVVAY

HRMEREGQTMGAMNFKLSAQFCHLNSIKNHLLKMKDVPRIYWKELVDFAYRGGWVVDR
QDIPEFQSIVKKRYAQTALGIEKLSHIPKDEIRKMRPNFYKRCEEYNQAYADLDLSIV
VPVYNCVDLLPQLMESLLKVNLKTDIFLIDDGSKDGSRELCEKYAKQYKNVYCIAQAN
KGAGVARNLVIPLLTGEYSYFVDADDFIDPKSLEESVKFAQKNNHDLVLFKYKIEFHE
KGNTRDMWDADKKIWAKLLVAKNNNERKILASQLINYPWNRAVKTSLLHDENIFFGKT
VVHNDVPYHWHTVVSANNIGIYDKPVCTHRKFEERQQITNISDYRRLMVLEAYRHTHE
LLKRYDSYALIFPHWQKFIRDLLTWARDRVPEDKLEFYKERHKQILDELKGVNL"

CDS 11003..12106

/gene="cps16D"

/codon_start=1

/transl_table=11

/product="capsular polysaccharide biosynthesis protein
Cps16D"

/protein_id="AVY03749.1"

/translation="MKNIAMAGAGLSCAVIARELAEQGYKVTIFEGRNHIAGNCHTQR
DENTGVMVHVYGPHIFHTDNENVWNYVNKYMEFKPYVNRVKSTILGKVYSLPINLHTI
NQFFGKNLSPKEAKAFIEQQADMSITDPQSFEEQAMRFVGKDLYEAFFKGYTKKQWGL
EPSSLPASILKRLPVRFNYDDNYFAHRFQGMPAEGYTKMVENILNHSNITVHLNTLFK
REQVQHYDHVFYSGTIDGYFNYEFGRLPYRTLDFKKYYDEGDFQGCAVMNYGDENIPY
TRISEHKHFANWEDHEKTVYFEEYSRQCTPNDIPYYPINLVEGNELITKYKEKAKREQ
KVTFVGRLGTFRYLDMDVIIKEALDIARGFYVK"

CDS 12096..12776

/gene="cps16E"

/codon_start=1

/transl_table=11

/product="capsular polysaccharide biosynthesis protein
Cps16E"

/protein_id="AVY03750.1"

/translation="MLSDGIRNQIKIGENIFRLICKDGRVIDNPEINGLQVIFQGRMG
GTVEIEEDAVFHNTKIYAGGAGFIRINKTHPKGIKNVTIKTVCPCPYKYLLIDEGCSI
EQAVFMLVNDEDLVVKIGKDCMLSSGIFFRAADGHTIFDVNTKNVMNYSSPIIIGDHV
WIGANSTFLKGAEVASNSIVGTHSLVTKKFSKQFCAIAGIPANVVREGINWDRLRITE
YKQHLSVK"

CDS 12818..13987

/gene="cps16F"

/codon_start=1

/transl_table=11

/product="capsular polysaccharide biosynthesis protein
Cps16F"

/protein_id="AVY03751.1"

/translation="MNITIIGTGYVGLSNAVLLAQKHNVTALDIDKEKVAKINAKQAP

IEDNEIEYFLANKKLSLLATVDRYLALENAEFVIIATPTNYDPKTNYFDTSSVESVIQ

QVLEINPNAIMVIKSTIPVGFTEQVKQKYKTENIIFSPEFLREGKALYDNLYPSRIIV

GEKSERAKTFANLLLEGAIKKDVDILFTDNTEAEAIKLFANTYLAMRVAYFNELDTYA

SIRGLNTEDIIKGVCLDPRIGDFYNNPSFGYGGYCLPKDAKQLLANYQDVPQNLINAI

VEANRTRKDFIAEDILAKSPKTVGVYRLVMKAGSDNFRASAIQGIMKRIKAQGIEVIV

YEPVLAEDTFFGSKVIKDLKQFKQTSDIILANRVTDNLVDVLDKVYTRDVFKGDS"

CDS 14026..14577

/gene="hypothetical"

/codon_start=1

/transl_table=11

/product="hypothetical protein"

/protein_id="AVY03752.1"

/translation="MFKKIILLSFIALIAGCSSSQPETFPGEFANADYVLSDKDAQRW

VVASRQAEQCIYPNLTRIQQQAFSKEDSYIHSQYVFFYPLEEIIGEQYVKIIQDDEKS

MGYAQYQFKKFRDGQEFEPLADKQCQVLREKAKNDLAVVKGQYKSGMVEETKSEGKNP

DGVATNQNKFFFDIIKWGSVLLL"

CDS complement(14659..15201)

/gene="ydeN"

/codon_start=1

/transl_table=11

/product="putative hydrolase YdeN"

/protein_id="AVY03753.1"

/translation="MKKVYVTHGYTANPTRNWFPWLKNELEKLGWECECLAMPNSDQP

NPQAWLEHHQNTLQLDENTLLIGHSLGCIALLNYLAVTQQKVKTAIFVSGFYEKLPTL

PELDSFADFYANQTAYLPQKSYVISALNDTVVPHSFSDRLAQYLQADYIRLETGGHFI

DREGITEMPVLLALIKQIVK"

ORIGIN

1 atgccaaaca tcaacattca caacgcctta ttttcccttg ctcaacacaa taaactgtcg

61 attgaatcgc tggaaatcaa tactcacgat ttctgggtga ttgtcggcgg taacggctcg

121 ggtaaaactg ctttcgccca agcgctacat aattcacttt cactatattc gggcgaatat

181 caaaacagct tccaacatat cgctttactt tccttcgagc agcaacaaaa aatcatcgag

241 caaatcttta aacaccgtaa caacgatatg atttcaccgg atgatttcgg tttaaccgcc

301 cgtcaaatta tcttaaacgg tagcgaaaaa acgcaattat gcgaagaata tgcagctaaa

361 ttacgtattc agccgttatt agatcgcccg tttattcagc tctccaccgg cgaaagtcgc

421 aaggtgttat tttgccaaat gttagttagc gaaccggatt tgctgatttt agatgagcct

481 tttgaggggt tagaccaagc ctcggtcgct tattggcaag acgtgatggc acaactcggc

541 aagcaaatgg cagtagtact gatttccaac cgttttaatg atattcccga ctgtgccaca

601 catattgctt tactggataa cttacaactg attttacaag gcgaacgcca agagattgaa

661 caacaagcgg tctattctca gctaaaattt gcagaacaga atgtgaatgc accgttgccg

721 gacagtgccg caccactgat tcaactccca ccgaatacca atccgttcga actgaaaaac

781 gtgatgattc gttacggcga aaaaaacatt attgatgatc taacttggac agttgcccca

841 aaacaacatt ggtggattaa aggcccgaac ggagcaggga aatcgacctt gctttcgatt

901 attaccggcg atcatccgca agcctatgct aattacgtac atttattcgg acgtcagcgt

961 ggctccggcg aaacgatttg ggatattaag aaaaatatcg gctatgtaag tagtcaatta

1021 catatggatt atcgggtgaa ttgttcggcg ttagatgtga ttttatctgg cttttttgat

1081 tcaatcggtg tttaccaaca agtgccaagt gccttacagc tcaaagtaat ggaatggtta

1141 gaacgcttac atttagccag tctggcgaaa aaaccgttcc gttcactttc gtggggacaa

1201 caacgtttat tattgattac tcgtgccatg gtaaaacatc cgccgattct gattttagac

1261 gaaccgctac aaggcttaga cggcgtaaac cgcaaattgg ttaaacagtt tatcgaacag

1321 cttgtgacta atagtcaaac ccagttgcta tttgtttcgc accaagatgc ggacgcccca

1381 aattgcatca cgcatttatt tgaatttgtt ccgcaagaga atggtggtta tcgttatgta

1441 cagacggctt tgaatcaaat agaaaaacgcc taagatttaa ccacggaaaa cacggattac

1501 acggagttta atgaatcggt cagttgacgt aatgtttgga taatattgtt tttttcaggt

1561 ttttcttatc tcgtaaggaa ctaataaaac tttaatcgga aactgcaact actcgcaata

1621 tcataatttg ttaaccttta aaggaaatcc ccctctttag taaagagggg ttaggggaga

1681 tttgtcaata gagatatgaa attgaataga acttcatttt ttatatttat aaaagcgtta

1741 attagcatat ttcttcgcta attcattctg tcaaatctcc tcctgcccct ctttgctaaa

1801 gaggggagat atgtgtggga ctttaaagcg ttgaattata gaacttataa gcctcgtcca

1861 tattctcaaa tttatacata atccctttat ctaataccat tgcattatcg caataagact

1921 tcattgctga cggactatgc gaaaccaaaa taatcgaacg atctttgcgc ttttcaaata

1981 attcatgttt acattttgcc gcaaagcgag agtcacctac cgcaattacc tcatcaatta

2041 agtagcaatc aaactcaact gaaagcgata aagcaaacgc aagtcgggct ttcataccgg

2101 aagaatattt cttcaccggc tcatataaat aatcgcctaa ttcggaaaac tcttcggtaa

2161 atgctttaac atagtcaata tccgcattat agatacggca aataaagcgt aaattatcca

2221 taccggttaa actgccttgg aacgccccgc tgaaagcgag cggccaagat atcgacatat

2281 tacgttcgat agtacctgat gttggtggtt caacaccact taacaaacgg attagcgttg

2341 atttccctgc accgttacgc cctaaaatac cgattttctc gcctttttc agctcaaaat

2401 taatatcttg caatacggtt tttttaccgc ttcgagtata gtaatcttta ctcacatttt

2461 ttacgctaat cattgcggtt cgatcccttt actgaagttt tttaccataa tgagcccaaa

2521 aagtaacatg gctacatcac atatgacgag ataacttata ctttcatatg tgataacact

2581 gtcgccaaaa taaccgtgac gaaacatttc cgtgccgtga atcatcggta ttaaggttgc

2641 atattgttga gcttggcttg gtagcgcatg cacaaagaaa aatgcgcctg aaagaggtaa

2701 aagaacaaag cttaatgttc cccaaatttt gccgaaggct tcaaattttt gtgcaataga

2761 acaaataatc aagcctaatc ctagtgcaaa aaatgccatt aatacccacg ccataaccat

2821 ataaaacgta tctttcggca tttctatcca gcctaataaa atgactaatg ccataataat

2881 gatttgggca atcgttgcac ctgctacttc aagtatgaca cgagccagta aggtatctaa

2941 tacgcgaacg ttacgatgat aaagaagact caaattaccg gaaattgcac cgatagtgcg

3001 gtttgatgca ttacgccaca tcattgccat tggataaccg gtaatcacaa aagcaataat

3061 atttaaatcg gaaacgcgat ccgctcggat aaatttccac atcaaaacga taaataaagt

3121 gagtaatagc ggctcaacaa acagccataa aaaacccaag ttttttcgtc cgtaacgcgt

3181 aataatttcc cgcatgagta acgcaccgat tactctccct tgaatggcga gagattggcg

3241 gaaagttgtt tgatcaccgt attgcattag tttttatgct ctcttacgct tgcaattaat

3301 aaacttaata caccataaag catcagaccg ataaagaatg tcgctaaaat attatataag

3361 cgataaggct cttccgccca gtccggtttg cttggctgac tgattacttc taaataaagt

3421 tgctggcgat ccgcttcatt tttggtattt tgcaaagaag ttaaagctgc ggttaattgt

3481 tgctgcgcca gctcgtttgc aagtactaag cgttggtaat cggcagtttg aatagcaata

3541 gagctattac tattactgga aagctgtttt gattgctcgt caatttcctt atgtaaactc

3601 ttttggcgca ttaataacgc atcaacttgc gggttatccg gcgtaatcga ttgtaattgc

3661 gctaattggg tttctacacg aatcaactcg ctttttaagc ttgaaattaa tgaaagttgt

3721 acgccggatt gtgccggtaa atcaaaaatt ttatttttga tacggtattt acttaaagca

3781 tttgccgttt cgtttacatt attttccgct tctgtaaccg cttgttccgc aaatgaaatg

3841 gtatcttttc ttgcacgttc gtttaaacgg ttaataagcg tttcaccttc ggcaaggagt

3901 ttttgattaa tttgttgccc ctcttccgcg ttaaatgctc gaatacgtaa gctggcgata

3961 ccggaaacag agtcaaagtc cacacttaag cgatctcgga aatatttata aaacgcttct

4021 ttactgttat ttaatccgaa tccattaaag cgagcgataa tatcgccctg attttcatag

4081 tattcacgta ttggcaagtc tttcattaac tgttctagtg ccgtacgaga atgcatatat

4141 tcttgtacgg tataagtatc atcttgagct cgagaaaatc cggaaccttg taataaggca

4201 ccgacaccgg ttaaagcggt ctgattttga ggagatctta caacgaagct tgattccgaa

4261 atataaatat cggaagcaac agaaccgaaa taaaaggctg ataataccgt aggaatcgct

4321 acagttaccc aaaataacgg attaagcttt tttaaccaac tttttttctg tttaaccggt

4381 ttttgtagtt tttctgtcgg acttgccgta atagttgttt ccatctttta tccttataaa

4441 ttcaatatat taataggcac gaacagcatt agtcgtactt gtaaccggcg aagtaatcga

4501 gaaaatcatt ctcaagaatt tttggaattc agacaacggt gcatttgata catagacaat

4561 atctttatct tgcatcggga agcgttgtaa taaaaacagt gattgcggct caagtaaatt

4621 cacacgataa actgtcggta catccatacc cataccatag cctttcgctc cccattgcgt

4681 ttgctgatct aaacttaatt gagaaaaagg cacatgacgg aagacgaata cccctctcgg

4741 atctgaacga gtatcgatta aaccgcccat cttaccgata gcttcggcaa gcgtaagacc

4801 tttacttgag aatttcattt gctggttatt gcctacggca cctaaaccgg taaagctata

4861 aggtgtgttt agcagtgaaa caacatcgcc agcacgtaac ataatatttt gcgctggatc

4921 ggaaattaac gtttcgaatg cgagtgtttt tacttcagaa ccacgggtta gcttgaccgt

4981 cacatcttca atattttccg ttgttccgcc tactgcagca accgcatcta atactcgttc

5041 attattagcg gttaatggca tacgaatact attaccttgg cgaataaccg taacatcagc

5101 agagttatta ttcgcaattt tcactaaggc ttgcggctga ttcgctttgc gctgcaatgc

5161 cccaataatt tgagactgaa tcgcttccgg cgttttgcct gcgacacgaa tgttacccac

5221 gaacggtacg gtaaccgtac cgttttgatt caccatttgt gccggtaatt gcgttaaatg

5281 cccgctacct tgtccctcag aactaaaagt accgccaaac aacaccgccg gcggggcttc

5341 ccaaattgat atttcaagta catcacccac attgaccgca ccggcataac ccgcaccgcc

5401 tgccgtacct aaaaaaccgg aaaaattctg tttttcttgg atgtgatata aattgtttac

5461 tatagctatg ttcagttcaa ttaaattcgc ttcaaattgc ccattcttat ttatttctgc

5521 tacttcattt agcgaaggtc ctgttgtagg taaagatgaa cagcctgaaa gtaaaataca

5581 taaatgtaat gtatatattt gtaatttcat tgggtgaacc ttatatttat agggtaaaat

5641 aaattataat tataatgtaa tttttagtat gtgttaagaa aaattataat tatatactta

5701 attagtaatg tatttttgtt tgctttgatg ttatagggaa tttattttgc tattatttaa

5761 atatttaaat tatataagat attttcttaa aataagagaa aaaataaaaa ataaagagtt

5821 tatatatgta ttagcaaaat ataaagattt tcttccatta aatttaaaat tatttttatt

5881 atacaaaaca ggattgtata ataaaataat agagaatata gactataaaa aatatattta

5941 ttatgatatt tcgaaggtag ctttgggata taaatttagt tatgaaagta ataatgcctt

6001 tttatctaag tatagaaata atatatcttt tattttaggc attctacctt ataatccaac

6061 cttggctttg gattgtcttt gccataaaaa gtattattta ttaaagcttt ttatattgta

6121 tcatttaggt caaaaaatag actttgttcc taacaagata aatcacccag atttatattt

6181 attatgtaat aatgatagtg taaattctaa tatagaaaat ataaataaat atctagatta

6241 ttacaattta agtaaaatca gattcattaa taaagaggct aataatttta ttaataatat

6301 aataacctct caattctcat ctcctgctca aatagatgat ttacctcttg tgtcaatttt

6361 aatgacaaca tttaatagtg aaaaatggat tgagtggtct attaactctt tattatcaca

6421 aacatatagc aatattgaaa ttattattat tgatgattca agcacagata gtaccatatc

6481 aataatagaa aagttaaaaa gaaaatccga caaaataaaa ttaattaaat tatcaaaaaa

6541 tgttggtact tatgtagcaa aaaatattgg tattagttta gctaaaggag agtttattac

6601 ctgtcaggat tcagatgatt gggctcatcc tcaaaaaata atgctacaga tatatccact

6661 tttggagaat cctaatttag tagttacatt ttctcaatgg tttcgtatat ataacgatgg

6721 aaaagcatat gccagaggag tatttcctct tacgagatta aatccttcat ctgcattatt

6781 tagaaaagat attgttatag aaaaaatagg attatgggat tgtgttagaa cgggagctga

6841 tagtgaattt aatgcaagac taaaattagt atttggtaat gaattatgtt tgacgataaa

6901 gaaacctctt actattggta ctcatagaaa ggattcttta atgacatcta gcgatactgg

6961 atatattaat ggtatttctt tagagcgttt aaattattgg gagagttgga atctttggca

7021 tatttccgaa atagaaaaag gaagagtacc ttattatcta ttaaataata aacgtccatt

7081 taacatatca gataaaaata aagttgatta taaagatata atatgtaatc tatgttctat

7141 taaatgagat ttattattag attagatttt tgtataaaga ggatatatga aaaaaatcat

7201 aattattggg cactctacat ctggatatca aagtgtagag gaacttttcc aagtagctgg

7261 tatgagccct gctctccctt ctaaaagaga tgggatgaca cctcaagaaa tagattctgt

7321 tttgaaaaaa gttatttttt ctccatctca ttcttcaaaa gaattattac cagcaaagcg

7381 ttaccagtca aaaaggcaag ctaaaaaaat tgcacaaaaa tttgatgttg tccaatcgtc

7441 tatagtgcca gcagagactt taatcaatcc aatatgggat catttagcgt tagatttaat

7501 gttggggaat ttagatcaat ctttttgggg atggtctaat ccaaatgcgt tggatacttt

7561 agaatattgg cagcgtattg atcctgaaat ttattttgtt tttgtctacg ataaccctca

7621 tagcgtattg ttacaatata cagaagaaga aatcctatta ttggatgaca agaaaattac

7681 agagaaatta gatacttggg ctaagtataa taaaaaaatg ttgaatgctt ttgaaaaatt

7741 tcaagatcgt tctgtattaa ttagtagtaa acaattaatt gaattttcag aaaattcaat

7801 tagaaccgta tatgatcaaa ttcaagcacc tattgactta gaatctagag aattaacatt

7861 agatttagtt agaaataata tatcttctaa taataagtat ataaatactt ttcttgtaga

7921 gtgtattatt aataattact ctgatatgat tcttatttat gagaatttac aagagagtgc

7981 tgatttgcct tatttatcta agatggatat taaaaatgga atggttctcc aatcatggaa

8041 agaaattatt aaagataaaa tattttttca gtctcaaata aaagaaaaaa ataaattatt

8101 aagtcaatat aaagaagaat atagtataag agaaaaattt gaatctgcga ataaagttat

8161 tttggctcag ctacatttaa cacaggatct tttagaaaaa gagttattaa ttaaacaaga

8221 aatggaaaaa aatagttcta tttataggca gcgtttagat gtttatgaga gtgataaaat

8281 tgatttaaca tctaaattaa ggagtttaga gaatgacaag aaagaactac aatctaaatt

8341 gaataattta gaaaatgata ataaaaatct acaatctata ttagagcatg tagagaattc

8401 ttataataat gtttcaaaca atgtagaaaa tattagagct caattaactt tgactgagac

8461 tgaaaatcaa aatttaattt tacagttaca tttaactcaa gatgagttag aaaaacaata

8521 tagtgttata tcatcgttac agaagcctat ttatttaggt gctgcagata gaataaagag

8581 tgaaatgcct tatcggctag gaaaaaaaat gatagaagct agcaaaagtc taaagggatg

8641 gctaactatg ccttgtctcc taaaaaagga ggcaaataag gttaaggaag aacaggaaaa

8701 gagtgaaggt cttaaatata tcaatattga agagtatgcg gatttttctg atgctgagaa

8761 agtaaaaaaa catttatctt ataaattggg agttaaatta ctaaaaaata taagaaaacc

8821 ttacttatgg atttttatac catttagttt attaagcaca atatatttat ttagaaaaaa

8881 ttaatatgtt tttctaaaaa gtagttgggt tatacaaaag gatttgttta taaagttata

8941 aattttattt ttaatttggt cttattgtta tgagtttaaa attagctaat caattatttc

9001 aagagggtaa atatcagcag gctttagttg aatatcaaaa agttaaatct gatcatcctt

9061 tatatcaaca tgcacaattt aatataaatt taattcatgt taaattgcat caagatatta

9121 tacctactaa tagtatctct aagttagaga gtaatgaaca gcctttagtc agcgttgtaa

9181 tgcctgtgtt taatgttgct ccatatttag atgctagtat tatgtcagta ttaaaccagt

9241 cttatacaaa tattgaacta attattgtta atgatgcttc aacagataat gggttaaata

9301 ttattaacat gtataaaaat tgggattcgc gtattaaggt agtaaattta gaatttaata

9361 ctatgggcgg ggctggtatc ccaagtaata ttggagtaga taatgctcga ggtgaatatt

9421 tagcatatgc agatagcgat gatatcttag ataaatatgc tattcaaaaa atgatggagt

9481 ctgctttaaa gcacgaggct gaagttatta ttgcggattt tagtaatttt aataatgaaa

9541 ttagagttat tgagaatggc tatgacaaga aaaattggaa taatatccct cttgatgaag

9601 ttttttctcc aaaagataaa tctgaaatat ttagattatc tccagttcca tggagaaaat

9661 tatataaagt ttctttttta aacaataata aaattcgttt tccagaaggt gactatttct

9721 acgaggataa tccattacat tggtttgttt taactaaagc taaacgagtt gtattgttag

9781 attatgtagt agcctatcat agaatggagc gagaagggca gactatggga gcaatgaatt

9841 tcaaattatc agctcaattt tgccatttaa attctattaa aaatcatctt ttaaaaatga

9901 aagatgtacc tcgtatctat tggaaagaat tagttgattt tgcttatcgt ggaggttggg

9961 ttgtagatag acaagatatt cctgaattcc agtcaattgt taagaaacgc tatgcccaaa

10021 cagctctggg aattgagaaa ttatctcata tccctaaaga tgaaattaga aaaatgcgtc

10081 ctaattttta taagcgttgt gaggaatata atcaagccta tgcagattta gatttatcta

10141 ttgtcgtgcc agtttataat tgtgtagatt tattgccaca actaatggaa tctttattaa

10201 aagtcaattt aaaaacagat atattcttaa ttgatgatgg ttctaaggat ggctcacgag

10261 aactatgtga aaaatatgct aaacaatata aaaatgtata ttgtattgct caagctaata

10321 aaggtgctgg agttgcaaga aatttagtta ttccattatt aacaggagaa tattcttatt

10381 ttgttgatgc tgatgatttt attgatccta agtcattaga agaatcagta aagtttgctc

10441 agaaaaataa tcatgattta gtattattta aatataaaat tgaatttcat gaaaaaggta

10501 atactcgtga tatgtgggat gcagataaaa aaatctgggc gaaattatta gtcgctaaaa

10561 ataataatga aagaaaaatt ttagcaagtc agctaataaa ttatccttgg aatcgagctg

10621 ttaaaacatc acttctccac gatgaaaaca ttttctttgg aaaaaccgtt gtacataatg

10681 atgtacctta ccattggcat acggtagttt ctgcaaataa tatcggaatt tatgataaac

10741 ctgtttgtac tcataggaaa tttgaagaac gtcagcaaat tactaatatt tcggattatc

10801 gtcgattaat ggtattagaa gcctatcgtc acactcatga gcttttaaaa cgttatgatt

10861 cttatgcatt gattttccct cattggcaaa aatttattcg tgatttactc acttgggcta

10921 gggatagggt gccggaagat aaattggagt tttataaaga acgtcataaa caaattcttg

10981 atgaattaaa aggagtaaat ctatgaaaaa tatcgcaatg gcaggagcag ggttatcttg

11041 tgctgttatc gctcgcgaac tagccgaaca aggttataag gtcactatct ttgaagggcg

11101 taatcatatt gctggtaatt gccacacgca aagagacgaa aatacaggag taatggtaca

11161 tgtttatgga ccacatattt tccatacaga taatgagaat gtatggaatt atgtaaataa

11221 atatatggaa tttaaacctt atgttaatcg agtaaaaagt actatattag gtaaagttta

11281 ttcattaccg attaatttgc atacgattaa ccaatttttt ggtaaaaatt tatctcctaa

11341 agaggcaaaa gcttttattg agcaacaagc tgatatgtct attacagatc ctcaaagctt

11401 tgaggaacaa gcaatgcgtt ttgttggtaa agatctatat gaagcttttt ttaaaggata

11461 tacaaaaaaa caatggggat tagaaccgag ttccttacca gcaagtattt taaaacgttt

11521 gcctgtgcgt tttaattatg atgataatta ttttgcccat cgttttcagg gaatgcctgc

11581 agaaggatat acaaagatgg tagaaaatat tttaaatcat agcaatatta ctgtacatct

11641 caatacgctt tttaaacgtg aacaagttca acattatgat catgtatttt attccgggac

11701 aattgatggc tattttaatt atgaatttgg tcgcttacct tatcgcacat tagattttaa

11761 aaaatattat gatgaaggtg attttcaagg ctgtgcagta atgaattacg gtgatgaaaa

11821 tattccttac actcgaattt ctgaacataa acattttgct aattgggaag atcatgagaa

11881 aacagtatat tttgaagaat acagtcgcca atgtacacct aatgatattc catattatcc

11941 aattaactta gtagaaggta atgagttaat tactaaatat aaagagaaag caaaacgaga

12001 acaaaaagtt acttttgtcg gtcgattggg tacattccgt tatttagata tggatgtaat

12061 tattaaagaa gctttagata tagcaagagg attttatgtt aagtgatggt atcagaaacc

12121 aaattaaaat tggtgaaaat atttttcgtt taatatgtaa agatggtcga gttattgata

12181 atcctgaaat aaatggttta caggttatat ttcaaggaag aatgggtggt acggttgaaa

12241 tagaggaaga tgctgtattt cataatacaa aaatttatgc cggtggagca ggttttattc

12301 gtataaataa aactcaccct aaaggtatca aaaacgtcac gattaaaaca gtttgtcctt

12361 gtccttataa atatctgttg attgatgagg gatgttctat agaacaggct gtatttatgt

12421 tagtaaatga tgaagattta gtggttaaaa ttggaaaaga ttgtatgtta agttccggaa

12481 ttttttcag agcagcagat ggacatacaa tatttgatgt taatactaaa aatgtaatga

12541 attattcttc tcctattatt attggagatc atgtatggat aggagcaaat agtacctttt

12601 tgaaaggtgc ggaagttgct agtaatagta ttgtagggac tcattcatta gttactaaaa

12661 aatttagtaa acaattttgt gctattgcag gtattcctgc taatgtagta agagaaggta

12721 ttaattggga taggttgaga ataacagaat ataagcagca cttaagtgtg aaataggtta

12781 tgaatatatt tatggttaat aattagggta gaattcaatg aatataacaa ttataggtac

12841 cggttatgtg ggattatcta atgctgtttt gttggcacaa aaacataatg ttacggcatt

12901 agatattgat aaggagaaag tagcgaaaat caatgcgaag caggctccta ttgaagataa

12961 tgaaatagag tattttttgg cgaataaaaa gttatcttta ttagctacag tagataggta

13021 tttagcgtta gaaaatgctg aatttgttat tattgctact ccaacaaatt acgatcctaa

13081 aacaaactat tttgatacaa gtagtgtgga gtctgttatt cagcaagtgc ttgaaattaa

13141 tccaaatgca ataatggtaa taaaatcaac cattcctgtt ggatttacgg aacaggtaaa

13201 acaaaagtat aaaacggaaa atattatttt ctcgcctgaa tttttacgag aaggtaaggc

13261 tttatatgat aatttatatc cttcaaggat tattgtggga gaaaagtcag aacgagcaaa

13321 aacttttgca aacttattac ttgaaggtgc tattaaaaaa gatgtagata tattatttac

13381 ggataatacg gaagcagagg cgattaaact gtttgcaaat acttatcttg ctatgcgtgt

13441 tgcatatttt aatgagttgg atacttatgc ttctattaga ggcttaaata cggaagatat

13501 tattaaaggc gtatgtttag atcctcgtat tggcgatttt tataataatc cttcctttgg

13561 atatggcggt tactgtttac caaaagatgc aaaacagtta ttagctaatt accaagatgt

13621 tccgcagaac ttaattaatg cgatagtaga ggcaaaccgt actcgtaaag attttattgc

13681 tgaagatatt ttagcaaaat cacctaaaac ggtaggtgtg tatcgtttag tgatgaaagc

13741 aggatctgat aatttccgag cttctgctat tcaagggatt atgaagcgga tcaaagctca

13801 gggaatagaa gtaattgttt atgaaccggt tttagccgaa gatactttct ttggttcaaa

13861 ggttataaag gatttaaaac aatttaaaca aacatctgat attatacttg ctaatcgtgt

13921 tacagataat cttgttgatg tgcttgataa agtatataca agagatgttt ttaaagggga

13981 tagttagtac cctttattta taggaaaatt aagggtatac atattatgtt caaaaaaatc

14041 attttattga gttttatcgc gttaatcgcc ggttgttctt cctcacaacc ggaaactttc

14101 ccgggggagt ttgcgaatgc ggattatgtg ttatcggaca aggatgcgca gcgttgggtg

14161 gtggcgagtc gtcaggcgga gcagtgtatt tatccgaact tgacgcggat tcagcagcaa

14221 gcgtttagca aggaagattc atatattcat tcgcaatacg tatttttcta tcctctggaa

14281 gagattatcg gcgagcaata tgtgaagatt atccaagacg atgaaaaatc catgggatat

14341 gcacaatatc aatttaagaa gtttagagac ggtcaagagt ttgagccgtt agcggataaa

14401 cagtgccaag ttttacgaga aaaagctaag aacgatttag cggtcgtaaa agggcagtat

14461 aagagcggaa tggttgaaga aacgaagtcc gaaggtaaaa atccggacgg tgtggcgacc

14521 aatcaaaata aattcttttt tgatattatc aaatggggat cggtactatt actgtaattc

14581 acgcttgttg tgatgttgga ctctccagat aagtacaaaa agagctgtcg gtacgttgtt

14641 atcgacagct ctttttgact attttacgat ttgcttgatt aacgccaata ataccggcat

14701 ttccgtaata ccttctcgat caataaaatg tccgcctgtt tccaagcgaa tatagtcggc

14761 ttgtaggtat tgtgctaacc gatcactgaa cgaatggggt acaaccgtat catttaatgc

14821 ggatatgacg taagactttt gcggtaaata agcggtctga tttgcataaa aatctgcaaa

14881 gctatctaat tccggcaaag ttggtaattt ctcataaaag ccggaaacaa aaattgccgt

14941 ttttactttt tgctgcgtta ccgccagata attcagtaac gcaatgcagc ccaaactatg

15001 tccgatgagt aaggtatttt catctaattg aagtgtattt tggtgatgtt ccagccatgc

15061 ttgcggattc ggctgatcgg aattcggcat cgctaaacat tcacattccc atcctaattt

15121 ttccaattcg tttttaagcc acggaaacca atttcttgtc gggttcgccg tataaccgtg

15181 cgttacatat acttttttca t

//

LOCUS **MG780416.1** 16020 bp DNA linear BCT 16-MAR-2021

DEFINITION Actinobacillus pleuropneumoniae serovar 17 strain 16287-1 capsule locus gene cluster, complete sequence.

ACCESSION MG780416

VERSION MG780416.1

KEYWORDS .

SOURCE Actinobacillus pleuropneumoniae serovar 17

ORGANISM Actinobacillus pleuropneumoniae serovar 17 Bacteria; Proteobacteria; Gammaproteobacteria; Pasteurellales; Pasteurellaceae; Actinobacillus.

REFERENCE 1 (bases 1 to 16020)

AUTHORS Bosse,J.T., Li,Y., Sarkoezi,R., Fodor,L., Lacouture,S., Gottschalk,M., Casas Amoribieta,M., Angen,O., Nedbalcova,K., Holden,M.T., Maskell,D.J., Tucker,A.W., Wren,B.W., Rycroft,A.N. and Langford,P.R.

TITLE Proposal of serovars 17 and 18 of Actinobacillus pleuropneumoniae based on serological and genotypic analysis

JOURNAL Vet. Microbiol. 217 (2018) In press

REMARK Publication Status: Available-Online prior to print

REFERENCE 2 (bases 1 to 16020)

AUTHORS Bosse,J.T., Li,Y., Sarkozi,R., Fodor,L., Lacouture,S., Gottschalk,M., Angen,O., Nedbalcova,K., Holden,M.T.G., Maskell,D.J., Tucker,A.W., Wren,B.W., Rycroft,A.N. and Langford,P.R.

TITLE Direct Submission

JOURNAL Submitted (11-JAN-2018) Medicine, Imperial College London, Norfolk Place, London W2 1PG, UK

```
FEATURES             Location/Qualifiers
     source          1..16020
                     /organism="Actinobacillus pleuropneumoniae serovar 17"
                     /mol_type="genomic DNA"
                     /strain="16287-1"
                     /serovar="17"
                     /note="K locus: KL17"
                     /isolation_source="lung"
                     /host="pig"
                     /db_xref="taxon:2138311"
                     /country="Denmark"
                     /collection_date="1997"
     CDS             1..1458
                     /gene="modF"
                     /codon_start=1
                     /transl_table=11
                     /product="putative molybdate ABC transporter ATP-binding
                     protein ModF"
                     /protein_id="AVT42387.1"
                     /translation="MPNINIQNALFSLAQHNKLSIESLEINTHDFWVIVGGNGSGKTA
                     FAQALHNSLSLYSGEYQNSFQHIALLSFEQQQKIIEQIFKHRNNDMVSPDDFGLTARQ
                     IILNGSERTQLCEEYAAKLRIQPLLDRPFIQLSTGESRKVLFCQMLVSEPDLLILDEP
                     FEGLDQASVTYWQEVMAQLGKQMAVVLISNRFNDIPDCATHIALLDNLQLILQGERQE
                     IEQQAVYSQLKFAEQNVNAPLPESATPLIQLPPNTNPFELKNVMIRYGEKTIIDDLTW
                     TVAPKQHWWIKGPNGAGKSTLLSIIAGDHPQSYANYVHLFGRQRGSGETIWDIKKNIG
                     YVSSQLHMDYRVNCSALDVILSGFFDSIGVYQQVPSALQLKAMEWLERLHLANLAKKP
                     FRSLSWGQQRLLLITRAMVKHPPILILDEPLQGLDGVNRKLVKQFIEQLVTNSQTQLL
                     FVSHQDADAPNCITHLFEFVPQTNGGYRYVQTALN"
     CDS             complement(1657..2307)
                     /gene="cpxA"
                     /codon_start=1
                     /transl_table=11
                     /product="capsular polysaccharide export protein CpxA"
                     /protein_id="AVT42388.1"
                     /translation="MISVKNVSKDYYTRSGKKTVLQDINFELKKGEKIGILGRNGAGK
                     STLIRLLSGVEPPTSGTIERNMSISWPLAFSGAFQGSLTGMDNLRFICRIYNADIEYV
                     KAFTEEFSELGDYLYEPVKKYSSGMKARLAFALSLSVEFDCYLIDEVIAVGDSRFAAK
                     CKHELFEKRKDRSIILVSHSPSAMKSYCDNAMVLDKGIMYKFENMDEAYKFYNSTL"
     CDS             complement(2304..3101)
```

/gene="cpxB"

/codon_start=1

/transl_table=11

/product="capsular polysaccharide export protein CpxB"

/protein_id="AVT42389.1"

/translation="MQYGDQATFRQSLAIQGRVIGALLMREIITRYGRKNLGFLWLFV

EPLLLTLFIVLMWKFIRADRVSDLNIIAFVITGYPMAMMWRNASNRTIGAISGNLSLL

YHRNVRVLDTLLARVLLEVAGATIAQIIIMALLILLGWIDMPKDTFYMIIAWVLMAFF

ALGLGLIICSIAQKFEAFGKIWGTLSFVLLPLSGAFFFVHALPSQAQQYATLLPMIHG

TEMFRHGYFGDNIITYESISYLVICDLAMLLIGLIMVKNFSKGIEPQ"

CDS complement(3101..4258)

/gene="cpxC"

/codon_start=1

/transl_table=11

/product="capsular polysaccharide export inner membrane

protein CpxC"

/protein_id="AVT42390.1"

/translation="METPIATSPAEKLQKPVKQKKSRFKKLNPLFWITVAIPTVLSAF

YFGSVASDIYISESSFVVRSPKNQTALTGVGALLQGSGFSRSQDDTYTVQEYMRSRTA

LEQLMQGLPVREYYENQGDIIARFNGFGLNNSKEAFYKYFRDRLSVDFDSVSGIASLR

IRAFNAEEGQQINQKLLAEGETLINRLNERARKDTISFAEQAVTEAENNVNETANALS

KYRIKNKIFDLPAQSGVQLSLISSLKSELIRVETQLAQLQSITPDNPQVDALLMRQKS

LRKEIDEQSKQLSGNSNSSIATQTADYQRLVLANELAQQQLTAALTSLQNTKNEADRQ

QLYLEVISQPSKPDWAEEPYRLYNILATFFIGLMLYGVLSLLIASVREHKN"

CDS complement(4284..5471)

/gene="cpxD"

/codon_start=1

/transl_table=11

/product="capsular polysaccharide export protein CpxD"

/protein_id="AVT42391.1"

/translation="MEIKKYNSIIGLALTTLFLSACSSLPTSGPSHSAILEANSQSSD

NPLPEVNVVELDNGLVQQLYQTQQSQQFSGFLGTVGSAGYAGAVNVGDVLEISIWEAP

PAVLFGGTFSSEGQGSGHLTQLPAQMVNQNGTVTVPFVGNIRVAGKTPEAIQSQIIGA

LQRKANHPQALVKIANNNSADVTVIRQGNSIRMPLTANNERVLDAVAAVGGTTENIED

VTVKLTRGSEVKTLAFETLISDPAQNIMLRAGDVVSLLNTPYSFTGLGAVGNNQQMKF

SSKGITLAEAIGKMGGLIDTRSDPRGVFVFRHVPFAQLSLEQQAQWQAKGYAIGMDVP

TVYRVNLLEPQSMFLLQRFPMQDKDIVYVSNAPLSEFQKFLRMIFSITSPVTSTTNAV

RAY"

CDS 5650..6792

/gene="cps17A"

/codon_start=1

/transl_table=11

/product="CDP-glycerol:poly(glycerophosphate)

glycerophosphotransferase 17A"

/protein_id="AVT42392.1"

/translation="MLMKIAFIAWNSFQVLHFKPLLQALPCALLIIEKRRRSVPICKD

ILRDINNNIAYIRHMDIYAKIDGNFDVLVAQTAFEQLYLFRHTKIALLQYGYAKEPHN

YGTWRALADLNLVYGSYAYERISYFSPTEITGCPRYDLWYLPSFHQKAKENYARVLDT

SKKTIVYAPSWGELSSFKLYIEEITKLSLFYNVLVKLHHNTLLLANKHQNYEKLYPNL

HFFYEGEDLLSLISVADIVISDFSGAIFDAIFCKKPVILFSIPLVNQPKLDKFSLEIA

HRSALGYEVFSPDQVAITVARALTEPKLVDETLYQQLFMHNKDATQQVINALQQLAEG

KYTLSQQQLYVRQTEKLLNIEKIKQQKNKKQSFNKIRQISKRLIKK"

CDS 6845..7273

/gene="cps17B"

/codon_start=1

/transl_table=11

/product="glycerol-3-phosphate cytidylyltransferase"

/protein_id="AVT42393.1"

/translation="MKKVLTYGTFDLLHHGHIRLLERARSLGDHLTVAISTDQFNLGK

GKVCAYTYEERAHILKAIRYVDEVIPETKWEQKIDDVKNHEIDVFVMGDDWEGKFDFL

ADYCEVVYLPRTPDISTTQVKKMLAKKDLATGQKQIHEKE"

CDS 7281..8423

/gene="cps17C"

/codon_start=1

/transl_table=11

/product="capsular polysaccharide biosynthesis protein

Cps17C"

/protein_id="AVT42394.1"

/translation="MFQILQKHLPTLQRVLREGYSQHSLLAYWYGLSLLTALEQANHP

QVRKLAEKMINKGINIGHYFLAQSYFLCGEYDLAEQAVKKIKNFVKIPEVVFLYADIL

VKCKRKEEAWQLLEQCALLNKRKKVWIYLANLVNTIADFQRLEQHIEKVRTTTPHLKF

ELLIHQRTNAALRAGLTETALALTELNPLPKQAKVKKKTTAYNDKLAAIALADLKKVL

DHKKIPFFLISGTLLGCIREGKLLGHDKDIDIGVWDKYSYEELANCLSTSGYFYVVPT

RTNHLVMLRHVNGIAIDVFIHYRESNDYWHAGVKIKWHNSPFNLVYTNFLGQQYLIPE

NYDLYLTENYGDWRTPKTQFDSAFDTPNMEVINEVEMKVYISKIQK"

CDS 8443..11400

/gene="cps17D"

/codon_start=1

/transl_table=11

/product="CDP-glycerol:poly(glycerophosphate)

glycerophosphotransferase 17D"

/protein_id="AVT42395.1"

/translation="MKFLKNSYHNVIAPKGYHRGLVLYRKKQWTEALSCFEAAYSTSP

LHAKNTFKLGLCHLKLGNFSEAHSFIAKALEIAPYNTHWRKQLQQAERHLNNTYSSPH

KITTVVTRMKQSGISQSIGTAIRKTVLLIPSDYNHRVMADISSFIQYYKDKFDVYIIL

RELPEDIVYKNTHVLVKNGTSFGEYLKFTADYVIDSGTMNYSYRITDTNKWVSVWHGI

PYKKMFVDFDIKNLATAIRYDLAYDSMVSMSNFYTDTFLRKAMRYDGEILQLGCAKID

NLFSSISTSNADKVNALRNELGLPNNKKVILYAPEFREVGELYFPFDPNKLLSHLGEE

YCLLTLLPFKGYIEQAENNIYYISDLDNKDALLIADLLISDYHELIYTFDRYNKPAVL

IQYDYESFVKQHTSRKQELEILASRKYVAKEANELYQFNWNLLKRYSKQSTLPEYLDS

SYIKHKLGIPFDKKIVLYAPTFRKAGAVQLPFDPNTLLNYLDNDYVLITKMHYLNYLA

NTYNGVIDCTSHENMAELMKIADILISDYSSLVLDFAVLNKPIILFQYDYDEYMKQRG

VYFNFGDYLPKEQIIRTEFELYTLNWNKLNSDNSKIINEFYPLEDGKSTQRIVDKINF

NADLRFSKDIIFLVNDLNQIGGVHSFLKNMAKYYKQKYNSRIYVIAIKEFAEANSEYH

LLESEYIDFKLSSQYLNGACANILQNTDGIVISLQFSAHMYFQKYLTNAKSVLMFHGD

VKDMISRELYGPHLDWLNKGKLYNYQKLLLLTQSALDLLKPHLNPEIQDKLGFMHNSI

DEEFSPIKQNKKHQLNTAVISRLDADKNIFAMIDLGKEILAQNSNVVVNIYGDGALKD

EFIAEITRHGLEHILKVRGFESNKSKIFSENNSLLLMSKSEGFPLVLLEAYAYGKPVI

VFDSFTAAKEIVKHNQSGFLLPYGDYGNVVKAIENSKNIKLKDIEMIFNNFSNPTVFA

KWDSLILALEQTA"

CDS 11413..12447

/gene="cps17E"

/codon_start=1

/transl_table=11

/product="glycosyltransferase family 2 protein Cps17E"

/protein_id="AVT42396.1"

/translation="MKLLKKLFGRNKLEQPLISILVPCYNSRKTLPATLKSIQQSNYK

NLDVMIVDDGHEVTVEDIVSSFNDPRFRYFYKKNEGLGLTRNFGIDNAKGEFIFFLDS

DDLIYPDAFSNLINYMLENNLDVVSGVTVRRDFETNVESEWCRALYRSKKISTFENRL

SLFDDALSTNKLYRLSMLREKDIRFETGLYEDKVFTAKLYSLVDRIGLIDNRVYIWFI

YGSQTSISTSKSVSNFKGRMAAINNLWQYIPEMRKTYQIAFYMNHDLLIYLREFEFYS

EEEKNEIYNIAYEFIHRHKKYIYNRLIPNSWNRTCLDALCEGNKEKFIYTANTLSKVF

QEELSKKQKV"

CDS 12449..13996

/gene="cps17F"

/codon_start=1

/transl_table=11

/product="capsular polysaccharide biosynthesis protein

Cps17F"

/protein_id="AVT42397.1"

/translation="MENLNSNLRNKINEKNRARLKNKDFSILSSNCNGGVMLHDLGLR

FNSPFINLYLKPKDFIKYCSNISHYISCDLKFIDNDKYPIAYLDDVEIRFLHYASNKE

AEEKWISRTKRINLDNLFIMMTERDGCTYDDLLSFDALPIENKVVFTHKYYPDIKSSI

YIPGFEDNEHVGVLLDFIGKSGERYYDYFNYVDWFNGVELSELKSGEMSEMFKMFLSE

KNKVTPNKKAINKINLLHIDNNKLLFIEGLNYIEGFNSPDYTYLIKNLKIINLATNVE

FEYPLGTVPKKEMSNTLYGDKYFDYTAAGTATMGFKGIDVNHLEEGLYEVQISVSENK

EERNYQNINFTAGHLDKYASDDYFEYRLFKNQNKIYLAKRKLIGRNPISDYFISIEKE

WIKGKTMHIEGAFVIPGIDITEFNQARYYLIAQKAITQKQYSFALGQIKKAGLGEKIN

NPQGSYNACYYATKMLKGIDMSALEFGFYDLYISLSYKSEVFTVKLNKQLEIGHQLLK

LVDNIEE"

CDS 14616..14729

/codon_start=1

/transl_table=11

/product="putative small periplasmic lipoprotein"

/protein_id="AVT42398.1"

/translation="MKKLLLAVLIASFGLAACGVKGPLYFPEQQPAQQQTK"

CDS 14860..15255

/gene="lysA"

/codon_start=1

/transl_table=11

/product="diaminopimelate decarboxylase"

/protein_id="AVT42399.1"

/translation="MARLGSGFDIVSQGELERVLAAGGEPSKVVFSGVAKSHSEIQRA

LEVGIRCFNIESIAELHRINEVAGQLGKIAPISLRVNPDVDAHTHPYISTGLKENKFG

VSVTNEGNKYWEKRYEKKHRRKTYCVKVR"

CDS complement(15478..16020)

/gene="ydeN"

/codon_start=1

/transl_table=11

/product="putative hydrolase ydeN"

/protein_id="AVT42400.1"

/translation="MKKVYVVTHGYTANPTRNWFPWLKNELEKLGWECECLAMPNSDQP

NPQAWLEHHQNTLQLDENTLLIGHSLGCIALLNYLAVTQQKVKTAIFVSGFYEKLPTL

PELDSFADFYANQTACLPQKSYVIYALNDVVVPHSFSDRLAQYLQADYIRLATGGHFV

DREGVTELPVLLELLKQILK"

ORIGIN

1 atgccaaata tcaacatcca gaacgcctta ttttcccttg ctcaacacaa taaactctcg
61 attgaatcac tggaaatcaa tactcacgat ttctgggtga ttgtcggcgg taacggctcg
121 ggcaaaaccg ctttcgccca agcgctacat aattcacttt cgttatattc gggtgaatat
181 caaaatagtt tccagcatat cgctttactt tccttcgagc agcaacaaaa aatcatcgag
241 caaatcttta aacaccgtaa caacgatatg gtttcaccgg atgatttcgg tttaaccgcc
301 cgtcaaatta tcttaaacgg tagcgaaaga acgcaattat gcgaggaata tgcggctaaa
361 ttacgtatcc agccgttatt agatcgcccg tttattcagc tctccaccgg cgaaagccgc
421 aaagtgttat tttgccaaat gttagtcagc gaaccggatt tattgatttt agatgagcct
481 tttgaagggt tagaccaagc ctcggtcact tattggcagg aagtgatggc acaactcggt
541 aagcaaatgg cggtggtact gatttccaac cgttttaatg atattcccga ctgtgccaca
601 catattgctt tactggataa cttacaactg attttacaag gcgaacgtca agagattgaa
661 caacaagcgg tctattctca gctaaaattt gcagaacaga atgtgaatgc accgttgccg
721 gagagtgcca caccgctgat tcaactccca ccgaatacta atccgtttga actgaaaaac
781 gtaatgatcc gttacggcga aaaaacgatt attgatgatc taacttggac ggttgcccca
841 aaacaacatt ggtggattaa aggcccgaac ggagcaggaa aatcgacctt actttctatt
901 attgccggcg atcatccgca atcttacgct aattatgtgc atttattcgg tcgtcagcgt
961 ggttcgggcg aaacgatttg ggatataaag aaaaatatcg gctatgtgag cagccaatta
1021 catatggatt atcgggtgaa ttgctctgcg ttagacgtga ttttatccgg cttttttgat
1081 tcaatcggcg tttatcaaca agtaccgagt gccttacagc taaaagcaat ggaatggctg
1141 gaacgcttgc atttagccaa tctggcgaaa aaaccgttcc gttcactttc gtgggggcaa
1201 caacggttat tattgattac tcgtgctatg gtaaaacacc cgccgattct gattttagac
1261 gaaccgctgc aaggtttgga cggtgtaaac cgcaaattgg ttaaacaatt tatcgaacag
1321 cttgtgacta atagtcaaac ccagttgcta tttgtttcgc accaagatgc ggacgcccca
1381 aattgtatca cccatttatt tgaatttgtt ccgcaaacta acggtggtta ccgttatgtg
1441 cagacggcgt taaattaggt ttttgacctt taaaggaaat ccccctcttt agtaaagagg
1501 gattagggga gatttgtcaa tagagagata tgaaattgaa tagaacttca tttttatat
1561 ttataaaagc gttaattagc atatttcttc gctaattcat tctgtcaaat ctcctcctgc
1621 ccctctttgc taaagagggg agatatgtgc gggactttaa agcgttgaat tatagaactt
1681 ataagcctcg tccatattct caaatttata cataatccct ttatctaata ccattgcatt
1741 atcgcaataa gacttcattg ctgacggact atgcgaaacc aaaataatcg aacgatcttt
1801 gcgcttttca aataattcat gtttacattt tgccgcaaag cgagagtcac ctaccgcaat
1861 tacctcatca attaagtagc aatcaaactc taccgaaagc gacaaagcaa aggcaagtcg
1921 ggctttcatg ccggaggaat atttcttaac cggctcatat aaataatcac ccaattcgga
1981 aaattcttcg gtaaaggctt taacgtattc aatatccgca ttatatatac ggcaaataaa
2041 gcgtaaatta tccataccgg ttaaactgcc ttggaacgcc ccgctgaaag cgagcggcca
2101 agatatcgac atattacgtt cgatagtacc tgatgttggc ggctcaacac cacttaacaa
2161 acggattagc gttgatttcc ctgcaccgtt acgccctaaa ataccgattt tctcgccttt
2221 tttcagctca aaattaatat cttgcaatac ggttttttta ccgcttcgag tatagtaatc
2281 tttactcaca ttttttacgc taatcattgc ggttcgattc ctttactgaa gtttttacc

2341 atgattaagc cgattaataa catggctaga tcacatatta cgagataact tatgctttca

2401 taagtgataa tgttgtcacc gaaataaccg tggcgaaaca tttccgtacc gtgaatcatt

2461 ggcaataagg tcgcatattg ttgcgcttgg cttggcagag cgtggacaaa gaaaaaagca

2521 ccggaaagcg gtaaaaggac aaaacttaat gtaccccaaa ttttaccgaa agcttcaaat

2581 ttttgtgcaa tggagcaaat gatcaagccg agccctaaag caaagaatgc cattaatacc

2641 catgctatta tcatatagaa cgtatctttc ggcatatcaa tccaacccaa taatattaat

2701 aatgccataa taatgatttg ggcaattgtt gcacctgcta cttccagtag gactcgagcg

2761 agtaaagtat ccaatacacg tacgttacga tgataaagaa gactcaaatt tcctgatatt

2821 gctccgatag ttctatttga agcattacgc cacatcattg ccatcggata accggtaatc

2881 acaaaagcaa taatatttaa atccgaaacg cgatccgctc ggataaactt ccacatcagt

2941 acaataaata aagtgagtaa tagcggctca acaaacagcc acaaaaagcc taagttcttc

3001 cgtccgtagc gagtaataat ttctcgcata agtaaagcgc cgattactct tccttgaatg

3061 gcaagtgact ggcggaaggt tgcttgatca ccgtattgca ttagttttta tgctctctta

3121 cacttgcgat taataagctt aatacgccgt acagcatcag tccgataaag aatgtcgcta

3181 aaatattata taagcgataa ggttcttccg cccagtccgg tttgcttggc tgactgatta

3241 cttctaaata aagctgctga cgatccgctt cgtttttgt attttgcaaa gaagttaagg

3301 ctgcggtcaa ttgctgttgc gctaattcgt ttgctaatac taaacgttga taatcggcag

3361 tttgagtagc aatcgagcta ttactgttac cggaaagctg ttttgattgc tcgtcaattt

3421 ctttacgtaa gcttttttgg cgcattaata acgcatcaac ttgcgggtta tccggcgtaa

3481 tcgattgtaa ttgcgctaat tgggtttcca cacgaatcaa ctcgcttttt aagcttgaaa

3541 ttaatgaaag ttgtacgccc gattgtgccg gcaaatcaaa aattttattt ttgatacggt

3601 atttacttaa agcatttgcc gtttcgttta cattattttc cgcttctgta accgcttgtt

3661 ccgcaaatga aatggtatct tttcttgcac gttcgtttaa acggttaata agcgtttcac

3721 cttcggcaag gagtttttga ttaatttgtt gcccctcttc cgcgttaaat gctcgaatac

3781 gtaagctggc gataccggaa acagagtcaa agtccacact taagcgatct cggaaatatt

3841 tataaaacgc ttctttactg ttatttaatc cgaatccatt aaagcgagcg ataatatcgc

3901 cctgattttc ataatattcg cgaacaggta atccttgcat tagttgctca agagcggtac

3961 gagaacgcat atattcctgt acggtataag tatcgtcttg cgaacgagaa aaacctgaac

4021 cttgcaataa ggcgcctacg cctgtcagag cggtctgatt tttaggcgat ctaacgacaa

4081 aacttgattc cgaaatataa atatcagaag cgaccgaacc gaaataaaac gctgatagca

4141 cggtagggat tgcgacagtt atccaaaata atggattgag ttttttaaag cggctttttt

4201 tctgtttaac cggtttctgt aatttttctg ctggactggt agcaataggt gtttccatct

4261 tttgtcctta tacattcaat atattaatag gcacgaacgg cattggtcgt actggtaacc

4321 ggcgaagtaa ttgagaaaat cattctcaag aatttttgga attcagacaa cggcgcattt

4381 gaaacataca caatatcttt atcttgcatt gggaaacgct gtaataaaaa catggattgc

4441 ggctcaagta agttcacacg ataaaccgtt ggtacatcca ttcctatagc gtagccttta

4501 gcttgccatt gtgcttgttg ttccaaactc aattgtgcaa aaggcacgtg acggaatacg

4561 aaaacccctc tcggatccga acgagtatca attaaaccgc ccatcttacc gatagcttcg

4621 gcaagcgtaa ttcctttact tgagaatttc atttgctggt tgttacccac agcgcctaaa

4681 ccggtaaaac tataaggcgt gtttagcaac gaaacgacat cgccggcacg taacataata

4741 ttttgcgccg gatcggaaat taacgtttcg aatgcgagtg tttttacttc agaaccacgg

4801 gttagcttga ccgtcacatc ttcaatgttt tccgttgttc cgcctactgc agcaaccgca

4861 tctaatacac gttcattatt agcggttaat ggcatacgaa tactattgcc ttgacgaata

4921 accgtaacat cagcagagtt attattcgca attttgacta atgcttgcgg atgattcgct

4981 ttgcgctgta gtgctccaat aatttgagac tgaatcgctt ccggtgtttt gcctgcgaca

5041 cgaatgttac ccacgaacgg cacggtaacc gtaccgtttt gattaaccat ttgtgccggt

5101 aattgcgtta aatgcccgct accttgtccc tcagaactaa aagtaccgcc aaacagcacc

5161 gccggcggag cttcccaaat tgatatttca agtacatcac ccacattgac cgcaccggca

5221 tagcccgcgc tgcctactgt gcctaaaaat ccggaaaatt gttggctttg ctgagtttga

5281 tacaactgtt gaactaaacc gttatccagt tccaccacat ttacttccgg taaggggtta

5341 tccgaacttt gtgaattagc ctctaagatc gcactatggc tagggcctga agttgggagg

5401 cttgagcaag cagaaaggaa caatgttgtt aaagctaacc ctatgattga attatatttt

5461 ttaatttcca tcttgtaaaa aggctctatt gaaaaagtgt gtaaaatgaa tataaaccat

5521 acataattat agatacaatg ctataattat atattatagt ctaatatctc gttttttaaa

5581 acgataacct aaaatattac ttttctatag aagaataggc attattttaa taaatataat

5641 taggtatgaa tgttgatgaa aatagcattt atcgcttgga atagttttca ggtgttacat

5701 ttcaagccct tattacaagc tttaccgtgt gcattattaa ttattgaaaa acggagacgt

5761 agtgtaccaa tctgtaagga tattttgcga gatataaaca ataatatcgc ttatatccgc

5821 catatggata tatatgcaaa aattgatggg aattttgatg ttttagttgc gcaaacggct

5881 tttgaacaac tttatttatt tcgccacaca aaaattgctt tgcttcaata tgggtatgct

5941 aaggagcctc ataactatgg tacttggaga gcattagcag atctaaattt ggtttatggg

6001 agttacgcct atgaacgaat ttcttatttt tctccgactg aaataacagg ctgtccgcga

6061 tatgatttat ggtatttacc ttcatttcat caaaaagcga aagaaaatta tgcgagagta

6121 ttagatacga gtaagaaaac gattgtatat gcaccaagtt ggggagaatt atccagcttt

6181 aaattatata tagaagaaat tacgaaatta tctttatttt acaacgtgtt agtaaaatta

6241 caccataaca cgcttttatt agcaaacaag catcagaatt atgaaaaatt gtatccgaat

6301 ttacattttt tctatgaagg tgaagatctt ctctcactta tttcggtagc cgacattgtt

6361 atttccgatt ttagcggtgc gatctttgac gcaattttct gtaaaaaacc agtaatatta

6421 ttttctatcc cattagtaaa tcaacccaaa ttagataaat ttagtttgga gatagcccat

6481 cgttcggcgt tgggatatga ggttttttcg ccggatcaag tagctataac agtggcacga

6541 gcacttacag agccgaaatt agtagatgaa acgctgtatc aacagctttt tatgcataac

6601 aaggatgcaa cacagcaagt aataaatgct ttacaacagc ttgctgaggg taagtataca

6661 ttatctcaac agcagttata tgtgcgacaa acagaaaaat tattaaatat tgaaaaaata

6721 aagcagcaaa aaaataaaaa acagtctttc aataaaataa gacagatttc taaaagatta

6781 attaaaaaat aatttttcat attttctttt tatatttttt tatttgacaa tacggagttt

6841 aggaatgaaa aaagtattaa cttatggaac ttttgatttg ttacatcatg gacatattcg

6901 tttattagaa agagcgagat cattaggaga tcatctcacc gttgccattt cgacggacca

6961 gtttaatcta ggtaaaggaa aagtatgtgc ttatacttac gaagagagag cacatatttt

7021 aaaagcaata cgttatgttg acgaagtaat tcctgaaact aagtgggaac aaaaaatcga

7081 tgatgtaaaa aatcatgaaa tcgatgtatt tgttatgggg gatgattggg aaggtaaatt

7141 cgacttctta gcagattatt gcgaagtagt ttatttacct agaactcctg atatttcaac

7201 cactcaagta aaaaaaatgc tcgcgaaaaa agatctcgca accggacaaa aacaaattca

7261 cgaaaaagag taatttgtta atgtttcaaa tcctacaaaa gcatttaccg accttgcaaa

7321 gagttctgag ggagggttac tcccagcatt ctttgcttgc ttattggtat gggcttagtc

7381 tacttactgc tcttgaacag gcgaatcatc ctcaagtaag aaaactggct gagaaaatga

7441 tcaataaagg tattaatatc gggcattatt ttttagcaca aagttatttc ttatgtggag

7501 aatatgattt agcggaacaa gcggtcaaaa aaatcaaaaa ttttgtaaaa atacccgaag

7561 ttgttttttt atatgcggac attctcgtta aatgcaaacg taaagaagag gcttggcaat

7621 tattagaaca atgcgcttta cttaataaaa gaaaaaaagt ttggatatat ctggcaaatt

7681 tagtaaatac tattgcggat tttcaacgtt tagaacagca tattgaaaaa gtaagaacaa

7741 cgacacctca cctaaaattt gaattattaa ttcaccaaag aacaaatgca gcattaaggg

7801 ctggtttaac agaaacggca ttagcactaa cagaacttaa ccctttgcca aagcaagcaa

7861 aagtgaagaa aaaaacaacc gcttataatg ataaattagc ggcaattgca ctagcggatc

7921 tcaaaaaagt attagatcac aaaaaaatac ctttctttct gattagcggt actctgctag

7981 gttgtattcg agaaggaaaa ttattagggc atgataaaga tattgatatc ggcgtttggg

8041 ataagtactc ctacgaagaa ttagcaaact gtttatctac atcgggatat ttttatgtag

8101 taccaacccg cacaaatcat ttagtcatgt taagacacgt taatggtatc gcaattgatg

8161 tgtttatcca ttatcgcgaa tctaatgatt attggcacgc aggtgtcaaa ataaaatggc

8221 ataactcgcc attcaacttg gtatatacaa attttctcgg acaacaatat ttaatacctg

8281 aaaattacga tttataccta acagaaaatt acggtgattg gcgtacgcca aaaactcaat

8341 ttgatagtgc atttgatacg ccaaatatgg aagttatcaa tgaagtggaa atgaaggttt

8401 atatatctaa aatacaaaaa taataaaata aggaataaat gaatgaaatt tttgaaaaac

8461 tcttatcata atgtgattgc tcctaaaggg tatcatcgag gattagtttt atacagaaaa

8521 aaacaatgga ctgaagcttt atcttgcttt gaagctgctt atagcacatc tcctttacat

8581 gccaaaaata cctttaagct aggattatgt caccttaaat tagggaattt ctcagaggct

8641 cattcattta ttgctaaagc tcttgaaata gctccatata atacgcattg gagaaagcag

8701 ctccaacagg ctgaacgcca ccttaataac acatactcat ctccacataa aatcactact

8761 gttgtaacta gaatgaaaca aagtggtatt agccagtcta ttggtaccgc tatcagaaaa

8821 acagtactac tcattccatc agactataac catcgagtaa tggctgatat ttcatcattt

8881 atccaatact ataaagataa atttgacgta tatattatat tacgtgaatt acccgaagat

8941 attgtgtata aaaatactca tgtattagtc aagaatggaa cttcatttgg tgaatattta

9001 aaatttactg cagattatgt tattgactca ggcacaatga actatagcta ccgtattact

9061 gatactaata aatgggtttc ggtatggcac ggtattcctt ataaaaaaat gttcgttgac

9121 tttgacatta aaaatctagc aacagcaatc cgctatgatc ttgcctatga cagcatggtt

9181 tcaatgtcta atttttatac tgatacattt ttacgtaaag caatgcgcta tgacggtgaa

9241 atattacaac ttggttgtgc caaaattgat aatttatttt catctatttc tacaagcaac

9301 gccgataaag tcaatgcctt acgtaatgaa ttaggtttac ctaataataa aaaggttatt

9361 ttatatgctc ctgaatttag agaagtaggt gaactttatt tcccatttga tcctaataaa

9421 ttattatctc atttaggtga agagtattgt ttacttactt tattaccttt taaaggatat

9481 atcgaacaag cagaaaataa tatctactat atttccgatt tagataataa ggatgccctt

9541 ttaattgctg atttattaat tagtgattat cacgaattaa tctatacatt tgatagatat

9601 aataagcctg cagttctaat acagtacgac tatgaatcat ttgtaaaaca acatacttcc

9661 agaaaacaag agctagaaat actagcatct agaaaatatg ttgcaaaaga agcgaacgaa

9721 ttatatcaat ttaattggaa tctactaaaa agatatagta aacagtctac tttacccgaa

9781 tatcttgata gctcatatat aaagcataaa ttaggtattc catttgataa gaaaattgtt

9841 ttatatgcac caactttccg caaagctggt gccgtacaat tgccttttga cccaaataca

9901 ttactaaatt acttagataa tgattatgta ttgatcacaa aaatgcatta cctaaattat

9961 ttagctaata catataacgg cgttattgac tgtacctcac atgaaaatat ggcagagcta

10021 atgaaaatcg ctgatatctt aatcagcgac tattcatcat tagttcttga cttcgctgta

10081 ttaaataaac caatcattct attccagtat gattatgatg aatatatgaa acaacgagga

10141 gtttacttca attttggaga ttatttacct aaagagcaaa ttattcgaac tgaatttgaa

10201 ttatatacat taaactggaa taagcttaac tcggacaata gtaagattat taatgaattt

10261 tatccacttg aagatggaaa atccacccag cgtattgtag ataaaataaa ttttaatgca

10321 gatttaagat tcagcaagga tattattttc ttagtaaatg atttaaatca aattggtggt

10381 gtccactcat ttttaaagaa tatggcaaaa tactataagc agaagtataa ctctcgcatt

10441 tatgttattg ctattaaaga atttgcagaa gcaaactctg aatatcactt attagaaagc

10501 gaatatattg actttaaatt atctagccaa tatttaaatg gtgcttgtgc taatatttta

10561 caaaatacag atggcattgt aatttcatta cagttttctg ctcatatgta tttccaaaaa

10621 tatttaacca acgctaaatc agttctgatg ttccatggcg atgttaaaga tatgatttcc

10681 agagagcttt atgggccaca tttagattgg ttaaataaag gcaaactcta taactatcaa

10741 aagttattat tgcttacaca atcagcttta gacttactta aacctcattt aaatcctgag

10801 atccaagata aattaggatt tatgcataat tctattgatg aagaatttag tccaattaaa

10861 cagaataaaa aacatcaatt aaatactgcg gtaattagcc gtctagatgc agataaaaat

10921 atttttgcaa tgattgacct tggaaaagag attcttgctc aaaattcaaa tgttgttgta

10981 aatatctatg gtgatggagc tttaaaagat gaatttatag ctgaaattac tcgtcatggt

11041 ctagaacata ttttaaaagt aagaggcttt gaaagtaata aatctaaaat tttctcagaa

11101 aataattcct tattattaat gagtaaatca gagggctttc ctcttgtttt actagaagcc

11161 tatgcttatg gcaagcctgt gattgttttt gattcattta cagctgctaa agaaattgta

11221 aaacataatc agtcaggatt tttattacct tacggcgatt atgggaatgt agttaaagcc

11281 attgagaata gtaaaaatat taagttaaag gatattgaaa tgatctttaa caatttctct

11341 aatccaactg tgtttgctaa atgggatagc ttaatcctag ctttagagca aacagcgtaa

11401 aggagataca gtatgaaact attaaaaaaa ttattcggcc gcaataaatt agaacagcct

11461 ttaatttcga ttcttgtacc ttgttacaat tctcgtaaaa cattgcctgc aacattaaaa

11521 tctattcaac aatcaaatta taaaaactta gatgtaatga ttgttgatga tgggcatgag

11581 gtgactgtag aggatattgt tagttcattt aacgatcctc gctttcgtta tttttataaa

11641 aaaaatgaag gcttaggctt aactagaaat tttggtattg acaatgcgaa aggcgaattt

11701 atcttctttt tagactcaga tgatctaatt tatcctgatg ctttctcaaa tttaataaac

11761 tatatgctgg aaaacaattt agatgtggta tctggtgtta cagtacgtcg cgactttgaa

11821 actaatgtag aaagtgaatg gtgtagggca ttatatagat ctaaaaaaat tagtacattt

11881 gaaaatagat tatccttatt tgatgatgct ttatctacaa ataaattata ccgattatcg

11941 atgttaagag aaaaagatat tcgttttgaa actggattat atgaagataa agtttttact

12001 gcaaaattat attctctagt agatcgtata gggttaattg ataatagggt atatatctgg

12061 tttatatatg gatcacaaac aagtatatct acatcaaaat ccgttagtaa ttttaaggga

12121 agaatggctg ctattaataa tctatggcag tatattcctg aaatgcgaaa gacataccaa

12181 atagcgtttt atatgaacca cgatttatta atttatttac gtgaatttga attttactct

12241 gaagaagaaa agaatgaaat ttacaatatc gcttatgaat tcatacatag gcacaagaaa

12301 tatatctaca atagattaat accaaatagc tggaatagaa cttgtcttga tgcgctatgt

12361 gaaggaaata aagaaaaatt tatttatact gcaaatactc tatcaaaagt attccaagaa

12421 gagttaagta aaaaacagaa ggtctgatat ggaaaattta aactccaatc ttagaaataa

12481 aataaatgaa aaaaatagag ctagactaaa aaacaaagat ttttcaatct tatctagtaa

12541 ttgtaatggc ggtgtaatgc tacatgattt aggattgcga tttaattctc catttattaa

12601 tttatattta aaacctaaag attttatcaa atactgtagt aacatcagtc attatatatc

12661 ttgtgactta aaatttattg ataatgataa atacccaatt gcatatttag atgatgttga

12721 aattagattt ttacattacg cctccaataa agaagccgaa gaaaaatgga tttcaagaac

12781 aaaaagaata aatttagata atttatttat tatgatgact gaaagagatg ggtgcactta

12841 tgatgatcta ctctcttttg atgcattacc aatagagaat aaagtcgtct ttacccataa

12901 atactatcca gatataaagt catcaatata tattcctggc tttgaagata atgagcatgt

12961 tggtgtttta ttagatttta ttggaaaatc tggggaaaga tattacgatt attttaatta

13021 tgttgattgg tttaatggag tagaactttc agaacttaaa agtggagaaa tgtcagaaat

13081 gtttaaaatg tttttatctg aaaaaaataa agtaacacca aataaaaaag caattaataa

13141 aataaactta ttacacattg ataataataa gctattattt attgagggat taaactatat

13201 tgaaggtttt aactcccccg attataccta tttaataaaa aatctgaaaa ttatcaactt

13261 agcaacaaat gttgagtttg aatacccact tggcacagtt cctaaaaaag aaatgtcaaa

13321 tacattatat ggagataaat attttgacta tactgcagca ggtactgcaa cgatgggctt

13381 taaaggtata gatgttaacc atcttgaaga agggttatat gaagttcaga tttctgtatc

13441 tgaaaataaa gaagaacgaa attatcaaaa tatcaacttt actgcaggtc atttagataa

13501 atatgcctca gatgattatt ttgaatatcg tttattcaaa aatcaaaaca agatctattt

13561 agcaaaaaga aagttaatcg gaagaaatcc tatttctgat tattttatta gtattgaaaa

13621 agaatggatc aaaggaaaaa caatgcatat tgaaggtgca tttgtaatcc ctggtattga

13681 tattactgaa tttaatcaag caagatacta tttaattgcc caaaaagcga ttacacagaa

13741 gcaatactcc tttgcattag gacagatcaa aaaagctgga ctaggagaaa aaatcaataa

13801 tccacaaggt tcttataatg cctgctacta tgcaacaaaa atgctaaaag gtattgatat

13861 gagtgcgcta gaatttggat tctatgattt atatatttct ttaagctaca aaagcgaagt

13921 ctttactgtt aaattaaata aacagttaga aattggacat caactcctga aattagttga

13981 taacattgag gagtaggctc ttatcttaac gaaagatagt tattactctt cctaagagac

14041 ttcccaaaaa agcggtcaaa tttttctaaa aaattgcaaa gtttaggaaa aataagaccg

14101 cttattttat tcatactctt atataaaatt agagtttatt atgggcgtag ccacgtacaa

14161 tggtaaggat atgctgtata gcaatattaa tacaatccta tcaatttcat attatcttaa

14221 tcagatttat tttttcgttt tttagacatt aagctgttat tttcttcgtt atttaattct

14281 gccaaatctc ccctatcccc tctttactaa agaggggggat ttcctttaga tattaacaag

14341 ctatgatctt tcgaatagct ttggtctctc aatagattca ggtgaatgat cgatatataa

14401 ggaaaataaa aacattcgtc gaagacgagc gccatcgtaa agaaatttgg agccacagca

14461 tggctcactc agccgtaggc tgatcgtaac cacgtacggc ttgccgtgcg tggctgttgg

14521 gtgattattt aaaaacaggc gaaacggctt ttttactttt tcattaccgc aatttgtttt

14581 agaatcaacg gacgatttac tctcaaggat ttatgatgaa aaaattactt ttggcggtgt

14641 taatcgcatc gttcgggttg gctgcctgcg gtgtaaaagg cccgctctat tttcccgagc

14701 agcaaccggc tcaacaacaa acaaaataat tgctaaccac ggtaattcaa aatctacgta

14761 taacaagcgg tcggatttat ccgatttttt gtaaatacaa gtcatcaatc aatacggtac

14821 accggcttat atctattctc gtgctacgct tgagcgtcat tggcacgcct cggttcgggc

14881 tttgatattg tgtcgcaagg cgaacttgaa cgtgtacttg ccgccggcgg cgagccgagc

14941 aaagtggtat tttccggtgt ggcaaaatca catagcgaaa ttcagcgtgc attggaagtc

15001 ggcattcgtt gctttaatat cgaatccatc gccgagttac accgcattaa tgaagttgcc

15061 ggtcaattag gtaaaatcgc accgatttca ttgcgtgtaa atccggatgt ggatgcacat

15121 actcaccctt atatttccac cggtttaaaa gaaaataaat ttggggtaag cgtaacgaac

15181 gagggaaaca agtactggga gaagcgttac gagaaaaaac acaggcggaa aacctattgc

15241 gtgaaagtgc gctaaattgg acgattctcc gcccgtgcgg gctgaatacg gacgaaggcg

15301 aaacctttcg tttaattgaa aatgcggctg aactgcccgg cagttatatg agccgtaaag

15361 cattagccaa tgcggtcttg tccgtactta acagtgaaaa cacaaactat aaaatcttct

15421 cagtctgtgc ctaacttcac aatccctttc actttggcac aagccatccg cttgtgccta

15481 tttcaaaatt tgttttaaca gttccaataa aaccggtaat tccgttactc cctctcgatc

15541 gacaaaatgc ccgcccgttg ccaagcgaat ataatccgct tgtaagtatt gcgctaatcg

15601 atcgctgaac gaatggggaa cgacaacatc atttaatgca tatatgacgt aagacttttg

15661 cggtaaacaa gcggtctgat ttgcataaaa atctgcaaag ctatctaatt ccggcaaagt

15721 tggtaatttc tcataaaagc cagaaacaaa aattgccgtt tttacttttt gctgcgttac

15781 cgcaagataa ttcagtaacg caatgcagcc caaactatgt ccgatgagta aggtattttc

15841 atctaattga agtgtatttt ggtgatgttc cagccatgct tgcggattcg gctgatcgga

15901 attcggcatc gctaaacatt cacattccca tcctaatttt tccaattcgt ttttaagcca

15961 cggaaaccaa tttcttgtcg ggttcgccgt ataaccgtgc gttacatata cttttttcat

//

LOCUS **MG780423.1** 13428 bp DNA linear BCT 16-MAR-2021

DEFINITION Actinobacillus pleuropneumoniae serovar 18 strain 7311555 capsule locus gene cluster, complete sequence.

ACCESSION MG780423

```
VERSION     MG780423.1
KEYWORDS    .
SOURCE      Actinobacillus pleuropneumoniae serovar 18
  ORGANISM  Actinobacillus pleuropneumoniae serovar 18
            Bacteria; Proteobacteria; Gammaproteobacteria; Pasteurellales;
            Pasteurellaceae; Actinobacillus.
REFERENCE   1  (bases 1 to 13428)
  AUTHORS   Bosse,J.T., Li,Y., Sarkoezi,R., Fodor,L., Lacouture,S.,
            Gottschalk,M., Casas Amoribieta,M., Angen,O., Nedbalcova,K.,
            Holden,M.T., Maskell,D.J., Tucker,A.W., Wren,B.W., Rycroft,A.N. and
            Langford,P.R.
  TITLE     Proposal of serovars 17 and 18 of Actinobacillus pleuropneumoniae
            based on serological and genotypic analysis
  JOURNAL   Vet. Microbiol. 217 (2018) In press
  REMARK    Publication Status: Available-Online prior to print
REFERENCE   2  (bases 1 to 13428)
  AUTHORS   Bosse,J.T., Li,Y., Sarkozi,R., Fodor,L., Lacouture,S.,
            Gottschalk,M., Angen,O., Nedbalcova,K., Holden,M.T.G.,
            Maskell,D.J., Tucker,A.W., Wren,B.W., Rycroft,A.N. and
            Langford,P.R.
  TITLE     Direct Submission
  JOURNAL   Submitted (11-JAN-2018) Medicine, Imperial College London, Norfolk
            Place, London W2 1PG, UK
FEATURES             Location/Qualifiers
     source          1..13428
                     /organism="Actinobacillus pleuropneumoniae serovar 18"
                     /mol_type="genomic DNA"
                     /strain="7311555"
                     /serovar="18"
                     /note="K locus: KL18"
                     /isolation_source="lung"
                     /host="pig"
                     /db_xref="taxon:2138312"
                     /country="Denmark"
                     /collection_date="2001"
     CDS             1..1473
                     /gene="modF"
                     /codon_start=1
                     /transl_table=11
```

/product="putative molybdate ABC transporter ATP-binding

protein ModF"

/protein_id="AVT42484.1"

/translation="MPNINIQNALFSLAQHNKLSIESLEINTHDFWVIVGGNGSGKTA

FAQALHNSLSLYSGEYQNSFQHIALLSFEQQQKIIEQIFKHRNNDMVSPDDFGLTARQ

IILNGSERTQLCEEYAAKLRIQPLLDRPFIQLSTGESRKVLFCQMLVSEPDLLILDEP

FEGLDQASVAYWQDVMAQLGKQMAVVLISNRFNDIPDCATHIALLDNLQLILQGERQE

IEQQAVYSQLKFAEQNVNAPLPESAAPLIQLPPNTNPFELKNVMIRYGEKTIIDDLTW

TVAPKQHWWIKGPNGAGKSTLLSIITGDHPQSYANYVHLFGRQRGSGETIWDIKKNIG

YVSSQLHMDYRVNCSALDVILSGFFDSIGVYQQVPSALQLKAMEWLERLHLANLAKKP

FRSLSWGQQRLLLITRAMVKHPPILILDEPLQGLDGVNRKLVKQFIEQLVTNSQTQLL

FVSHQDADAPNCITHLFEFVPQENGGYRYVQTALNQIENA"

CDS complement(1684..2334)

/gene="cpxA"

/codon_start=1

/transl_table=11

/product="capsular polysaccharide export protein CpxA"

/protein_id="AVT42485.1"

/translation="MISVKNVSKDYYTRSGKKTVLQDINFELKKGEKIGILGRNGAGK

STLIRLLSGVEPPTSGTIERNMSISWPLAFSGAFQGSLTGMDNLRFICRIYNADIDYV

KAFTEEFSELGDYLYEPVKKYSSGMKARLAFALSLSVEFDCYLIDEVIAVGDSRFAAK

CKHELFEKRKDRSIILVSHSPSAMKSYCDNAMVLDKGIMYKFENMDEAYKFYNSTL"

CDS complement(2331..3128)

/gene="cpxB"

/codon_start=1

/transl_table=11

/product="capsular polysaccharide export protein CpxB"

/protein_id="AVT42486.1"

/translation="MQYGDQTTFRQSLAIQGRVIGALLMREIITRYGRKNLGFLWLFV

EPLLLTLFIVLMWKFIRADRVSDLNIIAFVITGYPMAMMWRNASNRTIGAISGNLSLL

YHRNVRVLDTLLARVILEVAGATIAQIIIMALVILLGWIEMPKDTFYMVMAWVLMAFF

ALGLGLIICSIAQKFEAFGKIWGTLSFVLLPLSGAFFFVHALPSQAQQYATLIPMIHG

TEMFRHGYFGDSVITYESISYLVICDVAMLLFGLIMVKNFSKGIEPQ"

CDS complement(3128..4285)

/gene="cpxC"

/codon_start=1

/transl_table=11

/product="capsular polysaccharide export inner membrane

protein CpxC"

/protein_id="AVT42487.1"

/translation="METPIATSPAEKLQKPVKQKKSRFKKLNPLFWITVAIPTVLSAF

YFGSVASDIYISESSFVVRSPKNQTALTGVGALLQGSGFSRAQDDTYTVQEYMHSRTA

LEQLMKDLPIREYYENQGDIIARFNGFGLNNSKEAFYKYFRDRLSVDFDSVSGIASLR

IRAFNAEEGQQINQKLLAEGETLINRLNERARKDTISFAEQAVKEAENNVNATASDLS

KYRIKNKIFDLPAQSGVQLSLISSLKSELIRVETQLAQLQSITPDNPQVDALLMRQKS

LRKEIDEQSKQLSSNSNSSIAIQTADYQRLVLANELAQQQLTAALTSLQNTKNEADRQ

QLYLEVISQPSKPDWAEEPYRLYNILATFFIGLMLYGVLSLLIASVREHKN"

CDS complement(4311..5495)

/gene="cpxD"

/codon_start=1

/transl_table=11

/product="capsular polysaccharide export protein CpxD"

/protein_id="AVT42488.1"

/translation="MKLIKLRLLLSLGLVASLAACSSLPTSGPSHSAILEANSQSSDK

PLPEVNVVELDNGLVQQLYQTQQSQQFSGFLGTVGSAGYAGAVNVGDVLEISIWEAPP

AVLFGGTFSSEGQGSGHLTQLPAQMVNQNGTVTVPFVGNIRVAGKTPEAIQFQIIGAL

QRKANHPQALVKIANNNSADVTVIRQGNSIRMPLTANNERVLDAVAAVGGTTENIEDV

TVKLTRGSEVKTLAFETLISDPAQNIMLRAGDVVSLLNTPYSFTGLGAVGNNQQMKFS

SKGITLAEAIGKMGGLIDTRSDPRGVFVFRHVPFAQLSLEQQAQWQAKGYAIGMDVPT

VYRVNLLEPQSMFLLQRFPMQDKDIVYVSNAPLSEFQKFLRMIFSITSPVTSTTNAVR

AY"

CDS 5729..6841

/gene="cps18A"

/codon_start=1

/transl_table=11

/product="capsular polysaccharide phosphotransferase

Cps18A"

/protein_id="AVT42489.1"

/translation="MNKMNRKFSKLLKNPHIFFRDFLNKKYPIKNTELPFSESEEANL

IEANQKLDKIIQKNTLQQTNIDVVFTWVDGSDPSWQAKYSQYAPNYQAKSALYATDIA

RFEDHNELYYSVHAVLKYMPWVRHIFIITDNQKPKWLDETRQEKITLIDHQDIIDKEY

LPTFNSHVIEAFLHKIPNLSENFIYFNDDVFIARELQAEHFFQANGIASIFMSEKSLT

QMRDRGTITPTLSASEYSIRLLNKYYNTNIDSPLVHTYIPLKKSMYELAWQRYEKEIL

GFLPNKLRTNNDLNFANFLIPWLMYFEGKAMPKIDICYYFNIRSPNALTQYKKLLNKK

NIGEQPNSFCANDFNSQKSINNYQNQLFSFLNSYYS"

CDS 6852..10196

/gene="cps18B"

/codon_start=1

/transl_table=11

/product="capsular polysaccharide biosynthesis protein

Cps18B"

/protein_id="AVT42490.1"

/translation="MNKVKRKFRKLLRDPKLFFSDMYLKHNSKLKKFDTRKHTSQYQY

AIISAVYNAEKYLNDYFHSIVNQSLDFEKHIHIICVDDGSTDSSAEIIKSWQQKYPKN

ITYIYKKNGGQGSARNVGLKHLKADWVDFMDSDDFLDKDYFYHVDMAVTNNAKINLVC

CNQIYYFEDKNQYIDRHPLNYRFKTKDNIVPCNDLRKNLQFSAALSFFRVKDIPSDLI

FDEELKPTFEDGKFVNTFLLNQKEDSFVYFQPKSRYLNRKRADKSSTMDGVWQHKGQF

STVFERGYLDILSKCQSEKGYIPKHLQRTVLWEMLRLVKQFLNHEERLDFLTINEKEK

LLFLMDETFKYIEQDTIMNYELGNCGFSRQLGMLGCFKKLDVNRQVAYIDQVDQKNKL

FLLRYFSCFDGDSLITIDDNELIPRFYKKSERKFLSRTFLVEHRLWLPIVDNINGILQ

INIDGKLTNINYDGKRYHNGLPYEKITTNNVVSSSKKDYLVFMDRDDFAGDNAEFFYE

YVQRNHPQLPICFILNKSSPDWVRLKKKGFNLIKHNSKHHLNALKQASALISSQIGGI

TEPFKDLRREYKIIFLQHGVIKDDLSGWLNNVHMDMMLTSTHQEYNSIARNYSPYIYG

EKEIKLTGLPRFDSLYQHKDKFKNQIMVMFTWRKSIAGTFIDNSKSEREFNKQFTETD

YYKKINGFFNDPKLAYVNKKYGTEFVFCPHPNMKPYLKLFNLPSYIKAIDDNLRIHDV

INDSAMVITDFSSIAFDFAYQNKPVCYYQFDKKAFFSGEHTYTKGYFDYDRDGFGPVY

EELENVSRFIITTVNSNYKNQAIYTKRVTNTFKQRDDRNCERVYDEIHKMLHKKHILN

NDVEENYLSNRAKKSFNANNWNAVLFRYNHLVKITRSPERRLKYQYRYIKGLIHTGKY

SLANDQLTIFEQQVISLNNKYYYKVENLKALLFFYTANFYQAKKLWRNNISTLTNGDL

INYARSLFLTGDSLELSKLNVSKLNMNEKRIIELLIYNSDNIDHILLDNLLENNLSNN

DKKLLNFDLIQAEICYMKGKNYLAMKKLTEFETYSKDPMRRLLIALVSDQLGNRKKVK

KQIEALNEPISLFSQEIFELYYKSVN"

CDS 10207..11412

/gene="cps18C"

/codon_start=1

/transl_table=11

/product="capsular polysaccharide biosynthesis protein

Cps18C"

/protein_id="AVT42491.1"

/translation="MNNTEVINGIKITYKYQKRKYDTKHVIFIFSGFGGERGITYDFE

NALAHCPAHIIWIQDSFENAPSYYWCINMDFSYEEAISKFIEHKLEELDLTVNNCTFA

GFSKGGSAALYYAIKHNIDNIVITVPQMNVGSYVSNHWKRIAKHMMGNITEKNIHILD

RKLITALENDSLTNRNVYLFSSESDIQYATEVKPYLDRFEKYQNFNLFMANSLLVTEH

KLVTSYHVPLILGIFYSLAQGAIPHYGICTLSGDRTRGIVPEKPAPVTVLKRFKFSDK

LFFPEGLAYMKGVPCAKYGDIQTKLIISNLQNKFIFNLAKDHKPNLTKELYNDSFVNY

DKGWFCTLKYSGISLEEILPYKGTYQLSIHIFTKYYDAIANLIRNMKIWYLMKICTLS

YSPKEMLLI"

CDS 12024..12137

/codon_start=1

/transl_table=11

/product="putative small periplasmic lipoprotein"

/protein_id="AVT42492.1"

/translation="MKKLLLAVLIASFGLAACGVKGPLYFPEQQPAQQQTK"

CDS 12268..12663

/gene="lysA"

/codon_start=1

/transl_table=11

/product="diaminopimelate decarboxylase"

/protein_id="AVT42493.1"

/translation="MARLGSGFDIVSQGELERVLAAGGEPSKVVFSGVAKSHSEIQRA

LEVGIRCFNIESIAELHRINEVAGQLGKIAPISLRVNPDVDAHTHPYISTGLKENKFG

VSVTNEGNKYWEKRYEKKHRRKTYCVKVR"

CDS complement(12886..13428)

/gene="ydeN"

/codon_start=1

/transl_table=11

/product="putative hydrolase ydeN"

/protein_id="AVT42494.1"

/translation="MKKVYVTHGYTANPTRNWFPWLKNELEKLGWECECLAMPNSDQP

NPQAWLEHHQNTLQLDENTLLIGHSLGCIALLNYLAVTQQKVKTAIFVSGFYEQLPHL

PELDEFANFYTNQTACLPEKSYVIAALNDVVVPHSFSDRLAQYLQADYIRLATGGHFV

DREGVTELPVLLELLKQILK"

ORIGIN

1 atgccaaata tcaacatcca gaacgcctta ttttcccttg ctcaacacaa taaactctcg

61 attgaatcac tggaaatcaa tactcacgat ttctgggtga ttgtcggcgg taacggctcg

121 ggcaaaaccg ctttcgccca agcgctacat aattcacttt cgttatattc gggtgaatat

181 caaaatagtt tccagcatat cgctttactt tccttcgagc agcaacaaaa aatcatcgag

241 caaatcttta aacaccgtaa caacgatatg gtttcaccgg atgatttcgg tttaaccgcc

301 cgtcaaatta tcttaaacgg tagcgaaaga acgcaattat gcgaggaata tgcggctaaa

361 ttacgtattc agccgttatt agatcgcccg tttattcagc tctccaccgg cgaaagtcgc

421 aaggtgttat tttgccaaat gttagttagc gaaccggatt tgctgatttt agatgagcct

481 tttgaagggt tagaccaagc ctcggtcgct tattggcaag acgtgatggc acaactcggc

541 aagcaaatgg cggtggtact gatttccaac cgttttaatg atattcccga ctgtgccaca

601 catattgctt tactggataa cttacaactg attttacaag gcgaacgcca agagattgaa

661 caacaagcgg tctattctca gctaaaattt gcagaacaga atgtgaatgc accgttgccg

721 gagagtgccg caccgctgat tcaactccca ccgaatacta atccgtttga actgaaaaac

781 gtgatgattc gttacggcga aaaaaccatt atcgatgatc taacttggac ggttgcccca

841 aaacaacatt ggtggattaa aggcccgaac ggagcaggaa aatcgacctt actttctatt

901 attaccggcg atcatccgca atcttatgct aactacgtgc atttattcgg tcgtcagcgt

961 ggctcgggcg aaaccatttg ggatatcaag aaaaatatcg gctatgtgag cagccaatta

1021 catatggatt atcgggtgaa ttgctctgcg ttagacgtga ttttatccgg cttttttgat

1081 tcaatcggcg tttatcaaca agtaccgagt gcgttacagc tcaaagcaat ggaatggctg

1141 gaacgtttac atttggccaa tctggcgaaa aaaccgttcc gttcactttc gtgggggcaa

1201 cagcgtttat tattgattac ccgtgctatg gtaaaacatc cgccgattct gattttagac

1261 gaaccgctac aaggcttgga cggtgtaaac cgcaaattgg ttaaacagtt tatcgagcag

1321 ttggtcacca atagccaaac tcagttgcta tttgtttcgc accaagatgc ggacgcccca

1381 aattgcatca cgcatttatt tgaatttgtt ccgcaagaga atggtggtta tcgctatgta

1441 cagacggctt tgaatcaaat agaaaacgcc taagatttaa ccacggaaaa cacggattac

1501 acggagttta atgaatcggt cagttgacgt aatgtttgga taatattgtt ttttcaggtt

1561 tttcttatct cgtaaggaac taataaaact ttaatcggaa actgcaacta ctcgcaatat

1621 cataatttgt taacctttaa aggaaatccc cctctttagt aaagaggggg gatgtgtggg

1681 actttaaagc gttgaattat aaaacttata agcctcgtcc atattctcaa atttatacat

1741 aatcccttta tctaatacca ttgcattatc gcaataagac ttcattgctg acggactatg

1801 cgaaaccaaa ataatcgaac gatctttgcg cttttcaaat aattcatgtt tacattttgc

1861 cgcaaagcga gagtcaccta ccgcaattac ctcatcaatt aagtagcaat caaactctac

1921 cgaaagcgac aaagcaaagg caagtcgggc tttcataccg gaagaatatt tcttcaccgg

1981 ctcatataaa taatcgccta attcggaaaa ctcttcggta aatgctttaa catagtcaat

2041 atccgcatta tagatacggc aaataaagcg taaattatcc ataccggtta aactgccttg

2101 aaacgccccg ctgaaagcga gcggccaaga tatcgacata ttacgttcga tagtacctga

2161 tgttggcggc tcaacaccac ttaacaaacg gattagcgtt gatttccctg caccgttacg

2221 ccctaaaata ccgattttct cgccttttt cagctcaaaa ttaatatctt gcaatacggt

2281 ttttttaccg cttcgagtat agtaatcttt actcacattt tttacgctaa tcattgcggt

2341 tcgattcctt tactgaagtt ttttaccata atgagcccaa aaagtaacat ggctacatca

2401 catattacga gatagcttat actttcatat gtgataacac tgtcgccaaa ataaccgtga

2461 cgaaacattt ccgtgccgtg aatcatcggt attaaggttg catattgttg agcttggctt

2521 ggtagcgcat gcacaaagaa aaatgcgcct gaaagaggta aaagaacaaa gcttaatgtt

2581 ccccagattt tgccaaatgc ttcaaatttt tgtgcaatag aacaaataat caagcctaat

2641 cctaatgcaa aaaatgccat taatacccac gccataacca tataaaacgt atctttcggc

2701 atttctatcc agcctaataa aatgactaat gccataataa tgatttgggc aatcgttgca

2761 cccgctacct caagtatgac acgagccagt aaggtatcta atacgcgaac attacgatga

2821 taaagaagac tcaagttacc ggaaattgca ccgatagtgc ggtttgatgc attacgccac

2881 atcattgcca ttggataacc ggtaatcaca aaagcaataa tatttaaatc ggaaacgcga

2941 tccgctcgga taaatttcca catcaaaacg ataaataaag tgagtaatag cggctcaaca

3001 aacagccata aaaaacccaa attttttcgt ccgtaacgcg taataatttc ccgcatgagt

3061 aatgcaccga ttactctccc ttgaatggcg agagattggc ggaaagttgt ttgatcaccg

3121 tattgcatta gtttttgtgc tctcttacgc ttgcaattaa taaacttaat acaccataaa

3181 gcatcagacc gataaagaat gtcgctaaaa tattatataa gcgataaggc tcttccgccc

3241 agtccggttt gcttggctga ctgattactt ctaaataaag ttgctggcga tccgcttcat

3301 ttttcgtatt ttgtaatgag gttaatgctg cggtcaattg ttgctgtgcc agctcgtttg

3361 caagtactaa gcgttggtaa tcggcagttt gaatagcaat agagctatta ctgttactgg

3421 aaagctgttt tgattgctca tcgatttcct tacgtaaact tttttggcgc ataagcaatg

3481 catcaacttg tgggttgtcc ggtgtaatag attgcaattg agccaattgt gtttctacac

3541 gaatcaattc gctttttagg ctggaaatta atgaaagttg tacgccggat tgtgccggta

3601 aatcaaagat tttatttttg atacgatatt tacttaagtc gcttgccgtt gcgtttacat

3661 tattttccgc ttccttaacc gcttgttccg caaatgaaat ggtatctttt cttgcacgtt

3721 cgtttaaacg gttgatgagt gtttcacctt cggcaagtaa tttttgatta atttgttgtc

3781 cctcttctgc attaaaagca cgaatacgta agctggcaat accggataca gaatcgaaat

3841 caacacttaa gcgatctcgg aaatatttgt aaaacgcttc tttactatta tttaaaccaa

3901 atccattaaa gcgagcgata atatcgcctt gattctcata gtattcacgt attggtaggt

3961 ctttcattaa ctgttctaat gccgtacgag aatgcatata ttcttgtacg gtataagtat

4021 catcttgagc acgagaaaat ccggaacctt gtaataaggc cccgacaccg gttaaagcgg

4081 tctgattttt aggcgatcta acgacaaaac ttgattccga aatataaata tcagaagcga

4141 ccgaaccgaa ataaaacgct gatagcacgg tagggattgc gacagttatc caaaataatg

4201 gattgagttt tttaaagcgg cttttttct gtttaaccgg tttctgtaat ttttctgctg

4261 gactggtagc aataggtgtt tccatctttt gtccttatac attcaatata ttaataggca

4321 cgaacggcat tggtcgtact ggtaaccggc gaagtaattg agaaaatcat tctcaagaat

4381 ttttggaatt cagacaacgg cgcatttgaa acatacacaa tatctttatc ttgcattggg

4441 aaacgctgta ataaaaacat ggattgcggc tcaagtaagt tcacacgata aaccgttggt

4501 acatccattc ctatagcgta gcctttagct tgccattgtg cttgttgttc caaactcaat

4561 tgtgcaaaag gcacgtgacg gaatacgaaa acccctctcg gatccgaacg agtatcaatt

4621 aaaccgccca tcttaccgat agcttcggca agcgtaattc ctttacttga gaatttcatt

4681 tgctggttgt tacccacagc gcctaaaccg gtaaaactat aaggcgtgtt tagcaacgaa

4741 acgacatcgc cggcacgtaa cataatattt tgcgccggat cggaaattaa cgtttcgaat

4801 gcgagtgttt ttacttcaga accacgggtt agcttgaccg tcacatcttc aatgttttcc

4861 gttgttccgc ctactgcagc aaccgcatct aatacacgtt cattattagc ggttaatggc

4921 atacgaatac tattgccttg acgaataacc gtaacatcag cagagttatt attcgcaatt

4981 ttgactaatg cttgcggatg attcgctttg cgctgtagtg ctccaataat ttgaaactga

5041 atcgcttccg gtgttttgcc tgcgacacga atgttaccca cgaacggcac ggtaaccgta

5101 ccgttttgat taaccatttg tgccggtaat tgcgttaaat gcccgctacc ttgtccctca

5161 gaactaaatg taccgccaaa taatactgcc ggcggcgctt cccaaattga aatttcaagt

5221 acatcaccca cattgactgc accggcatag cccgcgctgc ctactgtgcc taaaaatccg

5281 gaaaattgtt ggctttgctg agtttgatac aactgttgaa ctaaaccgtt atccagttcc

5341 accacattta cttccggtaa gggtttatcc gaactttgtg aattagcctc taagatcgca

5401 ctatggctag gacctgaagt tgggaggctt gagcaggcag ccaaactagc aaccagcccc

5461 aaagaaagga gtaatctaag tttgatgagt ttcatctaat ttctcttcaa tatattaagg

5521 aataacaact atataggtat gtcttaaaat ccacataaag attgatttta ataagttacc

5581 taatcaagag aaattaaata taaaaaattt acaaaaaagc aataatgcgt ataaaaaaac

5641 atcatttgca aagaaagtaa atagagagga gggttcaaca gataagcatt ataatccaag

5701 atttatataa aatataattg ataatataat gaacaaaatg aatagaaaat tttctaagtt

5761 actaaaaaat ccacatattt tttttaggga ttttctaaat aaaaagtacc ctataaaaaa

5821 tacggaactt cccttctcag aatctgaaga agctaactta atagaagcaa accaaaaatt

5881 agataagatt atccaaaaga atacgttgca acaaactaat attgatgtgg tatttacttg

5941 ggtagatggt tcggatcctt catggcaagc taaatattcc caatatgcac caaattatca

6001 agcgaaatcc gctctatatg caacggatat cgcccgattt gaagatcata atgaattata

6061 ttattcagta catgctgtac ttaaatatat gccttgggtt aggcatatat ttattataac

6121 agataatcaa aagccaaagt ggctggatga gacgagacaa gaaaaaatta cactaatcga

6181 tcatcaagat attatagata aagaatatct tccaacgttt aattcccatg ttattgaagc

6241 atttttacat aaaattccta atttaagcga gaattttatc tattttaatg atgatgtctt

6301 tattgcacga gaactacaag ctgaacactt tttccaagca aatggtattg cctctatatt

6361 tatgtcggaa aaaagcctca ctcaaatgcg tgacagagga actattacac cgactctttc

6421 tgcttcggaa tatagtattc gcttactaaa caaatattac aatacaaata ttgactcacc

6481 actcgtacac acttatatcc cattgaaaaa aagtatgtat gaattggcat ggcagcgtta

6541 tgagaaagaa attcttggat ttttaccaaa taaattaaga acaaataacg atttaaattt

6601 tgcaaacttt cttattcctt ggttaatgta tttcgaaggg aaagcaatgc ctaaaataga

6661 tatttgttat tattttaata ttagatctcc aaatgcactt acacaatata aaaaactttt

6721 aaataaaaaa aacataggcg aacagcctaa ctccttttgt gcaaatgatt ttaatagtca

6781 aaaaagtatt aacaactatc aaaaccaatt gttttctttt ttaaactcct attacagtta

6841 aggataatat aatgaataaa gtaaaacgta aatttagaaa attactacga gatcctaagt

6901 tgttttttag tgatatgtac ttaaaacata acagtaaatt aaaaaaattt gatacaagaa

6961 agcatactag tcagtatcaa tacgctatta tttctgctgt atataatgca gaaaaatatt

7021 taaatgatta tttccatagt atcgttaatc aaagcttaga ttttgaaaaa catatccata

7081 ttatctgtgt tgatgatggc tctaccgata gttcagcaga aattattaag tcatggcaac

7141 aaaaatatcc taaaaacatt acctacattt ataaaaaaaa tggcgggcaa ggttctgcac

7201 gtaatgtggg gctcaaacat ttgaaagctg actgggttga ctttatggac tccgatgact

7261 ttctagacaa agactatttt tatcatgttg atatggctgt tacaaataat gctaaaatta

7321 atttggtgtg ctgtaatcaa atctattatt ttgaagataa aaaccaatat atcgaccgac

7381 atccattaaa ctatagattt aaaacaaagg ataatattgt tccttgtaac gatctaagaa

7441 aaaatcttca attctctgcg gcactatctt tttttagagt aaaagatatt ccaagtgatt

7501 taatatttga tgaagaatta aaacctacat ttgaagatgg taaatttgtt aatacttttt

7561 tactaaatca aaaagaagat tcttttgtct atttccagcc taaatctcgt tatttaaatc

7621 gtaaaagggc tgataaatcg tcaacaatgg acggagtttg gcagcataaa ggtcaattta

7681 gtactgtatt tgaaagaggc tatctagata ttttatctaa atgtcaatct gaaaaaggtt

7741 atataccaaa acacttacag cgtacggtct tatgggaaat gcttagatta gttaaacaat

7801 ttttaaatca cgaagaaagg ttagactttt taactatcaa tgaaaaagaa aaactcctat
7861 tcctaatgga tgaaacattt aagtatatcg agcaagatac tattatgaat tacgaattag
7921 gtaactgtgg tttttctcgt caattgggca tgttaggttg tttcaaaaaa ctggatgtaa
7981 accgacaagt tgcttatata gaccaagttg atcaaaaaaa taaacttttc ctactaagat
8041 atttttcatg ttttgacgga gactctctca tcactataga tgataatgaa ttaattccac
8101 gtttctataa aaaatcagaa cgtaaatttt tatcaagaac attcttagtt gagcatcgat
8161 tatggttacc tattgtggat aatatcaatg gtattttaca gatcaatatt gacggtaaat
8221 tgacaaatat aaactatgat ggaaaacgtt atcataacgg attaccctat gaaaaaatta
8281 caacgaacaa tgtcgtaagc tctagcaaaa aagactattt agtgtttatg gatagagatg
8341 actttgccgg agataatgct gaatttttct acgagtatgt acaacgaaat catccacaat
8401 taccaatttg ctttatttta aataaatcat cgccagactg ggtgagatta aagaaaaagg
8461 gctttaactt aattaagcat aatagtaagc atcatctaaa tgcattaaaa caagcctcag
8521 ctctaattag ctcacaaata ggtggaatta cagaaccctt taaagatctg cgaagagaat
8581 acaaaataat atttttacag catggtgtaa ttaaggatga cctttcaggc tggttaaata
8641 acgttcacat ggatatgatg ctgacatcaa cccatcaaga atataattct atcgcaagaa
8701 attattcacc atatatttat ggtgaaaaag aaataaaatt aacgggatta ccaagatttg
8761 attctttata ccaacataaa gataaattca aaaatcaaat tatggtaatg tttacttggc
8821 gtaaatctat tgcaggaaca tttatagata attcaaaatc tgaaagggaa ttcaataagc
8881 aatttactga aacagattat tacaaaaaaa ttaatggctt tttcaatgat cccaaattgg
8941 cttatgtgaa taaaaagtat ggaacagaat tcgttttctg tcctcaccca aatatgaaac
9001 cttatttgaa gctttttaat ttaccaagct atataaaagc gatcgacgat aatctacgca
9061 tacatgatgt tataaatgat tctgcaatgg taattactga tttctcatca atcgcttttg
9121 acttcgcata tcaaaataaa cctgtatgct actaccaatt tgataaaaaa gcattttta
9181 gtggcgagca tacctatact aaaggctact ttgattacga tcgagatgga ttcggccctg
9241 tatatgaaga attagagaac gtatctagat tcattataac tactgttaat agtaattata
9301 aaaatcaggc tatatacact aaaagagtaa caaatacgtt taaacaacgt gatgatagga
9361 actgtgaacg agtatatgat gaaattcata aaatgcttca taaaaagcat atcttaaata
9421 atgatgtaga ggaaaactat ctttcaaata gagcaaaaaa atcatttaac gcaaataact
9481 ggaatgcagt attatttcgg tataaccatc tagttaaaat tactagatct cctgagagaa
9541 gacttaaata ccaataccgt tatattaaag gactaattca taccggaaag tatagtttag
9601 ctaatgatca gttaaccata tttgaacagc aagtaatatc attaaataat aaatattact
9661 ataaagtgga gaacctaaag gcattattat ttttctatac tgcaaacttt tatcaggcta
9721 aaaaattatg gagaaataac atttctactt taacaaatgg tgatttaatt aactatgcca
9781 gaagtttatt tttaactgga gattccttag aattatctaa attaaatgta agcaaactaa
9841 atatgaatga aaaaagaatt atagagttat tgatttataa ttcagataat atagatcaca
9901 tcttattgga taacctatta gagaataatt tatccaataa tgataagaaa ttacttaatt
9961 ttgatctaat tcaagctgaa atatgctata tgaaaggtaa aaattattta gctatgaaaa
10021 agcttactga gtttgaaaca tattctaaag atcctatgcg aagactgctt attgctttag
10081 tatccgatca attaggaaat agaaaaaaag ttaaaaagca aatagaggct ctaaacgagc

10141 caatctccct atttagccaa gaaatatttg agctttatta taagagtgtt aactaaaggt

10201 aattttatga ataatacaga agtaattaat ggcattaaaa tcacgtataa atatcaaaaa

10261 aggaaatatg acactaaaca cgtaatcttt atcttttctg gatttggtgg tgaacgtggt

10321 ataacttacg attttgaaaa tgccttagct cattgccctg cgcatatcat atggattcaa

10381 gattcatttg aaaatgcacc ttcctattat tggtgtataa atatggattt ttcatatgaa

10441 gaagctataa gtaaatttat tgaacataaa ctagaagaat tagatttaac tgtaaataac

10501 tgcacatttg ctggtttttc gaaaggggga tcagcagctt tatattatgc tatcaaacat

10561 aatatagata atattgttat tactgtacca caaatgaatg ttggttctta tgtatcaaat

10621 cattggaaaa gaatcgcaaa acatatgatg ggaaatatta cagaaaaaaa tattcatata

10681 ttagatagaa agttaatcac agctttagaa aatgattcac taactaatag gaatgtatat

10741 ttattctcat cagaatcaga tattcaatat gcaacggaag taaaaccata tttagataga

10801 ttcgaaaaat atcaaaattt taatttattc atggctaatt ctttattagt aacagaacat

10861 aaattagtta cttcctatca tgtaccatta attttaggta tattttattc tttggcacaa

10921 ggagctatac ctcattatgg tatatgtact ttatcaggag atagaactcg cggaatcgtt

10981 ccagaaaaac cagctccagt aacggttcta aaacgcttta aattttcaga taagcttttt

11041 ttccccgaag gattagccta tatgaaaggg gttccgtgtg ctaaatatgg agatattcaa

11101 actaaattaa ttatatccaa cctacaaaat aaatttatat tcaatttagc gaaagatcat

11161 aaacctaatt taacaaaaga attatataat gactcttttg ttaattatga caaaggatgg

11221 ttttgcacat taaaatattc cggaatttct ttagaagaaa tactacctta taaaggtact

11281 tatcaacttt ccatacatat ttttacaaaa tattatgatg caatagctaa tttaataaga

11341 aatatgaaga tatggtactt gatgaaaata tgtactttaa gctattcacc caaggaaatg

11401 ttacttatct agaaataaaa taacctagta taggctatgg aacatttcta gctaaacaag

11461 cagtcaaatt tttctaaaaa attgcaaagt ttaggaaaaa taagaccgct tattttattc

11521 atactcttat ataaaattag agtttattat gggcgtagcc acgtacaatg gtaaggatat

11581 gctgtatagc aatattaata caatcctatc aatttcatat tatcttaatc agatttattt

11641 tttcgttttt tagacattaa gctgttattt tcttcgttat ttaattctgc caaatctccc

11701 ctatcccctc tttactaaag aggggaattt cctttagata ttaacaagct atgatttttc

11761 gaatagcttt ggtctctcaa tagattcagg taaatggtcg atatataagg aaaataaaaa

11821 cattcgtcaa agacgagcgc catcttagag aaatttggag ccacagcgtg gctcactcag

11881 ccgtaggctg atcgtaacca cgtacggctt gccgtgcgtg gctattgggt gattatttaa

11941 aaacaggcga aacggctttt ttactttttc attaccgcaa tttgttttag aatcaacgga

12001 cgatttactc tcaaggattt atgatgaaaa aattactttt ggcggtgtta atcgcatcgt

12061 tcgggttggc tgcctgcggt gtaaaaggcc cgctctattt tcccgagcag caaccggctc

12121 aacaacaaac aaaataattg ctaaccacgg taattcaaaa tctacgtata acaagcggtc

12181 ggatttatcc gattttttgt aaatacaagt catcaatcaa tacggtacac cggcttatat

12241 ctattctcgt gctacgcttg agcgtcattg gcacgcctcg gttcgggctt tgatattgtg

12301 tcgcaaggcg aacttgaacg tgtacttgcc gccggcggcg agccgagcaa agtggtattt

12361 tccggtgtgg caaaatcaca tagcgaaatt cagcgtgcat tagaagtcgg cattcgttgc

12421 tttaatatcg aatcaatcgc cgagctacac cgtattaatg aagtcgccgg tcaattaggt

12481 aaaatcgcac cgatttcatt gcgtgtaaat ccggatgtgg atgcacatac tcacccttat

12541 atttccaccg gtttaaaaga aaataaattt ggggtaagcg taacgaacga gggaaacaag

12601 tactgggaga agcgttacga gaaaaaacac aggcggaaaa cctactgcgt gaaagtgcgc

12661 taaattggac gattctccgc ccgtgcgggc tgaatacaac agaaggcgaa accttccgtt

12721 taattgaaaa tgcggctgaa ctgcccggca gttatatgag ccgtaaagca ttagccaatg

12781 cggtcttgtc cgtacttaac agtgaaaaca caaaccataa aatcttctca gtctgtgcct

12841 aacttcacaa tccctttcac tttggcacaa gccatccgct tgtgcctatt tcaaaatttg

12901 ttttaacagt tccaataaaa ccggtaattc cgttactccc tctcgatcga caaaatgccc

12961 gcccgttgcc aagcgaatat aatccgcttg taagtattgc gctaatcgat cgctgaacga

13021 atgggggacg acaacatcat ttaatgcggc aatcacataa gacttttctg gtaagcaagc

13081 ggtttgattt gtataaaaat ttgcaaactc atccaattcc ggtaaatgcg gtaattgttc

13141 ataaaaaccg gaaacaaaaa ttgccgtttt tactttttgc tgcgttaccg ccagataatt

13201 cagtaacgca atgcagccca aactatgtcc gatgagtaag gtattttcat ctaattgaag

13261 tgtattttgg tgatgttcca gccatgcttg cggattcggc tgatcggaat tcggcatcgc

13321 taaacattca cattcccatc ctaatttttc caattcgttt ttaagccacg gaaaccaatt

13381 tcttgtcggg ttcgccgtat aaccgtgcgt tacatatact tttttcat

//

LOCUS **MT468887.1** 17678 bp DNA linear BCT 23-MAR-2021

DEFINITION Actinobacillus pleuropneumoniae strain 7213384-1 capsule gene
locus, complete sequence.

ACCESSION MT468887

VERSION MT468887.1

KEYWORDS .

SOURCE Actinobacillus pleuropneumoniae

ORGANISM Actinobacillus pleuropneumoniae
Bacteria; Proteobacteria; Gammaproteobacteria; Pasteurellales;
Pasteurellaceae; Actinobacillus.

REFERENCE 1 (bases 1 to 17678)

AUTHORS Stringer,O.W., Bosse,J.T., Lacouture,S., Gottschalk,M., Fodor,L.,
Angen,O., Velazquez,E., Penny,P., Lei,L., Langford,P.R. and Li,Y.

TITLE Direct Submission

JOURNAL Submitted (12-MAY-2020) Infectious Disease, Imperial College
London, Norfolk Place, London W2 1PG, UK

FEATURES Location/Qualifiers

source 1..17678

/organism="Actinobacillus pleuropneumoniae"

/mol_type="genomic DNA"

/strain="7213384-1"

/serovar="19"

/note="K locus: KL19"

/isolation_source="lung"

/host="pig"

/db_xref="taxon:715"

/country="Denmark"

/collection_date="2000"

CDS 1..1458

/gene="modF"

/codon_start=1

/transl_table=11

/product="putative molybdate ABC transporter ATP-binding protein ModF"

/protein_id="QSG30232.1"

/translation="MPNINIQNALFSLAQHNKLSIESLEINTHDFWVIVGGNGSGKTA
FAQALHNSLSLYSGEYQNSFQHIALLSFEQQQKIIEQIFKHRNNDMVSPDDFGLTARQ
IILNGSERTQLCEEYAAKLRIQPLLDRPFIQLSTGESRKVLFCQMLVSEPDLLILDEP
FEGLDQASVTYWQEVMAQLGKQMAVVLISNRFNDIPDCATHIALLDNLQLILQGERQE
IEQQAVYSQLKFAEQNVNAPLPESATPLIQLPPNTNPFELKNVMIRYGEKTIIDDLTW
TVAPKQHWWIKGPNGAGKSTLLSIIAGDHPQSYANYVHLFGRQRGSGETIWDIKKNIG
YVSSQLHMDYRVNCSALDVILSGFFDSIGVYQQVPSALQLKAMEWLERLHLANLAKKP
FRSLSWGQQRLLLITRAMVKHPPILILDEPLQGLDGVNRKLVKQFIEQLVTNSQTQLL
FVSHQDADAPNCITHLFEFVPQTNGGYRYVQTALN"

CDS complement(1657..2307)

/gene="cpxA"

/codon_start=1

/transl_table=11

/product="capsular polysaccharide export protein CpxA"

/protein_id="QSG30233.1"

/translation="MISVKNVSKDYYTRSGKKTVLQDINFELKKGEKIGILGRNGAGK
STLIRLLSGVEPPTSGTIERNMSISWPLAFSGAFQGSLTGMDNLRFICRIYNADIEYV
KAFTEEFSELGDYLYEPVKKYSSGMKARLAFALSLSVEFDCYLIDEVIAVGDSRFAAK
CKHELFEKRKDHSIILVSHSPSAMKSYCDNAMVLDKGIMYKFENMDEAYKFYNSTL"

CDS complement(2304..3101)

/gene="cpxB"

/codon_start=1

/transl_table=11

/product="capsular polysaccharide export protein CpxB"

/protein_id="QSG30234.1"

/translation="MQYGDQTTFRQSLAIQGRVIGALLMREIITRYGRKNLGFLWLFV
EPLLLTLFIVLMWKFIRADRVSDLNIIAFVITGYPMAMMWRNASNRTIGAISGNLSLL
YHRNVRVLDTLLARVILEVAGATIAQIIIMALVILLGWIEMPKDTFYMVMAWVLMAFF
ALGLGLIICSIAQKFEAFGKIWGTLSFVLLPLSGAFFFVHALPSQAQQYATLIPMIHG
TEMFRHGYFGDSVITYESISYLVICDVAMLLFGLIMVKNFSKGIEPQ"

CDS complement(3101..4258)
/gene="cpxC"
/codon_start=1
/transl_table=11
/product="capsular polysaccharide export inner membrane
protein CpxC"
/protein_id="QSG30235.1"
/translation="METPIATSPAEKLQKPVKQKKSRFKKLNPLFWITVAIPTVLSAF
YFGSVASDIYISESSFVVRSPKNQTALTGVGALLQGSGFSRAQDDTYTVQEYMHSRTA
LEQLMKDLPIREYYENQGDIIARFNGFGLNNSKEAFYKYFRDRLSVDFDSVSGIASLR
IRAFNAEEGQQINQKLLAEGETLINRLNERARKDTISFAEQAVKEAENNVNATASDLS
KYRIKNKIFDLPAQSGVQLSLISSLKSELIRVETQLAQLQSITPDNPQVDALLMRQKS
LRKEIDEQSKQLSSNSNSSIAIQTADYQRLVLANELAQQQLTAALTSLQNTKNEADRQ
QLYLEVISQPSKPDWAEEPYRLYNILATFFIGLMLYGVLSLLIASVREHKN"

CDS complement(4284..5468)
/gene="cpxD"
/codon_start=1
/transl_table=11
/product="capsular polysaccharide export protein CpxD"
/protein_id="QSG30236.1"
/translation="MKLIKLRLLLSLGLVASLAACSSLPTSGPSHTAVLEANSRNSDK
PLPEVNLVELDNGLVQRLYQTQQSQQFSGFLGTVGGAEYAGAVNVGDVLEISIWEAPP
AVLFGGTFSSEGQGSGHLTQLPAQMVNQNGTVTVPFVGNIRVAGKTPEAIQSQIIGAL
QRKANHPQALVKIANNNSADVTVIRQGNSIRMPLTANNERVLDAVAAVGGTTENIEDV
TVKLTRGSEVKTLAFETLISDPAQNIMLRAGDVVSLLNTPYSFTGLGAVGNNQQMKFS
SKGITLAEAIGKMGGLIDTRSDPRGVFVFRHVPFAQLSLEQQAQWQAKGYAIGMDVPT
VYRVNLLEPQSMFLLQRFPMQDKDIVYVSNAPLSEFQKFLRMIFSITSPVTSTTNAVR
AY"

CDS 5702..6814
/gene="cps19A"
/codon_start=1
/transl_table=11
/product="capsular polysaccharide phosphotransferase"
/protein_id="QSG30237.1"

/translation="MNKMNRKFSKLLKNPHIFFRDFLNKKYPIKNTELPFSESEEANL
IEANQKLDKIIQKNTLQQANIDVVFTWVDGSDPSWQAKYSQYAPNYQAKSALYATDIA
RFEDHNELYYSVHAVLKYMPWVRHIFIITDNQKPKWLDETRQEKITLIDHQDIIDKEY
LPTFNSHVIEAFLHKIPNLSENFIYFNDDVFIARELQAEHFFQANGIASIFMSEKSLT
QMRDRGTITPTLSASEYSIRLLNKYYNTNIDSPLVHTYIPLKKSMYELAWQRYEKEIL
GFLPNKLRTNNDLNFANFLIPWLMYFEGKAMPKIDICYYFNIRSPNALTQYKKLLNKK
NIGEQPNSFCANDFNSQKSINNYQNQLFSFLNSYYS"

CDS 6825..10568

/gene="cps19B"

/codon_start=1

/transl_table=11

/product="CDP-glycerol glycerophosphotransferase family
protein"

/protein_id="QSG30238.1"

/translation="MNKVKRKFRKLLRDPKLFFSDMYFKHSIKIKKHLPVKYEGKHQF
TIVSAVYNVEKYLDDFFDSIVKQNLSFKKHIQIILVDDGSKDSSATIIKKWQKKYPNN
IHYYYKENGGQASARNLGLKYVQTEWVTFIDPDDFLSLNYFLEVDKKLSEHKNIAMIV
CNLLFFMEKKEIITDKHPLKFRFEKDVNCLSIKDLNNNLNLSVATSFFRTSVIQGNQL
LFDNRVKPNFEDGKFISDYLFELQHYNALFLKKPVYFYRKREDGTSTLDTSWQKPEKY
KNVLEYGFIPMLQKYHNKLSYVPNNIQKTALYDMYWYIQYLLNRPEKIRFLSEKDQAK
FYQLYDKVFEYIDVENIMQFNIAGAWFFHKVGMIGAFKNQRPPFQIAYIENIDREKKQ
ILISYFTYFDDCNSFRLNGRDTLPVYQKTVTNTFNEKLFTYEKRSWIPFEKEDDILTI
SLNGLMMRISVKGTLFSKGISINKILSAFTPQAKYLTDGSWLLMDRETKADDNAEHFY
RYMQAHHPEQRCYFVLNKSSIDWQRLKKDKFNLVEFGSIEYERRLEKASKIISSHLEA
HINNYFGDNYDFSKKFIFLQHGITKDDLSQWFNTKKNLSGVITATIPEYNSIVEELNK
YKIGKKETFLTGFPRHDKLLSGNIKGAKTILIVPTWRHYIMGTQIGKGANTRELNKAF
MTTNYAKAWYNLLHSQELKNLIKNLGYKVIFAPHPNIEPYLNEFNIPQYIDVWKSAIS
RESMQSLFQQSNLLITDYSSIAFEMAFLGKQTIYYQFDKEEFRSGIHTYQQGYFEYEK
DGFGPVAETLDALFAHLDKFVKGENDYINIYQSRIQKTFKYRDTNNCQRVYEAIINLD
IPDKYIDKNIILNALKSAYKAQDWSLVISRAKTLLAEIPDHSFAKNVLFEAVIASNNK
VQMQLDYSLLNQQEKSILQAVKCSQKLAWQDVLNHLKGIILSNEQLLVLSLKANAYLH
NAKGTQKTANKLSKIIDKKKQHLFKAWIAFAKQDWVSVIALLENNISGLNKKELELYL
PELLLARAYCQLNAFTSSHNCLVAYEKHSFASPLSRIEIAHLAYAKRNYTKCIDQLNK
CFAKELDNLPTESLAEYALSLLKGNNIEEFEQLVESPLSKKFEQYPLFKQEYVYFLVN
KKAWRKLIEYAQNWALQDKEIFNYPLMLAYYRLGNINYVYHNHIKPMAEHPYEYWKLI
AEAATLYEDLELSKHCYKGMISIYPEYDIKRNQQYFLTLLHKQ"

CDS 10593..12917

/gene="cps19C"

/codon_start=1

/transl_table=11

/product="putative acetyltransferase"

/protein_id="QSG30239.1"

/translation="MNNIYAFWESTKKTPAYLELCQKTWYKHIPNAKIHIINYKNLRE

YIGDTYDLEKLKTIPLAMQSDIISAAVLERFGGLFLDIDCIATNDVFNLFNQIAQDKL

VAFGRPNDCAIHLAVLYCKKPNNPILREWRIEAQKRLENKPEKFGWAYFGNEIINPLL

KSEKYVNDFHIIDRSISGNILESVAIKDSHPSKAIEDYKNFWFNPNFTFSTETLNLVT

CGIISLHNSWTPEQYRLISDETLFLEQDIPMSHLLKHVLVGNKSPTMMSEQLILEGYL

SSELNRKNILFKRKYFRNMLVLDFSINKKQFAFDISVTNKKIKVDLVLRNIPSSEVRK

SSFLESLNFNVNKVTLGVLDTNKQTLDLILKCYAEFINNNLLTSNELKENKFEVIQFI

DDVFIDLENFEIKNNKIYLSGIGFIQNLNVVEWSDIDYKLIFKSKEEKEYIKQLAKLH

KPEITQKYAVDSVKYDKCFFTTFQHNGIDVFDIPFGNYDIYLSITVAGVTKKQKLRTI

HQSILQHPLIRSSTINNGVFQLDTLTFKADFAKNIVLHNKSKQLITESILDTANNRVT

APKNSTNCFIRFEGSNNHIDIDPDANIRNLYIECLGSHNIVKIGKNVSLHGTIRLGFG

CEVNIGDGTSSTNPIYATCAEQTKLLIGRDCMFATNNQIRTDDAHPIYDVNTGKRVNM

SKDIQIGDHVWIGYGATILSGSAIGSGSVIGAGSIVRNKFPNNCVIAGTPAKVVKKDI

FWERPLLLNMSEEVVYSEEERRQKNYCKNTMETE"

CDS 12991..13731

/gene="cps19D"

/codon_start=1

/transl_table=11

/product="CatB-related O-acetyltransferase"

/protein_id="QSG30240.1"

/translation="MNNQFIPTDVTKISYEKISGLLQARKINVYHPIQAPQTIMLVNN

DIKLEGDNNLWGRVTSLWTMGSFSYTGSNLGYGVSIGRYSSLATGLSIMGAHHFPDWI

STSPSFYTNEHHDLIGKDVSNIARSKRRVVIGNDVWIGANVVLKNNITIGDGAIIAAN

SVVIKDVPPFSIVGGNPAKLIRMRFDEDTIKEIQSLKWWRFHRDDLKGLTANKPNEFL

KGLEKRILANEISPYNPKILTLEDFINS"

CDS complement(13994..14626)

/gene="hypothetical"

/codon_start=1

/transl_table=11

/product="putative transposase"

/protein_id="QSG30241.1"

/translation="MDTTHFKQRFAVLVLVDSLSSKPVYFRFIPAEKNQYYFEAISEL

MEKGIKIQSITCDGRRGLLNAYPDIPTQMCHFHQVGRGIFYLTKSPKSPAGKALLELY

YSLKSYTKETLNQALLQWRNEYKTYFNERSEHNAKRFKHKRLRSAYWSLKRSINYLFT

YQDYPELHIAHTTNLVESFFKLMKAKLAPHQGLTDEHKMVFIKDFICQRS"

CDS 16072..16185

/gene="hypothetical"

/codon_start=1

/transl_table=11

/product="putative small periplasmic lipoprotein"

/protein_id="QSG30242.1"

/translation="MKKLLLVVLLTSFGLTACGVKGPLYFPEQQPAQQQTK"

CDS 16518..16913

/gene="lysA"

/codon_start=1

/transl_table=11

/product="diaminopimelate decarboxylase"

/protein_id="QSG30243.1"

/translation="MARLGSGFDIVSQGELERVLAAGGEPSKVVFSGVAKSHSEIQRA

LEVGIRCFNIESIAELHRINEVAGQLGKIAPISLRVNPDVDAHTHPYISTGLKENKFG

VSVTNEGNKYWEKRYEKKHRRKTYCVKVR"

CDS complement(17136..17678)

/gene="ydeN"

/codon_start=1

/transl_table=11

/product="putative hydrolase YdeN"

/protein_id="QSG30244.1"

/translation="MKKVYVTHGYTANPTRNWFPWLKNELEKLGWECECLAMPNSDQP

NPQAWLEHHQNTLQLDENTLLIGHSLGCIALLNYLAVTQQKVKTAIFVSGFYEKLPTL

PELDSFADFYANQTACLPQKSYVISALNDVVVPHSFSDRLAQYLQADYIRLATGGHFV

DREGVTELPVLLELLKQILK"

ORIGIN

1 atgccaaata tcaacatcca gaacgcctta ttttcccttg ctcaacacaa taaactctcg

61 attgaatcac tggaaatcaa tactcacgat ttctgggtga ttgtcggcgg taacggctcg

121 ggcaaaaccg ctttcgccca agcgctacat aattcacttt cgttatattc gggtgaatat

181 caaaatagtt tccagcatat cgctttactt tccttcgagc agcaacaaaa aatcatcgag

241 caaatcttta aacaccgtaa caacgatatg gtttcaccgg atgatttcgg tttaaccgcc

301 cgtcaaatta tcttaaacgg tagcgaaaga acgcaattat gcgaggaata tgcggctaaa

361 ttacgtatcc agccgttatt agatcgcccg tttattcagc tctccaccgg cgaaagccgc

421 aaagtgttat tttgccaaat gttagtcagc gaaccggatt tattgatttt agatgagcct

481 tttgaagggt tagaccaagc ctcggtcact tattggcagg aagtgatggc acaactcggt

541 aagcaaatgg cggtggtact gatttccaac cgttttaatg atattcccga ctgtgccaca

601 catattgctt tactggataa cttacaactg attttacaag gcgaacgtca agagattgaa

661 caacaagcgg tctattctca gctaaaattt gcagaacaga atgtgaatgc accgttgccg

721 gagagtgcca caccgctgat tcaactccca ccgaatacta atccgtttga actgaaaaac

781 gtaatgatcc gttacggcga aaaaacgatt attgatgatc taacttggac ggttgcccca

841 aaacaacatt ggtggattaa aggcccgaac ggagcaggaa aatcgacctt actttctatt

901 attgccggcg atcatccgca atcttacgct aattatgtgc atttattcgg tcgtcagcgt

961 ggttcgggcg aaactatttg ggatataaag aaaaatatcg gctatgtgag cagccaatta

1021 catatggatt atcgggtgaa ttgctctgcg ttagacgtga ttttatccgg cttttttgat

1081 tcaatcggcg tttatcaaca agtaccgagt gccttacagc taaaagcaat ggaatggctg

1141 gaacgcttgc atttagccaa tctggcgaaa aaaccgttcc gttcactttc gtgggggcaa

1201 caacggttat tattgattac tcgtgctatg gtaaaacacc cgccgattct gattttagac

1261 gaaccgctgc aaggtttgga cggtgtaaac cgcaaattgg ttaaacaatt tatcgaacag

1321 cttgtgacta atagtcaaac ccagttgcta tttgtttcgc accaagatgc ggacgcccca

1381 aattgtatca cccatttatt tgaatttgtt ccgcaaacta acggtggtta ccgttatgtg

1441 cagacggcgt taaattaggt ttttgacctt taaaggaaat ccccctcttt agtaaagagg

1501 gattagggga gatttgtcaa tagagagata tgaaattgaa tagaacttca ttttttatat

1561 ttataaaagc gttaattagc atatttcttc gctaattcat tctgtcaaat ctcctcctgc

1621 ccctctttgc taaagagggg agatatgtgc gggactttaa agcgttgaat tatagaactt

1681 ataagcctcg tccatattct caaatttata cataatccct ttatctaata ccattgcatt

1741 atcgcaataa gacttcattg ctgacggact atgcgaaacc aaaataatcg aatgatcttt

1801 gcgcttttca aataattcat gtttacattt tgccgcaaag cgagagtcac ctaccgcaat

1861 tacctcatca attaagtagc aatcaaactc taccgaaagc gacaaagcaa aggcaagtct

1921 ggctttcatg ccggaggaat atttcttaac cggctcatat aaataatcac ccaattcgga

1981 aaattcttcg gtaaaggctt taacgtattc aatatccgca ttatatatac ggcaaataaa

2041 gcgtaaatta tccataccgg ttaaactgcc ttggaacgcc ccgctgaaag cgagcggcca

2101 agatatcgac atattacgtt cgatagtacc tgatgttggc ggctcaacac cacttaacaa

2161 acggattagc gttgatttcc ctgcaccgtt acgccctaaa ataccgattt tctcgccttt

2221 tttcagctca aaattaatat cttgcaatac ggttttttta ccgcttcgag tatagtaatc

2281 tttactcaca ttttttacgc taatcattgc ggttcgattc ctttactgaa gttttttacc

2341 ataatgagcc caaaaagtaa catggctaca tcacatatta cgagatagct tatactttca

2401 tatgtgataa cactgtcgcc aaaataaccg tgacgaaaca tttccgtgcc gtgaatcatc

2461 ggtattaagg ttgcatattg ttgagcttgg cttggtagcg catgcacaaa gaaaaatgcg

2521 cctgaaagag gtaaaagaac aaagcttaat gttccccaga ttttgccaaa tgcttcaaat

2581 ttttgtgcaa tagaacaaat aatcaagcct aatcctaatg caaaaaatgc cattaatacc

2641 cacgccataa ccatataaaa cgtatctttc ggcatttcta tccagcctaa taaaatgact

2701 aatgccataa taatgatttg ggcaatcgtt gcacccgcta cctcaagtat gacacgagcc

2761 agtaaggtat ctaatacgcg aacattacga tgataaagaa gactcaagtt accggaaatt

2821 gcaccgatag tgcggtttga tgcattacgc cacatcattg ccattggata accagtaatc

2881 acaaaagcaa taatatttaa atcggaaacg cgatccgctc ggataaattt ccacatcaaa

2941 acgataaata aagtgagtaa tagcggctca acaaacagcc ataaaaaacc caaatttttt

3001 cgtccgtaac gcgtaataat ttcccgcatg agtaatgcac cgattactct cccttgaatg

3061 gcgagagatt ggcggaaagt tgtttgatca ccgtattgca ttagtttttg tgctctctta

3121 cgcttgcaat taataaactt aatacaccat aaagcatcag accgataaag aatgtcgcta

3181 aaatattata taagcgataa ggctcttccg cccagtccgg tttgcttggc tgactgatta

3241 cttctaaata aagttgctgg cgatccgctt catttttcgt attttgtaat gaggttaatg

3301 ctgcggtcaa ttgttgctgt gccagctcgt ttgcaagtac taagcgttgg taatcggcag

3361 tttgaatagc aatagagcta ttactgttac tggaaagctg ttttgattgc tcatcgattt

3421 ccttacgtaa acttttttgg cgcataagca atgcatcaac ttgcgggttg tccggtgtaa

3481 tagattgcaa ttgagccaat tgtgtttcta cacgaatcaa ttcgcttttt aggctggaaa

3541 ttaatgaaag ttgtacgccg gattgtgccg gtaaatcaaa gattttattt ttgatacgat

3601 atttacttaa gtcgcttgcc gttgcgttta cattattttc cgcttcctta accgcttgtt

3661 ccgcaaatga aatggtatct tttcttgcac gttcgtttaa acggttgatg agtgtttcac

3721 cttcggcaag taatttttga ttaatttgtt gtccctcttc tgcattaaaa gcacgaatac

3781 gtaagctggc aataccggat acagaatcga aatcaacact taagcgatct cggaaatatt

3841 tgtaaaacgc ttctttacta ttatttaaac caaatccatt aaagcgagcg ataatatcgc

3901 cttgattctc atagtattca cgtattggta ggtctttcat taactgttct aatgccgtac

3961 gagaatgcat atattcttgt acggtataag tatcatcttg agcacgagaa aatccggaac

4021 cttgtaataa ggccccgaca ccggttaaag cggtctgatt tttaggcgat ctaacgacaa

4081 aacttgattc cgaaatataa atatcagaag cgaccgaacc gaaataaaac gctgatagca

4141 cggtagggat tgcgacagtt atccaaaata atggattgag ttttttaaag cggctttttt

4201 tctgtttaac cggtttctgt aatttttctg ctggactggt agcaataggt gtttccatct

4261 tttgtcctta tacattcaat atattaatag gcacgaacgg cattggtcgt actggtaacc

4321 ggcgaagtaa ttgagaaaat cattctcaag aatttttgga attcagacaa cggcgcattt

4381 gaaacataca caatatcttt atcttgcatt gggaaacgct gtaataaaaa catggattgc

4441 ggctcaagta agttcacacg ataaaccgtt ggtacatcca ttcctatagc gtagcccttta

4501 gcttgccatt gtgcttgttg ttccaaactc aattgtgcaa aaggcacgtg acggaatacg

4561 aaaacccctc tcggatccga acgagtatca attaaaccgc ccatcttacc gatagcttcg

4621 gcaagcgtaa ttcctttact tgagaatttc atttgctggt tgttacccac agcgcctaaa

4681 ccggtaaaac tataaggcgt gtttagcaac gaaacgacat cgccggcacg taacataata

4741 ttttgcgccg gatcggaaat taacgtttcg aatgcgagtg tttttacttc agaaccacgg

4801 gttagcttga ccgtcacatc ttcaatgttt tccgttgttc cgcctactgc agcaaccgca

4861 tctaatacac gttcattatt agcggttaat ggcatacgaa tactattgcc ttgacgaata

4921 accgtaacat cagcagagtt attattcgca attttgacta atgcttgcgg atgattcgct

4981 ttgcgctgta gtgctccaat aatttgagac tgaatcgctt ccggtgtttt gcctgcgaca

5041 cgaatgttac ccacgaacgg cacggttacc gtcccgtttt gattaaccat ttgtgccggt

5101 aattgcgtta aatgcccgct accttgtccc tcagaactaa aagtaccgcc aaacagcacc

5161 gccggcggag cttcccaaat tgatatttca agtacatcac ccacattgac cgcaccggca

5221 tattccgcac cgcctacagt acctaaaaaa ccagaaaatt gctgactttg ctgggtttga

5281 tacaaccgtt gaactaaacc attatctaat tccactaaat tgacttccgg taacggttta

5341 tccgaatttc gggagttagc ttctaaaacc gcagtatggc tagggcctga ggtcggaaga

5401 cttgagcagg cagccaaact agcaaccagc cccaaagaaa ggagtaatct aagtttgatg

5461 agtttcatct aatttctctt caatatatta aggaataaca actatatagg tatgtcttaa

5521 aatctatata aagattgatt ttaataagtt acctaatcaa gagaaattaa atataaaaaa

5581 tttacaaaaa agcaataatg cgtataaaaa aacatcattt gcaaagaaag taaatagaga

5641 ggagagttca atagataagc attataatcc aagatttata taaaatataa ttgataatat

5701 aatgaacaaa atgaatagaa aattttctaa gttactaaaa aatccacata tttttttag

5761 ggattttcta aataaaaagt accctataaa aaatacggaa cttcccttct cagaatctga

5821 agaagctaac ttaatagaag caaaccaaaa attagataag attatccaaa agaatacgtt

5881 gcaacaagct aatattgatg tggtatttac ttgggtagat ggttctgatc cttcatggca

5941 agctaaatat tcccaatatg caccaaatta tcaagcgaaa tccgctctat atgcaacgga

6001 tatcgcccga tttgaagatc ataatgaatt atattattca gtacatgctg tacttaaata

6061 tatgccttgg gttaggcata tatttattat aacagataat caaaagccaa agtggttgga

6121 tgagacgaga caagaaaaaa ttacactaat cgatcatcaa gatattatag ataaagaata

6181 tcttccaacg tttaattccc atgttattga agcattttta cataaaattc ctaatttaag

6241 cgagaatttt atctatttta atgatgatgt ctttattgca cgagaactac aagctgaaca

6301 ctttttccaa gcaaatggta ttgcctctat atttatgtcg gaaaaaagcc tcactcaaat

6361 gcgtgacaga ggaactatta caccgactct ttctgcttcg gaatatagta ttcgcttact

6421 aaacaaatat tacaatacaa atattgactc accactcgta cacacttata tcccattgaa

6481 aaaaagtatg tatgaattgg catggcagcg ttatgagaaa gaaattcttg gatttttacc

6541 caataaatta agaacaaata acgatttaaa ttttgcaaac tttcttattc cttggttaat

6601 gtatttcgaa gggaaagcaa tgcctaaaat agatatttgt tattatttta atattagatc

6661 tccaaatgca cttacacaat ataaaaaact tttaaataaa aaaaacatag gcgaacagcc

6721 taattcattt tgcgcaaatg attttaatag tcaaaaaagt attaacaact atcaaaatca

6781 attgttttct tttttaaact cctattacag ttaaggataa tataatgaat aaagtaaaac

6841 gtaaatttag aaaattactg cgtgatccta agttgttttt tagtgatatg tatttcaaac

6901 attctataaa aataaaaaaa catttacctg ttaaatatga aggaaaacat caatttacga

6961 ttgtttccgc tgtatataat gtagaaaaat atcttgatga tttctttgat agtatcgtta

7021 aacaaaattt atcatttaaa aaacacatac agattatctt agttgatgac ggctcaaaag

7081 attcatcagc aaccatcatc aaaaaatggc aaaaaaaata tccaaataat atccactatt

7141 attataaaga aaatggtggg caagcctctg ctcgtaattt aggactaaaa tacgtacaaa

7201 cagaatgggt tacctttatt gatccagatg attttcttag cctaaattat ttcttagaag

7261 tagataaaaa gttatcagaa cataaaaata ttgcaatgat tgtatgtaat ctattatttt

7321 ttatggaaaa gaaagaaatt attactgata aacatccttt aaaatttaga tttgaaaaag

7381 atgttaattg tttatcaatt aaagatctta ataataattt aaacttatct gtagcaacaa

7441 gtttctttag aacctctgta atacaaggta atcaactatt atttgataat agagtaaaac

7501 caaattttga agatggtaaa tttatttctg attatttatt cgaattacaa cactataatg

7561 ctttattttt aaagaaacct gtctattttt atcgaaaacg tgaagatggt acttcaactt

7621 tagatacttc ttggcaaaag cctgagaaat ataaaaacgt actagagtat ggttttattc

7681 caatgttaca gaaataccat aacaaactat catatgttcc taataacatt caaaaaacgg

7741 ctctttatga tatgtattgg tatattcaat atctattaaa tagaccagaa aaaataagat

7801 tcctatctga aaaagatcaa gctaaatttt atcaactcta tgataaagta ttcgaatata

7861 ttgatgtaga aaatattatg caatttaata ttgcaggagc ttggttcttt cataaagtag

7921 gtatgattgg agcatttaaa aatcaaagac ctccttttca aattgcatat atagaaaata

7981 ttgaccgtga gaaaaagcaa attcttatta gttactttac ttattttgat gattgtaatt

8041 cctttaggtt aaatggtaga gatacattac ctgtttacca aaaaacagta acaaatactt

8101 ttaatgagaa attatttacc tatgaaaaaa gaagctggat tccttttgaa aaagaggatg

8161 atattttaac tatttcttta aatggcctaa tgatgagaat atctgttaaa gggactcttt

8221 tcagtaaagg tatttctatt aataaaatct tatcagcatt tacaccgcaa gctaaatatt

8281 taaccgatgg cagttggctt ttaatggata gagaaacaaa agcagatgat aatgctgaac

8341 atttctaccg ttatatgcag gctcatcatc ctgagcaaag atgttacttt gttttgaata

8401 agagctcaat tgactggcaa agattgaaaa aagataaatt taatttagtt gaatttggct

8461 ctattgaata tgaaagacga ttagaaaaag caagtaaaat tattagtagt catttagagg

8521 cccatattaa taattatttt ggcgacaatt atgattttag taaaaaattt atatttttac

8581 agcatggtat aactaaagat gatttatctc aatggttcaa tactaaaaag aatttatctg

8641 gagtaattac ggcaactatt cctgaatata actcaatagt agaagaacta aataaatata

8701 aaattggtaa aaaggaaaca tttttaacag gatttcctcg ccatgataaa ttactatctg

8761 gaaatataaa aggagctaag acaattctca tcgtacctac atggcgacat tatattatgg

8821 ggactcaaat tggaaaagga gccaatacac gcgagctaaa taaagccttt atgacaacaa

8881 attatgctaa agcttggtat aatttattac atagtcagga attaaaaaat ttaattaaaa

8941 atttaggata taaagttatt tttgcaccgc accctaatat tgaaccatat ttaaatgagt

9001 ttaacattcc ccaatatatt gatgtgtgga aaagtgcaat atcaagagaa agtatgcaaa

9061 gtttattcca acaatcaaat ctattgatta cggactattc atctattgca tttgaaatgg

9121 catttctagg aaaacaaaca atctattacc aatttgataa agaggaattt agatctggaa

9181 ttcatacata tcaacaagga tactttgaat atgagaaaga tggatttggt cctgtagctg

9241 aaacattaga tgctttattt gctcacctag ataaattcgt aaaaggtgaa aatgattaca

9301 taaatattta tcaatctcgt atacaaaaaa catttaaata tcgagatacc aataattgcc

9361 aacgtgttta tgaggctatt attaacctag atataccaga taagtatatt gataaaaata

9421 ttatcttaaa tgctttaaaa tctgcttata aagctcaaga ctggagttta gttatttctc

9481 gtgccaaaac tttattagca gaaatacctg atcattcatt tgcaaaaaat gtattatttg

9541 aagctgttat tgcttcaaat aataaagttc aaatgcaatt agattattct ttattgaatc

9601 agcaggaaaa atctatctta caggcggtta aatgttctca aaaattagca tggcaagatg

9661 tgttaaatca tttaaaagga atcatacttt caaatgaaca attactagta ttatcattaa

9721 aagctaatgc ctatttacat aatgcaaaag gtacacaaaa gacggcaaat aagttatcaa

9781 aaattattga taaaaagaaa cagcatcttt tcaaagcttg gattgccttt gcaaaacagg

9841 attgggttag tgtaattgca ttacttgaga ataatatctc tgggttaaat aaaaaagaat

9901 tagagctata tttaccagaa ctattacttg ctcgagctta ctgtcaacta aatgctttta

9961 catcctcaca taattgtttg gtagcatacg aaaaacattc ttttgcatct ccattatcaa

10021 gaattgaaat agcacattta gcctatgcaa aacgaaatta tacaaaatgt attgatcagc

10081 ttaataagtg ttttgctaaa gagctagata atctaccaac agaaagttta gcagaatatg

10141 ctctttctct gcttaaagga aataatattg aggaatttga gcaactagta gaaagccctc

10201 ttagtaagaa gttcgaacaa taccctttgt ttaaacagga atatgtttat tttcttgtta

10261 ataaaaaagc ttggagaaaa ctaattgaat atgctcaaaa ttgggcattg caggataagg

10321 aaatatttaa ttatccatta atgcttgctt attatcgatt agggaatatt aattacgttt

10381 atcataatca tataaagcca atggcagaac acccttatga atattggaaa ttaattgccg

10441 aagcggctac attatatgaa gacttagaac tatctaaaca ttgttataaa ggaatgattt

10501 caatctatcc cgagtatgac ataaaacgta atcaacaata ctttttaaca ttattgcata

10561 agcaataatt attatttata gaggtctaaa aaatgaataa catttatgcg ttttgggaat

10621 caactaaaaa gactcctgct tatttagaat tatgccaaaa gacgtggtat aaacacattc

10681 ctaatgcaaa aattcatatt ataaattata aaaatctaag agaatacata ggggatacct

10741 atgatctcga aaaattaaaa accattccat tagcaatgca atcagatatt atttctgctg

10801 cagtgttaga aaggtttgga ggactatttt tagatatcga ttgtattgca acaaatgatg

10861 tatttaattt atttaatcaa attgctcaag ataaattagt tgcttttggt cgaccaaatg

10921 actgcgcaat ccatctagct gtattatatt gtaaaaaacc taataaccct attctaagag

10981 aatggagaat tgaagcacaa aaaaggttgg aaaataaacc tgaaaaattc ggatgggcat

11041 attttggtaa tgaaatcata aatccactat tgaaatctga gaaatacgta aacgacttcc

11101 atattattga ccgttcaatt agtggaaata ttttagaatc tgttgcaatt aaagatagcc

11161 acccatctaa agctattgaa gattacaaaa acttttggtt taatccaaat tttacttttt

11221 caacggaaac actaaatctt gttacttgtg gaattatttc tttacataat tcttggacac

11281 ctgaacaata tcgccttatt agtgatgaaa ctctatttct tgagcaagat attccaatga

11341 gtcatttatt aaagcatgtc cttgtaggaa ataagtcacc gactatgatg tctgaacaat

11401 taattctaga ggggtattta tcttcagaat taaacagaaa aaatatttta tttaaacgga

11461 aatacttccg caacatgctt gttttggatt tttcaattaa caagaagcaa tttgcatttg

11521 acatttctgt tacaaataaa aaaataaaag ttgatttagt cttacggaat attccaagta

11581 gtgaggttag aaaaagctca ttcttagaat ctttaaactt taatgtgaat aaagttacac

11641 taggagtttt agacacaaat aagcaaaccc tagacttgat cttaaaatgt tatgctgaat

11701 ttattaataa caatcttctt acatctaatg aattaaaaga gaataaattt gaagtcatac

11761 agtttattga tgatgttttt atcgatttag aaaattttga aataaaaaac aataaaattt

11821 atttatctgg aataggattt attcaaaacc tcaacgtagt tgagtggtct gatattgact

11881 ataaattaat ctttaaatct aaagaagaaa aagaatacat taaacagtta gctaaactac

11941 acaagccaga aattactcag aaatatgctg tagattctgt taaatatgat aaatgctttt

12001 ttactacatt ccaacataac ggtatcgacg tatttgatat accatttggt aattatgata

12061 tctatttatc cattacagtt gcaggagtaa cgaaaaaaca aaagcttcgc acaatacatc

12121 aatctatttt gcaacaccca ctgattagat catctaccat aaataatggt gtattccaat

12181 tggatacatt aacgtttaaa gctgattttg caaaaaatat tgttttacat aataaatcaa

12241 aacagctgat tacagagtct atactagata cggcaaataa tcgagttact gctcctaaaa

12301 atagtacaaa ttgttttatt cgatttgaag gaagcaataa ccatattgac attgatcctg

12361 atgctaatat tcgtaatctt tatattgaat gtttaggaag tcacaatatc gtcaaaatcg

12421 gtaaaaatgt ctctttacac ggaacaatta gattaggttt tggctgtgaa gtaaatattg

12481 gtgatggaac atcaagtacc aatcctattt atgctacttg tgcagaacaa actaagctgc

12541 ttataggtcg tgattgcatg tttgctacaa ataatcaaat tcgtactgat gatgctcatc

12601 caatttacga tgtaaacaca ggcaaacgag ttaatatgtc taaagatatt caaataggcg

12661 accatgtttg gattgggtat ggagcaacaa ttttatctgg ctcggcaata ggctccggct

12721 cagttatcgg agcaggctca attgtacgaa acaagtttcc taataattgt gttatagcag

12781 gtacccccgc aaaagtggta aaaaaagata tttttggga aagaccatta ctcttgaata

12841 tgagcgaaga ggttgtgtat tccgaagaag aaagaagaca gaaaaactac tgtaaaaata

12901 ctatggaaac agagtaatag gttatatttt taattattat acatcatgag ttaattttta

12961 taactcatga tctccttaaa gtgagttatt atgaataatc aatttatccc gacagatgtg

13021 actaagatat cttatgaaaa aataagtggc ttactacaag ctagaaaaat caatgtttat

13081 catcctattc aagcacctca gacaataatg ttagttaata atgatataaa gttagaggga

13141 gataataatt tatggggaag agtaactagc ttatggacaa tgggtagttt tagctatacg

13201 ggttctaatt taggatatgg agtatctatc ggtcgttatt caagcttagc aacaggtttg

13261 agtattatgg gggctcacca ttttcccgat tggatctcta cctctccatc cttttatacg

13321 aatgagcatc acgatttaat aggcaaggat gtaagtaata ttgctcgtag taaaagacgt

13381 gttgttattg gcaacgacgt atggatagga gcaaatgttg tgctaaaaaa taatattact

13441 atcggcgatg gtgcaattat tgcagcaaat tcagttgtca tcaaagatgt tcctccgttt

13501 tcaatagtag gaggaaatcc agctaagctt attagaatga gatttgatga agacacaatc

13561 aaagaaatac aatcattaaa atggtggcga ttccaccgtg atgatttaaa gggcttaacc

13621 gcaaacaaac caaacgaatt tttaaaagga ttggaaaaac gaatcttggc taatgaaatt

13681 agtccgtata acccaaaaat attaacgcta gaggatttta taaatagtta aacaacttcg

13741 taatggaata ctaaaatagt ttactttatc ttgttttagt tcattatagg tatctgtaat

13801 ttatcgatat agataacatt gttactgatg ttaaagaatt atgcatcaac ttgcattgga

13861 tactattgtt ttattttatc aaaaccaagc ggtcaaattt ttctaaaaaa ttggggatag

13921 tggacaaaat tgagtctaat ttccccatga tatagccata atggacattt ttgagtctgt

13981 tatggcttta tttttaactt cgctggcaaa taaaatcctt tataaacacc atcttatgtt

14041 catcagtcaa tccttggtga ggagctaatt ttgctttcat cagcttaaaa aatgactcga

14101 ctaaattcgt cgtatgtgca atatgcagtt caggataatc ttgataggta aataaataat

14161 ttatgctacg ttttagactc caataggcac ttcttaatcg tttgtgttta aaccgttttg

14221 cattatgctc cgagcgttcg ttaaagtaag ttttatactc attccgccat tgcagtaatg

14281 cttgattaag cgtctctttt gtataagatt taagtgaata gtatagctcc aataacgctt

14341 tcccagccgg agatttgggg gatttcgtta aataaaatat ccctctccca acttgatgaa

14401 aatgacacat ctgagttgga atatcaggat atgcattgag taatcccctc ctaccatcgc

14461 aggtaatcga ttgaatctta atgccttttt ccattaattc tgatatagct tcgaaataat

14521 actgattttt ctcagcggga ataaaacgaa agtagaccgg tttggaagag agtgaatcca

14581 ctaaaaccaa tacggcaaaa cgctgtttaa aatgtgtggt atccatcacg atgttaatgg

14641 tttcgggtaa aggtttttgc tcagctttga tggatttttg aagatgtctt ctgatggttc

14701 tttcagaaca ttgatattca atagtaagct gccgaatggt ttgttttagc gaggtatact

14761 taaaccaaat ttcttgtgaa ttaagtcgtt tggatgcaac aaaattacga tggcaatcga

14821 gacatttata gcgttgaaca ccattaattt tgccatattt tttgaggtta aaagatgagc

14881 agaaaataca attttttagt catttttata aaaagtgggt taaagcctta tagtacaagg

14941 ttttaaccca ttttgtagac tcaattttgt ccactatccc aaaaattgca aagtttagga

15001 aaaataagac cgcttatttt attcatactc ttatataaaa ttagggttta ttatgggcgt

15061 agccacatac aatggtaagg atatgctgta tagcaatatt gatataatcc gagaaagtgg

15121 aagcctgttt ttctccactt acctgtttct gcatttgctg cagtagcaga gaaaaagact

15181 gaacacgcag caattgcgat aagtgctttt gataaaaatg ttttcttcat aaaatgctcc

15241 ttaatgtagc aatacgacaa tgactaaaat gcatacaact ttagattaag taatatgcaa

15301 cttcattttg ttattaattt gtataattga aattagacca cattattact atttttaata

15361 aacaaagaat gagaaattat gtctaaatca ttcgcttaaa ataagccttt tatatttaat

15421 cttattcaaa acttattcaa gatttatata ctccactcaa tttcatatta tcttaatcaa

15481 atttattttt tcgtttttta aatattaaac tattatttac ttctgcaaat ctcccctatc

15541 ccctctttac taaagagcag gatttccttt agatattaac aagctatgat cgttcgaata

15601 gctttggtct ctcaatagat tcaggtaaat ggtcgatata taaggaaaat aaaaacattc

15661 gttaaagacg agcgccatct tagagaaatt tagagccaca gcgtggctca ctcagccgta

15721 ggctgatcgt aaccacgtac ggcttgccgt gcgtggctgt tacatcatga ggcgttttag

15781 ctacttcccc ctctccctgt ttttttcttc tagacgaaga aaaatctgtc cctctcccac

15841 aaggggcgag ggtgattatt tataagggca ttcgttaaag acgagcgcca tcttagagaa

15901 atttggagct acagcgtggc tcactcagcc gtaggctgat cgtaaccacg tacggcttgc

15961 cgtgcgtggc tattgggtga ttatttaaaa acaggcgaaa cggctttttt actttttcat

16021 taccgcaatt tgttttagaa tcaacggacg atttactctc aaggatttat gatgaaaaaa

16081 ttacttttag tggttttgct gacatcattc gggctaaccg cctgcggtgt aaaaggcccg

16141 ctctattttc ccgagcagca accggctcaa caacaaacaa aataattgct aaccacggta

16201 attcaaaatc tacgtataac aagcggtcgg atttatccga ttttttgtaa atacaagtgt

16261 agcgagtttg ccgtggtttt attttttcaa acacaacgga caaattaatg aatcatttca

16321 attataaaaa ccaacaactt tttgcggaag acgtttccgt ttcagacatc atcaatcaat

16381 acggtacgcc cgcttatatc tattctcgtg caacgcttga gcgccactga cacgcttttg

16441 ataaagcgtt tggctcgcac ccgcacttaa tttgttttgc ggtgaaatcc aattccaata

16501 ttgctttatt aaatgtgatg gcacgcctcg gttcgggctt tgatattgtg tcgcaaggcg

16561 aacttgaacg tgtacttgcc gccggcggcg agccgagcaa agtggtattt tccggtgtgg

16621 caaaatcaca tagcgaaatt cagcgtgcat tggaagtcgg cattcgttgc tttaatatcg

16681 aatccatcgc cgagttacac cgcattaatg aagtcgccgg tcaattaggt aaaatcgcac

16741 cgatttcatt gcgtgtaaat ccggatgtgg atgcacatac tcacccttat atttccaccg

16801 gtttaaaaga aaataaattt ggggtaagcg taacgaacga gggaaacaag tactgggaga

16861 agcgttacga gaaaaaacac aggcggaaaa cctactgcgt gaaagtgcgc taaattggac

16921 gattctccgc ccgtgcgggc tgaatacgga cgaaggcgaa acctttcgtt taattgaaaa

16981 tgcggctgaa ctgcccagca gttatatgag ccgtaaagca ttagccaatg cggtcttgtc

17041 cgtacttaac agtgaaaaca caaaccataa aatcttctca gtctgtgcct aacttcacaa

17101 tccctttcac tttggcacaa gccatccgct tgtgcctatt tcaaaatttg ttttaacagt

17161 tccaataaaa ccggtaattc cgttactccc tctcgatcga caaaatgccc gcccgttgcc

17221 aagcgaatat aatccgcttg taagtattgc gctaatcgat cgctgaacga atggggaacg

17281 acaacatcat ttaatgcaga tatgacgtaa gacttttgcg gtaaacaagc ggtctgattt

17341 gcataaaaat ctgcaaagct atctaattcc ggcaaagttg gtaatttctc ataaaagccg

17401 gaaacaaaaa ttgccgtttt tactttttgc tgcgttaccg ccagataatt cagtaacgca

17461 atgcagccca aactatgtcc gatgagtaag gtattttcat ctaattgaag tgtattttgg

17521 tgatgttcca gccatgcttg cggattcggc tgatcggaat tcggcatcgc taaacattca

17581 cattcccatc ctaatttttc caattcgttt ttaagccacg gaaaccaatt tcttgtcggg

17641 ttcgccgtat aaccgtgcgt tacatatact tttttcat

//
